# Body Fat Distribution and Risk of Breast, Endometrial, and Ovarian Cancer: A Two-Sample Mendelian Randomization Study

**DOI:** 10.3390/cancers13205053

**Published:** 2021-10-09

**Authors:** Dennis Freuer, Jakob Linseisen, Tracy A. O’Mara, Michael Leitzmann, Hansjörg Baurecht, Sebastian-Edgar Baumeister, Christa Meisinger

**Affiliations:** 1 Chair of Epidemiology, University of Augsburg, University Hospital Augsburg, 86156 Augsburg, Germany; jakob.linseisen@med.uni-augsburg.de (J.L.); christine.meisinger@med.uni-augsburg.de (C.M.); 2 Institute for Medical Information Processing, Biometry, and Epidemiology, Ludwig-Maximilians-Universität München, 81377 Munich, Germany; 3 German Research Center for Environmental Health, Independent Research Group Clinical Epidemiology, Helmholtz Zentrum München, 85764 Neuherberg, Germany; 4 Genetics and Computational Biology Department, QIMR Berghofer Medical Research Institute, Brisbane 4006, Australia; Tracy.OMara@qimrberghofer.edu.au; 5 Department of Epidemiology and Preventive Medicine, University of Regensburg, 93053 Regensburg, Germany; michael.leitzmann@klinik.uni-regensburg.de (M.L.); hansjoerg.baurecht@klinik.uni-regensburg.de (H.B.); 6 Institute of Health Services Research in Dentistry, University of Münster, 48149 Münster, Germany; sebastian.baumeister@uni-muenster.de

**Keywords:** body fat distribution, obesity, breast cancer, endometrial cancer, ovarian cancer, Mendelian randomization

## Abstract

**Simple Summary:**

The causal impact of body fat distribution on female-specific cancers is largely unknown. For the first time we used a two-sample multivariable Mendelian randomization (MR) approach to elucidate the role and causal relations of body composition assessed by segmental bioelectrical impedance analysis on the risks of breast, endometrial and ovarian cancers and their subtypes. We found that abdominal fat content increases the risk for ovarian cancer and its endometrioid and clear cell subtypes independent of overall fat content. General adiposity has a protective effect on risk of breast cancer and its ER- and ER+ subtypes but increases the risk for endometrial cancer, ovarian cancer, and the endometrioid ovarian cancer subtype. This study extends the literature by addressing specifically the causal role of visceral fat on female-specific cancers.

**Abstract:**

Background: Mounting evidence shows that adiposity increases female-specific cancer risk, but the role of body fat distribution is less clear. We used a two-sample Mendelian randomization (MR) approach to elucidate causal relations of body fat distribution to the risks of breast, endometrial and ovarian cancers and their subtypes. Methods: Body composition was assessed using segmental bioelectrical impedance analysis, yielding trunk, arm, and leg fat ratios (TFR, AFR, LFR) and BMI including 195,043 and 434,794 European women, respectively. The sample sizes for the outcomes ranged between 58,396 and 228,951. Causal effects were estimated per one standard deviation increment in the respective exposure within the radial regression framework. Robust sensitivity analyses were performed to verify MR assumptions. In a multivariable MR setting, the proportion of risk attributable to overall and abdominal fat content was assessed. Results: TFR, which represents abdominal fat content, was associated with ovarian cancer and its clear cell and endometrioid histotypes independent of overall fat content. BMI was inversely associated with breast cancer and its ER− and ER+ subtypes, but positively with endometrial cancer and ovarian cancer, including its endometrioid histotype. These estimates were confirmed using AFR as proxy for overall body fat. Conclusions: Visceral adiposity seems to be a driver of elevated ovarian cancer risk, particularly of the endometrioid and clear cell ovarian cancer histotypes. General adiposity decreases the risk of breast cancer but increases the risk of endometrial and ovarian cancer.

## 1. Introduction

Breast cancer is the most commonly diagnosed female cancer and endometrial as well as ovarian cancer incidence rank sixth and eighth, respectively, underscoring the global impact caused by these cancers [1]. Several meta-analyses and epidemiological studies implicated obesity as a player in the development of female-specific cancers [2,3,4]. In observational studies associations between body mass index (BMI) as a measure of general obesity and an increased risk of postmenopausal breast [5] and a decreasing risk of premenopausal breast cancer [6] were found. Obesity is the most established risk factor for endometrial cancer [7,8] and may be positively associated with ovarian cancer [9,10]. In recent Mendelian randomization (MR) analyses genetically predicted BMI was positively associated with endometrial cancer [11,12], but (contrary to the findings of observational studies) inversely associated with both pre- and postmenopausal breast cancer [13,14,15]. Another MR study reported that genetically predicted BMI was positively associated with non-high grade serous ovarian cancers but was unrelated to the more common and aggressive high-grade serous ovarian cancer [16].

A growing body of evidence suggests that body composition may play an important role in site-specific cancer development [17,18,19], but the data are inconclusive. Some investigations focusing on abdominal fat distribution as represented by the waist-to-hip ratio (WHR) found a positive association with breast cancer [20,21]. In a recent prospective observational study waist circumference (WC) and BMI were positively associated with endometrial and premenopausal breast cancers, but not with ovarian cancer [22]. Whether abdominal fat distribution is associated with the female-specific cancers independently of BMI remains to be clarified.

The present study is the first MR study using bioelectrical impedance analysis measurements of body fat distribution in addition to BMI as potential risk factors for female-specific cancers. We sought to generate more comprehensive and robust evidence of the impact of trunk, arm, and leg fat ratios (TFR, AFR, LFR) on breast, ovarian, as well as endometrial cancer including their histotypes based on univariable and multivariable MR analyses.

## 2. Materials and Methods

### 2.1. Study Design

A two-sample MR design allows for the assessment of a causal relationship using genome-wide association studies (GWASs) derived from different populations for the exposure and the outcome of interest. Three key assumptions need to be satisfied in a MR study: genetic instruments (i) must be associated with the respective risk factor; (ii) must not be associated with any measured or unmeasured confounder of the exposure-outcome association; and (iii) must influence the outcome only through the exposure. Details on the MR-design can be found elsewhere [23,24]. In the present study, we used both a univariable MR approach to investigate overall effects of general and abdominal fat content on female-specific cancers and a multivariable MR (MVMR) setting to investigate the direct effects simultaneously. Analyses were based on women of European ancestry as the underlying population for exposures and outcomes.

Our analytic approach included: (i) extraction of potential genetic instruments; (ii) conducting univariable and multivariable MR analyses to assess overall and direct effects; (iii) applying several sensitivity analyses to assess different patterns of pleiotropy and validate MR assumptions.

### 2.2. Exposures Definition and Data Availability

Regarding the amount of overall fat and for comparisons with prior MR-studies, we used BMI. To discriminate fat stored in different parts of the body, we used the fat ratio indexes TFR, AFR, LFR assessed by a segmental body impedance analysis (BIA) and calculated by dividing the fat mass in the respective body segments with total body fat mass [25]. Segmental BIA measurement is a more precise method than common anthropometric measures to discriminate between adipose and lean mass and regarding fat storage in different body compartments. Due to a strong correlation [25], AFR could be used as a measure for overall fat content and therefore replicate the results of BMI, while TFR could be utilized for investigating the impact of visceral fat content on female-specific cancers.

Genetic instruments for BMI were obtained from a GWAS (GIANT consortium and UK Biobank) of 434,794 European women [26] (Table 1). The GWAS of fat ratio indexes TFR, AFR, and LFR was derived from 195,043 female UK Biobank participants [25].

### 2.3. Outcomes Definition and Data Availability

As outcomes we considered breast, ovarian, and endometrial cancer. Additionally, we performed subgroup analyses of ER-positive (ER+) and ER-negative (ER−) breast cancer subtypes and the histotypes clear cell, endometrioid as well as high and low grade serous epithelial ovarian cancers.

The breast cancer GWAS was obtained from BCAC with 122,977 cases (thereof 69,501 ER+ and 21,468 ER−) and 105,974 controls [27] (Table 2). The ovarian cancer GWAS was attained from OCAC (25,509 cases and 40,941 controls) [28]. For the subgroup analyses 1366 clear cell, 2810 endometrioid, and 14,049 high or low grade serous ovarian cancer cases were available. Endometrial cancer GWAS was obtained using studies identified via the ECAC and E2C2 excluding subjects from UK Biobank with 12,270 cases and 46,126 controls [12]. There were no sample overlaps between the exposure and outcome datasets, minimizing the weak instrument bias.

### 2.4. Selection of Genetic Instruments

We selected single nucleotide polymorphisms (SNPs) associated with the respective body composition measure below the genome-wide association threshold of $p=5\times 10^{-8}$ and an imputation score >0.8. Independence of the genetic variants was ensured by performing a clumping algorithm using the stringent linkage disequilibrium (LD) cut-off $r^{2}=0.001$. For instruments missing from an outcome dataset proxy-SNPs with an LD $r^{2}>0.8$ were used.

### 2.5. Statistical Power

We calculated the statistical power according to Brion et al. [29] dependent on unknown true odds ratios (ORs) for each outcome in the range of 0.6 and 1.4 and using the type I error rate α=0.05, outcome-specific proportions of cases and the mean explained phenotypic variance of exposures by genetic instruments ($R^{2}=0.05$) (Figure S1).

### 2.6. Statistical Analyses

#### 2.6.1. Univariable MR

As principal analysis, we performed the radial inverse-variance weighted (IVW) regression with modified second-order weights. This approach provides large statistical power and allows even for balanced pleiotropy (i.e., zero mean of random pleiotropic effects). To account for directional horizontal pleiotropy, we performed radial MR-Egger regression with modified second-order weights and tested the deviation from zero applying Egger’s intercept test (Table S1).

Since the analyses were carried out iteratively, in each step we calculated SNP-specific Q-statistics and performed a leave-one-out-analysis to identify and remove outliers. Additionally, several global heterogeneity statistics were calculated and tested regarding the appropriate methods for the relationship between body composition and cancer types (Table S1).

Potential horizontal pleiotropy was assessed using robust MR approaches. The weighted median method provides a consistent estimate even if up to 50% of selected genetic instruments are invalid. The MR-PRESSO approach consists of three components, namely a global, outlier, and distortion test. The global test identifies horizontal pleiotropy based on the observed residual sum of squares (RSSobs). The outlier test corrects, if present, horizontal pleiotropy via outlier removal identified by SNP-specific RSSobs. The distortion test compares estimates before and after outlier removal. A many weak instrument analysis was done using the Robust Adjusted Profile Score, allowing instruments to be weak.

#### 2.6.2. Multivariable MR

To compare the direct effects and separate out what proportion of female-specific cancer risk is due to general and visceral fat content, we performed MVMR analyses. AFR and TFR were utilized as proxies for the degree of overall and abdominal fat content, respectively. The conditional F-statistics were at least 25.9 implicating absence of weak instrument bias. Horizontal pleiotropy was quantified and tested using the modified Cochran’s Q-statistics, while directional pleiotropy was investigated using MR-Egger intercept tests (Table S2). Causal effect estimates were obtained using the robust IVW approach with multiplicative random effects as the main method and Median and MR-Egger methods as sensitivity analyses. Effect estimation after outlier assessment and deletion was done by the MR-Lasso procedure. Finally, we used the Q-minimization approach to calculate robust point estimates that account for both weak instruments and substantial heterogeneity.

All estimates depict ORs per one standard deviation (SD) increments in the exposures. The type I error was set to α = 0.01 when investigating outliers based on Q-statistics within the radial framework and to α = 0.05 otherwise. To correct for multiple testing the Benjamini-Hochberg procedure was applied. Analyses were performed using mainly the packages TwoSampleMR (0.5.5), MendelianRandomization (0.4.3), RadialMR (1.0), and MRPRESSO (1.0) of the statistical Software R (Version: 4.0.3) (the R Foundation is seated in Vienna, Austria).

## 3. Results

### 3.1. Instrument Strength and Statistical Power

As genetic instruments we selected 297 BMI-, 202 TFR-, 116 AFR-, and 166 LFR-related SNPs (Table 1). For BMI 4.6% of the phenotypic variance was explained by the genetic instruments. For TFR, AFR, and LFR the explained variances were 6.8%, 3.1%, and 5.1%, respectively. The SNP-specific F-statistics ranging from 29.1 (BMI) to 941.4 (BMI) (Tables S3–S34), were clearly above the traditional threshold of 10, indicating absence of weak instrument bias. We determined a statistical power ≥ 0.8 when the true ORs for the impact of body fat indexes on breast, ovarian, and endometrial cancers were expected to be ≤0.95 or ≥1.05, ≤0.90 or ≥1.10, ≤0.88 or ≥1.12, respectively. The statistical power in the subgroup analyses were slightly lower for ER+ and ER− breast cancers and (high or low grade) serous ovarian cancer.

### 3.2. Effects of Body Fat on Breast Cancer and Its Subtypes

**Figure 1:**
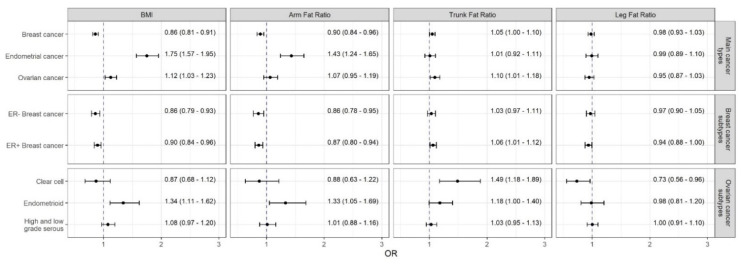
Causal estimates (odds ratios and 95% confidence intervals per one standard deviation change in the respective exposure) of the impact of obesity measures on female-specific cancers obtained by the radial inverse-variance weighted (IVW) approach with modified second-order weights. Abbreviations: FE, fixed effects; IVW (Mod. 2nd), inverse-variance weighted model with modified second-order weights; RAPS, Robust Adjusted Profile Score; RE, random effects; PRESSO, Pleiotropy RESidual Sum and Outlier.

The Radial IVW models with modified second-order weights as the main method (point estimates are subsequently referred to as OR_IVW and are given per 1 SD increment of the respective exposure) found an inverse association between BMI and breast cancer (${\mathrm{OR}}_{\mathrm{IVW}}$ = 0.86; 95% CI: [0.81, 0.91]) (Figure 1). These results were verified using the AFR as proxy for general fat content (${\mathrm{OR}}_{\mathrm{IVW}}$ = 0.90; 95% CI: [0.84, 0.96]). However, TFR as a proxy for visceral fat was positively but weakly associated with breast (${\mathrm{OR}}_{\mathrm{IVW}}$ = 1.05; 95% CI: [1.00, 1.10]). All corresponding scatterplots of the SNP-effects on both the exposures and outcomes can be found at Figures S2–S5.

Although there was evidence for substantial heterogeneity (suggesting the Radial MR-Egger with multiplicative random effects as the appropriate method) (Table S1), the strong results (i.e., impact of overall fat content on breast cancer) were supported by sensitivity analyses using robust methods (Figure S6).

**Figure 2:**
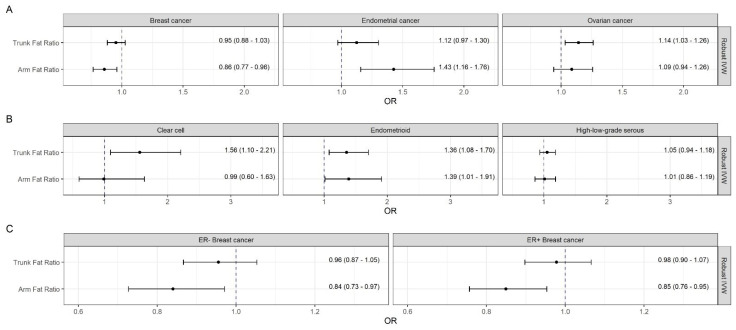
Mutually adjusted causal estimates (odds ratios and 95% confidence intervals per one SD change in the appropriate exposure) from multivariable Mendelian randomization analyses of genetically predicted trunk and arm fat ratios on female-specific cancers. Abbreviations: IVW, inverse-variance weighted; OR, odds ratio.

After mutual adjustment, the inverse effect of AFR with breast cancer (Robust IVW OR = 0.86; 95% CI: [0.77, 0.96]) did not changed remarkably, while the positive association between TFR and breast cancer attenuated in the multivariable setting (Figure 2).

Subgroup analyses revealed consistent inverse associations of genetically predicted BMI and AFR with ER− (${\mathrm{OR}}_{\mathrm{IVW}}$ = 0.86; 95% CI: [0.79, 0.93] and ${\mathrm{OR}}_{\mathrm{IVW}}$ = 0.86; 95% CI: [0.78, 0.95]) as well as ER+ breast cancer (${\mathrm{OR}}_{\mathrm{IVW}}$ = 0.90; 95% CI: [0.84, 0.96] and ${\mathrm{OR}}_{\mathrm{IVW}}$ = 0.87; 95% CI: [0.80, 0.94]) (Figure 1) despite heterogeneity in the relationships with the latter. TFR was positively and LFR inversely related to ER+ (with inconsistent Egger estimates) but not to ER− breast cancer (Figure S7).

The multivariable analyses confirmed the protective effects of AFR on ER− (Robust IVW OR = 0.84; 95% CI: [0.73, 0.97]) and ER+ breast cancer (Robust IVW OR = 0.85; 95% CI: [0.76, 0.95]), while the formerly association between TFR and ER+ breast cancer could not be substantiated.

### 3.3. Effects of Body Fat on Endometrial Cancer

Both genetically predicted BMI and AFR were robustly associated with endometrial cancer with ${\mathrm{OR}}_{\mathrm{IVW}}$ = 1.75 (95% CI: [1.57, 1.95]) and ${\mathrm{OR}}_{\mathrm{IVW}}$ = 1.43 (95% CI: [1.24, 1.65]), respectively (Figure 1). These effects were supported by the multivariable analysis, where the effect of AFR on endometrial cancer were adjusted for TFR (Robust IVW OR = 1.43; 95% CI: [1.16, 1.76]) (Figure 2). No further notable associations were observed.

### 3.4. Effects of Body Fat on Ovarian Cancer and Its Histotypes

Although there was not a clear association with ovarian cancer, consistently positive relations were observed of both BMI and AFR to endometrioid ovarian cancer, with ${\mathrm{OR}}_{\mathrm{IVW}}$ = 1.34 (95% CI: [1.11, 1.62]) and ${\mathrm{OR}}_{\mathrm{IVW}}$ = 1.33 (95% CI: [1.05, 1.69]), respectively (Figure 1). Regarding the visceral fat content, genetically predicted TFR was positively associated with ovarian cancer (${\mathrm{OR}}_{\mathrm{IVW}}$ = 1.10; 95% CI: [1.01, 1.18]) as well as the clear cell (${\mathrm{OR}}_{\mathrm{IVW}}$ = 1.49; 95% CI: [1.18, 1.89]), and endometrioid histotypes (${\mathrm{OR}}_{\mathrm{IVW}}$ = 1.18; 95% CI: [1.00, 1.40]. Apart from an inverse association between LFR and clear cell ovarian cancer (${\mathrm{OR}}_{\mathrm{IVW}}$ = 0.73; 95% CI: [0.56, 0.96]), there were no further notable relations. Again, the sensitivity analyses supported these findings (Figure S8).

In the multivariable setting, the association between AFR and the endometrioid ovarian cancer remained positive (Robust IVW OR = 1.39; 95% CI: [1.01, 1.91]). The effect of TFR on ovarian cancer and its clear cell and endometrioid histotypes were partly even stronger with Robust IVW OR = 1.14 (95% CI: [1.03, 1.26]), OR = 1.56 (95% CI: [1.10, 2.21]), and OR = 1.36 (95% CI: [1.08, 1.70]), respectively.

Although no directional pleiotropy was detected by MR-Egger intercept tests, there was evidence for substantial heterogeneity quantified by Q-statistics in the multivariable models (Table S2). However, all results were supported by sensitivity analyses (Figures S9–S11). Especially the heterogeneity robust Q-minimization models produced consistent point estimates to the robust IVW approach as the main method.

## 4. Discussion

Our MR study showed that visceral adiposity increased the risk of breast cancer, mainly ER+ breast cancer, and the risk of ovarian cancer, mainly clear cell ovarian cancer. Relations of genetically predicted visceral adiposity to breast and endometrial cancers were weaker than those with general adiposity. However, visceral adiposity appears to play a more important role than general adiposity for the risk of ovarian cancer, in particular the clear cell and endometrioid cancer subtypes. The findings confirmed a causal protective effect of general adiposity on breast cancer risk. This effect could be ascribed to associations with both ER- and ER+ breast cancers and was confirmed in multivariable analyses. In contrast, general adiposity was a strong causal risk factor for endometrial cancer and a weaker causal risk factor for ovarian cancer, in particular for the endometrioid histotype. LFR was not related to overall cancers but showed effects in the same direction as AFR on ER+ breast cancer and clear cell ovarian cancer.

Epidemiologic studies reported a positive relationship between obesity and postmenopausal breast cancer and an inverse association with premenopausal breast cancer [30,31,32,33]. One study using data from two chemoprevention trials found a positive association in premenopausal women [34]. In contrast, two prior MR studies showed that adult BMI is inversely related to postmenopausal breast cancer [13,14]. We also found an inverse causal effect of overall fat content on breast cancer risk; however, we were unable to distinguish between pre- and postmenopausal breast cancers. The apparent adverse effect of adult body size on breast cancer risk may be attributable to a large body size in childhood that persists into adulthood [35]. Of note, childhood BMI showed inverse associations with both premenopausal and postmenopausal breast cancer [36,37]. Because adult weight gain is a strong predictor of postmenopausal breast cancer risk [38], the positive association between adult BMI and postmenopausal breast cancer shown in epidemiologic studies is likely due to the effect of weight gain in adulthood. Weight gain may better reflect the dynamics of changes in body shape over time, with increased visceral and metabolically active fat accumulation, especially during menopause [39]. According to the WCRF, abdominal obesity and weight gain in adulthood convincingly increases the risk of postmenopausal breast cancer [31]. Our finding that genetically predicted TFR showed a positive effect on ER+ breast cancer suggests that body fat distribution plays a particular role in postmenopausal breast cancer. Supporting the assessment of the IARC Working Group, [30], we showed that an increasing BMI and AFR had a strongly positive effect on endometrial cancer. However, we found no evidence for a causal link between abdominal adiposity and endometrial cancer; this is in contrast to umbrella reviews [3,4,31], in which the association between WC and WHR and total endometrial cancer was supported by strong evidence. Our results support the findings of recent MR studies, where genetically elevated BMI, but not WHR, was found to be causally related to endometrial cancer risk [11,40].

The evidence relating body fatness to ovarian cancer and its subtypes is inconclusive [41]. The WCRF CUP graded the relationship as probably causal [31] and the IARC suggested there is strong evidence for a positive association between obesity and ovarian cancer [30]. Likewise, a systematic review and meta-analysis reported an increased risk of ovarian cancer with higher BMI, and a marginally significant positive association with WC, but no association with hip circumference or WHR [42]. An umbrella review of dose-response meta-analyses rated the evidence for an association between BMI and ovarian cancer as weak [43], but another umbrella review [2] rated the evidence as suggestive only [2]. Using a MR approach, Gao et al. [14] found a positive association between BMI and ovarian cancer, which is in line with our results; contrary, a one-sample MR study found no association between both BMI and fat mass and ovarian cancer [44]. However, our study was two-sampled, included more cases, and used larger GWAS with 201 more genetic instruments for the analyses resulting in higher statistical power. We further showed a positive association between TFR and ovarian cancer, in particular for the endometrioid and clear cell histotypes, a result that extends the existing evidence and may contribute to better understanding of the pathogenesis of this cancer.

Obese adipose tissue seems to create a pro-oncogenic environment. Specific biological mechanisms to explore the link between obesity and cancer risk are centered along metabolic and endocrine consequences of body fat accumulation. Changes in the metabolism of sex hormones, insulin and insulin-like growth factor signaling, inflammatory effects and Adipokine-related effects were identified. According to an IARC working group, the evidence for a role of sex hormones and chronic inflammation was judged as strong, while the evidence for insulin and IGF was moderate [30]. Among other factors, obesity-related changes in the tumor microenvironment, cellular perturbations, and the intestinal microbiome are likely to affect cancer development [45]. In addition, obesity-related changes in anti-tumor immunity and angiogenesis were observed [46]. Biological mechanisms that could explain the inverse effects of BMI and AFR on the development of breast cancer and its subtypes are widely unknown.

Our study has a number of considerable strengths. Using both BMI and AFR we could replicate the findings of a previous MR study, which provided support for our estimates. While BMI approximates total fat content, the fat ratio indexes used in our study discriminated between adipose and lean mass and are therefore more reflective of actual fat mass and body fat distribution. All exposures were modelled as continuous variables to avoid residual confounding and to boost statistical power. Sufficiently large F-statistics reduced the potential for weak instrument bias. The broad range of robust methods allowed us to strengthen our findings by considering different patterns of pleiotropy. The multivariable approach allowed us to distinguish between the direct effects of overall and abdominal fat content.

Our study also has certain limitations. TFR considers the fat content of the entire torso rather than that of the abdomen only, which may have underestimated causal associations. MR analyses assume relationships to be linear. Quantitative estimates may be misleading if the true relationship is non-linear; although estimates are still reflective of the presence and direction of the population-averaged causal effect [47]. Our analyses were based on European women and thus, results cannot be transferred to other ethnicities. Finally, we were unable to distinguish between pre- and postmenopausal breast cancer.

## 5. Conclusions

In conclusion, our MR study shows a protective effect of general adiposity on risk of breast cancer and its ER- and ER+ subtypes. In contrast, general adiposity increases the risk for endometrial cancer, ovarian cancer, and the endometrioid ovarian cancer subtype. Visceral adiposity is related to an increased risk for ovarian cancer and its endometrioid and clear cell subtypes and is suggestively associated with increased risk of breast cancer and its ER+ subtype. These results extend the literature by addressing the role of visceral adiposity regarding female-specific cancers and support previous findings on general adiposity. Thus, it can be concluded that different body fat distributions lead to different risk profiles and not obesity per se, but rather body fat distribution should be taken into consideration in connection with the risk of female-specific cancers.

## Figures and Tables

**Table 1:** Overview of genome-wide association studies used in the analyses for the exposures and characteristics of the Single Nucleotide Polymorphisms (SNPs) considered as instrumental variables.

Characteristics	BMI	AFR	TFR	LFR
Sample size	434,794	195,043	195,043	195,043
Consortium	GIANT, UK Biobank	UK Biobank	UK Biobank	UK Biobank
Number of genetic instruments ^a^	297	116	202	166
Explained variance by instruments	4.63%	3.13%	6.77%	5.10%
F-statistic, mean (min; max)	60.66 (29.07; 941.37)	51.37 (29.73; 380.64)	63.9 (29.84; 392.04)	58.51 (29.73; 340.77)
Reference	Pulit et al., 2018 [26]	Rask-Andersen et al., 2019 [25]	Rask-Andersen et al., 2019 [25]	Rask-Andersen et al., 2019 [25]

^a^ Extraction of genetic instruments based on the threshold $p=5\times 10^{-8}$, clumping cutoff $r^{2}=0.001$.

**Table 2:** Overview of genome-wide association studies used in the analyses for the outcomes.

Characteristics	Breast Cancer		Endometrial Cancer	Ovarian Cancer		
Sample size	228,951		58,396	66,450		
Controls	105,974		46,126	40,941		
Cases	122,977		12,270	25,509		
Subtypes	ER−	ER+		Clear cell	Endometrioid	Low or high grade serous
Cases	21,468	69,501		1366	2810	14,049
Consortium	BCAC		ECAC, E2C2 ^a^	OCAC		
Reference	Michailidou et al., 2018 [27]		O’Mara et al., 2019 [12]	Phelan et al., 2017 [28]		

^a^ excluding UK Biobank participants.

## Data Availability

The present study is based on summary-level data that have been made publically available. Summary data from genome-wide association studies for the BMI (Pulit et al.) is available at https://zenodo.org/record/1251813#.XxgQ2J5KiUl (accessed on 26 March 2021); Data for the ratios TFR, AFR, and LFR (Rask-Andersen et al.) can be obtained from https://myfiles.uu.se/ssf/s/readFile/share/3993/1270878243748486898/publicLink/GWAS_summary_stats_ratios.zip (accessed on 12 March 2021); Summary level data for breast cancer and its subgroups can be obtained from the BCAC Consortium (http://bcac.ccge.medschl.cam.ac.uk/bcacdata/oncoarray/oncoarray-and-combined-summary-result/gwas-summary-results-breast-cancer-risk-2017) (accessed on 3 February 2021) and for ovarian cancer and its subgroups from MRC IEU OpenGWAS Project database (https://gwas.mrcieu.ac.uk) (accessed on 10 February 2021). Endometrial cancer summary data (O’Mara et al.) restricted to subjects apart from UK Biobank is available on request from authors.

## Supplementary Materials

The following are available online at https://www.mdpi.com/article/10.3390/cancers13205053/s1, Figure S1: Power analysis, Figure S2: Sensitivity analyses of univariable Mendelian randomization for main cancer types, Figure S3: Sensitivity analyses of univariable Mendelian randomization for breast cancer subtypes, Figure S4: Sensitivity analyses of univariable Mendelian randomization for ovarian cancer subtypes, Figure S5: Sensitivity analyses of multivariable Mendelian randomization for main cancer types, Figure S6: Sensitivity analyses of multivariable Mendelian randomization for breast cancer subtypes, Figure S7: Sensitivity analyses of multivariable Mendelian randomization for ovarian cancer subtypes, Figure S8: Scatter plot matrix of SNP effects on both body mass index and female-specific cancers, Figure S9: Scatter plot matrix of SNP effects on both arm fat ratio and female-specific cancers, Figure S10: Scatter plot matrix of SNP effects on both trunk fat ratio and female-specific cancers, Figure S11: Scatter plot matrix of SNP effects on both leg fat ratio and female-specific cancers, Table S1: Heterogeneity statistics from univariable Mendelian randomization analyses, Table S2: Instrument strength and heterogeneity from multivariable Mendelian randomization, Table S3: BMI related SNP-associations with breast cancer, Table S4: BMI related SNP-associations with endometrial cancer, Table S5: BMI related SNP-associations with ovarian cancer, Table S6: BMI related SNP-associations with ER- breast cancer, Table S7: BMI related SNP-associations with ER+ breast cancer, Table S8: BMI related SNP-associations with clear cell ovarian cancer, Table S9: BMI related SNP-associations with endometrioid ovarian cancer, Table S10: BMI related SNP-associations with high or low serous ovarian cancer, Table S11: Arm fat ratio related SNP-associations with breast cancer, Table S12: Arm fat ratio related SNP-associations with endometrial cancer, Table S13: Arm fat ratio related SNP-associations with ovarian cancer, Table S14: Arm fat ratio related SNP-associations with ER- breast cancer, Table S15: Arm fat ratio related SNP-associations with ER+ breast cancer, Table S16: Arm fat ratio related SNP-associations with clear cell ovarian cancer, Table S17: Arm fat ratio related SNP-associations with endometrioid ovarian cancer, Table S18: Arm fat ratio related SNP-associations with high or low serous ovarian cancer, Table S19: Trunk fat ratio related SNP-associations with breast cancer, Table S20: Trunk fat ratio related SNP-associations with endometrial cancer, Table S21: Trunk fat ratio related SNP-associations with ovarian cancer, Table S22: Trunk fat ratio related SNP-associations with ER- breast cancer, Table S23: Trunk fat ratio related SNP-associations with ER+ breast cancer, Table S24: Trunk fat ratio related SNP-associations with clear cell ovarian cancer, Table S25: Trunk fat ratio related SNP-associations with endometrioid ovarian cancer, Table S26: Trunk fat ratio related SNP-associations with high or low serous ovarian cancer, Table S27: Leg fat ratio related SNP-associations with breast cancer, Table S28: Leg fat ratio related SNP-associations with endometrial cancer, Table S29: Leg fat ratio related SNP-associations with ovarian cancer, Table S30: Leg fat ratio related SNP-associations with ER- breast cancer, Table S31: Leg fat ratio related SNP-associations with ER+ breast cancer, Table S32: Leg fat ratio related SNP-associations with clear cell ovarian cancer, Table S33: Leg fat ratio related SNP-associations with endometrioid ovarian cancer, Table S34: Leg fat ratio related SNP-associations with high or low serous ovarian cancer.
