# Supplementary material for: Body Fat Distribution and Risk of Breast, Endometrial, and Ovarian Cancer: A Two-Sample Mendelian Randomization Study
Source: Cancers (Basel). 2021 Oct 9;13(20):5053. doi: 10.3390/cancers13205053 (PMC8534230; doi:10.3390/cancers13205053)
Supplement: Supplementary file 1 [file cancers-13-05053-s001.zip › cancers-1350215-supplementary.pdf]

cancers MDPI

Supplementary Material: Body Fat Distribution and Risk of Breast, Endometrial, and Ovarian Cancer: A Two-Sample Mendelian Randomization Study

Dennis Freuer, Jakob Linseisen, Tracy A. O'Mara, Michael Leitzmann, Hansjörg Baurecht, Sebastian-Edgar Baumeister and Christa Meisinger

Table of contents

Figures

Figure S1	Power analysis
Figure S2	Scatter plot matrix of SNP effects on both body mass index and female-specific cancers
Figure S3	Scatter plot matrix of SNP effects on both arm fat ratio and female-specific cancers
Figure S4	Scatter plot matrix of SNP effects on both trunk fat ratio and female-specific cancers
Figure S5	Scatter plot matrix of SNP effects on both leg fat ratio and female-specific cancers
Figure S6	Sensitivity analyses of univariable Mendelian randomization for main cancer types
Figure S7	Sensitivity analyses of univariable Mendelian randomization for breast cancer subtypes
Figure S8	Sensitivity analyses of univariable Mendelian randomization for ovarian cancer subtypes
Figure S9	Sensitivity analyses of multivariable Mendelian randomization for main cancer types
Figure S10	Sensitivity analyses of multivariable Mendelian randomization for breast cancer subtypes
Figure S11	Sensitivity analyses of multivariable Mendelian randomization for ovarian cancer subtypes

Tables

Table S1	Heterogeneity statistics from univariable Mendelian randomization analyses
Table S2	Instrument strength and heterogeneity from multivariable Mendelian randomization
Table S3	BMI related SNP-associations with breast cancer
Table S4	BMI related SNP-associations with endometrial cancer
Table S5	BMI related SNP-associations with ovarian cancer
Table S6	BMI related SNP-associations with ER- breast cancer
Table S7	BMI related SNP-associations with ER+ breast cancer
Table S8	BMI related SNP-associations with clear cell ovarian cancer
Table S9	BMI related SNP-associations with endometrioid ovarian cancer

Table S10	BMI related SNP-associations with high or low serous ovarian cancer
Table S11	Arm fat ratio related SNP-associations with breast cancer
Table S12	Arm fat ratio related SNP-associations with endometrial cancer
Table S13	Arm fat ratio related SNP-associations with ovarian cancer
Table S14	Arm fat ratio related SNP-associations with ER- breast cancer
Table S15	Arm fat ratio related SNP-associations with ER+ breast cancer
Table S16	Arm fat ratio related SNP-associations with clear cell ovarian cancer
Table S17	Arm fat ratio related SNP-associations with endometrioid ovarian cancer
Table S18	Arm fat ratio related SNP-associations with high or low serous ovarian cancer
Table S19	Trunk fat ratio related SNP-associations with breast cancer
Table S20	Trunk fat ratio related SNP-associations with endometrial cancer
Table S21	Trunk fat ratio related SNP-associations with ovarian cancer
Table S22	Trunk fat ratio related SNP-associations with ER- breast cancer
Table S23	Trunk fat ratio related SNP-associations with ER+ breast cancer
Table S24	Trunk fat ratio related SNP-associations with clear cell ovarian cancer
Table S25	Trunk fat ratio related SNP-associations with endometrioid ovarian cancer
Table S26	Trunk fat ratio related SNP-associations with high or low serous ovarian cancer
Table S27	Leg fat ratio related SNP-associations with breast cancer
Table S28	Leg fat ratio related SNP-associations with endometrial cancer
Table S29	Leg fat ratio related SNP-associations with ovarian cancer
Table S30	Leg fat ratio related SNP-associations with ER- breast cancer
Table S31	Leg fat ratio related SNP-associations with ER+ breast cancer
Table S32	Leg fat ratio related SNP-associations with clear cell ovarian cancer
Table S33	Leg fat ratio related SNP-associations with endometrioid ovarian cancer
Table S34	Leg fat ratio related SNP-associations with high or low serous ovarian cancer

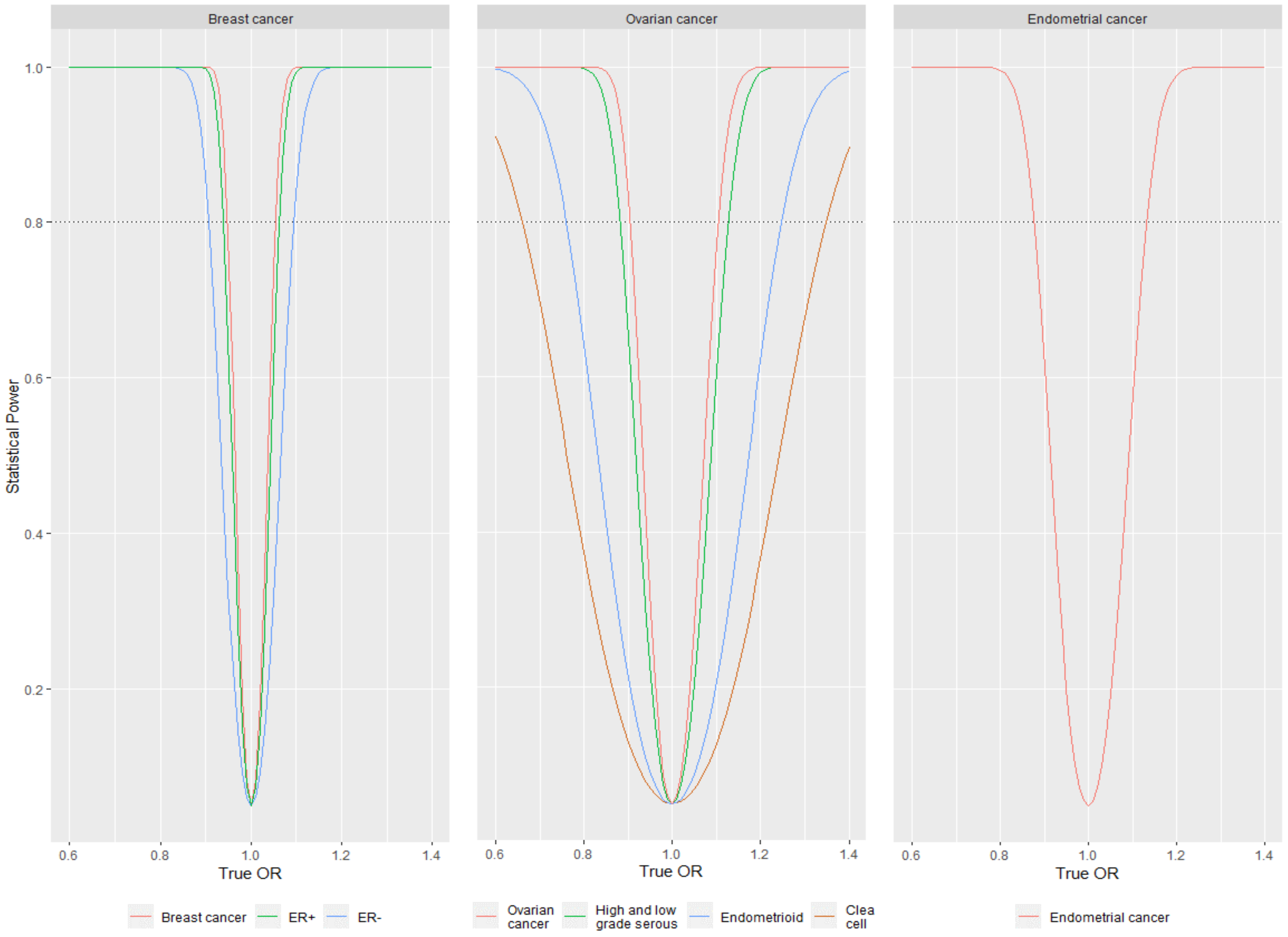

Figure S1. Mendelian randomization power analysis according to Brion et al. The dotted line represents a power of 0.8.

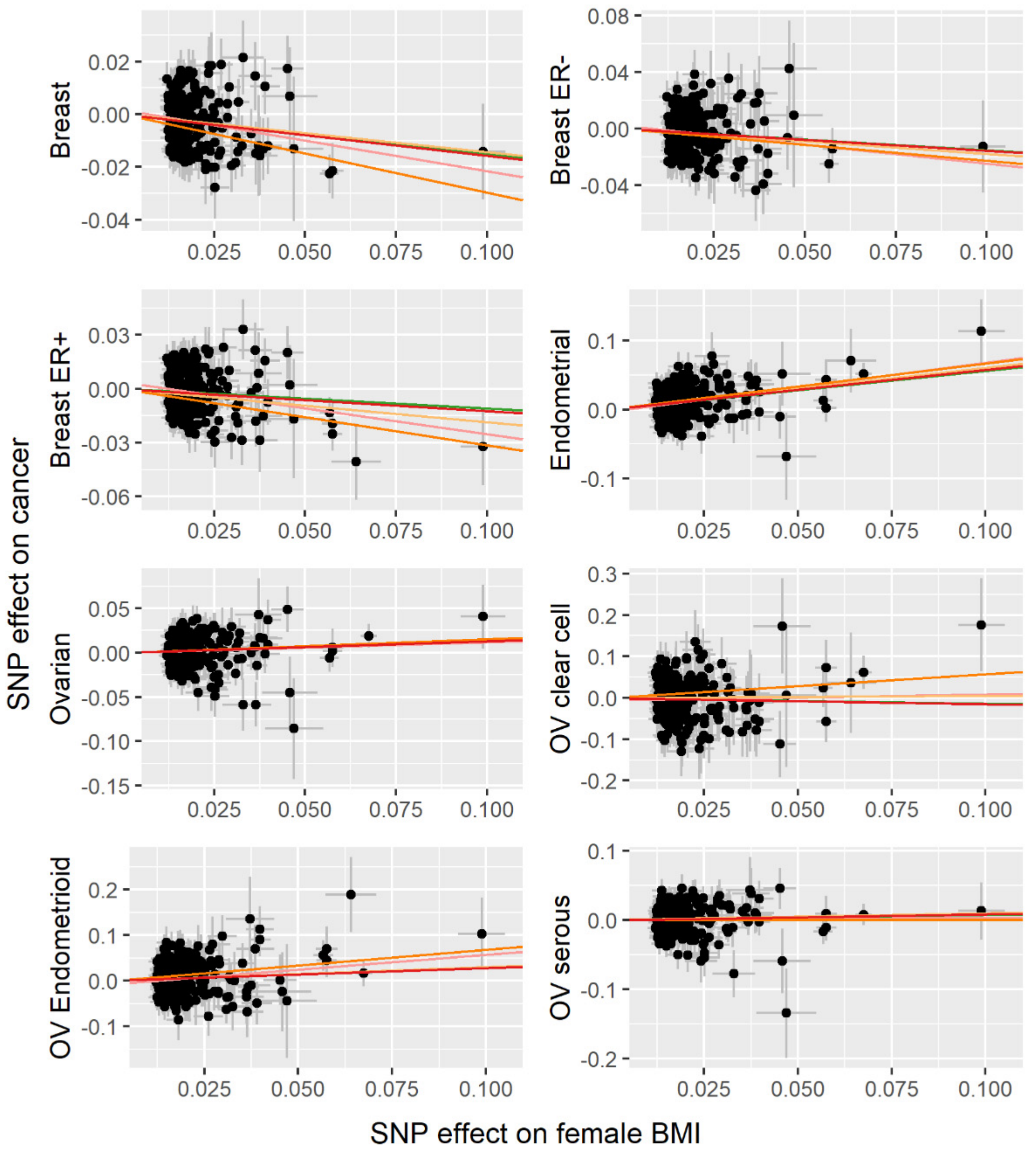

Figure S2. Scatter plot matrix of SNP effects on both body mass index (BMI) and female-specific cancers. Presented regression lines arised from different Mendelian randomization methods. Abbreviations: SNPs, Single Nucleotide Polymorphisms; OV, ovarian.

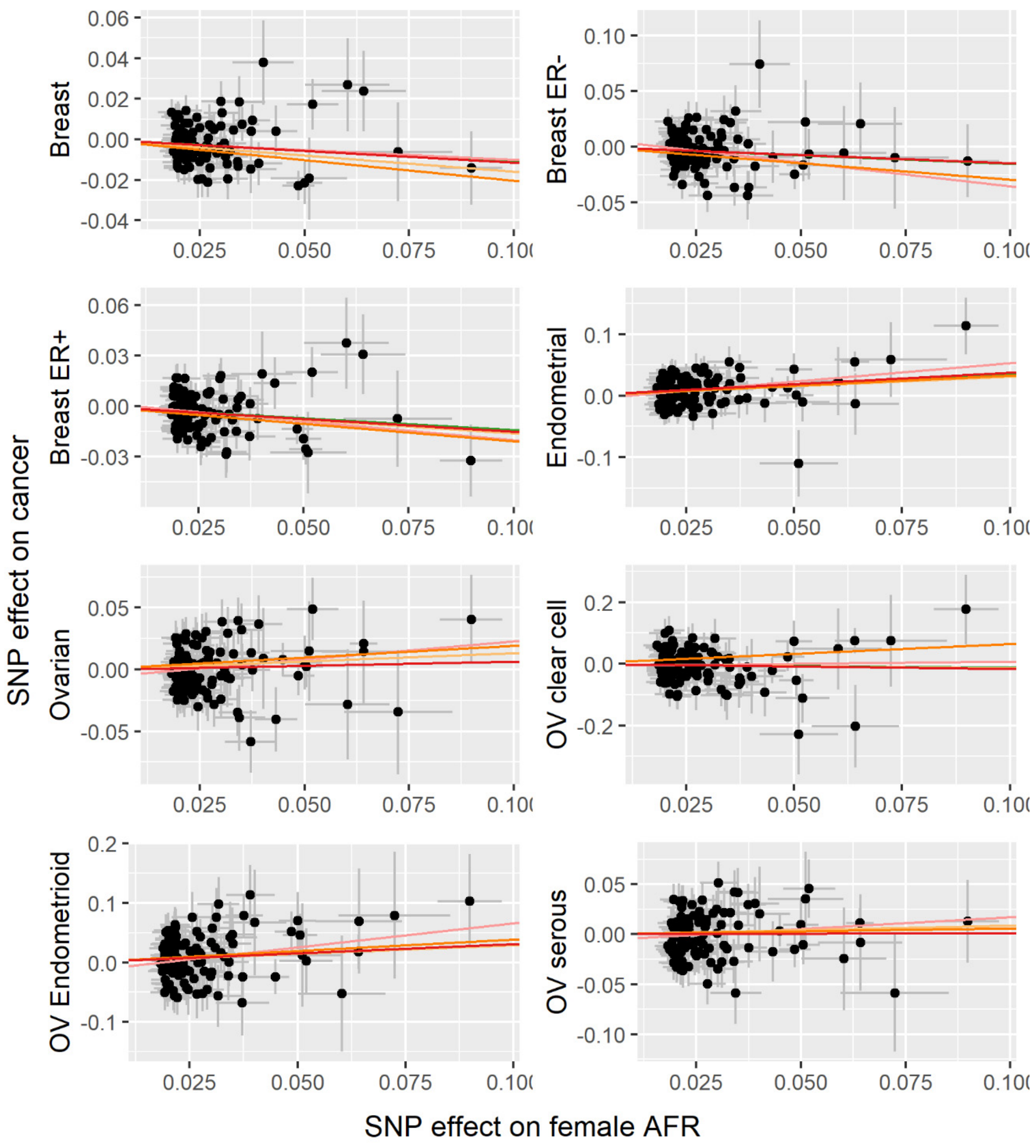

Figure S3. Scatter plot matrix of SNP effects on both arm fat ratio (AFR) and female-specific cancers. Presented regression lines arised from different Mendelian randomization methods. Abbreviations: SNPs, Single Nucleotide Polymorphisms; OV, ovarian.

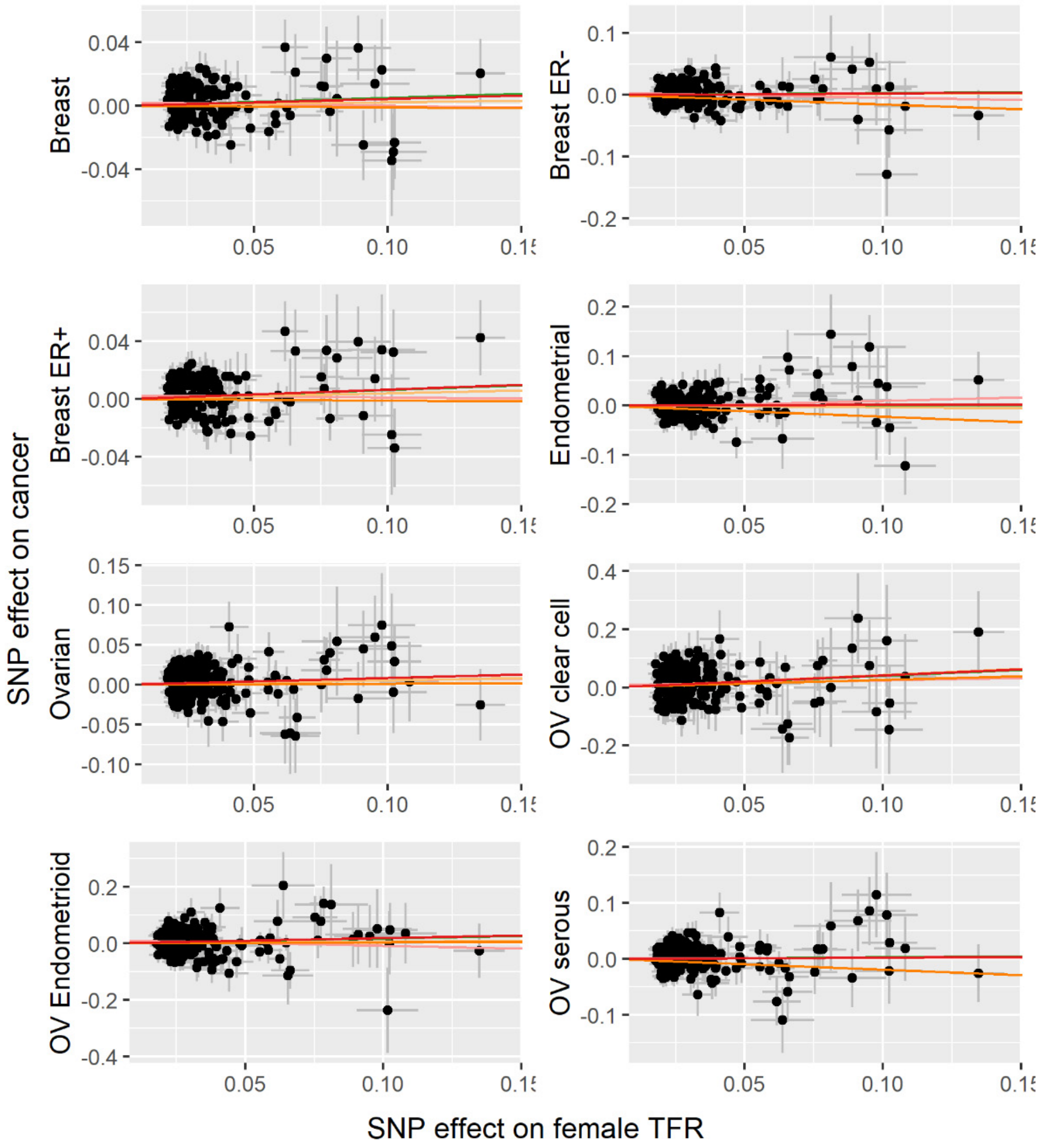

Figure S4. Scatter plot matrix of SNP effects on both trunk fat ratio (TFR) and female-specific cancers. Presented regression lines arised from different Mendelian randomization methods. Abbreviations: SNPs, Single Nucleotide Polymorphisms; OV, ovarian.

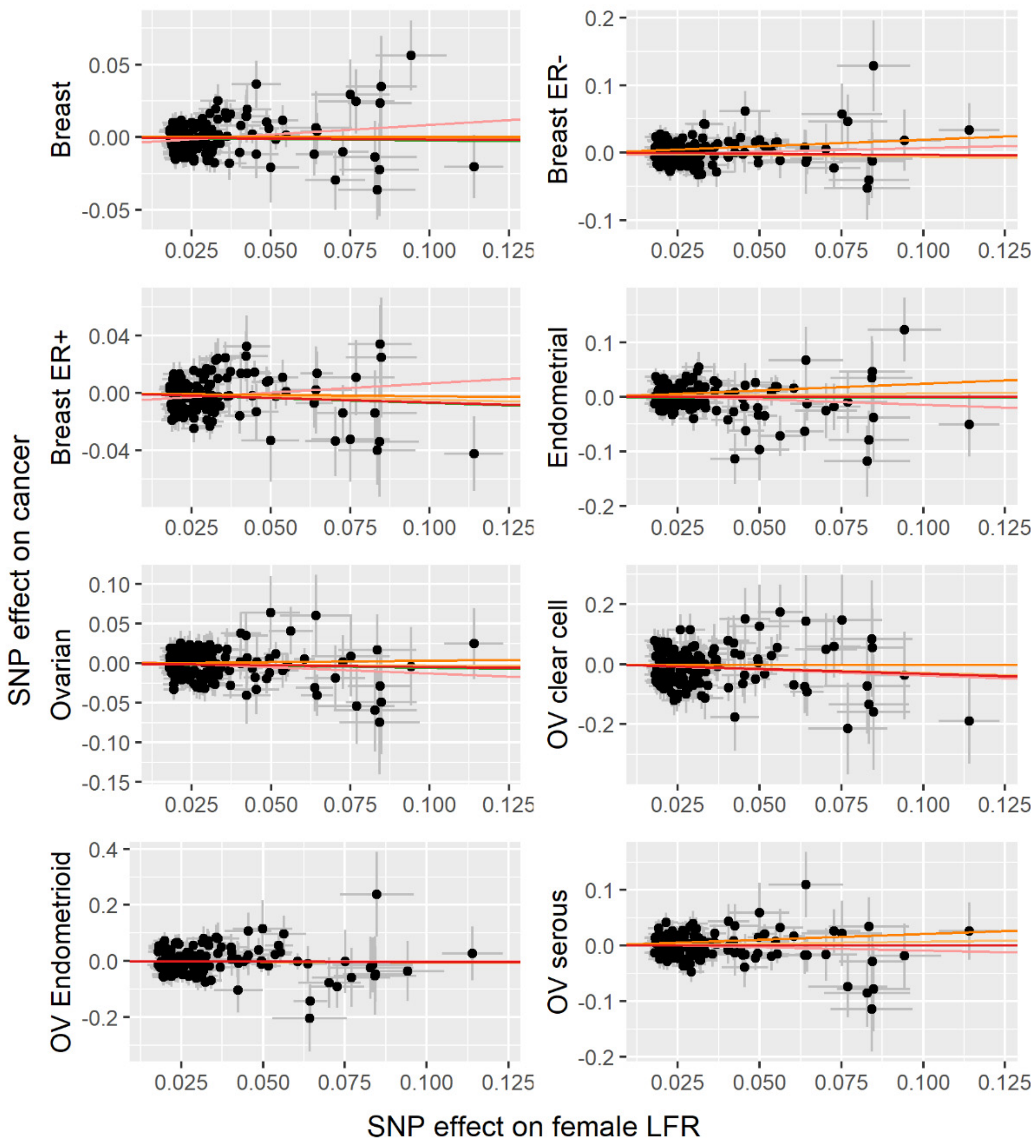

Figure S5. Scatter plot matrix of SNP effects on both leg fat ratio (LFR) and female-specific cancers. Presented regression lines arised from different Mendelian randomization methods. Abbreviations: SNPs, Single Nucleotide Polymorphisms; OV, ovarian.

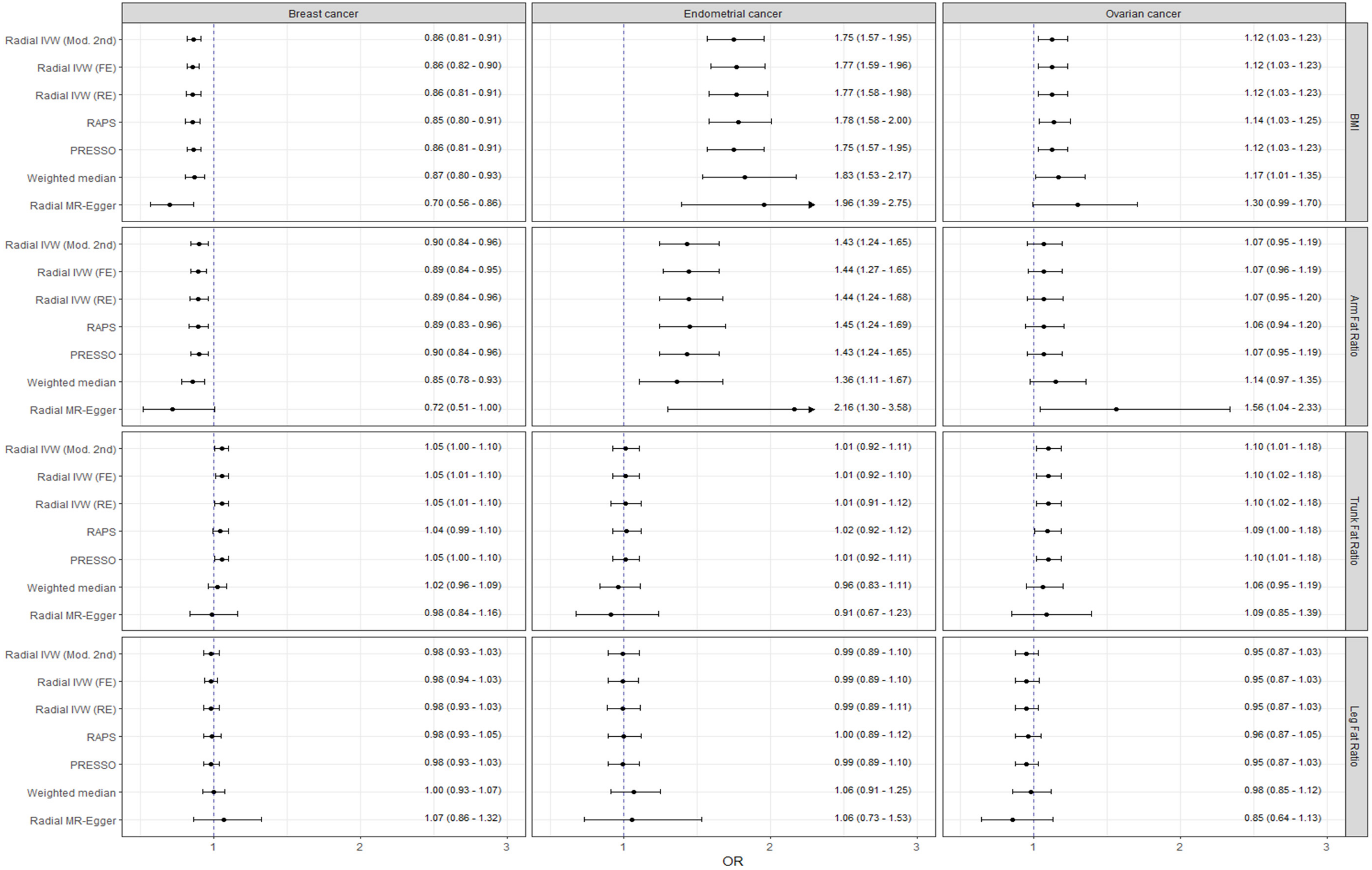

Figure S6. Mendelian randomization sensitivity analyses of the impact of obestiy measures on female specific cancers. Causal estimates represent odds ratios and 95 % confidence intervals per one standard deviation change in the respective exposure. Arrows indicate confidence intervals exceeding the plot range. Abbreviations: FE, fixed effects; IVW (Mod. 2nd), inverse-variance weighted model with modified second-order weights; RAPS, Robust Adjusted Profile Score; RE, random effects; PRESSO, Pleiotropy RESidual Sum and Outlier.

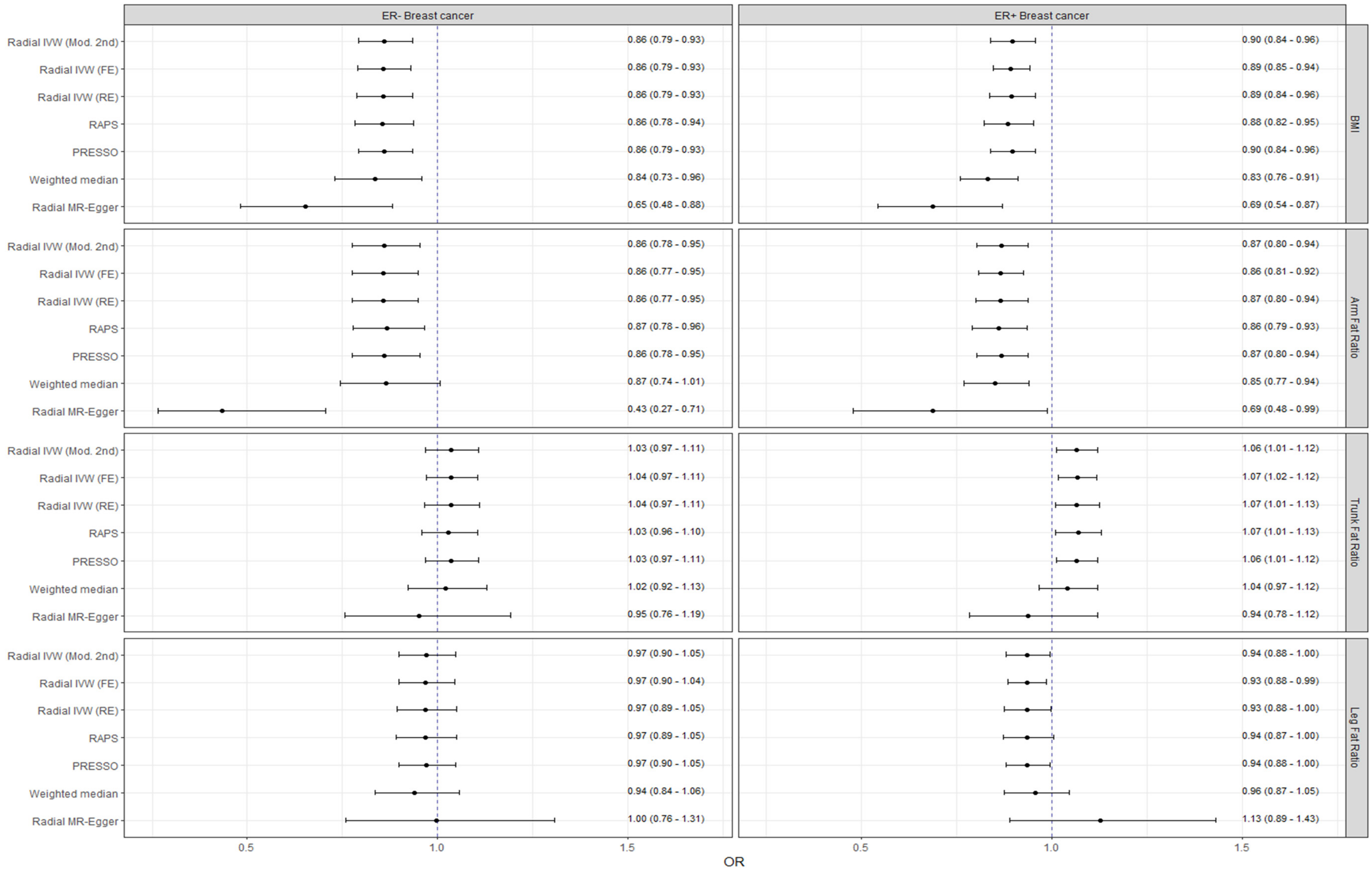

Figure S7. Mendelian randomization sensitivity analyses of the impact of obestiy measures on breast cancer subgroups. Causal estimates represent odds. ratios and 95 % confidence intervals per one standard deviation change in the respective exposure. Abbreviations: FE, fixed effects; IVW (Mod. 2nd), inverse-variance weighted model with modified second-order weights; RAPS, Robust Adjusted Profile Score; RE, random effects; PRESSO, Pleiotropy RESidual Sum and Outlier.

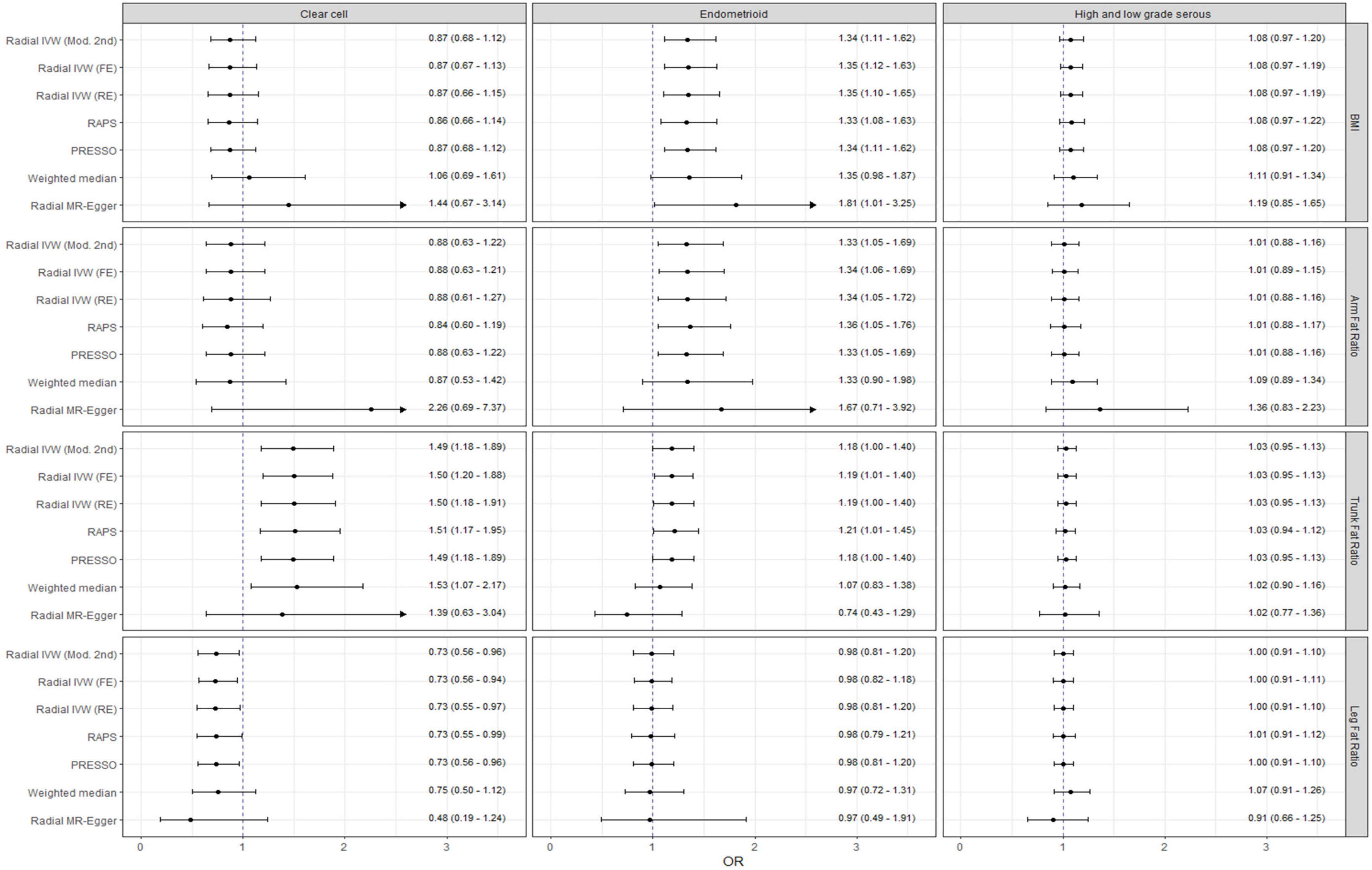

Figure S8. Mendelian randomization sensitivity analyses of the impact of obestiy measures on ovarian cancer subgroups. Causal estimates represent odds ratios and 95 % confidence intervals per one standard deviation change in the respective exposure. Arrows indicate confidence intervals exceeding the plot range. Abbreviations: FE, fixed effects; IVW (Mod. 2nd), inverse-variance weighted model with modified second-order weights; RAPS, Robust Adjusted Profile Score; RE, random effects; PRESSO, Pleiotropy RESidual Sum and Outlier.

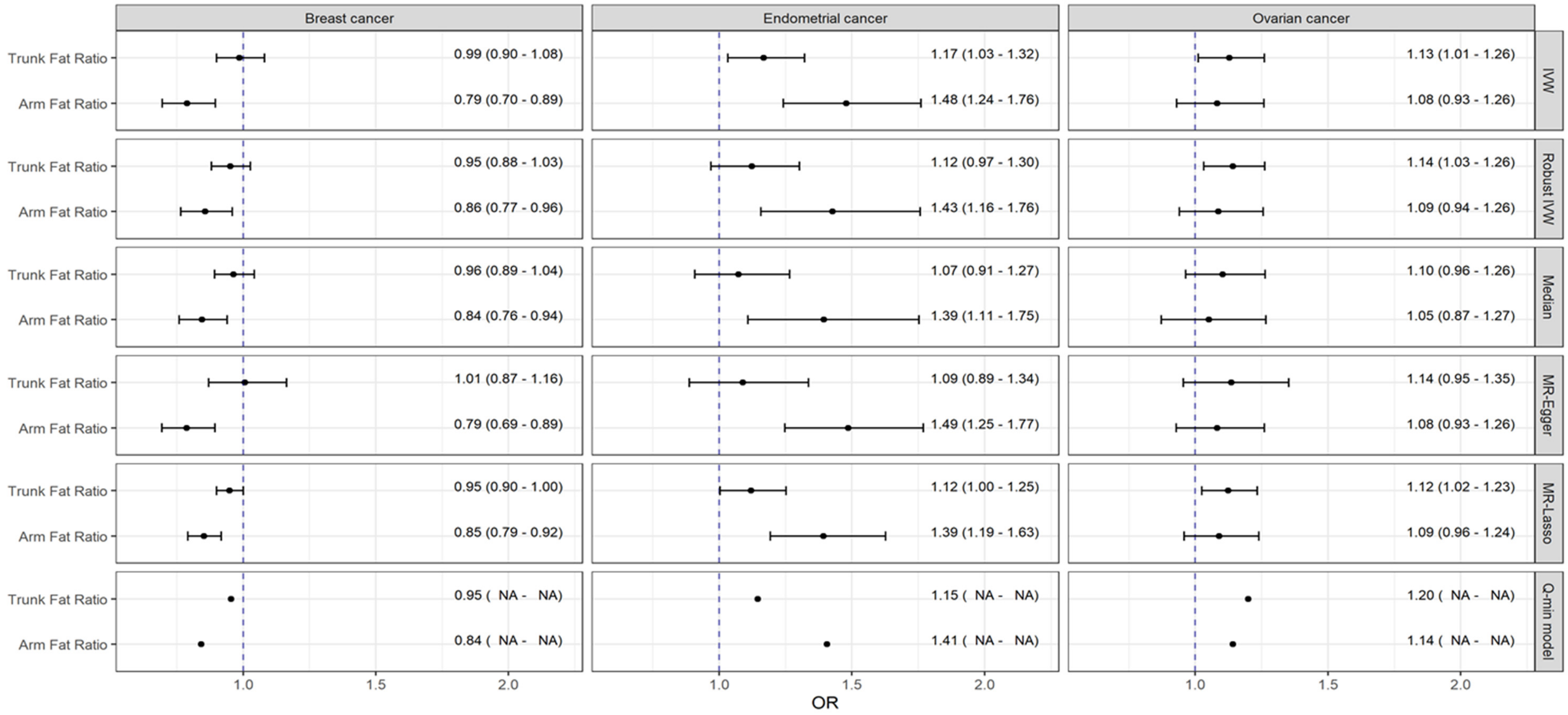

Figure S9. Multivariable Mendelian randomization sensitivity analyses of the impact of trunk and arm fat ratios on female-specific cancers. Causal estimates represent odds ratios and 95 % confidence intervals per one standard deviation change in the respective exposure. Abbreviations: IVW, inverse-variance weighted; OR, odds ratio.

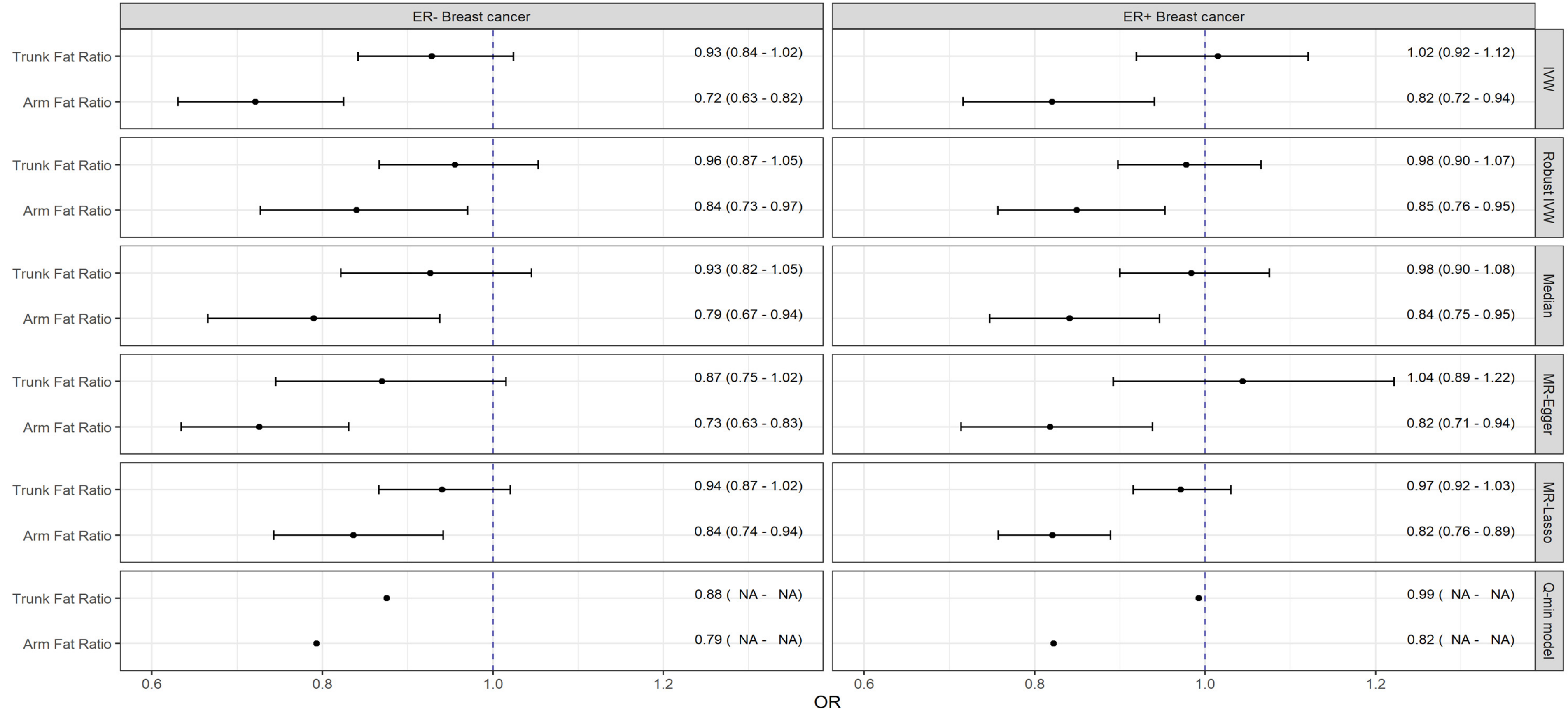

Figure S10. Multivariable Mendelian randomization sensitivity analyses of the impact of trunk and arm fat ratios on breast cancer subtypes. Causal estimates represent odds ratios and 95 % confidence intervals per one standard deviation change in the respective exposure. Abbreviations: IVW, inverse-variance weighted; OR, odds ratio.

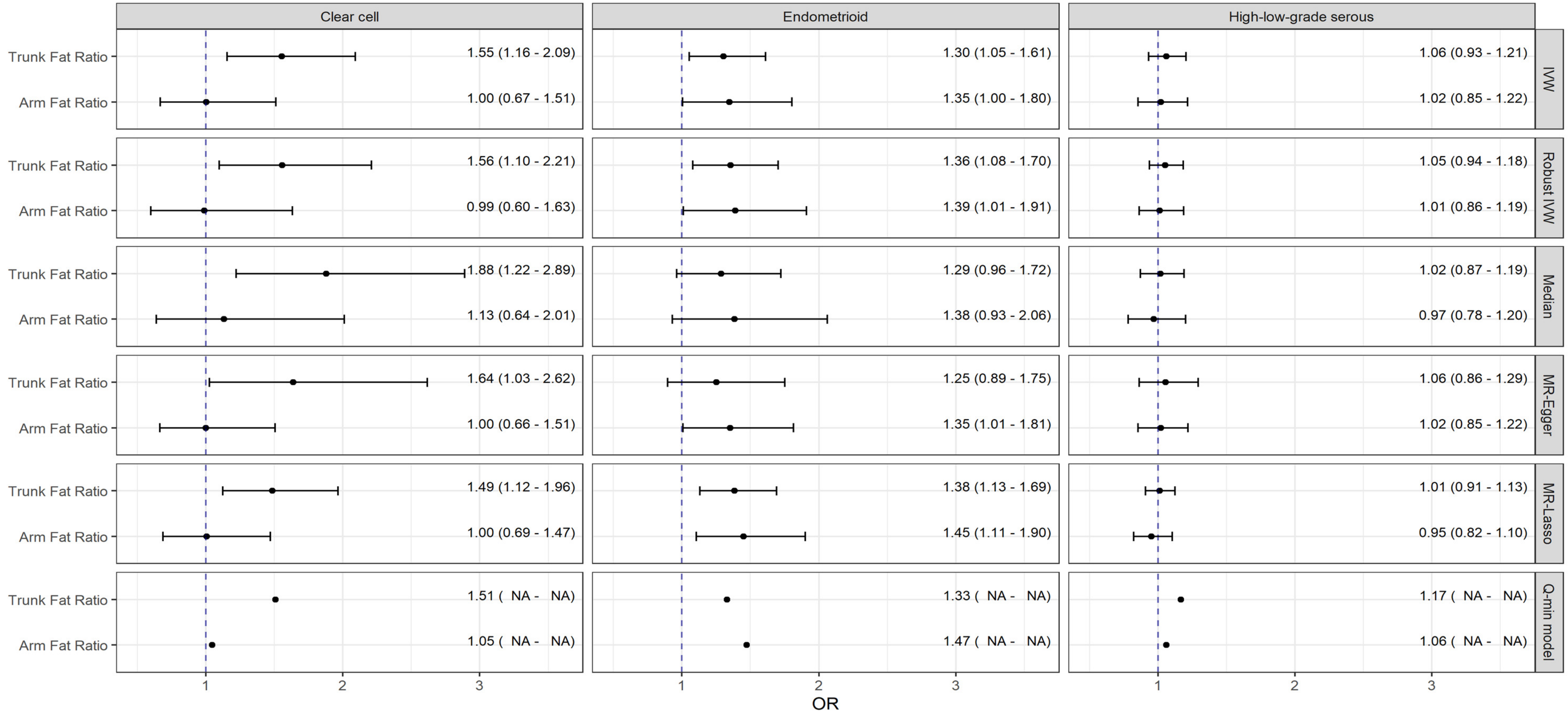

Figure S11. Multivariable Mendelian randomization sensitivity analyses of the impact of trunk and arm fat ratios on ovarian cancer subtypes. Causal estimates represent odds ratios and 95 % confidence intervals per one standard deviation change in the respective exposure. Abbreviations: IVW, inverse-variance weighted; OR, odds ratio.

Table S1. Results of the MR-PRESSO global and MR-Egger-intercept tests for detecting horizontal and directional pleiotropy and between SNP-heterogeneity based on the radial regression framework in the univariable Mendelian randomization setting.

Exposure	Outcome	PRESSO RSSobs	$P_{PRSSobs}$	Radial-Egger intercept	$P_{intercept}$	Cochrans Q	df	P_Q	Rueckers Q'	$P_{Q'}$	Q-Q'	$P_{Q-Q'}$	Q'/Q
**Main cancers**													
Body mass index	Breast cancer	351,603	2E-04	0,568	0,043	347,606	253,000	7E-05	337,250	3E-04	10,356	0,001	0,970
Body mass index	Endometrial cancer	335,140	0,074	-0,129	0,499	330,664	295,000	0,075	329,217	0,083	1,447	0,229	0,996
Body mass index	Ovarian cancer	296,797	0,424	-0,202	0,269	294,728	290,000	0,412	293,006	0,440	1,722	0,189	0,994
Trunk fat ratio	Breast cancer	219,376	0,004	0,263	0,413	216,581	165,000	0,004	215,822	0,005	0,759	0,384	0,996
Trunk fat ratio	Endometrial cancer	205,305	0,305	0,169	0,489	202,888	194,000	0,316	202,318	0,326	0,571	0,450	0,997
Trunk fat ratio	Ovarian cancer	199,868	0,290	0,018	0,942	197,729	188,000	0,299	197,741	0,299	-0,012	1,000	1,000
Arm fat ratio	Breast cancer	136,332	0,018	0,762	0,184	133,046	101,000	0,018	127,663	0,038	5,383	0,020	0,960
Arm fat ratio	Endometrial cancer	130,939	0,123	-0,600	0,102	127,615	110,000	0,120	122,277	0,200	5,338	0,021	0,958
Arm fat ratio	Ovarian cancer	120,525	0,283	-0,676	0,056	118,362	110,000	0,276	113,195	0,398	5,166	0,023	0,956
Leg fat ratio	Breast cancer	185,981	0,003	-0,328	0,416	183,181	132,000	0,002	182,076	0,003	1,105	0,293	0,994
Leg fat ratio	Endometrial cancer	161,236	0,368	-0,098	0,732	158,917	154,000	0,376	158,777	0,379	0,140	0,708	0,999
Leg fat ratio	Ovarian cancer	147,442	0,684	0,209	0,429	145,628	154,000	0,673	144,838	0,690	0,790	0,374	0,995
**Breast cancer subtypes**													
Body mass index	ER-	286,601	0,318	0,420	0,062	284,156	274,000	0,324	278,768	0,409	5,389	0,020	0,981
Body mass index	ER+	427,957	5E-09	0,615	0,023	424,255	272,000	9E-09	411,311	8E-07	12,944	3E-04	0,969
Trunk fat ratio	ER-	196,210	0,315	0,195	0,442	193,989	186,000	0,329	193,346	0,341	0,643	0,423	0,997
Trunk fat ratio	ER+	203,795	0,044	0,436	0,143	201,022	168,000	0,042	198,437	0,054	2,584	0,108	0,987
Arm fat ratio	ER-	112,360	0,375	1,277	0,006	110,132	106,000	0,372	97,427	0,712	12,705	4E-04	0,885
Arm fat ratio	ER+	134,251	0,025	0,678	0,200	131,129	101,000	0,024	126,362	0,045	4,768	0,029	0,964
Leg fat ratio	ER-	159,854	0,397	-0,059	0,838	157,685	154,000	0,403	157,653	0,403	0,031	0,859	1,000
Leg fat ratio	ER+	184,716	0,005	-0,603	0,111	181,736	135,000	0,005	177,979	0,008	3,757	0,053	0,979
**Ovarian cancer subtypes**													
Body mass index	Clear cell	264,126	0,889	-0,232	0,177	262,010	292,000	0,896	260,274	0,909	1,736	0,188	0,993
Body mass index	Endometrioid	296,290	0,453	-0,193	0,287	293,904	292,000	0,458	292,256	0,485	1,648	0,199	0,994
Body mass index	High and low grade serous	322,369	0,097	-0,118	0,536	320,318	289,000	0,099	319,722	0,103	0,596	0,440	0,998
Trunk fat ratio	Clear cell	208,210	0,192	0,048	0,849	205,843	189,000	0,191	205,871	0,190	-0,028	1,000	1,000
Trunk fat ratio	Endometrioid	210,907	0,137	0,435	0,082	208,570	187,000	0,134	205,044	0,174	3,526	0,060	0,983
Trunk fat ratio	High and low grade serous	192,387	0,399	0,021	0,930	190,394	186,000	0,397	190,391	0,397	0,003	0,958	1,000
Arm fat ratio	Clear cell	116,209	0,469	-0,550	0,107	113,772	114,000	0,488	110,737	0,569	3,036	0,081	0,973
Arm fat ratio	Endometrioid	114,572	0,474	-0,183	0,595	112,453	112,000	0,470	111,920	0,484	0,533	0,465	0,995
Arm fat ratio	High and low grade serous	131,653	0,098	-0,456	0,222	129,436	110,000	0,099	127,140	0,126	2,296	0,130	0,982
Leg fat ratio	Clear cell	175,410	0,164	0,265	0,362	173,069	156,000	0,166	171,664	0,185	1,405	0,236	0,992
Leg fat ratio	Endometrioid	187,641	0,063	0,017	0,953	185,333	157,000	0,061	185,327	0,061	0,006	0,937	1,000

Leg fat ratio	High and low grade serous	141,640	0,765	0,169	0,520	139,813	153,000	0,770	139,372	0,778	0,441	0,507	0,997

Table S2. Conditional F-statistics, Q-statistics, and Egger-intercept tests for multivariable Mendelian randomization models of genetically predicted trunk and arm fat ratios (TFR, AFR) on female specific cancers.

Outcome	F_{TFR}	F_{AFR}	Q_{IVW}	P_Q	Egger intercept	$P_{intercept}$
			**Main cancer types**			
Breast cancer	48,357	27,857	586,635	2E-28	-0,001	0,718
Endometrial cancer	50,273	25,826	282,960	0,063	0,002	0,402
Ovarian Cancer	48,357	27,857	364,844	5E-06	-2E-04	0,923
			**Breast cancer subtypes**			
ER-	48,357	27,857	297,776	0,028	0,002	0,286
ER+	48,357	27,857	466,177	8E-15	-0,001	0,651
			**Ovarian cancer subtypes**			
Clear cell	48,357	27,857	354,792	2E-05	-0,002	0,777
Endometrioid	48,357	27,857	319,165	0,003	0,001	0,766
High and low grade serous	48,357	27,857	333,399	0,001	1E-04	0,958

Table S3. Single Nucleotide Polymorphisms (SNPs) used as instruments in the Mendelian randomization analyses for the effect of BMI on breast cancer based on the genome-wide significance threshold P = 5E-08. R^2 represents the explained variance in the exposure. SNP-specific instrument strength is given by the respective F-statistic. Some of the genetic variants were identified as outliers in the iterative approach and therefore excluded from the analyses.

SNP	CHR	POS	EA	OA	R^2	F-stat	β_{BMI}	SE_{BMI}	P_{BMI}	β_{Breast}	SE_{Breast}	P_{breast}	$variant_{Breast}$
rs10002111	4	67815504	A	G	9,2E-05	36,430	0,0169	0,0028	9,3E-10	-0,0181	0,0074	0,0147	instrument
rs1003081	11	118913993	T	C	9,9E-05	42,843	0,0144	0,0022	6,3E-11	-0,0111	0,0064	0,0815	instrument
rs10048652	2	35449306	T	C	8,7E-05	34,452	0,0135	0,0023	3,5E-09	-0,0012	0,0063	0,8519	instrument
rs1009473	9	138777312	T	C	7,7E-05	30,489	-0,0127	0,0023	3,4E-08	0,0090	0,0062	0,1462	instrument
rs10099330	8	143383694	A	G	8,3E-05	32,440	-0,0131	0,0023	9,9E-09	-0,0015	0,0063	0,8071	instrument
rs10116186	9	80532374	A	G	1,2E-04	31,102	-0,0145	0,0026	4,1E-08	0,0150	0,0063	0,0170	instrument
rs10118701	9	103061366	A	G	1,4E-04	53,778	-0,0176	0,0024	2,2E-13	0,0009	0,0066	0,8888	instrument
rs10124645	9	11321119	A	G	8,9E-05	34,964	0,0136	0,0023	5,0E-09	-0,0071	0,0067	0,2838	instrument
rs10182181	2	25150296	A	G	6,2E-04	270,751	-0,0362	0,0022	5,0E-63	0,0403	0,0066	9,1E-10	outlier
rs10237317	7	70045941	A	G	9,1E-05	39,347	-0,0138	0,0022	6,8E-10	-0,0005	0,0064	0,9431	instrument
rs10271582	7	99121950	A	G	1,2E-04	32,222	0,0193	0,0034	8,4E-09	0,0064	0,0085	0,4534	instrument
rs10471636	5	63979856	A	G	1,3E-04	50,173	-0,0170	0,0024	4,7E-13	-0,0025	0,0067	0,7059	instrument
rs1048932	11	115044850	A	C	1,6E-04	68,438	-0,0182	0,0022	2,5E-16	0,0060	0,0063	0,3404	instrument

rs10499694	7	50614173	A	G	8,8E-05	38,215	0,0136	0,0022	4,0E-10	0,0029	0,0063	0,6523	instrument
rs10513801	3	185822353	T	G	2,8E-04	120,999	0,0352	0,0032	1,3E-27	-0,0074	0,0094	0,4344	instrument
rs10733682	9	129460914	A	G	1,2E-04	51,578	0,0158	0,0022	1,9E-12	-0,0233	0,0063	0,0002	outlier
rs10742752	11	45438374	T	C	8,3E-05	36,000	-0,0132	0,0022	4,0E-09	0,0069	0,0066	0,2966	instrument
rs10756714	9	15885041	A	G	2,1E-04	81,784	0,0208	0,0023	1,5E-19	0,0080	0,0062	0,1980	instrument
rs1075901	17	15943910	T	C	9,9E-05	42,843	-0,0144	0,0022	4,4E-11	0,0011	0,0062	0,8603	instrument
rs10761785	10	65318766	T	G	1,4E-04	61,124	-0,0172	0,0022	4,2E-15	-0,0239	0,0062	0,0001	outlier
rs10797115	9	92191256	T	C	1,2E-04	48,393	0,0160	0,0023	4,1E-12	-0,0056	0,0063	0,3712	instrument
rs10823893	10	53677313	A	G	1,6E-04	42,009	0,0175	0,0027	5,2E-11	-0,0167	0,0064	0,0094	instrument
rs10824347	10	77645229	A	T	1,1E-04	42,491	0,0176	0,0027	5,5E-11	0,0034	0,0074	0,6518	instrument
rs10909880	1	2727804	T	C	7,5E-05	31,768	-0,0124	0,0022	2,0E-08	0,0062	0,0063	0,3235	instrument
rs10920678	1	190239907	A	G	1,3E-04	54,894	0,0163	0,0022	7,5E-14	-0,0057	0,0065	0,3804	instrument
rs10938397	4	45182527	A	G	4,7E-04	205,009	-0,0315	0,0022	1,7E-45	0,0143	0,0062	0,0221	instrument
rs10962550	9	16720329	C	G	1,0E-04	40,960	0,0192	0,0030	1,7E-10	0,0157	0,0086	0,0696	instrument
rs10992867	9	96461013	A	G	1,3E-04	50,628	0,0185	0,0026	8,9E-13	-0,0007	0,0071	0,9270	instrument
rs11022762	11	13335926	T	C	1,0E-04	41,173	-0,0154	0,0024	5,6E-11	-0,0024	0,0063	0,7102	instrument
rs11073383	15	95274349	A	G	1,2E-04	47,790	-0,0159	0,0023	3,3E-12	0,0036	0,0062	0,5575	instrument
rs11165643	1	96924097	T	C	1,6E-04	67,688	0,0181	0,0022	1,7E-16	-0,0058	0,0065	0,3764	instrument
rs112646560	1	39560250	T	C	1,4E-04	37,515	0,0196	0,0032	1,1E-09	0,0102	0,0078	0,1879	instrument
rs11667280	19	30705747	C	G	7,5E-05	29,684	0,0158	0,0029	3,1E-08	0,0113	0,0085	0,1830	instrument
rs11672660	19	46180184	T	C	3,1E-04	131,429	-0,0321	0,0028	5,9E-31	0,0472	0,0080	4,1E-09	outlier
rs11677607	2	100751150	T	C	2,0E-04	79,012	-0,0240	0,0027	2,9E-19	0,0141	0,0073	0,0539	instrument
rs1167800	7	75176196	A	G	2,0E-04	82,644	0,0200	0,0022	3,7E-19	0,0145	0,0063	0,0208	outlier
rs11692326	2	208263279	T	C	8,3E-05	36,000	0,0156	0,0026	2,0E-09	0,0011	0,0075	0,8862	instrument
rs11727273	4	55509748	T	C	1,6E-04	42,250	-0,0169	0,0026	1,3E-10	0,0013	0,0066	0,8444	instrument
rs11751591	6	33794215	A	G	8,5E-05	33,423	-0,0185	0,0032	1,2E-08	0,0003	0,0089	0,9760	instrument
rs11754747	6	141494602	T	C	7,3E-05	31,532	0,0146	0,0026	1,3E-08	0,0026	0,0076	0,7335	instrument
rs11880870	19	18830704	A	G	1,8E-04	73,363	0,0197	0,0023	3,3E-18	0,0152	0,0066	0,0219	outlier
rs11945861	4	65700865	A	G	7,8E-05	30,864	-0,0150	0,0027	3,1E-08	-0,0103	0,0075	0,1671	instrument
rs11971098	7	26699061	A	G	8,5E-05	33,414	-0,0237	0,0041	6,5E-09	-0,0183	0,0111	0,0981	instrument
rs12042959	1	243533273	A	G	9,3E-05	36,754	0,0194	0,0032	2,3E-09	0,0196	0,0088	0,0261	outlier
rs12089815	1	91189933	A	G	1,3E-04	34,628	-0,0153	0,0026	6,0E-09	0,0088	0,0068	0,1925	instrument

rs12101625	15	26972431	C	G	8,4E-05	33,062	0,0138	0,0024	5,5E-09	-0,0198	0,0066	0,0025	outlier
rs12140153	1	62579891	T	G	1,8E-04	48,110	-0,0326	0,0047	2,9E-12	0,0121	0,0116	0,2998	instrument
rs12150665	17	34914787	T	C	1,3E-04	57,622	0,0167	0,0022	5,9E-14	0,0136	0,0071	0,0561	instrument
rs12202969	6	98576223	A	G	1,0E-04	39,745	-0,0145	0,0023	1,9E-10	-0,0023	0,0064	0,7167	instrument
rs12207125	6	154301575	A	G	1,3E-04	34,468	0,0182	0,0031	7,7E-09	-0,0100	0,0077	0,1939	instrument
rs12364470	11	134601012	T	G	1,0E-04	44,444	-0,0200	0,0030	2,5E-11	0,0088	0,0096	0,3618	instrument
rs12429545	13	54102206	A	G	1,8E-04	77,760	0,0291	0,0033	1,1E-18	0,0102	0,0098	0,3000	instrument
rs12448257	16	3599655	A	G	7,2E-05	30,864	0,0150	0,0027	3,1E-08	-0,0012	0,0079	0,8794	instrument
rs12462975	19	30272202	A	G	1,7E-04	65,934	0,0203	0,0025	2,6E-16	0,0213	0,0066	0,0013	outlier
rs12564992	1	174478100	A	G	8,0E-05	34,979	-0,0207	0,0035	2,4E-09	-0,0058	0,0098	0,5522	instrument
rs12593036	15	81058652	A	G	9,6E-05	37,945	0,0154	0,0025	6,6E-10	-0,0036	0,0069	0,6004	instrument
rs12612009	2	228982603	T	G	8,7E-05	34,515	-0,0141	0,0024	3,8E-09	-0,0002	0,0070	0,9730	instrument
rs12645001	4	96018337	A	G	8,7E-05	34,515	-0,0141	0,0024	4,1E-09	-0,0112	0,0066	0,0886	instrument
rs12680842	8	95582606	A	G	6,8E-05	29,340	0,0130	0,0024	3,4E-08	-0,0032	0,0067	0,6287	instrument
rs12681792	8	62054463	A	C	1,0E-04	39,820	0,0183	0,0029	2,6E-10	0,0048	0,0084	0,5697	instrument
rs12692596	2	161265910	T	C	9,3E-05	40,295	0,0146	0,0023	9,7E-11	0,0117	0,0065	0,0706	instrument
rs12692738	2	165558252	T	C	8,9E-05	38,822	-0,0162	0,0026	2,8E-10	0,0061	0,0073	0,4029	instrument
rs12714199	2	86812549	T	C	1,0E-04	40,295	-0,0146	0,0023	5,1E-10	0,0065	0,0065	0,3126	instrument
rs1285997	14	91513029	C	G	9,2E-05	36,000	-0,0150	0,0025	3,2E-09	-0,0001	0,0069	0,9843	instrument
rs12872889	13	28674628	T	C	1,7E-04	45,453	-0,0209	0,0031	1,5E-11	0,0054	0,0074	0,4689	instrument
rs1289736	3	107950892	T	C	9,3E-05	36,523	0,0139	0,0023	1,4E-09	-0,0097	0,0063	0,1253	instrument
rs12914489	15	74187937	A	G	9,1E-05	39,760	0,0227	0,0036	1,6E-10	0,0240	0,0103	0,0197	outlier
rs12949279	17	78558411	T	C	1,5E-04	66,200	0,0179	0,0022	6,1E-16	-0,0092	0,0065	0,1585	instrument
rs12981256	19	1865901	A	G	1,6E-04	62,616	0,0182	0,0023	3,9E-15	0,0051	0,0064	0,4244	instrument
rs13002158	2	144021712	A	G	1,3E-04	51,443	-0,0208	0,0029	1,2E-12	0,0164	0,0084	0,0508	instrument
rs13053342	22	48871624	A	G	1,0E-04	40,640	0,0153	0,0024	1,3E-10	-0,0003	0,0068	0,9614	instrument
rs13062093	3	35667057	T	G	8,3E-05	32,585	-0,0137	0,0024	8,0E-09	0,0012	0,0065	0,8498	instrument
rs13068138	3	85809425	T	C	1,7E-04	73,197	0,0231	0,0027	3,6E-17	-0,0119	0,0078	0,1274	instrument
rs13076052	3	44456573	C	G	1,2E-04	32,766	-0,0166	0,0029	1,3E-08	0,0130	0,0074	0,0777	instrument
rs13107325	4	103188709	T	C	2,4E-04	105,528	0,0452	0,0044	9,3E-25	0,0173	0,0125	0,1657	instrument
rs13110266	4	162129844	A	G	9,6E-05	41,661	-0,0142	0,0022	1,5E-10	-0,0044	0,0063	0,4902	instrument
rs13174863	5	139080745	A	G	1,8E-04	75,858	-0,0270	0,0031	4,5E-18	-0,0188	0,0098	0,0538	instrument

rs13209872	6	97753223	C	G	8,9E-05	35,007	-0,0142	0,0024	6,1E-09	0,0006	0,0067	0,9297	instrument
rs13213408	6	162998049	A	T	9,0E-05	35,637	0,0197	0,0033	4,3E-09	0,0116	0,0093	0,2105	instrument
rs1362910	8	30856464	A	G	8,2E-05	33,943	0,0134	0,0023	3,0E-09	0,0036	0,0064	0,5694	instrument
rs1403846	4	119101723	T	C	7,2E-05	31,206	0,0162	0,0029	1,6E-08	0,0065	0,0082	0,4261	instrument
rs1409818	20	21381121	T	C	1,2E-04	46,387	0,0252	0,0037	1,6E-11	-0,0202	0,0106	0,0563	instrument
rs141729694	5	87999371	T	C	2,2E-04	57,284	0,0386	0,0051	2,5E-14	-0,0132	0,0117	0,2593	instrument
rs1417665	6	104816944	T	C	1,2E-04	47,088	0,0199	0,0029	5,7E-12	-0,0233	0,0084	0,0055	outlier
rs1421598	2	50264710	A	C	9,7E-05	38,360	-0,0192	0,0031	6,8E-10	-0,0155	0,0085	0,0677	instrument
rs1436348	3	104612668	A	G	8,6E-05	33,943	-0,0134	0,0023	6,3E-09	0,0017	0,0064	0,7928	instrument
rs1441264	13	79580919	A	G	1,3E-04	55,276	0,0171	0,0023	5,2E-14	-0,0081	0,0069	0,2403	instrument
rs1452075	3	62481063	T	C	9,4E-05	40,449	0,0159	0,0025	1,5E-10	0,0157	0,0074	0,0347	instrument
rs1491333	4	172186288	T	G	8,1E-05	31,966	-0,0147	0,0026	9,9E-09	-0,0015	0,0076	0,8455	instrument
rs1492014	3	94071481	T	C	1,0E-04	39,745	-0,0145	0,0023	2,8E-10	0,0072	0,0063	0,2494	instrument
rs1518159	18	40760146	T	C	1,3E-04	52,841	-0,0189	0,0026	6,7E-13	-0,0050	0,0071	0,4826	instrument
rs1561589	10	126695673	A	G	1,1E-04	47,191	0,0158	0,0023	4,2E-12	-0,0051	0,0065	0,4294	instrument
rs1569979	14	29681294	A	G	1,8E-04	72,565	0,0230	0,0027	1,6E-17	-0,0016	0,0075	0,8364	instrument
rs1579557	6	40371918	T	C	1,8E-04	71,910	0,0212	0,0025	3,1E-17	-0,0014	0,0075	0,8529	instrument
rs158186	5	107440856	A	G	2,2E-04	85,711	-0,0287	0,0031	3,2E-20	-0,0039	0,0082	0,6360	instrument
rs159032	5	94206202	T	C	7,8E-05	30,454	0,0149	0,0027	4,5E-08	-0,0131	0,0072	0,0679	instrument
rs1681743	10	118565501	T	C	7,5E-05	29,793	-0,0131	0,0024	3,7E-08	-0,0112	0,0064	0,0818	instrument
rs16851483	3	141275436	T	G	1,6E-04	62,272	0,0363	0,0046	2,3E-15	0,0144	0,0129	0,2621	instrument
rs16912921	9	28413461	A	C	2,7E-04	117,204	0,0249	0,0023	1,6E-26	-0,0027	0,0067	0,6854	instrument
rs16975921	18	39914870	A	T	1,1E-04	42,510	-0,0163	0,0025	3,9E-11	0,0084	0,0066	0,2081	instrument
rs16989232	20	39291784	A	G	7,1E-05	30,972	0,0128	0,0023	1,5E-08	-0,0131	0,0067	0,0522	instrument
rs17014375	1	209543560	T	G	7,9E-05	31,228	-0,0190	0,0034	1,5E-08	0,0020	0,0092	0,8270	instrument
rs17066856	18	58049656	T	C	2,7E-04	115,707	0,0398	0,0037	1,4E-27	-0,0120	0,0107	0,2617	instrument
rs17405819	8	76806584	T	C	1,9E-04	82,507	0,0218	0,0024	7,8E-20	-0,0135	0,0067	0,0447	instrument
rs1750307	1	156488420	A	T	8,3E-05	32,585	0,0137	0,0024	8,2E-09	-0,0012	0,0064	0,8544	instrument
rs17522122	14	33302882	T	G	1,3E-04	57,622	0,0167	0,0022	6,5E-14	-0,0074	0,0065	0,2524	instrument
rs17619973	3	114417675	A	G	7,4E-05	31,673	0,0242	0,0043	1,3E-08	0,0183	0,0128	0,1524	instrument
rs17724992	19	18454825	A	G	1,1E-04	46,240	0,0170	0,0025	6,7E-12	0,0038	0,0071	0,5932	instrument
rs17751061	19	19413092	T	C	8,2E-05	31,993	-0,0181	0,0032	1,3E-08	0,0360	0,0086	3,0E-05	outlier

rs17767765	16	24539793	T	C	7,7E-05	30,489	-0,0127	0,0023	2,6E-08	-0,0029	0,0069	0,6699	instrument
rs17806224	20	51065854	A	G	2,3E-04	92,801	-0,0289	0,0030	3,5E-22	-0,0241	0,0086	0,0048	outlier
rs1799923	3	42306294	A	G	1,2E-04	48,203	-0,0243	0,0035	5,1E-12	0,0201	0,0107	0,0602	instrument
rs1801282	3	12393125	C	G	7,0E-05	30,417	-0,0182	0,0033	4,7E-08	0,0165	0,0094	0,0794	instrument
rs1808579	18	21104888	T	C	1,9E-04	81,000	-0,0198	0,0022	9,4E-20	-0,0096	0,0063	0,1268	instrument
rs1808629	8	73435964	A	G	2,5E-04	65,147	-0,0226	0,0028	5,8E-16	-0,0089	0,0070	0,2036	instrument
rs1812587	5	62991802	T	G	1,3E-04	34,177	0,0152	0,0026	7,0E-09	0,0084	0,0063	0,1830	instrument
rs185350	19	34306816	T	C	7,8E-05	33,851	0,0128	0,0022	7,1E-09	-0,0143	0,0062	0,0220	instrument
rs1861412	2	58893065	A	G	2,2E-04	87,381	-0,0215	0,0023	9,1E-21	0,0031	0,0062	0,6172	instrument
rs1884389	20	1410582	T	C	9,4E-05	37,051	-0,0140	0,0023	1,2E-09	0,0071	0,0062	0,2546	instrument
rs1884897	20	6612832	A	G	1,2E-04	53,990	-0,0169	0,0023	9,1E-14	0,0060	0,0066	0,3641	instrument
rs1916801	3	61187046	A	T	1,4E-04	59,710	0,0170	0,0022	1,8E-14	0,0026	0,0065	0,6892	instrument
rs1927790	13	96922191	T	C	8,7E-05	37,655	-0,0135	0,0022	1,3E-09	0,0038	0,0065	0,5575	instrument
rs1928295	9	120378483	T	C	1,2E-04	52,892	0,0160	0,0022	2,8E-13	0,0023	0,0063	0,7177	instrument
rs1949197	3	86273850	A	G	9,2E-05	36,523	0,0139	0,0023	3,0E-09	-0,0099	0,0064	0,1204	instrument
rs197374	1	112289983	T	C	1,4E-04	55,276	0,0171	0,0023	2,8E-13	-0,0024	0,0065	0,7141	instrument
rs1984559	2	135678888	A	G	7,9E-05	29,521	0,0163	0,0030	3,3E-08	0,0189	0,0075	0,0120	outlier
rs198665	6	143154886	A	C	7,8E-05	33,943	0,0134	0,0023	4,4E-09	-0,0029	0,0065	0,6607	instrument
rs2012502	16	81728081	A	C	1,0E-04	44,251	0,0153	0,0023	3,0E-11	-0,0075	0,0067	0,2665	instrument
rs2045293	7	114350102	T	C	1,7E-04	67,525	0,0189	0,0023	4,1E-16	-0,0170	0,0063	0,0072	instrument
rs2053682	5	170599327	A	C	1,4E-04	53,582	0,0183	0,0025	1,9E-13	-0,0015	0,0068	0,8237	instrument
rs209416	6	83534729	A	T	1,2E-04	47,840	0,0166	0,0024	8,7E-12	-0,0140	0,0067	0,0364	instrument
rs2112347	5	75015242	T	G	4,2E-04	180,493	0,0309	0,0023	2,1E-42	-0,0183	0,0064	0,0044	instrument
rs214249	16	348687	T	G	1,2E-04	48,418	0,0167	0,0024	1,4E-12	0,0019	0,0063	0,7685	instrument
rs2161097	5	103945178	T	C	8,0E-05	31,457	0,0129	0,0023	2,3E-08	0,0023	0,0063	0,7144	instrument
rs2178899	6	31606756	A	T	1,3E-04	56,477	0,0248	0,0033	7,2E-14	-0,0052	0,0096	0,5930	instrument
rs2185027	6	153381622	A	C	9,5E-05	41,173	-0,0154	0,0024	1,1E-10	0,0057	0,0068	0,4002	instrument
rs2228213	6	12124855	A	G	9,5E-05	41,406	-0,0148	0,0023	9,2E-11	-0,0029	0,0065	0,6587	instrument
rs2267812	7	74138121	A	C	9,2E-05	35,177	-0,0172	0,0029	1,7E-09	-0,0322	0,0076	2,6E-05	outlier
rs2267958	9	131015279	A	G	1,2E-04	45,002	-0,0161	0,0024	1,9E-11	-0,0006	0,0067	0,9282	instrument
rs2271046	4	52752812	A	T	7,6E-05	30,030	-0,0137	0,0025	2,4E-08	-0,0216	0,0070	0,0019	outlier
rs2289292	16	30097630	T	C	1,5E-04	57,760	-0,0190	0,0025	1,1E-14	0,0009	0,0072	0,9012	instrument

rs2289705	7	121983843	A	G	7,7E-05	33,284	-0,0150	0,0026	1,5E-08	-0,0129	0,0079	0,1055	instrument
rs2299383	7	103418846	T	C	1,6E-04	68,438	0,0182	0,0022	2,7E-16	-0,0137	0,0064	0,0319	instrument
rs2357760	6	120213880	A	G	1,1E-04	47,790	0,0159	0,0023	1,1E-11	-0,0086	0,0067	0,2003	instrument
rs2465043	6	51180765	A	G	8,9E-05	35,007	-0,0142	0,0024	3,2E-09	0,0064	0,0065	0,3263	instrument
rs2470520	3	15762920	T	C	7,6E-05	30,011	-0,0126	0,0023	4,2E-08	-0,0052	0,0063	0,4098	instrument
rs247975	3	173107443	T	C	1,6E-04	62,616	-0,0182	0,0023	3,6E-15	-0,0002	0,0071	0,9735	instrument
rs2479958	13	111984244	A	G	8,1E-05	31,640	0,0135	0,0024	1,4E-08	-0,0040	0,0065	0,5350	instrument
rs2528531	7	93236510	A	C	9,3E-05	36,502	-0,0145	0,0024	1,3E-09	0,0082	0,0065	0,2070	instrument
rs2568958	1	72765116	A	G	2,5E-04	106,464	0,0227	0,0022	1,3E-24	-0,0188	0,0064	0,0031	instrument
rs2605603	11	93221105	A	G	8,3E-05	36,000	-0,0132	0,0022	1,4E-09	0,0001	0,0063	0,9824	instrument
rs2619976	17	71754545	T	C	9,4E-05	37,007	0,0146	0,0024	6,2E-10	0,0017	0,0069	0,8023	instrument
rs2721965	8	116662038	A	C	1,5E-04	60,062	0,0186	0,0024	2,1E-14	0,0182	0,0066	0,0057	outlier
rs273504	19	18215247	A	G	1,1E-04	43,102	-0,0151	0,0023	1,3E-10	-0,0149	0,0063	0,0186	outlier
rs2754079	14	25886140	A	G	1,2E-04	46,004	-0,0156	0,0023	2,3E-11	0,0134	0,0066	0,0436	instrument
rs2814992	6	34617144	A	G	3,6E-04	155,706	-0,0287	0,0023	1,2E-35	0,0013	0,0066	0,8424	instrument
rs2820295	1	201800868	A	G	2,0E-04	79,507	0,0214	0,0024	8,0E-19	-0,0070	0,0066	0,2910	instrument
rs2836754	21	40291740	T	C	1,1E-04	45,870	-0,0149	0,0022	3,7E-11	0,0047	0,0064	0,4670	instrument
rs28489620	22	41804716	A	G	1,5E-04	38,955	-0,0181	0,0029	6,9E-10	0,0216	0,0069	0,0018	outlier
rs2861685	2	67837553	T	C	1,8E-04	47,929	0,0180	0,0026	1,1E-11	0,0194	0,0063	0,0021	outlier
rs2862961	11	43656535	A	G	2,3E-04	91,393	-0,0239	0,0025	5,3E-22	-0,0076	0,0067	0,2603	instrument
rs2959592	8	17002725	T	C	8,7E-05	36,862	0,0170	0,0028	6,3E-10	0,0000	0,0082	0,9985	instrument
rs2968864	7	150622162	T	C	9,2E-05	36,446	0,0163	0,0027	9,3E-10	-0,0174	0,0073	0,0168	instrument
rs2984618	1	47690438	T	G	1,3E-04	57,622	0,0167	0,0022	2,2E-14	-0,0035	0,0063	0,5840	instrument
rs329651	11	133767622	T	G	9,7E-05	41,787	0,0181	0,0028	2,0E-10	-0,0177	0,0081	0,0297	instrument
rs337637	4	38604470	A	G	6,8E-05	29,537	-0,0125	0,0023	3,0E-08	-0,0027	0,0065	0,6824	instrument
rs34517439	1	78450517	A	C	3,4E-04	90,481	0,0390	0,0041	2,0E-21	0,0104	0,0105	0,3211	instrument
rs34811474	4	25408838	A	G	4,0E-04	104,566	-0,0317	0,0031	5,2E-24	-0,0045	0,0089	0,6092	instrument
rs34930419	10	33987329	T	C	1,4E-04	36,316	-0,0458	0,0076	1,4E-09	-0,0067	0,0186	0,7179	instrument
rs35107973	4	143125948	T	C	1,2E-04	32,573	0,0234	0,0041	7,7E-09	0,0155	0,0104	0,1364	instrument
rs355754	3	154024165	T	C	9,2E-05	38,117	-0,0142	0,0023	4,3E-10	0,0014	0,0065	0,8345	instrument
rs35722922	2	47002226	A	G	1,8E-04	48,482	0,0188	0,0027	3,6E-12	0,0038	0,0064	0,5596	instrument
rs3803286	14	103246470	A	G	1,3E-04	51,361	0,0172	0,0024	8,3E-13	-0,0206	0,0065	0,0016	outlier

rs3806114	6	20482335	A	G	8,0E-05	34,515	-0,0141	0,0024	4,5E-09	-0,0204	0,0067	0,0023	outlier
rs3826408	17	7101292	T	C	7,0E-05	30,250	0,0121	0,0022	4,5E-08	0,0134	0,0063	0,0328	instrument
rs3829849	9	129390800	T	C	8,0E-05	34,964	0,0136	0,0023	1,9E-09	0,0244	0,0065	0,0002	outlier
rs3977755	10	104420210	T	C	8,0E-05	32,718	-0,0143	0,0025	8,5E-09	0,0147	0,0070	0,0348	instrument
rs4055791	13	59266053	T	C	1,5E-04	39,643	-0,0170	0,0027	1,8E-10	-0,0130	0,0065	0,0463	instrument
rs4148155	4	89054667	A	G	7,7E-05	33,640	0,0203	0,0035	6,2E-09	0,0414	0,0101	4,3E-05	outlier
rs4240673	8	10787612	T	C	1,5E-04	64,000	0,0176	0,0022	1,0E-15	-0,0119	0,0073	0,1024	instrument
rs427943	21	46570896	A	C	1,5E-04	59,223	-0,0177	0,0023	1,4E-14	0,0016	0,0063	0,7946	instrument
rs429358	19	45411941	T	C	2,2E-04	58,777	0,0276	0,0036	3,2E-14	0,0232	0,0092	0,0115	outlier
rs4295917	1	32173360	A	G	8,2E-05	31,640	0,0135	0,0024	1,6E-08	-0,0121	0,0071	0,0869	instrument
rs4307239	7	24354300	A	G	9,5E-05	37,582	-0,0141	0,0023	1,3E-09	0,0116	0,0065	0,0742	instrument
rs4320543	8	34422967	A	G	8,0E-05	31,457	-0,0129	0,0023	2,9E-08	0,0040	0,0067	0,5552	instrument
rs4421623	1	98316285	T	G	1,2E-04	46,441	0,0184	0,0027	1,1E-11	0,0040	0,0073	0,5849	instrument
rs4459316	11	130756176	T	C	1,1E-04	44,832	-0,0154	0,0023	2,3E-11	0,0073	0,0064	0,2573	instrument
rs4474229	1	195037001	A	G	1,6E-04	41,055	-0,0173	0,0027	1,6E-10	0,0043	0,0064	0,4970	instrument
rs4482463	2	205375909	A	C	2,0E-04	75,524	-0,0365	0,0042	5,2E-18	0,0148	0,0119	0,2161	instrument
rs4569924	5	153540025	T	C	1,2E-04	51,578	0,0158	0,0022	7,4E-13	-0,0103	0,0063	0,0998	instrument
rs4586287	13	99245334	C	G	7,7E-05	30,470	0,0138	0,0025	2,9E-08	-0,0161	0,0067	0,0163	instrument
rs4673553	2	211608379	T	G	1,1E-04	44,251	-0,0153	0,0023	2,6E-11	0,0041	0,0063	0,5188	instrument
rs4677812	3	194863794	A	C	8,4E-05	33,177	-0,0144	0,0025	9,4E-09	-0,0020	0,0071	0,7819	instrument
rs4776982	15	68114974	A	G	3,3E-04	143,078	0,0311	0,0026	4,2E-33	-0,0087	0,0074	0,2446	instrument
rs4783718	16	69547741	T	C	1,8E-04	78,669	0,0204	0,0023	1,8E-19	0,0145	0,0066	0,0284	outlier
rs4790292	17	1824305	A	C	1,7E-04	63,516	-0,0263	0,0033	1,7E-15	0,0155	0,0092	0,0914	instrument
rs4834272	4	113313986	T	C	8,9E-05	38,656	-0,0143	0,0023	8,2E-10	-0,0024	0,0070	0,7356	instrument
rs4858223	3	20581149	T	C	1,1E-04	41,473	-0,0161	0,0025	8,3E-11	0,0067	0,0068	0,3256	instrument
rs4864201	4	130731284	T	C	7,5E-05	32,440	0,0131	0,0023	6,3E-09	0,0016	0,0065	0,8103	instrument
rs4880341	10	133992689	T	C	9,8E-05	38,656	-0,0143	0,0023	4,5E-10	0,0006	0,0063	0,9230	instrument
rs4889606	16	31011183	A	G	3,1E-04	135,773	0,0268	0,0023	1,3E-32	-0,0140	0,0065	0,0319	instrument
rs4929923	11	8639200	T	C	2,1E-04	92,327	-0,0221	0,0023	2,5E-22	-0,0004	0,0064	0,9524	instrument
rs4936175	11	132641959	T	C	1,2E-04	47,191	-0,0158	0,0023	6,7E-12	0,0040	0,0063	0,5250	instrument
rs4969387	17	79081724	C	G	8,4E-05	32,532	-0,0154	0,0027	6,9E-09	-0,0138	0,0072	0,0560	instrument
rs4986044	17	21261560	T	C	1,4E-04	61,836	-0,0173	0,0022	2,2E-15	-0,0021	0,0064	0,7465	instrument

rs543874	1	177889480	A	G	1,0E-03	455,109	-0,0576	0,0027	8,7E-100	0,0302	0,0079	0,0001	outlier
rs56161855	17	46288649	A	T	1,7E-04	44,787	-0,0261	0,0039	1,5E-11	0,0032	0,0088	0,7156	instrument
rs563132	9	37245476	A	T	1,4E-04	56,250	0,0180	0,0024	2,0E-14	-0,0159	0,0064	0,0125	instrument
rs563296	10	99772404	A	G	1,4E-04	53,168	0,0175	0,0024	8,8E-14	0,0041	0,0066	0,5371	instrument
rs57221746	15	99230035	A	G	1,2E-04	30,594	-0,0177	0,0032	2,1E-08	0,0031	0,0075	0,6807	instrument
rs57800857	4	140863365	A	C	1,8E-04	47,456	0,0186	0,0027	1,1E-11	0,0138	0,0067	0,0381	instrument
rs6010784	20	61540319	T	C	7,6E-05	32,802	0,0126	0,0022	1,1E-08	0,0019	0,0062	0,7549	instrument
rs6050446	20	25195509	A	G	8,0E-05	33,785	-0,0372	0,0064	6,2E-09	0,0120	0,0191	0,5294	instrument
rs6142096	20	32686658	A	G	8,6E-05	33,943	0,0134	0,0023	4,2E-09	-0,0136	0,0063	0,0299	instrument
rs61813324	1	156049877	T	C	2,2E-04	58,385	0,0298	0,0039	3,6E-14	-0,0194	0,0102	0,0582	instrument
rs62007782	15	78029797	A	G	1,4E-04	35,601	-0,0179	0,0030	1,9E-09	-0,0019	0,0072	0,7963	instrument
rs62106258	2	417167	T	C	9,7E-04	254,452	0,0989	0,0062	8,7E-58	-0,0142	0,0181	0,4318	instrument
rs62412826	6	61881167	T	C	1,3E-04	33,518	0,0330	0,0057	8,0E-09	0,0215	0,0140	0,1252	instrument
rs625652	11	65641931	A	T	2,0E-04	52,160	-0,0195	0,0027	9,1E-13	-0,0305	0,0065	2,6E-06	outlier
rs6265	11	27679916	T	C	4,0E-04	172,734	-0,0368	0,0028	8,0E-40	-0,0093	0,0078	0,2364	outlier
rs6446187	3	49907111	A	C	4,3E-04	114,325	0,0278	0,0026	1,2E-26	0,0172	0,0062	0,0055	outlier
rs6493498	15	51754451	T	C	1,3E-04	54,894	0,0163	0,0022	1,5E-13	-0,0108	0,0063	0,0868	instrument
rs6548237	2	621461	A	C	9,1E-04	394,500	-0,0576	0,0029	4,4E-89	0,0214	0,0105	0,0416	instrument
rs6556810	5	164585949	T	C	7,4E-05	29,066	-0,0124	0,0023	5,0E-08	-0,0070	0,0064	0,2783	instrument
rs6561937	13	58257667	A	T	1,4E-04	53,236	-0,0197	0,0027	6,0E-13	-0,0206	0,0075	0,0061	outlier
rs6567160	18	57829135	T	C	1,1E-03	475,573	-0,0567	0,0026	8,7E-108	0,0226	0,0073	0,0020	instrument
rs657452	1	49589847	A	G	2,0E-04	85,142	0,0203	0,0022	1,1E-19	-0,0058	0,0064	0,3642	instrument
rs6585198	10	114762237	A	G	1,1E-04	49,000	-0,0154	0,0022	1,2E-12	0,0421	0,0062	9,9E-12	outlier
rs6669189	1	75011358	T	C	2,1E-04	91,115	0,0210	0,0022	4,7E-21	-0,0075	0,0064	0,2373	instrument
rs6720868	2	230663576	T	C	1,3E-04	49,585	0,0169	0,0024	4,6E-12	-0,0110	0,0066	0,0958	instrument
rs6728037	2	59297346	A	G	2,1E-04	81,000	0,0207	0,0023	6,5E-19	0,0037	0,0064	0,5645	instrument
rs6739199	2	105452326	T	C	1,0E-04	40,449	0,0159	0,0025	3,2E-10	-0,0003	0,0075	0,9687	instrument
rs6870983	5	87697533	T	C	1,5E-04	66,485	-0,0212	0,0026	9,6E-16	0,0120	0,0074	0,1082	instrument
rs6890310	5	27193573	A	G	9,6E-05	37,945	-0,0154	0,0025	1,4E-09	0,0081	0,0073	0,2682	instrument
rs6973700	7	1856669	A	G	1,4E-04	36,376	0,0193	0,0032	1,8E-09	0,0043	0,0077	0,5740	instrument
rs7024334	9	109072075	T	G	8,7E-05	37,398	0,0159	0,0026	1,6E-09	0,0167	0,0075	0,0252	instrument
rs7084454	10	21821274	A	G	2,1E-04	81,000	0,0225	0,0025	7,4E-20	0,0404	0,0066	9,1E-10	outlier

rs709400	14	104149475	A	G	1,5E-04	64,000	0,0184	0,0023	4,4E-16	-0,0211	0,0064	0,0009	outlier
rs7105462	11	112912048	A	G	9,9E-05	39,198	0,0144	0,0023	4,4E-10	0,0034	0,0063	0,5882	instrument
rs7107409	11	76492195	T	C	1,2E-04	30,941	-0,0178	0,0032	2,1E-08	0,0107	0,0075	0,1532	instrument
rs7124681	11	47529947	A	C	2,9E-04	126,051	0,0247	0,0022	9,3E-29	-0,0043	0,0063	0,5005	instrument
rs7141420	14	79899454	T	C	2,1E-04	91,115	0,0210	0,0022	7,8E-22	0,0172	0,0066	0,0092	outlier
rs7211966	17	54374611	T	C	8,9E-05	34,515	-0,0470	0,0080	4,9E-09	0,0132	0,0273	0,6286	instrument
rs7239114	18	45921214	A	G	1,2E-04	49,638	0,0155	0,0022	3,9E-12	-0,0003	0,0067	0,9626	instrument
rs7250833	19	33937277	T	C	8,9E-05	38,543	0,0149	0,0024	7,2E-10	-0,0102	0,0070	0,1426	instrument
rs73124396	7	71579606	T	C	1,2E-04	32,111	0,0187	0,0033	1,1E-08	0,0066	0,0076	0,3873	instrument
rs73213484	4	28489339	A	T	1,7E-04	45,653	0,0250	0,0037	2,3E-11	0,0005	0,0088	0,9522	instrument
rs7335249	13	109962579	T	C	8,7E-05	34,452	0,0135	0,0023	4,9E-09	-0,0002	0,0065	0,9800	instrument
rs7395632	11	958789	T	C	7,4E-05	29,340	-0,0130	0,0024	3,9E-08	0,0009	0,0066	0,8880	instrument
rs7428670	3	131579810	C	G	1,5E-04	60,361	0,0202	0,0026	3,1E-15	-0,0027	0,0071	0,7058	instrument
rs7498044	15	92573639	A	G	1,2E-04	31,640	-0,0180	0,0032	2,7E-08	0,0033	0,0079	0,6733	instrument
rs7498665	16	28883241	A	G	3,3E-04	143,999	-0,0264	0,0022	1,9E-32	-0,0043	0,0064	0,5024	instrument
rs750090	4	152931436	T	C	1,1E-04	42,250	0,0156	0,0024	7,4E-11	-0,0057	0,0067	0,3928	instrument
rs7550711	1	110082886	T	C	2,1E-04	88,858	0,0641	0,0068	3,4E-21	-0,0580	0,0179	0,0012	outlier
rs7571496	2	6169351	A	G	7,9E-05	31,102	0,0145	0,0026	1,4E-08	-0,0079	0,0073	0,2800	instrument
rs7588437	2	181575281	A	G	1,3E-04	58,555	-0,0176	0,0023	8,4E-15	0,0130	0,0065	0,0465	instrument
rs7594237	2	198187164	A	T	8,3E-05	32,718	-0,0143	0,0025	1,8E-08	-0,0189	0,0072	0,0084	outlier
rs7601870	2	15384392	A	G	8,3E-05	32,937	-0,0132	0,0023	1,2E-08	0,0040	0,0063	0,5241	instrument
rs7601895	2	55281901	C	G	1,1E-04	41,473	0,0161	0,0025	9,6E-11	0,0075	0,0070	0,2856	instrument
rs7621025	3	136272246	T	C	1,5E-04	64,000	-0,0200	0,0025	1,6E-15	0,0143	0,0071	0,0453	instrument
rs7647242	3	82642734	T	C	1,0E-04	39,745	0,0145	0,0023	3,9E-10	0,0055	0,0066	0,4051	instrument
rs768023	6	108876002	A	G	1,6E-04	70,712	0,0185	0,0022	1,4E-16	0,0064	0,0064	0,3206	instrument
rs76900466	2	145057760	T	G	1,2E-04	30,654	0,0227	0,0041	4,5E-08	-0,0210	0,0132	0,1113	instrument
rs77432547	13	86494817	A	G	1,4E-04	36,415	-0,0175	0,0029	2,3E-09	0,0114	0,0070	0,1042	instrument
rs7788008	7	112972483	A	G	1,4E-04	55,276	-0,0171	0,0023	1,1E-13	0,0016	0,0063	0,8033	instrument
rs7805441	7	78121458	T	C	1,0E-04	40,295	0,0146	0,0023	2,0E-10	0,0008	0,0062	0,8980	instrument
rs782971	5	43124688	A	G	1,1E-04	47,334	-0,0172	0,0025	4,9E-12	-0,0189	0,0075	0,0119	outlier
rs786420	2	44719893	T	C	8,4E-05	32,956	0,0155	0,0027	8,8E-09	-0,0166	0,0073	0,0228	instrument
rs78886584	1	16859325	A	G	1,3E-04	33,284	-0,0150	0,0026	1,3E-08	-0,0035	0,0070	0,6140	instrument

rs7899106	10	87410904	A	G	1,3E-04	56,250	-0,0375	0,0050	7,6E-14	0,0158	0,0148	0,2837	instrument
rs79113395	1	1590521	A	G	1,9E-04	49,000	-0,0210	0,0030	1,5E-12	-0,0073	0,0102	0,4780	instrument
rs8024806	15	53473990	T	C	8,5E-05	33,492	0,0272	0,0047	9,8E-09	-0,0053	0,0131	0,6838	instrument
rs8033510	15	61445514	T	C	8,3E-05	32,585	0,0137	0,0024	1,1E-08	0,0048	0,0064	0,4585	instrument
rs8038522	15	46585105	T	G	9,4E-05	37,051	-0,0140	0,0023	1,3E-09	-0,0065	0,0063	0,3025	instrument
rs8075273	17	61728881	A	C	1,0E-04	44,444	-0,0160	0,0024	5,6E-11	0,0101	0,0069	0,1466	instrument
rs811054	16	72251132	T	C	1,2E-04	54,223	0,0162	0,0022	3,2E-13	0,0193	0,0063	0,0022	outlier
rs836525	7	6465909	T	C	8,2E-05	32,233	0,0176	0,0031	9,8E-09	-0,0093	0,0086	0,2769	instrument
rs879620	16	4015729	T	C	2,9E-04	115,328	0,0247	0,0023	5,4E-26	0,0024	0,0066	0,7122	instrument
rs893006	11	64365796	A	C	1,2E-04	51,960	0,0173	0,0024	4,0E-13	-0,0006	0,0069	0,9340	instrument
rs895330	19	4060707	C	G	9,9E-05	38,955	0,0181	0,0029	6,6E-10	0,0164	0,0084	0,0504	instrument
rs915814	21	46493003	A	G	1,3E-04	34,810	-0,0177	0,0030	5,9E-09	0,0009	0,0073	0,8988	instrument
rs925018	3	62713143	C	G	8,8E-05	38,117	-0,0142	0,0023	9,8E-10	0,0149	0,0067	0,0253	instrument
rs9304665	19	47602577	A	T	1,4E-04	57,086	0,0204	0,0027	1,8E-14	-0,0218	0,0073	0,0030	outlier
rs9367369	6	13189941	A	G	1,4E-04	35,587	0,0173	0,0029	2,3E-09	-0,0004	0,0068	0,9560	instrument
rs942066	14	94031914	A	G	2,1E-04	55,972	-0,0202	0,0027	1,0E-13	0,0018	0,0065	0,7837	instrument
rs943005	6	50865820	T	C	4,3E-04	188,351	0,0398	0,0029	2,0E-43	0,0113	0,0082	0,1667	outlier
rs9531786	13	85983968	C	G	8,4E-05	33,062	-0,0138	0,0024	8,4E-09	-0,0088	0,0065	0,1743	instrument
rs9571687	13	67472713	A	C	9,2E-05	36,502	-0,0145	0,0024	3,2E-09	-0,0050	0,0068	0,4583	instrument
rs9579775	13	20616557	A	C	1,5E-04	39,690	-0,0252	0,0040	3,7E-10	0,0279	0,0117	0,0171	instrument
rs9595630	13	33065443	T	G	9,2E-05	39,745	0,0145	0,0023	2,0E-10	-0,0063	0,0064	0,3220	instrument
rs973345	9	29713169	T	C	9,7E-05	38,117	0,0142	0,0023	1,1E-09	0,0058	0,0063	0,3534	instrument
rs9906944	17	47091420	T	C	1,6E-04	62,673	-0,0190	0,0024	7,3E-15	-0,0031	0,0070	0,6522	instrument
rs9926784	16	19941968	T	C	1,9E-04	81,000	0,0252	0,0028	6,0E-20	-0,0108	0,0081	0,1823	instrument
rs9937053	16	53799507	A	G	2,2E-03	941,370	0,0675	0,0022	1,0E-200	-0,0578	0,0063	0,0000	outlier
rs994596	4	18459828	T	C	9,1E-05	36,000	0,0150	0,0025	1,0E-09	0,0047	0,0067	0,4878	instrument
rs9955276	18	1839339	T	C	1,2E-04	48,129	0,0222	0,0032	8,0E-12	-0,0010	0,0088	0,9129	instrument

Table S4. Single Nucleotide Polymorphisms (SNPs) used as instruments in the Mendelian randomization analyses for the effect of BMI on endometrial cancer based on the genome-wide significance threshold P = 5E-08. R^2 represents the explained variance in the exposure. SNP-specific instrument strength is given by the respective F-statistic. Some of the genetic variants were identified as outliers in the iterative approach and therefore excluded from the analyses.

SNP	CHR	POS	EA	OA	R^2	F-stat	β_{BMI}	SE_{BMI}	P_{BMI}	$\beta_{Endometrial}$	$SE_{Endometrial}$	$P_{Endometrial}$	$variant_{Endometrial}$
rs10002111	4	67815504	A	G	9,2E-05	36,430	0,0169	0,0028	9,3E-10	0,0024	0,0190	0,8982	instrument
rs1003081	11	118913993	T	C	9,9E-05	42,843	0,0144	0,0022	6,3E-11	-0,0039	0,0160	0,8068	instrument
rs10048652	2	35449306	T	C	8,7E-05	34,452	0,0135	0,0023	3,5E-09	0,0093	0,0160	0,5614	instrument
rs1009473	9	138777312	T	C	7,7E-05	30,489	-0,0127	0,0023	3,4E-08	-0,0221	0,0158	0,1637	instrument
rs10099330	8	143383694	A	G	8,3E-05	32,440	-0,0131	0,0023	9,9E-09	0,0151	0,0160	0,3466	instrument
rs10116186	9	80532374	A	G	1,2E-04	31,102	-0,0145	0,0026	4,1E-08	0,0007	0,0160	0,9660	instrument
rs10118701	9	103061366	A	G	1,4E-04	53,778	-0,0176	0,0024	2,2E-13	-0,0062	0,0169	0,7146	instrument
rs10124645	9	11321119	A	G	8,9E-05	34,964	0,0136	0,0023	5,0E-09	0,0277	0,0165	0,0946	instrument
rs10182181	2	25150296	A	G	6,2E-04	270,751	-0,0362	0,0022	5,0E-63	-0,0133	0,0163	0,4138	instrument
rs10237317	7	70045941	A	G	9,1E-05	39,347	-0,0138	0,0022	6,8E-10	-0,0040	0,0164	0,8050	instrument
rs10271582	7	99121950	A	G	1,2E-04	32,222	0,0193	0,0034	8,4E-09	0,0395	0,0222	0,0743	instrument
rs10471636	5	63979856	A	G	1,3E-04	50,173	-0,0170	0,0024	4,7E-13	0,0011	0,0166	0,9477	instrument
rs1048932	11	115044850	A	C	1,6E-04	68,438	-0,0182	0,0022	2,5E-16	0,0143	0,0161	0,3742	instrument
rs10499694	7	50614173	A	G	8,8E-05	38,215	0,0136	0,0022	4,0E-10	0,0273	0,0160	0,0876	instrument
rs10513801	3	185822353	T	G	2,8E-04	120,999	0,0352	0,0032	1,3E-27	-0,0077	0,0237	0,7459	instrument
rs10733682	9	129460914	A	G	1,2E-04	51,578	0,0158	0,0022	1,9E-12	0,0083	0,0159	0,6014	instrument
rs10742752	11	45438374	T	C	8,3E-05	36,000	-0,0132	0,0022	4,0E-09	-0,0097	0,0165	0,5562	instrument
rs10746306	12	84477374	T	G	1,3E-04	33,341	0,0179	0,0031	8,7E-09	-0,0345	0,0182	0,0578	instrument
rs1075901	17	15943910	T	C	9,9E-05	42,843	-0,0144	0,0022	4,4E-11	-0,0181	0,0159	0,2557	instrument
rs10761785	10	65318766	T	G	1,4E-04	61,124	-0,0172	0,0022	4,2E-15	-0,0115	0,0158	0,4675	instrument
rs10797115	9	92191256	T	C	1,2E-04	48,393	0,0160	0,0023	4,1E-12	-0,0212	0,0159	0,1805	instrument
rs10824347	10	77645229	A	T	1,1E-04	42,491	0,0176	0,0027	5,5E-11	0,0268	0,0187	0,1522	instrument
rs10920678	1	190239907	A	G	1,3E-04	54,894	0,0163	0,0022	7,5E-14	-0,0151	0,0162	0,3529	instrument
rs10938397	4	45182527	A	G	4,7E-04	205,009	-0,0315	0,0022	1,7E-45	-0,0292	0,0159	0,0669	instrument
rs10962550	9	16720329	C	G	1,0E-04	40,960	0,0192	0,0030	1,7E-10	-0,0108	0,0213	0,6098	instrument
rs10992867	9	96461013	A	G	1,3E-04	50,628	0,0185	0,0026	8,9E-13	-0,0289	0,0182	0,1116	instrument
rs11022762	11	13335926	T	C	1,0E-04	41,173	-0,0154	0,0024	5,6E-11	-0,0051	0,0162	0,7538	instrument
rs11073383	15	95274349	A	G	1,2E-04	47,790	-0,0159	0,0023	3,3E-12	-0,0286	0,0158	0,0703	instrument

rs11165643	1	96924097	T	C	1,6E-04	67,688	0,0181	0,0022	1,7E-16	0,0403	0,0163	0,0136	instrument
rs112646560	1	39560250	T	C	1,4E-04	37,515	0,0196	0,0032	1,1E-09	0,0396	0,0194	0,0414	instrument
rs11611246	12	939480	T	G	1,4E-04	61,651	0,0212	0,0027	1,3E-14	0,0140	0,0194	0,4695	instrument
rs11667280	19	30705747	C	G	7,5E-05	29,684	0,0158	0,0029	3,1E-08	0,0269	0,0208	0,1948	instrument
rs11672660	19	46180184	T	C	3,1E-04	131,429	-0,0321	0,0028	5,9E-31	-0,0348	0,0203	0,0873	instrument
rs11677607	2	100751150	T	C	2,0E-04	79,012	-0,0240	0,0027	2,9E-19	-0,0115	0,0187	0,5406	instrument
rs1167800	7	75176196	A	G	2,0E-04	82,644	0,0200	0,0022	3,7E-19	0,0055	0,0161	0,7312	instrument
rs11692326	2	208263279	T	C	8,3E-05	36,000	0,0156	0,0026	2,0E-09	0,0400	0,0189	0,0341	instrument
rs11727273	4	55509748	T	C	1,6E-04	42,250	-0,0169	0,0026	1,3E-10	-0,0195	0,0164	0,2342	instrument
rs11751591	6	33794215	A	G	8,5E-05	33,423	-0,0185	0,0032	1,2E-08	0,0128	0,0223	0,5661	instrument
rs11754747	6	141494602	T	C	7,3E-05	31,532	0,0146	0,0026	1,3E-08	-0,0242	0,0192	0,2091	instrument
rs11880870	19	18830704	A	G	1,8E-04	73,363	0,0197	0,0023	3,3E-18	0,0348	0,0164	0,0342	instrument
rs11945861	4	65700865	A	G	7,8E-05	30,864	-0,0150	0,0027	3,1E-08	-0,0016	0,0189	0,9315	instrument
rs11971098	7	26699061	A	G	8,5E-05	33,414	-0,0237	0,0041	6,5E-09	-0,0278	0,0281	0,3230	instrument
rs12042959	1	243533273	A	G	9,3E-05	36,754	0,0194	0,0032	2,3E-09	-0,0005	0,0224	0,9807	instrument
rs12089815	1	91189933	A	G	1,3E-04	34,628	-0,0153	0,0026	6,0E-09	-0,0227	0,0165	0,1685	instrument
rs12101625	15	26972431	C	G	8,4E-05	33,062	0,0138	0,0024	5,5E-09	-0,0340	0,0166	0,0404	instrument
rs12140153	1	62579891	T	G	1,8E-04	48,110	-0,0326	0,0047	2,9E-12	-0,0220	0,0289	0,4477	instrument
rs12150665	17	34914787	T	C	1,3E-04	57,622	0,0167	0,0022	5,9E-14	0,0075	0,0168	0,6545	instrument
rs12202969	6	98576223	A	G	1,0E-04	39,745	-0,0145	0,0023	1,9E-10	-0,0123	0,0160	0,4439	instrument
rs12207125	6	154301575	A	G	1,3E-04	34,468	0,0182	0,0031	7,7E-09	0,0271	0,0197	0,1689	instrument
rs12364470	11	134601012	T	G	1,0E-04	44,444	-0,0200	0,0030	2,5E-11	-0,0149	0,0231	0,5207	instrument
rs12429545	13	54102206	A	G	1,8E-04	77,760	0,0291	0,0033	1,1E-18	0,0367	0,0239	0,1244	instrument
rs12448257	16	3599655	A	G	7,2E-05	30,864	0,0150	0,0027	3,1E-08	0,0125	0,0198	0,5281	instrument
rs12462975	19	30272202	A	G	1,7E-04	65,934	0,0203	0,0025	2,6E-16	0,0023	0,0170	0,8903	instrument
rs12564992	1	174478100	A	G	8,0E-05	34,979	-0,0207	0,0035	2,4E-09	-0,0178	0,0251	0,4801	instrument
rs12593036	15	81058652	A	G	9,6E-05	37,945	0,0154	0,0025	6,6E-10	0,0364	0,0174	0,0360	instrument
rs12612009	2	228982603	T	G	8,7E-05	34,515	-0,0141	0,0024	3,8E-09	0,0134	0,0172	0,4363	instrument
rs12645001	4	96018337	A	G	8,7E-05	34,515	-0,0141	0,0024	4,1E-09	-0,0239	0,0167	0,1514	instrument
rs12680842	8	95582606	A	G	6,8E-05	29,340	0,0130	0,0024	3,4E-08	-0,0254	0,0168	0,1314	instrument
rs12681792	8	62054463	A	C	1,0E-04	39,820	0,0183	0,0029	2,6E-10	0,0626	0,0206	0,0024	instrument
rs12692596	2	161265910	T	C	9,3E-05	40,295	0,0146	0,0023	9,7E-11	0,0220	0,0164	0,1798	instrument

rs12692738	2	165558252	T	C	8,9E-05	38,822	-0,0162	0,0026	2,8E-10	0,0101	0,0188	0,5923	instrument
rs12714199	2	86812549	T	C	1,0E-04	40,295	-0,0146	0,0023	5,1E-10	-0,0267	0,0164	0,1039	instrument
rs1285997	14	91513029	C	G	9,2E-05	36,000	-0,0150	0,0025	3,2E-09	-0,0222	0,0176	0,2059	instrument
rs12872889	13	28674628	T	C	1,7E-04	45,453	-0,0209	0,0031	1,5E-11	0,0235	0,0191	0,2173	instrument
rs1289736	3	107950892	T	C	9,3E-05	36,523	0,0139	0,0023	1,4E-09	0,0112	0,0161	0,4851	instrument
rs12914489	15	74187937	A	G	9,1E-05	39,760	0,0227	0,0036	1,6E-10	0,0418	0,0257	0,1032	instrument
rs12949279	17	78558411	T	C	1,5E-04	66,200	0,0179	0,0022	6,1E-16	-0,0075	0,0162	0,6431	instrument
rs12981256	19	1865901	A	G	1,6E-04	62,616	0,0182	0,0023	3,9E-15	0,0010	0,0162	0,9511	instrument
rs13002158	2	144021712	A	G	1,3E-04	51,443	-0,0208	0,0029	1,2E-12	-0,0611	0,0209	0,0034	instrument
rs13053342	22	48871624	A	G	1,0E-04	40,640	0,0153	0,0024	1,3E-10	0,0102	0,0168	0,5444	instrument
rs13062093	3	35667057	T	G	8,3E-05	32,585	-0,0137	0,0024	8,0E-09	-0,0061	0,0164	0,7104	instrument
rs13076052	3	44456573	C	G	1,2E-04	32,766	-0,0166	0,0029	1,3E-08	0,0098	0,0184	0,5919	instrument
rs13107325	4	103188709	T	C	2,4E-04	105,528	0,0452	0,0044	9,3E-25	-0,0106	0,0315	0,7356	instrument
rs13110266	4	162129844	A	G	9,6E-05	41,661	-0,0142	0,0022	1,5E-10	-0,0060	0,0162	0,7115	instrument
rs13174863	5	139080745	A	G	1,8E-04	75,858	-0,0270	0,0031	4,5E-18	-0,0198	0,0240	0,4074	instrument
rs13209872	6	97753223	C	G	8,9E-05	35,007	-0,0142	0,0024	6,1E-09	-0,0176	0,0170	0,3009	instrument
rs13213408	6	162998049	A	T	9,0E-05	35,637	0,0197	0,0033	4,3E-09	-0,0133	0,0233	0,5665	instrument
rs1362910	8	30856464	A	G	8,2E-05	33,943	0,0134	0,0023	3,0E-09	0,0329	0,0161	0,0408	instrument
rs1403846	4	119101723	T	C	7,2E-05	31,206	0,0162	0,0029	1,6E-08	-0,0140	0,0209	0,5039	instrument
rs1409818	20	21381121	T	C	1,2E-04	46,387	0,0252	0,0037	1,6E-11	0,0380	0,0261	0,1457	instrument
rs141729694	5	87999371	T	C	2,2E-04	57,284	0,0386	0,0051	2,5E-14	0,0367	0,0298	0,2176	instrument
rs1417665	6	104816944	T	C	1,2E-04	47,088	0,0199	0,0029	5,7E-12	-0,0052	0,0205	0,7998	instrument
rs1421598	2	50264710	A	C	9,7E-05	38,360	-0,0192	0,0031	6,8E-10	-0,0287	0,0212	0,1766	instrument
rs1436348	3	104612668	A	G	8,6E-05	33,943	-0,0134	0,0023	6,3E-09	-0,0152	0,0162	0,3506	instrument
rs1441264	13	79580919	A	G	1,3E-04	55,276	0,0171	0,0023	5,2E-14	-0,0041	0,0168	0,8070	instrument
rs1452075	3	62481063	T	C	9,4E-05	40,449	0,0159	0,0025	1,5E-10	0,0387	0,0183	0,0343	instrument
rs1491333	4	172186288	T	G	8,1E-05	31,966	-0,0147	0,0026	9,9E-09	-0,0255	0,0186	0,1705	instrument
rs1492014	3	94071481	T	C	1,0E-04	39,745	-0,0145	0,0023	2,8E-10	-0,0063	0,0160	0,6942	instrument
rs1561589	10	126695673	A	G	1,1E-04	47,191	0,0158	0,0023	4,2E-12	0,0289	0,0164	0,0787	instrument
rs1569979	14	29681294	A	G	1,8E-04	72,565	0,0230	0,0027	1,6E-17	0,0246	0,0189	0,1930	instrument
rs1579557	6	40371918	T	C	1,8E-04	71,910	0,0212	0,0025	3,1E-17	0,0359	0,0181	0,0473	instrument
rs158186	5	107440856	A	G	2,2E-04	85,711	-0,0287	0,0031	3,2E-20	-0,0451	0,0213	0,0339	instrument

rs159032	5	94206202	T	C	7,8E-05	30,454	0,0149	0,0027	4,5E-08	-0,0047	0,0183	0,7980	instrument
rs1681743	10	118565501	T	C	7,5E-05	29,793	-0,0131	0,0024	3,7E-08	-0,0191	0,0163	0,2428	instrument
rs16851483	3	141275436	T	G	1,6E-04	62,272	0,0363	0,0046	2,3E-15	0,0201	0,0324	0,5349	instrument
rs16912921	9	28413461	A	C	2,7E-04	117,204	0,0249	0,0023	1,6E-26	0,0216	0,0169	0,2009	instrument
rs16926778	12	24002125	C	G	9,3E-05	36,560	0,0260	0,0043	1,4E-09	0,0056	0,0304	0,8537	instrument
rs16975921	18	39914870	A	T	1,1E-04	42,510	-0,0163	0,0025	3,9E-11	-0,0036	0,0169	0,8334	instrument
rs16989232	20	39291784	A	G	7,1E-05	30,972	0,0128	0,0023	1,5E-08	-0,0108	0,0167	0,5162	instrument
rs17014375	1	209543560	T	G	7,9E-05	31,228	-0,0190	0,0034	1,5E-08	-0,0030	0,0232	0,8982	instrument
rs17033633	12	103656343	T	C	1,2E-04	48,534	-0,0209	0,0030	7,3E-12	-0,0378	0,0211	0,0734	instrument
rs17066856	18	58049656	T	C	2,7E-04	115,707	0,0398	0,0037	1,4E-27	-0,0035	0,0276	0,8990	instrument
rs17405819	8	76806584	T	C	1,9E-04	82,507	0,0218	0,0024	7,8E-20	0,0294	0,0174	0,0908	instrument
rs1750307	1	156488420	A	T	8,3E-05	32,585	0,0137	0,0024	8,2E-09	0,0309	0,0163	0,0585	instrument
rs17508240	12	117577358	A	G	1,4E-04	35,626	0,0191	0,0032	2,8E-09	-0,0287	0,0194	0,1392	instrument
rs17522122	14	33302882	T	G	1,3E-04	57,622	0,0167	0,0022	6,5E-14	0,0307	0,0162	0,0577	instrument
rs17619973	3	114417675	A	G	7,4E-05	31,673	0,0242	0,0043	1,3E-08	0,0096	0,0317	0,7620	instrument
rs17724992	19	18454825	A	G	1,1E-04	46,240	0,0170	0,0025	6,7E-12	0,0377	0,0181	0,0368	instrument
rs17751061	19	19413092	T	C	8,2E-05	31,993	-0,0181	0,0032	1,3E-08	-0,0105	0,0220	0,6326	instrument
rs17767765	16	24539793	T	C	7,7E-05	30,489	-0,0127	0,0023	2,6E-08	-0,0102	0,0165	0,5379	instrument
rs17806224	20	51065854	A	G	2,3E-04	92,801	-0,0289	0,0030	3,5E-22	-0,0434	0,0211	0,0394	instrument
rs1799923	3	42306294	A	G	1,2E-04	48,203	-0,0243	0,0035	5,1E-12	0,0386	0,0254	0,1292	instrument
rs1801282	3	12393125	C	G	7,0E-05	30,417	-0,0182	0,0033	4,7E-08	0,0737	0,0242	0,0024	outlier
rs1808579	18	21104888	T	C	1,9E-04	81,000	-0,0198	0,0022	9,4E-20	-0,0234	0,0159	0,1419	instrument
rs1808629	8	73435964	A	G	2,5E-04	65,147	-0,0226	0,0028	5,8E-16	0,0089	0,0174	0,6100	instrument
rs1812587	5	62991802	T	G	1,3E-04	34,177	0,0152	0,0026	7,0E-09	0,0077	0,0160	0,6290	instrument
rs185350	19	34306816	T	C	7,8E-05	33,851	0,0128	0,0022	7,1E-09	-0,0077	0,0159	0,6262	instrument
rs1861412	2	58893065	A	G	2,2E-04	87,381	-0,0215	0,0023	9,1E-21	-0,0029	0,0159	0,8576	instrument
rs1884389	20	1410582	T	C	9,4E-05	37,051	-0,0140	0,0023	1,2E-09	0,0192	0,0160	0,2283	instrument
rs1884897	20	6612832	A	G	1,2E-04	53,990	-0,0169	0,0023	9,1E-14	-0,0284	0,0165	0,0866	instrument
rs1916801	3	61187046	A	T	1,4E-04	59,710	0,0170	0,0022	1,8E-14	-0,0183	0,0162	0,2610	instrument
rs1927790	13	96922191	T	C	8,7E-05	37,655	-0,0135	0,0022	1,3E-09	-0,0071	0,0162	0,6602	instrument
rs1928295	9	120378483	T	C	1,2E-04	52,892	0,0160	0,0022	2,8E-13	0,0024	0,0160	0,8801	instrument
rs197374	1	112289983	T	C	1,4E-04	55,276	0,0171	0,0023	2,8E-13	0,0202	0,0164	0,2184	instrument

rs1984559	2	135678888	A	G	7,9E-05	29,521	0,0163	0,0030	3,3E-08	0,0009	0,0198	0,9632	instrument
rs198665	6	143154886	A	C	7,8E-05	33,943	0,0134	0,0023	4,4E-09	-0,0045	0,0165	0,7877	instrument
rs2012502	16	81728081	A	C	1,0E-04	44,251	0,0153	0,0023	3,0E-11	-0,0186	0,0167	0,2637	instrument
rs2045293	7	114350102	T	C	1,7E-04	67,525	0,0189	0,0023	4,1E-16	-0,0218	0,0161	0,1763	instrument
rs2053682	5	170599327	A	C	1,4E-04	53,582	0,0183	0,0025	1,9E-13	-0,0154	0,0171	0,3689	instrument
rs209416	6	83534729	A	T	1,2E-04	47,840	0,0166	0,0024	8,7E-12	0,0136	0,0170	0,4231	instrument
rs2112347	5	75015242	T	G	4,2E-04	180,493	0,0309	0,0023	2,1E-42	0,0063	0,0165	0,7020	instrument
rs214249	16	348687	T	G	1,2E-04	48,418	0,0167	0,0024	1,4E-12	-0,0027	0,0162	0,8667	instrument
rs2161097	5	103945178	T	C	8,0E-05	31,457	0,0129	0,0023	2,3E-08	0,0114	0,0159	0,4724	instrument
rs2178899	6	31606756	A	T	1,3E-04	56,477	0,0248	0,0033	7,2E-14	0,0327	0,0245	0,1828	instrument
rs2185027	6	153381622	A	C	9,5E-05	41,173	-0,0154	0,0024	1,1E-10	-0,0117	0,0173	0,4991	instrument
rs2228213	6	12124855	A	G	9,5E-05	41,406	-0,0148	0,0023	9,2E-11	-0,0366	0,0165	0,0267	instrument
rs2267812	7	74138121	A	C	9,2E-05	35,177	-0,0172	0,0029	1,7E-09	-0,0251	0,0205	0,2211	instrument
rs2267958	9	131015279	A	G	1,2E-04	45,002	-0,0161	0,0024	1,9E-11	0,0025	0,0168	0,8800	instrument
rs2271046	4	52752812	A	T	7,6E-05	30,030	-0,0137	0,0025	2,4E-08	-0,0329	0,0175	0,0608	instrument
rs2271189	12	56494991	A	G	9,5E-05	37,007	-0,0146	0,0024	6,4E-10	-0,0302	0,0166	0,0679	instrument
rs2279574	12	89745477	A	C	1,4E-04	51,465	-0,0165	0,0023	2,0E-12	-0,0039	0,0168	0,8175	instrument
rs2289292	16	30097630	T	C	1,5E-04	57,760	-0,0190	0,0025	1,1E-14	-0,0088	0,0175	0,6142	instrument
rs2289705	7	121983843	A	G	7,7E-05	33,284	-0,0150	0,0026	1,5E-08	0,0087	0,0197	0,6583	instrument
rs2299383	7	103418846	T	C	1,6E-04	68,438	0,0182	0,0022	2,7E-16	-0,0010	0,0161	0,9512	instrument
rs2357760	6	120213880	A	G	1,1E-04	47,790	0,0159	0,0023	1,1E-11	0,0101	0,0170	0,5519	instrument
rs2470520	3	15762920	T	C	7,6E-05	30,011	-0,0126	0,0023	4,2E-08	0,0087	0,0160	0,5860	instrument
rs247975	3	173107443	T	C	1,6E-04	62,616	-0,0182	0,0023	3,6E-15	0,0049	0,0167	0,7694	instrument
rs2528531	7	93236510	A	C	9,3E-05	36,502	-0,0145	0,0024	1,3E-09	-0,0027	0,0165	0,8693	instrument
rs2568958	1	72765116	A	G	2,5E-04	106,464	0,0227	0,0022	1,3E-24	0,0102	0,0162	0,5312	instrument
rs2605603	11	93221105	A	G	8,3E-05	36,000	-0,0132	0,0022	1,4E-09	-0,0185	0,0159	0,2435	instrument
rs2619976	17	71754545	T	C	9,4E-05	37,007	0,0146	0,0024	6,2E-10	-0,0153	0,0169	0,3635	instrument
rs2712643	12	99638276	A	C	8,7E-05	37,945	-0,0154	0,0025	1,5E-09	0,0087	0,0183	0,6330	instrument
rs2721965	8	116662038	A	C	1,5E-04	60,062	0,0186	0,0024	2,1E-14	0,0354	0,0168	0,0356	instrument
rs273504	19	18215247	A	G	1,1E-04	43,102	-0,0151	0,0023	1,3E-10	-0,0257	0,0160	0,1084	instrument
rs2754079	14	25886140	A	G	1,2E-04	46,004	-0,0156	0,0023	2,3E-11	-0,0062	0,0164	0,7053	instrument
rs2814992	6	34617144	A	G	3,6E-04	155,706	-0,0287	0,0023	1,2E-35	-0,0025	0,0168	0,8828	instrument

rs2836754	21	40291740	T	C	1,1E-04	45,870	-0,0149	0,0022	3,7E-11	-0,0047	0,0164	0,7759	instrument
rs28489620	22	41804716	A	G	1,5E-04	38,955	-0,0181	0,0029	6,9E-10	-0,0267	0,0177	0,1319	instrument
rs2861685	2	67837553	T	C	1,8E-04	47,929	0,0180	0,0026	1,1E-11	0,0222	0,0161	0,1668	instrument
rs2862961	11	43656535	A	G	2,3E-04	91,393	-0,0239	0,0025	5,3E-22	0,0123	0,0171	0,4714	instrument
rs2959592	8	17002725	T	C	8,7E-05	36,862	0,0170	0,0028	6,3E-10	0,0250	0,0207	0,2276	instrument
rs2968864	7	150622162	T	C	9,2E-05	36,446	0,0163	0,0027	9,3E-10	0,0035	0,0186	0,8524	instrument
rs2984618	1	47690438	T	G	1,3E-04	57,622	0,0167	0,0022	2,2E-14	0,0080	0,0161	0,6198	instrument
rs329651	11	133767622	T	G	9,7E-05	41,787	0,0181	0,0028	2,0E-10	0,0307	0,0207	0,1382	instrument
rs337637	4	38604470	A	G	6,8E-05	29,537	-0,0125	0,0023	3,0E-08	-0,0313	0,0166	0,0593	instrument
rs34517439	1	78450517	A	C	3,4E-04	90,481	0,0390	0,0041	2,0E-21	0,0424	0,0254	0,0947	instrument
rs34811474	4	25408838	A	G	4,0E-04	104,566	-0,0317	0,0031	5,2E-24	0,0128	0,0220	0,5591	instrument
rs34930419	10	33987329	T	C	1,4E-04	36,316	-0,0458	0,0076	1,4E-09	-0,0518	0,0466	0,2662	instrument
rs35020117	14	73341655	T	G	1,4E-04	37,345	0,0165	0,0027	1,2E-09	0,0216	0,0171	0,2064	instrument
rs35107973	4	143125948	T	C	1,2E-04	32,573	0,0234	0,0041	7,7E-09	-0,0049	0,0255	0,8469	instrument
rs355754	3	154024165	T	C	9,2E-05	38,117	-0,0142	0,0023	4,3E-10	-0,0032	0,0163	0,8440	instrument
rs35722922	2	47002226	A	G	1,8E-04	48,482	0,0188	0,0027	3,6E-12	0,0269	0,0164	0,1012	instrument
rs3803286	14	103246470	A	G	1,3E-04	51,361	0,0172	0,0024	8,3E-13	-0,0043	0,0167	0,7966	instrument
rs3826408	17	7101292	T	C	7,0E-05	30,250	0,0121	0,0022	4,5E-08	0,0132	0,0159	0,4084	instrument
rs3829849	9	129390800	T	C	8,0E-05	34,964	0,0136	0,0023	1,9E-09	0,0132	0,0165	0,4251	instrument
rs3977755	10	104420210	T	C	8,0E-05	32,718	-0,0143	0,0025	8,5E-09	0,0287	0,0176	0,1032	instrument
rs4055791	13	59266053	T	C	1,5E-04	39,643	-0,0170	0,0027	1,8E-10	-0,0298	0,0163	0,0684	instrument
rs4148155	4	89054667	A	G	7,7E-05	33,640	0,0203	0,0035	6,2E-09	-0,0109	0,0252	0,6662	instrument
rs4240673	8	10787612	T	C	1,5E-04	64,000	0,0176	0,0022	1,0E-15	0,0060	0,0193	0,7558	instrument
rs427943	21	46570896	A	C	1,5E-04	59,223	-0,0177	0,0023	1,4E-14	0,0020	0,0160	0,9010	instrument
rs429358	19	45411941	T	C	2,2E-04	58,777	0,0276	0,0036	3,2E-14	0,0656	0,0233	0,0049	instrument
rs4295917	1	32173360	A	G	8,2E-05	31,640	0,0135	0,0024	1,6E-08	-0,0007	0,0186	0,9689	instrument
rs4307239	7	24354300	A	G	9,5E-05	37,582	-0,0141	0,0023	1,3E-09	-0,0127	0,0162	0,4344	instrument
rs4320543	8	34422967	A	G	8,0E-05	31,457	-0,0129	0,0023	2,9E-08	-0,0124	0,0166	0,4558	instrument
rs4421623	1	98316285	T	G	1,2E-04	46,441	0,0184	0,0027	1,1E-11	0,0061	0,0189	0,7478	instrument
rs4459316	11	130756176	T	C	1,1E-04	44,832	-0,0154	0,0023	2,3E-11	0,0032	0,0162	0,8445	instrument
rs4474229	1	195037001	A	G	1,6E-04	41,055	-0,0173	0,0027	1,6E-10	0,0073	0,0163	0,6546	instrument
rs4482463	2	205375909	A	C	2,0E-04	75,524	-0,0365	0,0042	5,2E-18	0,0064	0,0301	0,8315	instrument

rs4569924	5	153540025	T	C	1,2E-04	51,578	0,0158	0,0022	7,4E-13	-0,0012	0,0160	0,9405	instrument
rs4586287	13	99245334	C	G	7,7E-05	30,470	0,0138	0,0025	2,9E-08	0,0186	0,0171	0,2774	instrument
rs4673553	2	211608379	T	G	1,1E-04	44,251	-0,0153	0,0023	2,6E-11	0,0260	0,0160	0,1047	instrument
rs4677812	3	194863794	A	C	8,4E-05	33,177	-0,0144	0,0025	9,4E-09	-0,0042	0,0179	0,8133	instrument
rs4776982	15	68114974	A	G	3,3E-04	143,078	0,0311	0,0026	4,2E-33	0,0517	0,0192	0,0070	instrument
rs4783718	16	69547741	T	C	1,8E-04	78,669	0,0204	0,0023	1,8E-19	0,0226	0,0166	0,1727	instrument
rs4790292	17	1824305	A	C	1,7E-04	63,516	-0,0263	0,0033	1,7E-15	0,0205	0,0228	0,3684	instrument
rs4834272	4	113313986	T	C	8,9E-05	38,656	-0,0143	0,0023	8,2E-10	-0,0273	0,0173	0,1135	instrument
rs4858223	3	20581149	T	C	1,1E-04	41,473	-0,0161	0,0025	8,3E-11	-0,0138	0,0172	0,4219	instrument
rs4864201	4	130731284	T	C	7,5E-05	32,440	0,0131	0,0023	6,3E-09	0,0142	0,0165	0,3896	instrument
rs4880341	10	133992689	T	C	9,8E-05	38,656	-0,0143	0,0023	4,5E-10	0,0056	0,0160	0,7276	instrument
rs4889606	16	31011183	A	G	3,1E-04	135,773	0,0268	0,0023	1,3E-32	0,0205	0,0165	0,2139	instrument
rs4929923	11	8639200	T	C	2,1E-04	92,327	-0,0221	0,0023	2,5E-22	-0,0229	0,0166	0,1674	instrument
rs4936175	11	132641959	T	C	1,2E-04	47,191	-0,0158	0,0023	6,7E-12	-0,0096	0,0160	0,5483	instrument
rs4969387	17	79081724	C	G	8,4E-05	32,532	-0,0154	0,0027	6,9E-09	-0,0280	0,0183	0,1265	instrument
rs4986044	17	21261560	T	C	1,4E-04	61,836	-0,0173	0,0022	2,2E-15	0,0203	0,0160	0,2043	instrument
rs543874	1	177889480	A	G	1,0E-03	455,109	-0,0576	0,0027	8,7E-100	-0,0020	0,0198	0,9208	instrument
rs56161855	17	46288649	A	T	1,7E-04	44,787	-0,0261	0,0039	1,5E-11	0,0148	0,0231	0,5203	instrument
rs563132	9	37245476	A	T	1,4E-04	56,250	0,0180	0,0024	2,0E-14	-0,0081	0,0163	0,6167	instrument
rs563296	10	99772404	A	G	1,4E-04	53,168	0,0175	0,0024	8,8E-14	0,0084	0,0164	0,6070	instrument
rs57221746	15	99230035	A	G	1,2E-04	30,594	-0,0177	0,0032	2,1E-08	-0,0004	0,0190	0,9853	instrument
rs6010784	20	61540319	T	C	7,6E-05	32,802	0,0126	0,0022	1,1E-08	0,0012	0,0158	0,9402	instrument
rs6142096	20	32686658	A	G	8,6E-05	33,943	0,0134	0,0023	4,2E-09	0,0062	0,0158	0,6953	instrument
rs61813324	1	156049877	T	C	2,2E-04	58,385	0,0298	0,0039	3,6E-14	0,0120	0,0254	0,6375	instrument
rs62007782	15	78029797	A	G	1,4E-04	35,601	-0,0179	0,0030	1,9E-09	-0,0305	0,0182	0,0942	instrument
rs62106258	2	417167	T	C	9,7E-04	254,452	0,0989	0,0062	8,7E-58	0,1133	0,0461	0,0140	instrument
rs62412826	6	61881167	T	C	1,3E-04	33,518	0,0330	0,0057	8,0E-09	-0,0126	0,0361	0,7276	instrument
rs625652	11	65641931	A	T	2,0E-04	52,160	-0,0195	0,0027	9,1E-13	0,0062	0,0166	0,7109	instrument
rs6265	11	27679916	T	C	4,0E-04	172,734	-0,0368	0,0028	8,0E-40	-0,0482	0,0203	0,0174	instrument
rs6446187	3	49907111	A	C	4,3E-04	114,325	0,0278	0,0026	1,2E-26	-0,0131	0,0158	0,4076	instrument
rs645026	12	69757931	A	G	9,6E-05	37,870	-0,0160	0,0026	4,8E-10	0,0034	0,0184	0,8518	instrument
rs6489156	12	123100397	T	C	1,8E-04	72,905	0,0222	0,0026	1,0E-17	0,0255	0,0179	0,1538	instrument

rs6493498	15	51754451	T	C	1,3E-04	54,894	0,0163	0,0022	1,5E-13	-0,0018	0,0160	0,9102	instrument
rs6548237	2	621461	A	C	9,1E-04	394,500	-0,0576	0,0029	4,4E-89	-0,0428	0,0263	0,1032	instrument
rs6556810	5	164585949	T	C	7,4E-05	29,066	-0,0124	0,0023	5,0E-08	-0,0380	0,0161	0,0181	instrument
rs6561937	13	58257667	A	T	1,4E-04	53,236	-0,0197	0,0027	6,0E-13	-0,0214	0,0188	0,2536	instrument
rs6567160	18	57829135	T	C	1,1E-03	475,573	-0,0567	0,0026	8,7E-108	-0,0129	0,0186	0,4880	instrument
rs657452	1	49589847	A	G	2,0E-04	85,142	0,0203	0,0022	1,1E-19	0,0099	0,0163	0,5423	instrument
rs6585198	10	114762237	A	G	1,1E-04	49,000	-0,0154	0,0022	1,2E-12	-0,0128	0,0159	0,4214	instrument
rs6606686	12	110903380	C	G	1,4E-04	59,894	-0,0178	0,0023	2,5E-14	-0,0139	0,0171	0,4169	instrument
rs6669189	1	75011358	T	C	2,1E-04	91,115	0,0210	0,0022	4,7E-21	0,0050	0,0161	0,7550	instrument
rs6720868	2	230663576	T	C	1,3E-04	49,585	0,0169	0,0024	4,6E-12	-0,0292	0,0169	0,0834	instrument
rs6728037	2	59297346	A	G	2,1E-04	81,000	0,0207	0,0023	6,5E-19	0,0280	0,0163	0,0851	instrument
rs6739199	2	105452326	T	C	1,0E-04	40,449	0,0159	0,0025	3,2E-10	0,0221	0,0183	0,2266	instrument
rs6870983	5	87697533	T	C	1,5E-04	66,485	-0,0212	0,0026	9,6E-16	-0,0443	0,0193	0,0216	instrument
rs6890310	5	27193573	A	G	9,6E-05	37,945	-0,0154	0,0025	1,4E-09	-0,0263	0,0180	0,1445	instrument
rs6973700	7	1856669	A	G	1,4E-04	36,376	0,0193	0,0032	1,8E-09	0,0509	0,0197	0,0098	instrument
rs7024334	9	109072075	T	G	8,7E-05	37,398	0,0159	0,0026	1,6E-09	0,0345	0,0189	0,0671	instrument
rs7084454	10	21821274	A	G	2,1E-04	81,000	0,0225	0,0025	7,4E-20	0,0439	0,0167	0,0085	instrument
rs709400	14	104149475	A	G	1,5E-04	64,000	0,0184	0,0023	4,4E-16	-0,0007	0,0163	0,9676	instrument
rs7105462	11	112912048	A	G	9,9E-05	39,198	0,0144	0,0023	4,4E-10	-0,0086	0,0161	0,5953	instrument
rs7107409	11	76492195	T	C	1,2E-04	30,941	-0,0178	0,0032	2,1E-08	0,0124	0,0192	0,5202	instrument
rs7124681	11	47529947	A	C	2,9E-04	126,051	0,0247	0,0022	9,3E-29	0,0225	0,0161	0,1616	instrument
rs7133378	12	124409502	A	G	1,0E-04	43,340	0,0158	0,0024	1,9E-11	-0,0054	0,0173	0,7532	instrument
rs7138803	12	50247468	A	G	3,7E-04	160,077	0,0291	0,0023	9,7E-38	0,0301	0,0162	0,0630	instrument
rs7141420	14	79899454	T	C	2,1E-04	91,115	0,0210	0,0022	7,8E-22	0,0518	0,0164	0,0016	instrument
rs7211966	17	54374611	T	C	8,9E-05	34,515	-0,0470	0,0080	4,9E-09	0,0678	0,0633	0,2838	instrument
rs7239114	18	45921214	A	G	1,2E-04	49,638	0,0155	0,0022	3,9E-12	0,0201	0,0164	0,2210	instrument
rs7250833	19	33937277	T	C	8,9E-05	38,543	0,0149	0,0024	7,2E-10	0,0091	0,0175	0,6038	instrument
rs73124396	7	71579606	T	C	1,2E-04	32,111	0,0187	0,0033	1,1E-08	0,0262	0,0197	0,1833	instrument
rs73213484	4	28489339	A	T	1,7E-04	45,653	0,0250	0,0037	2,3E-11	0,0290	0,0225	0,1977	instrument
rs7335249	13	109962579	T	C	8,7E-05	34,452	0,0135	0,0023	4,9E-09	0,0206	0,0163	0,2051	instrument
rs7395632	11	958789	T	C	7,4E-05	29,340	-0,0130	0,0024	3,9E-08	-0,0089	0,0166	0,5920	instrument
rs7428670	3	131579810	C	G	1,5E-04	60,361	0,0202	0,0026	3,1E-15	-0,0003	0,0179	0,9850	instrument

rs7498044	15	92573639	A	G	1,2E-04	31,640	-0,0180	0,0032	2,7E-08	0,0127	0,0198	0,5221	instrument
rs7498665	16	28883241	A	G	3,3E-04	143,999	-0,0264	0,0022	1,9E-32	-0,0535	0,0161	0,0009	instrument
rs750090	4	152931436	T	C	1,1E-04	42,250	0,0156	0,0024	7,4E-11	-0,0041	0,0169	0,8091	instrument
rs7550711	1	110082886	T	C	2,1E-04	88,858	0,0641	0,0068	3,4E-21	0,0708	0,0458	0,1222	instrument
rs7571496	2	6169351	A	G	7,9E-05	31,102	0,0145	0,0026	1,4E-08	-0,0020	0,0183	0,9109	instrument
rs7588437	2	181575281	A	G	1,3E-04	58,555	-0,0176	0,0023	8,4E-15	-0,0037	0,0167	0,8262	instrument
rs7601870	2	15384392	A	G	8,3E-05	32,937	-0,0132	0,0023	1,2E-08	-0,0174	0,0161	0,2810	instrument
rs7601895	2	55281901	C	G	1,1E-04	41,473	0,0161	0,0025	9,6E-11	0,0121	0,0177	0,4950	instrument
rs7621025	3	136272246	T	C	1,5E-04	64,000	-0,0200	0,0025	1,6E-15	-0,0135	0,0181	0,4562	instrument
rs7647242	3	82642734	T	C	1,0E-04	39,745	0,0145	0,0023	3,9E-10	-0,0057	0,0164	0,7287	instrument
rs768023	6	108876002	A	G	1,6E-04	70,712	0,0185	0,0022	1,4E-16	0,0074	0,0163	0,6489	instrument
rs76900466	2	145057760	T	G	1,2E-04	30,654	0,0227	0,0041	4,5E-08	0,0014	0,0319	0,9656	instrument
rs77432547	13	86494817	A	G	1,4E-04	36,415	-0,0175	0,0029	2,3E-09	0,0030	0,0177	0,8630	instrument
rs7788008	7	112972483	A	G	1,4E-04	55,276	-0,0171	0,0023	1,1E-13	-0,0068	0,0160	0,6728	instrument
rs7805441	7	78121458	T	C	1,0E-04	40,295	0,0146	0,0023	2,0E-10	0,0086	0,0159	0,5892	instrument
rs782971	5	43124688	A	G	1,1E-04	47,334	-0,0172	0,0025	4,9E-12	-0,0470	0,0186	0,0115	instrument
rs786420	2	44719893	T	C	8,4E-05	32,956	0,0155	0,0027	8,8E-09	-0,0247	0,0185	0,1827	instrument
rs78886584	1	16859325	A	G	1,3E-04	33,284	-0,0150	0,0026	1,3E-08	-0,0233	0,0189	0,2178	instrument
rs7899106	10	87410904	A	G	1,3E-04	56,250	-0,0375	0,0050	7,6E-14	-0,0353	0,0368	0,3363	instrument
rs79113395	1	1590521	A	G	1,9E-04	49,000	-0,0210	0,0030	1,5E-12	-0,0011	0,0299	0,9695	instrument
rs7976757	12	19207948	T	C	1,0E-04	40,960	-0,0192	0,0030	2,9E-10	0,0381	0,0211	0,0711	instrument
rs8024806	15	53473990	T	C	8,5E-05	33,492	0,0272	0,0047	9,8E-09	0,0770	0,0343	0,0246	instrument
rs8033510	15	61445514	T	C	8,3E-05	32,585	0,0137	0,0024	1,1E-08	-0,0191	0,0164	0,2451	instrument
rs8038522	15	46585105	T	G	9,4E-05	37,051	-0,0140	0,0023	1,3E-09	0,0285	0,0160	0,0739	instrument
rs8075273	17	61728881	A	C	1,0E-04	44,444	-0,0160	0,0024	5,6E-11	0,0103	0,0175	0,5570	instrument
rs811054	16	72251132	T	C	1,2E-04	54,223	0,0162	0,0022	3,2E-13	-0,0103	0,0160	0,5212	instrument
rs836525	7	6465909	T	C	8,2E-05	32,233	0,0176	0,0031	9,8E-09	0,0279	0,0216	0,1955	instrument
rs879620	16	4015729	T	C	2,9E-04	115,328	0,0247	0,0023	5,4E-26	0,0126	0,0166	0,4453	instrument
rs893006	11	64365796	A	C	1,2E-04	51,960	0,0173	0,0024	4,0E-13	0,0069	0,0176	0,6932	instrument
rs895330	19	4060707	C	G	9,9E-05	38,955	0,0181	0,0029	6,6E-10	0,0168	0,0209	0,4222	instrument
rs915814	21	46493003	A	G	1,3E-04	34,810	-0,0177	0,0030	5,9E-09	0,0121	0,0188	0,5195	instrument
rs925018	3	62713143	C	G	8,8E-05	38,117	-0,0142	0,0023	9,8E-10	0,0111	0,0170	0,5154	instrument

rs9304665	19	47602577	A	T	1,4E-04	57,086	0,0204	0,0027	1,8E-14	-0,0024	0,0189	0,8981	instrument
rs942066	14	94031914	A	G	2,1E-04	55,972	-0,0202	0,0027	1,0E-13	0,0150	0,0165	0,3648	instrument
rs943005	6	50865820	T	C	4,3E-04	188,351	0,0398	0,0029	2,0E-43	0,0259	0,0208	0,2126	instrument
rs9531786	13	85983968	C	G	8,4E-05	33,062	-0,0138	0,0024	8,4E-09	-0,0026	0,0166	0,8765	instrument
rs9571687	13	67472713	A	C	9,2E-05	36,502	-0,0145	0,0024	3,2E-09	0,0223	0,0170	0,1892	instrument
rs9579775	13	20616557	A	C	1,5E-04	39,690	-0,0252	0,0040	3,7E-10	-0,0327	0,0298	0,2716	instrument
rs9595630	13	33065443	T	G	9,2E-05	39,745	0,0145	0,0023	2,0E-10	0,0030	0,0163	0,8525	instrument
rs973345	9	29713169	T	C	9,7E-05	38,117	0,0142	0,0023	1,1E-09	0,0133	0,0161	0,4094	instrument
rs9906944	17	47091420	T	C	1,6E-04	62,673	-0,0190	0,0024	7,3E-15	-0,0152	0,0173	0,3815	instrument
rs9926784	16	19941968	T	C	1,9E-04	81,000	0,0252	0,0028	6,0E-20	0,0572	0,0206	0,0055	instrument
rs9937053	16	53799507	A	G	2,2E-03	941,370	0,0675	0,0022	1,0E-200	0,0517	0,0159	0,0011	instrument
rs994596	4	18459828	T	C	9,1E-05	36,000	0,0150	0,0025	1,0E-09	0,0164	0,0170	0,3336	instrument
rs9955276	18	1839339	T	C	1,2E-04	48,129	0,0222	0,0032	8,0E-12	0,0238	0,0221	0,2812	instrument

Table S5. Single Nucleotide Polymorphisms (SNPs) used as instruments in the Mendelian randomization analyses for the effect of BMI on ovarian cancer based on the genome-wide significance threshold P = 5E-08. R^2 represents the explained variance in the exposure. SNP-specific instrument strength is given by the respective F-statistic. Some of the genetic variants were identified as outliers in the iterative approach and therefore excluded from the analyses.

SNP	CHR	POS	EA	OA	R^2	F-stat	β_{BMI}	SE_{BMI}	P_{BMI}	$\beta_{Ovarian}$	$SE_{Ovarian}$	$P_{Ovarian}$	$variant_{Ovarian}$
rs10002111	4	67815504	A	G	9,2E-05	36,430	0,0169	0,0028	9,3E-10	-0,0045	0,0159	0,7776	instrument
rs1003081	11	118913993	T	C	9,9E-05	42,843	0,0144	0,0022	6,3E-11	-0,0065	0,0135	0,6289	instrument
rs10048652	2	35449306	T	C	8,7E-05	34,452	0,0135	0,0023	3,5E-09	-0,0046	0,0133	0,7298	instrument
rs1009473	9	138777312	T	C	7,7E-05	30,489	-0,0127	0,0023	3,4E-08	-0,0185	0,0133	0,1639	instrument
rs10099330	8	143383694	A	G	8,3E-05	32,440	-0,0131	0,0023	9,9E-09	0,0008	0,0134	0,9535	instrument
rs10116186	9	80532374	A	G	1,2E-04	31,102	-0,0145	0,0026	4,1E-08	-0,0042	0,0134	0,7547	instrument
rs10118701	9	103061366	A	G	1,4E-04	53,778	-0,0176	0,0024	2,2E-13	0,0015	0,0140	0,9138	instrument
rs10124645	9	11321119	A	G	8,9E-05	34,964	0,0136	0,0023	5,0E-09	0,0034	0,0138	0,8039	instrument
rs10182181	2	25150296	A	G	6,2E-04	270,751	-0,0362	0,0022	5,0E-63	-0,0078	0,0136	0,5652	instrument
rs10237317	7	70045941	A	G	9,1E-05	39,347	-0,0138	0,0022	6,8E-10	-0,0086	0,0138	0,5316	instrument
rs10271582	7	99121950	A	G	1,2E-04	32,222	0,0193	0,0034	8,4E-09	-0,0270	0,0182	0,1387	instrument
rs10471636	5	63979856	A	G	1,3E-04	50,173	-0,0170	0,0024	4,7E-13	0,0133	0,0139	0,3402	instrument
rs1048932	11	115044850	A	C	1,6E-04	68,438	-0,0182	0,0022	2,5E-16	0,0178	0,0135	0,1857	instrument
rs10499694	7	50614173	A	G	8,8E-05	38,215	0,0136	0,0022	4,0E-10	0,0202	0,0133	0,1287	instrument

rs10513801	3	185822353	T	G	2,8E-04	120,999	0,0352	0,0032	1,3E-27	-2,3E-06	0,0199	0,9999	instrument
rs10733682	9	129460914	A	G	1,2E-04	51,578	0,0158	0,0022	1,9E-12	-0,0045	0,0133	0,7343	instrument
rs10742752	11	45438374	T	C	8,3E-05	36,000	-0,0132	0,0022	4,0E-09	0,0031	0,0137	0,8191	instrument
rs10756714	9	15885041	A	G	2,1E-04	81,784	0,0208	0,0023	1,5E-19	0,0241	0,0133	0,0699	instrument
rs1075901	17	15943910	T	C	9,9E-05	42,843	-0,0144	0,0022	4,4E-11	-0,0102	0,0134	0,4450	instrument
rs10761785	10	65318766	T	G	1,4E-04	61,124	-0,0172	0,0022	4,2E-15	0,0064	0,0132	0,6280	instrument
rs10797115	9	92191256	T	C	1,2E-04	48,393	0,0160	0,0023	4,1E-12	0,0044	0,0133	0,7387	instrument
rs10823893	10	53677313	A	G	1,6E-04	42,009	0,0175	0,0027	5,2E-11	-0,0236	0,0136	0,0820	instrument
rs10824347	10	77645229	A	T	1,1E-04	42,491	0,0176	0,0027	5,5E-11	0,0007	0,0156	0,9634	instrument
rs10909880	1	2727804	T	C	7,5E-05	31,768	-0,0124	0,0022	2,0E-08	0,0001	0,0135	0,9963	instrument
rs10920678	1	190239907	A	G	1,3E-04	54,894	0,0163	0,0022	7,5E-14	0,0154	0,0135	0,2564	instrument
rs10938397	4	45182527	A	G	4,7E-04	205,009	-0,0315	0,0022	1,7E-45	0,0072	0,0133	0,5918	instrument
rs10962550	9	16720329	C	G	1,0E-04	40,960	0,0192	0,0030	1,7E-10	0,0355	0,0180	0,0480	instrument
rs10992867	9	96461013	A	G	1,3E-04	50,628	0,0185	0,0026	8,9E-13	-0,0053	0,0152	0,7269	instrument
rs11022762	11	13335926	T	C	1,0E-04	41,173	-0,0154	0,0024	5,6E-11	-0,0130	0,0136	0,3380	instrument
rs11073383	15	95274349	A	G	1,2E-04	47,790	-0,0159	0,0023	3,3E-12	-0,0051	0,0133	0,7014	instrument
rs11165643	1	96924097	T	C	1,6E-04	67,688	0,0181	0,0022	1,7E-16	0,0090	0,0137	0,5097	instrument
rs112646560	1	39560250	T	C	1,4E-04	37,515	0,0196	0,0032	1,1E-09	-0,0428	0,0166	0,0099	outlier
rs11667280	19	30705747	C	G	7,5E-05	29,684	0,0158	0,0029	3,1E-08	-0,0135	0,0173	0,4350	instrument
rs11672660	19	46180184	T	C	3,1E-04	131,429	-0,0321	0,0028	5,9E-31	-0,0221	0,0168	0,1892	instrument
rs11677607	2	100751150	T	C	2,0E-04	79,012	-0,0240	0,0027	2,9E-19	-0,0106	0,0155	0,4931	instrument
rs1167800	7	75176196	A	G	2,0E-04	82,644	0,0200	0,0022	3,7E-19	-0,0151	0,0135	0,2637	instrument
rs11692326	2	208263279	T	C	8,3E-05	36,000	0,0156	0,0026	2,0E-09	-0,0011	0,0160	0,9468	instrument
rs11727273	4	55509748	T	C	1,6E-04	42,250	-0,0169	0,0026	1,3E-10	-0,0136	0,0136	0,3165	instrument
rs11751591	6	33794215	A	G	8,5E-05	33,423	-0,0185	0,0032	1,2E-08	-0,0040	0,0188	0,8304	instrument
rs11754747	6	141494602	T	C	7,3E-05	31,532	0,0146	0,0026	1,3E-08	-0,0085	0,0161	0,5964	instrument
rs11880870	19	18830704	A	G	1,8E-04	73,363	0,0197	0,0023	3,3E-18	-0,0048	0,0136	0,7209	instrument
rs11945861	4	65700865	A	G	7,8E-05	30,864	-0,0150	0,0027	3,1E-08	0,0197	0,0158	0,2126	instrument
rs11971098	7	26699061	A	G	8,5E-05	33,414	-0,0237	0,0041	6,5E-09	0,0117	0,0236	0,6199	instrument
rs12042959	1	243533273	A	G	9,3E-05	36,754	0,0194	0,0032	2,3E-09	-0,0159	0,0188	0,3988	instrument
rs12089815	1	91189933	A	G	1,3E-04	34,628	-0,0153	0,0026	6,0E-09	-0,0191	0,0137	0,1631	instrument
rs12101625	15	26972431	C	G	8,4E-05	33,062	0,0138	0,0024	5,5E-09	-0,0169	0,0138	0,2213	instrument

rs12140153	1	62579891	T	G	1,8E-04	48,110	-0,0326	0,0047	2,9E-12	0,0065	0,0244	0,7911	instrument
rs12150665	17	34914787	T	C	1,3E-04	57,622	0,0167	0,0022	5,9E-14	0,0065	0,0140	0,6451	instrument
rs12202969	6	98576223	A	G	1,0E-04	39,745	-0,0145	0,0023	1,9E-10	0,0086	0,0134	0,5198	instrument
rs12207125	6	154301575	A	G	1,3E-04	34,468	0,0182	0,0031	7,7E-09	-0,0231	0,0162	0,1552	instrument
rs12364470	11	134601012	T	G	1,0E-04	44,444	-0,0200	0,0030	2,5E-11	-0,0092	0,0196	0,6407	instrument
rs12429545	13	54102206	A	G	1,8E-04	77,760	0,0291	0,0033	1,1E-18	0,0190	0,0203	0,3512	instrument
rs12448257	16	3599655	A	G	7,2E-05	30,864	0,0150	0,0027	3,1E-08	0,0058	0,0166	0,7276	instrument
rs12462975	19	30272202	A	G	1,7E-04	65,934	0,0203	0,0025	2,6E-16	0,0383	0,0141	0,0065	instrument
rs12564992	1	174478100	A	G	8,0E-05	34,979	-0,0207	0,0035	2,4E-09	0,0452	0,0210	0,0310	instrument
rs12593036	15	81058652	A	G	9,6E-05	37,945	0,0154	0,0025	6,6E-10	0,0157	0,0146	0,2833	instrument
rs12612009	2	228982603	T	G	8,7E-05	34,515	-0,0141	0,0024	3,8E-09	-0,0106	0,0143	0,4595	instrument
rs12645001	4	96018337	A	G	8,7E-05	34,515	-0,0141	0,0024	4,1E-09	-0,0129	0,0139	0,3562	instrument
rs12680842	8	95582606	A	G	6,8E-05	29,340	0,0130	0,0024	3,4E-08	-0,0155	0,0141	0,2726	instrument
rs12681792	8	62054463	A	C	1,0E-04	39,820	0,0183	0,0029	2,6E-10	0,0179	0,0174	0,3040	instrument
rs12692596	2	161265910	T	C	9,3E-05	40,295	0,0146	0,0023	9,7E-11	0,0142	0,0138	0,3038	instrument
rs12692738	2	165558252	T	C	8,9E-05	38,822	-0,0162	0,0026	2,8E-10	0,0321	0,0157	0,0405	instrument
rs12714199	2	86812549	T	C	1,0E-04	40,295	-0,0146	0,0023	5,1E-10	0,0162	0,0138	0,2393	instrument
rs1285997	14	91513029	C	G	9,2E-05	36,000	-0,0150	0,0025	3,2E-09	-0,0255	0,0147	0,0832	instrument
rs12872889	13	28674628	T	C	1,7E-04	45,453	-0,0209	0,0031	1,5E-11	-0,0129	0,0158	0,4150	instrument
rs1289736	3	107950892	T	C	9,3E-05	36,523	0,0139	0,0023	1,4E-09	0,0232	0,0135	0,0848	instrument
rs12914489	15	74187937	A	G	9,1E-05	39,760	0,0227	0,0036	1,6E-10	0,0244	0,0214	0,2553	instrument
rs12949279	17	78558411	T	C	1,5E-04	66,200	0,0179	0,0022	6,1E-16	-0,0079	0,0136	0,5619	instrument
rs12981256	19	1865901	A	G	1,6E-04	62,616	0,0182	0,0023	3,9E-15	-0,0109	0,0136	0,4198	instrument
rs13002158	2	144021712	A	G	1,3E-04	51,443	-0,0208	0,0029	1,2E-12	-0,0010	0,0176	0,9547	instrument
rs13053342	22	48871624	A	G	1,0E-04	40,640	0,0153	0,0024	1,3E-10	-0,0092	0,0150	0,5409	instrument
rs13062093	3	35667057	T	G	8,3E-05	32,585	-0,0137	0,0024	8,0E-09	-0,0244	0,0137	0,0741	instrument
rs13068138	3	85809425	T	C	1,7E-04	73,197	0,0231	0,0027	3,6E-17	0,0218	0,0166	0,1884	instrument
rs13076052	3	44456573	C	G	1,2E-04	32,766	-0,0166	0,0029	1,3E-08	0,0250	0,0152	0,0989	instrument
rs13107325	4	103188709	T	C	2,4E-04	105,528	0,0452	0,0044	9,3E-25	0,0490	0,0255	0,0548	instrument
rs13110266	4	162129844	A	G	9,6E-05	41,661	-0,0142	0,0022	1,5E-10	0,0080	0,0135	0,5531	instrument
rs13174863	5	139080745	A	G	1,8E-04	75,858	-0,0270	0,0031	4,5E-18	0,0049	0,0197	0,8023	instrument
rs13209872	6	97753223	C	G	8,9E-05	35,007	-0,0142	0,0024	6,1E-09	-0,0054	0,0142	0,7026	instrument

rs13213408	6	162998049	A	T	9,0E-05	35,637	0,0197	0,0033	4,3E-09	0,0206	0,0196	0,2920	instrument
rs1362910	8	30856464	A	G	8,2E-05	33,943	0,0134	0,0023	3,0E-09	0,0091	0,0135	0,4999	instrument
rs1403846	4	119101723	T	C	7,2E-05	31,206	0,0162	0,0029	1,6E-08	0,0029	0,0174	0,8683	instrument
rs1409818	20	21381121	T	C	1,2E-04	46,387	0,0252	0,0037	1,6E-11	-0,0418	0,0221	0,0584	instrument
rs141729694	5	87999371	T	C	2,2E-04	57,284	0,0386	0,0051	2,5E-14	0,0161	0,0248	0,5170	instrument
rs1417665	6	104816944	T	C	1,2E-04	47,088	0,0199	0,0029	5,7E-12	-0,0300	0,0171	0,0794	instrument
rs1421598	2	50264710	A	C	9,7E-05	38,360	-0,0192	0,0031	6,8E-10	-0,0094	0,0180	0,6012	instrument
rs1436348	3	104612668	A	G	8,6E-05	33,943	-0,0134	0,0023	6,3E-09	-0,0178	0,0135	0,1876	instrument
rs1441264	13	79580919	A	G	1,3E-04	55,276	0,0171	0,0023	5,2E-14	0,0134	0,0139	0,3362	instrument
rs1452075	3	62481063	T	C	9,4E-05	40,449	0,0159	0,0025	1,5E-10	0,0280	0,0151	0,0641	instrument
rs1491333	4	172186288	T	G	8,1E-05	31,966	-0,0147	0,0026	9,9E-09	0,0099	0,0156	0,5257	instrument
rs1492014	3	94071481	T	C	1,0E-04	39,745	-0,0145	0,0023	2,8E-10	-0,0035	0,0134	0,7933	instrument
rs1518159	18	40760146	T	C	1,3E-04	52,841	-0,0189	0,0026	6,7E-13	0,0075	0,0152	0,6223	instrument
rs1561589	10	126695673	A	G	1,1E-04	47,191	0,0158	0,0023	4,2E-12	-0,0092	0,0137	0,5007	instrument
rs1569979	14	29681294	A	G	1,8E-04	72,565	0,0230	0,0027	1,6E-17	0,0216	0,0157	0,1702	instrument
rs1579557	6	40371918	T	C	1,8E-04	71,910	0,0212	0,0025	3,1E-17	0,0275	0,0149	0,0654	instrument
rs158186	5	107440856	A	G	2,2E-04	85,711	-0,0287	0,0031	3,2E-20	-0,0139	0,0175	0,4269	instrument
rs159032	5	94206202	T	C	7,8E-05	30,454	0,0149	0,0027	4,5E-08	0,0144	0,0153	0,3462	instrument
rs1681743	10	118565501	T	C	7,5E-05	29,793	-0,0131	0,0024	3,7E-08	-0,0173	0,0136	0,2011	instrument
rs16851483	3	141275436	T	G	1,6E-04	62,272	0,0363	0,0046	2,3E-15	0,0061	0,0268	0,8194	instrument
rs16912921	9	28413461	A	C	2,7E-04	117,204	0,0249	0,0023	1,6E-26	0,0305	0,0142	0,0319	instrument
rs16975921	18	39914870	A	T	1,1E-04	42,510	-0,0163	0,0025	3,9E-11	-0,0139	0,0141	0,3232	instrument
rs16989232	20	39291784	A	G	7,1E-05	30,972	0,0128	0,0023	1,5E-08	0,0039	0,0140	0,7793	instrument
rs17014375	1	209543560	T	G	7,9E-05	31,228	-0,0190	0,0034	1,5E-08	-0,0010	0,0194	0,9578	instrument
rs17066856	18	58049656	T	C	2,7E-04	115,707	0,0398	0,0037	1,4E-27	0,0368	0,0229	0,1088	instrument
rs17405819	8	76806584	T	C	1,9E-04	82,507	0,0218	0,0024	7,8E-20	0,0029	0,0143	0,8383	instrument
rs1750307	1	156488420	A	T	8,3E-05	32,585	0,0137	0,0024	8,2E-09	0,0255	0,0138	0,0639	instrument
rs17522122	14	33302882	T	G	1,3E-04	57,622	0,0167	0,0022	6,5E-14	0,0013	0,0135	0,9221	instrument
rs17619973	3	114417675	A	G	7,4E-05	31,673	0,0242	0,0043	1,3E-08	-0,0392	0,0268	0,1437	instrument
rs17724992	19	18454825	A	G	1,1E-04	46,240	0,0170	0,0025	6,7E-12	-0,0008	0,0150	0,9596	instrument
rs17751061	19	19413092	T	C	8,2E-05	31,993	-0,0181	0,0032	1,3E-08	0,0457	0,0180	0,0113	outlier
rs17767765	16	24539793	T	C	7,7E-05	30,489	-0,0127	0,0023	2,6E-08	0,0124	0,0138	0,3677	instrument

rs17806224	20	51065854	A	G	2,3E-04	92,801	-0,0289	0,0030	3,5E-22	0,0139	0,0176	0,4274	instrument
rs1799923	3	42306294	A	G	1,2E-04	48,203	-0,0243	0,0035	5,1E-12	0,0170	0,0220	0,4398	instrument
rs1801282	3	12393125	C	G	7,0E-05	30,417	-0,0182	0,0033	4,7E-08	0,0072	0,0200	0,7188	instrument
rs1808579	18	21104888	T	C	1,9E-04	81,000	-0,0198	0,0022	9,4E-20	-0,0113	0,0133	0,3941	instrument
rs1808629	8	73435964	A	G	2,5E-04	65,147	-0,0226	0,0028	5,8E-16	0,0092	0,0145	0,5252	instrument
rs1812587	5	62991802	T	G	1,3E-04	34,177	0,0152	0,0026	7,0E-09	-0,0124	0,0134	0,3512	instrument
rs185350	19	34306816	T	C	7,8E-05	33,851	0,0128	0,0022	7,1E-09	-0,0208	0,0133	0,1163	instrument
rs1861412	2	58893065	A	G	2,2E-04	87,381	-0,0215	0,0023	9,1E-21	-0,0187	0,0133	0,1600	instrument
rs1884389	20	1410582	T	C	9,4E-05	37,051	-0,0140	0,0023	1,2E-09	0,0060	0,0133	0,6526	instrument
rs1884897	20	6612832	A	G	1,2E-04	53,990	-0,0169	0,0023	9,1E-14	0,0076	0,0139	0,5827	instrument
rs1916801	3	61187046	A	T	1,4E-04	59,710	0,0170	0,0022	1,8E-14	-0,0196	0,0136	0,1495	instrument
rs1927790	13	96922191	T	C	8,7E-05	37,655	-0,0135	0,0022	1,3E-09	-0,0205	0,0135	0,1300	instrument
rs1928295	9	120378483	T	C	1,2E-04	52,892	0,0160	0,0022	2,8E-13	0,0020	0,0133	0,8794	instrument
rs1949197	3	86273850	A	G	9,2E-05	36,523	0,0139	0,0023	3,0E-09	0,0115	0,0136	0,3985	instrument
rs197374	1	112289983	T	C	1,4E-04	55,276	0,0171	0,0023	2,8E-13	0,0118	0,0137	0,3887	instrument
rs1984559	2	135678888	A	G	7,9E-05	29,521	0,0163	0,0030	3,3E-08	0,0335	0,0161	0,0372	instrument
rs198665	6	143154886	A	C	7,8E-05	33,943	0,0134	0,0023	4,4E-09	-0,0126	0,0140	0,3685	instrument
rs2012502	16	81728081	A	C	1,0E-04	44,251	0,0153	0,0023	3,0E-11	0,0098	0,0139	0,4798	instrument
rs2045293	7	114350102	T	C	1,7E-04	67,525	0,0189	0,0023	4,1E-16	-0,0118	0,0135	0,3823	instrument
rs2053682	5	170599327	A	C	1,4E-04	53,582	0,0183	0,0025	1,9E-13	-0,0091	0,0142	0,5234	instrument
rs209416	6	83534729	A	T	1,2E-04	47,840	0,0166	0,0024	8,7E-12	0,0130	0,0143	0,3624	instrument
rs2112347	5	75015242	T	G	4,2E-04	180,493	0,0309	0,0023	2,1E-42	-0,0237	0,0137	0,0837	instrument
rs214249	16	348687	T	G	1,2E-04	48,418	0,0167	0,0024	1,4E-12	0,0024	0,0134	0,8563	instrument
rs2161097	5	103945178	T	C	8,0E-05	31,457	0,0129	0,0023	2,3E-08	-0,0008	0,0133	0,9517	instrument
rs2178899	6	31606756	A	T	1,3E-04	56,477	0,0248	0,0033	7,2E-14	-0,0142	0,0204	0,4858	instrument
rs2185027	6	153381622	A	C	9,5E-05	41,173	-0,0154	0,0024	1,1E-10	-0,0063	0,0144	0,6617	instrument
rs2228213	6	12124855	A	G	9,5E-05	41,406	-0,0148	0,0023	9,2E-11	-0,0074	0,0138	0,5928	instrument
rs2267812	7	74138121	A	C	9,2E-05	35,177	-0,0172	0,0029	1,7E-09	-0,0290	0,0161	0,0716	instrument
rs2267958	9	131015279	A	G	1,2E-04	45,002	-0,0161	0,0024	1,9E-11	-0,0210	0,0141	0,1351	instrument
rs2271046	4	52752812	A	T	7,6E-05	30,030	-0,0137	0,0025	2,4E-08	-0,0186	0,0148	0,2081	instrument
rs2289292	16	30097630	T	C	1,5E-04	57,760	-0,0190	0,0025	1,1E-14	-0,0050	0,0147	0,7337	instrument
rs2289705	7	121983843	A	G	7,7E-05	33,284	-0,0150	0,0026	1,5E-08	-0,0207	0,0164	0,2070	instrument

rs2299383	7	103418846	T	C	1,6E-04	68,438	0,0182	0,0022	2,7E-16	-0,0164	0,0135	0,2239	instrument
rs2357760	6	120213880	A	G	1,1E-04	47,790	0,0159	0,0023	1,1E-11	-0,0206	0,0141	0,1432	instrument
rs2465043	6	51180765	A	G	8,9E-05	35,007	-0,0142	0,0024	3,2E-09	-0,0073	0,0139	0,6013	instrument
rs2470520	3	15762920	T	C	7,6E-05	30,011	-0,0126	0,0023	4,2E-08	0,0007	0,0134	0,9565	instrument
rs247975	3	173107443	T	C	1,6E-04	62,616	-0,0182	0,0023	3,6E-15	-0,0236	0,0140	0,0905	instrument
rs2479958	13	111984244	A	G	8,1E-05	31,640	0,0135	0,0024	1,4E-08	0,0020	0,0137	0,8841	instrument
rs2528531	7	93236510	A	C	9,3E-05	36,502	-0,0145	0,0024	1,3E-09	-0,0029	0,0138	0,8318	instrument
rs2568958	1	72765116	A	G	2,5E-04	106,464	0,0227	0,0022	1,3E-24	0,0149	0,0137	0,2747	instrument
rs2605603	11	93221105	A	G	8,3E-05	36,000	-0,0132	0,0022	1,4E-09	0,0257	0,0133	0,0528	instrument
rs2619976	17	71754545	T	C	9,4E-05	37,007	0,0146	0,0024	6,2E-10	0,0022	0,0141	0,8750	instrument
rs2721965	8	116662038	A	C	1,5E-04	60,062	0,0186	0,0024	2,1E-14	0,0397	0,0142	0,0051	outlier
rs273504	19	18215247	A	G	1,1E-04	43,102	-0,0151	0,0023	1,3E-10	0,0149	0,0135	0,2674	instrument
rs2754079	14	25886140	A	G	1,2E-04	46,004	-0,0156	0,0023	2,3E-11	-0,0002	0,0137	0,9906	instrument
rs2814992	6	34617144	A	G	3,6E-04	155,706	-0,0287	0,0023	1,2E-35	-0,0131	0,0139	0,3454	instrument
rs2820295	1	201800868	A	G	2,0E-04	79,507	0,0214	0,0024	8,0E-19	-0,0052	0,0141	0,7123	instrument
rs2836754	21	40291740	T	C	1,1E-04	45,870	-0,0149	0,0022	3,7E-11	0,0019	0,0136	0,8863	instrument
rs28489620	22	41804716	A	G	1,5E-04	38,955	-0,0181	0,0029	6,9E-10	-0,0164	0,0147	0,2625	instrument
rs2861685	2	67837553	T	C	1,8E-04	47,929	0,0180	0,0026	1,1E-11	-0,0262	0,0134	0,0511	instrument
rs2862961	11	43656535	A	G	2,3E-04	91,393	-0,0239	0,0025	5,3E-22	-0,0264	0,0142	0,0640	instrument
rs2959592	8	17002725	T	C	8,7E-05	36,862	0,0170	0,0028	6,3E-10	-0,0172	0,0173	0,3196	instrument
rs2968864	7	150622162	T	C	9,2E-05	36,446	0,0163	0,0027	9,3E-10	-0,0124	0,0154	0,4206	instrument
rs2984618	1	47690438	T	G	1,3E-04	57,622	0,0167	0,0022	2,2E-14	-0,0013	0,0135	0,9256	instrument
rs329651	11	133767622	T	G	9,7E-05	41,787	0,0181	0,0028	2,0E-10	0,0005	0,0173	0,9779	instrument
rs337637	4	38604470	A	G	6,8E-05	29,537	-0,0125	0,0023	3,0E-08	0,0038	0,0140	0,7835	instrument
rs34517439	1	78450517	A	C	3,4E-04	90,481	0,0390	0,0041	2,0E-21	-0,0016	0,0216	0,9426	instrument
rs34811474	4	25408838	A	G	4,0E-04	104,566	-0,0317	0,0031	5,2E-24	-0,0124	0,0184	0,4983	instrument
rs34930419	10	33987329	T	C	1,4E-04	36,316	-0,0458	0,0076	1,4E-09	0,0448	0,0399	0,2618	instrument
rs35107973	4	143125948	T	C	1,2E-04	32,573	0,0234	0,0041	7,7E-09	0,0116	0,0213	0,5855	instrument
rs355754	3	154024165	T	C	9,2E-05	38,117	-0,0142	0,0023	4,3E-10	-0,0040	0,0136	0,7676	instrument
rs35722922	2	47002226	A	G	1,8E-04	48,482	0,0188	0,0027	3,6E-12	-0,0075	0,0138	0,5864	instrument
rs3803286	14	103246470	A	G	1,3E-04	51,361	0,0172	0,0024	8,3E-13	0,0121	0,0140	0,3864	instrument
rs3806114	6	20482335	A	G	8,0E-05	34,515	-0,0141	0,0024	4,5E-09	-0,0250	0,0144	0,0825	instrument

rs3826408	17	7101292	T	C	7,0E-05	30,250	0,0121	0,0022	4,5E-08	-0,0034	0,0134	0,7981	instrument
rs3829849	9	129390800	T	C	8,0E-05	34,964	0,0136	0,0023	1,9E-09	-0,0173	0,0138	0,2116	instrument
rs3977755	10	104420210	T	C	8,0E-05	32,718	-0,0143	0,0025	8,5E-09	-0,0246	0,0148	0,0967	instrument
rs4055791	13	59266053	T	C	1,5E-04	39,643	-0,0170	0,0027	1,8E-10	0,0032	0,0136	0,8136	instrument
rs4148155	4	89054667	A	G	7,7E-05	33,640	0,0203	0,0035	6,2E-09	0,0114	0,0213	0,5929	instrument
rs4240673	8	10787612	T	C	1,5E-04	64,000	0,0176	0,0022	1,0E-15	0,0005	0,0174	0,9790	instrument
rs427943	21	46570896	A	C	1,5E-04	59,223	-0,0177	0,0023	1,4E-14	-0,0202	0,0136	0,1369	instrument
rs429358	19	45411941	T	C	2,2E-04	58,777	0,0276	0,0036	3,2E-14	-0,0015	0,0193	0,9395	instrument
rs4295917	1	32173360	A	G	8,2E-05	31,640	0,0135	0,0024	1,6E-08	-0,0049	0,0143	0,7303	instrument
rs4307239	7	24354300	A	G	9,5E-05	37,582	-0,0141	0,0023	1,3E-09	-0,0117	0,0135	0,3892	instrument
rs4320543	8	34422967	A	G	8,0E-05	31,457	-0,0129	0,0023	2,9E-08	0,0219	0,0139	0,1147	instrument
rs4421623	1	98316285	T	G	1,2E-04	46,441	0,0184	0,0027	1,1E-11	-0,0081	0,0159	0,6097	instrument
rs4459316	11	130756176	T	C	1,1E-04	44,832	-0,0154	0,0023	2,3E-11	0,0030	0,0135	0,8225	instrument
rs4474229	1	195037001	A	G	1,6E-04	41,055	-0,0173	0,0027	1,6E-10	0,0052	0,0136	0,7022	instrument
rs4482463	2	205375909	A	C	2,0E-04	75,524	-0,0365	0,0042	5,2E-18	0,0584	0,0253	0,0210	instrument
rs4569924	5	153540025	T	C	1,2E-04	51,578	0,0158	0,0022	7,4E-13	0,0313	0,0134	0,0195	instrument
rs4586287	13	99245334	C	G	7,7E-05	30,470	0,0138	0,0025	2,9E-08	-0,0131	0,0145	0,3650	instrument
rs4673553	2	211608379	T	G	1,1E-04	44,251	-0,0153	0,0023	2,6E-11	0,0028	0,0135	0,8330	instrument
rs4677812	3	194863794	A	C	8,4E-05	33,177	-0,0144	0,0025	9,4E-09	0,0037	0,0148	0,8047	instrument
rs4776982	15	68114974	A	G	3,3E-04	143,078	0,0311	0,0026	4,2E-33	0,0058	0,0159	0,7159	instrument
rs4783718	16	69547741	T	C	1,8E-04	78,669	0,0204	0,0023	1,8E-19	0,0226	0,0140	0,1063	instrument
rs4790292	17	1824305	A	C	1,7E-04	63,516	-0,0263	0,0033	1,7E-15	0,0340	0,0197	0,0838	instrument
rs4834272	4	113313986	T	C	8,9E-05	38,656	-0,0143	0,0023	8,2E-10	-0,0236	0,0144	0,1021	instrument
rs4858223	3	20581149	T	C	1,1E-04	41,473	-0,0161	0,0025	8,3E-11	0,0036	0,0143	0,8044	instrument
rs4864201	4	130731284	T	C	7,5E-05	32,440	0,0131	0,0023	6,3E-09	-0,0024	0,0138	0,8593	instrument
rs4880341	10	133992689	T	C	9,8E-05	38,656	-0,0143	0,0023	4,5E-10	-0,0042	0,0134	0,7509	instrument
rs4889606	16	31011183	A	G	3,1E-04	135,773	0,0268	0,0023	1,3E-32	0,0139	0,0137	0,3132	instrument
rs4929923	11	8639200	T	C	2,1E-04	92,327	-0,0221	0,0023	2,5E-22	-0,0270	0,0137	0,0485	instrument
rs4936175	11	132641959	T	C	1,2E-04	47,191	-0,0158	0,0023	6,7E-12	0,0057	0,0134	0,6682	instrument
rs4969387	17	79081724	C	G	8,4E-05	32,532	-0,0154	0,0027	6,9E-09	0,0020	0,0153	0,8948	instrument
rs4986044	17	21261560	T	C	1,4E-04	61,836	-0,0173	0,0022	2,2E-15	-0,0099	0,0132	0,4556	instrument
rs543874	1	177889480	A	G	1,0E-03	455,109	-0,0576	0,0027	8,7E-100	-0,0019	0,0169	0,9110	instrument

rs56161855	17	46288649	A	T	1,7E-04	44,787	-0,0261	0,0039	1,5E-11	0,0246	0,0193	0,2020	instrument
rs563132	9	37245476	A	T	1,4E-04	56,250	0,0180	0,0024	2,0E-14	-0,0106	0,0136	0,4382	instrument
rs563296	10	99772404	A	G	1,4E-04	53,168	0,0175	0,0024	8,8E-14	-0,0117	0,0136	0,3873	instrument
rs57221746	15	99230035	A	G	1,2E-04	30,594	-0,0177	0,0032	2,1E-08	-0,0023	0,0159	0,8856	instrument
rs57800857	4	140863365	A	C	1,8E-04	47,456	0,0186	0,0027	1,1E-11	0,0161	0,0138	0,2451	instrument
rs6010784	20	61540319	T	C	7,6E-05	32,802	0,0126	0,0022	1,1E-08	0,0082	0,0132	0,5353	instrument
rs6050446	20	25195509	A	G	8,0E-05	33,785	-0,0372	0,0064	6,2E-09	-0,0427	0,0411	0,2980	instrument
rs6142096	20	32686658	A	G	8,6E-05	33,943	0,0134	0,0023	4,2E-09	0,0103	0,0132	0,4340	instrument
rs61813324	1	156049877	T	C	2,2E-04	58,385	0,0298	0,0039	3,6E-14	0,0294	0,0213	0,1665	instrument
rs62007782	15	78029797	A	G	1,4E-04	35,601	-0,0179	0,0030	1,9E-09	-0,0052	0,0152	0,7303	instrument
rs62106258	2	417167	T	C	9,7E-04	254,452	0,0989	0,0062	8,7E-58	0,0406	0,0360	0,2584	instrument
rs62412826	6	61881167	T	C	1,3E-04	33,518	0,0330	0,0057	8,0E-09	-0,0589	0,0294	0,0452	instrument
rs625652	11	65641931	A	T	2,0E-04	52,160	-0,0195	0,0027	9,1E-13	0,0089	0,0139	0,5199	instrument
rs6265	11	27679916	T	C	4,0E-04	172,734	-0,0368	0,0028	8,0E-40	0,0142	0,0167	0,3959	instrument
rs6446187	3	49907111	A	C	4,3E-04	114,325	0,0278	0,0026	1,2E-26	0,0016	0,0132	0,9064	instrument
rs6493498	15	51754451	T	C	1,3E-04	54,894	0,0163	0,0022	1,5E-13	0,0046	0,0134	0,7297	instrument
rs6548237	2	621461	A	C	9,1E-04	394,500	-0,0576	0,0029	4,4E-89	-0,0056	0,0220	0,8009	instrument
rs6556810	5	164585949	T	C	7,4E-05	29,066	-0,0124	0,0023	5,0E-08	-0,0028	0,0135	0,8385	instrument
rs6561937	13	58257667	A	T	1,4E-04	53,236	-0,0197	0,0027	6,0E-13	0,0126	0,0160	0,4303	instrument
rs6567160	18	57829135	T	C	1,1E-03	475,573	-0,0567	0,0026	8,7E-108	0,0057	0,0156	0,7163	instrument
rs657452	1	49589847	A	G	2,0E-04	85,142	0,0203	0,0022	1,1E-19	-0,0095	0,0135	0,4809	instrument
rs6585198	10	114762237	A	G	1,1E-04	49,000	-0,0154	0,0022	1,2E-12	-0,0246	0,0132	0,0626	instrument
rs6669189	1	75011358	T	C	2,1E-04	91,115	0,0210	0,0022	4,7E-21	0,0094	0,0134	0,4865	instrument
rs6720868	2	230663576	T	C	1,3E-04	49,585	0,0169	0,0024	4,6E-12	0,0024	0,0140	0,8616	instrument
rs6728037	2	59297346	A	G	2,1E-04	81,000	0,0207	0,0023	6,5E-19	0,0241	0,0136	0,0769	instrument
rs6739199	2	105452326	T	C	1,0E-04	40,449	0,0159	0,0025	3,2E-10	0,0225	0,0153	0,1409	instrument
rs6870983	5	87697533	T	C	1,5E-04	66,485	-0,0212	0,0026	9,6E-16	0,0060	0,0158	0,7052	instrument
rs6890310	5	27193573	A	G	9,6E-05	37,945	-0,0154	0,0025	1,4E-09	0,0217	0,0150	0,1490	instrument
rs6973700	7	1856669	A	G	1,4E-04	36,376	0,0193	0,0032	1,8E-09	0,0206	0,0162	0,2050	instrument
rs7024334	9	109072075	T	G	8,7E-05	37,398	0,0159	0,0026	1,6E-09	0,0030	0,0158	0,8482	instrument
rs7084454	10	21821274	A	G	2,1E-04	81,000	0,0225	0,0025	7,4E-20	0,0883	0,0140	2,4E-10	outlier
rs709400	14	104149475	A	G	1,5E-04	64,000	0,0184	0,0023	4,4E-16	-0,0013	0,0136	0,9240	instrument

rs7105462	11	112912048	A	G	9,9E-05	39,198	0,0144	0,0023	4,4E-10	-0,0117	0,0135	0,3848	instrument
rs7107409	11	76492195	T	C	1,2E-04	30,941	-0,0178	0,0032	2,1E-08	0,0160	0,0159	0,3143	instrument
rs7124681	11	47529947	A	C	2,9E-04	126,051	0,0247	0,0022	9,3E-29	0,0054	0,0135	0,6876	instrument
rs7141420	14	79899454	T	C	2,1E-04	91,115	0,0210	0,0022	7,8E-22	-0,0092	0,0137	0,5013	instrument
rs7211966	17	54374611	T	C	8,9E-05	34,515	-0,0470	0,0080	4,9E-09	0,0856	0,0568	0,1316	instrument
rs7239114	18	45921214	A	G	1,2E-04	49,638	0,0155	0,0022	3,9E-12	-0,0122	0,0136	0,3721	instrument
rs7250833	19	33937277	T	C	8,9E-05	38,543	0,0149	0,0024	7,2E-10	0,0009	0,0147	0,9514	instrument
rs73124396	7	71579606	T	C	1,2E-04	32,111	0,0187	0,0033	1,1E-08	-0,0065	0,0163	0,6903	instrument
rs73213484	4	28489339	A	T	1,7E-04	45,653	0,0250	0,0037	2,3E-11	-0,0275	0,0185	0,1367	instrument
rs7335249	13	109962579	T	C	8,7E-05	34,452	0,0135	0,0023	4,9E-09	0,0060	0,0136	0,6598	instrument
rs7395632	11	958789	T	C	7,4E-05	29,340	-0,0130	0,0024	3,9E-08	-0,0092	0,0138	0,5032	instrument
rs7428670	3	131579810	C	G	1,5E-04	60,361	0,0202	0,0026	3,1E-15	0,0142	0,0150	0,3425	instrument
rs7498044	15	92573639	A	G	1,2E-04	31,640	-0,0180	0,0032	2,7E-08	-0,0158	0,0169	0,3500	instrument
rs7498665	16	28883241	A	G	3,3E-04	143,999	-0,0264	0,0022	1,9E-32	-0,0057	0,0136	0,6731	instrument
rs750090	4	152931436	T	C	1,1E-04	42,250	0,0156	0,0024	7,4E-11	0,0113	0,0142	0,4250	instrument
rs7550711	1	110082886	T	C	2,1E-04	88,858	0,0641	0,0068	3,4E-21	0,1205	0,0382	0,0016	outlier
rs7571496	2	6169351	A	G	7,9E-05	31,102	0,0145	0,0026	1,4E-08	-0,0012	0,0152	0,9362	instrument
rs7588437	2	181575281	A	G	1,3E-04	58,555	-0,0176	0,0023	8,4E-15	-0,0146	0,0137	0,2883	instrument
rs7594237	2	198187164	A	T	8,3E-05	32,718	-0,0143	0,0025	1,8E-08	-0,0109	0,0152	0,4722	instrument
rs7601870	2	15384392	A	G	8,3E-05	32,937	-0,0132	0,0023	1,2E-08	-0,0057	0,0135	0,6730	instrument
rs7601895	2	55281901	C	G	1,1E-04	41,473	0,0161	0,0025	9,6E-11	0,0051	0,0148	0,7309	instrument
rs7621025	3	136272246	T	C	1,5E-04	64,000	-0,0200	0,0025	1,6E-15	0,0124	0,0152	0,4144	instrument
rs7647242	3	82642734	T	C	1,0E-04	39,745	0,0145	0,0023	3,9E-10	0,0131	0,0136	0,3383	instrument
rs768023	6	108876002	A	G	1,6E-04	70,712	0,0185	0,0022	1,4E-16	-0,0053	0,0136	0,6968	instrument
rs76900466	2	145057760	T	G	1,2E-04	30,654	0,0227	0,0041	4,5E-08	-0,0061	0,0253	0,8101	instrument
rs77432547	13	86494817	A	G	1,4E-04	36,415	-0,0175	0,0029	2,3E-09	0,0090	0,0149	0,5435	instrument
rs7788008	7	112972483	A	G	1,4E-04	55,276	-0,0171	0,0023	1,1E-13	-0,0112	0,0133	0,3987	instrument
rs7805441	7	78121458	T	C	1,0E-04	40,295	0,0146	0,0023	2,0E-10	-0,0128	0,0132	0,3325	instrument
rs782971	5	43124688	A	G	1,1E-04	47,334	-0,0172	0,0025	4,9E-12	-0,0086	0,0155	0,5789	instrument
rs786420	2	44719893	T	C	8,4E-05	32,956	0,0155	0,0027	8,8E-09	-0,0054	0,0154	0,7238	instrument
rs78886584	1	16859325	A	G	1,3E-04	33,284	-0,0150	0,0026	1,3E-08	0,0140	0,0151	0,3516	instrument
rs7899106	10	87410904	A	G	1,3E-04	56,250	-0,0375	0,0050	7,6E-14	-0,0170	0,0317	0,5911	instrument

rs79113395	1	1590521	A	G	1,9E-04	49,000	-0,0210	0,0030	1,5E-12	0,0144	0,0193	0,4576	instrument
rs8024806	15	53473990	T	C	8,5E-05	33,492	0,0272	0,0047	9,8E-09	0,0229	0,0278	0,4109	instrument
rs8033510	15	61445514	T	C	8,3E-05	32,585	0,0137	0,0024	1,1E-08	-0,0021	0,0137	0,8787	instrument
rs8038522	15	46585105	T	G	9,4E-05	37,051	-0,0140	0,0023	1,3E-09	0,0201	0,0134	0,1324	instrument
rs8075273	17	61728881	A	C	1,0E-04	44,444	-0,0160	0,0024	5,6E-11	0,0231	0,0148	0,1183	instrument
rs811054	16	72251132	T	C	1,2E-04	54,223	0,0162	0,0022	3,2E-13	0,0115	0,0134	0,3906	instrument
rs836525	7	6465909	T	C	8,2E-05	32,233	0,0176	0,0031	9,8E-09	0,0152	0,0181	0,4013	instrument
rs879620	16	4015729	T	C	2,9E-04	115,328	0,0247	0,0023	5,4E-26	-0,0027	0,0138	0,8430	instrument
rs893006	11	64365796	A	C	1,2E-04	51,960	0,0173	0,0024	4,0E-13	0,0164	0,0146	0,2623	instrument
rs895330	19	4060707	C	G	9,9E-05	38,955	0,0181	0,0029	6,6E-10	-0,0154	0,0174	0,3775	instrument
rs915814	21	46493003	A	G	1,3E-04	34,810	-0,0177	0,0030	5,9E-09	-0,0065	0,0157	0,6807	instrument
rs925018	3	62713143	C	G	8,8E-05	38,117	-0,0142	0,0023	9,8E-10	-0,0162	0,0142	0,2540	instrument
rs9304665	19	47602577	A	T	1,4E-04	57,086	0,0204	0,0027	1,8E-14	0,0007	0,0156	0,9638	instrument
rs9367369	6	13189941	A	G	1,4E-04	35,587	0,0173	0,0029	2,3E-09	0,0184	0,0145	0,2044	instrument
rs942066	14	94031914	A	G	2,1E-04	55,972	-0,0202	0,0027	1,0E-13	0,0036	0,0138	0,7944	instrument
rs943005	6	50865820	T	C	4,3E-04	188,351	0,0398	0,0029	2,0E-43	0,0093	0,0174	0,5941	instrument
rs9531786	13	85983968	C	G	8,4E-05	33,062	-0,0138	0,0024	8,4E-09	-0,0072	0,0139	0,6039	instrument
rs9571687	13	67472713	A	C	9,2E-05	36,502	-0,0145	0,0024	3,2E-09	0,0017	0,0142	0,9019	instrument
rs9579775	13	20616557	A	C	1,5E-04	39,690	-0,0252	0,0040	3,7E-10	0,0488	0,0238	0,0403	instrument
rs9595630	13	33065443	T	G	9,2E-05	39,745	0,0145	0,0023	2,0E-10	-0,0036	0,0137	0,7917	instrument
rs973345	9	29713169	T	C	9,7E-05	38,117	0,0142	0,0023	1,1E-09	0,0056	0,0135	0,6785	instrument
rs9906944	17	47091420	T	C	1,6E-04	62,673	-0,0190	0,0024	7,3E-15	-0,0167	0,0143	0,2430	instrument
rs9926784	16	19941968	T	C	1,9E-04	81,000	0,0252	0,0028	6,0E-20	0,0277	0,0171	0,1056	instrument
rs9937053	16	53799507	A	G	2,2E-03	941,370	0,0675	0,0022	1,0E-200	0,0190	0,0133	0,1516	instrument
rs994596	4	18459828	T	C	9,1E-05	36,000	0,0150	0,0025	1,0E-09	-0,0267	0,0142	0,0591	instrument
rs9955276	18	1839339	T	C	1,2E-04	48,129	0,0222	0,0032	8,0E-12	-0,0095	0,0189	0,6143	instrument

Table S6. Single Nucleotide Polymorphisms (SNPs) used as instruments in the Mendelian randomization analyses for the effect of BMI on ER- breast cancer based on the genome-wide significance threshold P = 5E-08. R^2 represents the explained variance in the exposure. SNP-specific instrument strength is given by the respective F-statistic. Some of the genetic variants were identified as outliers in the iterative approach and therefore excluded from the analyses.

SNP	CHR	POS	EA	OA	R^2	F-stat	β_{BMI}	SE_{BMI}	P_{BMI}	β_{ER-}	SE_{ER-}	P_{ER-}	$variant_{ER-}$
rs10002111	4	67815504	A	G	9,2E-05	36,430	0,0169	0,0028	9,3E-10	-0,0098	0,0135	0,4701	instrument
rs1003081	11	118913993	T	C	9,9E-05	42,843	0,0144	0,0022	6,3E-11	-0,0078	0,0117	0,5083	instrument
rs10048652	2	35449306	T	C	8,7E-05	34,452	0,0135	0,0023	3,5E-09	-0,0119	0,0114	0,2991	instrument
rs1009473	9	138777312	T	C	7,7E-05	30,489	-0,0127	0,0023	3,4E-08	0,0231	0,0114	0,0424	instrument
rs10099330	8	143383694	A	G	8,3E-05	32,440	-0,0131	0,0023	9,9E-09	-0,0009	0,0114	0,9386	instrument
rs10116186	9	80532374	A	G	1,2E-04	31,102	-0,0145	0,0026	4,1E-08	0,0004	0,0114	0,9726	instrument
rs10118701	9	103061366	A	G	1,4E-04	53,778	-0,0176	0,0024	2,2E-13	0,0169	0,0120	0,1591	instrument
rs10124645	9	11321119	A	G	8,9E-05	34,964	0,0136	0,0023	5,0E-09	0,0071	0,0120	0,5542	instrument
rs10182181	2	25150296	A	G	6,2E-04	270,751	-0,0362	0,0022	5,0E-63	0,0538	0,0119	6,1E-06	outlier
rs10237317	7	70045941	A	G	9,1E-05	39,347	-0,0138	0,0022	6,8E-10	0,0083	0,0117	0,4768	instrument
rs10271582	7	99121950	A	G	1,2E-04	32,222	0,0193	0,0034	8,4E-09	0,0084	0,0156	0,5916	instrument
rs10471636	5	63979856	A	G	1,3E-04	50,173	-0,0170	0,0024	4,7E-13	0,0070	0,0121	0,5640	instrument
rs1048932	11	115044850	A	C	1,6E-04	68,438	-0,0182	0,0022	2,5E-16	-0,0027	0,0116	0,8156	instrument
rs10499694	7	50614173	A	G	8,8E-05	38,215	0,0136	0,0022	4,0E-10	0,0005	0,0115	0,9680	instrument
rs10513801	3	185822353	T	G	2,8E-04	120,999	0,0352	0,0032	1,3E-27	0,0034	0,0172	0,8415	instrument
rs10733682	9	129460914	A	G	1,2E-04	51,578	0,0158	0,0022	1,9E-12	-0,0193	0,0115	0,0947	instrument
rs10742752	11	45438374	T	C	8,3E-05	36,000	-0,0132	0,0022	4,0E-09	0,0079	0,0119	0,5079	instrument
rs10756714	9	15885041	A	G	2,1E-04	81,784	0,0208	0,0023	1,5E-19	0,0127	0,0114	0,2669	instrument
rs1075901	17	15943910	T	C	9,9E-05	42,843	-0,0144	0,0022	4,4E-11	0,0085	0,0114	0,4538	instrument
rs10761785	10	65318766	T	G	1,4E-04	61,124	-0,0172	0,0022	4,2E-15	-0,0048	0,0112	0,6695	instrument
rs10797115	9	92191256	T	C	1,2E-04	48,393	0,0160	0,0023	4,1E-12	-0,0335	0,0114	0,0032	outlier
rs10823893	10	53677313	A	G	1,6E-04	42,009	0,0175	0,0027	5,2E-11	-0,0080	0,0117	0,4926	instrument
rs10824347	10	77645229	A	T	1,1E-04	42,491	0,0176	0,0027	5,5E-11	-0,0224	0,0134	0,0955	instrument
rs10909880	1	2727804	T	C	7,5E-05	31,768	-0,0124	0,0022	2,0E-08	-0,0083	0,0114	0,4665	instrument
rs10920678	1	190239907	A	G	1,3E-04	54,894	0,0163	0,0022	7,5E-14	-0,0080	0,0118	0,5001	instrument
rs10938397	4	45182527	A	G	4,7E-04	205,009	-0,0315	0,0022	1,7E-45	0,0238	0,0114	0,0370	instrument
rs10962550	9	16720329	C	G	1,0E-04	40,960	0,0192	0,0030	1,7E-10	0,0016	0,0157	0,9164	instrument
rs10992867	9	96461013	A	G	1,3E-04	50,628	0,0185	0,0026	8,9E-13	-0,0064	0,0130	0,6222	instrument

rs11022762	11	13335926	T	C	1,0E-04	41,173	-0,0154	0,0024	5,6E-11	0,0062	0,0115	0,5930	instrument
rs11073383	15	95274349	A	G	1,2E-04	47,790	-0,0159	0,0023	3,3E-12	-0,0066	0,0113	0,5579	instrument
rs11165643	1	96924097	T	C	1,6E-04	67,688	0,0181	0,0022	1,7E-16	0,0019	0,0119	0,8701	instrument
rs112646560	1	39560250	T	C	1,4E-04	37,515	0,0196	0,0032	1,1E-09	0,0198	0,0141	0,1616	instrument
rs11667280	19	30705747	C	G	7,5E-05	29,684	0,0158	0,0029	3,1E-08	0,0118	0,0153	0,4394	instrument
rs11672660	19	46180184	T	C	3,1E-04	131,429	-0,0321	0,0028	5,9E-31	0,0274	0,0145	0,0586	instrument
rs11677607	2	100751150	T	C	2,0E-04	79,012	-0,0240	0,0027	2,9E-19	0,0166	0,0132	0,2082	instrument
rs1167800	7	75176196	A	G	2,0E-04	82,644	0,0200	0,0022	3,7E-19	0,0175	0,0115	0,1266	instrument
rs11692326	2	208263279	T	C	8,3E-05	36,000	0,0156	0,0026	2,0E-09	-0,0071	0,0137	0,6026	instrument
rs11727273	4	55509748	T	C	1,6E-04	42,250	-0,0169	0,0026	1,3E-10	-0,0150	0,0119	0,2095	instrument
rs11751591	6	33794215	A	G	8,5E-05	33,423	-0,0185	0,0032	1,2E-08	0,0249	0,0162	0,1234	instrument
rs11754747	6	141494602	T	C	7,3E-05	31,532	0,0146	0,0026	1,3E-08	0,0045	0,0140	0,7486	instrument
rs11880870	19	18830704	A	G	1,8E-04	73,363	0,0197	0,0023	3,3E-18	-0,0001	0,0120	0,9926	instrument
rs11945861	4	65700865	A	G	7,8E-05	30,864	-0,0150	0,0027	3,1E-08	0,0147	0,0135	0,2763	instrument
rs11971098	7	26699061	A	G	8,5E-05	33,414	-0,0237	0,0041	6,5E-09	0,0293	0,0205	0,1526	instrument
rs12042959	1	243533273	A	G	9,3E-05	36,754	0,0194	0,0032	2,3E-09	0,0309	0,0161	0,0551	instrument
rs12089815	1	91189933	A	G	1,3E-04	34,628	-0,0153	0,0026	6,0E-09	0,0083	0,0122	0,4999	instrument
rs12101625	15	26972431	C	G	8,4E-05	33,062	0,0138	0,0024	5,5E-09	-0,0232	0,0119	0,0508	instrument
rs12140153	1	62579891	T	G	1,8E-04	48,110	-0,0326	0,0047	2,9E-12	-0,0244	0,0216	0,2570	instrument
rs12150665	17	34914787	T	C	1,3E-04	57,622	0,0167	0,0022	5,9E-14	0,0054	0,0127	0,6689	instrument
rs12202969	6	98576223	A	G	1,0E-04	39,745	-0,0145	0,0023	1,9E-10	-0,0028	0,0116	0,8071	instrument
rs12207125	6	154301575	A	G	1,3E-04	34,468	0,0182	0,0031	7,7E-09	-0,0001	0,0139	0,9916	instrument
rs12364470	11	134601012	T	G	1,0E-04	44,444	-0,0200	0,0030	2,5E-11	0,0071	0,0174	0,6828	instrument
rs12429545	13	54102206	A	G	1,8E-04	77,760	0,0291	0,0033	1,1E-18	0,0358	0,0177	0,0429	instrument
rs12448257	16	3599655	A	G	7,2E-05	30,864	0,0150	0,0027	3,1E-08	-0,0126	0,0143	0,3808	instrument
rs12462975	19	30272202	A	G	1,7E-04	65,934	0,0203	0,0025	2,6E-16	0,0594	0,0120	7,1E-07	outlier
rs12564992	1	174478100	A	G	8,0E-05	34,979	-0,0207	0,0035	2,4E-09	-0,0070	0,0178	0,6945	instrument
rs12593036	15	81058652	A	G	9,6E-05	37,945	0,0154	0,0025	6,6E-10	0,0020	0,0125	0,8762	instrument
rs12612009	2	228982603	T	G	8,7E-05	34,515	-0,0141	0,0024	3,8E-09	-0,0070	0,0126	0,5801	instrument
rs12645001	4	96018337	A	G	8,7E-05	34,515	-0,0141	0,0024	4,1E-09	-0,0096	0,0119	0,4209	instrument
rs12680842	8	95582606	A	G	6,8E-05	29,340	0,0130	0,0024	3,4E-08	0,0034	0,0121	0,7758	instrument
rs12681792	8	62054463	A	C	1,0E-04	39,820	0,0183	0,0029	2,6E-10	-0,0235	0,0153	0,1240	instrument

rs12692596	2	161265910	T	C	9,3E-05	40,295	0,0146	0,0023	9,7E-11	0,0151	0,0118	0,2017	instrument
rs12692738	2	165558252	T	C	8,9E-05	38,822	-0,0162	0,0026	2,8E-10	0,0189	0,0134	0,1572	instrument
rs12714199	2	86812549	T	C	1,0E-04	40,295	-0,0146	0,0023	5,1E-10	0,0135	0,0118	0,2533	instrument
rs1285997	14	91513029	C	G	9,2E-05	36,000	-0,0150	0,0025	3,2E-09	-0,0278	0,0127	0,0280	instrument
rs12872889	13	28674628	T	C	1,7E-04	45,453	-0,0209	0,0031	1,5E-11	-0,0234	0,0135	0,0816	instrument
rs1289736	3	107950892	T	C	9,3E-05	36,523	0,0139	0,0023	1,4E-09	-0,0186	0,0115	0,1074	instrument
rs12914489	15	74187937	A	G	9,1E-05	39,760	0,0227	0,0036	1,6E-10	-0,0033	0,0189	0,8613	instrument
rs12949279	17	78558411	T	C	1,5E-04	66,200	0,0179	0,0022	6,1E-16	-0,0032	0,0118	0,7857	instrument
rs12981256	19	1865901	A	G	1,6E-04	62,616	0,0182	0,0023	3,9E-15	-0,0067	0,0116	0,5640	instrument
rs13002158	2	144021712	A	G	1,3E-04	51,443	-0,0208	0,0029	1,2E-12	0,0286	0,0153	0,0612	instrument
rs13053342	22	48871624	A	G	1,0E-04	40,640	0,0153	0,0024	1,3E-10	0,0209	0,0122	0,0863	instrument
rs13062093	3	35667057	T	G	8,3E-05	32,585	-0,0137	0,0024	8,0E-09	-0,0020	0,0118	0,8654	instrument
rs13068138	3	85809425	T	C	1,7E-04	73,197	0,0231	0,0027	3,6E-17	-0,0117	0,0142	0,4093	instrument
rs13076052	3	44456573	C	G	1,2E-04	32,766	-0,0166	0,0029	1,3E-08	0,0116	0,0136	0,3932	instrument
rs13107325	4	103188709	T	C	2,4E-04	105,528	0,0452	0,0044	9,3E-25	-0,0064	0,0226	0,7760	instrument
rs13110266	4	162129844	A	G	9,6E-05	41,661	-0,0142	0,0022	1,5E-10	-0,0096	0,0115	0,4064	instrument
rs13174863	5	139080745	A	G	1,8E-04	75,858	-0,0270	0,0031	4,5E-18	0,0087	0,0178	0,6271	instrument
rs13209872	6	97753223	C	G	8,9E-05	35,007	-0,0142	0,0024	6,1E-09	-0,0040	0,0121	0,7443	instrument
rs13213408	6	162998049	A	T	9,0E-05	35,637	0,0197	0,0033	4,3E-09	0,0385	0,0171	0,0248	instrument
rs1362910	8	30856464	A	G	8,2E-05	33,943	0,0134	0,0023	3,0E-09	0,0000	0,0116	0,9976	instrument
rs1403846	4	119101723	T	C	7,2E-05	31,206	0,0162	0,0029	1,6E-08	-0,0004	0,0149	0,9770	instrument
rs1409818	20	21381121	T	C	1,2E-04	46,387	0,0252	0,0037	1,6E-11	-0,0141	0,0193	0,4652	instrument
rs141729694	5	87999371	T	C	2,2E-04	57,284	0,0386	0,0051	2,5E-14	-0,0390	0,0215	0,0691	instrument
rs1417665	6	104816944	T	C	1,2E-04	47,088	0,0199	0,0029	5,7E-12	-0,0173	0,0152	0,2543	instrument
rs1421598	2	50264710	A	C	9,7E-05	38,360	-0,0192	0,0031	6,8E-10	-0,0040	0,0155	0,7948	instrument
rs1436348	3	104612668	A	G	8,6E-05	33,943	-0,0134	0,0023	6,3E-09	0,0080	0,0116	0,4915	instrument
rs1441264	13	79580919	A	G	1,3E-04	55,276	0,0171	0,0023	5,2E-14	-0,0198	0,0124	0,1097	instrument
rs1452075	3	62481063	T	C	9,4E-05	40,449	0,0159	0,0025	1,5E-10	0,0163	0,0134	0,2247	instrument
rs1491333	4	172186288	T	G	8,1E-05	31,966	-0,0147	0,0026	9,9E-09	-0,0027	0,0138	0,8445	instrument
rs1492014	3	94071481	T	C	1,0E-04	39,745	-0,0145	0,0023	2,8E-10	0,0163	0,0114	0,1538	instrument
rs1518159	18	40760146	T	C	1,3E-04	52,841	-0,0189	0,0026	6,7E-13	0,0092	0,0131	0,4787	instrument
rs1561589	10	126695673	A	G	1,1E-04	47,191	0,0158	0,0023	4,2E-12	-0,0214	0,0118	0,0692	instrument

rs1569979	14	29681294	A	G	1,8E-04	72,565	0,0230	0,0027	1,6E-17	-0,0088	0,0136	0,5198	instrument
rs1579557	6	40371918	T	C	1,8E-04	71,910	0,0212	0,0025	3,1E-17	0,0031	0,0135	0,8189	instrument
rs158186	5	107440856	A	G	2,2E-04	85,711	-0,0287	0,0031	3,2E-20	-0,0037	0,0150	0,8070	instrument
rs159032	5	94206202	T	C	7,8E-05	30,454	0,0149	0,0027	4,5E-08	-0,0227	0,0132	0,0851	instrument
rs1681743	10	118565501	T	C	7,5E-05	29,793	-0,0131	0,0024	3,7E-08	-0,0056	0,0116	0,6314	instrument
rs16851483	3	141275436	T	G	1,6E-04	62,272	0,0363	0,0046	2,3E-15	0,0180	0,0236	0,4444	instrument
rs16912921	9	28413461	A	C	2,7E-04	117,204	0,0249	0,0023	1,6E-26	-0,0079	0,0122	0,5183	instrument
rs16975921	18	39914870	A	T	1,1E-04	42,510	-0,0163	0,0025	3,9E-11	0,0261	0,0121	0,0307	instrument
rs16989232	20	39291784	A	G	7,1E-05	30,972	0,0128	0,0023	1,5E-08	0,0086	0,0121	0,4768	instrument
rs17014375	1	209543560	T	G	7,9E-05	31,228	-0,0190	0,0034	1,5E-08	0,0199	0,0167	0,2348	instrument
rs17066856	18	58049656	T	C	2,7E-04	115,707	0,0398	0,0037	1,4E-27	-0,0173	0,0195	0,3769	instrument
rs17405819	8	76806584	T	C	1,9E-04	82,507	0,0218	0,0024	7,8E-20	-0,0088	0,0123	0,4730	instrument
rs1750307	1	156488420	A	T	8,3E-05	32,585	0,0137	0,0024	8,2E-09	-0,0204	0,0118	0,0837	instrument
rs17522122	14	33302882	T	G	1,3E-04	57,622	0,0167	0,0022	6,5E-14	0,0073	0,0116	0,5293	instrument
rs17619973	3	114417675	A	G	7,4E-05	31,673	0,0242	0,0043	1,3E-08	0,0320	0,0234	0,1704	instrument
rs17724992	19	18454825	A	G	1,1E-04	46,240	0,0170	0,0025	6,7E-12	0,0062	0,0128	0,6273	instrument
rs17751061	19	19413092	T	C	8,2E-05	31,993	-0,0181	0,0032	1,3E-08	0,0458	0,0156	0,0033	outlier
rs17767765	16	24539793	T	C	7,7E-05	30,489	-0,0127	0,0023	2,6E-08	-0,0040	0,0123	0,7435	instrument
rs17806224	20	51065854	A	G	2,3E-04	92,801	-0,0289	0,0030	3,5E-22	-0,0365	0,0156	0,0195	outlier
rs1799923	3	42306294	A	G	1,2E-04	48,203	-0,0243	0,0035	5,1E-12	0,0264	0,0193	0,1709	instrument
rs1801282	3	12393125	C	G	7,0E-05	30,417	-0,0182	0,0033	4,7E-08	-0,0187	0,0170	0,2723	instrument
rs1808579	18	21104888	T	C	1,9E-04	81,000	-0,0198	0,0022	9,4E-20	-0,0129	0,0114	0,2570	instrument
rs1808629	8	73435964	A	G	2,5E-04	65,147	-0,0226	0,0028	5,8E-16	-0,0089	0,0126	0,4813	instrument
rs1812587	5	62991802	T	G	1,3E-04	34,177	0,0152	0,0026	7,0E-09	0,0064	0,0114	0,5738	instrument
rs185350	19	34306816	T	C	7,8E-05	33,851	0,0128	0,0022	7,1E-09	-0,0096	0,0114	0,3988	instrument
rs1861412	2	58893065	A	G	2,2E-04	87,381	-0,0215	0,0023	9,1E-21	0,0002	0,0114	0,9886	instrument
rs1884389	20	1410582	T	C	9,4E-05	37,051	-0,0140	0,0023	1,2E-09	-0,0021	0,0114	0,8534	instrument
rs1884897	20	6612832	A	G	1,2E-04	53,990	-0,0169	0,0023	9,1E-14	0,0179	0,0121	0,1367	instrument
rs1916801	3	61187046	A	T	1,4E-04	59,710	0,0170	0,0022	1,8E-14	-0,0048	0,0119	0,6852	instrument
rs1927790	13	96922191	T	C	8,7E-05	37,655	-0,0135	0,0022	1,3E-09	-0,0049	0,0117	0,6780	instrument
rs1928295	9	120378483	T	C	1,2E-04	52,892	0,0160	0,0022	2,8E-13	0,0042	0,0114	0,7103	instrument
rs1949197	3	86273850	A	G	9,2E-05	36,523	0,0139	0,0023	3,0E-09	-0,0208	0,0116	0,0726	instrument

rs197374	1	112289983	T	C	1,4E-04	55,276	0,0171	0,0023	2,8E-13	0,0058	0,0118	0,6217	instrument
rs1984559	2	135678888	A	G	7,9E-05	29,521	0,0163	0,0030	3,3E-08	0,0338	0,0136	0,0130	outlier
rs198665	6	143154886	A	C	7,8E-05	33,943	0,0134	0,0023	4,4E-09	0,0056	0,0119	0,6368	instrument
rs2012502	16	81728081	A	C	1,0E-04	44,251	0,0153	0,0023	3,0E-11	-0,0155	0,0123	0,2076	instrument
rs2045293	7	114350102	T	C	1,7E-04	67,525	0,0189	0,0023	4,1E-16	-0,0039	0,0116	0,7377	instrument
rs2053682	5	170599327	A	C	1,4E-04	53,582	0,0183	0,0025	1,9E-13	-0,0005	0,0124	0,9696	instrument
rs209416	6	83534729	A	T	1,2E-04	47,840	0,0166	0,0024	8,7E-12	-0,0074	0,0122	0,5465	instrument
rs2112347	5	75015242	T	G	4,2E-04	180,493	0,0309	0,0023	2,1E-42	-0,0343	0,0117	0,0033	instrument
rs214249	16	348687	T	G	1,2E-04	48,418	0,0167	0,0024	1,4E-12	0,0140	0,0116	0,2277	instrument
rs2161097	5	103945178	T	C	8,0E-05	31,457	0,0129	0,0023	2,3E-08	-0,0109	0,0114	0,3399	instrument
rs2178899	6	31606756	A	T	1,3E-04	56,477	0,0248	0,0033	7,2E-14	0,0099	0,0178	0,5787	instrument
rs2185027	6	153381622	A	C	9,5E-05	41,173	-0,0154	0,0024	1,1E-10	-0,0015	0,0123	0,9040	instrument
rs2228213	6	12124855	A	G	9,5E-05	41,406	-0,0148	0,0023	9,2E-11	0,0088	0,0118	0,4530	instrument
rs2267812	7	74138121	A	C	9,2E-05	35,177	-0,0172	0,0029	1,7E-09	-0,0513	0,0138	0,0002	outlier
rs2267958	9	131015279	A	G	1,2E-04	45,002	-0,0161	0,0024	1,9E-11	-0,0123	0,0122	0,3103	instrument
rs2271046	4	52752812	A	T	7,6E-05	30,030	-0,0137	0,0025	2,4E-08	-0,0178	0,0127	0,1607	instrument
rs2289292	16	30097630	T	C	1,5E-04	57,760	-0,0190	0,0025	1,1E-14	-0,0096	0,0128	0,4534	instrument
rs2289705	7	121983843	A	G	7,7E-05	33,284	-0,0150	0,0026	1,5E-08	-0,0171	0,0143	0,2294	instrument
rs2299383	7	103418846	T	C	1,6E-04	68,438	0,0182	0,0022	2,7E-16	-0,0175	0,0116	0,1315	instrument
rs2357760	6	120213880	A	G	1,1E-04	47,790	0,0159	0,0023	1,1E-11	-0,0194	0,0122	0,1097	instrument
rs2465043	6	51180765	A	G	8,9E-05	35,007	-0,0142	0,0024	3,2E-09	-0,0047	0,0118	0,6900	instrument
rs2470520	3	15762920	T	C	7,6E-05	30,011	-0,0126	0,0023	4,2E-08	0,0042	0,0115	0,7175	instrument
rs247975	3	173107443	T	C	1,6E-04	62,616	-0,0182	0,0023	3,6E-15	0,0060	0,0129	0,6397	instrument
rs2479958	13	111984244	A	G	8,1E-05	31,640	0,0135	0,0024	1,4E-08	-0,0181	0,0118	0,1249	instrument
rs2528531	7	93236510	A	C	9,3E-05	36,502	-0,0145	0,0024	1,3E-09	0,0167	0,0118	0,1579	instrument
rs2568958	1	72765116	A	G	2,5E-04	106,464	0,0227	0,0022	1,3E-24	-0,0160	0,0116	0,1690	instrument
rs2605603	11	93221105	A	G	8,3E-05	36,000	-0,0132	0,0022	1,4E-09	0,0139	0,0114	0,2217	instrument
rs2619976	17	71754545	T	C	9,4E-05	37,007	0,0146	0,0024	6,2E-10	0,0092	0,0124	0,4603	instrument
rs2721965	8	116662038	A	C	1,5E-04	60,062	0,0186	0,0024	2,1E-14	-0,0007	0,0120	0,9526	instrument
rs273504	19	18215247	A	G	1,1E-04	43,102	-0,0151	0,0023	1,3E-10	-0,0192	0,0115	0,0947	instrument
rs2754079	14	25886140	A	G	1,2E-04	46,004	-0,0156	0,0023	2,3E-11	0,0140	0,0120	0,2444	instrument
rs2814992	6	34617144	A	G	3,6E-04	155,706	-0,0287	0,0023	1,2E-35	0,0068	0,0121	0,5747	instrument

rs2820295	1	201800868	A	G	2,0E-04	79,507	0,0214	0,0024	8,0E-19	-0,0020	0,0121	0,8704	instrument
rs2836754	21	40291740	T	C	1,1E-04	45,870	-0,0149	0,0022	3,7E-11	-0,0011	0,0117	0,9250	instrument
rs28489620	22	41804716	A	G	1,5E-04	38,955	-0,0181	0,0029	6,9E-10	0,0203	0,0126	0,1066	instrument
rs2861685	2	67837553	T	C	1,8E-04	47,929	0,0180	0,0026	1,1E-11	0,0290	0,0115	0,0118	outlier
rs2862961	11	43656535	A	G	2,3E-04	91,393	-0,0239	0,0025	5,3E-22	0,0054	0,0123	0,6624	instrument
rs2959592	8	17002725	T	C	8,7E-05	36,862	0,0170	0,0028	6,3E-10	0,0016	0,0149	0,9140	instrument
rs2968864	7	150622162	T	C	9,2E-05	36,446	0,0163	0,0027	9,3E-10	-0,0153	0,0133	0,2486	instrument
rs2984618	1	47690438	T	G	1,3E-04	57,622	0,0167	0,0022	2,2E-14	0,0049	0,0116	0,6689	instrument
rs329651	11	133767622	T	G	9,7E-05	41,787	0,0181	0,0028	2,0E-10	-0,0180	0,0148	0,2239	instrument
rs337637	4	38604470	A	G	6,8E-05	29,537	-0,0125	0,0023	3,0E-08	0,0234	0,0119	0,0489	instrument
rs34517439	1	78450517	A	C	3,4E-04	90,481	0,0390	0,0041	2,0E-21	0,0054	0,0196	0,7819	instrument
rs34811474	4	25408838	A	G	4,0E-04	104,566	-0,0317	0,0031	5,2E-24	-0,0217	0,0165	0,1869	instrument
rs34930419	10	33987329	T	C	1,4E-04	36,316	-0,0458	0,0076	1,4E-09	-0,0426	0,0340	0,2097	instrument
rs35107973	4	143125948	T	C	1,2E-04	32,573	0,0234	0,0041	7,7E-09	0,0098	0,0188	0,6044	instrument
rs355754	3	154024165	T	C	9,2E-05	38,117	-0,0142	0,0023	4,3E-10	0,0090	0,0119	0,4476	instrument
rs35722922	2	47002226	A	G	1,8E-04	48,482	0,0188	0,0027	3,6E-12	0,0002	0,0118	0,9859	instrument
rs3803286	14	103246470	A	G	1,3E-04	51,361	0,0172	0,0024	8,3E-13	-0,0049	0,0119	0,6805	instrument
rs3806114	6	20482335	A	G	8,0E-05	34,515	-0,0141	0,0024	4,5E-09	-0,0335	0,0122	0,0060	outlier
rs3826408	17	7101292	T	C	7,0E-05	30,250	0,0121	0,0022	4,5E-08	0,0226	0,0114	0,0477	instrument
rs3829849	9	129390800	T	C	8,0E-05	34,964	0,0136	0,0023	1,9E-09	0,0343	0,0118	0,0037	outlier
rs3977755	10	104420210	T	C	8,0E-05	32,718	-0,0143	0,0025	8,5E-09	0,0141	0,0127	0,2681	instrument
rs4055791	13	59266053	T	C	1,5E-04	39,643	-0,0170	0,0027	1,8E-10	-0,0120	0,0118	0,3104	instrument
rs4148155	4	89054667	A	G	7,7E-05	33,640	0,0203	0,0035	6,2E-09	0,0540	0,0186	0,0038	outlier
rs4240673	8	10787612	T	C	1,5E-04	64,000	0,0176	0,0022	1,0E-15	-0,0155	0,0133	0,2442	instrument
rs427943	21	46570896	A	C	1,5E-04	59,223	-0,0177	0,0023	1,4E-14	0,0122	0,0115	0,2903	instrument
rs429358	19	45411941	T	C	2,2E-04	58,777	0,0276	0,0036	3,2E-14	0,0140	0,0169	0,4085	instrument
rs4295917	1	32173360	A	G	8,2E-05	31,640	0,0135	0,0024	1,6E-08	0,0060	0,0127	0,6371	instrument
rs4307239	7	24354300	A	G	9,5E-05	37,582	-0,0141	0,0023	1,3E-09	0,0173	0,0117	0,1406	instrument
rs4320543	8	34422967	A	G	8,0E-05	31,457	-0,0129	0,0023	2,9E-08	0,0132	0,0123	0,2826	instrument
rs4421623	1	98316285	T	G	1,2E-04	46,441	0,0184	0,0027	1,1E-11	0,0050	0,0134	0,7072	instrument
rs4459316	11	130756176	T	C	1,1E-04	44,832	-0,0154	0,0023	2,3E-11	0,0135	0,0117	0,2477	instrument
rs4474229	1	195037001	A	G	1,6E-04	41,055	-0,0173	0,0027	1,6E-10	-0,0018	0,0116	0,8748	instrument

rs4482463	2	205375909	A	C	2,0E-04	75,524	-0,0365	0,0042	5,2E-18	0,0435	0,0218	0,0461	instrument
rs4569924	5	153540025	T	C	1,2E-04	51,578	0,0158	0,0022	7,4E-13	-0,0200	0,0114	0,0807	instrument
rs4586287	13	99245334	C	G	7,7E-05	30,470	0,0138	0,0025	2,9E-08	-0,0118	0,0122	0,3348	instrument
rs4673553	2	211608379	T	G	1,1E-04	44,251	-0,0153	0,0023	2,6E-11	-0,0123	0,0116	0,2890	instrument
rs4677812	3	194863794	A	C	8,4E-05	33,177	-0,0144	0,0025	9,4E-09	-0,0010	0,0130	0,9363	instrument
rs4776982	15	68114974	A	G	3,3E-04	143,078	0,0311	0,0026	4,2E-33	-0,0047	0,0136	0,7296	instrument
rs4783718	16	69547741	T	C	1,8E-04	78,669	0,0204	0,0023	1,8E-19	0,0317	0,0120	0,0081	outlier
rs4790292	17	1824305	A	C	1,7E-04	63,516	-0,0263	0,0033	1,7E-15	0,0213	0,0167	0,2007	instrument
rs4834272	4	113313986	T	C	8,9E-05	38,656	-0,0143	0,0023	8,2E-10	-0,0214	0,0127	0,0916	instrument
rs4858223	3	20581149	T	C	1,1E-04	41,473	-0,0161	0,0025	8,3E-11	-0,0063	0,0124	0,6125	instrument
rs4864201	4	130731284	T	C	7,5E-05	32,440	0,0131	0,0023	6,3E-09	-0,0026	0,0118	0,8258	instrument
rs4880341	10	133992689	T	C	9,8E-05	38,656	-0,0143	0,0023	4,5E-10	-0,0035	0,0114	0,7613	instrument
rs4889606	16	31011183	A	G	3,1E-04	135,773	0,0268	0,0023	1,3E-32	-0,0119	0,0118	0,3120	instrument
rs4929923	11	8639200	T	C	2,1E-04	92,327	-0,0221	0,0023	2,5E-22	-0,0024	0,0117	0,8365	instrument
rs4936175	11	132641959	T	C	1,2E-04	47,191	-0,0158	0,0023	6,7E-12	-0,0164	0,0114	0,1505	instrument
rs4969387	17	79081724	C	G	8,4E-05	32,532	-0,0154	0,0027	6,9E-09	-0,0174	0,0132	0,1889	instrument
rs4986044	17	21261560	T	C	1,4E-04	61,836	-0,0173	0,0022	2,2E-15	0,0019	0,0115	0,8681	instrument
rs543874	1	177889480	A	G	1,0E-03	455,109	-0,0576	0,0027	8,7E-100	0,0141	0,0144	0,3269	instrument
rs56161855	17	46288649	A	T	1,7E-04	44,787	-0,0261	0,0039	1,5E-11	-0,0143	0,0161	0,3741	instrument
rs563132	9	37245476	A	T	1,4E-04	56,250	0,0180	0,0024	2,0E-14	-0,0136	0,0116	0,2414	instrument
rs563296	10	99772404	A	G	1,4E-04	53,168	0,0175	0,0024	8,8E-14	0,0151	0,0119	0,2053	instrument
rs57221746	15	99230035	A	G	1,2E-04	30,594	-0,0177	0,0032	2,1E-08	0,0076	0,0138	0,5808	instrument
rs57800857	4	140863365	A	C	1,8E-04	47,456	0,0186	0,0027	1,1E-11	0,0324	0,0121	0,0073	outlier
rs6010784	20	61540319	T	C	7,6E-05	32,802	0,0126	0,0022	1,1E-08	-0,0153	0,0113	0,1743	instrument
rs6050446	20	25195509	A	G	8,0E-05	33,785	-0,0372	0,0064	6,2E-09	0,0142	0,0354	0,6880	instrument
rs6142096	20	32686658	A	G	8,6E-05	33,943	0,0134	0,0023	4,2E-09	-0,0249	0,0115	0,0308	instrument
rs61813324	1	156049877	T	C	2,2E-04	58,385	0,0298	0,0039	3,6E-14	-0,0033	0,0188	0,8615	instrument
rs62007782	15	78029797	A	G	1,4E-04	35,601	-0,0179	0,0030	1,9E-09	-0,0090	0,0131	0,4945	instrument
rs62106258	2	417167	T	C	9,7E-04	254,452	0,0989	0,0062	8,7E-58	-0,0126	0,0326	0,6995	instrument
rs62412826	6	61881167	T	C	1,3E-04	33,518	0,0330	0,0057	8,0E-09	-0,0225	0,0259	0,3856	instrument
rs625652	11	65641931	A	T	2,0E-04	52,160	-0,0195	0,0027	9,1E-13	-0,0186	0,0120	0,1196	instrument
rs6265	11	27679916	T	C	4,0E-04	172,734	-0,0368	0,0028	8,0E-40	-0,0184	0,0143	0,1995	instrument

rs6446187	3	49907111	A	C	4,3E-04	114,325	0,0278	0,0026	1,2E-26	0,0359	0,0113	0,0015	outlier
rs6493498	15	51754451	T	C	1,3E-04	54,894	0,0163	0,0022	1,5E-13	0,0148	0,0115	0,1949	instrument
rs6548237	2	621461	A	C	9,1E-04	394,500	-0,0576	0,0029	4,4E-89	0,0591	0,0189	0,0018	outlier
rs6556810	5	164585949	T	C	7,4E-05	29,066	-0,0124	0,0023	5,0E-08	0,0040	0,0117	0,7316	instrument
rs6561937	13	58257667	A	T	1,4E-04	53,236	-0,0197	0,0027	6,0E-13	-0,0383	0,0137	0,0051	outlier
rs6567160	18	57829135	T	C	1,1E-03	475,573	-0,0567	0,0026	8,7E-108	0,0250	0,0134	0,0614	instrument
rs657452	1	49589847	A	G	2,0E-04	85,142	0,0203	0,0022	1,1E-19	-0,0186	0,0117	0,1117	instrument
rs6585198	10	114762237	A	G	1,1E-04	49,000	-0,0154	0,0022	1,2E-12	0,0586	0,0113	1,9E-07	outlier
rs6669189	1	75011358	T	C	2,1E-04	91,115	0,0210	0,0022	4,7E-21	-0,0110	0,0116	0,3407	instrument
rs6720868	2	230663576	T	C	1,3E-04	49,585	0,0169	0,0024	4,6E-12	-0,0033	0,0120	0,7851	instrument
rs6728037	2	59297346	A	G	2,1E-04	81,000	0,0207	0,0023	6,5E-19	0,0112	0,0116	0,3345	instrument
rs6739199	2	105452326	T	C	1,0E-04	40,449	0,0159	0,0025	3,2E-10	-0,0042	0,0136	0,7597	instrument
rs6870983	5	87697533	T	C	1,5E-04	66,485	-0,0212	0,0026	9,6E-16	0,0219	0,0134	0,1026	instrument
rs6890310	5	27193573	A	G	9,6E-05	37,945	-0,0154	0,0025	1,4E-09	-0,0073	0,0132	0,5806	instrument
rs6973700	7	1856669	A	G	1,4E-04	36,376	0,0193	0,0032	1,8E-09	0,0226	0,0140	0,1060	instrument
rs7024334	9	109072075	T	G	8,7E-05	37,398	0,0159	0,0026	1,6E-09	0,0133	0,0137	0,3313	instrument
rs7084454	10	21821274	A	G	2,1E-04	81,000	0,0225	0,0025	7,4E-20	-0,0405	0,0122	0,0009	outlier
rs709400	14	104149475	A	G	1,5E-04	64,000	0,0184	0,0023	4,4E-16	-0,0149	0,0116	0,1995	instrument
rs7105462	11	112912048	A	G	9,9E-05	39,198	0,0144	0,0023	4,4E-10	0,0118	0,0115	0,3063	instrument
rs7107409	11	76492195	T	C	1,2E-04	30,941	-0,0178	0,0032	2,1E-08	0,0003	0,0136	0,9826	instrument
rs7124681	11	47529947	A	C	2,9E-04	126,051	0,0247	0,0022	9,3E-29	-0,0101	0,0115	0,3826	instrument
rs7141420	14	79899454	T	C	2,1E-04	91,115	0,0210	0,0022	7,8E-22	0,0093	0,0120	0,4369	instrument
rs7211966	17	54374611	T	C	8,9E-05	34,515	-0,0470	0,0080	4,9E-09	-0,0095	0,0509	0,8523	instrument
rs7239114	18	45921214	A	G	1,2E-04	49,638	0,0155	0,0022	3,9E-12	-0,0048	0,0120	0,6881	instrument
rs7250833	19	33937277	T	C	8,9E-05	38,543	0,0149	0,0024	7,2E-10	0,0027	0,0127	0,8331	instrument
rs73124396	7	71579606	T	C	1,2E-04	32,111	0,0187	0,0033	1,1E-08	-0,0017	0,0139	0,9052	instrument
rs73213484	4	28489339	A	T	1,7E-04	45,653	0,0250	0,0037	2,3E-11	-0,0034	0,0159	0,8329	instrument
rs7335249	13	109962579	T	C	8,7E-05	34,452	0,0135	0,0023	4,9E-09	0,0087	0,0118	0,4570	instrument
rs7395632	11	958789	T	C	7,4E-05	29,340	-0,0130	0,0024	3,9E-08	0,0045	0,0119	0,7084	instrument
rs7428670	3	131579810	C	G	1,5E-04	60,361	0,0202	0,0026	3,1E-15	-0,0013	0,0130	0,9224	instrument
rs7498044	15	92573639	A	G	1,2E-04	31,640	-0,0180	0,0032	2,7E-08	-0,0137	0,0145	0,3446	instrument
rs7498665	16	28883241	A	G	3,3E-04	143,999	-0,0264	0,0022	1,9E-32	0,0059	0,0116	0,6134	instrument

rs750090	4	152931436	T	C	1,1E-04	42,250	0,0156	0,0024	7,4E-11	0,0114	0,0122	0,3502	instrument
rs7550711	1	110082886	T	C	2,1E-04	88,858	0,0641	0,0068	3,4E-21	-0,1024	0,0336	0,0023	outlier
rs7571496	2	6169351	A	G	7,9E-05	31,102	0,0145	0,0026	1,4E-08	0,0001	0,0132	0,9926	instrument
rs7588437	2	181575281	A	G	1,3E-04	58,555	-0,0176	0,0023	8,4E-15	0,0359	0,0119	0,0025	outlier
rs7594237	2	198187164	A	T	8,3E-05	32,718	-0,0143	0,0025	1,8E-08	-0,0182	0,0131	0,1654	instrument
rs7601870	2	15384392	A	G	8,3E-05	32,937	-0,0132	0,0023	1,2E-08	-0,0037	0,0115	0,7506	instrument
rs7601895	2	55281901	C	G	1,1E-04	41,473	0,0161	0,0025	9,6E-11	-0,0047	0,0128	0,7164	instrument
rs7621025	3	136272246	T	C	1,5E-04	64,000	-0,0200	0,0025	1,6E-15	0,0345	0,0130	0,0079	instrument
rs7647242	3	82642734	T	C	1,0E-04	39,745	0,0145	0,0023	3,9E-10	0,0020	0,0119	0,8650	instrument
rs768023	6	108876002	A	G	1,6E-04	70,712	0,0185	0,0022	1,4E-16	-0,0055	0,0116	0,6359	instrument
rs76900466	2	145057760	T	G	1,2E-04	30,654	0,0227	0,0041	4,5E-08	-0,0129	0,0241	0,5933	instrument
rs77432547	13	86494817	A	G	1,4E-04	36,415	-0,0175	0,0029	2,3E-09	0,0032	0,0127	0,8006	instrument
rs7788008	7	112972483	A	G	1,4E-04	55,276	-0,0171	0,0023	1,1E-13	0,0021	0,0115	0,8544	instrument
rs7805441	7	78121458	T	C	1,0E-04	40,295	0,0146	0,0023	2,0E-10	0,0013	0,0114	0,9068	instrument
rs782971	5	43124688	A	G	1,1E-04	47,334	-0,0172	0,0025	4,9E-12	-0,0058	0,0136	0,6675	instrument
rs786420	2	44719893	T	C	8,4E-05	32,956	0,0155	0,0027	8,8E-09	-0,0062	0,0133	0,6377	instrument
rs78886584	1	16859325	A	G	1,3E-04	33,284	-0,0150	0,0026	1,3E-08	-0,0065	0,0131	0,6169	instrument
rs7899106	10	87410904	A	G	1,3E-04	56,250	-0,0375	0,0050	7,6E-14	-0,0250	0,0267	0,3496	instrument
rs79113395	1	1590521	A	G	1,9E-04	49,000	-0,0210	0,0030	1,5E-12	-0,0087	0,0190	0,6478	instrument
rs8024806	15	53473990	T	C	8,5E-05	33,492	0,0272	0,0047	9,8E-09	0,0104	0,0239	0,6637	instrument
rs8033510	15	61445514	T	C	8,3E-05	32,585	0,0137	0,0024	1,1E-08	-0,0148	0,0117	0,2065	instrument
rs8038522	15	46585105	T	G	9,4E-05	37,051	-0,0140	0,0023	1,3E-09	0,0030	0,0115	0,7941	instrument
rs8075273	17	61728881	A	C	1,0E-04	44,444	-0,0160	0,0024	5,6E-11	0,0101	0,0127	0,4288	instrument
rs811054	16	72251132	T	C	1,2E-04	54,223	0,0162	0,0022	3,2E-13	0,0061	0,0115	0,5926	instrument
rs836525	7	6465909	T	C	8,2E-05	32,233	0,0176	0,0031	9,8E-09	0,0007	0,0155	0,9662	instrument
rs879620	16	4015729	T	C	2,9E-04	115,328	0,0247	0,0023	5,4E-26	-0,0021	0,0120	0,8603	instrument
rs893006	11	64365796	A	C	1,2E-04	51,960	0,0173	0,0024	4,0E-13	0,0023	0,0126	0,8559	instrument
rs895330	19	4060707	C	G	9,9E-05	38,955	0,0181	0,0029	6,6E-10	-0,0059	0,0152	0,6975	instrument
rs915814	21	46493003	A	G	1,3E-04	34,810	-0,0177	0,0030	5,9E-09	0,0116	0,0133	0,3838	instrument
rs925018	3	62713143	C	G	8,8E-05	38,117	-0,0142	0,0023	9,8E-10	0,0184	0,0122	0,1300	instrument
rs9304665	19	47602577	A	T	1,4E-04	57,086	0,0204	0,0027	1,8E-14	-0,0249	0,0133	0,0615	instrument
rs9367369	6	13189941	A	G	1,4E-04	35,587	0,0173	0,0029	2,3E-09	0,0197	0,0124	0,1116	instrument

rs942066	14	94031914	A	G	2,1E-04	55,972	-0,0202	0,0027	1,0E-13	-0,0046	0,0118	0,6998	instrument
rs943005	6	50865820	T	C	4,3E-04	188,351	0,0398	0,0029	2,0E-43	-0,0318	0,0150	0,0336	instrument
rs9531786	13	85983968	C	G	8,4E-05	33,062	-0,0138	0,0024	8,4E-09	-0,0056	0,0118	0,6373	instrument
rs9571687	13	67472713	A	C	9,2E-05	36,502	-0,0145	0,0024	3,2E-09	-0,0035	0,0123	0,7725	instrument
rs9579775	13	20616557	A	C	1,5E-04	39,690	-0,0252	0,0040	3,7E-10	0,0316	0,0216	0,1439	instrument
rs9595630	13	33065443	T	G	9,2E-05	39,745	0,0145	0,0023	2,0E-10	0,0094	0,0117	0,4221	instrument
rs973345	9	29713169	T	C	9,7E-05	38,117	0,0142	0,0023	1,1E-09	0,0043	0,0114	0,7027	instrument
rs9906944	17	47091420	T	C	1,6E-04	62,673	-0,0190	0,0024	7,3E-15	0,0010	0,0126	0,9378	instrument
rs9926784	16	19941968	T	C	1,9E-04	81,000	0,0252	0,0028	6,0E-20	-0,0230	0,0148	0,1199	instrument
rs9937053	16	53799507	A	G	2,2E-03	941,370	0,0675	0,0022	1,0E-200	-0,0740	0,0114	9,7E-11	outlier
rs994596	4	18459828	T	C	9,1E-05	36,000	0,0150	0,0025	1,0E-09	-0,0072	0,0122	0,5556	instrument
rs9955276	18	1839339	T	C	1,2E-04	48,129	0,0222	0,0032	8,0E-12	0,0250	0,0159	0,1162	instrument

Table S7. Single Nucleotide Polymorphisms (SNPs) used as instruments in the Mendelian randomization analyses for the effect of BMI on ER+ breast cancer based on the genome-wide significance threshold P = 5E-08. R^2 represents the explained variance in the exposure. SNP-specific instrument strength is given by the respective F-statistic. Some of the genetic variants were identified as outliers in the iterative approach and therefore excluded from the analyses.

SNP	CHR	POS	EA	OA	R^2	F-stat	β_{BMI}	SE_{BMI}	P_{BMI}	β_{ER+}	SE_{ER+}	P_{ER+}	$variant_{ER+}$
rs10002111	4	67815504	A	G	9,2E-05	36,430	0,0169	0,0028	9,3E-10	-0,0196	0,0089	0,0276	instrument
rs1003081	11	118913993	T	C	9,9E-05	42,843	0,0144	0,0022	6,3E-11	-0,0091	0,0077	0,2355	instrument
rs10048652	2	35449306	T	C	8,7E-05	34,452	0,0135	0,0023	3,5E-09	0,0007	0,0075	0,9210	instrument
rs1009473	9	138777312	T	C	7,7E-05	30,489	-0,0127	0,0023	3,4E-08	0,0085	0,0074	0,2519	instrument
rs10099330	8	143383694	A	G	8,3E-05	32,440	-0,0131	0,0023	9,9E-09	0,0051	0,0075	0,4949	instrument
rs10116186	9	80532374	A	G	1,2E-04	31,102	-0,0145	0,0026	4,1E-08	0,0136	0,0075	0,0700	instrument
rs10118701	9	103061366	A	G	1,4E-04	53,778	-0,0176	0,0024	2,2E-13	-0,0014	0,0079	0,8589	instrument
rs10124645	9	11321119	A	G	8,9E-05	34,964	0,0136	0,0023	5,0E-09	-0,0129	0,0080	0,1038	instrument
rs10182181	2	25150296	A	G	6,2E-04	270,751	-0,0362	0,0022	5,0E-63	0,0342	0,0078	1,3E-05	outlier
rs10237317	7	70045941	A	G	9,1E-05	39,347	-0,0138	0,0022	6,8E-10	-0,0024	0,0077	0,7563	instrument
rs10271582	7	99121950	A	G	1,2E-04	32,222	0,0193	0,0034	8,4E-09	-0,0030	0,0101	0,7647	instrument
rs10471636	5	63979856	A	G	1,3E-04	50,173	-0,0170	0,0024	4,7E-13	-0,0117	0,0080	0,1417	instrument
rs1048932	11	115044850	A	C	1,6E-04	68,438	-0,0182	0,0022	2,5E-16	0,0052	0,0076	0,4929	instrument
rs10499694	7	50614173	A	G	8,8E-05	38,215	0,0136	0,0022	4,0E-10	0,0032	0,0076	0,6731	instrument
rs10513801	3	185822353	T	G	2,8E-04	120,999	0,0352	0,0032	1,3E-27	-0,0024	0,0113	0,8308	instrument

rs10733682	9	129460914	A	G	1,2E-04	51,578	0,0158	0,0022	1,9E-12	-0,0223	0,0076	0,0032	outlier
rs10742752	11	45438374	T	C	8,3E-05	36,000	-0,0132	0,0022	4,0E-09	0,0124	0,0079	0,1141	instrument
rs10756714	9	15885041	A	G	2,1E-04	81,784	0,0208	0,0023	1,5E-19	0,0048	0,0075	0,5158	instrument
rs1075901	17	15943910	T	C	9,9E-05	42,843	-0,0144	0,0022	4,4E-11	-0,0046	0,0075	0,5351	instrument
rs10761785	10	65318766	T	G	1,4E-04	61,124	-0,0172	0,0022	4,2E-15	-0,0296	0,0074	0,0001	outlier
rs10797115	9	92191256	T	C	1,2E-04	48,393	0,0160	0,0023	4,1E-12	-0,0002	0,0075	0,9810	instrument
rs10823893	10	53677313	A	G	1,6E-04	42,009	0,0175	0,0027	5,2E-11	-0,0193	0,0077	0,0118	instrument
rs10824347	10	77645229	A	T	1,1E-04	42,491	0,0176	0,0027	5,5E-11	0,0125	0,0089	0,1617	instrument
rs10909880	1	2727804	T	C	7,5E-05	31,768	-0,0124	0,0022	2,0E-08	0,0076	0,0075	0,3116	instrument
rs10920678	1	190239907	A	G	1,3E-04	54,894	0,0163	0,0022	7,5E-14	-0,0060	0,0078	0,4392	instrument
rs10938397	4	45182527	A	G	4,7E-04	205,009	-0,0315	0,0022	1,7E-45	0,0192	0,0074	0,0099	instrument
rs10962550	9	16720329	C	G	1,0E-04	40,960	0,0192	0,0030	1,7E-10	0,0180	0,0103	0,0814	instrument
rs10992867	9	96461013	A	G	1,3E-04	50,628	0,0185	0,0026	8,9E-13	-0,0012	0,0085	0,8855	instrument
rs11022762	11	13335926	T	C	1,0E-04	41,173	-0,0154	0,0024	5,6E-11	-0,0024	0,0075	0,7548	instrument
rs11073383	15	95274349	A	G	1,2E-04	47,790	-0,0159	0,0023	3,3E-12	0,0096	0,0074	0,1937	instrument
rs11165643	1	96924097	T	C	1,6E-04	67,688	0,0181	0,0022	1,7E-16	-0,0115	0,0078	0,1406	instrument
rs112646560	1	39560250	T	C	1,4E-04	37,515	0,0196	0,0032	1,1E-09	0,0063	0,0094	0,5014	instrument
rs11667280	19	30705747	C	G	7,5E-05	29,684	0,0158	0,0029	3,1E-08	0,0161	0,0101	0,1125	instrument
rs11672660	19	46180184	T	C	3,1E-04	131,429	-0,0321	0,0028	5,9E-31	0,0557	0,0095	5,1E-09	outlier
rs11677607	2	100751150	T	C	2,0E-04	79,012	-0,0240	0,0027	2,9E-19	0,0129	0,0087	0,1366	instrument
rs1167800	7	75176196	A	G	2,0E-04	82,644	0,0200	0,0022	3,7E-19	0,0078	0,0075	0,2974	instrument
rs11692326	2	208263279	T	C	8,3E-05	36,000	0,0156	0,0026	2,0E-09	0,0080	0,0090	0,3716	instrument
rs11727273	4	55509748	T	C	1,6E-04	42,250	-0,0169	0,0026	1,3E-10	0,0096	0,0079	0,2212	instrument
rs11751591	6	33794215	A	G	8,5E-05	33,423	-0,0185	0,0032	1,2E-08	-0,0003	0,0106	0,9795	instrument
rs11754747	6	141494602	T	C	7,3E-05	31,532	0,0146	0,0026	1,3E-08	0,0096	0,0091	0,2924	instrument
rs11880870	19	18830704	A	G	1,8E-04	73,363	0,0197	0,0023	3,3E-18	0,0178	0,0079	0,0242	instrument
rs11945861	4	65700865	A	G	7,8E-05	30,864	-0,0150	0,0027	3,1E-08	-0,0145	0,0089	0,1055	instrument
rs11971098	7	26699061	A	G	8,5E-05	33,414	-0,0237	0,0041	6,5E-09	-0,0209	0,0132	0,1129	instrument
rs12042959	1	243533273	A	G	9,3E-05	36,754	0,0194	0,0032	2,3E-09	0,0128	0,0105	0,2232	instrument
rs12089815	1	91189933	A	G	1,3E-04	34,628	-0,0153	0,0026	6,0E-09	0,0050	0,0081	0,5346	instrument
rs12101625	15	26972431	C	G	8,4E-05	33,062	0,0138	0,0024	5,5E-09	-0,0193	0,0078	0,0138	instrument
rs12140153	1	62579891	T	G	1,8E-04	48,110	-0,0326	0,0047	2,9E-12	0,0286	0,0139	0,0391	instrument

rs12150665	17	34914787	T	C	1,3E-04	57,622	0,0167	0,0022	5,9E-14	0,0200	0,0085	0,0187	instrument
rs12202969	6	98576223	A	G	1,0E-04	39,745	-0,0145	0,0023	1,9E-10	-0,0027	0,0076	0,7199	instrument
rs12207125	6	154301575	A	G	1,3E-04	34,468	0,0182	0,0031	7,7E-09	-0,0147	0,0091	0,1079	instrument
rs12364470	11	134601012	T	G	1,0E-04	44,444	-0,0200	0,0030	2,5E-11	0,0064	0,0116	0,5837	instrument
rs12429545	13	54102206	A	G	1,8E-04	77,760	0,0291	0,0033	1,1E-18	0,0100	0,0117	0,3944	instrument
rs12448257	16	3599655	A	G	7,2E-05	30,864	0,0150	0,0027	3,1E-08	-0,0026	0,0094	0,7809	instrument
rs12462975	19	30272202	A	G	1,7E-04	65,934	0,0203	0,0025	2,6E-16	0,0171	0,0079	0,0303	instrument
rs12564992	1	174478100	A	G	8,0E-05	34,979	-0,0207	0,0035	2,4E-09	-0,0037	0,0117	0,7488	instrument
rs12593036	15	81058652	A	G	9,6E-05	37,945	0,0154	0,0025	6,6E-10	-0,0065	0,0082	0,4269	instrument
rs12612009	2	228982603	T	G	8,7E-05	34,515	-0,0141	0,0024	3,8E-09	0,0008	0,0083	0,9203	instrument
rs12645001	4	96018337	A	G	8,7E-05	34,515	-0,0141	0,0024	4,1E-09	-0,0121	0,0078	0,1233	instrument
rs12680842	8	95582606	A	G	6,8E-05	29,340	0,0130	0,0024	3,4E-08	-0,0021	0,0079	0,7932	instrument
rs12681792	8	62054463	A	C	1,0E-04	39,820	0,0183	0,0029	2,6E-10	0,0146	0,0100	0,1429	instrument
rs12692596	2	161265910	T	C	9,3E-05	40,295	0,0146	0,0023	9,7E-11	0,0081	0,0077	0,2924	instrument
rs12692738	2	165558252	T	C	8,9E-05	38,822	-0,0162	0,0026	2,8E-10	-0,0012	0,0088	0,8905	instrument
rs12714199	2	86812549	T	C	1,0E-04	40,295	-0,0146	0,0023	5,1E-10	0,0051	0,0077	0,5137	instrument
rs1285997	14	91513029	C	G	9,2E-05	36,000	-0,0150	0,0025	3,2E-09	0,0088	0,0082	0,2872	instrument
rs12872889	13	28674628	T	C	1,7E-04	45,453	-0,0209	0,0031	1,5E-11	0,0107	0,0089	0,2277	instrument
rs1289736	3	107950892	T	C	9,3E-05	36,523	0,0139	0,0023	1,4E-09	-0,0042	0,0076	0,5760	instrument
rs12914489	15	74187937	A	G	9,1E-05	39,760	0,0227	0,0036	1,6E-10	0,0219	0,0123	0,0754	instrument
rs12949279	17	78558411	T	C	1,5E-04	66,200	0,0179	0,0022	6,1E-16	-0,0043	0,0078	0,5787	instrument
rs12981256	19	1865901	A	G	1,6E-04	62,616	0,0182	0,0023	3,9E-15	0,0038	0,0076	0,6125	instrument
rs13002158	2	144021712	A	G	1,3E-04	51,443	-0,0208	0,0029	1,2E-12	0,0141	0,0100	0,1592	instrument
rs13053342	22	48871624	A	G	1,0E-04	40,640	0,0153	0,0024	1,3E-10	-0,0073	0,0081	0,3686	instrument
rs13062093	3	35667057	T	G	8,3E-05	32,585	-0,0137	0,0024	8,0E-09	-0,0037	0,0077	0,6339	instrument
rs13068138	3	85809425	T	C	1,7E-04	73,197	0,0231	0,0027	3,6E-17	-0,0054	0,0093	0,5630	instrument
rs13076052	3	44456573	C	G	1,2E-04	32,766	-0,0166	0,0029	1,3E-08	0,0112	0,0088	0,2034	instrument
rs13107325	4	103188709	T	C	2,4E-04	105,528	0,0452	0,0044	9,3E-25	0,0201	0,0149	0,1779	instrument
rs13110266	4	162129844	A	G	9,6E-05	41,661	-0,0142	0,0022	1,5E-10	-0,0014	0,0076	0,8575	instrument
rs13174863	5	139080745	A	G	1,8E-04	75,858	-0,0270	0,0031	4,5E-18	-0,0306	0,0116	0,0084	outlier
rs13209872	6	97753223	C	G	8,9E-05	35,007	-0,0142	0,0024	6,1E-09	-0,0035	0,0079	0,6624	instrument
rs13213408	6	162998049	A	T	9,0E-05	35,637	0,0197	0,0033	4,3E-09	-0,0032	0,0111	0,7698	instrument

rs1362910	8	30856464	A	G	8,2E-05	33,943	0,0134	0,0023	3,0E-09	0,0088	0,0076	0,2468	instrument
rs1403846	4	119101723	T	C	7,2E-05	31,206	0,0162	0,0029	1,6E-08	0,0083	0,0098	0,3974	instrument
rs1409818	20	21381121	T	C	1,2E-04	46,387	0,0252	0,0037	1,6E-11	-0,0234	0,0127	0,0651	instrument
rs141729694	5	87999371	T	C	2,2E-04	57,284	0,0386	0,0051	2,5E-14	-0,0152	0,0140	0,2794	instrument
rs1417665	6	104816944	T	C	1,2E-04	47,088	0,0199	0,0029	5,7E-12	-0,0251	0,0100	0,0124	instrument
rs1421598	2	50264710	A	C	9,7E-05	38,360	-0,0192	0,0031	6,8E-10	-0,0242	0,0101	0,0166	outlier
rs1436348	3	104612668	A	G	8,6E-05	33,943	-0,0134	0,0023	6,3E-09	0,0018	0,0077	0,8185	instrument
rs1441264	13	79580919	A	G	1,3E-04	55,276	0,0171	0,0023	5,2E-14	-0,0066	0,0083	0,4263	instrument
rs1452075	3	62481063	T	C	9,4E-05	40,449	0,0159	0,0025	1,5E-10	0,0169	0,0089	0,0569	instrument
rs1491333	4	172186288	T	G	8,1E-05	31,966	-0,0147	0,0026	9,9E-09	-0,0011	0,0091	0,9016	instrument
rs1492014	3	94071481	T	C	1,0E-04	39,745	-0,0145	0,0023	2,8E-10	0,0060	0,0075	0,4255	instrument
rs1518159	18	40760146	T	C	1,3E-04	52,841	-0,0189	0,0026	6,7E-13	-0,0128	0,0085	0,1345	instrument
rs1561589	10	126695673	A	G	1,1E-04	47,191	0,0158	0,0023	4,2E-12	0,0017	0,0077	0,8233	instrument
rs1569979	14	29681294	A	G	1,8E-04	72,565	0,0230	0,0027	1,6E-17	-0,0023	0,0089	0,7936	instrument
rs1579557	6	40371918	T	C	1,8E-04	71,910	0,0212	0,0025	3,1E-17	-0,0010	0,0090	0,9126	instrument
rs158186	5	107440856	A	G	2,2E-04	85,711	-0,0287	0,0031	3,2E-20	-0,0011	0,0098	0,9094	instrument
rs159032	5	94206202	T	C	7,8E-05	30,454	0,0149	0,0027	4,5E-08	-0,0132	0,0086	0,1238	instrument
rs1681743	10	118565501	T	C	7,5E-05	29,793	-0,0131	0,0024	3,7E-08	-0,0107	0,0076	0,1627	instrument
rs16851483	3	141275436	T	G	1,6E-04	62,272	0,0363	0,0046	2,3E-15	0,0214	0,0154	0,1643	instrument
rs16912921	9	28413461	A	C	2,7E-04	117,204	0,0249	0,0023	1,6E-26	-0,0001	0,0079	0,9901	instrument
rs16975921	18	39914870	A	T	1,1E-04	42,510	-0,0163	0,0025	3,9E-11	0,0129	0,0079	0,1038	instrument
rs16989232	20	39291784	A	G	7,1E-05	30,972	0,0128	0,0023	1,5E-08	-0,0163	0,0080	0,0432	instrument
rs17014375	1	209543560	T	G	7,9E-05	31,228	-0,0190	0,0034	1,5E-08	0,0144	0,0109	0,1878	instrument
rs17066856	18	58049656	T	C	2,7E-04	115,707	0,0398	0,0037	1,4E-27	-0,0074	0,0127	0,5626	instrument
rs17405819	8	76806584	T	C	1,9E-04	82,507	0,0218	0,0024	7,8E-20	-0,0141	0,0080	0,0786	instrument
rs1750307	1	156488420	A	T	8,3E-05	32,585	0,0137	0,0024	8,2E-09	0,0018	0,0077	0,8190	instrument
rs17522122	14	33302882	T	G	1,3E-04	57,622	0,0167	0,0022	6,5E-14	-0,0052	0,0077	0,4995	instrument
rs17619973	3	114417675	A	G	7,4E-05	31,673	0,0242	0,0043	1,3E-08	0,0038	0,0153	0,8037	instrument
rs17724992	19	18454825	A	G	1,1E-04	46,240	0,0170	0,0025	6,7E-12	0,0001	0,0084	0,9914	instrument
rs17751061	19	19413092	T	C	8,2E-05	31,993	-0,0181	0,0032	1,3E-08	0,0327	0,0103	0,0015	outlier
rs17767765	16	24539793	T	C	7,7E-05	30,489	-0,0127	0,0023	2,6E-08	-0,0031	0,0082	0,7004	instrument
rs17806224	20	51065854	A	G	2,3E-04	92,801	-0,0289	0,0030	3,5E-22	-0,0249	0,0102	0,0151	outlier

rs1799923	3	42306294	A	G	1,2E-04	48,203	-0,0243	0,0035	5,1E-12	0,0254	0,0128	0,0466	instrument
rs1801282	3	12393125	C	G	7,0E-05	30,417	-0,0182	0,0033	4,7E-08	0,0246	0,0112	0,0282	instrument
rs1808579	18	21104888	T	C	1,9E-04	81,000	-0,0198	0,0022	9,4E-20	-0,0147	0,0075	0,0487	instrument
rs1808629	8	73435964	A	G	2,5E-04	65,147	-0,0226	0,0028	5,8E-16	-0,0119	0,0083	0,1540	instrument
rs1812587	5	62991802	T	G	1,3E-04	34,177	0,0152	0,0026	7,0E-09	0,0110	0,0075	0,1421	instrument
rs185350	19	34306816	T	C	7,8E-05	33,851	0,0128	0,0022	7,1E-09	-0,0097	0,0074	0,1911	instrument
rs1861412	2	58893065	A	G	2,2E-04	87,381	-0,0215	0,0023	9,1E-21	-0,0017	0,0074	0,8223	instrument
rs1884389	20	1410582	T	C	9,4E-05	37,051	-0,0140	0,0023	1,2E-09	0,0087	0,0074	0,2438	instrument
rs1884897	20	6612832	A	G	1,2E-04	53,990	-0,0169	0,0023	9,1E-14	0,0046	0,0079	0,5612	instrument
rs1916801	3	61187046	A	T	1,4E-04	59,710	0,0170	0,0022	1,8E-14	0,0007	0,0077	0,9239	instrument
rs1927790	13	96922191	T	C	8,7E-05	37,655	-0,0135	0,0022	1,3E-09	-0,0024	0,0077	0,7594	instrument
rs1928295	9	120378483	T	C	1,2E-04	52,892	0,0160	0,0022	2,8E-13	-0,0015	0,0075	0,8420	instrument
rs1949197	3	86273850	A	G	9,2E-05	36,523	0,0139	0,0023	3,0E-09	-0,0074	0,0076	0,3291	instrument
rs197374	1	112289983	T	C	1,4E-04	55,276	0,0171	0,0023	2,8E-13	-0,0083	0,0078	0,2876	instrument
rs1984559	2	135678888	A	G	7,9E-05	29,521	0,0163	0,0030	3,3E-08	0,0178	0,0089	0,0463	instrument
rs198665	6	143154886	A	C	7,8E-05	33,943	0,0134	0,0023	4,4E-09	-0,0045	0,0078	0,5668	instrument
rs2012502	16	81728081	A	C	1,0E-04	44,251	0,0153	0,0023	3,0E-11	-0,0058	0,0080	0,4693	instrument
rs2045293	7	114350102	T	C	1,7E-04	67,525	0,0189	0,0023	4,1E-16	-0,0204	0,0076	0,0071	instrument
rs2053682	5	170599327	A	C	1,4E-04	53,582	0,0183	0,0025	1,9E-13	0,0018	0,0081	0,8291	instrument
rs209416	6	83534729	A	T	1,2E-04	47,840	0,0166	0,0024	8,7E-12	-0,0158	0,0080	0,0487	instrument
rs2112347	5	75015242	T	G	4,2E-04	180,493	0,0309	0,0023	2,1E-42	-0,0154	0,0076	0,0440	instrument
rs214249	16	348687	T	G	1,2E-04	48,418	0,0167	0,0024	1,4E-12	0,0093	0,0075	0,2193	instrument
rs2161097	5	103945178	T	C	8,0E-05	31,457	0,0129	0,0023	2,3E-08	0,0070	0,0075	0,3486	instrument
rs2178899	6	31606756	A	T	1,3E-04	56,477	0,0248	0,0033	7,2E-14	-0,0025	0,0115	0,8283	instrument
rs2185027	6	153381622	A	C	9,5E-05	41,173	-0,0154	0,0024	1,1E-10	0,0093	0,0081	0,2502	instrument
rs2228213	6	12124855	A	G	9,5E-05	41,406	-0,0148	0,0023	9,2E-11	-0,0032	0,0077	0,6782	instrument
rs2267812	7	74138121	A	C	9,2E-05	35,177	-0,0172	0,0029	1,7E-09	-0,0162	0,0091	0,0749	instrument
rs2267958	9	131015279	A	G	1,2E-04	45,002	-0,0161	0,0024	1,9E-11	-0,0026	0,0080	0,7405	instrument
rs2271046	4	52752812	A	T	7,6E-05	30,030	-0,0137	0,0025	2,4E-08	-0,0183	0,0083	0,0275	instrument
rs2289292	16	30097630	T	C	1,5E-04	57,760	-0,0190	0,0025	1,1E-14	-0,0006	0,0084	0,9404	instrument
rs2289705	7	121983843	A	G	7,7E-05	33,284	-0,0150	0,0026	1,5E-08	-0,0142	0,0095	0,1322	instrument
rs2299383	7	103418846	T	C	1,6E-04	68,438	0,0182	0,0022	2,7E-16	-0,0079	0,0076	0,3020	instrument

rs2357760	6	120213880	A	G	1,1E-04	47,790	0,0159	0,0023	1,1E-11	-0,0017	0,0080	0,8334	instrument
rs2465043	6	51180765	A	G	8,9E-05	35,007	-0,0142	0,0024	3,2E-09	0,0056	0,0078	0,4689	instrument
rs2470520	3	15762920	T	C	7,6E-05	30,011	-0,0126	0,0023	4,2E-08	-0,0142	0,0075	0,0596	instrument
rs247975	3	173107443	T	C	1,6E-04	62,616	-0,0182	0,0023	3,6E-15	-0,0056	0,0085	0,5136	instrument
rs2479958	13	111984244	A	G	8,1E-05	31,640	0,0135	0,0024	1,4E-08	0,0058	0,0078	0,4562	instrument
rs2528531	7	93236510	A	C	9,3E-05	36,502	-0,0145	0,0024	1,3E-09	0,0053	0,0077	0,4900	instrument
rs2568958	1	72765116	A	G	2,5E-04	106,464	0,0227	0,0022	1,3E-24	-0,0189	0,0076	0,0125	instrument
rs2605603	11	93221105	A	G	8,3E-05	36,000	-0,0132	0,0022	1,4E-09	-0,0066	0,0075	0,3788	instrument
rs2619976	17	71754545	T	C	9,4E-05	37,007	0,0146	0,0024	6,2E-10	0,0019	0,0082	0,8187	instrument
rs2721965	8	116662038	A	C	1,5E-04	60,062	0,0186	0,0024	2,1E-14	0,0314	0,0079	0,0001	outlier
rs273504	19	18215247	A	G	1,1E-04	43,102	-0,0151	0,0023	1,3E-10	-0,0159	0,0075	0,0344	instrument
rs2754079	14	25886140	A	G	1,2E-04	46,004	-0,0156	0,0023	2,3E-11	0,0163	0,0080	0,0403	instrument
rs2814992	6	34617144	A	G	3,6E-04	155,706	-0,0287	0,0023	1,2E-35	0,0040	0,0079	0,6161	instrument
rs2820295	1	201800868	A	G	2,0E-04	79,507	0,0214	0,0024	8,0E-19	-0,0099	0,0079	0,2117	instrument
rs2836754	21	40291740	T	C	1,1E-04	45,870	-0,0149	0,0022	3,7E-11	0,0032	0,0076	0,6802	instrument
rs28489620	22	41804716	A	G	1,5E-04	38,955	-0,0181	0,0029	6,9E-10	0,0261	0,0082	0,0015	outlier
rs2861685	2	67837553	T	C	1,8E-04	47,929	0,0180	0,0026	1,1E-11	0,0144	0,0075	0,0564	instrument
rs2862961	11	43656535	A	G	2,3E-04	91,393	-0,0239	0,0025	5,3E-22	-0,0085	0,0080	0,2910	instrument
rs2959592	8	17002725	T	C	8,7E-05	36,862	0,0170	0,0028	6,3E-10	0,0110	0,0098	0,2601	instrument
rs2968864	7	150622162	T	C	9,2E-05	36,446	0,0163	0,0027	9,3E-10	-0,0234	0,0087	0,0071	outlier
rs2984618	1	47690438	T	G	1,3E-04	57,622	0,0167	0,0022	2,2E-14	-0,0108	0,0076	0,1540	instrument
rs329651	11	133767622	T	G	9,7E-05	41,787	0,0181	0,0028	2,0E-10	-0,0168	0,0097	0,0816	instrument
rs337637	4	38604470	A	G	6,8E-05	29,537	-0,0125	0,0023	3,0E-08	-0,0077	0,0078	0,3240	instrument
rs34517439	1	78450517	A	C	3,4E-04	90,481	0,0390	0,0041	2,0E-21	0,0157	0,0126	0,2146	instrument
rs34811474	4	25408838	A	G	4,0E-04	104,566	-0,0317	0,0031	5,2E-24	0,0080	0,0106	0,4495	instrument
rs34930419	10	33987329	T	C	1,4E-04	36,316	-0,0458	0,0076	1,4E-09	-0,0021	0,0222	0,9260	instrument
rs35107973	4	143125948	T	C	1,2E-04	32,573	0,0234	0,0041	7,7E-09	0,0196	0,0125	0,1163	instrument
rs355754	3	154024165	T	C	9,2E-05	38,117	-0,0142	0,0023	4,3E-10	0,0055	0,0078	0,4781	instrument
rs35722922	2	47002226	A	G	1,8E-04	48,482	0,0188	0,0027	3,6E-12	0,0074	0,0077	0,3380	instrument
rs3803286	14	103246470	A	G	1,3E-04	51,361	0,0172	0,0024	8,3E-13	-0,0257	0,0078	0,0010	outlier
rs3806114	6	20482335	A	G	8,0E-05	34,515	-0,0141	0,0024	4,5E-09	-0,0187	0,0080	0,0200	instrument
rs3826408	17	7101292	T	C	7,0E-05	30,250	0,0121	0,0022	4,5E-08	0,0170	0,0075	0,0237	instrument

rs3829849	9	129390800	T	C	8,0E-05	34,964	0,0136	0,0023	1,9E-09	0,0171	0,0077	0,0265	instrument
rs3977755	10	104420210	T	C	8,0E-05	32,718	-0,0143	0,0025	8,5E-09	0,0170	0,0083	0,0407	instrument
rs4055791	13	59266053	T	C	1,5E-04	39,643	-0,0170	0,0027	1,8E-10	-0,0176	0,0078	0,0238	instrument
rs4148155	4	89054667	A	G	7,7E-05	33,640	0,0203	0,0035	6,2E-09	0,0365	0,0121	0,0027	outlier
rs4240673	8	10787612	T	C	1,5E-04	64,000	0,0176	0,0022	1,0E-15	-0,0134	0,0089	0,1326	instrument
rs427943	21	46570896	A	C	1,5E-04	59,223	-0,0177	0,0023	1,4E-14	0,0058	0,0075	0,4459	instrument
rs429358	19	45411941	T	C	2,2E-04	58,777	0,0276	0,0036	3,2E-14	0,0230	0,0109	0,0354	instrument
rs4295917	1	32173360	A	G	8,2E-05	31,640	0,0135	0,0024	1,6E-08	-0,0161	0,0084	0,0568	instrument
rs4307239	7	24354300	A	G	9,5E-05	37,582	-0,0141	0,0023	1,3E-09	0,0130	0,0077	0,0925	instrument
rs4320543	8	34422967	A	G	8,0E-05	31,457	-0,0129	0,0023	2,9E-08	0,0040	0,0080	0,6230	instrument
rs4421623	1	98316285	T	G	1,2E-04	46,441	0,0184	0,0027	1,1E-11	0,0064	0,0088	0,4642	instrument
rs4459316	11	130756176	T	C	1,1E-04	44,832	-0,0154	0,0023	2,3E-11	0,0060	0,0077	0,4361	instrument
rs4474229	1	195037001	A	G	1,6E-04	41,055	-0,0173	0,0027	1,6E-10	0,0098	0,0076	0,1993	instrument
rs4482463	2	205375909	A	C	2,0E-04	75,524	-0,0365	0,0042	5,2E-18	0,0181	0,0142	0,2038	instrument
rs4569924	5	153540025	T	C	1,2E-04	51,578	0,0158	0,0022	7,4E-13	-0,0034	0,0075	0,6485	instrument
rs4586287	13	99245334	C	G	7,7E-05	30,470	0,0138	0,0025	2,9E-08	-0,0161	0,0080	0,0444	instrument
rs4673553	2	211608379	T	G	1,1E-04	44,251	-0,0153	0,0023	2,6E-11	0,0083	0,0076	0,2724	instrument
rs4677812	3	194863794	A	C	8,4E-05	33,177	-0,0144	0,0025	9,4E-09	-0,0011	0,0085	0,8981	instrument
rs4776982	15	68114974	A	G	3,3E-04	143,078	0,0311	0,0026	4,2E-33	-0,0102	0,0089	0,2492	instrument
rs4783718	16	69547741	T	C	1,8E-04	78,669	0,0204	0,0023	1,8E-19	0,0088	0,0079	0,2661	instrument
rs4790292	17	1824305	A	C	1,7E-04	63,516	-0,0263	0,0033	1,7E-15	0,0179	0,0110	0,1042	instrument
rs4834272	4	113313986	T	C	8,9E-05	38,656	-0,0143	0,0023	8,2E-10	0,0002	0,0084	0,9832	instrument
rs4858223	3	20581149	T	C	1,1E-04	41,473	-0,0161	0,0025	8,3E-11	0,0031	0,0081	0,7031	instrument
rs4864201	4	130731284	T	C	7,5E-05	32,440	0,0131	0,0023	6,3E-09	0,0035	0,0077	0,6471	instrument
rs4880341	10	133992689	T	C	9,8E-05	38,656	-0,0143	0,0023	4,5E-10	-0,0003	0,0075	0,9706	instrument
rs4889606	16	31011183	A	G	3,1E-04	135,773	0,0268	0,0023	1,3E-32	-0,0034	0,0077	0,6566	instrument
rs4929923	11	8639200	T	C	2,1E-04	92,327	-0,0221	0,0023	2,5E-22	0,0005	0,0077	0,9476	instrument
rs4936175	11	132641959	T	C	1,2E-04	47,191	-0,0158	0,0023	6,7E-12	0,0132	0,0075	0,0778	instrument
rs4969387	17	79081724	C	G	8,4E-05	32,532	-0,0154	0,0027	6,9E-09	-0,0155	0,0086	0,0729	instrument
rs4986044	17	21261560	T	C	1,4E-04	61,836	-0,0173	0,0022	2,2E-15	0,0040	0,0076	0,6009	instrument
rs543874	1	177889480	A	G	1,0E-03	455,109	-0,0576	0,0027	8,7E-100	0,0252	0,0094	0,0073	instrument
rs56161855	17	46288649	A	T	1,7E-04	44,787	-0,0261	0,0039	1,5E-11	0,0008	0,0105	0,9422	instrument

rs563132	9	37245476	A	T	1,4E-04	56,250	0,0180	0,0024	2,0E-14	-0,0228	0,0076	0,0028	outlier
rs563296	10	99772404	A	G	1,4E-04	53,168	0,0175	0,0024	8,8E-14	0,0002	0,0079	0,9808	instrument
rs57221746	15	99230035	A	G	1,2E-04	30,594	-0,0177	0,0032	2,1E-08	0,0160	0,0090	0,0748	instrument
rs57800857	4	140863365	A	C	1,8E-04	47,456	0,0186	0,0027	1,1E-11	0,0114	0,0079	0,1499	instrument
rs6010784	20	61540319	T	C	7,6E-05	32,802	0,0126	0,0022	1,1E-08	0,0058	0,0074	0,4296	instrument
rs6050446	20	25195509	A	G	8,0E-05	33,785	-0,0372	0,0064	6,2E-09	-0,0085	0,0230	0,7124	instrument
rs6142096	20	32686658	A	G	8,6E-05	33,943	0,0134	0,0023	4,2E-09	-0,0016	0,0075	0,8335	instrument
rs61813324	1	156049877	T	C	2,2E-04	58,385	0,0298	0,0039	3,6E-14	-0,0271	0,0122	0,0265	instrument
rs62007782	15	78029797	A	G	1,4E-04	35,601	-0,0179	0,0030	1,9E-09	-0,0007	0,0086	0,9345	instrument
rs62106258	2	417167	T	C	9,7E-04	254,452	0,0989	0,0062	8,7E-58	-0,0324	0,0214	0,1305	instrument
rs62412826	6	61881167	T	C	1,3E-04	33,518	0,0330	0,0057	8,0E-09	0,0330	0,0167	0,0484	instrument
rs625652	11	65641931	A	T	2,0E-04	52,160	-0,0195	0,0027	9,1E-13	-0,0359	0,0077	3,4E-06	outlier
rs6265	11	27679916	T	C	4,0E-04	172,734	-0,0368	0,0028	8,0E-40	-0,0005	0,0093	0,9552	instrument
rs6446187	3	49907111	A	C	4,3E-04	114,325	0,0278	0,0026	1,2E-26	0,0188	0,0074	0,0109	outlier
rs6493498	15	51754451	T	C	1,3E-04	54,894	0,0163	0,0022	1,5E-13	-0,0201	0,0075	0,0076	instrument
rs6548237	2	621461	A	C	9,1E-04	394,500	-0,0576	0,0029	4,4E-89	0,0193	0,0125	0,1223	instrument
rs6556810	5	164585949	T	C	7,4E-05	29,066	-0,0124	0,0023	5,0E-08	-0,0069	0,0077	0,3674	instrument
rs6561937	13	58257667	A	T	1,4E-04	53,236	-0,0197	0,0027	6,0E-13	-0,0205	0,0090	0,0225	instrument
rs6567160	18	57829135	T	C	1,1E-03	475,573	-0,0567	0,0026	8,7E-108	0,0132	0,0087	0,1309	instrument
rs657452	1	49589847	A	G	2,0E-04	85,142	0,0203	0,0022	1,1E-19	-0,0116	0,0076	0,1279	instrument
rs6585198	10	114762237	A	G	1,1E-04	49,000	-0,0154	0,0022	1,2E-12	0,0310	0,0074	2,6E-05	outlier
rs6669189	1	75011358	T	C	2,1E-04	91,115	0,0210	0,0022	4,7E-21	-0,0097	0,0076	0,1981	instrument
rs6720868	2	230663576	T	C	1,3E-04	49,585	0,0169	0,0024	4,6E-12	-0,0094	0,0078	0,2318	instrument
rs6728037	2	59297346	A	G	2,1E-04	81,000	0,0207	0,0023	6,5E-19	-0,0003	0,0077	0,9664	instrument
rs6739199	2	105452326	T	C	1,0E-04	40,449	0,0159	0,0025	3,2E-10	0,0038	0,0090	0,6737	instrument
rs6870983	5	87697533	T	C	1,5E-04	66,485	-0,0212	0,0026	9,6E-16	0,0057	0,0089	0,5207	instrument
rs6890310	5	27193573	A	G	9,6E-05	37,945	-0,0154	0,0025	1,4E-09	0,0079	0,0087	0,3655	instrument
rs6973700	7	1856669	A	G	1,4E-04	36,376	0,0193	0,0032	1,8E-09	-0,0116	0,0091	0,2038	instrument
rs7024334	9	109072075	T	G	8,7E-05	37,398	0,0159	0,0026	1,6E-09	0,0245	0,0089	0,0058	outlier
rs7084454	10	21821274	A	G	2,1E-04	81,000	0,0225	0,0025	7,4E-20	0,0558	0,0079	1,5E-12	outlier
rs709400	14	104149475	A	G	1,5E-04	64,000	0,0184	0,0023	4,4E-16	-0,0208	0,0076	0,0060	instrument
rs7105462	11	112912048	A	G	9,9E-05	39,198	0,0144	0,0023	4,4E-10	0,0012	0,0075	0,8785	instrument

rs7107409	11	76492195	T	C	1,2E-04	30,941	-0,0178	0,0032	2,1E-08	0,0215	0,0089	0,0154	instrument
rs7124681	11	47529947	A	C	2,9E-04	126,051	0,0247	0,0022	9,3E-29	-0,0102	0,0076	0,1762	instrument
rs7141420	14	79899454	T	C	2,1E-04	91,115	0,0210	0,0022	7,8E-22	0,0121	0,0079	0,1265	instrument
rs7211966	17	54374611	T	C	8,9E-05	34,515	-0,0470	0,0080	4,9E-09	0,0167	0,0332	0,6158	instrument
rs7239114	18	45921214	A	G	1,2E-04	49,638	0,0155	0,0022	3,9E-12	0,0082	0,0079	0,3033	instrument
rs7250833	19	33937277	T	C	8,9E-05	38,543	0,0149	0,0024	7,2E-10	-0,0147	0,0083	0,0770	instrument
rs73124396	7	71579606	T	C	1,2E-04	32,111	0,0187	0,0033	1,1E-08	0,0057	0,0091	0,5300	instrument
rs73213484	4	28489339	A	T	1,7E-04	45,653	0,0250	0,0037	2,3E-11	0,0081	0,0104	0,4378	instrument
rs7335249	13	109962579	T	C	8,7E-05	34,452	0,0135	0,0023	4,9E-09	-0,0065	0,0077	0,4033	instrument
rs7395632	11	958789	T	C	7,4E-05	29,340	-0,0130	0,0024	3,9E-08	0,0030	0,0078	0,7059	instrument
rs7428670	3	131579810	C	G	1,5E-04	60,361	0,0202	0,0026	3,1E-15	-0,0089	0,0085	0,2953	instrument
rs7498044	15	92573639	A	G	1,2E-04	31,640	-0,0180	0,0032	2,7E-08	0,0053	0,0095	0,5752	instrument
rs7498665	16	28883241	A	G	3,3E-04	143,999	-0,0264	0,0022	1,9E-32	-0,0094	0,0076	0,2158	instrument
rs750090	4	152931436	T	C	1,1E-04	42,250	0,0156	0,0024	7,4E-11	-0,0090	0,0080	0,2602	instrument
rs7550711	1	110082886	T	C	2,1E-04	88,858	0,0641	0,0068	3,4E-21	-0,0406	0,0213	0,0565	instrument
rs7571496	2	6169351	A	G	7,9E-05	31,102	0,0145	0,0026	1,4E-08	-0,0101	0,0087	0,2444	instrument
rs7588437	2	181575281	A	G	1,3E-04	58,555	-0,0176	0,0023	8,4E-15	0,0079	0,0078	0,3130	instrument
rs7594237	2	198187164	A	T	8,3E-05	32,718	-0,0143	0,0025	1,8E-08	-0,0222	0,0086	0,0095	outlier
rs7601870	2	15384392	A	G	8,3E-05	32,937	-0,0132	0,0023	1,2E-08	0,0044	0,0075	0,5586	instrument
rs7601895	2	55281901	C	G	1,1E-04	41,473	0,0161	0,0025	9,6E-11	0,0164	0,0084	0,0503	instrument
rs7621025	3	136272246	T	C	1,5E-04	64,000	-0,0200	0,0025	1,6E-15	0,0078	0,0085	0,3606	instrument
rs7647242	3	82642734	T	C	1,0E-04	39,745	0,0145	0,0023	3,9E-10	0,0084	0,0078	0,2830	instrument
rs768023	6	108876002	A	G	1,6E-04	70,712	0,0185	0,0022	1,4E-16	0,0053	0,0076	0,4892	instrument
rs76900466	2	145057760	T	G	1,2E-04	30,654	0,0227	0,0041	4,5E-08	-0,0118	0,0158	0,4562	instrument
rs77432547	13	86494817	A	G	1,4E-04	36,415	-0,0175	0,0029	2,3E-09	0,0105	0,0083	0,2088	instrument
rs7788008	7	112972483	A	G	1,4E-04	55,276	-0,0171	0,0023	1,1E-13	0,0056	0,0075	0,4601	instrument
rs7805441	7	78121458	T	C	1,0E-04	40,295	0,0146	0,0023	2,0E-10	0,0030	0,0074	0,6881	instrument
rs782971	5	43124688	A	G	1,1E-04	47,334	-0,0172	0,0025	4,9E-12	-0,0180	0,0090	0,0457	instrument
rs786420	2	44719893	T	C	8,4E-05	32,956	0,0155	0,0027	8,8E-09	-0,0159	0,0087	0,0668	instrument
rs78886584	1	16859325	A	G	1,3E-04	33,284	-0,0150	0,0026	1,3E-08	-0,0098	0,0083	0,2380	instrument
rs7899106	10	87410904	A	G	1,3E-04	56,250	-0,0375	0,0050	7,6E-14	0,0287	0,0177	0,1036	instrument
rs79113395	1	1590521	A	G	1,9E-04	49,000	-0,0210	0,0030	1,5E-12	-0,0117	0,0119	0,3274	instrument

rs8024806	15	53473990	T	C	8,5E-05	33,492	0,0272	0,0047	9,8E-09	0,0044	0,0156	0,7799	instrument
rs8033510	15	61445514	T	C	8,3E-05	32,585	0,0137	0,0024	1,1E-08	0,0010	0,0077	0,8989	instrument
rs8038522	15	46585105	T	G	9,4E-05	37,051	-0,0140	0,0023	1,3E-09	-0,0022	0,0075	0,7676	instrument
rs8075273	17	61728881	A	C	1,0E-04	44,444	-0,0160	0,0024	5,6E-11	0,0095	0,0083	0,2546	instrument
rs811054	16	72251132	T	C	1,2E-04	54,223	0,0162	0,0022	3,2E-13	0,0216	0,0075	0,0040	outlier
rs836525	7	6465909	T	C	8,2E-05	32,233	0,0176	0,0031	9,8E-09	-0,0097	0,0102	0,3406	instrument
rs879620	16	4015729	T	C	2,9E-04	115,328	0,0247	0,0023	5,4E-26	0,0059	0,0079	0,4540	instrument
rs893006	11	64365796	A	C	1,2E-04	51,960	0,0173	0,0024	4,0E-13	-0,0041	0,0082	0,6210	instrument
rs895330	19	4060707	C	G	9,9E-05	38,955	0,0181	0,0029	6,6E-10	0,0168	0,0100	0,0936	instrument
rs915814	21	46493003	A	G	1,3E-04	34,810	-0,0177	0,0030	5,9E-09	-0,0009	0,0088	0,9195	instrument
rs925018	3	62713143	C	G	8,8E-05	38,117	-0,0142	0,0023	9,8E-10	0,0105	0,0079	0,1844	instrument
rs9304665	19	47602577	A	T	1,4E-04	57,086	0,0204	0,0027	1,8E-14	-0,0202	0,0087	0,0207	instrument
rs9367369	6	13189941	A	G	1,4E-04	35,587	0,0173	0,0029	2,3E-09	-0,0007	0,0081	0,9285	instrument
rs942066	14	94031914	A	G	2,1E-04	55,972	-0,0202	0,0027	1,0E-13	0,0057	0,0077	0,4623	instrument
rs943005	6	50865820	T	C	4,3E-04	188,351	0,0398	0,0029	2,0E-43	0,0223	0,0097	0,0218	outlier
rs9531786	13	85983968	C	G	8,4E-05	33,062	-0,0138	0,0024	8,4E-09	-0,0004	0,0077	0,9574	instrument
rs9571687	13	67472713	A	C	9,2E-05	36,502	-0,0145	0,0024	3,2E-09	-0,0057	0,0081	0,4791	instrument
rs9579775	13	20616557	A	C	1,5E-04	39,690	-0,0252	0,0040	3,7E-10	0,0298	0,0139	0,0322	instrument
rs9595630	13	33065443	T	G	9,2E-05	39,745	0,0145	0,0023	2,0E-10	-0,0159	0,0076	0,0368	instrument
rs973345	9	29713169	T	C	9,7E-05	38,117	0,0142	0,0023	1,1E-09	-0,0003	0,0075	0,9717	instrument
rs9906944	17	47091420	T	C	1,6E-04	62,673	-0,0190	0,0024	7,3E-15	-0,0062	0,0083	0,4567	instrument
rs9926784	16	19941968	T	C	1,9E-04	81,000	0,0252	0,0028	6,0E-20	-0,0040	0,0097	0,6785	instrument
rs9937053	16	53799507	A	G	2,2E-03	941,370	0,0675	0,0022	1,0E-200	-0,0534	0,0074	7,6E-13	outlier
rs994596	4	18459828	T	C	9,1E-05	36,000	0,0150	0,0025	1,0E-09	0,0041	0,0080	0,6109	instrument
rs9955276	18	1839339	T	C	1,2E-04	48,129	0,0222	0,0032	8,0E-12	0,0005	0,0104	0,9621	instrument

Table S8. Single Nucleotide Polymorphisms (SNPs) used as instruments in the Mendelian randomization analyses for the effect of BMI on clear cell ovarian cancer based on the genome-wide significance threshold P = 5E-08. R^2 represents the explained variance in the exposure. SNP-specific instrument strength is given by the respective F-statistic. Some of the genetic variants were identified as outliers in the iterative approach and therefore excluded from the analyses.

SNP	CHR	POS	EA	OA	R^2	F-stat	β_{BMI}	SE_{BMI}	P_{BMI}	$\beta_{Clear\ cell}$	$SE_{Clear\ cell}$	$P_{Clear\ cell}$	$variant_{Clear\ cell}$
rs10002111	4	67815504	A	G	9,2E-05	36,430	0,0169	0,0028	9,3E-10	-0,0452	0,0484	0,3499	instrument
rs1003081	11	118913993	T	C	9,9E-05	42,843	0,0144	0,0022	6,3E-11	-0,0316	0,0410	0,4406	instrument
rs10048652	2	35449306	T	C	8,7E-05	34,452	0,0135	0,0023	3,5E-09	-0,0165	0,0404	0,6829	instrument
rs1009473	9	138777312	T	C	7,7E-05	30,489	-0,0127	0,0023	3,4E-08	-0,0746	0,0404	0,0644	instrument
rs10099330	8	143383694	A	G	8,3E-05	32,440	-0,0131	0,0023	9,9E-09	0,0366	0,0404	0,3650	instrument
rs10116186	9	80532374	A	G	1,2E-04	31,102	-0,0145	0,0026	4,1E-08	-0,0140	0,0406	0,7308	instrument
rs10118701	9	103061366	A	G	1,4E-04	53,778	-0,0176	0,0024	2,2E-13	-0,0292	0,0422	0,4889	instrument
rs10124645	9	11321119	A	G	8,9E-05	34,964	0,0136	0,0023	5,0E-09	0,0206	0,0417	0,6217	instrument
rs10182181	2	25150296	A	G	6,2E-04	270,751	-0,0362	0,0022	5,0E-63	0,0222	0,0411	0,5891	instrument
rs10237317	7	70045941	A	G	9,1E-05	39,347	-0,0138	0,0022	6,8E-10	0,0293	0,0418	0,4838	instrument
rs10271582	7	99121950	A	G	1,2E-04	32,222	0,0193	0,0034	8,4E-09	-0,0080	0,0556	0,8862	instrument
rs10471636	5	63979856	A	G	1,3E-04	50,173	-0,0170	0,0024	4,7E-13	0,0751	0,0420	0,0736	instrument
rs1048932	11	115044850	A	C	1,6E-04	68,438	-0,0182	0,0022	2,5E-16	0,0042	0,0409	0,9181	instrument
rs10499694	7	50614173	A	G	8,8E-05	38,215	0,0136	0,0022	4,0E-10	0,0393	0,0400	0,3266	instrument
rs10513801	3	185822353	T	G	2,8E-04	120,999	0,0352	0,0032	1,3E-27	-0,0829	0,0593	0,1619	instrument
rs10733682	9	129460914	A	G	1,2E-04	51,578	0,0158	0,0022	1,9E-12	0,0226	0,0404	0,5761	instrument
rs10742752	11	45438374	T	C	8,3E-05	36,000	-0,0132	0,0022	4,0E-09	-0,0174	0,0415	0,6750	instrument
rs10756714	9	15885041	A	G	2,1E-04	81,784	0,0208	0,0023	1,5E-19	0,0441	0,0404	0,2743	instrument
rs1075901	17	15943910	T	C	9,9E-05	42,843	-0,0144	0,0022	4,4E-11	0,0119	0,0405	0,7686	instrument
rs10761785	10	65318766	T	G	1,4E-04	61,124	-0,0172	0,0022	4,2E-15	0,0120	0,0400	0,7654	instrument
rs10797115	9	92191256	T	C	1,2E-04	48,393	0,0160	0,0023	4,1E-12	0,0468	0,0403	0,2451	instrument
rs10823893	10	53677313	A	G	1,6E-04	42,009	0,0175	0,0027	5,2E-11	-0,0055	0,0411	0,8938	instrument
rs10824347	10	77645229	A	T	1,1E-04	42,491	0,0176	0,0027	5,5E-11	0,0885	0,0480	0,0652	instrument
rs10909880	1	2727804	T	C	7,5E-05	31,768	-0,0124	0,0022	2,0E-08	-0,0506	0,0410	0,2173	instrument
rs10920678	1	190239907	A	G	1,3E-04	54,894	0,0163	0,0022	7,5E-14	0,0563	0,0410	0,1689	instrument
rs10938397	4	45182527	A	G	4,7E-04	205,009	-0,0315	0,0022	1,7E-45	-0,0144	0,0404	0,7212	instrument
rs10962550	9	16720329	C	G	1,0E-04	40,960	0,0192	0,0030	1,7E-10	-0,0359	0,0545	0,5091	instrument
rs10992867	9	96461013	A	G	1,3E-04	50,628	0,0185	0,0026	8,9E-13	-0,1242	0,0469	0,0081	outlier

rs11022762	11	13335926	T	C	1,0E-04	41,173	-0,0154	0,0024	5,6E-11	-0,0049	0,0411	0,9053	instrument
rs11073383	15	95274349	A	G	1,2E-04	47,790	-0,0159	0,0023	3,3E-12	0,0063	0,0402	0,8761	instrument
rs11165643	1	96924097	T	C	1,6E-04	67,688	0,0181	0,0022	1,7E-16	-0,0231	0,0412	0,5750	instrument
rs112646560	1	39560250	T	C	1,4E-04	37,515	0,0196	0,0032	1,1E-09	0,0007	0,0503	0,9894	instrument
rs11667280	19	30705747	C	G	7,5E-05	29,684	0,0158	0,0029	3,1E-08	-0,0573	0,0513	0,2641	instrument
rs11672660	19	46180184	T	C	3,1E-04	131,429	-0,0321	0,0028	5,9E-31	-0,0244	0,0512	0,6330	instrument
rs11677607	2	100751150	T	C	2,0E-04	79,012	-0,0240	0,0027	2,9E-19	-0,0932	0,0475	0,0499	instrument
rs1167800	7	75176196	A	G	2,0E-04	82,644	0,0200	0,0022	3,7E-19	0,0227	0,0410	0,5805	instrument
rs11692326	2	208263279	T	C	8,3E-05	36,000	0,0156	0,0026	2,0E-09	0,0176	0,0482	0,7146	instrument
rs11727273	4	55509748	T	C	1,6E-04	42,250	-0,0169	0,0026	1,3E-10	-0,0191	0,0411	0,6426	instrument
rs11751591	6	33794215	A	G	8,5E-05	33,423	-0,0185	0,0032	1,2E-08	0,0278	0,0563	0,6207	instrument
rs11754747	6	141494602	T	C	7,3E-05	31,532	0,0146	0,0026	1,3E-08	-0,0090	0,0488	0,8545	instrument
rs11880870	19	18830704	A	G	1,8E-04	73,363	0,0197	0,0023	3,3E-18	0,0205	0,0409	0,6165	instrument
rs11945861	4	65700865	A	G	7,8E-05	30,864	-0,0150	0,0027	3,1E-08	-0,0562	0,0484	0,2432	instrument
rs11971098	7	26699061	A	G	8,5E-05	33,414	-0,0237	0,0041	6,5E-09	0,1223	0,0736	0,0965	instrument
rs12042959	1	243533273	A	G	9,3E-05	36,754	0,0194	0,0032	2,3E-09	-0,0303	0,0566	0,5925	instrument
rs12089815	1	91189933	A	G	1,3E-04	34,628	-0,0153	0,0026	6,0E-09	0,0054	0,0412	0,8960	instrument
rs12101625	15	26972431	C	G	8,4E-05	33,062	0,0138	0,0024	5,5E-09	-0,0274	0,0416	0,5096	instrument
rs12140153	1	62579891	T	G	1,8E-04	48,110	-0,0326	0,0047	2,9E-12	0,0035	0,0734	0,9621	instrument
rs12150665	17	34914787	T	C	1,3E-04	57,622	0,0167	0,0022	5,9E-14	-0,0029	0,0420	0,9444	instrument
rs12202969	6	98576223	A	G	1,0E-04	39,745	-0,0145	0,0023	1,9E-10	0,0160	0,0405	0,6924	instrument
rs12207125	6	154301575	A	G	1,3E-04	34,468	0,0182	0,0031	7,7E-09	-0,0021	0,0492	0,9660	instrument
rs12364470	11	134601012	T	G	1,0E-04	44,444	-0,0200	0,0030	2,5E-11	-0,0209	0,0590	0,7229	instrument
rs12429545	13	54102206	A	G	1,8E-04	77,760	0,0291	0,0033	1,1E-18	-0,0212	0,0622	0,7331	instrument
rs12448257	16	3599655	A	G	7,2E-05	30,864	0,0150	0,0027	3,1E-08	0,0325	0,0502	0,5174	instrument
rs12462975	19	30272202	A	G	1,7E-04	65,934	0,0203	0,0025	2,6E-16	-0,0796	0,0431	0,0647	instrument
rs12564992	1	174478100	A	G	8,0E-05	34,979	-0,0207	0,0035	2,4E-09	0,0800	0,0646	0,2156	instrument
rs12593036	15	81058652	A	G	9,6E-05	37,945	0,0154	0,0025	6,6E-10	0,0669	0,0448	0,1352	instrument
rs12612009	2	228982603	T	G	8,7E-05	34,515	-0,0141	0,0024	3,8E-09	-0,0322	0,0429	0,4529	instrument
rs12645001	4	96018337	A	G	8,7E-05	34,515	-0,0141	0,0024	4,1E-09	0,0462	0,0419	0,2708	instrument
rs12680842	8	95582606	A	G	6,8E-05	29,340	0,0130	0,0024	3,4E-08	-0,0333	0,0427	0,4349	instrument
rs12681792	8	62054463	A	C	1,0E-04	39,820	0,0183	0,0029	2,6E-10	-0,0056	0,0524	0,9154	instrument

rs12692596	2	161265910	T	C	9,3E-05	40,295	0,0146	0,0023	9,7E-11	0,0242	0,0416	0,5604	instrument
rs12692738	2	165558252	T	C	8,9E-05	38,822	-0,0162	0,0026	2,8E-10	0,0328	0,0478	0,4933	instrument
rs12714199	2	86812549	T	C	1,0E-04	40,295	-0,0146	0,0023	5,1E-10	0,0277	0,0417	0,5068	instrument
rs1285997	14	91513029	C	G	9,2E-05	36,000	-0,0150	0,0025	3,2E-09	-0,0333	0,0448	0,4574	instrument
rs12872889	13	28674628	T	C	1,7E-04	45,453	-0,0209	0,0031	1,5E-11	-0,0189	0,0478	0,6929	instrument
rs1289736	3	107950892	T	C	9,3E-05	36,523	0,0139	0,0023	1,4E-09	0,0183	0,0405	0,6523	instrument
rs12914489	15	74187937	A	G	9,1E-05	39,760	0,0227	0,0036	1,6E-10	0,0206	0,0642	0,7484	instrument
rs12949279	17	78558411	T	C	1,5E-04	66,200	0,0179	0,0022	6,1E-16	-0,0103	0,0410	0,8027	instrument
rs12981256	19	1865901	A	G	1,6E-04	62,616	0,0182	0,0023	3,9E-15	-0,0502	0,0410	0,2212	instrument
rs13002158	2	144021712	A	G	1,3E-04	51,443	-0,0208	0,0029	1,2E-12	-0,0465	0,0528	0,3789	instrument
rs13053342	22	48871624	A	G	1,0E-04	40,640	0,0153	0,0024	1,3E-10	-0,0873	0,0454	0,0540	instrument
rs13062093	3	35667057	T	G	8,3E-05	32,585	-0,0137	0,0024	8,0E-09	-0,0265	0,0415	0,5225	instrument
rs13068138	3	85809425	T	C	1,7E-04	73,197	0,0231	0,0027	3,6E-17	0,0271	0,0503	0,5897	instrument
rs13076052	3	44456573	C	G	1,2E-04	32,766	-0,0166	0,0029	1,3E-08	0,0153	0,0458	0,7391	instrument
rs13107325	4	103188709	T	C	2,4E-04	105,528	0,0452	0,0044	9,3E-25	-0,1122	0,0798	0,1598	instrument
rs13110266	4	162129844	A	G	9,6E-05	41,661	-0,0142	0,0022	1,5E-10	0,0084	0,0407	0,8363	instrument
rs13174863	5	139080745	A	G	1,8E-04	75,858	-0,0270	0,0031	4,5E-18	0,0300	0,0596	0,6142	instrument
rs13209872	6	97753223	C	G	8,9E-05	35,007	-0,0142	0,0024	6,1E-09	0,0255	0,0430	0,5537	instrument
rs13213408	6	162998049	A	T	9,0E-05	35,637	0,0197	0,0033	4,3E-09	0,0051	0,0589	0,9305	instrument
rs1362910	8	30856464	A	G	8,2E-05	33,943	0,0134	0,0023	3,0E-09	0,0257	0,0407	0,5281	instrument
rs1403846	4	119101723	T	C	7,2E-05	31,206	0,0162	0,0029	1,6E-08	-0,0591	0,0517	0,2531	instrument
rs1409818	20	21381121	T	C	1,2E-04	46,387	0,0252	0,0037	1,6E-11	-0,0629	0,0672	0,3499	instrument
rs141729694	5	87999371	T	C	2,2E-04	57,284	0,0386	0,0051	2,5E-14	-0,0520	0,0776	0,5026	instrument
rs1417665	6	104816944	T	C	1,2E-04	47,088	0,0199	0,0029	5,7E-12	-0,0447	0,0512	0,3824	instrument
rs1421598	2	50264710	A	C	9,7E-05	38,360	-0,0192	0,0031	6,8E-10	0,1094	0,0559	0,0503	instrument
rs1436348	3	104612668	A	G	8,6E-05	33,943	-0,0134	0,0023	6,3E-09	-0,0231	0,0411	0,5738	instrument
rs1441264	13	79580919	A	G	1,3E-04	55,276	0,0171	0,0023	5,2E-14	-0,0413	0,0417	0,3214	instrument
rs1452075	3	62481063	T	C	9,4E-05	40,449	0,0159	0,0025	1,5E-10	0,0088	0,0456	0,8465	instrument
rs1491333	4	172186288	T	G	8,1E-05	31,966	-0,0147	0,0026	9,9E-09	0,0447	0,0467	0,3384	instrument
rs1492014	3	94071481	T	C	1,0E-04	39,745	-0,0145	0,0023	2,8E-10	0,0515	0,0405	0,2033	instrument
rs1518159	18	40760146	T	C	1,3E-04	52,841	-0,0189	0,0026	6,7E-13	0,0195	0,0462	0,6724	instrument
rs1561589	10	126695673	A	G	1,1E-04	47,191	0,0158	0,0023	4,2E-12	0,0430	0,0415	0,3003	instrument

rs1569979	14	29681294	A	G	1,8E-04	72,565	0,0230	0,0027	1,6E-17	-0,0494	0,0471	0,2944	instrument
rs1579557	6	40371918	T	C	1,8E-04	71,910	0,0212	0,0025	3,1E-17	0,0203	0,0450	0,6516	instrument
rs158186	5	107440856	A	G	2,2E-04	85,711	-0,0287	0,0031	3,2E-20	0,0452	0,0527	0,3911	instrument
rs159032	5	94206202	T	C	7,8E-05	30,454	0,0149	0,0027	4,5E-08	0,0873	0,0459	0,0570	instrument
rs1681743	10	118565501	T	C	7,5E-05	29,793	-0,0131	0,0024	3,7E-08	-0,0147	0,0413	0,7216	instrument
rs16851483	3	141275436	T	G	1,6E-04	62,272	0,0363	0,0046	2,3E-15	-0,0289	0,0827	0,7262	instrument
rs16912921	9	28413461	A	C	2,7E-04	117,204	0,0249	0,0023	1,6E-26	0,0490	0,0430	0,2547	instrument
rs16975921	18	39914870	A	T	1,1E-04	42,510	-0,0163	0,0025	3,9E-11	0,0251	0,0427	0,5563	instrument
rs16989232	20	39291784	A	G	7,1E-05	30,972	0,0128	0,0023	1,5E-08	0,0077	0,0422	0,8544	instrument
rs17014375	1	209543560	T	G	7,9E-05	31,228	-0,0190	0,0034	1,5E-08	0,1297	0,0605	0,0320	instrument
rs17066856	18	58049656	T	C	2,7E-04	115,707	0,0398	0,0037	1,4E-27	-0,0107	0,0692	0,8769	instrument
rs17405819	8	76806584	T	C	1,9E-04	82,507	0,0218	0,0024	7,8E-20	0,0015	0,0434	0,9721	instrument
rs1750307	1	156488420	A	T	8,3E-05	32,585	0,0137	0,0024	8,2E-09	0,0577	0,0416	0,1654	instrument
rs17522122	14	33302882	T	G	1,3E-04	57,622	0,0167	0,0022	6,5E-14	-0,0129	0,0408	0,7513	instrument
rs17619973	3	114417675	A	G	7,4E-05	31,673	0,0242	0,0043	1,3E-08	-0,1015	0,0805	0,2073	instrument
rs17724992	19	18454825	A	G	1,1E-04	46,240	0,0170	0,0025	6,7E-12	-0,0608	0,0452	0,1779	instrument
rs17751061	19	19413092	T	C	8,2E-05	31,993	-0,0181	0,0032	1,3E-08	0,0490	0,0546	0,3688	instrument
rs17767765	16	24539793	T	C	7,7E-05	30,489	-0,0127	0,0023	2,6E-08	-0,0300	0,0413	0,4680	instrument
rs17806224	20	51065854	A	G	2,3E-04	92,801	-0,0289	0,0030	3,5E-22	0,0536	0,0526	0,3083	instrument
rs1799923	3	42306294	A	G	1,2E-04	48,203	-0,0243	0,0035	5,1E-12	-0,0516	0,0672	0,4423	instrument
rs1801282	3	12393125	C	G	7,0E-05	30,417	-0,0182	0,0033	4,7E-08	0,0344	0,0614	0,5751	instrument
rs1808579	18	21104888	T	C	1,9E-04	81,000	-0,0198	0,0022	9,4E-20	-0,0122	0,0404	0,7632	instrument
rs1808629	8	73435964	A	G	2,5E-04	65,147	-0,0226	0,0028	5,8E-16	-0,0227	0,0435	0,6024	instrument
rs1812587	5	62991802	T	G	1,3E-04	34,177	0,0152	0,0026	7,0E-09	0,0475	0,0406	0,2421	instrument
rs185350	19	34306816	T	C	7,8E-05	33,851	0,0128	0,0022	7,1E-09	-0,0101	0,0401	0,8003	instrument
rs1861412	2	58893065	A	G	2,2E-04	87,381	-0,0215	0,0023	9,1E-21	0,0552	0,0404	0,1718	instrument
rs1884389	20	1410582	T	C	9,4E-05	37,051	-0,0140	0,0023	1,2E-09	-0,0254	0,0405	0,5302	instrument
rs1884897	20	6612832	A	G	1,2E-04	53,990	-0,0169	0,0023	9,1E-14	-0,0232	0,0420	0,5808	instrument
rs1916801	3	61187046	A	T	1,4E-04	59,710	0,0170	0,0022	1,8E-14	-0,1320	0,0410	0,0013	outlier
rs1927790	13	96922191	T	C	8,7E-05	37,655	-0,0135	0,0022	1,3E-09	-0,0940	0,0409	0,0214	instrument
rs1928295	9	120378483	T	C	1,2E-04	52,892	0,0160	0,0022	2,8E-13	-0,0293	0,0402	0,4662	instrument
rs1949197	3	86273850	A	G	9,2E-05	36,523	0,0139	0,0023	3,0E-09	-0,0261	0,0412	0,5265	instrument

rs197374	1	112289983	T	C	1,4E-04	55,276	0,0171	0,0023	2,8E-13	0,0270	0,0412	0,5127	instrument
rs1984559	2	135678888	A	G	7,9E-05	29,521	0,0163	0,0030	3,3E-08	0,0566	0,0493	0,2505	instrument
rs198665	6	143154886	A	C	7,8E-05	33,943	0,0134	0,0023	4,4E-09	-0,0046	0,0423	0,9137	instrument
rs2012502	16	81728081	A	C	1,0E-04	44,251	0,0153	0,0023	3,0E-11	-0,0169	0,0420	0,6881	instrument
rs2045293	7	114350102	T	C	1,7E-04	67,525	0,0189	0,0023	4,1E-16	-0,0981	0,0412	0,0174	instrument
rs2053682	5	170599327	A	C	1,4E-04	53,582	0,0183	0,0025	1,9E-13	0,0081	0,0431	0,8511	instrument
rs209416	6	83534729	A	T	1,2E-04	47,840	0,0166	0,0024	8,7E-12	-0,0682	0,0437	0,1183	instrument
rs2112347	5	75015242	T	G	4,2E-04	180,493	0,0309	0,0023	2,1E-42	-0,0774	0,0415	0,0620	instrument
rs214249	16	348687	T	G	1,2E-04	48,418	0,0167	0,0024	1,4E-12	0,0042	0,0407	0,9180	instrument
rs2161097	5	103945178	T	C	8,0E-05	31,457	0,0129	0,0023	2,3E-08	-0,0120	0,0402	0,7646	instrument
rs2178899	6	31606756	A	T	1,3E-04	56,477	0,0248	0,0033	7,2E-14	0,1046	0,0630	0,0966	instrument
rs2185027	6	153381622	A	C	9,5E-05	41,173	-0,0154	0,0024	1,1E-10	-0,0666	0,0433	0,1255	instrument
rs2228213	6	12124855	A	G	9,5E-05	41,406	-0,0148	0,0023	9,2E-11	-0,0524	0,0421	0,2131	instrument
rs2267812	7	74138121	A	C	9,2E-05	35,177	-0,0172	0,0029	1,7E-09	-0,0052	0,0490	0,9149	instrument
rs2267958	9	131015279	A	G	1,2E-04	45,002	-0,0161	0,0024	1,9E-11	0,0008	0,0424	0,9851	instrument
rs2271046	4	52752812	A	T	7,6E-05	30,030	-0,0137	0,0025	2,4E-08	0,0109	0,0449	0,8082	instrument
rs2289292	16	30097630	T	C	1,5E-04	57,760	-0,0190	0,0025	1,1E-14	-0,0124	0,0446	0,7813	instrument
rs2289705	7	121983843	A	G	7,7E-05	33,284	-0,0150	0,0026	1,5E-08	0,0447	0,0501	0,3722	instrument
rs2299383	7	103418846	T	C	1,6E-04	68,438	0,0182	0,0022	2,7E-16	-0,0668	0,0409	0,1022	instrument
rs2357760	6	120213880	A	G	1,1E-04	47,790	0,0159	0,0023	1,1E-11	-0,0300	0,0426	0,4810	instrument
rs2465043	6	51180765	A	G	8,9E-05	35,007	-0,0142	0,0024	3,2E-09	-0,0400	0,0426	0,3467	instrument
rs2470520	3	15762920	T	C	7,6E-05	30,011	-0,0126	0,0023	4,2E-08	-0,0613	0,0407	0,1319	instrument
rs247975	3	173107443	T	C	1,6E-04	62,616	-0,0182	0,0023	3,6E-15	0,0194	0,0419	0,6433	instrument
rs2479958	13	111984244	A	G	8,1E-05	31,640	0,0135	0,0024	1,4E-08	-0,0185	0,0411	0,6533	instrument
rs2528531	7	93236510	A	C	9,3E-05	36,502	-0,0145	0,0024	1,3E-09	0,0581	0,0421	0,1682	instrument
rs2568958	1	72765116	A	G	2,5E-04	106,464	0,0227	0,0022	1,3E-24	0,0435	0,0415	0,2946	instrument
rs2605603	11	93221105	A	G	8,3E-05	36,000	-0,0132	0,0022	1,4E-09	-0,0198	0,0403	0,6221	instrument
rs2619976	17	71754545	T	C	9,4E-05	37,007	0,0146	0,0024	6,2E-10	-0,0196	0,0426	0,6446	instrument
rs2721965	8	116662038	A	C	1,5E-04	60,062	0,0186	0,0024	2,1E-14	-0,0208	0,0426	0,6257	instrument
rs273504	19	18215247	A	G	1,1E-04	43,102	-0,0151	0,0023	1,3E-10	-0,0139	0,0408	0,7343	instrument
rs2754079	14	25886140	A	G	1,2E-04	46,004	-0,0156	0,0023	2,3E-11	0,0169	0,0415	0,6828	instrument
rs2814992	6	34617144	A	G	3,6E-04	155,706	-0,0287	0,0023	1,2E-35	0,0384	0,0426	0,3664	instrument

rs2820295	1	201800868	A	G	2,0E-04	79,507	0,0214	0,0024	8,0E-19	0,0542	0,0421	0,1985	instrument
rs2836754	21	40291740	T	C	1,1E-04	45,870	-0,0149	0,0022	3,7E-11	-0,0265	0,0415	0,5232	instrument
rs28489620	22	41804716	A	G	1,5E-04	38,955	-0,0181	0,0029	6,9E-10	-0,0469	0,0446	0,2931	instrument
rs2861685	2	67837553	T	C	1,8E-04	47,929	0,0180	0,0026	1,1E-11	-0,0397	0,0406	0,3283	instrument
rs2862961	11	43656535	A	G	2,3E-04	91,393	-0,0239	0,0025	5,3E-22	-0,0427	0,0432	0,3228	instrument
rs2959592	8	17002725	T	C	8,7E-05	36,862	0,0170	0,0028	6,3E-10	0,0451	0,0533	0,3977	instrument
rs2968864	7	150622162	T	C	9,2E-05	36,446	0,0163	0,0027	9,3E-10	-0,0221	0,0466	0,6352	instrument
rs2984618	1	47690438	T	G	1,3E-04	57,622	0,0167	0,0022	2,2E-14	-0,0085	0,0410	0,8352	instrument
rs329651	11	133767622	T	G	9,7E-05	41,787	0,0181	0,0028	2,0E-10	-0,0589	0,0516	0,2534	instrument
rs337637	4	38604470	A	G	6,8E-05	29,537	-0,0125	0,0023	3,0E-08	0,0519	0,0419	0,2155	instrument
rs34517439	1	78450517	A	C	3,4E-04	90,481	0,0390	0,0041	2,0E-21	-0,0774	0,0654	0,2362	instrument
rs34811474	4	25408838	A	G	4,0E-04	104,566	-0,0317	0,0031	5,2E-24	0,0837	0,0546	0,1253	instrument
rs34930419	10	33987329	T	C	1,4E-04	36,316	-0,0458	0,0076	1,4E-09	-0,1734	0,1149	0,1314	instrument
rs35107973	4	143125948	T	C	1,2E-04	32,573	0,0234	0,0041	7,7E-09	0,1151	0,0625	0,0652	instrument
rs355754	3	154024165	T	C	9,2E-05	38,117	-0,0142	0,0023	4,3E-10	0,0025	0,0412	0,9518	instrument
rs35722922	2	47002226	A	G	1,8E-04	48,482	0,0188	0,0027	3,6E-12	0,0304	0,0420	0,4692	instrument
rs3803286	14	103246470	A	G	1,3E-04	51,361	0,0172	0,0024	8,3E-13	0,0203	0,0424	0,6313	instrument
rs3806114	6	20482335	A	G	8,0E-05	34,515	-0,0141	0,0024	4,5E-09	-0,0684	0,0431	0,1124	instrument
rs3826408	17	7101292	T	C	7,0E-05	30,250	0,0121	0,0022	4,5E-08	0,0596	0,0407	0,1435	instrument
rs3829849	9	129390800	T	C	8,0E-05	34,964	0,0136	0,0023	1,9E-09	-0,0318	0,0419	0,4477	instrument
rs3977755	10	104420210	T	C	8,0E-05	32,718	-0,0143	0,0025	8,5E-09	-0,0362	0,0446	0,4168	instrument
rs4055791	13	59266053	T	C	1,5E-04	39,643	-0,0170	0,0027	1,8E-10	0,0027	0,0410	0,9473	instrument
rs4148155	4	89054667	A	G	7,7E-05	33,640	0,0203	0,0035	6,2E-09	0,0941	0,0660	0,1536	instrument
rs4240673	8	10787612	T	C	1,5E-04	64,000	0,0176	0,0022	1,0E-15	-0,0147	0,0537	0,7841	instrument
rs427943	21	46570896	A	C	1,5E-04	59,223	-0,0177	0,0023	1,4E-14	0,0813	0,0410	0,0475	instrument
rs429358	19	45411941	T	C	2,2E-04	58,777	0,0276	0,0036	3,2E-14	0,0314	0,0582	0,5895	instrument
rs4295917	1	32173360	A	G	8,2E-05	31,640	0,0135	0,0024	1,6E-08	-0,0422	0,0430	0,3264	instrument
rs4307239	7	24354300	A	G	9,5E-05	37,582	-0,0141	0,0023	1,3E-09	0,0268	0,0410	0,5137	instrument
rs4320543	8	34422967	A	G	8,0E-05	31,457	-0,0129	0,0023	2,9E-08	-0,0042	0,0420	0,9200	instrument
rs4421623	1	98316285	T	G	1,2E-04	46,441	0,0184	0,0027	1,1E-11	0,0172	0,0481	0,7208	instrument
rs4459316	11	130756176	T	C	1,1E-04	44,832	-0,0154	0,0023	2,3E-11	-0,0250	0,0404	0,5371	instrument
rs4474229	1	195037001	A	G	1,6E-04	41,055	-0,0173	0,0027	1,6E-10	0,0066	0,0412	0,8735	instrument

rs4482463	2	205375909	A	C	2,0E-04	75,524	-0,0365	0,0042	5,2E-18	0,0674	0,0765	0,3782	instrument
rs4569924	5	153540025	T	C	1,2E-04	51,578	0,0158	0,0022	7,4E-13	0,0187	0,0407	0,6457	instrument
rs4586287	13	99245334	C	G	7,7E-05	30,470	0,0138	0,0025	2,9E-08	-0,0899	0,0434	0,0385	instrument
rs4673553	2	211608379	T	G	1,1E-04	44,251	-0,0153	0,0023	2,6E-11	-0,0315	0,0404	0,4360	instrument
rs4677812	3	194863794	A	C	8,4E-05	33,177	-0,0144	0,0025	9,4E-09	-0,0057	0,0451	0,8987	instrument
rs4776982	15	68114974	A	G	3,3E-04	143,078	0,0311	0,0026	4,2E-33	0,0488	0,0487	0,3159	instrument
rs4783718	16	69547741	T	C	1,8E-04	78,669	0,0204	0,0023	1,8E-19	-0,0260	0,0423	0,5385	instrument
rs4790292	17	1824305	A	C	1,7E-04	63,516	-0,0263	0,0033	1,7E-15	0,0986	0,0589	0,0940	instrument
rs4834272	4	113313986	T	C	8,9E-05	38,656	-0,0143	0,0023	8,2E-10	0,0211	0,0437	0,6291	instrument
rs4858223	3	20581149	T	C	1,1E-04	41,473	-0,0161	0,0025	8,3E-11	-0,0470	0,0435	0,2803	instrument
rs4864201	4	130731284	T	C	7,5E-05	32,440	0,0131	0,0023	6,3E-09	0,0286	0,0419	0,4943	instrument
rs4880341	10	133992689	T	C	9,8E-05	38,656	-0,0143	0,0023	4,5E-10	-0,0050	0,0405	0,9012	instrument
rs4889606	16	31011183	A	G	3,1E-04	135,773	0,0268	0,0023	1,3E-32	-0,0267	0,0417	0,5226	instrument
rs4929923	11	8639200	T	C	2,1E-04	92,327	-0,0221	0,0023	2,5E-22	0,0060	0,0418	0,8852	instrument
rs4936175	11	132641959	T	C	1,2E-04	47,191	-0,0158	0,0023	6,7E-12	-0,0004	0,0404	0,9915	instrument
rs4969387	17	79081724	C	G	8,4E-05	32,532	-0,0154	0,0027	6,9E-09	-0,0061	0,0460	0,8950	instrument
rs4986044	17	21261560	T	C	1,4E-04	61,836	-0,0173	0,0022	2,2E-15	-0,0554	0,0401	0,1677	instrument
rs543874	1	177889480	A	G	1,0E-03	455,109	-0,0576	0,0027	8,7E-100	0,0565	0,0514	0,2717	instrument
rs56161855	17	46288649	A	T	1,7E-04	44,787	-0,0261	0,0039	1,5E-11	-0,0169	0,0582	0,7721	instrument
rs563132	9	37245476	A	T	1,4E-04	56,250	0,0180	0,0024	2,0E-14	0,0133	0,0412	0,7473	instrument
rs563296	10	99772404	A	G	1,4E-04	53,168	0,0175	0,0024	8,8E-14	0,0805	0,0408	0,0484	instrument
rs57221746	15	99230035	A	G	1,2E-04	30,594	-0,0177	0,0032	2,1E-08	-0,0007	0,0482	0,9884	instrument
rs57800857	4	140863365	A	C	1,8E-04	47,456	0,0186	0,0027	1,1E-11	-0,0135	0,0415	0,7447	instrument
rs6010784	20	61540319	T	C	7,6E-05	32,802	0,0126	0,0022	1,1E-08	-0,0267	0,0403	0,5071	instrument
rs6050446	20	25195509	A	G	8,0E-05	33,785	-0,0372	0,0064	6,2E-09	0,0073	0,1225	0,9522	instrument
rs6142096	20	32686658	A	G	8,6E-05	33,943	0,0134	0,0023	4,2E-09	0,0126	0,0400	0,7533	instrument
rs61813324	1	156049877	T	C	2,2E-04	58,385	0,0298	0,0039	3,6E-14	0,0826	0,0638	0,1953	instrument
rs62007782	15	78029797	A	G	1,4E-04	35,601	-0,0179	0,0030	1,9E-09	-0,0411	0,0464	0,3751	instrument
rs62106258	2	417167	T	C	9,7E-04	254,452	0,0989	0,0062	8,7E-58	0,1763	0,1121	0,1157	instrument
rs62412826	6	61881167	T	C	1,3E-04	33,518	0,0330	0,0057	8,0E-09	-0,0232	0,0904	0,7975	instrument
rs625652	11	65641931	A	T	2,0E-04	52,160	-0,0195	0,0027	9,1E-13	0,0031	0,0419	0,9414	instrument
rs6265	11	27679916	T	C	4,0E-04	172,734	-0,0368	0,0028	8,0E-40	-0,0125	0,0512	0,8077	instrument

rs6446187	3	49907111	A	C	4,3E-04	114,325	0,0278	0,0026	1,2E-26	0,0094	0,0400	0,8146	instrument
rs6493498	15	51754451	T	C	1,3E-04	54,894	0,0163	0,0022	1,5E-13	0,0505	0,0405	0,2127	instrument
rs6548237	2	621461	A	C	9,1E-04	394,500	-0,0576	0,0029	4,4E-89	-0,0722	0,0674	0,2843	instrument
rs6556810	5	164585949	T	C	7,4E-05	29,066	-0,0124	0,0023	5,0E-08	0,0052	0,0409	0,8983	instrument
rs6561937	13	58257667	A	T	1,4E-04	53,236	-0,0197	0,0027	6,0E-13	-0,0238	0,0478	0,6188	instrument
rs6567160	18	57829135	T	C	1,1E-03	475,573	-0,0567	0,0026	8,7E-108	-0,0242	0,0469	0,6049	instrument
rs657452	1	49589847	A	G	2,0E-04	85,142	0,0203	0,0022	1,1E-19	-0,0544	0,0410	0,1843	instrument
rs6585198	10	114762237	A	G	1,1E-04	49,000	-0,0154	0,0022	1,2E-12	0,0382	0,0399	0,3374	instrument
rs6669189	1	75011358	T	C	2,1E-04	91,115	0,0210	0,0022	4,7E-21	0,0333	0,0408	0,4135	instrument
rs6720868	2	230663576	T	C	1,3E-04	49,585	0,0169	0,0024	4,6E-12	0,0062	0,0425	0,8848	instrument
rs6728037	2	59297346	A	G	2,1E-04	81,000	0,0207	0,0023	6,5E-19	-0,0153	0,0414	0,7116	instrument
rs6739199	2	105452326	T	C	1,0E-04	40,449	0,0159	0,0025	3,2E-10	-0,0190	0,0461	0,6804	instrument
rs6870983	5	87697533	T	C	1,5E-04	66,485	-0,0212	0,0026	9,6E-16	-0,0042	0,0479	0,9303	instrument
rs6890310	5	27193573	A	G	9,6E-05	37,945	-0,0154	0,0025	1,4E-09	0,0649	0,0453	0,1521	instrument
rs6973700	7	1856669	A	G	1,4E-04	36,376	0,0193	0,0032	1,8E-09	-0,0278	0,0490	0,5699	instrument
rs7024334	9	109072075	T	G	8,7E-05	37,398	0,0159	0,0026	1,6E-09	-0,0705	0,0488	0,1485	instrument
rs7084454	10	21821274	A	G	2,1E-04	81,000	0,0225	0,0025	7,4E-20	0,1097	0,0418	0,0090	outlier
rs709400	14	104149475	A	G	1,5E-04	64,000	0,0184	0,0023	4,4E-16	-0,0159	0,0412	0,6987	instrument
rs7105462	11	112912048	A	G	9,9E-05	39,198	0,0144	0,0023	4,4E-10	-0,0217	0,0409	0,5949	instrument
rs7107409	11	76492195	T	C	1,2E-04	30,941	-0,0178	0,0032	2,1E-08	0,0420	0,0483	0,3844	instrument
rs7124681	11	47529947	A	C	2,9E-04	126,051	0,0247	0,0022	9,3E-29	0,0089	0,0408	0,8269	instrument
rs7141420	14	79899454	T	C	2,1E-04	91,115	0,0210	0,0022	7,8E-22	-0,0725	0,0411	0,0780	instrument
rs7211966	17	54374611	T	C	8,9E-05	34,515	-0,0470	0,0080	4,9E-09	-0,0059	0,1731	0,9726	instrument
rs7239114	18	45921214	A	G	1,2E-04	49,638	0,0155	0,0022	3,9E-12	-0,0632	0,0408	0,1214	instrument
rs7250833	19	33937277	T	C	8,9E-05	38,543	0,0149	0,0024	7,2E-10	-0,0280	0,0447	0,5314	instrument
rs73124396	7	71579606	T	C	1,2E-04	32,111	0,0187	0,0033	1,1E-08	0,0121	0,0497	0,8081	instrument
rs73213484	4	28489339	A	T	1,7E-04	45,653	0,0250	0,0037	2,3E-11	-0,0911	0,0559	0,1035	instrument
rs7335249	13	109962579	T	C	8,7E-05	34,452	0,0135	0,0023	4,9E-09	-0,0218	0,0410	0,5946	instrument
rs7395632	11	958789	T	C	7,4E-05	29,340	-0,0130	0,0024	3,9E-08	-0,0417	0,0420	0,3215	instrument
rs7428670	3	131579810	C	G	1,5E-04	60,361	0,0202	0,0026	3,1E-15	-0,0399	0,0453	0,3780	instrument
rs7498044	15	92573639	A	G	1,2E-04	31,640	-0,0180	0,0032	2,7E-08	-0,0075	0,0505	0,8817	instrument
rs7498665	16	28883241	A	G	3,3E-04	143,999	-0,0264	0,0022	1,9E-32	-0,0708	0,0412	0,0851	instrument

rs750090	4	152931436	T	C	1,1E-04	42,250	0,0156	0,0024	7,4E-11	0,0013	0,0432	0,9760	instrument
rs7550711	1	110082886	T	C	2,1E-04	88,858	0,0641	0,0068	3,4E-21	0,0360	0,1215	0,7673	instrument
rs7571496	2	6169351	A	G	7,9E-05	31,102	0,0145	0,0026	1,4E-08	0,0141	0,0462	0,7607	instrument
rs7588437	2	181575281	A	G	1,3E-04	58,555	-0,0176	0,0023	8,4E-15	0,0598	0,0413	0,1473	instrument
rs7594237	2	198187164	A	T	8,3E-05	32,718	-0,0143	0,0025	1,8E-08	-0,0223	0,0462	0,6300	instrument
rs7601870	2	15384392	A	G	8,3E-05	32,937	-0,0132	0,0023	1,2E-08	0,0169	0,0410	0,6810	instrument
rs7601895	2	55281901	C	G	1,1E-04	41,473	0,0161	0,0025	9,6E-11	0,0284	0,0444	0,5231	instrument
rs7621025	3	136272246	T	C	1,5E-04	64,000	-0,0200	0,0025	1,6E-15	0,0895	0,0450	0,0469	instrument
rs7647242	3	82642734	T	C	1,0E-04	39,745	0,0145	0,0023	3,9E-10	-0,0045	0,0414	0,9128	instrument
rs768023	6	108876002	A	G	1,6E-04	70,712	0,0185	0,0022	1,4E-16	-0,0014	0,0415	0,9725	instrument
rs76900466	2	145057760	T	G	1,2E-04	30,654	0,0227	0,0041	4,5E-08	0,1346	0,0781	0,0847	instrument
rs77432547	13	86494817	A	G	1,4E-04	36,415	-0,0175	0,0029	2,3E-09	0,0133	0,0452	0,7685	instrument
rs7788008	7	112972483	A	G	1,4E-04	55,276	-0,0171	0,0023	1,1E-13	-0,0545	0,0406	0,1791	instrument
rs7805441	7	78121458	T	C	1,0E-04	40,295	0,0146	0,0023	2,0E-10	-0,0049	0,0402	0,9032	instrument
rs782971	5	43124688	A	G	1,1E-04	47,334	-0,0172	0,0025	4,9E-12	0,0514	0,0473	0,2773	instrument
rs786420	2	44719893	T	C	8,4E-05	32,956	0,0155	0,0027	8,8E-09	-0,0261	0,0467	0,5760	instrument
rs78886584	1	16859325	A	G	1,3E-04	33,284	-0,0150	0,0026	1,3E-08	0,0276	0,0452	0,5408	instrument
rs7899106	10	87410904	A	G	1,3E-04	56,250	-0,0375	0,0050	7,6E-14	-0,0092	0,0959	0,9237	instrument
rs79113395	1	1590521	A	G	1,9E-04	49,000	-0,0210	0,0030	1,5E-12	0,0512	0,0577	0,3752	instrument
rs8024806	15	53473990	T	C	8,5E-05	33,492	0,0272	0,0047	9,8E-09	-0,0257	0,0837	0,7586	instrument
rs8033510	15	61445514	T	C	8,3E-05	32,585	0,0137	0,0024	1,1E-08	-0,0459	0,0418	0,2725	instrument
rs8038522	15	46585105	T	G	9,4E-05	37,051	-0,0140	0,0023	1,3E-09	0,0948	0,0404	0,0188	instrument
rs8075273	17	61728881	A	C	1,0E-04	44,444	-0,0160	0,0024	5,6E-11	0,0434	0,0447	0,3321	instrument
rs811054	16	72251132	T	C	1,2E-04	54,223	0,0162	0,0022	3,2E-13	-0,0075	0,0406	0,8540	instrument
rs836525	7	6465909	T	C	8,2E-05	32,233	0,0176	0,0031	9,8E-09	0,0338	0,0547	0,5360	instrument
rs879620	16	4015729	T	C	2,9E-04	115,328	0,0247	0,0023	5,4E-26	0,0227	0,0417	0,5867	instrument
rs893006	11	64365796	A	C	1,2E-04	51,960	0,0173	0,0024	4,0E-13	0,0089	0,0444	0,8415	instrument
rs895330	19	4060707	C	G	9,9E-05	38,955	0,0181	0,0029	6,6E-10	-0,0167	0,0526	0,7514	instrument
rs915814	21	46493003	A	G	1,3E-04	34,810	-0,0177	0,0030	5,9E-09	-0,0062	0,0477	0,8970	instrument
rs925018	3	62713143	C	G	8,8E-05	38,117	-0,0142	0,0023	9,8E-10	0,0433	0,0432	0,3155	instrument
rs9304665	19	47602577	A	T	1,4E-04	57,086	0,0204	0,0027	1,8E-14	0,0969	0,0481	0,0438	instrument
rs9367369	6	13189941	A	G	1,4E-04	35,587	0,0173	0,0029	2,3E-09	-0,0341	0,0439	0,4373	instrument

rs942066	14	94031914	A	G	2,1E-04	55,972	-0,0202	0,0027	1,0E-13	-0,0014	0,0418	0,9733	instrument
rs943005	6	50865820	T	C	4,3E-04	188,351	0,0398	0,0029	2,0E-43	-0,0577	0,0535	0,2814	instrument
rs9531786	13	85983968	C	G	8,4E-05	33,062	-0,0138	0,0024	8,4E-09	-0,0099	0,0422	0,8152	instrument
rs9571687	13	67472713	A	C	9,2E-05	36,502	-0,0145	0,0024	3,2E-09	-0,0021	0,0431	0,9610	instrument
rs9579775	13	20616557	A	C	1,5E-04	39,690	-0,0252	0,0040	3,7E-10	0,0490	0,0713	0,4919	instrument
rs9595630	13	33065443	T	G	9,2E-05	39,745	0,0145	0,0023	2,0E-10	-0,1025	0,0409	0,0122	instrument
rs973345	9	29713169	T	C	9,7E-05	38,117	0,0142	0,0023	1,1E-09	-0,0070	0,0409	0,8645	instrument
rs9906944	17	47091420	T	C	1,6E-04	62,673	-0,0190	0,0024	7,3E-15	0,0159	0,0428	0,7097	instrument
rs9926784	16	19941968	T	C	1,9E-04	81,000	0,0252	0,0028	6,0E-20	0,0378	0,0520	0,4671	instrument
rs9937053	16	53799507	A	G	2,2E-03	941,370	0,0675	0,0022	1,0E-200	0,0612	0,0401	0,1271	instrument
rs994596	4	18459828	T	C	9,1E-05	36,000	0,0150	0,0025	1,0E-09	-0,0640	0,0433	0,1395	instrument
rs9955276	18	1839339	T	C	1,2E-04	48,129	0,0222	0,0032	8,0E-12	-0,0567	0,0582	0,3304	instrument

Table S9. Single Nucleotide Polymorphisms (SNPs) used as instruments in the Mendelian randomization analyses for the effect of BMI on endometrioid ovarian cancer based on the genome-wide significance threshold P = 5E-08. R² represents the explained variance in the exposure. SNP-specific instrument strength is given by the respective F-statistic. Some of the genetic variants were identified as outliers in the iterative approach and therefore excluded from the analyses.

SNP	CHR	POS	EA	OA	R^2	F-stat	β_{BMI}	SE_{BMI}	P_{BMI}	$\beta_{Endometrioid}$	$SE_{Endometrioid}$	$P_{Endometrioid}$	$variant_{Endometrioid}$
rs10002111	4	67815504	A	G	9,2E-05	36,430	0,0169	0,0028	9,3E-10	-0,0325	0,0344	0,3450	instrument
rs1003081	11	118913993	T	C	9,9E-05	42,843	0,0144	0,0022	6,3E-11	0,0049	0,0292	0,8657	instrument
rs10048652	2	35449306	T	C	8,7E-05	34,452	0,0135	0,0023	3,5E-09	0,0406	0,0288	0,1588	instrument
rs1009473	9	138777312	T	C	7,7E-05	30,489	-0,0127	0,0023	3,4E-08	-0,0252	0,0289	0,3831	instrument
rs10099330	8	143383694	A	G	8,3E-05	32,440	-0,0131	0,0023	9,9E-09	-0,0484	0,0289	0,0942	instrument
rs10116186	9	80532374	A	G	1,2E-04	31,102	-0,0145	0,0026	4,1E-08	0,0568	0,0291	0,0509	instrument
rs10118701	9	103061366	A	G	1,4E-04	53,778	-0,0176	0,0024	2,2E-13	0,0216	0,0303	0,4759	instrument
rs10124645	9	11321119	A	G	8,9E-05	34,964	0,0136	0,0023	5,0E-09	-0,0277	0,0298	0,3529	instrument
rs10182181	2	25150296	A	G	6,2E-04	270,751	-0,0362	0,0022	5,0E-63	0,0239	0,0295	0,4184	instrument
rs10237317	7	70045941	A	G	9,1E-05	39,347	-0,0138	0,0022	6,8E-10	-0,0132	0,0298	0,6578	instrument
rs10271582	7	99121950	A	G	1,2E-04	32,222	0,0193	0,0034	8,4E-09	-0,0134	0,0396	0,7354	instrument
rs10471636	5	63979856	A	G	1,3E-04	50,173	-0,0170	0,0024	4,7E-13	0,0059	0,0301	0,8451	instrument
rs1048932	11	115044850	A	C	1,6E-04	68,438	-0,0182	0,0022	2,5E-16	0,0147	0,0292	0,6152	instrument
rs10499694	7	50614173	A	G	8,8E-05	38,215	0,0136	0,0022	4,0E-10	0,0208	0,0288	0,4707	instrument
rs10513801	3	185822353	T	G	2,8E-04	120,999	0,0352	0,0032	1,3E-27	0,0389	0,0433	0,3685	instrument

rs10733682	9	129460914	A	G	1,2E-04	51,578	0,0158	0,0022	1,9E-12	0,0370	0,0289	0,2003	instrument
rs10742752	11	45438374	T	C	8,3E-05	36,000	-0,0132	0,0022	4,0E-09	-0,0051	0,0298	0,8650	instrument
rs10756714	9	15885041	A	G	2,1E-04	81,784	0,0208	0,0023	1,5E-19	0,0429	0,0289	0,1370	instrument
rs1075901	17	15943910	T	C	9,9E-05	42,843	-0,0144	0,0022	4,4E-11	-0,0394	0,0290	0,1735	instrument
rs10761785	10	65318766	T	G	1,4E-04	61,124	-0,0172	0,0022	4,2E-15	0,0777	0,0287	0,0067	outlier
rs10797115	9	92191256	T	C	1,2E-04	48,393	0,0160	0,0023	4,1E-12	0,0180	0,0289	0,5336	instrument
rs10823893	10	53677313	A	G	1,6E-04	42,009	0,0175	0,0027	5,2E-11	-0,0657	0,0294	0,0255	instrument
rs10824347	10	77645229	A	T	1,1E-04	42,491	0,0176	0,0027	5,5E-11	-0,0082	0,0339	0,8089	instrument
rs10909880	1	2727804	T	C	7,5E-05	31,768	-0,0124	0,0022	2,0E-08	-0,0425	0,0293	0,1468	instrument
rs10920678	1	190239907	A	G	1,3E-04	54,894	0,0163	0,0022	7,5E-14	0,0399	0,0294	0,1743	instrument
rs10938397	4	45182527	A	G	4,7E-04	205,009	-0,0315	0,0022	1,7E-45	0,0346	0,0289	0,2313	instrument
rs10962550	9	16720329	C	G	1,0E-04	40,960	0,0192	0,0030	1,7E-10	0,0648	0,0382	0,0919	instrument
rs10992867	9	96461013	A	G	1,3E-04	50,628	0,0185	0,0026	8,9E-13	-0,0081	0,0329	0,8048	instrument
rs11022762	11	13335926	T	C	1,0E-04	41,173	-0,0154	0,0024	5,6E-11	-0,0215	0,0295	0,4647	instrument
rs11073383	15	95274349	A	G	1,2E-04	47,790	-0,0159	0,0023	3,3E-12	-0,0135	0,0288	0,6398	instrument
rs11165643	1	96924097	T	C	1,6E-04	67,688	0,0181	0,0022	1,7E-16	-0,0144	0,0297	0,6274	instrument
rs112646560	1	39560250	T	C	1,4E-04	37,515	0,0196	0,0032	1,1E-09	-0,0134	0,0359	0,7099	instrument
rs11667280	19	30705747	C	G	7,5E-05	29,684	0,0158	0,0029	3,1E-08	0,0121	0,0376	0,7483	instrument
rs11672660	19	46180184	T	C	3,1E-04	131,429	-0,0321	0,0028	5,9E-31	-0,0029	0,0364	0,9376	instrument
rs11677607	2	100751150	T	C	2,0E-04	79,012	-0,0240	0,0027	2,9E-19	0,0013	0,0334	0,9689	instrument
rs1167800	7	75176196	A	G	2,0E-04	82,644	0,0200	0,0022	3,7E-19	-0,0474	0,0293	0,1058	instrument
rs11692326	2	208263279	T	C	8,3E-05	36,000	0,0156	0,0026	2,0E-09	0,0140	0,0345	0,6835	instrument
rs11727273	4	55509748	T	C	1,6E-04	42,250	-0,0169	0,0026	1,3E-10	-0,0067	0,0295	0,8195	instrument
rs11751591	6	33794215	A	G	8,5E-05	33,423	-0,0185	0,0032	1,2E-08	0,0179	0,0406	0,6599	instrument
rs11754747	6	141494602	T	C	7,3E-05	31,532	0,0146	0,0026	1,3E-08	0,0143	0,0347	0,6816	instrument
rs11880870	19	18830704	A	G	1,8E-04	73,363	0,0197	0,0023	3,3E-18	-0,0045	0,0294	0,8785	instrument
rs11945861	4	65700865	A	G	7,8E-05	30,864	-0,0150	0,0027	3,1E-08	0,0289	0,0340	0,3965	instrument
rs11971098	7	26699061	A	G	8,5E-05	33,414	-0,0237	0,0041	6,5E-09	-0,0185	0,0506	0,7148	instrument
rs12042959	1	243533273	A	G	9,3E-05	36,754	0,0194	0,0032	2,3E-09	0,0481	0,0413	0,2442	instrument
rs12089815	1	91189933	A	G	1,3E-04	34,628	-0,0153	0,0026	6,0E-09	0,0374	0,0296	0,2066	instrument
rs12101625	15	26972431	C	G	8,4E-05	33,062	0,0138	0,0024	5,5E-09	-0,0356	0,0297	0,2302	instrument
rs12140153	1	62579891	T	G	1,8E-04	48,110	-0,0326	0,0047	2,9E-12	0,0568	0,0521	0,2761	instrument

rs12150665	17	34914787	T	C	1,3E-04	57,622	0,0167	0,0022	5,9E-14	0,0134	0,0304	0,6591	instrument
rs12202969	6	98576223	A	G	1,0E-04	39,745	-0,0145	0,0023	1,9E-10	0,0052	0,0291	0,8577	instrument
rs12207125	6	154301575	A	G	1,3E-04	34,468	0,0182	0,0031	7,7E-09	-0,0377	0,0350	0,2814	instrument
rs12364470	11	134601012	T	G	1,0E-04	44,444	-0,0200	0,0030	2,5E-11	0,0449	0,0428	0,2948	instrument
rs12429545	13	54102206	A	G	1,8E-04	77,760	0,0291	0,0033	1,1E-18	0,0377	0,0440	0,3923	instrument
rs12448257	16	3599655	A	G	7,2E-05	30,864	0,0150	0,0027	3,1E-08	0,0838	0,0356	0,0186	instrument
rs12462975	19	30272202	A	G	1,7E-04	65,934	0,0203	0,0025	2,6E-16	0,0205	0,0305	0,5026	instrument
rs12564992	1	174478100	A	G	8,0E-05	34,979	-0,0207	0,0035	2,4E-09	0,0406	0,0453	0,3699	instrument
rs12593036	15	81058652	A	G	9,6E-05	37,945	0,0154	0,0025	6,6E-10	-0,0354	0,0316	0,2627	instrument
rs12612009	2	228982603	T	G	8,7E-05	34,515	-0,0141	0,0024	3,8E-09	0,0431	0,0311	0,1657	instrument
rs12645001	4	96018337	A	G	8,7E-05	34,515	-0,0141	0,0024	4,1E-09	-0,0230	0,0302	0,4463	instrument
rs12680842	8	95582606	A	G	6,8E-05	29,340	0,0130	0,0024	3,4E-08	-0,0342	0,0305	0,2618	instrument
rs12681792	8	62054463	A	C	1,0E-04	39,820	0,0183	0,0029	2,6E-10	0,0387	0,0374	0,3012	instrument
rs12692596	2	161265910	T	C	9,3E-05	40,295	0,0146	0,0023	9,7E-11	0,0014	0,0298	0,9626	instrument
rs12692738	2	165558252	T	C	8,9E-05	38,822	-0,0162	0,0026	2,8E-10	0,0150	0,0341	0,6609	instrument
rs12714199	2	86812549	T	C	1,0E-04	40,295	-0,0146	0,0023	5,1E-10	0,0352	0,0299	0,2387	instrument
rs1285997	14	91513029	C	G	9,2E-05	36,000	-0,0150	0,0025	3,2E-09	-0,0696	0,0321	0,0304	instrument
rs12872889	13	28674628	T	C	1,7E-04	45,453	-0,0209	0,0031	1,5E-11	-0,0432	0,0341	0,2057	instrument
rs1289736	3	107950892	T	C	9,3E-05	36,523	0,0139	0,0023	1,4E-09	-0,0166	0,0290	0,5668	instrument
rs12914489	15	74187937	A	G	9,1E-05	39,760	0,0227	0,0036	1,6E-10	0,0276	0,0463	0,5510	instrument
rs12949279	17	78558411	T	C	1,5E-04	66,200	0,0179	0,0022	6,1E-16	-0,0038	0,0294	0,8972	instrument
rs12981256	19	1865901	A	G	1,6E-04	62,616	0,0182	0,0023	3,9E-15	-0,0247	0,0294	0,4006	instrument
rs13002158	2	144021712	A	G	1,3E-04	51,443	-0,0208	0,0029	1,2E-12	-0,0123	0,0381	0,7470	instrument
rs13053342	22	48871624	A	G	1,0E-04	40,640	0,0153	0,0024	1,3E-10	-0,0147	0,0325	0,6505	instrument
rs13062093	3	35667057	T	G	8,3E-05	32,585	-0,0137	0,0024	8,0E-09	-0,0088	0,0298	0,7667	instrument
rs13068138	3	85809425	T	C	1,7E-04	73,197	0,0231	0,0027	3,6E-17	0,0142	0,0361	0,6944	instrument
rs13076052	3	44456573	C	G	1,2E-04	32,766	-0,0166	0,0029	1,3E-08	-0,0060	0,0329	0,8548	instrument
rs13107325	4	103188709	T	C	2,4E-04	105,528	0,0452	0,0044	9,3E-25	0,0022	0,0554	0,9682	instrument
rs13110266	4	162129844	A	G	9,6E-05	41,661	-0,0142	0,0022	1,5E-10	-0,0260	0,0293	0,3745	instrument
rs13174863	5	139080745	A	G	1,8E-04	75,858	-0,0270	0,0031	4,5E-18	-0,0336	0,0424	0,4284	instrument
rs13209872	6	97753223	C	G	8,9E-05	35,007	-0,0142	0,0024	6,1E-09	0,0138	0,0307	0,6528	instrument
rs13213408	6	162998049	A	T	9,0E-05	35,637	0,0197	0,0033	4,3E-09	0,0175	0,0425	0,6799	instrument

rs1362910	8	30856464	A	G	8,2E-05	33,943	0,0134	0,0023	3,0E-09	0,0063	0,0291	0,8289	instrument
rs1403846	4	119101723	T	C	7,2E-05	31,206	0,0162	0,0029	1,6E-08	0,0224	0,0376	0,5507	instrument
rs1409818	20	21381121	T	C	1,2E-04	46,387	0,0252	0,0037	1,6E-11	0,0293	0,0472	0,5349	instrument
rs141729694	5	87999371	T	C	2,2E-04	57,284	0,0386	0,0051	2,5E-14	0,0694	0,0535	0,1946	instrument
rs1417665	6	104816944	T	C	1,2E-04	47,088	0,0199	0,0029	5,7E-12	-0,0277	0,0372	0,4555	instrument
rs1421598	2	50264710	A	C	9,7E-05	38,360	-0,0192	0,0031	6,8E-10	-0,0098	0,0390	0,8028	instrument
rs1436348	3	104612668	A	G	8,6E-05	33,943	-0,0134	0,0023	6,3E-09	-0,0225	0,0294	0,4447	instrument
rs1441264	13	79580919	A	G	1,3E-04	55,276	0,0171	0,0023	5,2E-14	0,0184	0,0301	0,5412	instrument
rs1452075	3	62481063	T	C	9,4E-05	40,449	0,0159	0,0025	1,5E-10	0,0448	0,0329	0,1735	instrument
rs1491333	4	172186288	T	G	8,1E-05	31,966	-0,0147	0,0026	9,9E-09	0,0266	0,0336	0,4285	instrument
rs1492014	3	94071481	T	C	1,0E-04	39,745	-0,0145	0,0023	2,8E-10	0,0091	0,0290	0,7530	instrument
rs1518159	18	40760146	T	C	1,3E-04	52,841	-0,0189	0,0026	6,7E-13	-0,0189	0,0333	0,5700	instrument
rs1561589	10	126695673	A	G	1,1E-04	47,191	0,0158	0,0023	4,2E-12	0,0421	0,0296	0,1547	instrument
rs1569979	14	29681294	A	G	1,8E-04	72,565	0,0230	0,0027	1,6E-17	0,0212	0,0341	0,5335	instrument
rs1579557	6	40371918	T	C	1,8E-04	71,910	0,0212	0,0025	3,1E-17	0,0084	0,0324	0,7954	instrument
rs158186	5	107440856	A	G	2,2E-04	85,711	-0,0287	0,0031	3,2E-20	0,0242	0,0378	0,5231	instrument
rs159032	5	94206202	T	C	7,8E-05	30,454	0,0149	0,0027	4,5E-08	0,0341	0,0332	0,3043	instrument
rs1681743	10	118565501	T	C	7,5E-05	29,793	-0,0131	0,0024	3,7E-08	-0,0260	0,0295	0,3782	instrument
rs16851483	3	141275436	T	G	1,6E-04	62,272	0,0363	0,0046	2,3E-15	-0,0351	0,0591	0,5523	instrument
rs16912921	9	28413461	A	C	2,7E-04	117,204	0,0249	0,0023	1,6E-26	0,0337	0,0308	0,2736	instrument
rs16975921	18	39914870	A	T	1,1E-04	42,510	-0,0163	0,0025	3,9E-11	-0,0780	0,0303	0,0100	instrument
rs16989232	20	39291784	A	G	7,1E-05	30,972	0,0128	0,0023	1,5E-08	0,0101	0,0302	0,7395	instrument
rs17014375	1	209543560	T	G	7,9E-05	31,228	-0,0190	0,0034	1,5E-08	-0,0391	0,0416	0,3480	instrument
rs17066856	18	58049656	T	C	2,7E-04	115,707	0,0398	0,0037	1,4E-27	0,1131	0,0509	0,0263	instrument
rs17405819	8	76806584	T	C	1,9E-04	82,507	0,0218	0,0024	7,8E-20	0,0160	0,0311	0,6076	instrument
rs1750307	1	156488420	A	T	8,3E-05	32,585	0,0137	0,0024	8,2E-09	-0,0128	0,0299	0,6680	instrument
rs17522122	14	33302882	T	G	1,3E-04	57,622	0,0167	0,0022	6,5E-14	-0,0424	0,0293	0,1486	instrument
rs17619973	3	114417675	A	G	7,4E-05	31,673	0,0242	0,0043	1,3E-08	0,0425	0,0596	0,4760	instrument
rs17724992	19	18454825	A	G	1,1E-04	46,240	0,0170	0,0025	6,7E-12	-0,0183	0,0325	0,5731	instrument
rs17751061	19	19413092	T	C	8,2E-05	31,993	-0,0181	0,0032	1,3E-08	-0,0060	0,0397	0,8805	instrument
rs17767765	16	24539793	T	C	7,7E-05	30,489	-0,0127	0,0023	2,6E-08	-0,0245	0,0297	0,4092	instrument
rs17806224	20	51065854	A	G	2,3E-04	92,801	-0,0289	0,0030	3,5E-22	-0,0460	0,0387	0,2342	instrument

rs1799923	3	42306294	A	G	1,2E-04	48,203	-0,0243	0,0035	5,1E-12	0,0089	0,0478	0,8520	instrument
rs1801282	3	12393125	C	G	7,0E-05	30,417	-0,0182	0,0033	4,7E-08	0,0851	0,0443	0,0545	instrument
rs1808579	18	21104888	T	C	1,9E-04	81,000	-0,0198	0,0022	9,4E-20	-0,0107	0,0289	0,7104	instrument
rs1808629	8	73435964	A	G	2,5E-04	65,147	-0,0226	0,0028	5,8E-16	0,0238	0,0314	0,4489	instrument
rs1812587	5	62991802	T	G	1,3E-04	34,177	0,0152	0,0026	7,0E-09	-0,0027	0,0290	0,9261	instrument
rs185350	19	34306816	T	C	7,8E-05	33,851	0,0128	0,0022	7,1E-09	-0,0117	0,0287	0,6831	instrument
rs1861412	2	58893065	A	G	2,2E-04	87,381	-0,0215	0,0023	9,1E-21	0,0088	0,0289	0,7602	instrument
rs1884389	20	1410582	T	C	9,4E-05	37,051	-0,0140	0,0023	1,2E-09	0,0028	0,0290	0,9236	instrument
rs1884897	20	6612832	A	G	1,2E-04	53,990	-0,0169	0,0023	9,1E-14	-0,0164	0,0300	0,5836	instrument
rs1916801	3	61187046	A	T	1,4E-04	59,710	0,0170	0,0022	1,8E-14	-0,0190	0,0295	0,5180	instrument
rs1927790	13	96922191	T	C	8,7E-05	37,655	-0,0135	0,0022	1,3E-09	-0,0229	0,0294	0,4358	instrument
rs1928295	9	120378483	T	C	1,2E-04	52,892	0,0160	0,0022	2,8E-13	-0,0035	0,0288	0,9043	instrument
rs1949197	3	86273850	A	G	9,2E-05	36,523	0,0139	0,0023	3,0E-09	0,0305	0,0294	0,2997	instrument
rs197374	1	112289983	T	C	1,4E-04	55,276	0,0171	0,0023	2,8E-13	-0,0238	0,0297	0,4225	instrument
rs1984559	2	135678888	A	G	7,9E-05	29,521	0,0163	0,0030	3,3E-08	0,0270	0,0349	0,4388	instrument
rs198665	6	143154886	A	C	7,8E-05	33,943	0,0134	0,0023	4,4E-09	0,0127	0,0302	0,6738	instrument
rs2012502	16	81728081	A	C	1,0E-04	44,251	0,0153	0,0023	3,0E-11	0,0130	0,0301	0,6672	instrument
rs2045293	7	114350102	T	C	1,7E-04	67,525	0,0189	0,0023	4,1E-16	0,0061	0,0293	0,8351	instrument
rs2053682	5	170599327	A	C	1,4E-04	53,582	0,0183	0,0025	1,9E-13	0,0385	0,0310	0,2140	instrument
rs209416	6	83534729	A	T	1,2E-04	47,840	0,0166	0,0024	8,7E-12	-0,0298	0,0311	0,3377	instrument
rs2112347	5	75015242	T	G	4,2E-04	180,493	0,0309	0,0023	2,1E-42	-0,0630	0,0297	0,0337	instrument
rs214249	16	348687	T	G	1,2E-04	48,418	0,0167	0,0024	1,4E-12	-0,0120	0,0290	0,6796	instrument
rs2161097	5	103945178	T	C	8,0E-05	31,457	0,0129	0,0023	2,3E-08	-0,0181	0,0288	0,5297	instrument
rs2178899	6	31606756	A	T	1,3E-04	56,477	0,0248	0,0033	7,2E-14	0,0290	0,0446	0,5156	instrument
rs2185027	6	153381622	A	C	9,5E-05	41,173	-0,0154	0,0024	1,1E-10	-0,0008	0,0313	0,9800	instrument
rs2228213	6	12124855	A	G	9,5E-05	41,406	-0,0148	0,0023	9,2E-11	-0,0108	0,0299	0,7175	instrument
rs2267812	7	74138121	A	C	9,2E-05	35,177	-0,0172	0,0029	1,7E-09	-0,0212	0,0349	0,5445	instrument
rs2267958	9	131015279	A	G	1,2E-04	45,002	-0,0161	0,0024	1,9E-11	-0,0227	0,0304	0,4552	instrument
rs2271046	4	52752812	A	T	7,6E-05	30,030	-0,0137	0,0025	2,4E-08	0,0229	0,0323	0,4783	instrument
rs2289292	16	30097630	T	C	1,5E-04	57,760	-0,0190	0,0025	1,1E-14	-0,0532	0,0321	0,0968	instrument
rs2289705	7	121983843	A	G	7,7E-05	33,284	-0,0150	0,0026	1,5E-08	0,0082	0,0357	0,8181	instrument
rs2299383	7	103418846	T	C	1,6E-04	68,438	0,0182	0,0022	2,7E-16	-0,0125	0,0292	0,6693	instrument

rs2357760	6	120213880	A	G	1,1E-04	47,790	0,0159	0,0023	1,1E-11	0,0031	0,0306	0,9183	instrument
rs2465043	6	51180765	A	G	8,9E-05	35,007	-0,0142	0,0024	3,2E-09	0,0173	0,0302	0,5669	instrument
rs2470520	3	15762920	T	C	7,6E-05	30,011	-0,0126	0,0023	4,2E-08	-0,0099	0,0291	0,7327	instrument
rs247975	3	173107443	T	C	1,6E-04	62,616	-0,0182	0,0023	3,6E-15	0,0093	0,0302	0,7585	instrument
rs2479958	13	111984244	A	G	8,1E-05	31,640	0,0135	0,0024	1,4E-08	0,0292	0,0295	0,3211	instrument
rs2528531	7	93236510	A	C	9,3E-05	36,502	-0,0145	0,0024	1,3E-09	-0,0547	0,0298	0,0665	instrument
rs2568958	1	72765116	A	G	2,5E-04	106,464	0,0227	0,0022	1,3E-24	0,0567	0,0297	0,0561	instrument
rs2605603	11	93221105	A	G	8,3E-05	36,000	-0,0132	0,0022	1,4E-09	0,0082	0,0288	0,7744	instrument
rs2619976	17	71754545	T	C	9,4E-05	37,007	0,0146	0,0024	6,2E-10	-0,0088	0,0306	0,7737	instrument
rs2721965	8	116662038	A	C	1,5E-04	60,062	0,0186	0,0024	2,1E-14	0,0956	0,0309	0,0019	outlier
rs273504	19	18215247	A	G	1,1E-04	43,102	-0,0151	0,0023	1,3E-10	-0,0206	0,0291	0,4785	instrument
rs2754079	14	25886140	A	G	1,2E-04	46,004	-0,0156	0,0023	2,3E-11	-0,0313	0,0297	0,2931	instrument
rs2814992	6	34617144	A	G	3,6E-04	155,706	-0,0287	0,0023	1,2E-35	0,0270	0,0304	0,3751	instrument
rs2820295	1	201800868	A	G	2,0E-04	79,507	0,0214	0,0024	8,0E-19	0,0594	0,0301	0,0494	instrument
rs2836754	21	40291740	T	C	1,1E-04	45,870	-0,0149	0,0022	3,7E-11	-0,0033	0,0295	0,9122	instrument
rs28489620	22	41804716	A	G	1,5E-04	38,955	-0,0181	0,0029	6,9E-10	-0,0631	0,0320	0,0485	instrument
rs2861685	2	67837553	T	C	1,8E-04	47,929	0,0180	0,0026	1,1E-11	-0,0312	0,0290	0,2824	instrument
rs2862961	11	43656535	A	G	2,3E-04	91,393	-0,0239	0,0025	5,3E-22	-0,0437	0,0309	0,1571	instrument
rs2959592	8	17002725	T	C	8,7E-05	36,862	0,0170	0,0028	6,3E-10	-0,0558	0,0376	0,1376	instrument
rs2968864	7	150622162	T	C	9,2E-05	36,446	0,0163	0,0027	9,3E-10	-0,0298	0,0332	0,3695	instrument
rs2984618	1	47690438	T	G	1,3E-04	57,622	0,0167	0,0022	2,2E-14	0,0254	0,0293	0,3859	instrument
rs329651	11	133767622	T	G	9,7E-05	41,787	0,0181	0,0028	2,0E-10	-0,0020	0,0374	0,9564	instrument
rs337637	4	38604470	A	G	6,8E-05	29,537	-0,0125	0,0023	3,0E-08	-0,0119	0,0302	0,6940	instrument
rs34517439	1	78450517	A	C	3,4E-04	90,481	0,0390	0,0041	2,0E-21	-0,0488	0,0471	0,3001	instrument
rs34811474	4	25408838	A	G	4,0E-04	104,566	-0,0317	0,0031	5,2E-24	-0,0097	0,0399	0,8088	instrument
rs34930419	10	33987329	T	C	1,4E-04	36,316	-0,0458	0,0076	1,4E-09	0,0241	0,0862	0,7796	instrument
rs35107973	4	143125948	T	C	1,2E-04	32,573	0,0234	0,0041	7,7E-09	0,0758	0,0454	0,0951	instrument
rs355754	3	154024165	T	C	9,2E-05	38,117	-0,0142	0,0023	4,3E-10	0,0028	0,0296	0,9249	instrument
rs35722922	2	47002226	A	G	1,8E-04	48,482	0,0188	0,0027	3,6E-12	-0,0170	0,0299	0,5701	instrument
rs3803286	14	103246470	A	G	1,3E-04	51,361	0,0172	0,0024	8,3E-13	0,0131	0,0302	0,6655	instrument
rs3806114	6	20482335	A	G	8,0E-05	34,515	-0,0141	0,0024	4,5E-09	-0,0302	0,0310	0,3291	instrument
rs3826408	17	7101292	T	C	7,0E-05	30,250	0,0121	0,0022	4,5E-08	-0,0023	0,0292	0,9367	instrument

rs3829849	9	129390800	T	C	8,0E-05	34,964	0,0136	0,0023	1,9E-09	-0,0152	0,0300	0,6130	instrument
rs3977755	10	104420210	T	C	8,0E-05	32,718	-0,0143	0,0025	8,5E-09	-0,0294	0,0320	0,3571	instrument
rs4055791	13	59266053	T	C	1,5E-04	39,643	-0,0170	0,0027	1,8E-10	-0,0001	0,0294	0,9962	instrument
rs4148155	4	89054667	A	G	7,7E-05	33,640	0,0203	0,0035	6,2E-09	0,0146	0,0465	0,7527	instrument
rs4240673	8	10787612	T	C	1,5E-04	64,000	0,0176	0,0022	1,0E-15	0,0179	0,0377	0,6349	instrument
rs427943	21	46570896	A	C	1,5E-04	59,223	-0,0177	0,0023	1,4E-14	-0,0019	0,0294	0,9491	instrument
rs429358	19	45411941	T	C	2,2E-04	58,777	0,0276	0,0036	3,2E-14	-0,0236	0,0414	0,5687	instrument
rs4295917	1	32173360	A	G	8,2E-05	31,640	0,0135	0,0024	1,6E-08	0,0052	0,0311	0,8658	instrument
rs4307239	7	24354300	A	G	9,5E-05	37,582	-0,0141	0,0023	1,3E-09	-0,0106	0,0293	0,7189	instrument
rs4320543	8	34422967	A	G	8,0E-05	31,457	-0,0129	0,0023	2,9E-08	0,0467	0,0300	0,1201	instrument
rs4421623	1	98316285	T	G	1,2E-04	46,441	0,0184	0,0027	1,1E-11	0,0371	0,0342	0,2774	instrument
rs4459316	11	130756176	T	C	1,1E-04	44,832	-0,0154	0,0023	2,3E-11	0,0226	0,0291	0,4385	instrument
rs4474229	1	195037001	A	G	1,6E-04	41,055	-0,0173	0,0027	1,6E-10	0,0465	0,0294	0,1144	instrument
rs4482463	2	205375909	A	C	2,0E-04	75,524	-0,0365	0,0042	5,2E-18	0,0682	0,0551	0,2158	instrument
rs4569924	5	153540025	T	C	1,2E-04	51,578	0,0158	0,0022	7,4E-13	0,0303	0,0291	0,2966	instrument
rs4586287	13	99245334	C	G	7,7E-05	30,470	0,0138	0,0025	2,9E-08	-0,0398	0,0312	0,2020	instrument
rs4673553	2	211608379	T	G	1,1E-04	44,251	-0,0153	0,0023	2,6E-11	0,0117	0,0291	0,6880	instrument
rs4677812	3	194863794	A	C	8,4E-05	33,177	-0,0144	0,0025	9,4E-09	0,0457	0,0320	0,1530	instrument
rs4776982	15	68114974	A	G	3,3E-04	143,078	0,0311	0,0026	4,2E-33	-0,0365	0,0342	0,2859	instrument
rs4783718	16	69547741	T	C	1,8E-04	78,669	0,0204	0,0023	1,8E-19	0,0482	0,0303	0,1117	instrument
rs4790292	17	1824305	A	C	1,7E-04	63,516	-0,0263	0,0033	1,7E-15	0,0293	0,0428	0,4927	instrument
rs4834272	4	113313986	T	C	8,9E-05	38,656	-0,0143	0,0023	8,2E-10	-0,0704	0,0311	0,0236	instrument
rs4858223	3	20581149	T	C	1,1E-04	41,473	-0,0161	0,0025	8,3E-11	0,0157	0,0310	0,6123	instrument
rs4864201	4	130731284	T	C	7,5E-05	32,440	0,0131	0,0023	6,3E-09	0,0138	0,0300	0,6444	instrument
rs4880341	10	133992689	T	C	9,8E-05	38,656	-0,0143	0,0023	4,5E-10	0,0012	0,0290	0,9679	instrument
rs4889606	16	31011183	A	G	3,1E-04	135,773	0,0268	0,0023	1,3E-32	0,0430	0,0300	0,1518	instrument
rs4929923	11	8639200	T	C	2,1E-04	92,327	-0,0221	0,0023	2,5E-22	-0,0175	0,0298	0,5579	instrument
rs4936175	11	132641959	T	C	1,2E-04	47,191	-0,0158	0,0023	6,7E-12	-0,0239	0,0289	0,4085	instrument
rs4969387	17	79081724	C	G	8,4E-05	32,532	-0,0154	0,0027	6,9E-09	0,0452	0,0333	0,1745	instrument
rs4986044	17	21261560	T	C	1,4E-04	61,836	-0,0173	0,0022	2,2E-15	-0,0082	0,0287	0,7737	instrument
rs543874	1	177889480	A	G	1,0E-03	455,109	-0,0576	0,0027	8,7E-100	-0,0448	0,0362	0,2159	instrument
rs56161855	17	46288649	A	T	1,7E-04	44,787	-0,0261	0,0039	1,5E-11	0,0785	0,0429	0,0650	instrument

rs563132	9	37245476	A	T	1,4E-04	56,250	0,0180	0,0024	2,0E-14	-0,0393	0,0293	0,1799	instrument
rs563296	10	99772404	A	G	1,4E-04	53,168	0,0175	0,0024	8,8E-14	-0,0647	0,0292	0,0267	instrument
rs57221746	15	99230035	A	G	1,2E-04	30,594	-0,0177	0,0032	2,1E-08	0,0364	0,0343	0,2885	instrument
rs57800857	4	140863365	A	C	1,8E-04	47,456	0,0186	0,0027	1,1E-11	0,0820	0,0301	0,0064	instrument
rs6010784	20	61540319	T	C	7,6E-05	32,802	0,0126	0,0022	1,1E-08	-0,0021	0,0288	0,9417	instrument
rs6050446	20	25195509	A	G	8,0E-05	33,785	-0,0372	0,0064	6,2E-09	-0,1357	0,0917	0,1386	instrument
rs6142096	20	32686658	A	G	8,6E-05	33,943	0,0134	0,0023	4,2E-09	0,0175	0,0286	0,5413	instrument
rs61813324	1	156049877	T	C	2,2E-04	58,385	0,0298	0,0039	3,6E-14	0,0977	0,0455	0,0319	instrument
rs62007782	15	78029797	A	G	1,4E-04	35,601	-0,0179	0,0030	1,9E-09	0,0133	0,0328	0,6847	instrument
rs62106258	2	417167	T	C	9,7E-04	254,452	0,0989	0,0062	8,7E-58	0,1031	0,0793	0,1938	instrument
rs62412826	6	61881167	T	C	1,3E-04	33,518	0,0330	0,0057	8,0E-09	0,0002	0,0632	0,9977	instrument
rs625652	11	65641931	A	T	2,0E-04	52,160	-0,0195	0,0027	9,1E-13	0,0446	0,0301	0,1387	instrument
rs6265	11	27679916	T	C	4,0E-04	172,734	-0,0368	0,0028	8,0E-40	0,0137	0,0364	0,7058	instrument
rs6446187	3	49907111	A	C	4,3E-04	114,325	0,0278	0,0026	1,2E-26	0,0140	0,0287	0,6243	instrument
rs6493498	15	51754451	T	C	1,3E-04	54,894	0,0163	0,0022	1,5E-13	-0,0124	0,0291	0,6704	instrument
rs6548237	2	621461	A	C	9,1E-04	394,500	-0,0576	0,0029	4,4E-89	-0,0704	0,0483	0,1449	instrument
rs6556810	5	164585949	T	C	7,4E-05	29,066	-0,0124	0,0023	5,0E-08	0,0362	0,0294	0,2173	instrument
rs6561937	13	58257667	A	T	1,4E-04	53,236	-0,0197	0,0027	6,0E-13	0,0408	0,0346	0,2378	instrument
rs6567160	18	57829135	T	C	1,1E-03	475,573	-0,0567	0,0026	8,7E-108	-0,0559	0,0334	0,0944	instrument
rs657452	1	49589847	A	G	2,0E-04	85,142	0,0203	0,0022	1,1E-19	-0,0388	0,0293	0,1848	instrument
rs6585198	10	114762237	A	G	1,1E-04	49,000	-0,0154	0,0022	1,2E-12	-0,0254	0,0286	0,3746	instrument
rs6669189	1	75011358	T	C	2,1E-04	91,115	0,0210	0,0022	4,7E-21	0,0356	0,0291	0,2215	instrument
rs6720868	2	230663576	T	C	1,3E-04	49,585	0,0169	0,0024	4,6E-12	0,0502	0,0302	0,0968	instrument
rs6728037	2	59297346	A	G	2,1E-04	81,000	0,0207	0,0023	6,5E-19	0,0629	0,0295	0,0327	instrument
rs6739199	2	105452326	T	C	1,0E-04	40,449	0,0159	0,0025	3,2E-10	0,0530	0,0329	0,1068	instrument
rs6870983	5	87697533	T	C	1,5E-04	66,485	-0,0212	0,0026	9,6E-16	-0,0536	0,0345	0,1211	instrument
rs6890310	5	27193573	A	G	9,6E-05	37,945	-0,0154	0,0025	1,4E-09	-0,0037	0,0329	0,9110	instrument
rs6973700	7	1856669	A	G	1,4E-04	36,376	0,0193	0,0032	1,8E-09	0,0137	0,0352	0,6965	instrument
rs7024334	9	109072075	T	G	8,7E-05	37,398	0,0159	0,0026	1,6E-09	0,0272	0,0342	0,4270	instrument
rs7084454	10	21821274	A	G	2,1E-04	81,000	0,0225	0,0025	7,4E-20	0,0598	0,0302	0,0484	instrument
rs709400	14	104149475	A	G	1,5E-04	64,000	0,0184	0,0023	4,4E-16	0,0145	0,0295	0,6219	instrument
rs7105462	11	112912048	A	G	9,9E-05	39,198	0,0144	0,0023	4,4E-10	0,0165	0,0292	0,5734	instrument

rs7107409	11	76492195	T	C	1,2E-04	30,941	-0,0178	0,0032	2,1E-08	0,0374	0,0345	0,2780	instrument
rs7124681	11	47529947	A	C	2,9E-04	126,051	0,0247	0,0022	9,3E-29	-0,0273	0,0292	0,3503	instrument
rs7141420	14	79899454	T	C	2,1E-04	91,115	0,0210	0,0022	7,8E-22	0,0369	0,0295	0,2115	instrument
rs7211966	17	54374611	T	C	8,9E-05	34,515	-0,0470	0,0080	4,9E-09	0,0443	0,1245	0,7222	instrument
rs7239114	18	45921214	A	G	1,2E-04	49,638	0,0155	0,0022	3,9E-12	-0,0193	0,0294	0,5123	instrument
rs7250833	19	33937277	T	C	8,9E-05	38,543	0,0149	0,0024	7,2E-10	-0,0024	0,0318	0,9394	instrument
rs73124396	7	71579606	T	C	1,2E-04	32,111	0,0187	0,0033	1,1E-08	-0,0094	0,0352	0,7898	instrument
rs73213484	4	28489339	A	T	1,7E-04	45,653	0,0250	0,0037	2,3E-11	-0,0015	0,0405	0,9707	instrument
rs7335249	13	109962579	T	C	8,7E-05	34,452	0,0135	0,0023	4,9E-09	-0,0320	0,0294	0,2761	instrument
rs7395632	11	958789	T	C	7,4E-05	29,340	-0,0130	0,0024	3,9E-08	-0,0236	0,0300	0,4311	instrument
rs7428670	3	131579810	C	G	1,5E-04	60,361	0,0202	0,0026	3,1E-15	-0,0160	0,0325	0,6217	instrument
rs7498044	15	92573639	A	G	1,2E-04	31,640	-0,0180	0,0032	2,7E-08	-0,0727	0,0369	0,0490	instrument
rs7498665	16	28883241	A	G	3,3E-04	143,999	-0,0264	0,0022	1,9E-32	-0,0680	0,0295	0,0210	instrument
rs750090	4	152931436	T	C	1,1E-04	42,250	0,0156	0,0024	7,4E-11	0,1001	0,0312	0,0013	outlier
rs7550711	1	110082886	T	C	2,1E-04	88,858	0,0641	0,0068	3,4E-21	0,1883	0,0821	0,0218	instrument
rs7571496	2	6169351	A	G	7,9E-05	31,102	0,0145	0,0026	1,4E-08	-0,0182	0,0330	0,5806	instrument
rs7588437	2	181575281	A	G	1,3E-04	58,555	-0,0176	0,0023	8,4E-15	-0,0174	0,0298	0,5587	instrument
rs7594237	2	198187164	A	T	8,3E-05	32,718	-0,0143	0,0025	1,8E-08	-0,0007	0,0329	0,9840	instrument
rs7601870	2	15384392	A	G	8,3E-05	32,937	-0,0132	0,0023	1,2E-08	-0,0086	0,0293	0,7683	instrument
rs7601895	2	55281901	C	G	1,1E-04	41,473	0,0161	0,0025	9,6E-11	0,0052	0,0318	0,8704	instrument
rs7621025	3	136272246	T	C	1,5E-04	64,000	-0,0200	0,0025	1,6E-15	0,0194	0,0328	0,5534	instrument
rs7647242	3	82642734	T	C	1,0E-04	39,745	0,0145	0,0023	3,9E-10	0,0035	0,0296	0,9057	instrument
rs768023	6	108876002	A	G	1,6E-04	70,712	0,0185	0,0022	1,4E-16	-0,0267	0,0296	0,3670	instrument
rs76900466	2	145057760	T	G	1,2E-04	30,654	0,0227	0,0041	4,5E-08	-0,0423	0,0542	0,4351	instrument
rs77432547	13	86494817	A	G	1,4E-04	36,415	-0,0175	0,0029	2,3E-09	0,0537	0,0324	0,0970	instrument
rs7788008	7	112972483	A	G	1,4E-04	55,276	-0,0171	0,0023	1,1E-13	-0,0422	0,0290	0,1451	instrument
rs7805441	7	78121458	T	C	1,0E-04	40,295	0,0146	0,0023	2,0E-10	0,0022	0,0287	0,9388	instrument
rs782971	5	43124688	A	G	1,1E-04	47,334	-0,0172	0,0025	4,9E-12	-0,0093	0,0337	0,7831	instrument
rs786420	2	44719893	T	C	8,4E-05	32,956	0,0155	0,0027	8,8E-09	-0,0183	0,0333	0,5819	instrument
rs78886584	1	16859325	A	G	1,3E-04	33,284	-0,0150	0,0026	1,3E-08	-0,0607	0,0327	0,0635	instrument
rs7899106	10	87410904	A	G	1,3E-04	56,250	-0,0375	0,0050	7,6E-14	0,0092	0,0693	0,8949	instrument
rs79113395	1	1590521	A	G	1,9E-04	49,000	-0,0210	0,0030	1,5E-12	0,0474	0,0417	0,2558	instrument

rs8024806	15	53473990	T	C	8,5E-05	33,492	0,0272	0,0047	9,8E-09	0,0802	0,0611	0,1893	instrument
rs8033510	15	61445514	T	C	8,3E-05	32,585	0,0137	0,0024	1,1E-08	0,0271	0,0297	0,3606	instrument
rs8038522	15	46585105	T	G	9,4E-05	37,051	-0,0140	0,0023	1,3E-09	0,0073	0,0290	0,8005	instrument
rs8075273	17	61728881	A	C	1,0E-04	44,444	-0,0160	0,0024	5,6E-11	0,0352	0,0320	0,2717	instrument
rs811054	16	72251132	T	C	1,2E-04	54,223	0,0162	0,0022	3,2E-13	0,0149	0,0290	0,6088	instrument
rs836525	7	6465909	T	C	8,2E-05	32,233	0,0176	0,0031	9,8E-09	0,0203	0,0392	0,6042	instrument
rs879620	16	4015729	T	C	2,9E-04	115,328	0,0247	0,0023	5,4E-26	-0,0122	0,0298	0,6816	instrument
rs893006	11	64365796	A	C	1,2E-04	51,960	0,0173	0,0024	4,0E-13	0,0226	0,0318	0,4783	instrument
rs895330	19	4060707	C	G	9,9E-05	38,955	0,0181	0,0029	6,6E-10	-0,0217	0,0376	0,5649	instrument
rs915814	21	46493003	A	G	1,3E-04	34,810	-0,0177	0,0030	5,9E-09	0,0021	0,0340	0,9513	instrument
rs925018	3	62713143	C	G	8,8E-05	38,117	-0,0142	0,0023	9,8E-10	0,0044	0,0309	0,8869	instrument
rs9304665	19	47602577	A	T	1,4E-04	57,086	0,0204	0,0027	1,8E-14	-0,0209	0,0337	0,5352	instrument
rs9367369	6	13189941	A	G	1,4E-04	35,587	0,0173	0,0029	2,3E-09	0,0131	0,0315	0,6779	instrument
rs942066	14	94031914	A	G	2,1E-04	55,972	-0,0202	0,0027	1,0E-13	0,0561	0,0298	0,0594	instrument
rs943005	6	50865820	T	C	4,3E-04	188,351	0,0398	0,0029	2,0E-43	0,0903	0,0370	0,0147	instrument
rs9531786	13	85983968	C	G	8,4E-05	33,062	-0,0138	0,0024	8,4E-09	0,0130	0,0301	0,6666	instrument
rs9571687	13	67472713	A	C	9,2E-05	36,502	-0,0145	0,0024	3,2E-09	0,0406	0,0307	0,1859	instrument
rs9579775	13	20616557	A	C	1,5E-04	39,690	-0,0252	0,0040	3,7E-10	0,0315	0,0512	0,5382	instrument
rs9595630	13	33065443	T	G	9,2E-05	39,745	0,0145	0,0023	2,0E-10	-0,0202	0,0295	0,4939	instrument
rs973345	9	29713169	T	C	9,7E-05	38,117	0,0142	0,0023	1,1E-09	-0,0044	0,0292	0,8793	instrument
rs9906944	17	47091420	T	C	1,6E-04	62,673	-0,0190	0,0024	7,3E-15	-0,0382	0,0309	0,2168	instrument
rs9926784	16	19941968	T	C	1,9E-04	81,000	0,0252	0,0028	6,0E-20	0,0190	0,0370	0,6075	instrument
rs9937053	16	53799507	A	G	2,2E-03	941,370	0,0675	0,0022	1,0E-200	0,0169	0,0288	0,5572	instrument
rs994596	4	18459828	T	C	9,1E-05	36,000	0,0150	0,0025	1,0E-09	-0,0349	0,0308	0,2572	instrument
rs9955276	18	1839339	T	C	1,2E-04	48,129	0,0222	0,0032	8,0E-12	0,0376	0,0407	0,3550	instrument

Table S10. Single Nucleotide Polymorphisms (SNPs) used as instruments in the Mendelian randomization analyses for the effect of BMI on high or low serous ovarian cancer based on the genome-wide significance threshold P = 5E-08. R² represents the explained variance in the exposure. SNP-specific instrument strength is given by the respective F-statistic. Some of the genetic variants were identified as outliers in the iterative approach and therefore excluded from the analyses.

SNP	CHR	POS	EA	OA	R^2	F-stat	β_{BMI}	SE_{BMI}	P_{BMI}	$\beta_{High\ low\ serous}$	$S_{High\ low\ serous}$	$P_{High\ low\ serous}$	$variant_{High\ low\ serous}$
rs10002111	4	67815504	A	G	9,2E-05	36,430	0,0169	0,0028	9,3E-10	-0,0021	0,0183	0,9082	instrument
rs1003081	11	118913993	T	C	9,9E-05	42,843	0,0144	0,0022	6,3E-11	-0,0129	0,0156	0,4072	instrument
rs10048652	2	35449306	T	C	8,7E-05	34,452	0,0135	0,0023	3,5E-09	-0,0090	0,0154	0,5585	instrument
rs1009473	9	138777312	T	C	7,7E-05	30,489	-0,0127	0,0023	3,4E-08	-0,0174	0,0154	0,2577	instrument
rs10099330	8	143383694	A	G	8,3E-05	32,440	-0,0131	0,0023	9,9E-09	0,0112	0,0155	0,4701	instrument
rs10116186	9	80532374	A	G	1,2E-04	31,102	-0,0145	0,0026	4,1E-08	-0,0071	0,0155	0,6461	instrument
rs10118701	9	103061366	A	G	1,4E-04	53,778	-0,0176	0,0024	2,2E-13	0,0016	0,0162	0,9237	instrument
rs10124645	9	11321119	A	G	8,9E-05	34,964	0,0136	0,0023	5,0E-09	-0,0012	0,0159	0,9405	instrument
rs10182181	2	25150296	A	G	6,2E-04	270,751	-0,0362	0,0022	5,0E-63	-0,0032	0,0157	0,8391	instrument
rs10237317	7	70045941	A	G	9,1E-05	39,347	-0,0138	0,0022	6,8E-10	-0,0113	0,0159	0,4764	instrument
rs10271582	7	99121950	A	G	1,2E-04	32,222	0,0193	0,0034	8,4E-09	-0,0272	0,0211	0,1973	instrument
rs10471636	5	63979856	A	G	1,3E-04	50,173	-0,0170	0,0024	4,7E-13	0,0092	0,0161	0,5671	instrument
rs1048932	11	115044850	A	C	1,6E-04	68,438	-0,0182	0,0022	2,5E-16	0,0155	0,0155	0,3180	instrument
rs10499694	7	50614173	A	G	8,8E-05	38,215	0,0136	0,0022	4,0E-10	0,0249	0,0154	0,1047	instrument
rs10513801	3	185822353	T	G	2,8E-04	120,999	0,0352	0,0032	1,3E-27	0,0123	0,0231	0,5947	instrument
rs10733682	9	129460914	A	G	1,2E-04	51,578	0,0158	0,0022	1,9E-12	-0,0041	0,0154	0,7898	instrument
rs10742752	11	45438374	T	C	8,3E-05	36,000	-0,0132	0,0022	4,0E-09	-0,0108	0,0159	0,4961	instrument
rs10756714	9	15885041	A	G	2,1E-04	81,784	0,0208	0,0023	1,5E-19	0,0177	0,0154	0,2507	instrument
rs1075901	17	15943910	T	C	9,9E-05	42,843	-0,0144	0,0022	4,4E-11	-0,0227	0,0154	0,1422	instrument
rs10761785	10	65318766	T	G	1,4E-04	61,124	-0,0172	0,0022	4,2E-15	0,0039	0,0152	0,7991	instrument
rs10797115	9	92191256	T	C	1,2E-04	48,393	0,0160	0,0023	4,1E-12	-0,0133	0,0154	0,3864	instrument
rs10823893	10	53677313	A	G	1,6E-04	42,009	0,0175	0,0027	5,2E-11	-0,0077	0,0157	0,6223	instrument
rs10824347	10	77645229	A	T	1,1E-04	42,491	0,0176	0,0027	5,5E-11	-0,0084	0,0180	0,6404	instrument
rs10909880	1	2727804	T	C	7,5E-05	31,768	-0,0124	0,0022	2,0E-08	-0,0006	0,0155	0,9695	instrument
rs10920678	1	190239907	A	G	1,3E-04	54,894	0,0163	0,0022	7,5E-14	0,0029	0,0157	0,8525	instrument
rs10938397	4	45182527	A	G	4,7E-04	205,009	-0,0315	0,0022	1,7E-45	0,0174	0,0154	0,2590	instrument
rs10962550	9	16720329	C	G	1,0E-04	40,960	0,0192	0,0030	1,7E-10	0,0456	0,0207	0,0277	instrument
rs10992867	9	96461013	A	G	1,3E-04	50,628	0,0185	0,0026	8,9E-13	0,0152	0,0175	0,3853	instrument

rs11022762	11	13335926	T	C	1,0E-04	41,173	-0,0154	0,0024	5,6E-11	-0,0082	0,0157	0,6003	instrument
rs11073383	15	95274349	A	G	1,2E-04	47,790	-0,0159	0,0023	3,3E-12	-0,0048	0,0153	0,7527	instrument
rs11165643	1	96924097	T	C	1,6E-04	67,688	0,0181	0,0022	1,7E-16	0,0088	0,0158	0,5775	instrument
rs112646560	1	39560250	T	C	1,4E-04	37,515	0,0196	0,0032	1,1E-09	-0,0500	0,0192	0,0092	outlier
rs11667280	19	30705747	C	G	7,5E-05	29,684	0,0158	0,0029	3,1E-08	-0,0137	0,0200	0,4922	instrument
rs11672660	19	46180184	T	C	3,1E-04	131,429	-0,0321	0,0028	5,9E-31	-0,0157	0,0194	0,4185	instrument
rs11677607	2	100751150	T	C	2,0E-04	79,012	-0,0240	0,0027	2,9E-19	-0,0105	0,0179	0,5570	instrument
rs1167800	7	75176196	A	G	2,0E-04	82,644	0,0200	0,0022	3,7E-19	-0,0111	0,0156	0,4744	instrument
rs11692326	2	208263279	T	C	8,3E-05	36,000	0,0156	0,0026	2,0E-09	0,0084	0,0184	0,6467	instrument
rs11727273	4	55509748	T	C	1,6E-04	42,250	-0,0169	0,0026	1,3E-10	-0,0099	0,0157	0,5265	instrument
rs11751591	6	33794215	A	G	8,5E-05	33,423	-0,0185	0,0032	1,2E-08	0,0066	0,0217	0,7604	instrument
rs11754747	6	141494602	T	C	7,3E-05	31,532	0,0146	0,0026	1,3E-08	-0,0126	0,0186	0,4983	instrument
rs11880870	19	18830704	A	G	1,8E-04	73,363	0,0197	0,0023	3,3E-18	-0,0082	0,0156	0,5998	instrument
rs11945861	4	65700865	A	G	7,8E-05	30,864	-0,0150	0,0027	3,1E-08	0,0346	0,0181	0,0567	instrument
rs11971098	7	26699061	A	G	8,5E-05	33,414	-0,0237	0,0041	6,5E-09	0,0206	0,0272	0,4494	instrument
rs12042959	1	243533273	A	G	9,3E-05	36,754	0,0194	0,0032	2,3E-09	-0,0202	0,0217	0,3516	instrument
rs12089815	1	91189933	A	G	1,3E-04	34,628	-0,0153	0,0026	6,0E-09	-0,0270	0,0158	0,0876	instrument
rs12101625	15	26972431	C	G	8,4E-05	33,062	0,0138	0,0024	5,5E-09	-0,0234	0,0159	0,1412	instrument
rs12140153	1	62579891	T	G	1,8E-04	48,110	-0,0326	0,0047	2,9E-12	0,0111	0,0282	0,6948	instrument
rs12150665	17	34914787	T	C	1,3E-04	57,622	0,0167	0,0022	5,9E-14	-0,0110	0,0162	0,4947	instrument
rs12202969	6	98576223	A	G	1,0E-04	39,745	-0,0145	0,0023	1,9E-10	0,0184	0,0154	0,2328	instrument
rs12207125	6	154301575	A	G	1,3E-04	34,468	0,0182	0,0031	7,7E-09	-0,0171	0,0188	0,3621	instrument
rs12364470	11	134601012	T	G	1,0E-04	44,444	-0,0200	0,0030	2,5E-11	-0,0082	0,0227	0,7196	instrument
rs12429545	13	54102206	A	G	1,8E-04	77,760	0,0291	0,0033	1,1E-18	0,0347	0,0234	0,1379	instrument
rs12448257	16	3599655	A	G	7,2E-05	30,864	0,0150	0,0027	3,1E-08	0,0062	0,0192	0,7478	instrument
rs12462975	19	30272202	A	G	1,7E-04	65,934	0,0203	0,0025	2,6E-16	0,0476	0,0163	0,0034	outlier
rs12564992	1	174478100	A	G	8,0E-05	34,979	-0,0207	0,0035	2,4E-09	0,0511	0,0242	0,0346	instrument
rs12593036	15	81058652	A	G	9,6E-05	37,945	0,0154	0,0025	6,6E-10	0,0089	0,0168	0,5972	instrument
rs12612009	2	228982603	T	G	8,7E-05	34,515	-0,0141	0,0024	3,8E-09	-0,0113	0,0165	0,4926	instrument
rs12645001	4	96018337	A	G	8,7E-05	34,515	-0,0141	0,0024	4,1E-09	-0,0127	0,0161	0,4308	instrument
rs12680842	8	95582606	A	G	6,8E-05	29,340	0,0130	0,0024	3,4E-08	-0,0115	0,0163	0,4785	instrument
rs12681792	8	62054463	A	C	1,0E-04	39,820	0,0183	0,0029	2,6E-10	0,0143	0,0202	0,4792	instrument

rs12692596	2	161265910	T	C	9,3E-05	40,295	0,0146	0,0023	9,7E-11	0,0119	0,0160	0,4541	instrument
rs12692738	2	165558252	T	C	8,9E-05	38,822	-0,0162	0,0026	2,8E-10	0,0344	0,0181	0,0574	instrument
rs12714199	2	86812549	T	C	1,0E-04	40,295	-0,0146	0,0023	5,1E-10	0,0078	0,0159	0,6235	instrument
rs1285997	14	91513029	C	G	9,2E-05	36,000	-0,0150	0,0025	3,2E-09	-0,0228	0,0170	0,1789	instrument
rs12872889	13	28674628	T	C	1,7E-04	45,453	-0,0209	0,0031	1,5E-11	-0,0174	0,0183	0,3399	instrument
rs1289736	3	107950892	T	C	9,3E-05	36,523	0,0139	0,0023	1,4E-09	0,0239	0,0155	0,1231	instrument
rs12914489	15	74187937	A	G	9,1E-05	39,760	0,0227	0,0036	1,6E-10	0,0334	0,0248	0,1779	instrument
rs12949279	17	78558411	T	C	1,5E-04	66,200	0,0179	0,0022	6,1E-16	-0,0195	0,0157	0,2128	instrument
rs12981256	19	1865901	A	G	1,6E-04	62,616	0,0182	0,0023	3,9E-15	-0,0022	0,0156	0,8861	instrument
rs13002158	2	144021712	A	G	1,3E-04	51,443	-0,0208	0,0029	1,2E-12	0,0064	0,0203	0,7539	instrument
rs13053342	22	48871624	A	G	1,0E-04	40,640	0,0153	0,0024	1,3E-10	-0,0195	0,0173	0,2605	instrument
rs13062093	3	35667057	T	G	8,3E-05	32,585	-0,0137	0,0024	8,0E-09	-0,0268	0,0158	0,0898	instrument
rs13068138	3	85809425	T	C	1,7E-04	73,197	0,0231	0,0027	3,6E-17	0,0236	0,0191	0,2162	instrument
rs13076052	3	44456573	C	G	1,2E-04	32,766	-0,0166	0,0029	1,3E-08	0,0261	0,0175	0,1369	instrument
rs13107325	4	103188709	T	C	2,4E-04	105,528	0,0452	0,0044	9,3E-25	0,0455	0,0294	0,1210	instrument
rs13110266	4	162129844	A	G	9,6E-05	41,661	-0,0142	0,0022	1,5E-10	0,0068	0,0156	0,6632	instrument
rs13174863	5	139080745	A	G	1,8E-04	75,858	-0,0270	0,0031	4,5E-18	-0,0006	0,0227	0,9791	instrument
rs13209872	6	97753223	C	G	8,9E-05	35,007	-0,0142	0,0024	6,1E-09	-0,0076	0,0164	0,6452	instrument
rs13213408	6	162998049	A	T	9,0E-05	35,637	0,0197	0,0033	4,3E-09	0,0025	0,0225	0,9125	instrument
rs1362910	8	30856464	A	G	8,2E-05	33,943	0,0134	0,0023	3,0E-09	0,0024	0,0156	0,8755	instrument
rs1403846	4	119101723	T	C	7,2E-05	31,206	0,0162	0,0029	1,6E-08	0,0127	0,0201	0,5270	instrument
rs1409818	20	21381121	T	C	1,2E-04	46,387	0,0252	0,0037	1,6E-11	-0,0477	0,0256	0,0624	instrument
rs141729694	5	87999371	T	C	2,2E-04	57,284	0,0386	0,0051	2,5E-14	0,0097	0,0286	0,7357	instrument
rs1417665	6	104816944	T	C	1,2E-04	47,088	0,0199	0,0029	5,7E-12	-0,0233	0,0198	0,2400	instrument
rs1421598	2	50264710	A	C	9,7E-05	38,360	-0,0192	0,0031	6,8E-10	-0,0246	0,0208	0,2373	instrument
rs1436348	3	104612668	A	G	8,6E-05	33,943	-0,0134	0,0023	6,3E-09	-0,0107	0,0156	0,4926	instrument
rs1441264	13	79580919	A	G	1,3E-04	55,276	0,0171	0,0023	5,2E-14	0,0284	0,0161	0,0768	instrument
rs1452075	3	62481063	T	C	9,4E-05	40,449	0,0159	0,0025	1,5E-10	0,0214	0,0175	0,2195	instrument
rs1491333	4	172186288	T	G	8,1E-05	31,966	-0,0147	0,0026	9,9E-09	0,0118	0,0180	0,5098	instrument
rs1492014	3	94071481	T	C	1,0E-04	39,745	-0,0145	0,0023	2,8E-10	-0,0131	0,0154	0,3955	instrument
rs1518159	18	40760146	T	C	1,3E-04	52,841	-0,0189	0,0026	6,7E-13	0,0145	0,0176	0,4076	instrument
rs1561589	10	126695673	A	G	1,1E-04	47,191	0,0158	0,0023	4,2E-12	-0,0165	0,0158	0,2985	instrument

rs1569979	14	29681294	A	G	1,8E-04	72,565	0,0230	0,0027	1,6E-17	0,0259	0,0182	0,1544 instrument
rs1579557	6	40371918	T	C	1,8E-04	71,910	0,0212	0,0025	3,1E-17	0,0325	0,0172	0,0589 instrument
rs158186	5	107440856	A	G	2,2E-04	85,711	-0,0287	0,0031	3,2E-20	-0,0297	0,0203	0,1443 instrument
rs159032	5	94206202	T	C	7,8E-05	30,454	0,0149	0,0027	4,5E-08	0,0171	0,0177	0,3340 instrument
rs1681743	10	118565501	T	C	7,5E-05	29,793	-0,0131	0,0024	3,7E-08	-0,0183	0,0157	0,2429 instrument
rs16851483	3	141275436	T	G	1,6E-04	62,272	0,0363	0,0046	2,3E-15	0,0178	0,0309	0,5641 instrument
rs16912921	9	28413461	A	C	2,7E-04	117,204	0,0249	0,0023	1,6E-26	0,0320	0,0165	0,0522 instrument
rs16975921	18	39914870	A	T	1,1E-04	42,510	-0,0163	0,0025	3,9E-11	0,0014	0,0162	0,9292 instrument
rs16989232	20	39291784	A	G	7,1E-05	30,972	0,0128	0,0023	1,5E-08	0,0076	0,0161	0,6368 instrument
rs17014375	1	209543560	T	G	7,9E-05	31,228	-0,0190	0,0034	1,5E-08	0,0016	0,0223	0,9427 instrument
rs17066856	18	58049656	T	C	2,7E-04	115,707	0,0398	0,0037	1,4E-27	0,0306	0,0264	0,2475 instrument
rs17405819	8	76806584	T	C	1,9E-04	82,507	0,0218	0,0024	7,8E-20	-0,0211	0,0166	0,2037 instrument
rs1750307	1	156488420	A	T	8,3E-05	32,585	0,0137	0,0024	8,2E-09	0,0341	0,0159	0,0320 instrument
rs17522122	14	33302882	T	G	1,3E-04	57,622	0,0167	0,0022	6,5E-14	2,8E-05	0,0156	0,9986 instrument
rs17619973	3	114417675	A	G	7,4E-05	31,673	0,0242	0,0043	1,3E-08	-0,0589	0,0308	0,0557 instrument
rs17724992	19	18454825	A	G	1,1E-04	46,240	0,0170	0,0025	6,7E-12	0,0179	0,0174	0,3054 instrument
rs17751061	19	19413092	T	C	8,2E-05	31,993	-0,0181	0,0032	1,3E-08	0,0501	0,0208	0,0159 instrument
rs17767765	16	24539793	T	C	7,7E-05	30,489	-0,0127	0,0023	2,6E-08	0,0255	0,0159	0,1093 instrument
rs17806224	20	51065854	A	G	2,3E-04	92,801	-0,0289	0,0030	3,5E-22	0,0358	0,0202	0,0762 instrument
rs1799923	3	42306294	A	G	1,2E-04	48,203	-0,0243	0,0035	5,1E-12	0,0195	0,0253	0,4420 instrument
rs1801282	3	12393125	C	G	7,0E-05	30,417	-0,0182	0,0033	4,7E-08	-0,0152	0,0230	0,5079 instrument
rs1808579	18	21104888	T	C	1,9E-04	81,000	-0,0198	0,0022	9,4E-20	-0,0105	0,0154	0,4935 instrument
rs1808629	8	73435964	A	G	2,5E-04	65,147	-0,0226	0,0028	5,8E-16	0,0293	0,0168	0,0810 instrument
rs1812587	5	62991802	T	G	1,3E-04	34,177	0,0152	0,0026	7,0E-09	-0,0286	0,0154	0,0636 instrument
rs185350	19	34306816	T	C	7,8E-05	33,851	0,0128	0,0022	7,1E-09	-0,0229	0,0153	0,1358 instrument
rs1861412	2	58893065	A	G	2,2E-04	87,381	-0,0215	0,0023	9,1E-21	-0,0451	0,0154	0,0035 outlier
rs1884389	20	1410582	T	C	9,4E-05	37,051	-0,0140	0,0023	1,2E-09	0,0176	0,0154	0,2530 instrument
rs1884897	20	6612832	A	G	1,2E-04	53,990	-0,0169	0,0023	9,1E-14	0,0146	0,0160	0,3623 instrument
rs1916801	3	61187046	A	T	1,4E-04	59,710	0,0170	0,0022	1,8E-14	-0,0126	0,0157	0,4219 instrument
rs1927790	13	96922191	T	C	8,7E-05	37,655	-0,0135	0,0022	1,3E-09	-0,0286	0,0156	0,0674 instrument
rs1928295	9	120378483	T	C	1,2E-04	52,892	0,0160	0,0022	2,8E-13	0,0106	0,0153	0,4913 instrument
rs1949197	3	86273850	A	G	9,2E-05	36,523	0,0139	0,0023	3,0E-09	0,0069	0,0157	0,6615 instrument

rs197374	1	112289983	T	C	1,4E-04	55,276	0,0171	0,0023	2,8E-13	0,0205	0,0158	0,1950	instrument
rs1984559	2	135678888	A	G	7,9E-05	29,521	0,0163	0,0030	3,3E-08	0,0280	0,0186	0,1311	instrument
rs198665	6	143154886	A	C	7,8E-05	33,943	0,0134	0,0023	4,4E-09	-0,0166	0,0162	0,3041	instrument
rs2012502	16	81728081	A	C	1,0E-04	44,251	0,0153	0,0023	3,0E-11	0,0136	0,0161	0,3963	instrument
rs2045293	7	114350102	T	C	1,7E-04	67,525	0,0189	0,0023	4,1E-16	-0,0081	0,0157	0,6057	instrument
rs2053682	5	170599327	A	C	1,4E-04	53,582	0,0183	0,0025	1,9E-13	-0,0160	0,0164	0,3316	instrument
rs209416	6	83534729	A	T	1,2E-04	47,840	0,0166	0,0024	8,7E-12	0,0248	0,0165	0,1324	instrument
rs2112347	5	75015242	T	G	4,2E-04	180,493	0,0309	0,0023	2,1E-42	-0,0123	0,0158	0,4360	instrument
rs214249	16	348687	T	G	1,2E-04	48,418	0,0167	0,0024	1,4E-12	-0,0079	0,0154	0,6068	instrument
rs2161097	5	103945178	T	C	8,0E-05	31,457	0,0129	0,0023	2,3E-08	0,0063	0,0154	0,6805	instrument
rs2178899	6	31606756	A	T	1,3E-04	56,477	0,0248	0,0033	7,2E-14	-0,0354	0,0235	0,1320	instrument
rs2185027	6	153381622	A	C	9,5E-05	41,173	-0,0154	0,0024	1,1E-10	-0,0002	0,0166	0,9911	instrument
rs2228213	6	12124855	A	G	9,5E-05	41,406	-0,0148	0,0023	9,2E-11	-0,0024	0,0159	0,8803	instrument
rs2267812	7	74138121	A	C	9,2E-05	35,177	-0,0172	0,0029	1,7E-09	-0,0232	0,0186	0,2131	instrument
rs2267958	9	131015279	A	G	1,2E-04	45,002	-0,0161	0,0024	1,9E-11	-0,0227	0,0162	0,1606	instrument
rs2271046	4	52752812	A	T	7,6E-05	30,030	-0,0137	0,0025	2,4E-08	-0,0424	0,0170	0,0127	instrument
rs2289292	16	30097630	T	C	1,5E-04	57,760	-0,0190	0,0025	1,1E-14	0,0060	0,0170	0,7235	instrument
rs2289705	7	121983843	A	G	7,7E-05	33,284	-0,0150	0,0026	1,5E-08	-0,0128	0,0189	0,5010	instrument
rs2299383	7	103418846	T	C	1,6E-04	68,438	0,0182	0,0022	2,7E-16	-0,0133	0,0156	0,3944	instrument
rs2357760	6	120213880	A	G	1,1E-04	47,790	0,0159	0,0023	1,1E-11	-0,0220	0,0163	0,1775	instrument
rs2465043	6	51180765	A	G	8,9E-05	35,007	-0,0142	0,0024	3,2E-09	-0,0157	0,0161	0,3289	instrument
rs2470520	3	15762920	T	C	7,6E-05	30,011	-0,0126	0,0023	4,2E-08	0,0034	0,0156	0,8264	instrument
rs247975	3	173107443	T	C	1,6E-04	62,616	-0,0182	0,0023	3,6E-15	-0,0310	0,0161	0,0544	instrument
rs2479958	13	111984244	A	G	8,1E-05	31,640	0,0135	0,0024	1,4E-08	-0,0116	0,0158	0,4634	instrument
rs2528531	7	93236510	A	C	9,3E-05	36,502	-0,0145	0,0024	1,3E-09	0,0054	0,0160	0,7376	instrument
rs2568958	1	72765116	A	G	2,5E-04	106,464	0,0227	0,0022	1,3E-24	0,0051	0,0158	0,7460	instrument
rs2605603	11	93221105	A	G	8,3E-05	36,000	-0,0132	0,0022	1,4E-09	0,0345	0,0153	0,0245	instrument
rs2619976	17	71754545	T	C	9,4E-05	37,007	0,0146	0,0024	6,2E-10	0,0102	0,0163	0,5312	instrument
rs2721965	8	116662038	A	C	1,5E-04	60,062	0,0186	0,0024	2,1E-14	0,0331	0,0164	0,0433	instrument
rs273504	19	18215247	A	G	1,1E-04	43,102	-0,0151	0,0023	1,3E-10	0,0250	0,0156	0,1076	instrument
rs2754079	14	25886140	A	G	1,2E-04	46,004	-0,0156	0,0023	2,3E-11	0,0085	0,0158	0,5932	instrument
rs2814992	6	34617144	A	G	3,6E-04	155,706	-0,0287	0,0023	1,2E-35	-0,0246	0,0161	0,1251	instrument

rs2820295	1	201800868	A	G	2,0E-04	79,507	0,0214	0,0024	8,0E-19	-0,0292	0,0163	0,0740	instrument
rs2836754	21	40291740	T	C	1,1E-04	45,870	-0,0149	0,0022	3,7E-11	0,0108	0,0157	0,4912	instrument
rs28489620	22	41804716	A	G	1,5E-04	38,955	-0,0181	0,0029	6,9E-10	-0,0127	0,0169	0,4526	instrument
rs2861685	2	67837553	T	C	1,8E-04	47,929	0,0180	0,0026	1,1E-11	-0,0210	0,0155	0,1764	instrument
rs2862961	11	43656535	A	G	2,3E-04	91,393	-0,0239	0,0025	5,3E-22	-0,0196	0,0165	0,2332	instrument
rs2959592	8	17002725	T	C	8,7E-05	36,862	0,0170	0,0028	6,3E-10	-0,0213	0,0200	0,2868	instrument
rs2968864	7	150622162	T	C	9,2E-05	36,446	0,0163	0,0027	9,3E-10	-0,0031	0,0179	0,8623	instrument
rs2984618	1	47690438	T	G	1,3E-04	57,622	0,0167	0,0022	2,2E-14	-0,0055	0,0156	0,7275	instrument
rs329651	11	133767622	T	G	9,7E-05	41,787	0,0181	0,0028	2,0E-10	-0,0030	0,0199	0,8797	instrument
rs337637	4	38604470	A	G	6,8E-05	29,537	-0,0125	0,0023	3,0E-08	-0,0092	0,0161	0,5671	instrument
rs34517439	1	78450517	A	C	3,4E-04	90,481	0,0390	0,0041	2,0E-21	0,0075	0,0249	0,7636	instrument
rs34811474	4	25408838	A	G	4,0E-04	104,566	-0,0317	0,0031	5,2E-24	-0,0216	0,0212	0,3093	instrument
rs34930419	10	33987329	T	C	1,4E-04	36,316	-0,0458	0,0076	1,4E-09	0,0592	0,0465	0,2033	instrument
rs35107973	4	143125948	T	C	1,2E-04	32,573	0,0234	0,0041	7,7E-09	0,0020	0,0247	0,9343	instrument
rs355754	3	154024165	T	C	9,2E-05	38,117	-0,0142	0,0023	4,3E-10	0,0095	0,0158	0,5475	instrument
rs35722922	2	47002226	A	G	1,8E-04	48,482	0,0188	0,0027	3,6E-12	0,0026	0,0160	0,8727	instrument
rs3803286	14	103246470	A	G	1,3E-04	51,361	0,0172	0,0024	8,3E-13	0,0033	0,0161	0,8366	instrument
rs3806114	6	20482335	A	G	8,0E-05	34,515	-0,0141	0,0024	4,5E-09	-0,0209	0,0166	0,2096	instrument
rs3826408	17	7101292	T	C	7,0E-05	30,250	0,0121	0,0022	4,5E-08	-0,0117	0,0156	0,4510	instrument
rs3829849	9	129390800	T	C	8,0E-05	34,964	0,0136	0,0023	1,9E-09	-0,0060	0,0159	0,7045	instrument
rs3977755	10	104420210	T	C	8,0E-05	32,718	-0,0143	0,0025	8,5E-09	-0,0174	0,0171	0,3080	instrument
rs4055791	13	59266053	T	C	1,5E-04	39,643	-0,0170	0,0027	1,8E-10	0,0200	0,0157	0,2029	instrument
rs4148155	4	89054667	A	G	7,7E-05	33,640	0,0203	0,0035	6,2E-09	-0,0064	0,0246	0,7961	instrument
rs4240673	8	10787612	T	C	1,5E-04	64,000	0,0176	0,0022	1,0E-15	0,0060	0,0201	0,7651	instrument
rs427943	21	46570896	A	C	1,5E-04	59,223	-0,0177	0,0023	1,4E-14	-0,0228	0,0157	0,1459	instrument
rs429358	19	45411941	T	C	2,2E-04	58,777	0,0276	0,0036	3,2E-14	0,0070	0,0223	0,7544	instrument
rs4295917	1	32173360	A	G	8,2E-05	31,640	0,0135	0,0024	1,6E-08	0,0026	0,0165	0,8763	instrument
rs4307239	7	24354300	A	G	9,5E-05	37,582	-0,0141	0,0023	1,3E-09	-0,0223	0,0157	0,1536	instrument
rs4320543	8	34422967	A	G	8,0E-05	31,457	-0,0129	0,0023	2,9E-08	0,0178	0,0160	0,2686	instrument
rs4421623	1	98316285	T	G	1,2E-04	46,441	0,0184	0,0027	1,1E-11	-0,0215	0,0184	0,2430	instrument
rs4459316	11	130756176	T	C	1,1E-04	44,832	-0,0154	0,0023	2,3E-11	0,0062	0,0155	0,6921	instrument
rs4474229	1	195037001	A	G	1,6E-04	41,055	-0,0173	0,0027	1,6E-10	-0,0004	0,0157	0,9806	instrument

rs4482463	2	205375909	A	C	2,0E-04	75,524	-0,0365	0,0042	5,2E-18	0,0804	0,0293	0,0061	outlier
rs4569924	5	153540025	T	C	1,2E-04	51,578	0,0158	0,0022	7,4E-13	0,0326	0,0155	0,0356	instrument
rs4586287	13	99245334	C	G	7,7E-05	30,470	0,0138	0,0025	2,9E-08	-0,0078	0,0168	0,6399	instrument
rs4673553	2	211608379	T	G	1,1E-04	44,251	-0,0153	0,0023	2,6E-11	0,0055	0,0155	0,7243	instrument
rs4677812	3	194863794	A	C	8,4E-05	33,177	-0,0144	0,0025	9,4E-09	-0,0081	0,0172	0,6379	instrument
rs4776982	15	68114974	A	G	3,3E-04	143,078	0,0311	0,0026	4,2E-33	0,0211	0,0183	0,2505	instrument
rs4783718	16	69547741	T	C	1,8E-04	78,669	0,0204	0,0023	1,8E-19	0,0228	0,0161	0,1580	instrument
rs4790292	17	1824305	A	C	1,7E-04	63,516	-0,0263	0,0033	1,7E-15	0,0250	0,0228	0,2729	instrument
rs4834272	4	113313986	T	C	8,9E-05	38,656	-0,0143	0,0023	8,2E-10	-0,0258	0,0167	0,1217	instrument
rs4858223	3	20581149	T	C	1,1E-04	41,473	-0,0161	0,0025	8,3E-11	0,0118	0,0166	0,4755	instrument
rs4864201	4	130731284	T	C	7,5E-05	32,440	0,0131	0,0023	6,3E-09	-0,0077	0,0160	0,6305	instrument
rs4880341	10	133992689	T	C	9,8E-05	38,656	-0,0143	0,0023	4,5E-10	-0,0095	0,0154	0,5364	instrument
rs4889606	16	31011183	A	G	3,1E-04	135,773	0,0268	0,0023	1,3E-32	0,0116	0,0159	0,4668	instrument
rs4929923	11	8639200	T	C	2,1E-04	92,327	-0,0221	0,0023	2,5E-22	-0,0420	0,0159	0,0081	instrument
rs4936175	11	132641959	T	C	1,2E-04	47,191	-0,0158	0,0023	6,7E-12	0,0104	0,0154	0,4996	instrument
rs4969387	17	79081724	C	G	8,4E-05	32,532	-0,0154	0,0027	6,9E-09	0,0063	0,0177	0,7220	instrument
rs4986044	17	21261560	T	C	1,4E-04	61,836	-0,0173	0,0022	2,2E-15	-0,0075	0,0153	0,6249	instrument
rs543874	1	177889480	A	G	1,0E-03	455,109	-0,0576	0,0027	8,7E-100	0,0108	0,0195	0,5791	instrument
rs56161855	17	46288649	A	T	1,7E-04	44,787	-0,0261	0,0039	1,5E-11	0,0263	0,0223	0,2396	instrument
rs563132	9	37245476	A	T	1,4E-04	56,250	0,0180	0,0024	2,0E-14	-0,0083	0,0157	0,5974	instrument
rs563296	10	99772404	A	G	1,4E-04	53,168	0,0175	0,0024	8,8E-14	-0,0081	0,0156	0,6053	instrument
rs57221746	15	99230035	A	G	1,2E-04	30,594	-0,0177	0,0032	2,1E-08	-0,0023	0,0184	0,9004	instrument
rs57800857	4	140863365	A	C	1,8E-04	47,456	0,0186	0,0027	1,1E-11	0,0118	0,0159	0,4598	instrument
rs6010784	20	61540319	T	C	7,6E-05	32,802	0,0126	0,0022	1,1E-08	0,0124	0,0153	0,4185	instrument
rs6050446	20	25195509	A	G	8,0E-05	33,785	-0,0372	0,0064	6,2E-09	-0,0431	0,0476	0,3652	instrument
rs6142096	20	32686658	A	G	8,6E-05	33,943	0,0134	0,0023	4,2E-09	0,0012	0,0152	0,9394	instrument
rs61813324	1	156049877	T	C	2,2E-04	58,385	0,0298	0,0039	3,6E-14	0,0215	0,0247	0,3839	instrument
rs62007782	15	78029797	A	G	1,4E-04	35,601	-0,0179	0,0030	1,9E-09	0,0045	0,0176	0,7957	instrument
rs62106258	2	417167	T	C	9,7E-04	254,452	0,0989	0,0062	8,7E-58	0,0130	0,0414	0,7538	instrument
rs62412826	6	61881167	T	C	1,3E-04	33,518	0,0330	0,0057	8,0E-09	-0,0777	0,0340	0,0223	instrument
rs625652	11	65641931	A	T	2,0E-04	52,160	-0,0195	0,0027	9,1E-13	0,0013	0,0160	0,9367	instrument
rs6265	11	27679916	T	C	4,0E-04	172,734	-0,0368	0,0028	8,0E-40	0,0021	0,0193	0,9132	instrument

rs6446187	3	49907111	A	C	4,3E-04	114,325	0,0278	0,0026	1,2E-26	0,0063	0,0152	0,6786	instrument
rs6493498	15	51754451	T	C	1,3E-04	54,894	0,0163	0,0022	1,5E-13	0,0133	0,0155	0,3896	instrument
rs6548237	2	621461	A	C	9,1E-04	394,500	-0,0576	0,0029	4,4E-89	-0,0096	0,0255	0,7076	instrument
rs6556810	5	164585949	T	C	7,4E-05	29,066	-0,0124	0,0023	5,0E-08	-0,0132	0,0156	0,3996	instrument
rs6561937	13	58257667	A	T	1,4E-04	53,236	-0,0197	0,0027	6,0E-13	0,0119	0,0184	0,5177	instrument
rs6567160	18	57829135	T	C	1,1E-03	475,573	-0,0567	0,0026	8,7E-108	0,0171	0,0180	0,3433	instrument
rs657452	1	49589847	A	G	2,0E-04	85,142	0,0203	0,0022	1,1E-19	0,0045	0,0156	0,7733	instrument
rs6585198	10	114762237	A	G	1,1E-04	49,000	-0,0154	0,0022	1,2E-12	-0,0295	0,0153	0,0531	instrument
rs6669189	1	75011358	T	C	2,1E-04	91,115	0,0210	0,0022	4,7E-21	0,0129	0,0155	0,4069	instrument
rs6720868	2	230663576	T	C	1,3E-04	49,585	0,0169	0,0024	4,6E-12	0,0104	0,0162	0,5208	instrument
rs6728037	2	59297346	A	G	2,1E-04	81,000	0,0207	0,0023	6,5E-19	0,0322	0,0157	0,0405	instrument
rs6739199	2	105452326	T	C	1,0E-04	40,449	0,0159	0,0025	3,2E-10	0,0230	0,0176	0,1922	instrument
rs6870983	5	87697533	T	C	1,5E-04	66,485	-0,0212	0,0026	9,6E-16	0,0172	0,0182	0,3449	instrument
rs6890310	5	27193573	A	G	9,6E-05	37,945	-0,0154	0,0025	1,4E-09	0,0248	0,0174	0,1532	instrument
rs6973700	7	1856669	A	G	1,4E-04	36,376	0,0193	0,0032	1,8E-09	0,0129	0,0187	0,4911	instrument
rs7024334	9	109072075	T	G	8,7E-05	37,398	0,0159	0,0026	1,6E-09	-0,0066	0,0183	0,7186	instrument
rs7084454	10	21821274	A	G	2,1E-04	81,000	0,0225	0,0025	7,4E-20	0,0985	0,0161	9,5E-10	outlier
rs709400	14	104149475	A	G	1,5E-04	64,000	0,0184	0,0023	4,4E-16	-0,0137	0,0157	0,3813	instrument
rs7105462	11	112912048	A	G	9,9E-05	39,198	0,0144	0,0023	4,4E-10	-0,0192	0,0155	0,2164	instrument
rs7107409	11	76492195	T	C	1,2E-04	30,941	-0,0178	0,0032	2,1E-08	0,0156	0,0183	0,3946	instrument
rs7124681	11	47529947	A	C	2,9E-04	126,051	0,0247	0,0022	9,3E-29	0,0170	0,0156	0,2750	instrument
rs7141420	14	79899454	T	C	2,1E-04	91,115	0,0210	0,0022	7,8E-22	-0,0151	0,0158	0,3403	instrument
rs7211966	17	54374611	T	C	8,9E-05	34,515	-0,0470	0,0080	4,9E-09	0,1346	0,0646	0,0373	instrument
rs7239114	18	45921214	A	G	1,2E-04	49,638	0,0155	0,0022	3,9E-12	-0,0066	0,0157	0,6768	instrument
rs7250833	19	33937277	T	C	8,9E-05	38,543	0,0149	0,0024	7,2E-10	0,0010	0,0170	0,9535	instrument
rs73124396	7	71579606	T	C	1,2E-04	32,111	0,0187	0,0033	1,1E-08	-0,0036	0,0188	0,8459	instrument
rs73213484	4	28489339	A	T	1,7E-04	45,653	0,0250	0,0037	2,3E-11	-0,0330	0,0214	0,1219	instrument
rs7335249	13	109962579	T	C	8,7E-05	34,452	0,0135	0,0023	4,9E-09	0,0230	0,0157	0,1429	instrument
rs7395632	11	958789	T	C	7,4E-05	29,340	-0,0130	0,0024	3,9E-08	-0,0034	0,0159	0,8310	instrument
rs7428670	3	131579810	C	G	1,5E-04	60,361	0,0202	0,0026	3,1E-15	0,0314	0,0172	0,0687	instrument
rs7498044	15	92573639	A	G	1,2E-04	31,640	-0,0180	0,0032	2,7E-08	-0,0087	0,0195	0,6547	instrument
rs7498665	16	28883241	A	G	3,3E-04	143,999	-0,0264	0,0022	1,9E-32	0,0052	0,0157	0,7403	instrument

rs750090	4	152931436	T	C	1,1E-04	42,250	0,0156	0,0024	7,4E-11	-0,0041	0,0164	0,8030 instrument
rs7550711	1	110082886	T	C	2,1E-04	88,858	0,0641	0,0068	3,4E-21	0,1329	0,0441	0,0026 outlier
rs7571496	2	6169351	A	G	7,9E-05	31,102	0,0145	0,0026	1,4E-08	-0,0008	0,0176	0,9654 instrument
rs7588437	2	181575281	A	G	1,3E-04	58,555	-0,0176	0,0023	8,4E-15	-0,0263	0,0159	0,0971 instrument
rs7594237	2	198187164	A	T	8,3E-05	32,718	-0,0143	0,0025	1,8E-08	-0,0087	0,0176	0,6187 instrument
rs7601870	2	15384392	A	G	8,3E-05	32,937	-0,0132	0,0023	1,2E-08	-0,0128	0,0156	0,4125 instrument
rs7601895	2	55281901	C	G	1,1E-04	41,473	0,0161	0,0025	9,6E-11	-0,0033	0,0170	0,8463 instrument
rs7621025	3	136272246	T	C	1,5E-04	64,000	-0,0200	0,0025	1,6E-15	-0,0015	0,0175	0,9325 instrument
rs7647242	3	82642734	T	C	1,0E-04	39,745	0,0145	0,0023	3,9E-10	0,0109	0,0158	0,4878 instrument
rs768023	6	108876002	A	G	1,6E-04	70,712	0,0185	0,0022	1,4E-16	-0,0076	0,0157	0,6285 instrument
rs76900466	2	145057760	T	G	1,2E-04	30,654	0,0227	0,0041	4,5E-08	0,0078	0,0293	0,7888 instrument
rs77432547	13	86494817	A	G	1,4E-04	36,415	-0,0175	0,0029	2,3E-09	0,0046	0,0172	0,7913 instrument
rs7788008	7	112972483	A	G	1,4E-04	55,276	-0,0171	0,0023	1,1E-13	0,0004	0,0154	0,9786 instrument
rs7805441	7	78121458	T	C	1,0E-04	40,295	0,0146	0,0023	2,0E-10	-0,0148	0,0153	0,3342 instrument
rs782971	5	43124688	A	G	1,1E-04	47,334	-0,0172	0,0025	4,9E-12	0,0004	0,0179	0,9824 instrument
rs786420	2	44719893	T	C	8,4E-05	32,956	0,0155	0,0027	8,8E-09	-0,0060	0,0178	0,7346 instrument
rs78886584	1	16859325	A	G	1,3E-04	33,284	-0,0150	0,0026	1,3E-08	0,0346	0,0174	0,0465 instrument
rs7899106	10	87410904	A	G	1,3E-04	56,250	-0,0375	0,0050	7,6E-14	-0,0384	0,0365	0,2919 instrument
rs79113395	1	1590521	A	G	1,9E-04	49,000	-0,0210	0,0030	1,5E-12	0,0046	0,0223	0,8383 instrument
rs8024806	15	53473990	T	C	8,5E-05	33,492	0,0272	0,0047	9,8E-09	0,0163	0,0321	0,6121 instrument
rs8033510	15	61445514	T	C	8,3E-05	32,585	0,0137	0,0024	1,1E-08	-0,0010	0,0158	0,9489 instrument
rs8038522	15	46585105	T	G	9,4E-05	37,051	-0,0140	0,0023	1,3E-09	0,0218	0,0155	0,1592 instrument
rs8075273	17	61728881	A	C	1,0E-04	44,444	-0,0160	0,0024	5,6E-11	0,0316	0,0171	0,0642 instrument
rs811054	16	72251132	T	C	1,2E-04	54,223	0,0162	0,0022	3,2E-13	0,0144	0,0155	0,3535 instrument
rs836525	7	6465909	T	C	8,2E-05	32,233	0,0176	0,0031	9,8E-09	0,0190	0,0209	0,3640 instrument
rs879620	16	4015729	T	C	2,9E-04	115,328	0,0247	0,0023	5,4E-26	-0,0009	0,0159	0,9524 instrument
rs893006	11	64365796	A	C	1,2E-04	51,960	0,0173	0,0024	4,0E-13	0,0269	0,0169	0,1118 instrument
rs895330	19	4060707	C	G	9,9E-05	38,955	0,0181	0,0029	6,6E-10	-0,0191	0,0201	0,3403 instrument
rs915814	21	46493003	A	G	1,3E-04	34,810	-0,0177	0,0030	5,9E-09	-0,0055	0,0181	0,7630 instrument
rs925018	3	62713143	C	G	8,8E-05	38,117	-0,0142	0,0023	9,8E-10	-0,0308	0,0164	0,0604 instrument
rs9304665	19	47602577	A	T	1,4E-04	57,086	0,0204	0,0027	1,8E-14	-0,0043	0,0180	0,8123 instrument
rs9367369	6	13189941	A	G	1,4E-04	35,587	0,0173	0,0029	2,3E-09	0,0305	0,0168	0,0703 instrument

rs942066	14	94031914	A	G	2,1E-04	55,972	-0,0202	0,0027	1,0E-13	-0,0152	0,0160	0,3409	instrument
rs943005	6	50865820	T	C	4,3E-04	188,351	0,0398	0,0029	2,0E-43	-0,0022	0,0201	0,9122	instrument
rs9531786	13	85983968	C	G	8,4E-05	33,062	-0,0138	0,0024	8,4E-09	-0,0103	0,0160	0,5220	instrument
rs9571687	13	67472713	A	C	9,2E-05	36,502	-0,0145	0,0024	3,2E-09	0,0069	0,0164	0,6717	instrument
rs9579775	13	20616557	A	C	1,5E-04	39,690	-0,0252	0,0040	3,7E-10	0,0542	0,0276	0,0492	instrument
rs9595630	13	33065443	T	G	9,2E-05	39,745	0,0145	0,0023	2,0E-10	0,0157	0,0158	0,3204	instrument
rs973345	9	29713169	T	C	9,7E-05	38,117	0,0142	0,0023	1,1E-09	0,0065	0,0156	0,6749	instrument
rs9906944	17	47091420	T	C	1,6E-04	62,673	-0,0190	0,0024	7,3E-15	-0,0208	0,0165	0,2077	instrument
rs9926784	16	19941968	T	C	1,9E-04	81,000	0,0252	0,0028	6,0E-20	0,0177	0,0197	0,3683	instrument
rs9937053	16	53799507	A	G	2,2E-03	941,370	0,0675	0,0022	1,0E-200	0,0077	0,0153	0,6159	instrument
rs994596	4	18459828	T	C	9,1E-05	36,000	0,0150	0,0025	1,0E-09	-0,0372	0,0164	0,0231	instrument
rs9955276	18	1839339	T	C	1,2E-04	48,129	0,0222	0,0032	8,0E-12	-0,0156	0,0219	0,4776	instrument

Table S11. Single Nucleotide Polymorphisms (SNPs) used as instruments in the Mendelian randomization analyses for the effect of TFR on breast cancer based on the genome-wide significance threshold P = 5E-08. R^2 represents the explained variance in the exposure. SNP-specific instrument strength is given by the respective F-statistic. Some of the genetic variants were identified as outliers in the iterative approach and therefore excluded from the analyses.

SNP	CHR	POS	EA	OA	R^2	F-stat	β_{TFR}	SE_{TFR}	P_{TFR}	β_{Breast}	SE_{Breast}	P_{breast}	$variant_{Breast}$
rs1002436	1	89146852	A	G	0,0003	48,0799	-0,0225	0,0032	4,1E-12	-0,0172	0,0063	0,0063	instrument
rs10059884	5	32832474	C	A	0,0002	45,7106	0,0222	0,0033	1,4E-11	-0,0063	0,0067	0,3461	instrument
rs10152591	15	70048157	C	A	0,0003	52,0701	-0,0379	0,0053	5,4E-13	0,0056	0,0107	0,6014	instrument
rs10179126	2	165511794	G	C	0,0002	41,1261	0,0215	0,0034	1,4E-10	-0,0080	0,0064	0,2081	instrument
rs1020048	9	86666993	A	C	0,0003	54,2721	0,0308	0,0042	1,8E-13	0,0077	0,0079	0,3258	instrument
rs1026029	2	177065248	C	T	0,0002	32,7295	-0,0409	0,0071	1,1E-08	0,0037	0,0145	0,7954	instrument
rs10760110	9	123412563	C	T	0,0002	33,6745	0,0204	0,0035	6,5E-09	0,0045	0,0069	0,5207	instrument
rs10761129	9	94486321	C	T	0,0002	41,9640	0,0222	0,0034	9,3E-11	0,0035	0,0072	0,6260	instrument
rs10823149	10	69935396	G	T	0,0002	38,5513	-0,0202	0,0032	5,3E-10	-0,0024	0,0062	0,6939	instrument
rs10874671	1	103456389	G	C	0,0003	53,4209	0,0257	0,0035	2,7E-13	-0,0059	0,0067	0,3798	instrument
rs10901171	9	133427194	C	T	0,0003	52,6200	-0,0242	0,0033	4,1E-13	-0,0023	0,0068	0,7416	instrument
rs10933385	2	232916068	A	G	0,0003	57,3043	-0,0910	0,0120	3,7E-14	0,0250	0,0221	0,2585	instrument
rs11031005	11	30226356	C	T	0,0002	30,8244	0,0256	0,0046	2,8E-08	0,0120	0,0088	0,1698	instrument
rs11130321	3	52762698	T	C	0,0003	60,2015	-0,0255	0,0033	8,6E-15	-0,0080	0,0063	0,1990	instrument
rs11186507	10	92983831	G	A	0,0002	40,4873	0,0257	0,0040	2,0E-10	0,0007	0,0080	0,9316	instrument

rs111947781	3	38021115	T	C	0,0002	42,4187	0,0290	0,0045	7,4E-11	0,0012	0,0089	0,8911	instrument
rs11205303	1	149906413	C	T	0,0020	392,0359	0,0648	0,0033	3,6E-87	0,0497	0,0064	1,1E-14	outlier
rs11259936	15	84580582	A	C	0,0017	324,7170	-0,0581	0,0032	1,6E-72	0,0059	0,0062	0,3413	instrument
rs112635299	14	94838142	T	G	0,0005	93,4886	0,1081	0,0112	4,1E-22	-0,0562	0,0242	0,0203	outlier
rs113059026	10	32158478	A	C	0,0002	38,8499	-0,0223	0,0036	4,6E-10	-0,0213	0,0068	0,0019	outlier
rs115912456	5	82815158	G	A	0,0005	86,3032	-0,0753	0,0081	1,6E-20	-0,0122	0,0162	0,4488	instrument
rs11689727	2	25458100	A	C	0,0006	106,5013	-0,0354	0,0034	6,0E-25	-0,0178	0,0070	0,0111	instrument
rs11717301	3	72397284	A	C	0,0003	58,9818	-0,0289	0,0038	1,6E-14	-0,0140	0,0074	0,0579	instrument
rs12129970	1	54877581	G	A	0,0002	34,2690	-0,0307	0,0052	4,8E-09	-0,0004	0,0117	0,9711	instrument
rs12172195	22	45813687	A	G	0,0003	50,8649	0,0324	0,0045	9,9E-13	0,0088	0,0088	0,3191	instrument
rs12409899	1	172227011	G	C	0,0004	85,0260	0,0363	0,0039	3,0E-20	-0,0084	0,0076	0,2666	instrument
rs12507561	4	73464895	T	G	0,0007	127,2371	0,0365	0,0032	1,8E-29	0,0075	0,0065	0,2503	instrument
rs12685003	9	17022667	G	A	0,0002	34,9514	-0,0304	0,0051	3,4E-09	-0,0058	0,0103	0,5749	instrument
rs12756815	1	113207243	A	C	0,0004	76,3518	-0,0349	0,0040	2,4E-18	-0,0059	0,0077	0,4400	instrument
rs13107395	4	8601846	C	T	0,0003	62,2988	-0,0258	0,0033	3,0E-15	-0,0062	0,0068	0,3587	instrument
rs13128180	4	56286188	T	G	0,0003	51,6668	0,0253	0,0035	6,6E-13	0,0055	0,0067	0,4080	instrument
rs1313237	14	60848224	C	A	0,0002	46,4710	0,0226	0,0033	9,3E-12	-0,0008	0,0064	0,9020	instrument
rs13144767	4	17974219	G	A	0,0004	68,3922	-0,0395	0,0048	1,3E-16	-0,0070	0,0092	0,4503	instrument
rs13170063	5	157895013	G	A	0,0002	45,5485	-0,0227	0,0034	1,5E-11	-0,0115	0,0065	0,0765	instrument
rs13306455	19	7184721	T	C	0,0002	37,7115	0,0264	0,0043	8,2E-10	-0,0021	0,0083	0,8001	instrument
rs13429301	2	242061508	C	A	0,0002	32,8326	0,0330	0,0058	1,0E-08	-0,0004	0,0114	0,9749	instrument
rs138809270	2	219929394	T	C	0,0003	52,6925	-0,0952	0,0131	3,9E-13	-0,0136	0,0247	0,5800	instrument
rs1388900	16	73800629	T	C	0,0002	33,4774	-0,0208	0,0036	7,2E-09	-0,0010	0,0071	0,8914	instrument
rs1421896	5	141016288	T	G	0,0002	30,5032	0,0183	0,0033	3,3E-08	0,0018	0,0065	0,7866	instrument
rs142599545	6	19844384	G	A	0,0002	37,4540	0,0812	0,0133	9,4E-10	0,0044	0,0366	0,9035	instrument
rs143384	20	34025756	G	A	0,0011	215,2067	0,0482	0,0033	1,1E-48	-0,0020	0,0064	0,7561	instrument
rs143840904	11	2813322	T	C	0,0003	60,4811	-0,0978	0,0126	7,4E-15	-0,0224	0,0321	0,4851	instrument
rs1452815	8	78123634	G	A	0,0002	35,6764	0,0213	0,0036	2,3E-09	0,0195	0,0068	0,0045	outlier
rs1513304	3	99269750	C	T	0,0002	44,5418	0,0236	0,0035	2,5E-11	0,0273	0,0073	0,0002	outlier
rs1528589	5	88748768	C	T	0,0002	43,3617	0,0222	0,0034	4,6E-11	0,0027	0,0065	0,6817	instrument
rs1550436	15	74221157	T	C	0,0006	111,7237	-0,0343	0,0032	4,0E-26	0,0127	0,0062	0,0400	outlier

rs16942324	15	89383854	A	C	0,0005	104,6518	-0,1025	0,0100	1,4E-24	0,0232	0,0230	0,3129	instrument
rs16953461	17	46261736	G	T	0,0003	62,7574	-0,0312	0,0039	2,3E-15	0,0064	0,0076	0,3970	instrument
rs16996444	19	9997238	C	T	0,0002	47,0866	-0,0245	0,0036	6,8E-12	0,0065	0,0069	0,3496	instrument
rs17194431	8	120612627	T	C	0,0002	29,9206	0,0196	0,0036	4,5E-08	-0,0028	0,0070	0,6866	instrument
rs1749824	10	80923862	A	C	0,0002	32,5011	-0,0185	0,0032	1,2E-08	0,0133	0,0063	0,0338	instrument
rs17511102	2	37960613	T	A	0,0002	34,6446	0,0331	0,0056	4,0E-09	0,0004	0,0126	0,9773	instrument
rs17654949	2	219890955	T	C	0,0003	51,3940	0,0617	0,0086	7,6E-13	0,0366	0,0174	0,0353	instrument
rs1805007	16	89986117	T	C	0,0003	56,8359	-0,0395	0,0052	4,7E-14	-0,0167	0,0120	0,1631	instrument
rs194059	5	56197416	G	A	0,0003	48,9575	-0,0238	0,0034	2,6E-12	0,1027	0,0064	0,0000	outlier
rs1952256	1	184035116	G	A	0,0006	106,9145	0,0351	0,0034	4,6E-25	0,0102	0,0065	0,1185	instrument
rs1966265	5	176516631	A	G	0,0003	51,8251	0,0268	0,0037	6,1E-13	-0,0201	0,0074	0,0064	outlier
rs1980030	10	120970027	G	A	0,0003	47,7476	0,0227	0,0033	4,8E-12	0,0026	0,0066	0,6889	instrument
rs2006122	17	61987405	T	A	0,0003	47,1827	0,0252	0,0037	6,5E-12	0,0086	0,0070	0,2208	instrument
rs200889152	6	117507982	C	A	0,0002	42,3146	-0,0218	0,0034	7,8E-11	0,0059	0,0065	0,3653	instrument
rs2035901	4	145521867	G	A	0,0002	30,3267	0,0179	0,0032	3,7E-08	0,0037	0,0065	0,5709	instrument
rs2050157	6	142658162	A	G	0,0008	146,8929	-0,0434	0,0036	8,4E-34	0,0007	0,0068	0,9129	instrument
rs2076112	22	38538822	T	C	0,0003	64,3999	-0,0261	0,0032	1,0E-15	0,0332	0,0062	7,3E-08	outlier
rs2194411	3	185548663	A	G	0,0002	42,9020	0,0324	0,0049	5,8E-11	-0,0001	0,0103	0,9913	instrument
rs239458	8	37213496	G	A	0,0002	29,8441	-0,0315	0,0058	4,7E-08	0,0012	0,0112	0,9160	instrument
rs256904	5	55810305	A	T	0,0005	87,0667	0,0345	0,0037	1,1E-20	-0,0041	0,0075	0,5811	instrument
rs264762	5	64384386	A	C	0,0003	53,4648	-0,0250	0,0034	2,6E-13	0,0205	0,0066	0,0018	outlier
rs2712169	2	217671349	G	A	0,0002	44,7289	0,0221	0,0033	2,3E-11	0,0140	0,0063	0,0274	instrument
rs2722285	7	37994955	T	C	0,0002	32,0466	-0,0286	0,0051	1,5E-08	0,0168	0,0101	0,0982	instrument
rs2731340	3	13559829	C	T	0,0002	47,4440	0,0370	0,0054	5,7E-12	-0,0082	0,0107	0,4404	instrument
rs273945	7	137611566	A	C	0,0002	38,3157	-0,0202	0,0033	6,0E-10	-0,0042	0,0063	0,5007	instrument
rs2763265	6	168814600	G	A	0,0002	39,0371	-0,0239	0,0038	4,2E-10	0,0124	0,0072	0,0846	instrument
rs2797963	6	34185749	C	T	0,0005	88,1148	0,0556	0,0059	6,2E-21	-0,0164	0,0118	0,1669	instrument
rs2811462	3	129303436	G	T	0,0003	53,0561	0,0282	0,0039	3,2E-13	0,0225	0,0077	0,0035	outlier
rs2820443	1	219753509	C	T	0,0006	109,8293	0,0369	0,0035	1,0E-25	-0,0182	0,0069	0,0089	outlier
rs2823989	21	18080943	G	A	0,0002	32,8326	-0,0197	0,0034	1,0E-08	0,0003	0,0066	0,9639	instrument
rs284660	19	41932084	G	T	0,0003	49,9278	0,0233	0,0033	1,6E-12	0,0061	0,0067	0,3563	instrument

rs28539246	17	29226630	A	T	0,0003	65,3180	-0,0268	0,0033	6,4E-16	-0,0348	0,0064	4,6E-08	outlier
rs2911450	16	88987497	A	C	0,0002	40,2967	-0,0216	0,0034	2,2E-10	0,0151	0,0071	0,0336	instrument
rs3020421	6	152348122	A	G	0,0003	47,8444	0,0240	0,0035	4,6E-12	0,0081	0,0066	0,2198	instrument
rs34134267	7	20417034	A	C	0,0005	86,0247	-0,0304	0,0033	1,8E-20	-0,0034	0,0062	0,5849	instrument
rs34517439	1	78450517	A	C	0,0002	30,6138	0,0276	0,0050	3,1E-08	0,0104	0,0105	0,3211	instrument
rs34831515	19	17275777	T	C	0,0002	38,8748	-0,0239	0,0038	4,5E-10	0,0007	0,0074	0,9248	instrument
rs34919557	20	32510282	T	C	0,0002	31,5616	-0,0315	0,0056	1,9E-08	0,0101	0,0106	0,3403	instrument
rs35307904	9	78511889	A	G	0,0007	137,8261	-0,0584	0,0050	8,0E-32	0,0115	0,0105	0,2767	instrument
rs35699973	18	20726731	G	A	0,0006	113,8477	-0,0419	0,0039	1,5E-26	0,0156	0,0077	0,0434	outlier
rs35874463	15	67457698	G	A	0,0005	91,8329	0,0663	0,0069	9,6E-22	0,0672	0,0142	2,3E-06	outlier
rs3738814	1	17331676	G	A	0,0005	102,4133	-0,0329	0,0033	4,4E-24	-0,0102	0,0062	0,1005	instrument
rs3791679	2	56096892	G	A	0,0012	235,3131	-0,0591	0,0038	4,4E-53	-0,0014	0,0073	0,8457	instrument
rs3794782	17	18127914	C	G	0,0002	35,3427	0,0198	0,0033	2,8E-09	-0,0100	0,0064	0,1171	instrument
rs3800162	6	2134391	A	T	0,0003	53,6990	-0,0241	0,0033	2,3E-13	-0,0142	0,0067	0,0330	instrument
rs395523	6	105396187	G	C	0,0006	123,6531	0,0384	0,0035	1,1E-28	-0,0024	0,0066	0,7221	instrument
rs39875	16	2268737	G	A	0,0002	32,2620	0,0214	0,0038	1,4E-08	-0,0138	0,0072	0,0575	instrument
rs4073717	5	170864021	T	G	0,0002	35,3308	-0,0240	0,0040	2,8E-09	0,0030	0,0080	0,7117	instrument
rs41284816	13	50655989	T	G	0,0003	52,9251	0,0890	0,0122	3,5E-13	0,0364	0,0204	0,0748	instrument
rs4321401	2	69348216	T	C	0,0003	49,0975	-0,0227	0,0032	2,4E-12	-0,0068	0,0068	0,3145	instrument
rs4383453	3	123068359	A	G	0,0002	30,7800	0,0229	0,0041	2,9E-08	-0,0056	0,0085	0,5095	instrument
rs4421120	5	32763118	A	G	0,0004	78,0034	0,0387	0,0044	1,0E-18	-0,0017	0,0090	0,8471	instrument
rs4432932	5	115039414	G	A	0,0002	39,5888	0,0230	0,0037	3,1E-10	-0,0046	0,0069	0,5020	instrument
rs4513485	3	49139966	G	A	0,0003	60,8238	-0,0266	0,0034	6,3E-15	0,0018	0,0066	0,7893	instrument
rs4626892	1	221234485	T	C	0,0002	44,7824	0,0218	0,0033	2,2E-11	0,0137	0,0065	0,0362	instrument
rs4699384	4	100570681	A	T	0,0002	32,4783	0,0234	0,0041	1,2E-08	0,0016	0,0080	0,8454	instrument
rs4731420	7	127863295	C	G	0,0002	30,8910	0,0234	0,0042	2,7E-08	-0,0064	0,0085	0,4490	instrument
rs475390	1	41570459	G	A	0,0002	32,3416	0,0221	0,0039	1,3E-08	-0,0111	0,0074	0,1347	instrument
rs4777230	15	70365762	C	T	0,0002	35,9636	0,0274	0,0046	2,0E-09	0,0122	0,0092	0,1831	instrument
rs4793987	17	46855837	C	T	0,0002	36,5175	-0,0202	0,0033	1,5E-09	-0,0013	0,0064	0,8385	instrument
rs481237	9	98316094	A	G	0,0003	59,7059	-0,0470	0,0061	1,1E-14	-0,0066	0,0131	0,6154	instrument
rs4842133	9	139127566	A	C	0,0005	93,9145	0,0348	0,0036	3,3E-22	0,0162	0,0072	0,0254	instrument

rs4848645	2	121623691	G	C	0,0002	30,7467	-0,0240	0,0043	3,0E-08	0,0000	0,0092	0,9963	instrument
rs4867721	5	172983457	G	T	0,0002	38,5637	0,0205	0,0033	5,3E-10	0,0072	0,0064	0,2560	instrument
rs4901541	14	55235282	C	T	0,0002	39,4757	-0,0208	0,0033	3,3E-10	-0,0048	0,0070	0,4947	instrument
rs4923575	11	28876429	C	T	0,0002	40,3729	-0,0207	0,0033	2,1E-10	0,0067	0,0063	0,2887	instrument
rs4936099	11	130280725	C	A	0,0002	42,7188	-0,0216	0,0033	6,3E-11	0,0041	0,0070	0,5621	instrument
rs4961091	8	89357874	T	A	0,0002	36,3243	0,0213	0,0035	1,7E-09	-0,0010	0,0067	0,8838	instrument
rs496298	10	131355130	A	G	0,0003	58,6137	-0,0261	0,0034	1,9E-14	-0,0026	0,0066	0,6917	instrument
rs537124	7	28203142	C	T	0,0006	114,4888	0,0378	0,0035	9,9E-27	-0,0089	0,0068	0,1928	instrument
rs550373	6	116754150	T	G	0,0002	41,3702	0,0212	0,0033	1,3E-10	-0,0066	0,0063	0,2926	instrument
rs56186137	16	28825953	G	A	0,0003	55,5914	0,0245	0,0033	8,9E-14	0,0042	0,0064	0,5078	instrument
rs57674902	20	4096741	T	C	0,0003	50,9791	0,0243	0,0034	9,3E-13	0,0076	0,0071	0,2846	instrument
rs584961	11	75277628	A	G	0,0003	55,6063	0,0379	0,0051	8,9E-14	0,0345	0,0108	0,0014	outlier
rs58697961	17	1665424	A	G	0,0002	37,5520	0,0209	0,0034	8,9E-10	-0,0032	0,0068	0,6345	instrument
rs60843830	2	286756	G	C	0,0002	33,7325	0,0198	0,0034	6,3E-09	0,0174	0,0066	0,0084	instrument
rs61732778	3	187443314	A	G	0,0002	32,6837	0,0358	0,0063	1,1E-08	-0,0182	0,0128	0,1551	instrument
rs61734651	20	61451332	T	C	0,0003	51,0362	0,0488	0,0068	9,1E-13	-0,0144	0,0147	0,3280	instrument
rs62055545	17	43964561	T	C	0,0004	78,6761	-0,0345	0,0039	7,4E-19	-0,0482	0,0076	2,5E-10	outlier
rs62076439	17	47404628	T	G	0,0004	73,1701	-0,0287	0,0034	1,2E-17	0,0108	0,0064	0,0918	instrument
rs62271373	3	150066540	A	T	0,0002	38,9247	-0,0441	0,0071	4,4E-10	-0,0119	0,0164	0,4672	instrument
rs62330027	5	318557	A	T	0,0002	29,9863	0,0298	0,0054	4,4E-08	0,0234	0,0109	0,0320	instrument
rs62515452	8	57187356	T	C	0,0002	44,5285	0,0305	0,0046	2,5E-11	0,0011	0,0098	0,9094	instrument
rs62621197	19	8670147	T	C	0,0011	211,1187	-0,1347	0,0093	8,2E-48	-0,0203	0,0218	0,3535	instrument
rs6439629	3	135901270	A	G	0,0002	45,9408	-0,0247	0,0036	1,2E-11	0,0051	0,0070	0,4665	instrument
rs6486059	11	12824581	A	G	0,0003	49,8290	0,0232	0,0033	1,7E-12	-0,0004	0,0065	0,9483	instrument
rs6492538	13	91993746	A	C	0,0002	46,7441	0,0268	0,0039	8,1E-12	0,0119	0,0074	0,1085	instrument
rs6557679	8	23383743	T	G	0,0002	35,3070	0,0221	0,0037	2,8E-09	0,0094	0,0070	0,1780	instrument
rs6585827	10	124165615	A	G	0,0002	38,1920	0,0199	0,0032	6,4E-10	0,0031	0,0064	0,6294	instrument
rs6601503	8	10540767	C	T	0,0002	38,8250	0,0230	0,0037	4,6E-10	0,0168	0,0078	0,0321	instrument
rs6739701	2	105132332	G	A	0,0004	66,6829	0,0267	0,0033	3,2E-16	-0,0057	0,0071	0,4201	instrument
rs6777217	3	36979042	A	G	0,0002	33,0047	0,0187	0,0032	9,2E-09	0,0078	0,0063	0,2168	instrument
rs6785012	3	141109348	T	C	0,0015	291,7233	0,0555	0,0032	2,4E-65	0,0473	0,0062	3,3E-14	outlier

rs68049170	10	72432047	A	G	0,0003	57,7746	0,0277	0,0036	2,9E-14	0,0005	0,0070	0,9440	instrument
rs6976501	7	50760220	G	A	0,0003	57,6227	0,0294	0,0039	3,2E-14	0,0000	0,0078	0,9986	instrument
rs6978655	7	46171683	T	C	0,0002	39,0246	0,0228	0,0036	4,2E-10	-0,0017	0,0073	0,8192	instrument
rs6990354	8	145282578	C	T	0,0002	40,3856	-0,0206	0,0032	2,1E-10	0,0087	0,0071	0,2194	instrument
rs702605	5	53167028	T	C	0,0002	33,7441	0,0188	0,0032	6,3E-09	-0,0171	0,0062	0,0057	outlier
rs7101582	11	47714610	A	G	0,0002	30,9133	0,0224	0,0040	2,7E-08	-0,0017	0,0078	0,8240	instrument
rs711245	2	36768875	A	G	0,0002	46,4574	-0,0235	0,0034	9,4E-12	-0,0028	0,0067	0,6794	instrument
rs7155279	14	92485881	T	G	0,0003	63,3291	-0,0267	0,0034	1,8E-15	0,0088	0,0064	0,1687	instrument
rs7170686	15	48821350	A	G	0,0003	56,0396	-0,0414	0,0055	7,1E-14	0,0249	0,0117	0,0338	instrument
rs7184427	16	88804734	A	G	0,0002	30,0301	-0,0248	0,0045	4,3E-08	-0,0185	0,0091	0,0423	instrument
rs7223156	17	68679731	T	C	0,0002	34,1637	-0,0206	0,0035	5,1E-09	-0,0038	0,0067	0,5761	instrument
rs72502575	6	31356087	T	C	0,0003	49,9561	-0,0319	0,0045	1,6E-12	-0,0064	0,0082	0,4317	instrument
rs72708236	1	155980320	G	T	0,0003	66,4707	-0,0773	0,0095	3,6E-16	-0,0297	0,0202	0,1424	instrument
rs72755233	15	100692953	A	G	0,0012	238,3911	-0,0784	0,0051	9,8E-54	0,0039	0,0127	0,7583	instrument
rs72940413	11	69202148	T	C	0,0002	32,3530	-0,0320	0,0056	1,3E-08	-0,0225	0,0104	0,0304	instrument
rs73111557	3	61523251	T	C	0,0002	36,7596	-0,0325	0,0054	1,3E-09	0,0128	0,0111	0,2507	instrument
rs73407643	6	35169123	C	T	0,0004	68,0618	-0,1022	0,0124	1,6E-16	0,0292	0,0242	0,2281	instrument
rs74469561	3	70929100	T	G	0,0002	39,7904	-0,0383	0,0061	2,8E-10	0,0071	0,0122	0,5616	instrument
rs74829909	4	12964932	C	T	0,0002	31,9448	-0,0305	0,0054	1,6E-08	-0,0009	0,0111	0,9342	instrument
rs75223147	6	19831020	G	T	0,0004	81,5942	0,1015	0,0112	1,7E-19	-0,0347	0,0349	0,3202	instrument
rs7534091	1	118864616	G	A	0,0003	54,6115	-0,0271	0,0037	1,5E-13	-0,0151	0,0073	0,0388	instrument
rs7536227	1	93262390	C	T	0,0002	31,2478	-0,0300	0,0054	2,3E-08	-0,0060	0,0103	0,5600	instrument
rs7540754	1	227850588	A	T	0,0004	76,0899	-0,0374	0,0043	2,7E-18	-0,0065	0,0085	0,4435	instrument
rs7674894	4	120680189	G	A	0,0002	37,3806	-0,0212	0,0035	9,7E-10	0,0055	0,0067	0,4101	instrument
rs7689420	4	145568352	T	C	0,0011	211,4094	-0,0625	0,0043	7,1E-48	0,0012	0,0084	0,8830	instrument
rs7718768	5	178536089	A	G	0,0004	79,9585	0,0305	0,0034	3,9E-19	0,0080	0,0068	0,2359	instrument
rs7736102	5	131604257	G	A	0,0003	66,0149	-0,0264	0,0032	4,5E-16	-0,0229	0,0062	0,0002	outlier
rs7757703	6	47578705	G	A	0,0002	44,3685	-0,0235	0,0035	2,7E-11	0,0010	0,0067	0,8823	instrument
rs7804293	7	92287849	G	T	0,0004	78,9069	0,0325	0,0037	6,6E-19	0,0255	0,0069	0,0002	outlier
rs781659	4	57779851	G	A	0,0002	33,5817	-0,0189	0,0033	6,8E-09	-0,0140	0,0063	0,0263	instrument
rs78198962	2	233094868	T	C	0,0003	51,3797	0,0655	0,0091	7,6E-13	0,0210	0,0239	0,3795	instrument

rs7846627	8	130736263	G	A	0,0003	58,7669	-0,0249	0,0032	1,8E-14	-0,0007	0,0062	0,9115	instrument
rs7874161	9	109639062	T	C	0,0002	31,3373	0,0189	0,0034	2,2E-08	0,0124	0,0067	0,0641	instrument
rs79112217	1	218622949	C	T	0,0003	61,3239	0,0412	0,0053	4,9E-15	0,0112	0,0105	0,2852	instrument
rs79312203	20	32082962	A	G	0,0002	37,6010	0,0329	0,0054	8,7E-10	-0,0193	0,0105	0,0657	instrument
rs7952436	11	67024534	T	C	0,0009	172,6578	-0,0764	0,0058	1,9E-39	-0,0119	0,0143	0,4048	instrument
rs798488	7	2802522	C	T	0,0010	187,9621	-0,0482	0,0035	9,7E-43	0,0138	0,0068	0,0406	outlier
rs7994151	13	115038879	G	A	0,0002	36,3846	-0,0271	0,0045	1,6E-09	0,0088	0,0087	0,3080	instrument
rs8003703	14	33411639	A	T	0,0002	31,3149	-0,0637	0,0114	2,2E-08	0,0063	0,0253	0,8024	instrument
rs80085026	7	148636421	G	C	0,0004	77,0525	0,0364	0,0041	1,7E-18	0,0306	0,0084	0,0003	outlier
rs8103728	19	33900257	C	G	0,0004	70,8620	-0,0289	0,0034	3,8E-17	0,0168	0,0067	0,0125	outlier
rs8105888	19	19704909	T	A	0,0003	64,1915	0,0324	0,0040	1,1E-15	0,0202	0,0078	0,0095	instrument
rs862041	14	74986403	T	C	0,0004	72,1473	-0,0286	0,0034	2,0E-17	-0,0010	0,0066	0,8769	instrument
rs9262136	6	30619988	A	C	0,0002	32,8211	-0,0264	0,0046	1,0E-08	-0,0093	0,0087	0,2824	instrument
rs927415	6	7698384	T	C	0,0002	33,7325	-0,0194	0,0033	6,3E-09	-0,0022	0,0064	0,7256	instrument
rs9285863	5	108071655	C	T	0,0003	51,5662	0,0246	0,0034	7,0E-13	0,0118	0,0065	0,0702	instrument
rs9321266	6	131316458	G	A	0,0003	56,3995	0,0249	0,0033	5,9E-14	-0,0086	0,0064	0,1782	instrument
rs9344005	6	81173371	G	C	0,0002	40,1698	0,0318	0,0050	2,3E-10	-0,0285	0,0100	0,0043	outlier
rs9344115	6	81834237	A	G	0,0002	32,3303	-0,0208	0,0037	1,3E-08	0,0039	0,0070	0,5808	instrument
rs9358912	6	26211146	T	G	0,0012	231,9505	-0,0553	0,0036	2,4E-52	0,0175	0,0068	0,0097	outlier
rs9442571	1	9349611	A	T	0,0003	66,2915	0,0396	0,0049	3,9E-16	-0,0052	0,0102	0,6119	instrument
rs979012	20	6623374	T	C	0,0004	78,9957	0,0297	0,0033	6,3E-19	0,0125	0,0066	0,0597	instrument
rs994014	4	82165790	C	T	0,0006	118,8088	0,0381	0,0035	1,2E-27	-0,0125	0,0068	0,0663	instrument

Table S12. Single Nucleotide Polymorphisms (SNPs) used as instruments in the Mendelian randomization analyses for the effect of TFR on endometrial cancer based on the genome-wide significance threshold P = 5E-08. R^2 represents the explained variance in the exposure. SNP-specific instrument strength is given by the respective F-statistic. Some of the genetic variants were identified as outliers in the iterative approach and therefore excluded from the analyses.

SNP	CHR	POS	EA	OA	R^2	F-stat	β_{TFR}	SE_{TFR}	P_{TFR}	$\beta_{Endometrial}$	$SE_{Endometrial}$	$P_{Endometrial}$	$variant_{Endometrial}$
rs1002436	1	89146852	A	G	0,0003	48,0799	-0,0225	0,0032	4,1E-12	0,0291	0,0160	0,0689	instrument
rs10059884	5	32832474	C	A	0,0002	45,7106	0,0222	0,0033	1,4E-11	0,0150	0,0166	0,3654	instrument
rs10152591	15	70048157	C	A	0,0003	52,0701	-0,0379	0,0053	5,4E-13	0,0153	0,0266	0,5642	instrument
rs10179126	2	165511794	G	C	0,0002	41,1261	0,0215	0,0034	1,4E-10	-0,0027	0,0163	0,8671	instrument

rs1020048	9	86666993	A	C	0,0003	54,2721	0,0308	0,0042	1,8E-13	-0,0083	0,0202	0,6811	instrument
rs1026029	2	177065248	C	T	0,0002	32,7295	-0,0409	0,0071	1,1E-08	-0,0475	0,0373	0,2023	instrument
rs10760110	9	123412563	C	T	0,0002	33,6745	0,0204	0,0035	6,5E-09	-0,0023	0,0174	0,8931	instrument
rs10761129	9	94486321	C	T	0,0002	41,9640	0,0222	0,0034	9,3E-11	0,0057	0,0176	0,7442	instrument
rs10823149	10	69935396	G	T	0,0002	38,5513	-0,0202	0,0032	5,3E-10	0,0026	0,0160	0,8708	instrument
rs10874671	1	103456389	G	C	0,0003	53,4209	0,0257	0,0035	2,7E-13	0,0084	0,0172	0,6259	instrument
rs10878987	12	69832392	T	C	0,0002	41,7441	0,0319	0,0049	1,0E-10	0,0022	0,0259	0,9327	instrument
rs10901171	9	133427194	C	T	0,0003	52,6200	-0,0242	0,0033	4,1E-13	-0,0015	0,0167	0,9280	instrument
rs10933385	2	232916068	A	G	0,0003	57,3043	-0,0910	0,0120	3,7E-14	-0,0108	0,0568	0,8493	instrument
rs11107120	12	93985482	C	T	0,0004	67,3870	0,0318	0,0039	2,3E-16	-0,0014	0,0192	0,9423	instrument
rs11130321	3	52762698	T	C	0,0003	60,2015	-0,0255	0,0033	8,6E-15	0,0284	0,0160	0,0765	instrument
rs11186507	10	92983831	G	A	0,0002	40,4873	0,0257	0,0040	2,0E-10	-0,0028	0,0200	0,8895	instrument
rs111947781	3	38021115	T	C	0,0002	42,4187	0,0290	0,0045	7,4E-11	-0,0175	0,0222	0,4309	instrument
rs11205303	1	149906413	C	T	0,0020	392,0359	0,0648	0,0033	3,6E-87	-0,0151	0,0163	0,3533	instrument
rs11259936	15	84580582	A	C	0,0017	324,7170	-0,0581	0,0032	1,6E-72	-0,0197	0,0158	0,2121	instrument
rs112635299	14	94838142	T	G	0,0005	93,4886	0,1081	0,0112	4,1E-22	-0,1230	0,0585	0,0355	instrument
rs113059026	10	32158478	A	C	0,0002	38,8499	-0,0223	0,0036	4,6E-10	0,0044	0,0173	0,8008	instrument
rs115912456	5	82815158	G	A	0,0005	86,3032	-0,0753	0,0081	1,6E-20	-0,0191	0,0421	0,6500	instrument
rs11689727	2	25458100	A	C	0,0006	106,5013	-0,0354	0,0034	6,0E-25	0,0171	0,0173	0,3241	instrument
rs11717301	3	72397284	A	C	0,0003	58,9818	-0,0289	0,0038	1,6E-14	-0,0026	0,0188	0,8898	instrument
rs12129970	1	54877581	G	A	0,0002	34,2690	-0,0307	0,0052	4,8E-09	0,0421	0,0275	0,1265	instrument
rs12172195	22	45813687	A	G	0,0003	50,8649	0,0324	0,0045	9,9E-13	-0,0285	0,0227	0,2099	instrument
rs12409899	1	172227011	G	C	0,0004	85,0260	0,0363	0,0039	3,0E-20	-0,0366	0,0193	0,0585	instrument
rs12507561	4	73464895	T	G	0,0007	127,2371	0,0365	0,0032	1,8E-29	-0,0124	0,0162	0,4430	instrument
rs12685003	9	17022667	G	A	0,0002	34,9514	-0,0304	0,0051	3,4E-09	-0,0159	0,0259	0,5405	instrument
rs12756815	1	113207243	A	C	0,0004	76,3518	-0,0349	0,0040	2,4E-18	0,0020	0,0194	0,9165	instrument
rs12811109	12	123471094	A	G	0,0002	37,7361	0,0248	0,0040	8,1E-10	-0,0177	0,0200	0,3755	instrument
rs13107395	4	8601846	C	T	0,0003	62,2988	-0,0258	0,0033	3,0E-15	0,0229	0,0169	0,1743	instrument
rs13128180	4	56286188	T	G	0,0003	51,6668	0,0253	0,0035	6,6E-13	0,0042	0,0171	0,8063	instrument
rs1313237	14	60848224	C	A	0,0002	46,4710	0,0226	0,0033	9,3E-12	-0,0060	0,0164	0,7134	instrument
rs13144767	4	17974219	G	A	0,0004	68,3922	-0,0395	0,0048	1,3E-16	0,0047	0,0235	0,8409	instrument

rs13170063	5	157895013	G	A	0,0002	45,5485	-0,0227	0,0034	1,5E-11	0,0069	0,0163	0,6731	instrument
rs13306455	19	7184721	T	C	0,0002	37,7115	0,0264	0,0043	8,2E-10	-0,0159	0,0210	0,4488	instrument
rs13429301	2	242061508	C	A	0,0002	32,8326	0,0330	0,0058	1,0E-08	0,0427	0,0284	0,1327	instrument
rs138809270	2	219929394	T	C	0,0003	52,6925	-0,0952	0,0131	3,9E-13	-0,1178	0,0653	0,0713	instrument
rs1388900	16	73800629	T	C	0,0002	33,4774	-0,0208	0,0036	7,2E-09	-0,0241	0,0178	0,1756	instrument
rs1421896	5	141016288	T	G	0,0002	30,5032	0,0183	0,0033	3,3E-08	0,0291	0,0164	0,0771	instrument
rs142599545	6	19844384	G	A	0,0002	37,4540	0,0812	0,0133	9,4E-10	0,1437	0,0811	0,0763	instrument
rs143384	20	34025756	G	A	0,0011	215,2067	0,0482	0,0033	1,1E-48	0,0593	0,0162	0,0003	outlier
rs143840904	11	2813322	T	C	0,0003	60,4811	-0,0978	0,0126	7,4E-15	0,0349	0,0765	0,6476	instrument
rs1452815	8	78123634	G	A	0,0002	35,6764	0,0213	0,0036	2,3E-09	-0,0005	0,0174	0,9786	instrument
rs1513304	3	99269750	C	T	0,0002	44,5418	0,0236	0,0035	2,5E-11	0,0180	0,0182	0,3232	instrument
rs1528589	5	88748768	C	T	0,0002	43,3617	0,0222	0,0034	4,6E-11	0,0159	0,0165	0,3355	instrument
rs1550436	15	74221157	T	C	0,0006	111,7237	-0,0343	0,0032	4,0E-26	0,0289	0,0158	0,0686	instrument
rs16942324	15	89383854	A	C	0,0005	104,6518	-0,1025	0,0100	1,4E-24	0,0455	0,0551	0,4088	instrument
rs16953461	17	46261736	G	T	0,0003	62,7574	-0,0312	0,0039	2,3E-15	0,0625	0,0190	0,0010	outlier
rs16996444	19	9997238	C	T	0,0002	47,0866	-0,0245	0,0036	6,8E-12	0,0099	0,0175	0,5714	instrument
rs17194431	8	120612627	T	C	0,0002	29,9206	0,0196	0,0036	4,5E-08	0,0127	0,0176	0,4719	instrument
rs1749824	10	80923862	A	C	0,0002	32,5011	-0,0185	0,0032	1,2E-08	-0,0245	0,0160	0,1257	instrument
rs17511102	2	37960613	T	A	0,0002	34,6446	0,0331	0,0056	4,0E-09	0,0231	0,0312	0,4598	instrument
rs17654949	2	219890955	T	C	0,0003	51,3940	0,0617	0,0086	7,6E-13	-0,0001	0,0437	0,9978	instrument
rs1805007	16	89986117	T	C	0,0003	56,8359	-0,0395	0,0052	4,7E-14	-0,0441	0,0290	0,1281	instrument
rs194059	5	56197416	G	A	0,0003	48,9575	-0,0238	0,0034	2,6E-12	-0,0485	0,0165	0,0033	outlier
rs1952256	1	184035116	G	A	0,0006	106,9145	0,0351	0,0034	4,6E-25	-0,0183	0,0166	0,2690	instrument
rs1980030	10	120970027	G	A	0,0003	47,7476	0,0227	0,0033	4,8E-12	0,0041	0,0164	0,8039	instrument
rs2006122	17	61987405	T	A	0,0003	47,1827	0,0252	0,0037	6,5E-12	0,0139	0,0178	0,4341	instrument
rs200889152	6	117507982	C	A	0,0002	42,3146	-0,0218	0,0034	7,8E-11	-0,0096	0,0167	0,5679	instrument
rs2035901	4	145521867	G	A	0,0002	30,3267	0,0179	0,0032	3,7E-08	0,0239	0,0162	0,1410	instrument
rs2050157	6	142658162	A	G	0,0008	146,8929	-0,0434	0,0036	8,4E-34	0,0107	0,0175	0,5419	instrument
rs2076112	22	38538822	T	C	0,0003	64,3999	-0,0261	0,0032	1,0E-15	-0,0236	0,0158	0,1351	instrument
rs2194411	3	185548663	A	G	0,0002	42,9020	0,0324	0,0049	5,8E-11	-0,0354	0,0249	0,1563	instrument
rs239458	8	37213496	G	A	0,0002	29,8441	-0,0315	0,0058	4,7E-08	0,0082	0,0287	0,7748	instrument

rs256904	5	55810305	A	T	0,0005	87,0667	0,0345	0,0037	1,1E-20	-0,0090	0,0187	0,6298	instrument
rs264762	5	64384386	A	C	0,0003	53,4648	-0,0250	0,0034	2,6E-13	0,0157	0,0169	0,3527	instrument
rs2712169	2	217671349	G	A	0,0002	44,7289	0,0221	0,0033	2,3E-11	0,0061	0,0162	0,7068	instrument
rs2722285	7	37994955	T	C	0,0002	32,0466	-0,0286	0,0051	1,5E-08	0,0082	0,0254	0,7483	instrument
rs2731340	3	13559829	C	T	0,0002	47,4440	0,0370	0,0054	5,7E-12	0,0083	0,0271	0,7590	instrument
rs273945	7	137611566	A	C	0,0002	38,3157	-0,0202	0,0033	6,0E-10	0,0110	0,0160	0,4910	instrument
rs2763265	6	168814600	G	A	0,0002	39,0371	-0,0239	0,0038	4,2E-10	-0,0075	0,0184	0,6851	instrument
rs2797963	6	34185749	C	T	0,0005	88,1148	0,0556	0,0059	6,2E-21	0,0526	0,0292	0,0721	instrument
rs2811462	3	129303436	G	T	0,0003	53,0561	0,0282	0,0039	3,2E-13	0,0104	0,0194	0,5909	instrument
rs2820443	1	219753509	C	T	0,0006	109,8293	0,0369	0,0035	1,0E-25	-0,0539	0,0176	0,0022	outlier
rs2823989	21	18080943	G	A	0,0002	32,8326	-0,0197	0,0034	1,0E-08	0,0163	0,0168	0,3299	instrument
rs284660	19	41932084	G	T	0,0003	49,9278	0,0233	0,0033	1,6E-12	0,0050	0,0165	0,7637	instrument
rs28539246	17	29226630	A	T	0,0003	65,3180	-0,0268	0,0033	6,4E-16	-0,0173	0,0162	0,2860	instrument
rs2911450	16	88987497	A	C	0,0002	40,2967	-0,0216	0,0034	2,2E-10	0,0044	0,0175	0,8030	instrument
rs3020421	6	152348122	A	G	0,0003	47,8444	0,0240	0,0035	4,6E-12	0,0699	0,0167	2,7E-05	outlier
rs34134267	7	20417034	A	C	0,0005	86,0247	-0,0304	0,0033	1,8E-20	-0,0179	0,0159	0,2607	instrument
rs34517439	1	78450517	A	C	0,0002	30,6138	0,0276	0,0050	3,1E-08	0,0424	0,0254	0,0947	instrument
rs34716573	12	576037	C	T	0,0003	48,8736	0,0238	0,0034	2,7E-12	-0,0099	0,0167	0,5533	instrument
rs34831515	19	17275777	T	C	0,0002	38,8748	-0,0239	0,0038	4,5E-10	0,0058	0,0187	0,7564	instrument
rs34919557	20	32510282	T	C	0,0002	31,5616	-0,0315	0,0056	1,9E-08	-0,0300	0,0276	0,2755	instrument
rs35307904	9	78511889	A	G	0,0007	137,8261	-0,0584	0,0050	8,0E-32	0,0094	0,0256	0,7133	instrument
rs35699973	18	20726731	G	A	0,0006	113,8477	-0,0419	0,0039	1,5E-26	0,0274	0,0197	0,1641	instrument
rs35874463	15	67457698	G	A	0,0005	91,8329	0,0663	0,0069	9,6E-22	0,0717	0,0367	0,0509	instrument
rs3738814	1	17331676	G	A	0,0005	102,4133	-0,0329	0,0033	4,4E-24	0,0016	0,0158	0,9207	instrument
rs3791679	2	56096892	G	A	0,0012	235,3131	-0,0591	0,0038	4,4E-53	-0,0347	0,0187	0,0631	instrument
rs3794782	17	18127914	C	G	0,0002	35,3427	0,0198	0,0033	2,8E-09	-0,0123	0,0163	0,4495	instrument
rs3800162	6	2134391	A	T	0,0003	53,6990	-0,0241	0,0033	2,3E-13	-0,0143	0,0165	0,3868	instrument
rs3898068	12	28610017	T	C	0,0005	92,2551	-0,0331	0,0035	7,7E-22	-0,0322	0,0170	0,0586	instrument
rs395523	6	105396187	G	C	0,0006	123,6531	0,0384	0,0035	1,1E-28	0,0210	0,0168	0,2127	instrument
rs39875	16	2268737	G	A	0,0002	32,2620	0,0214	0,0038	1,4E-08	-0,0277	0,0185	0,1351	instrument
rs4073717	5	170864021	T	G	0,0002	35,3308	-0,0240	0,0040	2,8E-09	-0,0330	0,0201	0,1008	instrument

rs41284816	13	50655989	T	G	0,0003	52,9251	0,0890	0,0122	3,5E-13	0,0784	0,0530	0,1389	instrument
rs4319547	12	123079035	A	G	0,0002	30,8133	-0,0203	0,0037	2,8E-08	-0,0269	0,0181	0,1376	instrument
rs4321401	2	69348216	T	C	0,0003	49,0975	-0,0227	0,0032	2,4E-12	-0,0226	0,0168	0,1785	instrument
rs4383453	3	123068359	A	G	0,0002	30,7800	0,0229	0,0041	2,9E-08	-0,0238	0,0209	0,2536	instrument
rs4421120	5	32763118	A	G	0,0004	78,0034	0,0387	0,0044	1,0E-18	-0,0467	0,0221	0,0350	instrument
rs4432932	5	115039414	G	A	0,0002	39,5888	0,0230	0,0037	3,1E-10	0,0269	0,0176	0,1257	instrument
rs4513485	3	49139966	G	A	0,0003	60,8238	-0,0266	0,0034	6,3E-15	0,0188	0,0167	0,2603	instrument
rs4699384	4	100570681	A	T	0,0002	32,4783	0,0234	0,0041	1,2E-08	0,0023	0,0204	0,9098	instrument
rs4731420	7	127863295	C	G	0,0002	30,8910	0,0234	0,0042	2,7E-08	0,0149	0,0211	0,4809	instrument
rs475390	1	41570459	G	A	0,0002	32,3416	0,0221	0,0039	1,3E-08	-0,0137	0,0190	0,4716	instrument
rs4777230	15	70365762	C	T	0,0002	35,9636	0,0274	0,0046	2,0E-09	-0,0278	0,0230	0,2270	instrument
rs4793987	17	46855837	C	T	0,0002	36,5175	-0,0202	0,0033	1,5E-09	-0,0072	0,0163	0,6594	instrument
rs481237	9	98316094	A	G	0,0003	59,7059	-0,0470	0,0061	1,1E-14	0,0753	0,0318	0,0180	instrument
rs4842133	9	139127566	A	C	0,0005	93,9145	0,0348	0,0036	3,3E-22	-0,0040	0,0183	0,8269	instrument
rs4848645	2	121623691	G	C	0,0002	30,7467	-0,0240	0,0043	3,0E-08	-0,0240	0,0222	0,2795	instrument
rs4867721	5	172983457	G	T	0,0002	38,5637	0,0205	0,0033	5,3E-10	0,0218	0,0162	0,1783	instrument
rs4901541	14	55235282	C	T	0,0002	39,4757	-0,0208	0,0033	3,3E-10	0,0056	0,0169	0,7412	instrument
rs4923575	11	28876429	C	T	0,0002	40,3729	-0,0207	0,0033	2,1E-10	-0,0017	0,0160	0,9133	instrument
rs4936099	11	130280725	C	A	0,0002	42,7188	-0,0216	0,0033	6,3E-11	-0,0062	0,0170	0,7159	instrument
rs4961091	8	89357874	T	A	0,0002	36,3243	0,0213	0,0035	1,7E-09	-0,0341	0,0172	0,0478	instrument
rs496298	10	131355130	A	G	0,0003	58,6137	-0,0261	0,0034	1,9E-14	0,0033	0,0167	0,8417	instrument
rs537124	7	28203142	C	T	0,0006	114,4888	0,0378	0,0035	9,9E-27	-0,0118	0,0173	0,4953	instrument
rs550373	6	116754150	T	G	0,0002	41,3702	0,0212	0,0033	1,3E-10	0,0055	0,0161	0,7322	instrument
rs56186137	16	28825953	G	A	0,0003	55,5914	0,0245	0,0033	8,9E-14	0,0542	0,0161	0,0008	outlier
rs57674902	20	4096741	T	C	0,0003	50,9791	0,0243	0,0034	9,3E-13	0,0242	0,0172	0,1598	instrument
rs584961	11	75277628	A	G	0,0003	55,6063	0,0379	0,0051	8,9E-14	0,0012	0,0262	0,9628	instrument
rs58697961	17	1665424	A	G	0,0002	37,5520	0,0209	0,0034	8,9E-10	-0,0203	0,0173	0,2411	instrument
rs60843830	2	286756	G	C	0,0002	33,7325	0,0198	0,0034	6,3E-09	0,0412	0,0168	0,0139	instrument
rs61732778	3	187443314	A	G	0,0002	32,6837	0,0358	0,0063	1,1E-08	0,0411	0,0318	0,1956	instrument
rs61734651	20	61451332	T	C	0,0003	51,0362	0,0488	0,0068	9,1E-13	0,0270	0,0370	0,4652	instrument
rs62055545	17	43964561	T	C	0,0004	78,6761	-0,0345	0,0039	7,4E-19	-0,0133	0,0193	0,4905	instrument

rs62076439	17	47404628	T	G	0,0004	73,1701	-0,0287	0,0034	1,2E-17	0,0123	0,0164	0,4540	instrument
rs62271373	3	150066540	A	T	0,0002	38,9247	-0,0441	0,0071	4,4E-10	-0,0181	0,0386	0,6400	instrument
rs62330027	5	318557	A	T	0,0002	29,9863	0,0298	0,0054	4,4E-08	-0,0067	0,0271	0,8040	instrument
rs62515452	8	57187356	T	C	0,0002	44,5285	0,0305	0,0046	2,5E-11	0,0159	0,0240	0,5055	instrument
rs62621197	19	8670147	T	C	0,0011	211,1187	-0,1347	0,0093	8,2E-48	-0,0510	0,0583	0,3809	instrument
rs6439629	3	135901270	A	G	0,0002	45,9408	-0,0247	0,0036	1,2E-11	0,0095	0,0178	0,5927	instrument
rs6486059	11	12824581	A	G	0,0003	49,8290	0,0232	0,0033	1,7E-12	0,0098	0,0163	0,5473	instrument
rs6492538	13	91993746	A	C	0,0002	46,7441	0,0268	0,0039	8,1E-12	0,0108	0,0188	0,5657	instrument
rs6557679	8	23383743	T	G	0,0002	35,3070	0,0221	0,0037	2,8E-09	-0,0363	0,0179	0,0426	instrument
rs6581626	12	65711378	A	G	0,0002	35,3308	0,0192	0,0032	2,8E-09	-0,0046	0,0158	0,7701	instrument
rs6585827	10	124165615	A	G	0,0002	38,1920	0,0199	0,0032	6,4E-10	0,0013	0,0161	0,9358	instrument
rs6601503	8	10540767	C	T	0,0002	38,8250	0,0230	0,0037	4,6E-10	0,0112	0,0194	0,5625	instrument
rs6739701	2	105132332	G	A	0,0004	66,6829	0,0267	0,0033	3,2E-16	0,0166	0,0170	0,3298	instrument
rs6777217	3	36979042	A	G	0,0002	33,0047	0,0187	0,0032	9,2E-09	-0,0075	0,0159	0,6359	instrument
rs6785012	3	141109348	T	C	0,0015	291,7233	0,0555	0,0032	2,4E-65	0,0343	0,0159	0,0307	instrument
rs68049170	10	72432047	A	G	0,0003	57,7746	0,0277	0,0036	2,9E-14	0,0047	0,0178	0,7933	instrument
rs6976501	7	50760220	G	A	0,0003	57,6227	0,0294	0,0039	3,2E-14	0,0114	0,0194	0,5560	instrument
rs6978655	7	46171683	T	C	0,0002	39,0246	0,0228	0,0036	4,2E-10	0,0171	0,0181	0,3448	instrument
rs701006	12	58106836	A	G	0,0002	35,6525	-0,0196	0,0033	2,4E-09	0,0012	0,0160	0,9422	instrument
rs702605	5	53167028	T	C	0,0002	33,7441	0,0188	0,0032	6,3E-09	-0,0088	0,0157	0,5754	instrument
rs7101582	11	47714610	A	G	0,0002	30,9133	0,0224	0,0040	2,7E-08	0,0001	0,0200	0,9951	instrument
rs711245	2	36768875	A	G	0,0002	46,4574	-0,0235	0,0034	9,4E-12	0,0134	0,0169	0,4269	instrument
rs7137574	12	77667041	G	A	0,0002	31,8318	-0,0204	0,0036	1,7E-08	0,0081	0,0178	0,6475	instrument
rs7155279	14	92485881	T	G	0,0003	63,3291	-0,0267	0,0034	1,8E-15	0,0016	0,0165	0,9219	instrument
rs7170686	15	48821350	A	G	0,0003	56,0396	-0,0414	0,0055	7,1E-14	-0,0107	0,0283	0,7050	instrument
rs7184427	16	88804734	A	G	0,0002	30,0301	-0,0248	0,0045	4,3E-08	-0,0158	0,0229	0,4909	instrument
rs7223156	17	68679731	T	C	0,0002	34,1637	-0,0206	0,0035	5,1E-09	0,0111	0,0172	0,5169	instrument
rs72502575	6	31356087	T	C	0,0003	49,9561	-0,0319	0,0045	1,6E-12	0,0409	0,0215	0,0565	instrument
rs72708236	1	155980320	G	T	0,0003	66,4707	-0,0773	0,0095	3,6E-16	-0,0252	0,0505	0,6176	instrument
rs72755233	15	100692953	A	G	0,0012	238,3911	-0,0784	0,0051	9,8E-54	-0,0123	0,0309	0,6900	instrument
rs72940413	11	69202148	T	C	0,0002	32,3530	-0,0320	0,0056	1,3E-08	-0,0008	0,0266	0,9774	instrument

rs73111557	3	61523251	T	C	0,0002	36,7596	-0,0325	0,0054	1,3E-09	0,0057	0,0270	0,8321	instrument
rs74469561	3	70929100	T	G	0,0002	39,7904	-0,0383	0,0061	2,8E-10	0,0190	0,0301	0,5281	instrument
rs74829909	4	12964932	C	T	0,0002	31,9448	-0,0305	0,0054	1,6E-08	-0,0170	0,0270	0,5285	instrument
rs75223147	6	19831020	G	T	0,0004	81,5942	0,1015	0,0112	1,7E-19	0,0378	0,0821	0,6450	instrument
rs7534091	1	118864616	G	A	0,0003	54,6115	-0,0271	0,0037	1,5E-13	0,0066	0,0182	0,7179	instrument
rs753480	12	102958125	C	T	0,0002	33,0392	-0,0984	0,0171	9,0E-09	-0,0444	0,0752	0,5544	instrument
rs7536227	1	93262390	C	T	0,0002	31,2478	-0,0300	0,0054	2,3E-08	-0,0146	0,0261	0,5751	instrument
rs7540754	1	227850588	A	T	0,0004	76,0899	-0,0374	0,0043	2,7E-18	-0,0308	0,0214	0,1507	instrument
rs7674894	4	120680189	G	A	0,0002	37,3806	-0,0212	0,0035	9,7E-10	-0,0110	0,0171	0,5181	instrument
rs7689420	4	145568352	T	C	0,0011	211,4094	-0,0625	0,0043	7,1E-48	0,0183	0,0214	0,3913	instrument
rs76929617	12	120867798	G	A	0,0002	44,6219	-0,0554	0,0083	2,4E-11	-0,0141	0,0450	0,7546	instrument
rs7718768	5	178536089	A	G	0,0004	79,9585	0,0305	0,0034	3,9E-19	-0,0255	0,0170	0,1347	instrument
rs7736102	5	131604257	G	A	0,0003	66,0149	-0,0264	0,0032	4,5E-16	-0,0029	0,0158	0,8535	instrument
rs7757703	6	47578705	G	A	0,0002	44,3685	-0,0235	0,0035	2,7E-11	0,0152	0,0171	0,3740	instrument
rs7804293	7	92287849	G	T	0,0004	78,9069	0,0325	0,0037	6,6E-19	-0,0079	0,0178	0,6595	instrument
rs781659	4	57779851	G	A	0,0002	33,5817	-0,0189	0,0033	6,8E-09	-0,0444	0,0160	0,0056	outlier
rs78198962	2	233094868	T	C	0,0003	51,3797	0,0655	0,0091	7,6E-13	0,0972	0,0563	0,0841	instrument
rs7846627	8	130736263	G	A	0,0003	58,7669	-0,0249	0,0032	1,8E-14	0,0063	0,0158	0,6897	instrument
rs7874161	9	109639062	T	C	0,0002	31,3373	0,0189	0,0034	2,2E-08	-0,0055	0,0169	0,7428	instrument
rs78759565	12	94404805	G	A	0,0002	30,6359	-0,0383	0,0069	3,1E-08	-0,0198	0,0365	0,5871	instrument
rs79112217	1	218622949	C	T	0,0003	61,3239	0,0412	0,0053	4,9E-15	0,0063	0,0265	0,8122	instrument
rs79312203	20	32082962	A	G	0,0002	37,6010	0,0329	0,0054	8,7E-10	0,0097	0,0271	0,7204	instrument
rs7952436	11	67024534	T	C	0,0009	172,6578	-0,0764	0,0058	1,9E-39	-0,0637	0,0349	0,0677	instrument
rs7959830	12	66347368	T	G	0,0005	95,6865	-0,0321	0,0033	1,4E-22	-0,0053	0,0162	0,7440	instrument
rs7968440	12	51134741	G	A	0,0004	79,3516	0,0301	0,0034	5,2E-19	-0,0159	0,0167	0,3412	instrument
rs7970001	12	32452376	C	G	0,0002	31,4942	0,0306	0,0055	2,0E-08	-0,0155	0,0275	0,5736	instrument
rs798488	7	2802522	C	T	0,0010	187,9621	-0,0482	0,0035	9,7E-43	-0,0019	0,0173	0,9128	instrument
rs7994151	13	115038879	G	A	0,0002	36,3846	-0,0271	0,0045	1,6E-09	-0,0140	0,0223	0,5302	instrument
rs8003703	14	33411639	A	T	0,0002	31,3149	-0,0637	0,0114	2,2E-08	0,0672	0,0609	0,2693	instrument
rs80085026	7	148636421	G	C	0,0004	77,0525	0,0364	0,0041	1,7E-18	0,0162	0,0208	0,4345	instrument
rs8103728	19	33900257	C	G	0,0004	70,8620	-0,0289	0,0034	3,8E-17	0,0222	0,0170	0,1911	instrument

rs8105888	19	19704909	T	A	0,0003	64,1915	0,0324	0,0040	1,1E-15	0,0033	0,0202	0,8688	instrument
rs862041	14	74986403	T	C	0,0004	72,1473	-0,0286	0,0034	2,0E-17	0,0084	0,0166	0,6119	instrument
rs9262136	6	30619988	A	C	0,0002	32,8211	-0,0264	0,0046	1,0E-08	0,0123	0,0228	0,5888	instrument
rs927415	6	7698384	T	C	0,0002	33,7325	-0,0194	0,0033	6,3E-09	-0,0141	0,0163	0,3859	instrument
rs9285863	5	108071655	C	T	0,0003	51,5662	0,0246	0,0034	7,0E-13	-0,0321	0,0168	0,0555	instrument
rs9321266	6	131316458	G	A	0,0003	56,3995	0,0249	0,0033	5,9E-14	-0,0156	0,0163	0,3374	instrument
rs9344005	6	81173371	G	C	0,0002	40,1698	0,0318	0,0050	2,3E-10	0,0130	0,0249	0,6020	instrument
rs9344115	6	81834237	A	G	0,0002	32,3303	-0,0208	0,0037	1,3E-08	0,0376	0,0179	0,0354	instrument
rs9358912	6	26211146	T	G	0,0012	231,9505	-0,0553	0,0036	2,4E-52	0,0180	0,0174	0,2991	instrument
rs9442571	1	9349611	A	T	0,0003	66,2915	0,0396	0,0049	3,9E-16	-0,0128	0,0251	0,6097	instrument
rs979012	20	6623374	T	C	0,0004	78,9957	0,0297	0,0033	6,3E-19	-0,0358	0,0167	0,0315	instrument
rs994014	4	82165790	C	T	0,0006	118,8088	0,0381	0,0035	1,2E-27	-0,0178	0,0172	0,2989	instrument

Table S13. Single Nucleotide Polymorphisms (SNPs) used as instruments in the Mendelian randomization analyses for the effect of TFR on ovarian cancer based on the genome-wide significance threshold P = 5E-08. R^2 represents the explained variance in the exposure. SNP-specific instrument strength is given by the respective F-statistic. Some of the genetic variants were identified as outliers in the iterative approach and therefore excluded from the analyses.

SNP	CHR	POS	EA	OA	R^2	F-stat	β_{TFR}	SE_{TFR}	P_{TFR}	$\beta_{Ovarian}$	$SE_{Ovarian}$	$P_{Ovarian}$	$variant_{Ovarian}$
rs1002436	1	89146852	A	G	0,0003	48,0799	-0,0225	0,0032	4,1E-12	-0,0093	0,0134	0,4867	instrument
rs10059884	5	32832474	C	A	0,0002	45,7106	0,0222	0,0033	1,4E-11	-0,0102	0,0138	0,4614	instrument
rs10152591	15	70048157	C	A	0,0003	52,0701	-0,0379	0,0053	5,4E-13	-0,0083	0,0228	0,7142	instrument
rs10179126	2	165511794	G	C	0,0002	41,1261	0,0215	0,0034	1,4E-10	-0,0145	0,0137	0,2882	instrument
rs1020048	9	86666993	A	C	0,0003	54,2721	0,0308	0,0042	1,8E-13	-0,0139	0,0168	0,4069	instrument
rs1026029	2	177065248	C	T	0,0002	32,7295	-0,0409	0,0071	1,1E-08	-0,0730	0,0317	0,0213	instrument
rs10760110	9	123412563	C	T	0,0002	33,6745	0,0204	0,0035	6,5E-09	0,0159	0,0146	0,2777	instrument
rs10761129	9	94486321	C	T	0,0002	41,9640	0,0222	0,0034	9,3E-11	0,0314	0,0145	0,0309	instrument
rs10823149	10	69935396	G	T	0,0002	38,5513	-0,0202	0,0032	5,3E-10	-0,0083	0,0133	0,5317	instrument
rs10874671	1	103456389	G	C	0,0003	53,4209	0,0257	0,0035	2,7E-13	0,0026	0,0143	0,8572	instrument
rs10901171	9	133427194	C	T	0,0003	52,6200	-0,0242	0,0033	4,1E-13	0,0161	0,0140	0,2503	instrument
rs10933385	2	232916068	A	G	0,0003	57,3043	-0,0910	0,0120	3,7E-14	-0,0454	0,0478	0,3421	instrument
rs11031005	11	30226356	C	T	0,0002	30,8244	0,0256	0,0046	2,8E-08	-0,0345	0,0191	0,0706	instrument
rs11130321	3	52762698	T	C	0,0003	60,2015	-0,0255	0,0033	8,6E-15	0,0090	0,0134	0,5034	instrument

rs11186507	10	92983831	G	A	0,0002	40,4873	0,0257	0,0040	2,0E-10	0,0027	0,0170	0,8754	instrument
rs111947781	3	38021115	T	C	0,0002	42,4187	0,0290	0,0045	7,4E-11	0,0123	0,0185	0,5045	instrument
rs11205303	1	149906413	C	T	0,0020	392,0359	0,0648	0,0033	3,6E-87	-0,0053	0,0135	0,6933	instrument
rs11259936	15	84580582	A	C	0,0017	324,7170	-0,0581	0,0032	1,6E-72	-0,0121	0,0132	0,3576	instrument
rs112635299	14	94838142	T	G	0,0005	93,4886	0,1081	0,0112	4,1E-22	0,0039	0,0498	0,9382	instrument
rs113059026	10	32158478	A	C	0,0002	38,8499	-0,0223	0,0036	4,6E-10	-0,0076	0,0146	0,5999	instrument
rs115912456	5	82815158	G	A	0,0005	86,3032	-0,0753	0,0081	1,6E-20	-2,4E-05	0,0342	0,9994	instrument
rs11689727	2	25458100	A	C	0,0006	106,5013	-0,0354	0,0034	6,0E-25	-0,0266	0,0146	0,0679	instrument
rs11717301	3	72397284	A	C	0,0003	58,9818	-0,0289	0,0038	1,6E-14	0,0107	0,0156	0,4938	instrument
rs12129970	1	54877581	G	A	0,0002	34,2690	-0,0307	0,0052	4,8E-09	-0,0279	0,0234	0,2340	instrument
rs12172195	22	45813687	A	G	0,0003	50,8649	0,0324	0,0045	9,9E-13	-0,0011	0,0187	0,9511	instrument
rs12409899	1	172227011	G	C	0,0004	85,0260	0,0363	0,0039	3,0E-20	0,0146	0,0161	0,3658	instrument
rs12507561	4	73464895	T	G	0,0007	127,2371	0,0365	0,0032	1,8E-29	0,0014	0,0136	0,9198	instrument
rs12685003	9	17022667	G	A	0,0002	34,9514	-0,0304	0,0051	3,4E-09	-0,1022	0,0229	7,4E-06	outlier
rs12756815	1	113207243	A	C	0,0004	76,3518	-0,0349	0,0040	2,4E-18	-0,0248	0,0163	0,1286	instrument
rs13107395	4	8601846	C	T	0,0003	62,2988	-0,0258	0,0033	3,0E-15	0,0049	0,0139	0,7233	instrument
rs13128180	4	56286188	T	G	0,0003	51,6668	0,0253	0,0035	6,6E-13	0,0302	0,0142	0,0337	instrument
rs1313237	14	60848224	C	A	0,0002	46,4710	0,0226	0,0033	9,3E-12	-0,0146	0,0136	0,2834	instrument
rs13144767	4	17974219	G	A	0,0004	68,3922	-0,0395	0,0048	1,3E-16	0,0223	0,0194	0,2510	instrument
rs13170063	5	157895013	G	A	0,0002	45,5485	-0,0227	0,0034	1,5E-11	-0,0239	0,0136	0,0782	instrument
rs13306455	19	7184721	T	C	0,0002	37,7115	0,0264	0,0043	8,2E-10	-0,0233	0,0176	0,1867	instrument
rs13429301	2	242061508	C	A	0,0002	32,8326	0,0330	0,0058	1,0E-08	-0,0049	0,0238	0,8371	instrument
rs138809270	2	219929394	T	C	0,0003	52,6925	-0,0952	0,0131	3,9E-13	-0,0596	0,0522	0,2534	instrument
rs1388900	16	73800629	T	C	0,0002	33,4774	-0,0208	0,0036	7,2E-09	0,0013	0,0149	0,9333	instrument
rs1421896	5	141016288	T	G	0,0002	30,5032	0,0183	0,0033	3,3E-08	0,0165	0,0138	0,2319	instrument
rs142599545	6	19844384	G	A	0,0002	37,4540	0,0812	0,0133	9,4E-10	0,0550	0,0684	0,4214	instrument
rs143384	20	34025756	G	A	0,0011	215,2067	0,0482	0,0033	1,1E-48	0,0061	0,0134	0,6497	instrument
rs143840904	11	2813322	T	C	0,0003	60,4811	-0,0978	0,0126	7,4E-15	-0,0747	0,0653	0,2527	instrument
rs1452815	8	78123634	G	A	0,0002	35,6764	0,0213	0,0036	2,3E-09	-0,0168	0,0147	0,2523	instrument
rs1513304	3	99269750	C	T	0,0002	44,5418	0,0236	0,0035	2,5E-11	-0,0004	0,0154	0,9786	instrument
rs1528589	5	88748768	C	T	0,0002	43,3617	0,0222	0,0034	4,6E-11	-0,0113	0,0138	0,4136	instrument

rs1550436	15	74221157	T	C	0,0006	111,7237	-0,0343	0,0032	4,0E-26	0,0111	0,0132	0,4019	instrument
rs16942324	15	89383854	A	C	0,0005	104,6518	-0,1025	0,0100	1,4E-24	-0,0291	0,0446	0,5138	instrument
rs16953461	17	46261736	G	T	0,0003	62,7574	-0,0312	0,0039	2,3E-15	-0,0319	0,0167	0,0552	instrument
rs16996444	19	9997238	C	T	0,0002	47,0866	-0,0245	0,0036	6,8E-12	0,0260	0,0149	0,0810	instrument
rs17194431	8	120612627	T	C	0,0002	29,9206	0,0196	0,0036	4,5E-08	0,0231	0,0148	0,1170	instrument
rs1749824	10	80923862	A	C	0,0002	32,5011	-0,0185	0,0032	1,2E-08	0,0089	0,0133	0,5039	instrument
rs17511102	2	37960613	T	A	0,0002	34,6446	0,0331	0,0056	4,0E-09	-0,0453	0,0330	0,1695	instrument
rs17654949	2	219890955	T	C	0,0003	51,3940	0,0617	0,0086	7,6E-13	-0,0622	0,0367	0,0901	instrument
rs1805007	16	89986117	T	C	0,0003	56,8359	-0,0395	0,0052	4,7E-14	0,0257	0,0249	0,3012	instrument
rs194059	5	56197416	G	A	0,0003	48,9575	-0,0238	0,0034	2,6E-12	-0,0263	0,0138	0,0572	instrument
rs1952256	1	184035116	G	A	0,0006	106,9145	0,0351	0,0034	4,6E-25	0,0027	0,0139	0,8487	instrument
rs1966265	5	176516631	A	G	0,0003	51,8251	0,0268	0,0037	6,1E-13	0,0107	0,0155	0,4895	instrument
rs1980030	10	120970027	G	A	0,0003	47,7476	0,0227	0,0033	4,8E-12	0,0010	0,0138	0,9431	instrument
rs2006122	17	61987405	T	A	0,0003	47,1827	0,0252	0,0037	6,5E-12	0,0269	0,0150	0,0715	instrument
rs200889152	6	117507982	C	A	0,0002	42,3146	-0,0218	0,0034	7,8E-11	0,0141	0,0138	0,3061	instrument
rs2035901	4	145521867	G	A	0,0002	30,3267	0,0179	0,0032	3,7E-08	0,0083	0,0136	0,5395	instrument
rs2050157	6	142658162	A	G	0,0008	146,8929	-0,0434	0,0036	8,4E-34	0,0175	0,0146	0,2330	instrument
rs2076112	22	38538822	T	C	0,0003	64,3999	-0,0261	0,0032	1,0E-15	0,0112	0,0132	0,3957	instrument
rs2194411	3	185548663	A	G	0,0002	42,9020	0,0324	0,0049	5,8E-11	-0,0006	0,0208	0,9764	instrument
rs239458	8	37213496	G	A	0,0002	29,8441	-0,0315	0,0058	4,7E-08	-0,0035	0,0236	0,8817	instrument
rs256904	5	55810305	A	T	0,0005	87,0667	0,0345	0,0037	1,1E-20	0,0104	0,0154	0,4984	instrument
rs264762	5	64384386	A	C	0,0003	53,4648	-0,0250	0,0034	2,6E-13	-0,0157	0,0140	0,2623	instrument
rs2712169	2	217671349	G	A	0,0002	44,7289	0,0221	0,0033	2,3E-11	0,0183	0,0134	0,1745	instrument
rs2722285	7	37994955	T	C	0,0002	32,0466	-0,0286	0,0051	1,5E-08	-0,0299	0,0217	0,1683	instrument
rs2731340	3	13559829	C	T	0,0002	47,4440	0,0370	0,0054	5,7E-12	-0,0085	0,0222	0,7007	instrument
rs273945	7	137611566	A	C	0,0002	38,3157	-0,0202	0,0033	6,0E-10	0,0031	0,0133	0,8150	instrument
rs2763265	6	168814600	G	A	0,0002	39,0371	-0,0239	0,0038	4,2E-10	0,0012	0,0154	0,9405	instrument
rs2797963	6	34185749	C	T	0,0005	88,1148	0,0556	0,0059	6,2E-21	0,0419	0,0247	0,0890	instrument
rs2811462	3	129303436	G	T	0,0003	53,0561	0,0282	0,0039	3,2E-13	0,0241	0,0161	0,1343	instrument
rs2820443	1	219753509	C	T	0,0006	109,8293	0,0369	0,0035	1,0E-25	-0,0007	0,0147	0,9642	instrument
rs2823989	21	18080943	G	A	0,0002	32,8326	-0,0197	0,0034	1,0E-08	-0,0121	0,0141	0,3887	instrument

rs284660	19	41932084	G	T	0,0003	49,9278	0,0233	0,0033	1,6E-12	0,0033	0,0137	0,8100	instrument
rs28539246	17	29226630	A	T	0,0003	65,3180	-0,0268	0,0033	6,4E-16	-0,0413	0,0135	0,0022	outlier
rs2911450	16	88987497	A	C	0,0002	40,2967	-0,0216	0,0034	2,2E-10	0,0280	0,0148	0,0575	instrument
rs3020421	6	152348122	A	G	0,0003	47,8444	0,0240	0,0035	4,6E-12	0,0142	0,0141	0,3127	instrument
rs34134267	7	20417034	A	C	0,0005	86,0247	-0,0304	0,0033	1,8E-20	-0,0117	0,0133	0,3777	instrument
rs34517439	1	78450517	A	C	0,0002	30,6138	0,0276	0,0050	3,1E-08	-0,0016	0,0216	0,9426	instrument
rs34831515	19	17275777	T	C	0,0002	38,8748	-0,0239	0,0038	4,5E-10	0,0180	0,0159	0,2574	instrument
rs34919557	20	32510282	T	C	0,0002	31,5616	-0,0315	0,0056	1,9E-08	0,0219	0,0238	0,3579	instrument
rs35307904	9	78511889	A	G	0,0007	137,8261	-0,0584	0,0050	8,0E-32	-0,0092	0,0218	0,6741	instrument
rs35699973	18	20726731	G	A	0,0006	113,8477	-0,0419	0,0039	1,5E-26	-0,0274	0,0164	0,0948	instrument
rs35874463	15	67457698	G	A	0,0005	91,8329	0,0663	0,0069	9,6E-22	-0,0411	0,0301	0,1720	instrument
rs3738814	1	17331676	G	A	0,0005	102,4133	-0,0329	0,0033	4,4E-24	-0,0361	0,0133	0,0066	instrument
rs3791679	2	56096892	G	A	0,0012	235,3131	-0,0591	0,0038	4,4E-53	0,0115	0,0154	0,4535	instrument
rs3794782	17	18127914	C	G	0,0002	35,3427	0,0198	0,0033	2,8E-09	-0,0156	0,0136	0,2500	instrument
rs3800162	6	2134391	A	T	0,0003	53,6990	-0,0241	0,0033	2,3E-13	0,0019	0,0137	0,8924	instrument
rs395523	6	105396187	G	C	0,0006	123,6531	0,0384	0,0035	1,1E-28	0,0152	0,0142	0,2832	instrument
rs39875	16	2268737	G	A	0,0002	32,2620	0,0214	0,0038	1,4E-08	-0,0010	0,0152	0,9469	instrument
rs4073717	5	170864021	T	G	0,0002	35,3308	-0,0240	0,0040	2,8E-09	0,0080	0,0167	0,6313	instrument
rs41284816	13	50655989	T	G	0,0003	52,9251	0,0890	0,0122	3,5E-13	-0,0167	0,0453	0,7121	instrument
rs4321401	2	69348216	T	C	0,0003	49,0975	-0,0227	0,0032	2,4E-12	0,0015	0,0143	0,9167	instrument
rs4383453	3	123068359	A	G	0,0002	30,7800	0,0229	0,0041	2,9E-08	-0,0043	0,0173	0,8043	instrument
rs4421120	5	32763118	A	G	0,0004	78,0034	0,0387	0,0044	1,0E-18	-0,0054	0,0185	0,7718	instrument
rs4432932	5	115039414	G	A	0,0002	39,5888	0,0230	0,0037	3,1E-10	-0,0043	0,0146	0,7674	instrument
rs4513485	3	49139966	G	A	0,0003	60,8238	-0,0266	0,0034	6,3E-15	-0,0151	0,0140	0,2799	instrument
rs4626892	1	221234485	T	C	0,0002	44,7824	0,0218	0,0033	2,2E-11	0,0051	0,0135	0,7059	instrument
rs4699384	4	100570681	A	T	0,0002	32,4783	0,0234	0,0041	1,2E-08	-0,0105	0,0170	0,5358	instrument
rs4731420	7	127863295	C	G	0,0002	30,8910	0,0234	0,0042	2,7E-08	0,0048	0,0174	0,7824	instrument
rs475390	1	41570459	G	A	0,0002	32,3416	0,0221	0,0039	1,3E-08	0,0009	0,0159	0,9552	instrument
rs4777230	15	70365762	C	T	0,0002	35,9636	0,0274	0,0046	2,0E-09	0,0099	0,0198	0,6170	instrument
rs4793987	17	46855837	C	T	0,0002	36,5175	-0,0202	0,0033	1,5E-09	-0,0106	0,0135	0,4329	instrument
rs481237	9	98316094	A	G	0,0003	59,7059	-0,0470	0,0061	1,1E-14	0,0107	0,0263	0,6847	instrument

rs4842133	9	139127566	A	C	0,0005	93,9145	0,0348	0,0036	3,3E-22	-0,0110	0,0152	0,4679	instrument
rs4848645	2	121623691	G	C	0,0002	30,7467	-0,0240	0,0043	3,0E-08	-0,0034	0,0194	0,8622	instrument
rs4867721	5	172983457	G	T	0,0002	38,5637	0,0205	0,0033	5,3E-10	-0,0264	0,0135	0,0507	instrument
rs4901541	14	55235282	C	T	0,0002	39,4757	-0,0208	0,0033	3,3E-10	0,0037	0,0142	0,7938	instrument
rs4923575	11	28876429	C	T	0,0002	40,3729	-0,0207	0,0033	2,1E-10	-0,0199	0,0134	0,1365	instrument
rs4936099	11	130280725	C	A	0,0002	42,7188	-0,0216	0,0033	6,3E-11	-0,0023	0,0142	0,8733	instrument
rs4961091	8	89357874	T	A	0,0002	36,3243	0,0213	0,0035	1,7E-09	0,0058	0,0143	0,6843	instrument
rs496298	10	131355130	A	G	0,0003	58,6137	-0,0261	0,0034	1,9E-14	-0,0312	0,0140	0,0256	instrument
rs537124	7	28203142	C	T	0,0006	114,4888	0,0378	0,0035	9,9E-27	-0,0217	0,0146	0,1384	instrument
rs550373	6	116754150	T	G	0,0002	41,3702	0,0212	0,0033	1,3E-10	0,0220	0,0134	0,1001	instrument
rs56186137	16	28825953	G	A	0,0003	55,5914	0,0245	0,0033	8,9E-14	0,0059	0,0136	0,6641	instrument
rs57674902	20	4096741	T	C	0,0003	50,9791	0,0243	0,0034	9,3E-13	-0,0042	0,0144	0,7725	instrument
rs584961	11	75277628	A	G	0,0003	55,6063	0,0379	0,0051	8,9E-14	0,0111	0,0220	0,6126	instrument
rs58697961	17	1665424	A	G	0,0002	37,5520	0,0209	0,0034	8,9E-10	0,0153	0,0144	0,2853	instrument
rs60843830	2	286756	G	C	0,0002	33,7325	0,0198	0,0034	6,3E-09	-0,0211	0,0142	0,1356	instrument
rs61732778	3	187443314	A	G	0,0002	32,6837	0,0358	0,0063	1,1E-08	-0,0091	0,0260	0,7271	instrument
rs61734651	20	61451332	T	C	0,0003	51,0362	0,0488	0,0068	9,1E-13	-0,0351	0,0303	0,2474	instrument
rs62055545	17	43964561	T	C	0,0004	78,6761	-0,0345	0,0039	7,4E-19	0,1187	0,0164	5,1E-13	outlier
rs62076439	17	47404628	T	G	0,0004	73,1701	-0,0287	0,0034	1,2E-17	-0,0232	0,0136	0,0886	instrument
rs62271373	3	150066540	A	T	0,0002	38,9247	-0,0441	0,0071	4,4E-10	-0,0333	0,0311	0,2850	instrument
rs62330027	5	318557	A	T	0,0002	29,9863	0,0298	0,0054	4,4E-08	0,0172	0,0224	0,4424	instrument
rs62515452	8	57187356	T	C	0,0002	44,5285	0,0305	0,0046	2,5E-11	-0,0076	0,0202	0,7068	instrument
rs62621197	19	8670147	T	C	0,0011	211,1187	-0,1347	0,0093	8,2E-48	0,0250	0,0449	0,5772	instrument
rs6439629	3	135901270	A	G	0,0002	45,9408	-0,0247	0,0036	1,2E-11	0,0120	0,0149	0,4197	instrument
rs6486059	11	12824581	A	G	0,0003	49,8290	0,0232	0,0033	1,7E-12	-0,0210	0,0137	0,1237	instrument
rs6492538	13	91993746	A	C	0,0002	46,7441	0,0268	0,0039	8,1E-12	0,0069	0,0159	0,6648	instrument
rs6557679	8	23383743	T	G	0,0002	35,3070	0,0221	0,0037	2,8E-09	0,0016	0,0150	0,9129	instrument
rs6585827	10	124165615	A	G	0,0002	38,1920	0,0199	0,0032	6,4E-10	0,0151	0,0134	0,2582	instrument
rs6601503	8	10540767	C	T	0,0002	38,8250	0,0230	0,0037	4,6E-10	0,0219	0,0169	0,1941	instrument
rs6739701	2	105132332	G	A	0,0004	66,6829	0,0267	0,0033	3,2E-16	-0,0160	0,0141	0,2569	instrument
rs6777217	3	36979042	A	G	0,0002	33,0047	0,0187	0,0032	9,2E-09	0,0135	0,0134	0,3152	instrument

rs6785012	3	141109348	T	C	0,0015	291,7233	0,0555	0,0032	2,4E-65	-0,0060	0,0133	0,6519	instrument
rs68049170	10	72432047	A	G	0,0003	57,7746	0,0277	0,0036	2,9E-14	0,0134	0,0149	0,3694	instrument
rs6976501	7	50760220	G	A	0,0003	57,6227	0,0294	0,0039	3,2E-14	0,0379	0,0163	0,0200	instrument
rs6978655	7	46171683	T	C	0,0002	39,0246	0,0228	0,0036	4,2E-10	0,0167	0,0151	0,2688	instrument
rs6990354	8	145282578	C	T	0,0002	40,3856	-0,0206	0,0032	2,1E-10	-0,0126	0,0139	0,3670	instrument
rs702605	5	53167028	T	C	0,0002	33,7441	0,0188	0,0032	6,3E-09	-0,0099	0,0132	0,4531	instrument
rs7101582	11	47714610	A	G	0,0002	30,9133	0,0224	0,0040	2,7E-08	0,0089	0,0167	0,5911	instrument
rs711245	2	36768875	A	G	0,0002	46,4574	-0,0235	0,0034	9,4E-12	0,0009	0,0142	0,9495	instrument
rs7155279	14	92485881	T	G	0,0003	63,3291	-0,0267	0,0034	1,8E-15	0,0024	0,0137	0,8609	instrument
rs7170686	15	48821350	A	G	0,0003	56,0396	-0,0414	0,0055	7,1E-14	-0,0028	0,0231	0,9050	instrument
rs7184427	16	88804734	A	G	0,0002	30,0301	-0,0248	0,0045	4,3E-08	0,0006	0,0188	0,9762	instrument
rs7223156	17	68679731	T	C	0,0002	34,1637	-0,0206	0,0035	5,1E-09	-0,0294	0,0143	0,0395	instrument
rs72502575	6	31356087	T	C	0,0003	49,9561	-0,0319	0,0045	1,6E-12	-0,0240	0,0172	0,1646	instrument
rs72708236	1	155980320	G	T	0,0003	66,4707	-0,0773	0,0095	3,6E-16	-0,0183	0,0411	0,6558	instrument
rs72755233	15	100692953	A	G	0,0012	238,3911	-0,0784	0,0051	9,8E-54	-0,0402	0,0260	0,1224	instrument
rs72940413	11	69202148	T	C	0,0002	32,3530	-0,0320	0,0056	1,3E-08	-0,0115	0,0222	0,6045	instrument
rs73111557	3	61523251	T	C	0,0002	36,7596	-0,0325	0,0054	1,3E-09	-0,0247	0,0231	0,2848	instrument
rs73407643	6	35169123	C	T	0,0004	68,0618	-0,1022	0,0124	1,6E-16	0,0091	0,0513	0,8585	instrument
rs74469561	3	70929100	T	G	0,0002	39,7904	-0,0383	0,0061	2,8E-10	0,0458	0,0251	0,0679	instrument
rs74829909	4	12964932	C	T	0,0002	31,9448	-0,0305	0,0054	1,6E-08	0,0315	0,0227	0,1658	instrument
rs75223147	6	19831020	G	T	0,0004	81,5942	0,1015	0,0112	1,7E-19	0,0492	0,0659	0,4558	instrument
rs7534091	1	118864616	G	A	0,0003	54,6115	-0,0271	0,0037	1,5E-13	-0,0016	0,0151	0,9145	instrument
rs7536227	1	93262390	C	T	0,0002	31,2478	-0,0300	0,0054	2,3E-08	-0,0098	0,0223	0,6615	instrument
rs7540754	1	227850588	A	T	0,0004	76,0899	-0,0374	0,0043	2,7E-18	-0,0172	0,0182	0,3436	instrument
rs7674894	4	120680189	G	A	0,0002	37,3806	-0,0212	0,0035	9,7E-10	-0,0018	0,0143	0,9013	instrument
rs7689420	4	145568352	T	C	0,0011	211,4094	-0,0625	0,0043	7,1E-48	-0,0052	0,0179	0,7733	instrument
rs7718768	5	178536089	A	G	0,0004	79,9585	0,0305	0,0034	3,9E-19	-0,0227	0,0142	0,1103	instrument
rs7736102	5	131604257	G	A	0,0003	66,0149	-0,0264	0,0032	4,5E-16	-0,0098	0,0132	0,4601	instrument
rs7757703	6	47578705	G	A	0,0002	44,3685	-0,0235	0,0035	2,7E-11	0,0113	0,0143	0,4287	instrument
rs7804293	7	92287849	G	T	0,0004	78,9069	0,0325	0,0037	6,6E-19	0,0208	0,0148	0,1598	instrument
rs781659	4	57779851	G	A	0,0002	33,5817	-0,0189	0,0033	6,8E-09	-0,0117	0,0134	0,3802	instrument

rs78198962	2	233094868	T	C	0,0003	51,3797	0,0655	0,0091	7,6E-13	-0,0638	0,0465	0,1695	instrument
rs7846627	8	130736263	G	A	0,0003	58,7669	-0,0249	0,0032	1,8E-14	0,0172	0,0132	0,1932	instrument
rs7874161	9	109639062	T	C	0,0002	31,3373	0,0189	0,0034	2,2E-08	0,0039	0,0140	0,7819	instrument
rs79112217	1	218622949	C	T	0,0003	61,3239	0,0412	0,0053	4,9E-15	-0,0091	0,0223	0,6824	instrument
rs79312203	20	32082962	A	G	0,0002	37,6010	0,0329	0,0054	8,7E-10	0,0084	0,0222	0,7061	instrument
rs7952436	11	67024534	T	C	0,0009	172,6578	-0,0764	0,0058	1,9E-39	-0,0313	0,0292	0,2849	instrument
rs798488	7	2802522	C	T	0,0010	187,9621	-0,0482	0,0035	9,7E-43	-0,0222	0,0145	0,1256	instrument
rs7994151	13	115038879	G	A	0,0002	36,3846	-0,0271	0,0045	1,6E-09	-0,0226	0,0186	0,2244	instrument
rs8003703	14	33411639	A	T	0,0002	31,3149	-0,0637	0,0114	2,2E-08	0,0605	0,0511	0,2365	instrument
rs80085026	7	148636421	G	C	0,0004	77,0525	0,0364	0,0041	1,7E-18	0,0019	0,0173	0,9106	instrument
rs8103728	19	33900257	C	G	0,0004	70,8620	-0,0289	0,0034	3,8E-17	0,0054	0,0141	0,7025	instrument
rs8105888	19	19704909	T	A	0,0003	64,1915	0,0324	0,0040	1,1E-15	0,0296	0,0165	0,0721	instrument
rs862041	14	74986403	T	C	0,0004	72,1473	-0,0286	0,0034	2,0E-17	0,0107	0,0139	0,4408	instrument
rs9262136	6	30619988	A	C	0,0002	32,8211	-0,0264	0,0046	1,0E-08	0,0027	0,0181	0,8825	instrument
rs927415	6	7698384	T	C	0,0002	33,7325	-0,0194	0,0033	6,3E-09	0,0067	0,0135	0,6188	instrument
rs9285863	5	108071655	C	T	0,0003	51,5662	0,0246	0,0034	7,0E-13	0,0065	0,0141	0,6460	instrument
rs9321266	6	131316458	G	A	0,0003	56,3995	0,0249	0,0033	5,9E-14	0,0050	0,0136	0,7154	instrument
rs9344005	6	81173371	G	C	0,0002	40,1698	0,0318	0,0050	2,3E-10	-0,0121	0,0215	0,5734	instrument
rs9344115	6	81834237	A	G	0,0002	32,3303	-0,0208	0,0037	1,3E-08	0,0219	0,0150	0,1433	instrument
rs9358912	6	26211146	T	G	0,0012	231,9505	-0,0553	0,0036	2,4E-52	-0,0029	0,0144	0,8407	instrument
rs9442571	1	9349611	A	T	0,0003	66,2915	0,0396	0,0049	3,9E-16	-0,0174	0,0210	0,4068	instrument
rs979012	20	6623374	T	C	0,0004	78,9957	0,0297	0,0033	6,3E-19	0,0077	0,0139	0,5808	instrument
rs994014	4	82165790	C	T	0,0006	118,8088	0,0381	0,0035	1,2E-27	-0,0182	0,0143	0,2042	instrument

Table S14. Single Nucleotide Polymorphisms (SNPs) used as instruments in the Mendelian randomization analyses for the effect of TFR on ER- breast cancer based on the genome-wide significance threshold P = 5E-08. R^2 represents the explained variance in the exposure. SNP-specific instrument strength is given by the respective F-statistic. Some of the genetic variants were identified as outliers in the iterative approach and therefore excluded from the analyses.

SNP	CHR	POS	EA	OA	R^2	F-stat	β_{TFR}	SE_{TFR}	P_{TFR}	β_{ER-}	SE_{ER-}	P_{ER-}	variant$_{ER-}$
rs1002436	1	89146852	A	G	0,0003	48,0799	-0,0225	0,0032	4,1E-12	-0,0110	0,0115	0,3361	instrument
rs10059884	5	32832474	C	A	0,0002	45,7106	0,0222	0,0033	1,4E-11	0,0044	0,0121	0,7130	instrument
rs10152591	15	70048157	C	A	0,0003	52,0701	-0,0379	0,0053	5,4E-13	0,0044	0,0196	0,8220	instrument

rs10179126	2	165511794	G	C	0,0002	41,1261	0,0215	0,0034	1,4E-10	-0,0100	0,0116	0,3891	instrument
rs1020048	9	86666993	A	C	0,0003	54,2721	0,0308	0,0042	1,8E-13	0,0072	0,0144	0,6152	instrument
rs1026029	2	177065248	C	T	0,0002	32,7295	-0,0409	0,0071	1,1E-08	-0,0142	0,0268	0,5974	instrument
rs10760110	9	123412563	C	T	0,0002	33,6745	0,0204	0,0035	6,5E-09	0,0011	0,0127	0,9313	instrument
rs10761129	9	94486321	C	T	0,0002	41,9640	0,0222	0,0034	9,3E-11	0,0008	0,0130	0,9531	instrument
rs10823149	10	69935396	G	T	0,0002	38,5513	-0,0202	0,0032	5,3E-10	0,0013	0,0113	0,9075	instrument
rs10874671	1	103456389	G	C	0,0003	53,4209	0,0257	0,0035	2,7E-13	0,0006	0,0123	0,9607	instrument
rs10901171	9	133427194	C	T	0,0003	52,6200	-0,0242	0,0033	4,1E-13	0,0032	0,0123	0,7979	instrument
rs10933385	2	232916068	A	G	0,0003	57,3043	-0,0910	0,0120	3,7E-14	0,0396	0,0403	0,3267	instrument
rs11031005	11	30226356	C	T	0,0002	30,8244	0,0256	0,0046	2,8E-08	-0,0006	0,0162	0,9696	instrument
rs11130321	3	52762698	T	C	0,0003	60,2015	-0,0255	0,0033	8,6E-15	0,0145	0,0114	0,2036	instrument
rs11186507	10	92983831	G	A	0,0002	40,4873	0,0257	0,0040	2,0E-10	0,0115	0,0145	0,4295	instrument
rs111947781	3	38021115	T	C	0,0002	42,4187	0,0290	0,0045	7,4E-11	0,0027	0,0164	0,8706	instrument
rs11205303	1	149906413	C	T	0,0020	392,0359	0,0648	0,0033	3,6E-87	0,0404	0,0119	0,0007	outlier
rs11259936	15	84580582	A	C	0,0017	324,7170	-0,0581	0,0032	1,6E-72	-0,0041	0,0113	0,7128	instrument
rs112635299	14	94838142	T	G	0,0005	93,4886	0,1081	0,0112	4,1E-22	-0,0183	0,0454	0,6876	instrument
rs113059026	10	32158478	A	C	0,0002	38,8499	-0,0223	0,0036	4,6E-10	-0,0224	0,0125	0,0739	instrument
rs115912456	5	82815158	G	A	0,0005	86,3032	-0,0753	0,0081	1,6E-20	-0,0250	0,0295	0,3980	instrument
rs11689727	2	25458100	A	C	0,0006	106,5013	-0,0354	0,0034	6,0E-25	-0,0324	0,0128	0,0111	instrument
rs11717301	3	72397284	A	C	0,0003	58,9818	-0,0289	0,0038	1,6E-14	-0,0171	0,0135	0,2059	instrument
rs12129970	1	54877581	G	A	0,0002	34,2690	-0,0307	0,0052	4,8E-09	-0,0379	0,0212	0,0732	instrument
rs12172195	22	45813687	A	G	0,0003	50,8649	0,0324	0,0045	9,9E-13	0,0184	0,0160	0,2514	instrument
rs12409899	1	172227011	G	C	0,0004	85,0260	0,0363	0,0039	3,0E-20	0,0057	0,0137	0,6781	instrument
rs12507561	4	73464895	T	G	0,0007	127,2371	0,0365	0,0032	1,8E-29	0,0178	0,0118	0,1300	instrument
rs12685003	9	17022667	G	A	0,0002	34,9514	-0,0304	0,0051	3,4E-09	0,0015	0,0190	0,9359	instrument
rs12756815	1	113207243	A	C	0,0004	76,3518	-0,0349	0,0040	2,4E-18	0,0064	0,0141	0,6509	instrument
rs13107395	4	8601846	C	T	0,0003	62,2988	-0,0258	0,0033	3,0E-15	-0,0029	0,0124	0,8185	instrument
rs13128180	4	56286188	T	G	0,0003	51,6668	0,0253	0,0035	6,6E-13	0,0188	0,0121	0,1189	instrument
rs1313237	14	60848224	C	A	0,0002	46,4710	0,0226	0,0033	9,3E-12	0,0105	0,0117	0,3654	instrument
rs13144767	4	17974219	G	A	0,0004	68,3922	-0,0395	0,0048	1,3E-16	0,0336	0,0166	0,0434	instrument
rs13170063	5	157895013	G	A	0,0002	45,5485	-0,0227	0,0034	1,5E-11	-0,0159	0,0118	0,1764	instrument

rs13306455	19	7184721	T	C	0,0002	37,7115	0,0264	0,0043	8,2E-10	-0,0139	0,0152	0,3609	instrument
rs13429301	2	242061508	C	A	0,0002	32,8326	0,0330	0,0058	1,0E-08	0,0072	0,0208	0,7310	instrument
rs138809270	2	219929394	T	C	0,0003	52,6925	-0,0952	0,0131	3,9E-13	-0,0528	0,0467	0,2584	instrument
rs1388900	16	73800629	T	C	0,0002	33,4774	-0,0208	0,0036	7,2E-09	-0,0067	0,0129	0,6054	instrument
rs1421896	5	141016288	T	G	0,0002	30,5032	0,0183	0,0033	3,3E-08	-0,0150	0,0119	0,2054	instrument
rs142599545	6	19844384	G	A	0,0002	37,4540	0,0812	0,0133	9,4E-10	0,0605	0,0678	0,3721	instrument
rs143384	20	34025756	G	A	0,0011	215,2067	0,0482	0,0033	1,1E-48	-0,0221	0,0116	0,0558	instrument
rs143840904	11	2813322	T	C	0,0003	60,4811	-0,0978	0,0126	7,4E-15	-0,0093	0,0587	0,8738	instrument
rs1452815	8	78123634	G	A	0,0002	35,6764	0,0213	0,0036	2,3E-09	0,0074	0,0125	0,5561	instrument
rs1513304	3	99269750	C	T	0,0002	44,5418	0,0236	0,0035	2,5E-11	0,0281	0,0133	0,0345	instrument
rs1528589	5	88748768	C	T	0,0002	43,3617	0,0222	0,0034	4,6E-11	0,0171	0,0119	0,1490	instrument
rs1550436	15	74221157	T	C	0,0006	111,7237	-0,0343	0,0032	4,0E-26	0,0085	0,0113	0,4513	instrument
rs16942324	15	89383854	A	C	0,0005	104,6518	-0,1025	0,0100	1,4E-24	-0,0129	0,0425	0,7621	instrument
rs16953461	17	46261736	G	T	0,0003	62,7574	-0,0312	0,0039	2,3E-15	-0,0071	0,0139	0,6075	instrument
rs16996444	19	9997238	C	T	0,0002	47,0866	-0,0245	0,0036	6,8E-12	0,0088	0,0126	0,4841	instrument
rs17194431	8	120612627	T	C	0,0002	29,9206	0,0196	0,0036	4,5E-08	-0,0065	0,0127	0,6092	instrument
rs1749824	10	80923862	A	C	0,0002	32,5011	-0,0185	0,0032	1,2E-08	0,0206	0,0114	0,0710	instrument
rs17511102	2	37960613	T	A	0,0002	34,6446	0,0331	0,0056	4,0E-09	-0,0139	0,0234	0,5518	instrument
rs17654949	2	219890955	T	C	0,0003	51,3940	0,0617	0,0086	7,6E-13	-0,0083	0,0326	0,7996	instrument
rs1805007	16	89986117	T	C	0,0003	56,8359	-0,0395	0,0052	4,7E-14	-0,0430	0,0227	0,0579	instrument
rs194059	5	56197416	G	A	0,0003	48,9575	-0,0238	0,0034	2,6E-12	0,0490	0,0117	2,9E-05	outlier
rs1952256	1	184035116	G	A	0,0006	106,9145	0,0351	0,0034	4,6E-25	0,0273	0,0118	0,0212	instrument
rs1966265	5	176516631	A	G	0,0003	51,8251	0,0268	0,0037	6,1E-13	-0,0055	0,0135	0,6843	instrument
rs1980030	10	120970027	G	A	0,0003	47,7476	0,0227	0,0033	4,8E-12	0,0011	0,0120	0,9257	instrument
rs2006122	17	61987405	T	A	0,0003	47,1827	0,0252	0,0037	6,5E-12	0,0003	0,0129	0,9840	instrument
rs200889152	6	117507982	C	A	0,0002	42,3146	-0,0218	0,0034	7,8E-11	0,0211	0,0118	0,0741	instrument
rs2035901	4	145521867	G	A	0,0002	30,3267	0,0179	0,0032	3,7E-08	-0,0063	0,0118	0,5940	instrument
rs2050157	6	142658162	A	G	0,0008	146,8929	-0,0434	0,0036	8,4E-34	0,0088	0,0125	0,4798	instrument
rs2076112	22	38538822	T	C	0,0003	64,3999	-0,0261	0,0032	1,0E-15	0,0143	0,0113	0,2037	instrument
rs2194411	3	185548663	A	G	0,0002	42,9020	0,0324	0,0049	5,8E-11	-0,0126	0,0187	0,5013	instrument
rs239458	8	37213496	G	A	0,0002	29,8441	-0,0315	0,0058	4,7E-08	-0,0055	0,0204	0,7862	instrument

rs256904	5	55810305	A	T	0,0005	87,0667	0,0345	0,0037	1,1E-20	-0,0132	0,0135	0,3284	instrument
rs264762	5	64384386	A	C	0,0003	53,4648	-0,0250	0,0034	2,6E-13	0,0165	0,0120	0,1682	instrument
rs2712169	2	217671349	G	A	0,0002	44,7289	0,0221	0,0033	2,3E-11	-0,0070	0,0116	0,5446	instrument
rs2722285	7	37994955	T	C	0,0002	32,0466	-0,0286	0,0051	1,5E-08	0,0114	0,0185	0,5372	instrument
rs2731340	3	13559829	C	T	0,0002	47,4440	0,0370	0,0054	5,7E-12	-0,0268	0,0196	0,1716	instrument
rs273945	7	137611566	A	C	0,0002	38,3157	-0,0202	0,0033	6,0E-10	-0,0004	0,0114	0,9752	instrument
rs2763265	6	168814600	G	A	0,0002	39,0371	-0,0239	0,0038	4,2E-10	0,0033	0,0131	0,8001	instrument
rs2797963	6	34185749	C	T	0,0005	88,1148	0,0556	0,0059	6,2E-21	-0,0191	0,0217	0,3772	instrument
rs2811462	3	129303436	G	T	0,0003	53,0561	0,0282	0,0039	3,2E-13	0,0206	0,0139	0,1381	instrument
rs2820443	1	219753509	C	T	0,0006	109,8293	0,0369	0,0035	1,0E-25	0,0063	0,0127	0,6187	instrument
rs2823989	21	18080943	G	A	0,0002	32,8326	-0,0197	0,0034	1,0E-08	-0,0048	0,0121	0,6908	instrument
rs284660	19	41932084	G	T	0,0003	49,9278	0,0233	0,0033	1,6E-12	-0,0029	0,0122	0,8140	instrument
rs28539246	17	29226630	A	T	0,0003	65,3180	-0,0268	0,0033	6,4E-16	-0,0330	0,0116	0,0046	outlier
rs2911450	16	88987497	A	C	0,0002	40,2967	-0,0216	0,0034	2,2E-10	0,0094	0,0129	0,4672	instrument
rs3020421	6	152348122	A	G	0,0003	47,8444	0,0240	0,0035	4,6E-12	-0,0165	0,0120	0,1691	instrument
rs34134267	7	20417034	A	C	0,0005	86,0247	-0,0304	0,0033	1,8E-20	0,0118	0,0114	0,2990	instrument
rs34517439	1	78450517	A	C	0,0002	30,6138	0,0276	0,0050	3,1E-08	0,0054	0,0196	0,7819	instrument
rs34831515	19	17275777	T	C	0,0002	38,8748	-0,0239	0,0038	4,5E-10	-0,0155	0,0134	0,2467	instrument
rs34919557	20	32510282	T	C	0,0002	31,5616	-0,0315	0,0056	1,9E-08	0,0056	0,0194	0,7736	instrument
rs35307904	9	78511889	A	G	0,0007	137,8261	-0,0584	0,0050	8,0E-32	0,0021	0,0190	0,9117	instrument
rs35699973	18	20726731	G	A	0,0006	113,8477	-0,0419	0,0039	1,5E-26	0,0140	0,0141	0,3202	instrument
rs35874463	15	67457698	G	A	0,0005	91,8329	0,0663	0,0069	9,6E-22	0,0120	0,0265	0,6505	instrument
rs3738814	1	17331676	G	A	0,0005	102,4133	-0,0329	0,0033	4,4E-24	-0,0082	0,0113	0,4676	instrument
rs3791679	2	56096892	G	A	0,0012	235,3131	-0,0591	0,0038	4,4E-53	0,0094	0,0131	0,4732	instrument
rs3794782	17	18127914	C	G	0,0002	35,3427	0,0198	0,0033	2,8E-09	-0,0161	0,0116	0,1657	instrument
rs3800162	6	2134391	A	T	0,0003	53,6990	-0,0241	0,0033	2,3E-13	-0,0137	0,0120	0,2540	instrument
rs395523	6	105396187	G	C	0,0006	123,6531	0,0384	0,0035	1,1E-28	-0,0093	0,0121	0,4408	instrument
rs39875	16	2268737	G	A	0,0002	32,2620	0,0214	0,0038	1,4E-08	-0,0237	0,0132	0,0736	instrument
rs4073717	5	170864021	T	G	0,0002	35,3308	-0,0240	0,0040	2,8E-09	0,0139	0,0145	0,3376	instrument
rs41284816	13	50655989	T	G	0,0003	52,9251	0,0890	0,0122	3,5E-13	0,0410	0,0376	0,2756	instrument
rs4321401	2	69348216	T	C	0,0003	49,0975	-0,0227	0,0032	2,4E-12	-0,0114	0,0123	0,3548	instrument

rs4383453	3	123068359	A	G	0,0002	30,7800	0,0229	0,0041	2,9E-08	0,0019	0,0153	0,8989	instrument
rs4421120	5	32763118	A	G	0,0004	78,0034	0,0387	0,0044	1,0E-18	0,0109	0,0163	0,5022	instrument
rs4432932	5	115039414	G	A	0,0002	39,5888	0,0230	0,0037	3,1E-10	0,0147	0,0125	0,2409	instrument
rs4513485	3	49139966	G	A	0,0003	60,8238	-0,0266	0,0034	6,3E-15	-0,0134	0,0122	0,2720	instrument
rs4626892	1	221234485	T	C	0,0002	44,7824	0,0218	0,0033	2,2E-11	0,0105	0,0118	0,3741	instrument
rs4699384	4	100570681	A	T	0,0002	32,4783	0,0234	0,0041	1,2E-08	0,0136	0,0145	0,3476	instrument
rs4731420	7	127863295	C	G	0,0002	30,8910	0,0234	0,0042	2,7E-08	-0,0125	0,0155	0,4194	instrument
rs475390	1	41570459	G	A	0,0002	32,3416	0,0221	0,0039	1,3E-08	-0,0439	0,0136	0,0013	outlier
rs4777230	15	70365762	C	T	0,0002	35,9636	0,0274	0,0046	2,0E-09	0,0269	0,0168	0,1081	instrument
rs4793987	17	46855837	C	T	0,0002	36,5175	-0,0202	0,0033	1,5E-09	0,0043	0,0116	0,7113	instrument
rs481237	9	98316094	A	G	0,0003	59,7059	-0,0470	0,0061	1,1E-14	0,0209	0,0238	0,3785	instrument
rs4842133	9	139127566	A	C	0,0005	93,9145	0,0348	0,0036	3,3E-22	0,0264	0,0133	0,0479	instrument
rs4848645	2	121623691	G	C	0,0002	30,7467	-0,0240	0,0043	3,0E-08	-0,0075	0,0170	0,6598	instrument
rs4867721	5	172983457	G	T	0,0002	38,5637	0,0205	0,0033	5,3E-10	0,0168	0,0116	0,1461	instrument
rs4901541	14	55235282	C	T	0,0002	39,4757	-0,0208	0,0033	3,3E-10	0,0018	0,0126	0,8882	instrument
rs4923575	11	28876429	C	T	0,0002	40,3729	-0,0207	0,0033	2,1E-10	0,0007	0,0115	0,9483	instrument
rs4936099	11	130280725	C	A	0,0002	42,7188	-0,0216	0,0033	6,3E-11	0,0228	0,0126	0,0702	instrument
rs4961091	8	89357874	T	A	0,0002	36,3243	0,0213	0,0035	1,7E-09	0,0151	0,0122	0,2171	instrument
rs496298	10	131355130	A	G	0,0003	58,6137	-0,0261	0,0034	1,9E-14	-0,0137	0,0121	0,2569	instrument
rs537124	7	28203142	C	T	0,0006	114,4888	0,0378	0,0035	9,9E-27	0,0043	0,0125	0,7276	instrument
rs550373	6	116754150	T	G	0,0002	41,3702	0,0212	0,0033	1,3E-10	-0,0054	0,0115	0,6366	instrument
rs56186137	16	28825953	G	A	0,0003	55,5914	0,0245	0,0033	8,9E-14	-0,0054	0,0116	0,6412	instrument
rs57674902	20	4096741	T	C	0,0003	50,9791	0,0243	0,0034	9,3E-13	0,0065	0,0128	0,6119	instrument
rs584961	11	75277628	A	G	0,0003	55,6063	0,0379	0,0051	8,9E-14	0,0122	0,0198	0,5372	instrument
rs58697961	17	1665424	A	G	0,0002	37,5520	0,0209	0,0034	8,9E-10	-0,0013	0,0124	0,9167	instrument
rs60843830	2	286756	G	C	0,0002	33,7325	0,0198	0,0034	6,3E-09	0,0257	0,0121	0,0333	instrument
rs61732778	3	187443314	A	G	0,0002	32,6837	0,0358	0,0063	1,1E-08	-0,0082	0,0233	0,7254	instrument
rs61734651	20	61451332	T	C	0,0003	51,0362	0,0488	0,0068	9,1E-13	-0,0114	0,0273	0,6767	instrument
rs62055545	17	43964561	T	C	0,0004	78,6761	-0,0345	0,0039	7,4E-19	-0,0374	0,0139	0,0070	outlier
rs62076439	17	47404628	T	G	0,0004	73,1701	-0,0287	0,0034	1,2E-17	0,0081	0,0117	0,4886	instrument
rs62271373	3	150066540	A	T	0,0002	38,9247	-0,0441	0,0071	4,4E-10	-0,0003	0,0293	0,9923	instrument

rs62330027	5	318557	A	T	0,0002	29,9863	0,0298	0,0054	4,4E-08	0,0398	0,0198	0,0450	instrument
rs62515452	8	57187356	T	C	0,0002	44,5285	0,0305	0,0046	2,5E-11	-0,0048	0,0181	0,7913	instrument
rs62621197	19	8670147	T	C	0,0011	211,1187	-0,1347	0,0093	8,2E-48	0,0331	0,0403	0,4108	instrument
rs6439629	3	135901270	A	G	0,0002	45,9408	-0,0247	0,0036	1,2E-11	-0,0067	0,0128	0,5985	instrument
rs6486059	11	12824581	A	G	0,0003	49,8290	0,0232	0,0033	1,7E-12	-0,0062	0,0118	0,5977	instrument
rs6492538	13	91993746	A	C	0,0002	46,7441	0,0268	0,0039	8,1E-12	0,0031	0,0136	0,8189	instrument
rs6557679	8	23383743	T	G	0,0002	35,3070	0,0221	0,0037	2,8E-09	0,0066	0,0127	0,6071	instrument
rs6585827	10	124165615	A	G	0,0002	38,1920	0,0199	0,0032	6,4E-10	0,0091	0,0116	0,4340	instrument
rs6601503	8	10540767	C	T	0,0002	38,8250	0,0230	0,0037	4,6E-10	0,0148	0,0143	0,2986	instrument
rs6739701	2	105132332	G	A	0,0004	66,6829	0,0267	0,0033	3,2E-16	-0,0085	0,0127	0,5048	instrument
rs6777217	3	36979042	A	G	0,0002	33,0047	0,0187	0,0032	9,2E-09	0,0263	0,0114	0,0214	instrument
rs6785012	3	141109348	T	C	0,0015	291,7233	0,0555	0,0032	2,4E-65	0,0150	0,0114	0,1861	instrument
rs68049170	10	72432047	A	G	0,0003	57,7746	0,0277	0,0036	2,9E-14	0,0112	0,0128	0,3792	instrument
rs6976501	7	50760220	G	A	0,0003	57,6227	0,0294	0,0039	3,2E-14	-0,0081	0,0142	0,5690	instrument
rs6978655	7	46171683	T	C	0,0002	39,0246	0,0228	0,0036	4,2E-10	0,0094	0,0133	0,4808	instrument
rs6990354	8	145282578	C	T	0,0002	40,3856	-0,0206	0,0032	2,1E-10	0,0142	0,0126	0,2603	instrument
rs702605	5	53167028	T	C	0,0002	33,7441	0,0188	0,0032	6,3E-09	-0,0177	0,0112	0,1150	instrument
rs7101582	11	47714610	A	G	0,0002	30,9133	0,0224	0,0040	2,7E-08	-0,0011	0,0142	0,9388	instrument
rs711245	2	36768875	A	G	0,0002	46,4574	-0,0235	0,0034	9,4E-12	-0,0018	0,0122	0,8854	instrument
rs7155279	14	92485881	T	G	0,0003	63,3291	-0,0267	0,0034	1,8E-15	-0,0083	0,0117	0,4812	instrument
rs7170686	15	48821350	A	G	0,0003	56,0396	-0,0414	0,0055	7,1E-14	0,0420	0,0207	0,0423	instrument
rs7184427	16	88804734	A	G	0,0002	30,0301	-0,0248	0,0045	4,3E-08	0,0136	0,0164	0,4062	instrument
rs7223156	17	68679731	T	C	0,0002	34,1637	-0,0206	0,0035	5,1E-09	0,0069	0,0123	0,5756	instrument
rs72502575	6	31356087	T	C	0,0003	49,9561	-0,0319	0,0045	1,6E-12	-0,0028	0,0147	0,8512	instrument
rs72708236	1	155980320	G	T	0,0003	66,4707	-0,0773	0,0095	3,6E-16	0,0054	0,0369	0,8842	instrument
rs72755233	15	100692953	A	G	0,0012	238,3911	-0,0784	0,0051	9,8E-54	-0,0090	0,0234	0,7019	instrument
rs72940413	11	69202148	T	C	0,0002	32,3530	-0,0320	0,0056	1,3E-08	0,0001	0,0189	0,9951	instrument
rs73111557	3	61523251	T	C	0,0002	36,7596	-0,0325	0,0054	1,3E-09	-0,0028	0,0203	0,8912	instrument
rs73407643	6	35169123	C	T	0,0004	68,0618	-0,1022	0,0124	1,6E-16	0,0573	0,0446	0,1983	instrument
rs74469561	3	70929100	T	G	0,0002	39,7904	-0,0383	0,0061	2,8E-10	0,0173	0,0224	0,4397	instrument
rs74829909	4	12964932	C	T	0,0002	31,9448	-0,0305	0,0054	1,6E-08	-0,0091	0,0201	0,6501	instrument

rs75223147	6	19831020	G	T	0,0004	81,5942	0,1015	0,0112	1,7E-19	-0,1286	0,0674	0,0563	instrument
rs7534091	1	118864616	G	A	0,0003	54,6115	-0,0271	0,0037	1,5E-13	-0,0153	0,0131	0,2427	instrument
rs7536227	1	93262390	C	T	0,0002	31,2478	-0,0300	0,0054	2,3E-08	0,0226	0,0188	0,2281	instrument
rs7540754	1	227850588	A	T	0,0004	76,0899	-0,0374	0,0043	2,7E-18	-0,0026	0,0156	0,8685	instrument
rs7674894	4	120680189	G	A	0,0002	37,3806	-0,0212	0,0035	9,7E-10	0,0258	0,0122	0,0346	instrument
rs7689420	4	145568352	T	C	0,0011	211,4094	-0,0625	0,0043	7,1E-48	0,0147	0,0152	0,3338	instrument
rs7718768	5	178536089	A	G	0,0004	79,9585	0,0305	0,0034	3,9E-19	0,0146	0,0123	0,2344	instrument
rs7736102	5	131604257	G	A	0,0003	66,0149	-0,0264	0,0032	4,5E-16	-0,0025	0,0113	0,8252	instrument
rs7757703	6	47578705	G	A	0,0002	44,3685	-0,0235	0,0035	2,7E-11	0,0103	0,0122	0,4008	instrument
rs7804293	7	92287849	G	T	0,0004	78,9069	0,0325	0,0037	6,6E-19	0,0229	0,0125	0,0665	instrument
rs781659	4	57779851	G	A	0,0002	33,5817	-0,0189	0,0033	6,8E-09	-0,0063	0,0115	0,5805	instrument
rs78198962	2	233094868	T	C	0,0003	51,3797	0,0655	0,0091	7,6E-13	-0,0185	0,0437	0,6720	instrument
rs7846627	8	130736263	G	A	0,0003	58,7669	-0,0249	0,0032	1,8E-14	-0,0062	0,0112	0,5820	instrument
rs7874161	9	109639062	T	C	0,0002	31,3373	0,0189	0,0034	2,2E-08	0,0021	0,0122	0,8632	instrument
rs79112217	1	218622949	C	T	0,0003	61,3239	0,0412	0,0053	4,9E-15	0,0100	0,0191	0,5999	instrument
rs79312203	20	32082962	A	G	0,0002	37,6010	0,0329	0,0054	8,7E-10	-0,0212	0,0192	0,2692	instrument
rs7952436	11	67024534	T	C	0,0009	172,6578	-0,0764	0,0058	1,9E-39	0,0077	0,0265	0,7712	instrument
rs798488	7	2802522	C	T	0,0010	187,9621	-0,0482	0,0035	9,7E-43	0,0019	0,0126	0,8769	instrument
rs7994151	13	115038879	G	A	0,0002	36,3846	-0,0271	0,0045	1,6E-09	-0,0041	0,0158	0,7980	instrument
rs8003703	14	33411639	A	T	0,0002	31,3149	-0,0637	0,0114	2,2E-08	-0,0140	0,0471	0,7658	instrument
rs80085026	7	148636421	G	C	0,0004	77,0525	0,0364	0,0041	1,7E-18	0,0030	0,0152	0,8416	instrument
rs8103728	19	33900257	C	G	0,0004	70,8620	-0,0289	0,0034	3,8E-17	0,0070	0,0122	0,5672	instrument
rs8105888	19	19704909	T	A	0,0003	64,1915	0,0324	0,0040	1,1E-15	0,0229	0,0141	0,1048	instrument
rs862041	14	74986403	T	C	0,0004	72,1473	-0,0286	0,0034	2,0E-17	0,0004	0,0120	0,9724	instrument
rs9262136	6	30619988	A	C	0,0002	32,8211	-0,0264	0,0046	1,0E-08	-0,0227	0,0158	0,1496	instrument
rs927415	6	7698384	T	C	0,0002	33,7325	-0,0194	0,0033	6,3E-09	-0,0126	0,0116	0,2781	instrument
rs9285863	5	108071655	C	T	0,0003	51,5662	0,0246	0,0034	7,0E-13	0,0183	0,0121	0,1310	instrument
rs9321266	6	131316458	G	A	0,0003	56,3995	0,0249	0,0033	5,9E-14	-0,0041	0,0116	0,7251	instrument
rs9344005	6	81173371	G	C	0,0002	40,1698	0,0318	0,0050	2,3E-10	-0,0372	0,0184	0,0428	instrument
rs9344115	6	81834237	A	G	0,0002	32,3303	-0,0208	0,0037	1,3E-08	-0,0024	0,0128	0,8505	instrument
rs9358912	6	26211146	T	G	0,0012	231,9505	-0,0553	0,0036	2,4E-52	0,0153	0,0123	0,2127	instrument

rs9442571	1	9349611	A	T	0,0003	66,2915	0,0396	0,0049	3,9E-16	0,0329	0,0184	0,0743	instrument
rs979012	20	6623374	T	C	0,0004	78,9957	0,0297	0,0033	6,3E-19	0,0246	0,0120	0,0411	instrument
rs994014	4	82165790	C	T	0,0006	118,8088	0,0381	0,0035	1,2E-27	-0,0095	0,0124	0,4442	instrument

Table S15. Single Nucleotide Polymorphisms (SNPs) used as instruments in the Mendelian randomization analyses for the effect of TFR on ER+ breast cancer based on the genome-wide significance threshold P = 5E-08. R^2 represents the explained variance in the exposure. SNP-specific instrument strength is given by the respective F-statistic. Some of the genetic variants were identified as outliers in the iterative approach and therefore excluded from the analyses.

SNP	CHR	POS	EA	OA	R^2	F-stat	β_{TFR}	SE_{TFR}	P_{TFR}	β_{ER+}	SE_{ER+}	P_{ER+}	$variant_{ER+}$
rs1002436	1	89146852	A	G	0,0003	48,0799	-0,0225	0,0032	4,1E-12	-0,0182	0,0075	0,0157	instrument
rs10059884	5	32832474	C	A	0,0002	45,7106	0,0222	0,0033	1,4E-11	-0,0051	0,0079	0,5246	instrument
rs10152591	15	70048157	C	A	0,0003	52,0701	-0,0379	0,0053	5,4E-13	0,0034	0,0128	0,7885	instrument
rs10179126	2	165511794	G	C	0,0002	41,1261	0,0215	0,0034	1,4E-10	-0,0018	0,0076	0,8116	instrument
rs1020048	9	86666993	A	C	0,0003	54,2721	0,0308	0,0042	1,8E-13	0,0058	0,0094	0,5354	instrument
rs1026029	2	177065248	C	T	0,0002	32,7295	-0,0409	0,0071	1,1E-08	0,0001	0,0172	0,9977	instrument
rs10760110	9	123412563	C	T	0,0002	33,6745	0,0204	0,0035	6,5E-09	0,0045	0,0083	0,5835	instrument
rs10761129	9	94486321	C	T	0,0002	41,9640	0,0222	0,0034	9,3E-11	0,0081	0,0086	0,3461	instrument
rs10823149	10	69935396	G	T	0,0002	38,5513	-0,0202	0,0032	5,3E-10	-0,0029	0,0074	0,6914	instrument
rs10874671	1	103456389	G	C	0,0003	53,4209	0,0257	0,0035	2,7E-13	-0,0091	0,0080	0,2592	instrument
rs10901171	9	133427194	C	T	0,0003	52,6200	-0,0242	0,0033	4,1E-13	-0,0007	0,0082	0,9284	instrument
rs10933385	2	232916068	A	G	0,0003	57,3043	-0,0910	0,0120	3,7E-14	0,0117	0,0263	0,6563	instrument
rs11031005	11	30226356	C	T	0,0002	30,8244	0,0256	0,0046	2,8E-08	0,0223	0,0104	0,0326	instrument
rs11130321	3	52762698	T	C	0,0003	60,2015	-0,0255	0,0033	8,6E-15	-0,0104	0,0075	0,1621	instrument
rs11186507	10	92983831	G	A	0,0002	40,4873	0,0257	0,0040	2,0E-10	-0,0062	0,0095	0,5149	instrument
rs111947781	3	38021115	T	C	0,0002	42,4187	0,0290	0,0045	7,4E-11	-0,0026	0,0107	0,8086	instrument
rs11205303	1	149906413	C	T	0,0020	392,0359	0,0648	0,0033	3,6E-87	0,0572	0,0077	8,4E-14	outlier
rs11259936	15	84580582	A	C	0,0017	324,7170	-0,0581	0,0032	1,6E-72	0,0084	0,0074	0,2536	instrument
rs112635299	14	94838142	T	G	0,0005	93,4886	0,1081	0,0112	4,1E-22	-0,0745	0,0288	0,0097	outlier
rs113059026	10	32158478	A	C	0,0002	38,8499	-0,0223	0,0036	4,6E-10	-0,0262	0,0082	0,0014	outlier
rs115912456	5	82815158	G	A	0,0005	86,3032	-0,0753	0,0081	1,6E-20	-0,0150	0,0193	0,4367	instrument
rs11689727	2	25458100	A	C	0,0006	106,5013	-0,0354	0,0034	6,0E-25	-0,0136	0,0084	0,1050	instrument
rs11717301	3	72397284	A	C	0,0003	58,9818	-0,0289	0,0038	1,6E-14	-0,0109	0,0088	0,2154	instrument

rs12129970	1	54877581	G	A	0,0002	34,2690	-0,0307	0,0052	4,8E-09	0,0056	0,0139	0,6869	instrument
rs12172195	22	45813687	A	G	0,0003	50,8649	0,0324	0,0045	9,9E-13	0,0106	0,0105	0,3140	instrument
rs12409899	1	172227011	G	C	0,0004	85,0260	0,0363	0,0039	3,0E-20	-0,0175	0,0090	0,0527	instrument
rs12507561	4	73464895	T	G	0,0007	127,2371	0,0365	0,0032	1,8E-29	0,0103	0,0078	0,1836	instrument
rs12685003	9	17022667	G	A	0,0002	34,9514	-0,0304	0,0051	3,4E-09	-0,0135	0,0124	0,2767	instrument
rs12756815	1	113207243	A	C	0,0004	76,3518	-0,0349	0,0040	2,4E-18	-0,0021	0,0091	0,8170	instrument
rs13107395	4	8601846	C	T	0,0003	62,2988	-0,0258	0,0033	3,0E-15	-0,0091	0,0080	0,2561	instrument
rs13128180	4	56286188	T	G	0,0003	51,6668	0,0253	0,0035	6,6E-13	-0,0048	0,0079	0,5484	instrument
rs1313237	14	60848224	C	A	0,0002	46,4710	0,0226	0,0033	9,3E-12	-0,0007	0,0076	0,9245	instrument
rs13144767	4	17974219	G	A	0,0004	68,3922	-0,0395	0,0048	1,3E-16	-0,0156	0,0110	0,1578	instrument
rs13170063	5	157895013	G	A	0,0002	45,5485	-0,0227	0,0034	1,5E-11	-0,0103	0,0077	0,1806	instrument
rs13306455	19	7184721	T	C	0,0002	37,7115	0,0264	0,0043	8,2E-10	-0,0045	0,0099	0,6469	instrument
rs13429301	2	242061508	C	A	0,0002	32,8326	0,0330	0,0058	1,0E-08	0,0098	0,0136	0,4721	instrument
rs138809270	2	219929394	T	C	0,0003	52,6925	-0,0952	0,0131	3,9E-13	-0,0138	0,0294	0,6383	instrument
rs1388900	16	73800629	T	C	0,0002	33,4774	-0,0208	0,0036	7,2E-09	-0,0014	0,0085	0,8679	instrument
rs1421896	5	141016288	T	G	0,0002	30,5032	0,0183	0,0033	3,3E-08	0,0021	0,0078	0,7906	instrument
rs142599545	6	19844384	G	A	0,0002	37,4540	0,0812	0,0133	9,4E-10	0,0283	0,0438	0,5183	instrument
rs143384	20	34025756	G	A	0,0011	215,2067	0,0482	0,0033	1,1E-48	0,0019	0,0076	0,8018	instrument
rs143840904	11	2813322	T	C	0,0003	60,4811	-0,0978	0,0126	7,4E-15	-0,0338	0,0385	0,3797	instrument
rs1452815	8	78123634	G	A	0,0002	35,6764	0,0213	0,0036	2,3E-09	0,0230	0,0082	0,0049	outlier
rs1513304	3	99269750	C	T	0,0002	44,5418	0,0236	0,0035	2,5E-11	0,0317	0,0087	0,0003	outlier
rs1528589	5	88748768	C	T	0,0002	43,3617	0,0222	0,0034	4,6E-11	0,0019	0,0078	0,8029	instrument
rs1550436	15	74221157	T	C	0,0006	111,7237	-0,0343	0,0032	4,0E-26	0,0139	0,0074	0,0595	instrument
rs16942324	15	89383854	A	C	0,0005	104,6518	-0,1025	0,0100	1,4E-24	0,0341	0,0272	0,2091	instrument
rs16953461	17	46261736	G	T	0,0003	62,7574	-0,0312	0,0039	2,3E-15	0,0126	0,0091	0,1631	instrument
rs16996444	19	9997238	C	T	0,0002	47,0866	-0,0245	0,0036	6,8E-12	-0,0005	0,0083	0,9485	instrument
rs17194431	8	120612627	T	C	0,0002	29,9206	0,0196	0,0036	4,5E-08	-0,0033	0,0083	0,6908	instrument
rs1749824	10	80923862	A	C	0,0002	32,5011	-0,0185	0,0032	1,2E-08	0,0144	0,0075	0,0543	instrument
rs17511102	2	37960613	T	A	0,0002	34,6446	0,0331	0,0056	4,0E-09	-0,0005	0,0153	0,9743	instrument
rs17654949	2	219890955	T	C	0,0003	51,3940	0,0617	0,0086	7,6E-13	0,0468	0,0207	0,0241	instrument
rs1805007	16	89986117	T	C	0,0003	56,8359	-0,0395	0,0052	4,7E-14	0,0018	0,0143	0,8975	instrument

rs194059	5	56197416	G	A	0,0003	48,9575	-0,0238	0,0034	2,6E-12	0,1162	0,0076	0,0000	outlier
rs1952256	1	184035116	G	A	0,0006	106,9145	0,0351	0,0034	4,6E-25	0,0083	0,0078	0,2846	instrument
rs1966265	5	176516631	A	G	0,0003	51,8251	0,0268	0,0037	6,1E-13	-0,0223	0,0088	0,0111	outlier
rs1980030	10	120970027	G	A	0,0003	47,7476	0,0227	0,0033	4,8E-12	0,0079	0,0079	0,3159	instrument
rs2006122	17	61987405	T	A	0,0003	47,1827	0,0252	0,0037	6,5E-12	0,0100	0,0084	0,2341	instrument
rs200889152	6	117507982	C	A	0,0002	42,3146	-0,0218	0,0034	7,8E-11	0,0047	0,0077	0,5473	instrument
rs2035901	4	145521867	G	A	0,0002	30,3267	0,0179	0,0032	3,7E-08	0,0077	0,0078	0,3221	instrument
rs2050157	6	142658162	A	G	0,0008	146,8929	-0,0434	0,0036	8,4E-34	-0,0018	0,0082	0,8209	instrument
rs2076112	22	38538822	T	C	0,0003	64,3999	-0,0261	0,0032	1,0E-15	0,0346	0,0074	2,6E-06	outlier
rs2194411	3	185548663	A	G	0,0002	42,9020	0,0324	0,0049	5,8E-11	0,0031	0,0122	0,8021	instrument
rs239458	8	37213496	G	A	0,0002	29,8441	-0,0315	0,0058	4,7E-08	-0,0028	0,0134	0,8333	instrument
rs256904	5	55810305	A	T	0,0005	87,0667	0,0345	0,0037	1,1E-20	-0,0035	0,0089	0,6969	instrument
rs264762	5	64384386	A	C	0,0003	53,4648	-0,0250	0,0034	2,6E-13	0,0250	0,0078	0,0014	outlier
rs2712169	2	217671349	G	A	0,0002	44,7289	0,0221	0,0033	2,3E-11	0,0141	0,0076	0,0626	instrument
rs2722285	7	37994955	T	C	0,0002	32,0466	-0,0286	0,0051	1,5E-08	0,0126	0,0121	0,2982	instrument
rs2731340	3	13559829	C	T	0,0002	47,4440	0,0370	0,0054	5,7E-12	0,0074	0,0127	0,5612	instrument
rs273945	7	137611566	A	C	0,0002	38,3157	-0,0202	0,0033	6,0E-10	-0,0072	0,0075	0,3345	instrument
rs2763265	6	168814600	G	A	0,0002	39,0371	-0,0239	0,0038	4,2E-10	0,0134	0,0086	0,1174	instrument
rs2797963	6	34185749	C	T	0,0005	88,1148	0,0556	0,0059	6,2E-21	-0,0155	0,0141	0,2717	instrument
rs2811462	3	129303436	G	T	0,0003	53,0561	0,0282	0,0039	3,2E-13	0,0305	0,0092	0,0009	outlier
rs2820443	1	219753509	C	T	0,0006	109,8293	0,0369	0,0035	1,0E-25	-0,0250	0,0083	0,0026	outlier
rs2823989	21	18080943	G	A	0,0002	32,8326	-0,0197	0,0034	1,0E-08	0,0037	0,0078	0,6412	instrument
rs284660	19	41932084	G	T	0,0003	49,9278	0,0233	0,0033	1,6E-12	0,0095	0,0080	0,2355	instrument
rs28539246	17	29226630	A	T	0,0003	65,3180	-0,0268	0,0033	6,4E-16	-0,0325	0,0076	1,9E-05	outlier
rs2911450	16	88987497	A	C	0,0002	40,2967	-0,0216	0,0034	2,2E-10	0,0096	0,0085	0,2593	instrument
rs3020421	6	152348122	A	G	0,0003	47,8444	0,0240	0,0035	4,6E-12	0,0128	0,0078	0,1036	instrument
rs34134267	7	20417034	A	C	0,0005	86,0247	-0,0304	0,0033	1,8E-20	-0,0079	0,0074	0,2879	instrument
rs34517439	1	78450517	A	C	0,0002	30,6138	0,0276	0,0050	3,1E-08	0,0157	0,0126	0,2146	instrument
rs34831515	19	17275777	T	C	0,0002	38,8748	-0,0239	0,0038	4,5E-10	0,0017	0,0087	0,8417	instrument
rs34919557	20	32510282	T	C	0,0002	31,5616	-0,0315	0,0056	1,9E-08	0,0168	0,0126	0,1819	instrument
rs35307904	9	78511889	A	G	0,0007	137,8261	-0,0584	0,0050	8,0E-32	0,0108	0,0125	0,3857	instrument

rs35699973	18	20726731	G	A	0,0006	113,8477	-0,0419	0,0039	1,5E-26	0,0142	0,0092	0,1236	instrument
rs35874463	15	67457698	G	A	0,0005	91,8329	0,0663	0,0069	9,6E-22	0,0819	0,0166	8,6E-07	outlier
rs3738814	1	17331676	G	A	0,0005	102,4133	-0,0329	0,0033	4,4E-24	-0,0130	0,0074	0,0803	instrument
rs3791679	2	56096892	G	A	0,0012	235,3131	-0,0591	0,0038	4,4E-53	-0,0023	0,0087	0,7921	instrument
rs3794782	17	18127914	C	G	0,0002	35,3427	0,0198	0,0033	2,8E-09	-0,0097	0,0076	0,2017	instrument
rs3800162	6	2134391	A	T	0,0003	53,6990	-0,0241	0,0033	2,3E-13	-0,0132	0,0080	0,0990	instrument
rs3898068	12	28610017	T	C	0,0005	92,2551	-0,0331	0,0035	7,7E-22				
rs395523	6	105396187	G	C	0,0006	123,6531	0,0384	0,0035	1,1E-28	0,0017	0,0079	0,8266	instrument
rs39875	16	2268737	G	A	0,0002	32,2620	0,0214	0,0038	1,4E-08	-0,0179	0,0086	0,0365	instrument
rs4073717	5	170864021	T	G	0,0002	35,3308	-0,0240	0,0040	2,8E-09	-0,0009	0,0095	0,9217	instrument
rs41284816	13	50655989	T	G	0,0003	52,9251	0,0890	0,0122	3,5E-13	0,0397	0,0243	0,1029	instrument
rs4321401	2	69348216	T	C	0,0003	49,0975	-0,0227	0,0032	2,4E-12	-0,0012	0,0081	0,8871	instrument
rs4383453	3	123068359	A	G	0,0002	30,7800	0,0229	0,0041	2,9E-08	0,0043	0,0101	0,6706	instrument
rs4421120	5	32763118	A	G	0,0004	78,0034	0,0387	0,0044	1,0E-18	-0,0062	0,0108	0,5671	instrument
rs4432932	5	115039414	G	A	0,0002	39,5888	0,0230	0,0037	3,1E-10	-0,0062	0,0082	0,4531	instrument
rs4513485	3	49139966	G	A	0,0003	60,8238	-0,0266	0,0034	6,3E-15	0,0030	0,0079	0,7082	instrument
rs4626892	1	221234485	T	C	0,0002	44,7824	0,0218	0,0033	2,2E-11	0,0091	0,0078	0,2422	instrument
rs4699384	4	100570681	A	T	0,0002	32,4783	0,0234	0,0041	1,2E-08	-0,0078	0,0096	0,4126	instrument
rs4731420	7	127863295	C	G	0,0002	30,8910	0,0234	0,0042	2,7E-08	-0,0058	0,0102	0,5665	instrument
rs475390	1	41570459	G	A	0,0002	32,3416	0,0221	0,0039	1,3E-08	-0,0065	0,0088	0,4616	instrument
rs4777230	15	70365762	C	T	0,0002	35,9636	0,0274	0,0046	2,0E-09	0,0127	0,0109	0,2448	instrument
rs4793987	17	46855837	C	T	0,0002	36,5175	-0,0202	0,0033	1,5E-09	-0,0067	0,0076	0,3761	instrument
rs481237	9	98316094	A	G	0,0003	59,7059	-0,0470	0,0061	1,1E-14	-0,0159	0,0157	0,3102	instrument
rs4842133	9	139127566	A	C	0,0005	93,9145	0,0348	0,0036	3,3E-22	0,0187	0,0086	0,0304	instrument
rs4848645	2	121623691	G	C	0,0002	30,7467	-0,0240	0,0043	3,0E-08	0,0056	0,0110	0,6094	instrument
rs4867721	5	172983457	G	T	0,0002	38,5637	0,0205	0,0033	5,3E-10	0,0039	0,0076	0,6073	instrument
rs4901541	14	55235282	C	T	0,0002	39,4757	-0,0208	0,0033	3,3E-10	-0,0007	0,0084	0,9357	instrument
rs4923575	11	28876429	C	T	0,0002	40,3729	-0,0207	0,0033	2,1E-10	0,0111	0,0075	0,1377	instrument
rs4936099	11	130280725	C	A	0,0002	42,7188	-0,0216	0,0033	6,3E-11	-0,0041	0,0084	0,6210	instrument
rs4961091	8	89357874	T	A	0,0002	36,3243	0,0213	0,0035	1,7E-09	-0,0038	0,0080	0,6313	instrument
rs496298	10	131355130	A	G	0,0003	58,6137	-0,0261	0,0034	1,9E-14	0,0003	0,0079	0,9689	instrument

rs537124	7	28203142	C	T	0,0006	114,4888	0,0378	0,0035	9,9E-27	-0,0201	0,0082	0,0135	outlier
rs550373	6	116754150	T	G	0,0002	41,3702	0,0212	0,0033	1,3E-10	-0,0040	0,0075	0,5975	instrument
rs56186137	16	28825953	G	A	0,0003	55,5914	0,0245	0,0033	8,9E-14	0,0091	0,0076	0,2308	instrument
rs57674902	20	4096741	T	C	0,0003	50,9791	0,0243	0,0034	9,3E-13	0,0091	0,0085	0,2863	instrument
rs584961	11	75277628	A	G	0,0003	55,6063	0,0379	0,0051	8,9E-14	0,0377	0,0129	0,0034	outlier
rs58697961	17	1665424	A	G	0,0002	37,5520	0,0209	0,0034	8,9E-10	-0,0077	0,0081	0,3444	instrument
rs60843830	2	286756	G	C	0,0002	33,7325	0,0198	0,0034	6,3E-09	0,0174	0,0079	0,0269	instrument
rs61732778	3	187443314	A	G	0,0002	32,6837	0,0358	0,0063	1,1E-08	-0,0149	0,0152	0,3268	instrument
rs61734651	20	61451332	T	C	0,0003	51,0362	0,0488	0,0068	9,1E-13	-0,0256	0,0175	0,1435	instrument
rs62055545	17	43964561	T	C	0,0004	78,6761	-0,0345	0,0039	7,4E-19	-0,0440	0,0091	1,4E-06	outlier
rs62076439	17	47404628	T	G	0,0004	73,1701	-0,0287	0,0034	1,2E-17	0,0106	0,0077	0,1679	instrument
rs62271373	3	150066540	A	T	0,0002	38,9247	-0,0441	0,0071	4,4E-10	-0,0130	0,0196	0,5088	instrument
rs62330027	5	318557	A	T	0,0002	29,9863	0,0298	0,0054	4,4E-08	0,0162	0,0130	0,2116	instrument
rs62515452	8	57187356	T	C	0,0002	44,5285	0,0305	0,0046	2,5E-11	0,0117	0,0117	0,3193	instrument
rs62621197	19	8670147	T	C	0,0011	211,1187	-0,1347	0,0093	8,2E-48	-0,0424	0,0260	0,1033	instrument
rs6439629	3	135901270	A	G	0,0002	45,9408	-0,0247	0,0036	1,2E-11	0,0116	0,0083	0,1653	instrument
rs6486059	11	12824581	A	G	0,0003	49,8290	0,0232	0,0033	1,7E-12	0,0037	0,0077	0,6354	instrument
rs6492538	13	91993746	A	C	0,0002	46,7441	0,0268	0,0039	8,1E-12	0,0243	0,0088	0,0058	instrument
rs6557679	8	23383743	T	G	0,0002	35,3070	0,0221	0,0037	2,8E-09	0,0055	0,0083	0,5088	instrument
rs6585827	10	124165615	A	G	0,0002	38,1920	0,0199	0,0032	6,4E-10	-0,0058	0,0076	0,4503	instrument
rs6601503	8	10540767	C	T	0,0002	38,8250	0,0230	0,0037	4,6E-10	0,0154	0,0094	0,1031	instrument
rs6739701	2	105132332	G	A	0,0004	66,6829	0,0267	0,0033	3,2E-16	0,0023	0,0085	0,7874	instrument
rs6777217	3	36979042	A	G	0,0002	33,0047	0,0187	0,0032	9,2E-09	0,0097	0,0075	0,1951	instrument
rs6785012	3	141109348	T	C	0,0015	291,7233	0,0555	0,0032	2,4E-65	0,0546	0,0074	2,1E-13	outlier
rs68049170	10	72432047	A	G	0,0003	57,7746	0,0277	0,0036	2,9E-14	0,0079	0,0083	0,3413	instrument
rs6976501	7	50760220	G	A	0,0003	57,6227	0,0294	0,0039	3,2E-14	-0,0002	0,0094	0,9822	instrument
rs6978655	7	46171683	T	C	0,0002	39,0246	0,0228	0,0036	4,2E-10	-0,0016	0,0088	0,8577	instrument
rs6990354	8	145282578	C	T	0,0002	40,3856	-0,0206	0,0032	2,1E-10	0,0035	0,0084	0,6744	instrument
rs702605	5	53167028	T	C	0,0002	33,7441	0,0188	0,0032	6,3E-09	-0,0187	0,0074	0,0112	outlier
rs7101582	11	47714610	A	G	0,0002	30,9133	0,0224	0,0040	2,7E-08	-0,0065	0,0093	0,4876	instrument
rs711245	2	36768875	A	G	0,0002	46,4574	-0,0235	0,0034	9,4E-12	-0,0084	0,0080	0,2937	instrument

rs7155279	14	92485881	T	G	0,0003	63,3291	-0,0267	0,0034	1,8E-15	0,0063	0,0077	0,4109	instrument
rs7170686	15	48821350	A	G	0,0003	56,0396	-0,0414	0,0055	7,1E-14	0,0241	0,0140	0,0849	instrument
rs7184427	16	88804734	A	G	0,0002	30,0301	-0,0248	0,0045	4,3E-08	-0,0182	0,0108	0,0927	instrument
rs7223156	17	68679731	T	C	0,0002	34,1637	-0,0206	0,0035	5,1E-09	-0,0093	0,0080	0,2453	instrument
rs72502575	6	31356087	T	C	0,0003	49,9561	-0,0319	0,0045	1,6E-12	-0,0085	0,0097	0,3813	instrument
rs72708236	1	155980320	G	T	0,0003	66,4707	-0,0773	0,0095	3,6E-16	-0,0336	0,0242	0,1660	instrument
rs72755233	15	100692953	A	G	0,0012	238,3911	-0,0784	0,0051	9,8E-54	0,0136	0,0152	0,3710	instrument
rs72940413	11	69202148	T	C	0,0002	32,3530	-0,0320	0,0056	1,3E-08	-0,0201	0,0124	0,1055	instrument
rs73111557	3	61523251	T	C	0,0002	36,7596	-0,0325	0,0054	1,3E-09	0,0219	0,0132	0,0983	instrument
rs73407643	6	35169123	C	T	0,0004	68,0618	-0,1022	0,0124	1,6E-16	-0,0324	0,0293	0,2682	instrument
rs74469561	3	70929100	T	G	0,0002	39,7904	-0,0383	0,0061	2,8E-10	0,0055	0,0146	0,7084	instrument
rs74829909	4	12964932	C	T	0,0002	31,9448	-0,0305	0,0054	1,6E-08	-0,0101	0,0133	0,4486	instrument
rs75223147	6	19831020	G	T	0,0004	81,5942	0,1015	0,0112	1,7E-19	-0,0247	0,0417	0,5539	instrument
rs7534091	1	118864616	G	A	0,0003	54,6115	-0,0271	0,0037	1,5E-13	-0,0160	0,0086	0,0635	instrument
rs7536227	1	93262390	C	T	0,0002	31,2478	-0,0300	0,0054	2,3E-08	-0,0142	0,0123	0,2480	instrument
rs7540754	1	227850588	A	T	0,0004	76,0899	-0,0374	0,0043	2,7E-18	-0,0037	0,0101	0,7139	instrument
rs7674894	4	120680189	G	A	0,0002	37,3806	-0,0212	0,0035	9,7E-10	0,0028	0,0080	0,7306	instrument
rs7689420	4	145568352	T	C	0,0011	211,4094	-0,0625	0,0043	7,1E-48	0,0010	0,0099	0,9228	instrument
rs7718768	5	178536089	A	G	0,0004	79,9585	0,0305	0,0034	3,9E-19	0,0046	0,0081	0,5657	instrument
rs7736102	5	131604257	G	A	0,0003	66,0149	-0,0264	0,0032	4,5E-16	-0,0257	0,0074	0,0005	outlier
rs7757703	6	47578705	G	A	0,0002	44,3685	-0,0235	0,0035	2,7E-11	-0,0017	0,0080	0,8369	instrument
rs7804293	7	92287849	G	T	0,0004	78,9069	0,0325	0,0037	6,6E-19	0,0199	0,0082	0,0153	instrument
rs781659	4	57779851	G	A	0,0002	33,5817	-0,0189	0,0033	6,8E-09	-0,0143	0,0075	0,0573	instrument
rs78198962	2	233094868	T	C	0,0003	51,3797	0,0655	0,0091	7,6E-13	0,0332	0,0285	0,2454	instrument
rs7846627	8	130736263	G	A	0,0003	58,7669	-0,0249	0,0032	1,8E-14	-0,0003	0,0074	0,9727	instrument
rs7874161	9	109639062	T	C	0,0002	31,3373	0,0189	0,0034	2,2E-08	0,0153	0,0080	0,0563	instrument
rs79112217	1	218622949	C	T	0,0003	61,3239	0,0412	0,0053	4,9E-15	0,0154	0,0125	0,2168	instrument
rs79312203	20	32082962	A	G	0,0002	37,6010	0,0329	0,0054	8,7E-10	-0,0228	0,0125	0,0684	instrument
rs7952436	11	67024534	T	C	0,0009	172,6578	-0,0764	0,0058	1,9E-39	-0,0073	0,0171	0,6692	instrument
rs798488	7	2802522	C	T	0,0010	187,9621	-0,0482	0,0035	9,7E-43	0,0132	0,0081	0,1021	instrument
rs7994151	13	115038879	G	A	0,0002	36,3846	-0,0271	0,0045	1,6E-09	0,0098	0,0104	0,3443	instrument

rs8003703	14	33411639	A	T	0,0002	31,3149	-0,0637	0,0114	2,2E-08	0,0022	0,0303	0,9424	instrument
rs80085026	7	148636421	G	C	0,0004	77,0525	0,0364	0,0041	1,7E-18	0,0311	0,0100	0,0018	outlier
rs8103728	19	33900257	C	G	0,0004	70,8620	-0,0289	0,0034	3,8E-17	0,0264	0,0080	0,0010	outlier
rs8105888	19	19704909	T	A	0,0003	64,1915	0,0324	0,0040	1,1E-15	0,0158	0,0093	0,0903	instrument
rs862041	14	74986403	T	C	0,0004	72,1473	-0,0286	0,0034	2,0E-17	-0,0017	0,0079	0,8275	instrument
rs9262136	6	30619988	A	C	0,0002	32,8211	-0,0264	0,0046	1,0E-08	0,0039	0,0103	0,7062	instrument
rs927415	6	7698384	T	C	0,0002	33,7325	-0,0194	0,0033	6,3E-09	-0,0035	0,0076	0,6427	instrument
rs9285863	5	108071655	C	T	0,0003	51,5662	0,0246	0,0034	7,0E-13	0,0090	0,0078	0,2493	instrument
rs9321266	6	131316458	G	A	0,0003	56,3995	0,0249	0,0033	5,9E-14	-0,0031	0,0076	0,6868	instrument
rs9344005	6	81173371	G	C	0,0002	40,1698	0,0318	0,0050	2,3E-10	-0,0299	0,0119	0,0120	outlier
rs9344115	6	81834237	A	G	0,0002	32,3303	-0,0208	0,0037	1,3E-08	0,0060	0,0084	0,4742	instrument
rs9358912	6	26211146	T	G	0,0012	231,9505	-0,0553	0,0036	2,4E-52	0,0189	0,0081	0,0191	outlier
rs9442571	1	9349611	A	T	0,0003	66,2915	0,0396	0,0049	3,9E-16	-0,0161	0,0123	0,1920	instrument
rs979012	20	6623374	T	C	0,0004	78,9957	0,0297	0,0033	6,3E-19	0,0133	0,0079	0,0929	instrument
rs994014	4	82165790	C	T	0,0006	118,8088	0,0381	0,0035	1,2E-27	-0,0099	0,0081	0,2241	instrument

Table S16. Single Nucleotide Polymorphisms (SNPs) used as instruments in the Mendelian randomization analyses for the effect of TFR on clear cell ovarian cancer based on the genome-wide significance threshold P = 5E-08. R² represents the explained variance in the exposure. SNP-specific instrument strength is given by the respective F-statistic. Some of the genetic variants were identified as outliers in the iterative approach and therefore excluded from the analyses.

SNP	CHR	POS	EA	OA	R^2	F-stat	β_{TFR}	SE_{TFR}	P_{TFR}	$\beta_{Clear\ cell}$	$SE_{Clear\ cell}$	$P_{Clear\ cell}$	$variant_{Clear\ cell}$
rs1002436	1	89146852	A	G	0,0003	48,0799	-0,0225	0,0032	4,1E-12	-0,0513	0,0406	0,2066	instrument
rs10059884	5	32832474	C	A	0,0002	45,7106	0,0222	0,0033	1,4E-11	0,0361	0,0418	0,3883	instrument
rs10152591	15	70048157	C	A	0,0003	52,0701	-0,0379	0,0053	5,4E-13	-0,0690	0,0693	0,3201	instrument
rs10179126	2	165511794	G	C	0,0002	41,1261	0,0215	0,0034	1,4E-10	-0,0192	0,0413	0,6428	instrument
rs1020048	9	86666993	A	C	0,0003	54,2721	0,0308	0,0042	1,8E-13	0,0220	0,0505	0,6634	instrument
rs1026029	2	177065248	C	T	0,0002	32,7295	-0,0409	0,0071	1,1E-08	-0,1652	0,1003	0,0924	instrument
rs10760110	9	123412563	C	T	0,0002	33,6745	0,0204	0,0035	6,5E-09	-0,0177	0,0444	0,6900	instrument
rs10761129	9	94486321	C	T	0,0002	41,9640	0,0222	0,0034	9,3E-11	-0,0751	0,0443	0,0904	instrument
rs10823149	10	69935396	G	T	0,0002	38,5513	-0,0202	0,0032	5,3E-10	-0,0090	0,0402	0,8225	instrument
rs10874671	1	103456389	G	C	0,0003	53,4209	0,0257	0,0035	2,7E-13	0,0417	0,0432	0,3350	instrument
rs10901171	9	133427194	C	T	0,0003	52,6200	-0,0242	0,0033	4,1E-13	0,0735	0,0421	0,0804	instrument

rs10933385	2	232916068	A	G	0,0003	57,3043	-0,0910	0,0120	3,7E-14	-0,2377	0,1543	0,1236	instrument
rs11031005	11	30226356	C	T	0,0002	30,8244	0,0256	0,0046	2,8E-08	-0,0719	0,0587	0,2210	instrument
rs11130321	3	52762698	T	C	0,0003	60,2015	-0,0255	0,0033	8,6E-15	-0,0535	0,0408	0,1899	instrument
rs11186507	10	92983831	G	A	0,0002	40,4873	0,0257	0,0040	2,0E-10	-0,0280	0,0516	0,5876	instrument
rs111947781	3	38021115	T	C	0,0002	42,4187	0,0290	0,0045	7,4E-11	0,0581	0,0554	0,2946	instrument
rs11205303	1	149906413	C	T	0,0020	392,0359	0,0648	0,0033	3,6E-87	0,0691	0,0408	0,0902	instrument
rs11259936	15	84580582	A	C	0,0017	324,7170	-0,0581	0,0032	1,6E-72	0,0173	0,0400	0,6648	instrument
rs112635299	14	94838142	T	G	0,0005	93,4886	0,1081	0,0112	4,1E-22	0,0373	0,1471	0,7998	instrument
rs113059026	10	32158478	A	C	0,0002	38,8499	-0,0223	0,0036	4,6E-10	-0,0967	0,0445	0,0297	instrument
rs115912456	5	82815158	G	A	0,0005	86,3032	-0,0753	0,0081	1,6E-20	0,0545	0,1033	0,5977	instrument
rs11689727	2	25458100	A	C	0,0006	106,5013	-0,0354	0,0034	6,0E-25	-0,1037	0,0441	0,0186	instrument
rs11717301	3	72397284	A	C	0,0003	58,9818	-0,0289	0,0038	1,6E-14	-0,0342	0,0471	0,4676	instrument
rs12129970	1	54877581	G	A	0,0002	34,2690	-0,0307	0,0052	4,8E-09	-0,0695	0,0711	0,3281	instrument
rs12172195	22	45813687	A	G	0,0003	50,8649	0,0324	0,0045	9,9E-13	0,0500	0,0563	0,3750	instrument
rs12409899	1	172227011	G	C	0,0004	85,0260	0,0363	0,0039	3,0E-20	0,0312	0,0488	0,5233	instrument
rs12507561	4	73464895	T	G	0,0007	127,2371	0,0365	0,0032	1,8E-29	0,0591	0,0409	0,1487	instrument
rs12685003	9	17022667	G	A	0,0002	34,9514	-0,0304	0,0051	3,4E-09	0,0026	0,0668	0,9686	instrument
rs12756815	1	113207243	A	C	0,0004	76,3518	-0,0349	0,0040	2,4E-18	-0,0029	0,0495	0,9529	instrument
rs13107395	4	8601846	C	T	0,0003	62,2988	-0,0258	0,0033	3,0E-15	-0,0564	0,0420	0,1793	instrument
rs13128180	4	56286188	T	G	0,0003	51,6668	0,0253	0,0035	6,6E-13	-0,0321	0,0435	0,4600	instrument
rs1313237	14	60848224	C	A	0,0002	46,4710	0,0226	0,0033	9,3E-12	-0,0459	0,0414	0,2679	instrument
rs13144767	4	17974219	G	A	0,0004	68,3922	-0,0395	0,0048	1,3E-16	0,0486	0,0581	0,4035	instrument
rs13170063	5	157895013	G	A	0,0002	45,5485	-0,0227	0,0034	1,5E-11	-0,0624	0,0411	0,1295	instrument
rs13306455	19	7184721	T	C	0,0002	37,7115	0,0264	0,0043	8,2E-10	0,0601	0,0524	0,2518	instrument
rs13429301	2	242061508	C	A	0,0002	32,8326	0,0330	0,0058	1,0E-08	0,1271	0,0693	0,0667	instrument
rs138809270	2	219929394	T	C	0,0003	52,6925	-0,0952	0,0131	3,9E-13	-0,0737	0,1582	0,6411	instrument
rs1388900	16	73800629	T	C	0,0002	33,4774	-0,0208	0,0036	7,2E-09	-0,0602	0,0453	0,1837	instrument
rs1421896	5	141016288	T	G	0,0002	30,5032	0,0183	0,0033	3,3E-08	0,0475	0,0416	0,2531	instrument
rs142599545	6	19844384	G	A	0,0002	37,4540	0,0812	0,0133	9,4E-10	-0,0016	0,2048	0,9939	instrument
rs143384	20	34025756	G	A	0,0011	215,2067	0,0482	0,0033	1,1E-48	-0,0305	0,0409	0,4556	instrument
rs143840904	11	2813322	T	C	0,0003	60,4811	-0,0978	0,0126	7,4E-15	0,0848	0,1937	0,6616	instrument

rs1452815	8	78123634	G	A	0,0002	35,6764	0,0213	0,0036	2,3E-09	0,0722	0,0438	0,0989	instrument
rs1513304	3	99269750	C	T	0,0002	44,5418	0,0236	0,0035	2,5E-11	0,0025	0,0464	0,9570	instrument
rs1528589	5	88748768	C	T	0,0002	43,3617	0,0222	0,0034	4,6E-11	-0,0436	0,0420	0,3000	instrument
rs1550436	15	74221157	T	C	0,0006	111,7237	-0,0343	0,0032	4,0E-26	0,0171	0,0400	0,6682	instrument
rs16942324	15	89383854	A	C	0,0005	104,6518	-0,1025	0,0100	1,4E-24	0,0555	0,1307	0,6712	instrument
rs16953461	17	46261736	G	T	0,0003	62,7574	-0,0312	0,0039	2,3E-15	0,0019	0,0501	0,9705	instrument
rs16996444	19	9997238	C	T	0,0002	47,0866	-0,0245	0,0036	6,8E-12	-0,0690	0,0457	0,1316	instrument
rs17194431	8	120612627	T	C	0,0002	29,9206	0,0196	0,0036	4,5E-08	0,0297	0,0447	0,5070	instrument
rs1749824	10	80923862	A	C	0,0002	32,5011	-0,0185	0,0032	1,2E-08	0,0320	0,0404	0,4283	instrument
rs17511102	2	37960613	T	A	0,0002	34,6446	0,0331	0,0056	4,0E-09	-0,0591	0,1021	0,5606	instrument
rs17654949	2	219890955	T	C	0,0003	51,3940	0,0617	0,0086	7,6E-13	0,0135	0,1089	0,9014	instrument
rs1805007	16	89986117	T	C	0,0003	56,8359	-0,0395	0,0052	4,7E-14	0,0756	0,0736	0,3038	instrument
rs194059	5	56197416	G	A	0,0003	48,9575	-0,0238	0,0034	2,6E-12	-0,0245	0,0420	0,5597	instrument
rs1952256	1	184035116	G	A	0,0006	106,9145	0,0351	0,0034	4,6E-25	0,0115	0,0421	0,7849	instrument
rs1966265	5	176516631	A	G	0,0003	51,8251	0,0268	0,0037	6,1E-13	0,0662	0,0463	0,1530	instrument
rs1980030	10	120970027	G	A	0,0003	47,7476	0,0227	0,0033	4,8E-12	0,0610	0,0413	0,1397	instrument
rs2006122	17	61987405	T	A	0,0003	47,1827	0,0252	0,0037	6,5E-12	0,0545	0,0453	0,2293	instrument
rs200889152	6	117507982	C	A	0,0002	42,3146	-0,0218	0,0034	7,8E-11	-0,0067	0,0416	0,8730	instrument
rs2035901	4	145521867	G	A	0,0002	30,3267	0,0179	0,0032	3,7E-08	0,0799	0,0409	0,0505	instrument
rs2050157	6	142658162	A	G	0,0008	146,8929	-0,0434	0,0036	8,4E-34	0,0058	0,0446	0,8963	instrument
rs2076112	22	38538822	T	C	0,0003	64,3999	-0,0261	0,0032	1,0E-15	0,0736	0,0399	0,0649	instrument
rs2194411	3	185548663	A	G	0,0002	42,9020	0,0324	0,0049	5,8E-11	0,0274	0,0623	0,6605	instrument
rs239458	8	37213496	G	A	0,0002	29,8441	-0,0315	0,0058	4,7E-08	0,0599	0,0704	0,3944	instrument
rs256904	5	55810305	A	T	0,0005	87,0667	0,0345	0,0037	1,1E-20	-0,0619	0,0471	0,1891	instrument
rs264762	5	64384386	A	C	0,0003	53,4648	-0,0250	0,0034	2,6E-13	-0,1148	0,0431	0,0077	instrument
rs2712169	2	217671349	G	A	0,0002	44,7289	0,0221	0,0033	2,3E-11	0,0395	0,0405	0,3301	instrument
rs2722285	7	37994955	T	C	0,0002	32,0466	-0,0286	0,0051	1,5E-08	-0,0776	0,0662	0,2414	instrument
rs2731340	3	13559829	C	T	0,0002	47,4440	0,0370	0,0054	5,7E-12	-0,0758	0,0683	0,2665	instrument
rs273945	7	137611566	A	C	0,0002	38,3157	-0,0202	0,0033	6,0E-10	-0,0632	0,0405	0,1190	instrument
rs2763265	6	168814600	G	A	0,0002	39,0371	-0,0239	0,0038	4,2E-10	-0,0382	0,0472	0,4179	instrument
rs2797963	6	34185749	C	T	0,0005	88,1148	0,0556	0,0059	6,2E-21	0,0863	0,0740	0,2438	instrument

rs2811462	3	129303436	G	T	0,0003	53,0561	0,0282	0,0039	3,2E-13	-0,0307	0,0492	0,5334	instrument
rs2820443	1	219753509	C	T	0,0006	109,8293	0,0369	0,0035	1,0E-25	0,0781	0,0440	0,0759	instrument
rs2823989	21	18080943	G	A	0,0002	32,8326	-0,0197	0,0034	1,0E-08	-0,0014	0,0424	0,9744	instrument
rs284660	19	41932084	G	T	0,0003	49,9278	0,0233	0,0033	1,6E-12	-0,0434	0,0416	0,2969	instrument
rs28539246	17	29226630	A	T	0,0003	65,3180	-0,0268	0,0033	6,4E-16	-0,0391	0,0413	0,3437	instrument
rs2911450	16	88987497	A	C	0,0002	40,2967	-0,0216	0,0034	2,2E-10	-0,0450	0,0447	0,3139	instrument
rs3020421	6	152348122	A	G	0,0003	47,8444	0,0240	0,0035	4,6E-12	-0,0742	0,0434	0,0870	instrument
rs34134267	7	20417034	A	C	0,0005	86,0247	-0,0304	0,0033	1,8E-20	-0,0172	0,0406	0,6715	instrument
rs34517439	1	78450517	A	C	0,0002	30,6138	0,0276	0,0050	3,1E-08	-0,0774	0,0654	0,2362	instrument
rs34831515	19	17275777	T	C	0,0002	38,8748	-0,0239	0,0038	4,5E-10	-0,0102	0,0486	0,8343	instrument
rs34919557	20	32510282	T	C	0,0002	31,5616	-0,0315	0,0056	1,9E-08	-0,0101	0,0737	0,8914	instrument
rs35307904	9	78511889	A	G	0,0007	137,8261	-0,0584	0,0050	8,0E-32	0,0279	0,0644	0,6661	instrument
rs35699973	18	20726731	G	A	0,0006	113,8477	-0,0419	0,0039	1,5E-26	0,0026	0,0494	0,9579	instrument
rs35874463	15	67457698	G	A	0,0005	91,8329	0,0663	0,0069	9,6E-22	-0,1731	0,0936	0,0642	instrument
rs3738814	1	17331676	G	A	0,0005	102,4133	-0,0329	0,0033	4,4E-24	-0,0997	0,0405	0,0139	instrument
rs3791679	2	56096892	G	A	0,0012	235,3131	-0,0591	0,0038	4,4E-53	-0,0321	0,0476	0,4992	instrument
rs3794782	17	18127914	C	G	0,0002	35,3427	0,0198	0,0033	2,8E-09	-0,0552	0,0413	0,1811	instrument
rs3800162	6	2134391	A	T	0,0003	53,6990	-0,0241	0,0033	2,3E-13	0,0008	0,0413	0,9844	instrument
rs395523	6	105396187	G	C	0,0006	123,6531	0,0384	0,0035	1,1E-28	0,0256	0,0426	0,5477	instrument
rs39875	16	2268737	G	A	0,0002	32,2620	0,0214	0,0038	1,4E-08	-0,0839	0,0467	0,0724	instrument
rs4073717	5	170864021	T	G	0,0002	35,3308	-0,0240	0,0040	2,8E-09	-0,0432	0,0509	0,3964	instrument
rs41284816	13	50655989	T	G	0,0003	52,9251	0,0890	0,0122	3,5E-13	0,1337	0,1317	0,3100	instrument
rs4321401	2	69348216	T	C	0,0003	49,0975	-0,0227	0,0032	2,4E-12	-0,0159	0,0434	0,7148	instrument
rs4383453	3	123068359	A	G	0,0002	30,7800	0,0229	0,0041	2,9E-08	0,0607	0,0514	0,2375	instrument
rs4421120	5	32763118	A	G	0,0004	78,0034	0,0387	0,0044	1,0E-18	0,0514	0,0550	0,3501	instrument
rs4432932	5	115039414	G	A	0,0002	39,5888	0,0230	0,0037	3,1E-10	0,0127	0,0441	0,7735	instrument
rs4513485	3	49139966	G	A	0,0003	60,8238	-0,0266	0,0034	6,3E-15	-0,0214	0,0425	0,6140	instrument
rs4626892	1	221234485	T	C	0,0002	44,7824	0,0218	0,0033	2,2E-11	-0,0151	0,0406	0,7106	instrument
rs4699384	4	100570681	A	T	0,0002	32,4783	0,0234	0,0041	1,2E-08	-0,0494	0,0519	0,3412	instrument
rs4731420	7	127863295	C	G	0,0002	30,8910	0,0234	0,0042	2,7E-08	0,0998	0,0518	0,0539	instrument
rs475390	1	41570459	G	A	0,0002	32,3416	0,0221	0,0039	1,3E-08	-0,0169	0,0483	0,7259	instrument

rs4777230	15	70365762	C	T	0,0002	35,9636	0,0274	0,0046	2,0E-09	-0,0647	0,0601	0,2821	instrument
rs4793987	17	46855837	C	T	0,0002	36,5175	-0,0202	0,0033	1,5E-09	-0,0424	0,0413	0,3037	instrument
rs481237	9	98316094	A	G	0,0003	59,7059	-0,0470	0,0061	1,1E-14	-0,0179	0,0799	0,8224	instrument
rs4842133	9	139127566	A	C	0,0005	93,9145	0,0348	0,0036	3,3E-22	-0,0093	0,0461	0,8395	instrument
rs4848645	2	121623691	G	C	0,0002	30,7467	-0,0240	0,0043	3,0E-08	0,0640	0,0566	0,2607	instrument
rs4867721	5	172983457	G	T	0,0002	38,5637	0,0205	0,0033	5,3E-10	0,0234	0,0409	0,5683	instrument
rs4901541	14	55235282	C	T	0,0002	39,4757	-0,0208	0,0033	3,3E-10	-0,0561	0,0427	0,1888	instrument
rs4923575	11	28876429	C	T	0,0002	40,3729	-0,0207	0,0033	2,1E-10	-0,1070	0,0404	0,0082	instrument
rs4936099	11	130280725	C	A	0,0002	42,7188	-0,0216	0,0033	6,3E-11	0,0119	0,0427	0,7802	instrument
rs4961091	8	89357874	T	A	0,0002	36,3243	0,0213	0,0035	1,7E-09	0,0337	0,0433	0,4367	instrument
rs496298	10	131355130	A	G	0,0003	58,6137	-0,0261	0,0034	1,9E-14	-0,0897	0,0425	0,0350	instrument
rs537124	7	28203142	C	T	0,0006	114,4888	0,0378	0,0035	9,9E-27	0,0209	0,0441	0,6355	instrument
rs550373	6	116754150	T	G	0,0002	41,3702	0,0212	0,0033	1,3E-10	0,0567	0,0404	0,1602	instrument
rs56186137	16	28825953	G	A	0,0003	55,5914	0,0245	0,0033	8,9E-14	0,0709	0,0411	0,0845	instrument
rs57674902	20	4096741	T	C	0,0003	50,9791	0,0243	0,0034	9,3E-13	-0,0058	0,0435	0,8936	instrument
rs584961	11	75277628	A	G	0,0003	55,6063	0,0379	0,0051	8,9E-14	0,0530	0,0645	0,4113	instrument
rs58697961	17	1665424	A	G	0,0002	37,5520	0,0209	0,0034	8,9E-10	0,0012	0,0436	0,9779	instrument
rs60843830	2	286756	G	C	0,0002	33,7325	0,0198	0,0034	6,3E-09	0,0342	0,0429	0,4257	instrument
rs61732778	3	187443314	A	G	0,0002	32,6837	0,0358	0,0063	1,1E-08	-0,0307	0,0791	0,6975	instrument
rs61734651	20	61451332	T	C	0,0003	51,0362	0,0488	0,0068	9,1E-13	-0,0706	0,0917	0,4413	instrument
rs62055545	17	43964561	T	C	0,0004	78,6761	-0,0345	0,0039	7,4E-19	0,0298	0,0502	0,5541	instrument
rs62076439	17	47404628	T	G	0,0004	73,1701	-0,0287	0,0034	1,2E-17	-0,0432	0,0413	0,2955	instrument
rs62271373	3	150066540	A	T	0,0002	38,9247	-0,0441	0,0071	4,4E-10	-0,0361	0,0932	0,6982	instrument
rs62330027	5	318557	A	T	0,0002	29,9863	0,0298	0,0054	4,4E-08	-0,0414	0,0686	0,5464	instrument
rs62515452	8	57187356	T	C	0,0002	44,5285	0,0305	0,0046	2,5E-11	0,0471	0,0601	0,4326	instrument
rs62621197	19	8670147	T	C	0,0011	211,1187	-0,1347	0,0093	8,2E-48	-0,1894	0,1422	0,1827	instrument
rs6439629	3	135901270	A	G	0,0002	45,9408	-0,0247	0,0036	1,2E-11	0,0414	0,0450	0,3573	instrument
rs6486059	11	12824581	A	G	0,0003	49,8290	0,0232	0,0033	1,7E-12	-0,0491	0,0414	0,2358	instrument
rs6492538	13	91993746	A	C	0,0002	46,7441	0,0268	0,0039	8,1E-12	-0,0610	0,0485	0,2086	instrument
rs6557679	8	23383743	T	G	0,0002	35,3070	0,0221	0,0037	2,8E-09	-0,0366	0,0454	0,4193	instrument
rs6585827	10	124165615	A	G	0,0002	38,1920	0,0199	0,0032	6,4E-10	0,0200	0,0405	0,6220	instrument

rs6601503	8	10540767	C	T	0,0002	38,8250	0,0230	0,0037	4,6E-10	0,0253	0,0504	0,6155	instrument
rs6739701	2	105132332	G	A	0,0004	66,6829	0,0267	0,0033	3,2E-16	-0,0410	0,0422	0,3309	instrument
rs6777217	3	36979042	A	G	0,0002	33,0047	0,0187	0,0032	9,2E-09	0,0114	0,0405	0,7779	instrument
rs6785012	3	141109348	T	C	0,0015	291,7233	0,0555	0,0032	2,4E-65	-0,0052	0,0403	0,8980	instrument
rs68049170	10	72432047	A	G	0,0003	57,7746	0,0277	0,0036	2,9E-14	0,0668	0,0448	0,1362	instrument
rs6976501	7	50760220	G	A	0,0003	57,6227	0,0294	0,0039	3,2E-14	0,0733	0,0485	0,1305	instrument
rs6978655	7	46171683	T	C	0,0002	39,0246	0,0228	0,0036	4,2E-10	0,0178	0,0456	0,6957	instrument
rs6990354	8	145282578	C	T	0,0002	40,3856	-0,0206	0,0032	2,1E-10	-0,0711	0,0417	0,0883	instrument
rs702605	5	53167028	T	C	0,0002	33,7441	0,0188	0,0032	6,3E-09	-0,0743	0,0403	0,0650	instrument
rs7101582	11	47714610	A	G	0,0002	30,9133	0,0224	0,0040	2,7E-08	0,0164	0,0503	0,7441	instrument
rs711245	2	36768875	A	G	0,0002	46,4574	-0,0235	0,0034	9,4E-12	0,0343	0,0429	0,4244	instrument
rs7155279	14	92485881	T	G	0,0003	63,3291	-0,0267	0,0034	1,8E-15	0,0192	0,0419	0,6462	instrument
rs7170686	15	48821350	A	G	0,0003	56,0396	-0,0414	0,0055	7,1E-14	-0,1126	0,0711	0,1133	instrument
rs7184427	16	88804734	A	G	0,0002	30,0301	-0,0248	0,0045	4,3E-08	0,0062	0,0565	0,9119	instrument
rs7223156	17	68679731	T	C	0,0002	34,1637	-0,0206	0,0035	5,1E-09	0,0830	0,0429	0,0530	instrument
rs72502575	6	31356087	T	C	0,0003	49,9561	-0,0319	0,0045	1,6E-12	-0,0879	0,0537	0,1018	instrument
rs72708236	1	155980320	G	T	0,0003	66,4707	-0,0773	0,0095	3,6E-16	0,0492	0,1217	0,6861	instrument
rs72755233	15	100692953	A	G	0,0012	238,3911	-0,0784	0,0051	9,8E-54	-0,0926	0,0791	0,2423	instrument
rs72940413	11	69202148	T	C	0,0002	32,3530	-0,0320	0,0056	1,3E-08	-0,0412	0,0679	0,5437	instrument
rs73111557	3	61523251	T	C	0,0002	36,7596	-0,0325	0,0054	1,3E-09	-0,0197	0,0696	0,7769	instrument
rs73407643	6	35169123	C	T	0,0004	68,0618	-0,1022	0,0124	1,6E-16	0,1468	0,1512	0,3318	instrument
rs74469561	3	70929100	T	G	0,0002	39,7904	-0,0383	0,0061	2,8E-10	-0,0813	0,0775	0,2941	instrument
rs74829909	4	12964932	C	T	0,0002	31,9448	-0,0305	0,0054	1,6E-08	0,0191	0,0689	0,7811	instrument
rs75223147	6	19831020	G	T	0,0004	81,5942	0,1015	0,0112	1,7E-19	0,1598	0,1922	0,4057	instrument
rs7534091	1	118864616	G	A	0,0003	54,6115	-0,0271	0,0037	1,5E-13	-0,0214	0,0462	0,6436	instrument
rs7536227	1	93262390	C	T	0,0002	31,2478	-0,0300	0,0054	2,3E-08	-0,1176	0,0699	0,0923	instrument
rs7540754	1	227850588	A	T	0,0004	76,0899	-0,0374	0,0043	2,7E-18	-0,0245	0,0550	0,6553	instrument
rs7674894	4	120680189	G	A	0,0002	37,3806	-0,0212	0,0035	9,7E-10	-0,0575	0,0434	0,1855	instrument
rs7689420	4	145568352	T	C	0,0011	211,4094	-0,0625	0,0043	7,1E-48	-0,1967	0,0565	0,0005	outlier
rs7718768	5	178536089	A	G	0,0004	79,9585	0,0305	0,0034	3,9E-19	-0,1289	0,0435	0,0031	outlier
rs7736102	5	131604257	G	A	0,0003	66,0149	-0,0264	0,0032	4,5E-16	0,0030	0,0401	0,9400	instrument

rs7757703	6	47578705	G	A	0,0002	44,3685	-0,0235	0,0035	2,7E-11	0,0344	0,0433	0,4272	instrument
rs7804293	7	92287849	G	T	0,0004	78,9069	0,0325	0,0037	6,6E-19	0,0286	0,0449	0,5241	instrument
rs781659	4	57779851	G	A	0,0002	33,5817	-0,0189	0,0033	6,8E-09	0,0183	0,0406	0,6516	instrument
rs78198962	2	233094868	T	C	0,0003	51,3797	0,0655	0,0091	7,6E-13	-0,1269	0,1397	0,3636	instrument
rs7846627	8	130736263	G	A	0,0003	58,7669	-0,0249	0,0032	1,8E-14	0,0629	0,0398	0,1141	instrument
rs7874161	9	109639062	T	C	0,0002	31,3373	0,0189	0,0034	2,2E-08	0,0109	0,0424	0,7966	instrument
rs79112217	1	218622949	C	T	0,0003	61,3239	0,0412	0,0053	4,9E-15	-0,0203	0,0674	0,7637	instrument
rs79312203	20	32082962	A	G	0,0002	37,6010	0,0329	0,0054	8,7E-10	0,0141	0,0668	0,8324	instrument
rs7952436	11	67024534	T	C	0,0009	172,6578	-0,0764	0,0058	1,9E-39	-0,0751	0,0885	0,3962	instrument
rs798488	7	2802522	C	T	0,0010	187,9621	-0,0482	0,0035	9,7E-43	-0,0765	0,0444	0,0852	instrument
rs7994151	13	115038879	G	A	0,0002	36,3846	-0,0271	0,0045	1,6E-09	0,1141	0,0550	0,0380	instrument
rs8003703	14	33411639	A	T	0,0002	31,3149	-0,0637	0,0114	2,2E-08	0,1440	0,1517	0,3426	instrument
rs80085026	7	148636421	G	C	0,0004	77,0525	0,0364	0,0041	1,7E-18	-0,0009	0,0523	0,9869	instrument
rs8103728	19	33900257	C	G	0,0004	70,8620	-0,0289	0,0034	3,8E-17	0,0232	0,0425	0,5857	instrument
rs8105888	19	19704909	T	A	0,0003	64,1915	0,0324	0,0040	1,1E-15	0,0624	0,0497	0,2094	instrument
rs862041	14	74986403	T	C	0,0004	72,1473	-0,0286	0,0034	2,0E-17	0,0322	0,0422	0,4453	instrument
rs9262136	6	30619988	A	C	0,0002	32,8211	-0,0264	0,0046	1,0E-08	-0,0568	0,0556	0,3071	instrument
rs927415	6	7698384	T	C	0,0002	33,7325	-0,0194	0,0033	6,3E-09	-0,0333	0,0409	0,4145	instrument
rs9285863	5	108071655	C	T	0,0003	51,5662	0,0246	0,0034	7,0E-13	0,0094	0,0426	0,8254	instrument
rs9321266	6	131316458	G	A	0,0003	56,3995	0,0249	0,0033	5,9E-14	0,0102	0,0412	0,8054	instrument
rs9344005	6	81173371	G	C	0,0002	40,1698	0,0318	0,0050	2,3E-10	-0,0528	0,0652	0,4180	instrument
rs9344115	6	81834237	A	G	0,0002	32,3303	-0,0208	0,0037	1,3E-08	0,0511	0,0451	0,2571	instrument
rs9358912	6	26211146	T	G	0,0012	231,9505	-0,0553	0,0036	2,4E-52	0,0556	0,0438	0,2041	instrument
rs9442571	1	9349611	A	T	0,0003	66,2915	0,0396	0,0049	3,9E-16	-0,0294	0,0641	0,6465	instrument
rs979012	20	6623374	T	C	0,0004	78,9957	0,0297	0,0033	6,3E-19	-0,0068	0,0421	0,8722	instrument
rs994014	4	82165790	C	T	0,0006	118,8088	0,0381	0,0035	1,2E-27	0,0006	0,0434	0,9896	instrument

Table S17. Single Nucleotide Polymorphisms (SNPs) used as instruments in the Mendelian randomization analyses for the effect of TFR on endometrioid ovarian cancer based on the genome-wide significance threshold P = 5E-08. R^2 represents the explained variance in the exposure. SNP-specific instrument strength is given by the respective F-statistic. Some of the genetic variants were identified as outliers in the iterative approach and therefore excluded from the analyses.

SNP	CHR	POS	EA	OA	R^2	F-stat	β_{TFR}	SE_{TFR}	P_{TFR}	$\beta_{Endometrioid}$	$SE_{Endometrioid}$	$P_{Endometrioid}$	$variant_{Endometrioid}$
rs1002436	1	89146852	A	G	0,0003	48,0799	-0,0225	0,0032	4,1E-12	-0,0196	0,0291	0,4992	instrument
rs10059884	5	32832474	C	A	0,0002	45,7106	0,0222	0,0033	1,4E-11	0,0056	0,0301	0,8518	instrument
rs10152591	15	70048157	C	A	0,0003	52,0701	-0,0379	0,0053	5,4E-13	0,0935	0,0480	0,0514	instrument
rs10179126	2	165511794	G	C	0,0002	41,1261	0,0215	0,0034	1,4E-10	-0,0032	0,0296	0,9136	instrument
rs1020048	9	86666993	A	C	0,0003	54,2721	0,0308	0,0042	1,8E-13	0,0010	0,0362	0,9782	instrument
rs1026029	2	177065248	C	T	0,0002	32,7295	-0,0409	0,0071	1,1E-08	-0,1244	0,0709	0,0752	instrument
rs10760110	9	123412563	C	T	0,0002	33,6745	0,0204	0,0035	6,5E-09	0,0286	0,0317	0,3661	instrument
rs10761129	9	94486321	C	T	0,0002	41,9640	0,0222	0,0034	9,3E-11	0,0797	0,0314	0,0112	instrument
rs10823149	10	69935396	G	T	0,0002	38,5513	-0,0202	0,0032	5,3E-10	0,0087	0,0287	0,7609	instrument
rs10874671	1	103456389	G	C	0,0003	53,4209	0,0257	0,0035	2,7E-13	-0,0296	0,0311	0,3417	instrument
rs10901171	9	133427194	C	T	0,0003	52,6200	-0,0242	0,0033	4,1E-13	-0,0198	0,0305	0,5161	instrument
rs10933385	2	232916068	A	G	0,0003	57,3043	-0,0910	0,0120	3,7E-14	-0,0305	0,1050	0,7713	instrument
rs11031005	11	30226356	C	T	0,0002	30,8244	0,0256	0,0046	2,8E-08	-0,1389	0,0424	0,0011	outlier
rs11130321	3	52762698	T	C	0,0003	60,2015	-0,0255	0,0033	8,6E-15	0,0062	0,0291	0,8305	instrument
rs11186507	10	92983831	G	A	0,0002	40,4873	0,0257	0,0040	2,0E-10	0,0017	0,0367	0,9634	instrument
rs111947781	3	38021115	T	C	0,0002	42,4187	0,0290	0,0045	7,4E-11	0,0268	0,0397	0,4987	instrument
rs11205303	1	149906413	C	T	0,0020	392,0359	0,0648	0,0033	3,6E-87	0,0015	0,0293	0,9587	instrument
rs11259936	15	84580582	A	C	0,0017	324,7170	-0,0581	0,0032	1,6E-72	0,0009	0,0286	0,9754	instrument
rs112635299	14	94838142	T	G	0,0005	93,4886	0,1081	0,0112	4,1E-22	0,0353	0,1070	0,7417	instrument
rs113059026	10	32158478	A	C	0,0002	38,8499	-0,0223	0,0036	4,6E-10	-0,0011	0,0315	0,9718	instrument
rs115912456	5	82815158	G	A	0,0005	86,3032	-0,0753	0,0081	1,6E-20	-0,0909	0,0768	0,2365	instrument
rs11689727	2	25458100	A	C	0,0006	106,5013	-0,0354	0,0034	6,0E-25	-0,0740	0,0316	0,0193	instrument
rs11717301	3	72397284	A	C	0,0003	58,9818	-0,0289	0,0038	1,6E-14	0,0316	0,0335	0,3465	instrument
rs12129970	1	54877581	G	A	0,0002	34,2690	-0,0307	0,0052	4,8E-09	-0,0083	0,0505	0,8700	instrument
rs12172195	22	45813687	A	G	0,0003	50,8649	0,0324	0,0045	9,9E-13	0,0455	0,0402	0,2582	instrument
rs12409899	1	172227011	G	C	0,0004	85,0260	0,0363	0,0039	3,0E-20	0,0589	0,0348	0,0902	instrument
rs12507561	4	73464895	T	G	0,0007	127,2371	0,0365	0,0032	1,8E-29	0,0312	0,0294	0,2888	instrument

rs12685003	9	17022667	G	A	0,0002	34,9514	-0,0304	0,0051	3,4E-09	-0,1096	0,0501	0,0268	instrument
rs12756815	1	113207243	A	C	0,0004	76,3518	-0,0349	0,0040	2,4E-18	-0,0311	0,0355	0,3811	instrument
rs13107395	4	8601846	C	T	0,0003	62,2988	-0,0258	0,0033	3,0E-15	0,0252	0,0300	0,4016	instrument
rs13128180	4	56286188	T	G	0,0003	51,6668	0,0253	0,0035	6,6E-13	0,0492	0,0308	0,1100	instrument
rs1313237	14	60848224	C	A	0,0002	46,4710	0,0226	0,0033	9,3E-12	0,0395	0,0294	0,1796	instrument
rs13144767	4	17974219	G	A	0,0004	68,3922	-0,0395	0,0048	1,3E-16	0,0638	0,0413	0,1225	instrument
rs13170063	5	157895013	G	A	0,0002	45,5485	-0,0227	0,0034	1,5E-11	0,0070	0,0294	0,8125	instrument
rs13306455	19	7184721	T	C	0,0002	37,7115	0,0264	0,0043	8,2E-10	-0,0589	0,0385	0,1257	instrument
rs13429301	2	242061508	C	A	0,0002	32,8326	0,0330	0,0058	1,0E-08	-0,0681	0,0524	0,1937	instrument
rs138809270	2	219929394	T	C	0,0003	52,6925	-0,0952	0,0131	3,9E-13	-0,0243	0,1104	0,8258	instrument
rs1388900	16	73800629	T	C	0,0002	33,4774	-0,0208	0,0036	7,2E-09	-0,0228	0,0324	0,4808	instrument
rs1421896	5	141016288	T	G	0,0002	30,5032	0,0183	0,0033	3,3E-08	0,0266	0,0297	0,3706	instrument
rs142599545	6	19844384	G	A	0,0002	37,4540	0,0812	0,0133	9,4E-10	0,1362	0,1434	0,3422	instrument
rs143384	20	34025756	G	A	0,0011	215,2067	0,0482	0,0033	1,1E-48	0,0006	0,0292	0,9841	instrument
rs143840904	11	2813322	T	C	0,0003	60,4811	-0,0978	0,0126	7,4E-15	-0,0519	0,1402	0,7112	instrument
rs1452815	8	78123634	G	A	0,0002	35,6764	0,0213	0,0036	2,3E-09	-0,0415	0,0318	0,1920	instrument
rs1513304	3	99269750	C	T	0,0002	44,5418	0,0236	0,0035	2,5E-11	0,0238	0,0333	0,4744	instrument
rs1528589	5	88748768	C	T	0,0002	43,3617	0,0222	0,0034	4,6E-11	-0,0361	0,0301	0,2301	instrument
rs1550436	15	74221157	T	C	0,0006	111,7237	-0,0343	0,0032	4,0E-26	0,0102	0,0286	0,7219	instrument
rs16942324	15	89383854	A	C	0,0005	104,6518	-0,1025	0,0100	1,4E-24	-0,0464	0,0972	0,6332	instrument
rs16953461	17	46261736	G	T	0,0003	62,7574	-0,0312	0,0039	2,3E-15	0,0186	0,0358	0,6039	instrument
rs16996444	19	9997238	C	T	0,0002	47,0866	-0,0245	0,0036	6,8E-12	0,0671	0,0322	0,0371	instrument
rs17194431	8	120612627	T	C	0,0002	29,9206	0,0196	0,0036	4,5E-08	0,0307	0,0320	0,3384	instrument
rs1749824	10	80923862	A	C	0,0002	32,5011	-0,0185	0,0032	1,2E-08	0,0402	0,0288	0,1633	instrument
rs17511102	2	37960613	T	A	0,0002	34,6446	0,0331	0,0056	4,0E-09	-0,0101	0,0709	0,8865	instrument
rs17654949	2	219890955	T	C	0,0003	51,3940	0,0617	0,0086	7,6E-13	0,0765	0,0766	0,3185	instrument
rs1805007	16	89986117	T	C	0,0003	56,8359	-0,0395	0,0052	4,7E-14	0,0059	0,0539	0,9133	instrument
rs194059	5	56197416	G	A	0,0003	48,9575	-0,0238	0,0034	2,6E-12	0,0412	0,0299	0,1675	instrument
rs1952256	1	184035116	G	A	0,0006	106,9145	0,0351	0,0034	4,6E-25	-0,0657	0,0303	0,0302	instrument
rs1966265	5	176516631	A	G	0,0003	51,8251	0,0268	0,0037	6,1E-13	0,0602	0,0334	0,0715	instrument
rs1980030	10	120970027	G	A	0,0003	47,7476	0,0227	0,0033	4,8E-12	0,0211	0,0296	0,4766	instrument

rs2006122	17	61987405	T	A	0,0003	47,1827	0,0252	0,0037	6,5E-12	0,0271	0,0325	0,4037	instrument
rs200889152	6	117507982	C	A	0,0002	42,3146	-0,0218	0,0034	7,8E-11	0,0368	0,0297	0,2155	instrument
rs2035901	4	145521867	G	A	0,0002	30,3267	0,0179	0,0032	3,7E-08	0,0001	0,0293	0,9979	instrument
rs2050157	6	142658162	A	G	0,0008	146,8929	-0,0434	0,0036	8,4E-34	0,0264	0,0317	0,4057	instrument
rs2076112	22	38538822	T	C	0,0003	64,3999	-0,0261	0,0032	1,0E-15	0,0093	0,0285	0,7452	instrument
rs2194411	3	185548663	A	G	0,0002	42,9020	0,0324	0,0049	5,8E-11	0,0529	0,0446	0,2362	instrument
rs239458	8	37213496	G	A	0,0002	29,8441	-0,0315	0,0058	4,7E-08	-0,0489	0,0517	0,3435	instrument
rs256904	5	55810305	A	T	0,0005	87,0667	0,0345	0,0037	1,1E-20	0,0232	0,0334	0,4875	instrument
rs264762	5	64384386	A	C	0,0003	53,4648	-0,0250	0,0034	2,6E-13	-0,0162	0,0305	0,5953	instrument
rs2712169	2	217671349	G	A	0,0002	44,7289	0,0221	0,0033	2,3E-11	0,0452	0,0290	0,1194	instrument
rs2722285	7	37994955	T	C	0,0002	32,0466	-0,0286	0,0051	1,5E-08	-0,0327	0,0467	0,4830	instrument
rs2731340	3	13559829	C	T	0,0002	47,4440	0,0370	0,0054	5,7E-12	-0,0463	0,0486	0,3404	instrument
rs273945	7	137611566	A	C	0,0002	38,3157	-0,0202	0,0033	6,0E-10	0,0157	0,0289	0,5878	instrument
rs2763265	6	168814600	G	A	0,0002	39,0371	-0,0239	0,0038	4,2E-10	-0,0364	0,0337	0,2789	instrument
rs2797963	6	34185749	C	T	0,0005	88,1148	0,0556	0,0059	6,2E-21	0,1557	0,0519	0,0027	outlier
rs2811462	3	129303436	G	T	0,0003	53,0561	0,0282	0,0039	3,2E-13	0,0902	0,0346	0,0091	instrument
rs2820443	1	219753509	C	T	0,0006	109,8293	0,0369	0,0035	1,0E-25	-0,0332	0,0321	0,3007	instrument
rs2823989	21	18080943	G	A	0,0002	32,8326	-0,0197	0,0034	1,0E-08	-0,0390	0,0305	0,2003	instrument
rs284660	19	41932084	G	T	0,0003	49,9278	0,0233	0,0033	1,6E-12	0,0191	0,0297	0,5201	instrument
rs28539246	17	29226630	A	T	0,0003	65,3180	-0,0268	0,0033	6,4E-16	-0,0233	0,0294	0,4278	instrument
rs2911450	16	88987497	A	C	0,0002	40,2967	-0,0216	0,0034	2,2E-10	0,0240	0,0318	0,4509	instrument
rs3020421	6	152348122	A	G	0,0003	47,8444	0,0240	0,0035	4,6E-12	0,0504	0,0305	0,0982	instrument
rs34134267	7	20417034	A	C	0,0005	86,0247	-0,0304	0,0033	1,8E-20	-0,0075	0,0289	0,7965	instrument
rs34517439	1	78450517	A	C	0,0002	30,6138	0,0276	0,0050	3,1E-08	-0,0488	0,0471	0,3001	instrument
rs34831515	19	17275777	T	C	0,0002	38,8748	-0,0239	0,0038	4,5E-10	-0,0323	0,0348	0,3518	instrument
rs34919557	20	32510282	T	C	0,0002	31,5616	-0,0315	0,0056	1,9E-08	0,0331	0,0515	0,5216	instrument
rs35307904	9	78511889	A	G	0,0007	137,8261	-0,0584	0,0050	8,0E-32	0,0239	0,0469	0,6111	instrument
rs35699973	18	20726731	G	A	0,0006	113,8477	-0,0419	0,0039	1,5E-26	0,0546	0,0352	0,1202	instrument
rs35874463	15	67457698	G	A	0,0005	91,8329	0,0663	0,0069	9,6E-22	-0,0966	0,0659	0,1425	instrument
rs3738814	1	17331676	G	A	0,0005	102,4133	-0,0329	0,0033	4,4E-24	-0,0481	0,0288	0,0952	instrument
rs3791679	2	56096892	G	A	0,0012	235,3131	-0,0591	0,0038	4,4E-53	-0,0184	0,0336	0,5840	instrument

rs3794782	17	18127914	C	G	0,0002	35,3427	0,0198	0,0033	2,8E-09	0,0270	0,0294	0,3586	instrument
rs3800162	6	2134391	A	T	0,0003	53,6990	-0,0241	0,0033	2,3E-13	-0,0309	0,0298	0,2998	instrument
rs395523	6	105396187	G	C	0,0006	123,6531	0,0384	0,0035	1,1E-28	0,0164	0,0306	0,5915	instrument
rs39875	16	2268737	G	A	0,0002	32,2620	0,0214	0,0038	1,4E-08	0,0559	0,0329	0,0889	instrument
rs4073717	5	170864021	T	G	0,0002	35,3308	-0,0240	0,0040	2,8E-09	-0,0036	0,0361	0,9213	instrument
rs41284816	13	50655989	T	G	0,0003	52,9251	0,0890	0,0122	3,5E-13	0,0135	0,0973	0,8893	instrument
rs4321401	2	69348216	T	C	0,0003	49,0975	-0,0227	0,0032	2,4E-12	-0,0294	0,0311	0,3443	instrument
rs4383453	3	123068359	A	G	0,0002	30,7800	0,0229	0,0041	2,9E-08	0,0494	0,0372	0,1843	instrument
rs4421120	5	32763118	A	G	0,0004	78,0034	0,0387	0,0044	1,0E-18	0,0031	0,0401	0,9375	instrument
rs4432932	5	115039414	G	A	0,0002	39,5888	0,0230	0,0037	3,1E-10	-0,0445	0,0318	0,1616	instrument
rs4513485	3	49139966	G	A	0,0003	60,8238	-0,0266	0,0034	6,3E-15	-0,0375	0,0304	0,2177	instrument
rs4626892	1	221234485	T	C	0,0002	44,7824	0,0218	0,0033	2,2E-11	0,0082	0,0292	0,7780	instrument
rs4699384	4	100570681	A	T	0,0002	32,4783	0,0234	0,0041	1,2E-08	-0,0020	0,0368	0,9575	instrument
rs4731420	7	127863295	C	G	0,0002	30,8910	0,0234	0,0042	2,7E-08	-0,0046	0,0377	0,9030	instrument
rs475390	1	41570459	G	A	0,0002	32,3416	0,0221	0,0039	1,3E-08	-0,0018	0,0344	0,9582	instrument
rs4777230	15	70365762	C	T	0,0002	35,9636	0,0274	0,0046	2,0E-09	-0,0222	0,0428	0,6039	instrument
rs4793987	17	46855837	C	T	0,0002	36,5175	-0,0202	0,0033	1,5E-09	-0,0252	0,0294	0,3912	instrument
rs481237	9	98316094	A	G	0,0003	59,7059	-0,0470	0,0061	1,1E-14	0,0661	0,0564	0,2415	instrument
rs4842133	9	139127566	A	C	0,0005	93,9145	0,0348	0,0036	3,3E-22	0,0072	0,0329	0,8272	instrument
rs4848645	2	121623691	G	C	0,0002	30,7467	-0,0240	0,0043	3,0E-08	-0,0307	0,0422	0,4661	instrument
rs4867721	5	172983457	G	T	0,0002	38,5637	0,0205	0,0033	5,3E-10	-0,0172	0,0294	0,5581	instrument
rs4901541	14	55235282	C	T	0,0002	39,4757	-0,0208	0,0033	3,3E-10	0,0150	0,0306	0,6250	instrument
rs4923575	11	28876429	C	T	0,0002	40,3729	-0,0207	0,0033	2,1E-10	-0,0318	0,0290	0,2722	instrument
rs4936099	11	130280725	C	A	0,0002	42,7188	-0,0216	0,0033	6,3E-11	-0,0398	0,0308	0,1968	instrument
rs4961091	8	89357874	T	A	0,0002	36,3243	0,0213	0,0035	1,7E-09	-0,0187	0,0312	0,5489	instrument
rs496298	10	131355130	A	G	0,0003	58,6137	-0,0261	0,0034	1,9E-14	-0,0089	0,0302	0,7682	instrument
rs537124	7	28203142	C	T	0,0006	114,4888	0,0378	0,0035	9,9E-27	0,0456	0,0315	0,1481	instrument
rs550373	6	116754150	T	G	0,0002	41,3702	0,0212	0,0033	1,3E-10	-0,0020	0,0289	0,9454	instrument
rs56186137	16	28825953	G	A	0,0003	55,5914	0,0245	0,0033	8,9E-14	0,0697	0,0294	0,0179	instrument
rs57674902	20	4096741	T	C	0,0003	50,9791	0,0243	0,0034	9,3E-13	0,0274	0,0312	0,3805	instrument
rs584961	11	75277628	A	G	0,0003	55,6063	0,0379	0,0051	8,9E-14	0,0577	0,0467	0,2158	instrument

rs58697961	17	1665424	A	G	0,0002	37,5520	0,0209	0,0034	8,9E-10	0,0257	0,0311	0,4090	instrument
rs60843830	2	286756	G	C	0,0002	33,7325	0,0198	0,0034	6,3E-09	-0,0003	0,0307	0,9927	instrument
rs61732778	3	187443314	A	G	0,0002	32,6837	0,0358	0,0063	1,1E-08	0,0190	0,0561	0,7347	instrument
rs61734651	20	61451332	T	C	0,0003	51,0362	0,0488	0,0068	9,1E-13	-0,0076	0,0652	0,9073	instrument
rs62055545	17	43964561	T	C	0,0004	78,6761	-0,0345	0,0039	7,4E-19	0,1100	0,0353	0,0020	outlier
rs62076439	17	47404628	T	G	0,0004	73,1701	-0,0287	0,0034	1,2E-17	-0,0226	0,0296	0,4454	instrument
rs62271373	3	150066540	A	T	0,0002	38,9247	-0,0441	0,0071	4,4E-10	0,1071	0,0649	0,0986	instrument
rs62330027	5	318557	A	T	0,0002	29,9863	0,0298	0,0054	4,4E-08	0,0315	0,0482	0,5130	instrument
rs62515452	8	57187356	T	C	0,0002	44,5285	0,0305	0,0046	2,5E-11	-0,0321	0,0437	0,4633	instrument
rs62621197	19	8670147	T	C	0,0011	211,1187	-0,1347	0,0093	8,2E-48	0,0270	0,0964	0,7798	instrument
rs6439629	3	135901270	A	G	0,0002	45,9408	-0,0247	0,0036	1,2E-11	0,0171	0,0322	0,5953	instrument
rs6486059	11	12824581	A	G	0,0003	49,8290	0,0232	0,0033	1,7E-12	-0,0657	0,0296	0,0263	instrument
rs6492538	13	91993746	A	C	0,0002	46,7441	0,0268	0,0039	8,1E-12	0,0458	0,0342	0,1797	instrument
rs6557679	8	23383743	T	G	0,0002	35,3070	0,0221	0,0037	2,8E-09	0,0044	0,0324	0,8910	instrument
rs6585827	10	124165615	A	G	0,0002	38,1920	0,0199	0,0032	6,4E-10	0,0251	0,0290	0,3857	instrument
rs6601503	8	10540767	C	T	0,0002	38,8250	0,0230	0,0037	4,6E-10	0,0522	0,0364	0,1513	instrument
rs6739701	2	105132332	G	A	0,0004	66,6829	0,0267	0,0033	3,2E-16	-0,0124	0,0305	0,6854	instrument
rs6777217	3	36979042	A	G	0,0002	33,0047	0,0187	0,0032	9,2E-09	0,0305	0,0289	0,2916	instrument
rs6785012	3	141109348	T	C	0,0015	291,7233	0,0555	0,0032	2,4E-65	0,0107	0,0288	0,7112	instrument
rs68049170	10	72432047	A	G	0,0003	57,7746	0,0277	0,0036	2,9E-14	0,0553	0,0321	0,0851	instrument
rs6976501	7	50760220	G	A	0,0003	57,6227	0,0294	0,0039	3,2E-14	-3,6E-05	0,0354	0,9992	instrument
rs6978655	7	46171683	T	C	0,0002	39,0246	0,0228	0,0036	4,2E-10	-0,0146	0,0328	0,6574	instrument
rs6990354	8	145282578	C	T	0,0002	40,3856	-0,0206	0,0032	2,1E-10	-0,0010	0,0302	0,9723	instrument
rs702605	5	53167028	T	C	0,0002	33,7441	0,0188	0,0032	6,3E-09	0,0010	0,0287	0,9723	instrument
rs7101582	11	47714610	A	G	0,0002	30,9133	0,0224	0,0040	2,7E-08	0,1001	0,0355	0,0048	outlier
rs711245	2	36768875	A	G	0,0002	46,4574	-0,0235	0,0034	9,4E-12	-0,0018	0,0308	0,9543	instrument
rs7155279	14	92485881	T	G	0,0003	63,3291	-0,0267	0,0034	1,8E-15	0,0648	0,0297	0,0292	instrument
rs7170686	15	48821350	A	G	0,0003	56,0396	-0,0414	0,0055	7,1E-14	0,0567	0,0494	0,2506	instrument
rs7184427	16	88804734	A	G	0,0002	30,0301	-0,0248	0,0045	4,3E-08	-0,0108	0,0407	0,7913	instrument
rs7223156	17	68679731	T	C	0,0002	34,1637	-0,0206	0,0035	5,1E-09	-0,0344	0,0311	0,2678	instrument
rs72502575	6	31356087	T	C	0,0003	49,9561	-0,0319	0,0045	1,6E-12	-0,0378	0,0376	0,3149	instrument

rs72708236	1	155980320	G	T	0,0003	66,4707	-0,0773	0,0095	3,6E-16	-0,0768	0,0905	0,3964	instrument
rs72755233	15	100692953	A	G	0,0012	238,3911	-0,0784	0,0051	9,8E-54	-0,1417	0,0577	0,0140	instrument
rs72940413	11	69202148	T	C	0,0002	32,3530	-0,0320	0,0056	1,3E-08	-0,0595	0,0488	0,2225	instrument
rs73111557	3	61523251	T	C	0,0002	36,7596	-0,0325	0,0054	1,3E-09	0,0867	0,0486	0,0745	instrument
rs73407643	6	35169123	C	T	0,0004	68,0618	-0,1022	0,0124	1,6E-16	-0,0024	0,1124	0,9828	instrument
rs74469561	3	70929100	T	G	0,0002	39,7904	-0,0383	0,0061	2,8E-10	0,0526	0,0539	0,3292	instrument
rs74829909	4	12964932	C	T	0,0002	31,9448	-0,0305	0,0054	1,6E-08	0,0420	0,0492	0,3928	instrument
rs75223147	6	19831020	G	T	0,0004	81,5942	0,1015	0,0112	1,7E-19	-0,2370	0,1510	0,1165	instrument
rs7534091	1	118864616	G	A	0,0003	54,6115	-0,0271	0,0037	1,5E-13	-0,0478	0,0331	0,1483	instrument
rs7536227	1	93262390	C	T	0,0002	31,2478	-0,0300	0,0054	2,3E-08	-0,0511	0,0492	0,2991	instrument
rs7540754	1	227850588	A	T	0,0004	76,0899	-0,0374	0,0043	2,7E-18	-0,0473	0,0396	0,2325	instrument
rs7674894	4	120680189	G	A	0,0002	37,3806	-0,0212	0,0035	9,7E-10	0,0173	0,0309	0,5754	instrument
rs7689420	4	145568352	T	C	0,0011	211,4094	-0,0625	0,0043	7,1E-48	0,0562	0,0381	0,1409	instrument
rs7718768	5	178536089	A	G	0,0004	79,9585	0,0305	0,0034	3,9E-19	-0,0034	0,0307	0,9128	instrument
rs7736102	5	131604257	G	A	0,0003	66,0149	-0,0264	0,0032	4,5E-16	-0,0153	0,0287	0,5950	instrument
rs7757703	6	47578705	G	A	0,0002	44,3685	-0,0235	0,0035	2,7E-11	-0,0189	0,0311	0,5422	instrument
rs7804293	7	92287849	G	T	0,0004	78,9069	0,0325	0,0037	6,6E-19	-0,0130	0,0323	0,6879	instrument
rs781659	4	57779851	G	A	0,0002	33,5817	-0,0189	0,0033	6,8E-09	-0,0003	0,0290	0,9925	instrument
rs78198962	2	233094868	T	C	0,0003	51,3797	0,0655	0,0091	7,6E-13	-0,1154	0,1018	0,2567	instrument
rs7846627	8	130736263	G	A	0,0003	58,7669	-0,0249	0,0032	1,8E-14	0,0497	0,0286	0,0817	instrument
rs7874161	9	109639062	T	C	0,0002	31,3373	0,0189	0,0034	2,2E-08	-0,0086	0,0305	0,7789	instrument
rs79112217	1	218622949	C	T	0,0003	61,3239	0,0412	0,0053	4,9E-15	-0,0581	0,0488	0,2339	instrument
rs79312203	20	32082962	A	G	0,0002	37,6010	0,0329	0,0054	8,7E-10	-0,0172	0,0483	0,7220	instrument
rs7952436	11	67024534	T	C	0,0009	172,6578	-0,0764	0,0058	1,9E-39	-0,0092	0,0629	0,8840	instrument
rs798488	7	2802522	C	T	0,0010	187,9621	-0,0482	0,0035	9,7E-43	0,0025	0,0313	0,9369	instrument
rs7994151	13	115038879	G	A	0,0002	36,3846	-0,0271	0,0045	1,6E-09	-0,0249	0,0404	0,5385	instrument
rs8003703	14	33411639	A	T	0,0002	31,3149	-0,0637	0,0114	2,2E-08	-0,2034	0,1184	0,0859	instrument
rs80085026	7	148636421	G	C	0,0004	77,0525	0,0364	0,0041	1,7E-18	0,0531	0,0370	0,1511	instrument
rs8103728	19	33900257	C	G	0,0004	70,8620	-0,0289	0,0034	3,8E-17	-0,0506	0,0306	0,0982	instrument
rs8105888	19	19704909	T	A	0,0003	64,1915	0,0324	0,0040	1,1E-15	0,0101	0,0360	0,7796	instrument
rs862041	14	74986403	T	C	0,0004	72,1473	-0,0286	0,0034	2,0E-17	-0,0022	0,0302	0,9411	instrument

rs9262136	6	30619988	A	C	0,0002	32,8211	-0,0264	0,0046	1,0E-08	-0,0089	0,0394	0,8208	instrument
rs927415	6	7698384	T	C	0,0002	33,7325	-0,0194	0,0033	6,3E-09	0,0006	0,0292	0,9829	instrument
rs9285863	5	108071655	C	T	0,0003	51,5662	0,0246	0,0034	7,0E-13	0,0255	0,0304	0,4021	instrument
rs9321266	6	131316458	G	A	0,0003	56,3995	0,0249	0,0033	5,9E-14	-0,0096	0,0295	0,7438	instrument
rs9344005	6	81173371	G	C	0,0002	40,1698	0,0318	0,0050	2,3E-10	0,0732	0,0456	0,1085	instrument
rs9344115	6	81834237	A	G	0,0002	32,3303	-0,0208	0,0037	1,3E-08	-0,0264	0,0327	0,4194	instrument
rs9358912	6	26211146	T	G	0,0012	231,9505	-0,0553	0,0036	2,4E-52	0,0317	0,0313	0,3097	instrument
rs9442571	1	9349611	A	T	0,0003	66,2915	0,0396	0,0049	3,9E-16	-0,0117	0,0457	0,7981	instrument
rs979012	20	6623374	T	C	0,0004	78,9957	0,0297	0,0033	6,3E-19	-0,0243	0,0301	0,4194	instrument
rs994014	4	82165790	C	T	0,0006	118,8088	0,0381	0,0035	1,2E-27	-0,0729	0,0313	0,0200	instrument

Table S18. Single Nucleotide Polymorphisms (SNPs) used as instruments in the Mendelian randomization analyses for the effect of TFR on high or low serous ovarian cancer based on the genome-wide significance threshold P = 5E-08. R^2 represents the explained variance in the exposure. SNP-specific instrument strength is given by the respective F-statistic. Some of the genetic variants were identified as outliers in the iterative approach and therefore excluded from the analyses.

SNP	CHR	POS	EA	OA	R^2	F-stat	β_{TFR}	SE_{TFR}	P_{TFR}	$\beta_{High\ low\ serous}$	$S_{High\ low\ serous}$	$P_{High\ low\ serous}$	variant$_{High\ low\ serous}$
rs1002436	1	89146852	A	G	0,0003	48,0799	-0,0225	0,0032	4,1E-12	0,0044	0,0155	0,7764	instrument
rs10059884	5	32832474	C	A	0,0002	45,7106	0,0222	0,0033	1,4E-11	-0,0260	0,0160	0,1033	instrument
rs10152591	15	70048157	C	A	0,0003	52,0701	-0,0379	0,0053	5,4E-13	-0,0171	0,0264	0,5172	instrument
rs10179126	2	165511794	G	C	0,0002	41,1261	0,0215	0,0034	1,4E-10	-0,0264	0,0158	0,0948	instrument
rs1020048	9	86666993	A	C	0,0003	54,2721	0,0308	0,0042	1,8E-13	-0,0150	0,0194	0,4400	instrument
rs1026029	2	177065248	C	T	0,0002	32,7295	-0,0409	0,0071	1,1E-08	-0,0821	0,0368	0,0249	instrument
rs10760110	9	123412563	C	T	0,0002	33,6745	0,0204	0,0035	6,5E-09	0,0154	0,0169	0,3647	instrument
rs10761129	9	94486321	C	T	0,0002	41,9640	0,0222	0,0034	9,3E-11	0,0333	0,0168	0,0465	instrument
rs10823149	10	69935396	G	T	0,0002	38,5513	-0,0202	0,0032	5,3E-10	-0,0024	0,0153	0,8733	instrument
rs10874671	1	103456389	G	C	0,0003	53,4209	0,0257	0,0035	2,7E-13	0,0092	0,0166	0,5795	instrument
rs10901171	9	133427194	C	T	0,0003	52,6200	-0,0242	0,0033	4,1E-13	0,0117	0,0162	0,4693	instrument
rs10933385	2	232916068	A	G	0,0003	57,3043	-0,0910	0,0120	3,7E-14	-0,0683	0,0554	0,2179	instrument
rs11031005	11	30226356	C	T	0,0002	30,8244	0,0256	0,0046	2,8E-08	-0,0239	0,0220	0,2764	instrument
rs11130321	3	52762698	T	C	0,0003	60,2015	-0,0255	0,0033	8,6E-15	0,0230	0,0155	0,1372	instrument
rs11186507	10	92983831	G	A	0,0002	40,4873	0,0257	0,0040	2,0E-10	0,0009	0,0197	0,9649	instrument
rs111947781	3	38021115	T	C	0,0002	42,4187	0,0290	0,0045	7,4E-11	0,0033	0,0214	0,8765	instrument

rs11205303	1	149906413	C	T	0,0020	392,0359	0,0648	0,0033	3,6E-87	-0,0170	0,0156	0,2760	instrument
rs11259936	15	84580582	A	C	0,0017	324,7170	-0,0581	0,0032	1,6E-72	-0,0138	0,0152	0,3622	instrument
rs112635299	14	94838142	T	G	0,0005	93,4886	0,1081	0,0112	4,1E-22	0,0182	0,0574	0,7513	instrument
rs113059026	10	32158478	A	C	0,0002	38,8499	-0,0223	0,0036	4,6E-10	0,0008	0,0168	0,9602	instrument
rs115912456	5	82815158	G	A	0,0005	86,3032	-0,0753	0,0081	1,6E-20	0,0235	0,0393	0,5495	instrument
rs11689727	2	25458100	A	C	0,0006	106,5013	-0,0354	0,0034	6,0E-25	-0,0153	0,0168	0,3619	instrument
rs11717301	3	72397284	A	C	0,0003	58,9818	-0,0289	0,0038	1,6E-14	0,0094	0,0180	0,6022	instrument
rs12129970	1	54877581	G	A	0,0002	34,2690	-0,0307	0,0052	4,8E-09	-0,0171	0,0271	0,5290	instrument
rs12172195	22	45813687	A	G	0,0003	50,8649	0,0324	0,0045	9,9E-13	-0,0113	0,0216	0,6002	instrument
rs12409899	1	172227011	G	C	0,0004	85,0260	0,0363	0,0039	3,0E-20	-0,0032	0,0187	0,8662	instrument
rs12507561	4	73464895	T	G	0,0007	127,2371	0,0365	0,0032	1,8E-29	-0,0038	0,0156	0,8078	instrument
rs12685003	9	17022667	G	A	0,0002	34,9514	-0,0304	0,0051	3,4E-09	-0,1288	0,0267	1,2E-06	outlier
rs12756815	1	113207243	A	C	0,0004	76,3518	-0,0349	0,0040	2,4E-18	-0,0091	0,0189	0,6303	instrument
rs13107395	4	8601846	C	T	0,0003	62,2988	-0,0258	0,0033	3,0E-15	0,0027	0,0160	0,8666	instrument
rs13128180	4	56286188	T	G	0,0003	51,6668	0,0253	0,0035	6,6E-13	0,0367	0,0164	0,0255	instrument
rs1313237	14	60848224	C	A	0,0002	46,4710	0,0226	0,0033	9,3E-12	-0,0194	0,0157	0,2176	instrument
rs13144767	4	17974219	G	A	0,0004	68,3922	-0,0395	0,0048	1,3E-16	0,0168	0,0224	0,4539	instrument
rs13170063	5	157895013	G	A	0,0002	45,5485	-0,0227	0,0034	1,5E-11	-0,0291	0,0157	0,0636	instrument
rs13306455	19	7184721	T	C	0,0002	37,7115	0,0264	0,0043	8,2E-10	-0,0156	0,0204	0,4440	instrument
rs13429301	2	242061508	C	A	0,0002	32,8326	0,0330	0,0058	1,0E-08	-0,0130	0,0275	0,6360	instrument
rs138809270	2	219929394	T	C	0,0003	52,6925	-0,0952	0,0131	3,9E-13	-0,0857	0,0606	0,1572	instrument
rs1388900	16	73800629	T	C	0,0002	33,4774	-0,0208	0,0036	7,2E-09	-0,0014	0,0173	0,9358	instrument
rs1421896	5	141016288	T	G	0,0002	30,5032	0,0183	0,0033	3,3E-08	0,0174	0,0159	0,2744	instrument
rs142599545	6	19844384	G	A	0,0002	37,4540	0,0812	0,0133	9,4E-10	0,0584	0,0790	0,4603	instrument
rs143384	20	34025756	G	A	0,0011	215,2067	0,0482	0,0033	1,1E-48	0,0034	0,0155	0,8268	instrument
rs143840904	11	2813322	T	C	0,0003	60,4811	-0,0978	0,0126	7,4E-15	-0,1142	0,0762	0,1340	instrument
rs1452815	8	78123634	G	A	0,0002	35,6764	0,0213	0,0036	2,3E-09	-0,0146	0,0170	0,3899	instrument
rs1513304	3	99269750	C	T	0,0002	44,5418	0,0236	0,0035	2,5E-11	0,0079	0,0178	0,6549	instrument
rs1528589	5	88748768	C	T	0,0002	43,3617	0,0222	0,0034	4,6E-11	-0,0048	0,0160	0,7639	instrument
rs1550436	15	74221157	T	C	0,0006	111,7237	-0,0343	0,0032	4,0E-26	0,0037	0,0152	0,8064	instrument
rs16942324	15	89383854	A	C	0,0005	104,6518	-0,1025	0,0100	1,4E-24	-0,0287	0,0513	0,5765	instrument

rs16953461	17	46261736	G	T	0,0003	62,7574	-0,0312	0,0039	2,3E-15	-0,0468	0,0193	0,0153	instrument
rs16996444	19	9997238	C	T	0,0002	47,0866	-0,0245	0,0036	6,8E-12	0,0329	0,0172	0,0565	instrument
rs17194431	8	120612627	T	C	0,0002	29,9206	0,0196	0,0036	4,5E-08	0,0196	0,0171	0,2496	instrument
rs1749824	10	80923862	A	C	0,0002	32,5011	-0,0185	0,0032	1,2E-08	-0,0069	0,0154	0,6554	instrument
rs17511102	2	37960613	T	A	0,0002	34,6446	0,0331	0,0056	4,0E-09	-0,0643	0,0385	0,0940	instrument
rs17654949	2	219890955	T	C	0,0003	51,3940	0,0617	0,0086	7,6E-13	-0,0767	0,0426	0,0718	instrument
rs1805007	16	89986117	T	C	0,0003	56,8359	-0,0395	0,0052	4,7E-14	0,0385	0,0288	0,1807	instrument
rs194059	5	56197416	G	A	0,0003	48,9575	-0,0238	0,0034	2,6E-12	-0,0408	0,0160	0,0108	instrument
rs1952256	1	184035116	G	A	0,0006	106,9145	0,0351	0,0034	4,6E-25	0,0102	0,0161	0,5243	instrument
rs1966265	5	176516631	A	G	0,0003	51,8251	0,0268	0,0037	6,1E-13	0,0054	0,0180	0,7636	instrument
rs1980030	10	120970027	G	A	0,0003	47,7476	0,0227	0,0033	4,8E-12	-0,0145	0,0159	0,3641	instrument
rs2006122	17	61987405	T	A	0,0003	47,1827	0,0252	0,0037	6,5E-12	0,0315	0,0173	0,0680	instrument
rs200889152	6	117507982	C	A	0,0002	42,3146	-0,0218	0,0034	7,8E-11	0,0084	0,0159	0,5982	instrument
rs2035901	4	145521867	G	A	0,0002	30,3267	0,0179	0,0032	3,7E-08	-0,0032	0,0157	0,8382	instrument
rs2050157	6	142658162	A	G	0,0008	146,8929	-0,0434	0,0036	8,4E-34	0,0214	0,0169	0,2048	instrument
rs2076112	22	38538822	T	C	0,0003	64,3999	-0,0261	0,0032	1,0E-15	0,0068	0,0152	0,6559	instrument
rs2194411	3	185548663	A	G	0,0002	42,9020	0,0324	0,0049	5,8E-11	-0,0062	0,0239	0,7951	instrument
rs239458	8	37213496	G	A	0,0002	29,8441	-0,0315	0,0058	4,7E-08	0,0039	0,0273	0,8856	instrument
rs256904	5	55810305	A	T	0,0005	87,0667	0,0345	0,0037	1,1E-20	0,0183	0,0177	0,3012	instrument
rs264762	5	64384386	A	C	0,0003	53,4648	-0,0250	0,0034	2,6E-13	-0,0079	0,0162	0,6256	instrument
rs2712169	2	217671349	G	A	0,0002	44,7289	0,0221	0,0033	2,3E-11	0,0174	0,0155	0,2616	instrument
rs2722285	7	37994955	T	C	0,0002	32,0466	-0,0286	0,0051	1,5E-08	-0,0208	0,0250	0,4048	instrument
rs2731340	3	13559829	C	T	0,0002	47,4440	0,0370	0,0054	5,7E-12	0,0171	0,0255	0,5031	instrument
rs273945	7	137611566	A	C	0,0002	38,3157	-0,0202	0,0033	6,0E-10	0,0078	0,0154	0,6112	instrument
rs2763265	6	168814600	G	A	0,0002	39,0371	-0,0239	0,0038	4,2E-10	0,0258	0,0178	0,1472	instrument
rs2797963	6	34185749	C	T	0,0005	88,1148	0,0556	0,0059	6,2E-21	0,0238	0,0285	0,4037	instrument
rs2811462	3	129303436	G	T	0,0003	53,0561	0,0282	0,0039	3,2E-13	0,0237	0,0186	0,2035	instrument
rs2820443	1	219753509	C	T	0,0006	109,8293	0,0369	0,0035	1,0E-25	0,0006	0,0170	0,9707	instrument
rs2823989	21	18080943	G	A	0,0002	32,8326	-0,0197	0,0034	1,0E-08	-0,0067	0,0162	0,6775	instrument
rs284660	19	41932084	G	T	0,0003	49,9278	0,0233	0,0033	1,6E-12	0,0012	0,0159	0,9395	instrument
rs28539246	17	29226630	A	T	0,0003	65,3180	-0,0268	0,0033	6,4E-16	-0,0437	0,0156	0,0051	outlier

rs2911450	16	88987497	A	C	0,0002	40,2967	-0,0216	0,0034	2,2E-10	0,0327	0,0170	0,0548	instrument
rs3020421	6	152348122	A	G	0,0003	47,8444	0,0240	0,0035	4,6E-12	0,0222	0,0163	0,1732	instrument
rs34134267	7	20417034	A	C	0,0005	86,0247	-0,0304	0,0033	1,8E-20	0,0053	0,0153	0,7283	instrument
rs34517439	1	78450517	A	C	0,0002	30,6138	0,0276	0,0050	3,1E-08	0,0075	0,0249	0,7636	instrument
rs34831515	19	17275777	T	C	0,0002	38,8748	-0,0239	0,0038	4,5E-10	0,0281	0,0183	0,1249	instrument
rs34919557	20	32510282	T	C	0,0002	31,5616	-0,0315	0,0056	1,9E-08	0,0257	0,0274	0,3480	instrument
rs35307904	9	78511889	A	G	0,0007	137,8261	-0,0584	0,0050	8,0E-32	-0,0195	0,0252	0,4386	instrument
rs35699973	18	20726731	G	A	0,0006	113,8477	-0,0419	0,0039	1,5E-26	-0,0552	0,0190	0,0037	outlier
rs35874463	15	67457698	G	A	0,0005	91,8329	0,0663	0,0069	9,6E-22	-0,0322	0,0347	0,3532	instrument
rs3738814	1	17331676	G	A	0,0005	102,4133	-0,0329	0,0033	4,4E-24	-0,0235	0,0154	0,1254	instrument
rs3791679	2	56096892	G	A	0,0012	235,3131	-0,0591	0,0038	4,4E-53	0,0207	0,0178	0,2437	instrument
rs3794782	17	18127914	C	G	0,0002	35,3427	0,0198	0,0033	2,8E-09	-0,0312	0,0157	0,0475	instrument
rs3800162	6	2134391	A	T	0,0003	53,6990	-0,0241	0,0033	2,3E-13	-0,0058	0,0158	0,7127	instrument
rs395523	6	105396187	G	C	0,0006	123,6531	0,0384	0,0035	1,1E-28	0,0145	0,0164	0,3757	instrument
rs39875	16	2268737	G	A	0,0002	32,2620	0,0214	0,0038	1,4E-08	0,0042	0,0176	0,8108	instrument
rs4073717	5	170864021	T	G	0,0002	35,3308	-0,0240	0,0040	2,8E-09	0,0261	0,0192	0,1738	instrument
rs41284816	13	50655989	T	G	0,0003	52,9251	0,0890	0,0122	3,5E-13	-0,0344	0,0528	0,5149	instrument
rs4321401	2	69348216	T	C	0,0003	49,0975	-0,0227	0,0032	2,4E-12	0,0217	0,0165	0,1886	instrument
rs4383453	3	123068359	A	G	0,0002	30,7800	0,0229	0,0041	2,9E-08	-0,0208	0,0200	0,3002	instrument
rs4421120	5	32763118	A	G	0,0004	78,0034	0,0387	0,0044	1,0E-18	-0,0086	0,0214	0,6866	instrument
rs4432932	5	115039414	G	A	0,0002	39,5888	0,0230	0,0037	3,1E-10	0,0049	0,0169	0,7708	instrument
rs4513485	3	49139966	G	A	0,0003	60,8238	-0,0266	0,0034	6,3E-15	-0,0063	0,0161	0,6968	instrument
rs4626892	1	221234485	T	C	0,0002	44,7824	0,0218	0,0033	2,2E-11	0,0058	0,0156	0,7106	instrument
rs4699384	4	100570681	A	T	0,0002	32,4783	0,0234	0,0041	1,2E-08	-0,0073	0,0196	0,7081	instrument
rs4731420	7	127863295	C	G	0,0002	30,8910	0,0234	0,0042	2,7E-08	0,0012	0,0201	0,9537	instrument
rs475390	1	41570459	G	A	0,0002	32,3416	0,0221	0,0039	1,3E-08	0,0102	0,0183	0,5769	instrument
rs4777230	15	70365762	C	T	0,0002	35,9636	0,0274	0,0046	2,0E-09	0,0119	0,0228	0,6035	instrument
rs4793987	17	46855837	C	T	0,0002	36,5175	-0,0202	0,0033	1,5E-09	0,0032	0,0156	0,8368	instrument
rs481237	9	98316094	A	G	0,0003	59,7059	-0,0470	0,0061	1,1E-14	0,0167	0,0304	0,5829	instrument
rs4842133	9	139127566	A	C	0,0005	93,9145	0,0348	0,0036	3,3E-22	-0,0172	0,0176	0,3269	instrument
rs4848645	2	121623691	G	C	0,0002	30,7467	-0,0240	0,0043	3,0E-08	-0,0081	0,0224	0,7182	instrument

rs4867721	5	172983457	G	T	0,0002	38,5637	0,0205	0,0033	5,3E-10	-0,0353	0,0156	0,0240	instrument
rs4901541	14	55235282	C	T	0,0002	39,4757	-0,0208	0,0033	3,3E-10	0,0076	0,0163	0,6417	instrument
rs4923575	11	28876429	C	T	0,0002	40,3729	-0,0207	0,0033	2,1E-10	-0,0124	0,0154	0,4195	instrument
rs4936099	11	130280725	C	A	0,0002	42,7188	-0,0216	0,0033	6,3E-11	-0,0012	0,0164	0,9401	instrument
rs4961091	8	89357874	T	A	0,0002	36,3243	0,0213	0,0035	1,7E-09	0,0022	0,0165	0,8950	instrument
rs496298	10	131355130	A	G	0,0003	58,6137	-0,0261	0,0034	1,9E-14	-0,0370	0,0161	0,0220	instrument
rs537124	7	28203142	C	T	0,0006	114,4888	0,0378	0,0035	9,9E-27	-0,0370	0,0170	0,0293	instrument
rs550373	6	116754150	T	G	0,0002	41,3702	0,0212	0,0033	1,3E-10	0,0136	0,0155	0,3771	instrument
rs56186137	16	28825953	G	A	0,0003	55,5914	0,0245	0,0033	8,9E-14	-0,0054	0,0157	0,7332	instrument
rs57674902	20	4096741	T	C	0,0003	50,9791	0,0243	0,0034	9,3E-13	-0,0108	0,0166	0,5170	instrument
rs584961	11	75277628	A	G	0,0003	55,6063	0,0379	0,0051	8,9E-14	0,0014	0,0255	0,9553	instrument
rs58697961	17	1665424	A	G	0,0002	37,5520	0,0209	0,0034	8,9E-10	0,0095	0,0166	0,5683	instrument
rs60843830	2	286756	G	C	0,0002	33,7325	0,0198	0,0034	6,3E-09	-0,0225	0,0164	0,1697	instrument
rs61732778	3	187443314	A	G	0,0002	32,6837	0,0358	0,0063	1,1E-08	-0,0134	0,0300	0,6566	instrument
rs61734651	20	61451332	T	C	0,0003	51,0362	0,0488	0,0068	9,1E-13	-0,0087	0,0350	0,8025	instrument
rs62055545	17	43964561	T	C	0,0004	78,6761	-0,0345	0,0039	7,4E-19	0,1247	0,0188	4,1E-11	outlier
rs62076439	17	47404628	T	G	0,0004	73,1701	-0,0287	0,0034	1,2E-17	-0,0298	0,0158	0,0594	instrument
rs62271373	3	150066540	A	T	0,0002	38,9247	-0,0441	0,0071	4,4E-10	-0,0393	0,0359	0,2731	instrument
rs62330027	5	318557	A	T	0,0002	29,9863	0,0298	0,0054	4,4E-08	0,0261	0,0259	0,3136	instrument
rs62515452	8	57187356	T	C	0,0002	44,5285	0,0305	0,0046	2,5E-11	0,0030	0,0233	0,8978	instrument
rs62621197	19	8670147	T	C	0,0011	211,1187	-0,1347	0,0093	8,2E-48	0,0262	0,0517	0,6119	instrument
rs6439629	3	135901270	A	G	0,0002	45,9408	-0,0247	0,0036	1,2E-11	0,0111	0,0172	0,5198	instrument
rs6486059	11	12824581	A	G	0,0003	49,8290	0,0232	0,0033	1,7E-12	-0,0093	0,0157	0,5565	instrument
rs6492538	13	91993746	A	C	0,0002	46,7441	0,0268	0,0039	8,1E-12	0,0104	0,0183	0,5690	instrument
rs6557679	8	23383743	T	G	0,0002	35,3070	0,0221	0,0037	2,8E-09	0,0036	0,0173	0,8367	instrument
rs6585827	10	124165615	A	G	0,0002	38,1920	0,0199	0,0032	6,4E-10	0,0207	0,0154	0,1803	instrument
rs6601503	8	10540767	C	T	0,0002	38,8250	0,0230	0,0037	4,6E-10	0,0232	0,0195	0,2332	instrument
rs6739701	2	105132332	G	A	0,0004	66,6829	0,0267	0,0033	3,2E-16	-0,0206	0,0162	0,2041	instrument
rs6777217	3	36979042	A	G	0,0002	33,0047	0,0187	0,0032	9,2E-09	0,0151	0,0155	0,3287	instrument
rs6785012	3	141109348	T	C	0,0015	291,7233	0,0555	0,0032	2,4E-65	-0,0149	0,0154	0,3336	instrument
rs68049170	10	72432047	A	G	0,0003	57,7746	0,0277	0,0036	2,9E-14	0,0003	0,0173	0,9841	instrument

rs6976501	7	50760220	G	A	0,0003	57,6227	0,0294	0,0039	3,2E-14	0,0465	0,0188	0,0134	instrument
rs6978655	7	46171683	T	C	0,0002	39,0246	0,0228	0,0036	4,2E-10	0,0040	0,0175	0,8170	instrument
rs6990354	8	145282578	C	T	0,0002	40,3856	-0,0206	0,0032	2,1E-10	-0,0077	0,0161	0,6330	instrument
rs702605	5	53167028	T	C	0,0002	33,7441	0,0188	0,0032	6,3E-09	-0,0090	0,0153	0,5570	instrument
rs7101582	11	47714610	A	G	0,0002	30,9133	0,0224	0,0040	2,7E-08	-0,0030	0,0193	0,8771	instrument
rs711245	2	36768875	A	G	0,0002	46,4574	-0,0235	0,0034	9,4E-12	0,0109	0,0164	0,5065	instrument
rs7155279	14	92485881	T	G	0,0003	63,3291	-0,0267	0,0034	1,8E-15	-0,0055	0,0159	0,7309	instrument
rs7170686	15	48821350	A	G	0,0003	56,0396	-0,0414	0,0055	7,1E-14	-0,0095	0,0266	0,7215	instrument
rs7184427	16	88804734	A	G	0,0002	30,0301	-0,0248	0,0045	4,3E-08	0,0022	0,0218	0,9180	instrument
rs7223156	17	68679731	T	C	0,0002	34,1637	-0,0206	0,0035	5,1E-09	-0,0486	0,0165	0,0033	outlier
rs72502575	6	31356087	T	C	0,0003	49,9561	-0,0319	0,0045	1,6E-12	-0,0192	0,0199	0,3345	instrument
rs72708236	1	155980320	G	T	0,0003	66,4707	-0,0773	0,0095	3,6E-16	-0,0161	0,0476	0,7358	instrument
rs72755233	15	100692953	A	G	0,0012	238,3911	-0,0784	0,0051	9,8E-54	-0,0173	0,0300	0,5644	instrument
rs72940413	11	69202148	T	C	0,0002	32,3530	-0,0320	0,0056	1,3E-08	-0,0066	0,0257	0,7985	instrument
rs73111557	3	61523251	T	C	0,0002	36,7596	-0,0325	0,0054	1,3E-09	-0,0414	0,0268	0,1222	instrument
rs73407643	6	35169123	C	T	0,0004	68,0618	-0,1022	0,0124	1,6E-16	0,0218	0,0591	0,7126	instrument
rs74469561	3	70929100	T	G	0,0002	39,7904	-0,0383	0,0061	2,8E-10	0,0434	0,0290	0,1348	instrument
rs74829909	4	12964932	C	T	0,0002	31,9448	-0,0305	0,0054	1,6E-08	0,0364	0,0262	0,1651	instrument
rs75223147	6	19831020	G	T	0,0004	81,5942	0,1015	0,0112	1,7E-19	0,0785	0,0756	0,2993	instrument
rs7534091	1	118864616	G	A	0,0003	54,6115	-0,0271	0,0037	1,5E-13	0,0122	0,0175	0,4854	instrument
rs7536227	1	93262390	C	T	0,0002	31,2478	-0,0300	0,0054	2,3E-08	0,0036	0,0258	0,8890	instrument
rs7540754	1	227850588	A	T	0,0004	76,0899	-0,0374	0,0043	2,7E-18	-0,0120	0,0211	0,5696	instrument
rs7674894	4	120680189	G	A	0,0002	37,3806	-0,0212	0,0035	9,7E-10	-0,0008	0,0165	0,9632	instrument
rs7689420	4	145568352	T	C	0,0011	211,4094	-0,0625	0,0043	7,1E-48	0,0074	0,0206	0,7208	instrument
rs7718768	5	178536089	A	G	0,0004	79,9585	0,0305	0,0034	3,9E-19	-0,0173	0,0164	0,2929	instrument
rs7736102	5	131604257	G	A	0,0003	66,0149	-0,0264	0,0032	4,5E-16	-0,0222	0,0153	0,1458	instrument
rs7757703	6	47578705	G	A	0,0002	44,3685	-0,0235	0,0035	2,7E-11	0,0172	0,0165	0,2974	instrument
rs7804293	7	92287849	G	T	0,0004	78,9069	0,0325	0,0037	6,6E-19	0,0254	0,0171	0,1364	instrument
rs781659	4	57779851	G	A	0,0002	33,5817	-0,0189	0,0033	6,8E-09	-0,0208	0,0155	0,1788	instrument
rs78198962	2	233094868	T	C	0,0003	51,3797	0,0655	0,0091	7,6E-13	-0,0593	0,0535	0,2681	instrument
rs7846627	8	130736263	G	A	0,0003	58,7669	-0,0249	0,0032	1,8E-14	0,0072	0,0152	0,6353	instrument

rs7874161	9	109639062	T	C	0,0002	31,3373	0,0189	0,0034	2,2E-08	0,0156	0,0162	0,3361	instrument
rs79112217	1	218622949	C	T	0,0003	61,3239	0,0412	0,0053	4,9E-15	0,0075	0,0257	0,7715	instrument
rs79312203	20	32082962	A	G	0,0002	37,6010	0,0329	0,0054	8,7E-10	-0,0083	0,0256	0,7448	instrument
rs7952436	11	67024534	T	C	0,0009	172,6578	-0,0764	0,0058	1,9E-39	-0,0172	0,0337	0,6090	instrument
rs798488	7	2802522	C	T	0,0010	187,9621	-0,0482	0,0035	9,7E-43	-0,0211	0,0168	0,2095	instrument
rs7994151	13	115038879	G	A	0,0002	36,3846	-0,0271	0,0045	1,6E-09	-0,0328	0,0215	0,1271	instrument
rs8003703	14	33411639	A	T	0,0002	31,3149	-0,0637	0,0114	2,2E-08	0,1097	0,0585	0,0606	instrument
rs80085026	7	148636421	G	C	0,0004	77,0525	0,0364	0,0041	1,7E-18	-0,0041	0,0200	0,8358	instrument
rs8103728	19	33900257	C	G	0,0004	70,8620	-0,0289	0,0034	3,8E-17	0,0078	0,0162	0,6303	instrument
rs8105888	19	19704909	T	A	0,0003	64,1915	0,0324	0,0040	1,1E-15	0,0241	0,0191	0,2054	instrument
rs862041	14	74986403	T	C	0,0004	72,1473	-0,0286	0,0034	2,0E-17	0,0029	0,0161	0,8556	instrument
rs9262136	6	30619988	A	C	0,0002	32,8211	-0,0264	0,0046	1,0E-08	0,0063	0,0209	0,7632	instrument
rs927415	6	7698384	T	C	0,0002	33,7325	-0,0194	0,0033	6,3E-09	0,0066	0,0156	0,6697	instrument
rs9285863	5	108071655	C	T	0,0003	51,5662	0,0246	0,0034	7,0E-13	0,0126	0,0162	0,4372	instrument
rs9321266	6	131316458	G	A	0,0003	56,3995	0,0249	0,0033	5,9E-14	0,0153	0,0157	0,3295	instrument
rs9344005	6	81173371	G	C	0,0002	40,1698	0,0318	0,0050	2,3E-10	-0,0214	0,0249	0,3892	instrument
rs9344115	6	81834237	A	G	0,0002	32,3303	-0,0208	0,0037	1,3E-08	0,0189	0,0172	0,2738	instrument
rs9358912	6	26211146	T	G	0,0012	231,9505	-0,0553	0,0036	2,4E-52	-0,0152	0,0167	0,3640	instrument
rs9442571	1	9349611	A	T	0,0003	66,2915	0,0396	0,0049	3,9E-16	-0,0134	0,0244	0,5821	instrument
rs979012	20	6623374	T	C	0,0004	78,9957	0,0297	0,0033	6,3E-19	0,0170	0,0161	0,2904	instrument
rs994014	4	82165790	C	T	0,0006	118,8088	0,0381	0,0035	1,2E-27	-0,0034	0,0165	0,8388	instrument

Table S19. Single Nucleotide Polymorphisms (SNPs) used as instruments in the Mendelian randomization analyses for the effect of AFR on breast cancer based on the genome-wide significance threshold P = 5E-08. R^2 represents the explained variance in the exposure. SNP-specific instrument strength is given by the respective F-statistic. Some of the genetic variants were identified as outliers in the iterative approach and therefore excluded from the analyses.

SNP	CHR	POS	EA	OA	R^2	F-stat	β_{AFR}	SE_{AFR}	P_{AFR}	β_{Breast}	SE_{Breast}	P_{breast}	$variant_{Breast}$
rs10013032	4	120149419	C	T	0,0002	38,6880	0,0208	0,0033	5,0E-10	-0,0041	0,0066	0,5362	instrument
rs10057598	5	64070812	C	T	0,0002	35,5451	0,0196	0,0033	2,5E-09	-0,0082	0,0063	0,1972	instrument
rs10182458	2	25150641	G	A	0,0010	192,9301	0,0449	0,0032	8,1E-44	-0,0396	0,0066	1,8E-09	outlier
rs10484759	6	127037042	T	C	0,0002	31,1472	0,0208	0,0037	2,4E-08	-0,0003	0,0074	0,9661	instrument
rs1048932	11	115044850	A	C	0,0002	32,5809	-0,0187	0,0033	1,1E-08	0,0060	0,0063	0,3404	instrument

rs10504303	8	60859424	A	T	0,0002	33,6281	-0,0194	0,0034	6,7E-09	-0,0007	0,0069	0,9153	instrument
rs10761785	10	65318766	G	T	0,0002	42,7188	0,0212	0,0032	6,3E-11	0,0239	0,0062	0,0001	outlier
rs10797119	9	92202495	T	C	0,0002	33,4543	-0,0189	0,0033	7,3E-09	0,0055	0,0063	0,3803	instrument
rs10800938	1	190220059	A	G	0,0002	38,7627	0,0202	0,0032	4,8E-10	-0,0053	0,0065	0,4129	instrument
rs10916174	1	227804041	A	G	0,0002	35,8438	0,0266	0,0044	2,1E-09	-0,0078	0,0088	0,3754	instrument
rs10962549	9	16719445	T	C	0,0003	48,5107	0,0303	0,0043	3,3E-12	0,0131	0,0089	0,1426	instrument
rs11030119	11	27728102	A	G	0,0003	61,7004	0,0274	0,0035	4,0E-15	-0,0044	0,0069	0,5246	instrument
rs11159247	14	77509872	A	G	0,0002	31,7642	0,0199	0,0035	1,7E-08	-0,0316	0,0068	3,0E-06	outlier
rs112566467	1	39562627	T	C	0,0002	31,8431	0,0225	0,0040	1,7E-08	0,0079	0,0078	0,3093	instrument
rs112726905	19	32822348	G	A	0,0002	29,8878	-0,0201	0,0037	4,6E-08	-0,0005	0,0076	0,9467	instrument
rs112920189	7	75094545	A	C	0,0003	55,8900	-0,0243	0,0033	7,7E-14	-0,0107	0,0063	0,0919	instrument
rs113996403	2	95911816	C	G	0,0002	31,2925	0,0724	0,0129	2,2E-08	-0,0061	0,0243	0,8012	instrument
rs114938354	5	176732485	C	G	0,0002	40,6530	0,0642	0,0101	1,8E-10	0,0238	0,0198	0,2283	instrument
rs11642015	16	53802494	T	C	0,0020	380,6361	0,0641	0,0033	1,1E-84	-0,0624	0,0063	0,0000	outlier
rs11721374	4	8599520	A	G	0,0002	43,0200	0,0215	0,0033	5,4E-11	-0,0056	0,0068	0,4102	instrument
rs1173734	5	32766417	C	T	0,0002	31,3149	-0,0206	0,0037	2,2E-08	0,0042	0,0076	0,5774	instrument
rs11766944	7	1888051	A	G	0,0002	35,6405	-0,0250	0,0042	2,4E-09	-0,0056	0,0077	0,4719	instrument
rs117700741	19	8637832	T	C	0,0002	31,6403	0,0511	0,0091	1,9E-08	-0,0193	0,0203	0,3406	instrument
rs11851616	14	104065600	A	G	0,0003	57,5620	0,0266	0,0035	3,3E-14	-0,0031	0,0067	0,6467	instrument
rs11881199	19	18837459	T	C	0,0002	36,8202	0,0197	0,0033	1,3E-09	0,0122	0,0066	0,0624	instrument
rs1207949	13	33008393	C	T	0,0002	45,8866	-0,0229	0,0034	1,3E-11	0,0058	0,0065	0,3678	instrument
rs12140153	1	62579891	T	G	0,0002	30,2497	-0,0316	0,0057	3,8E-08	0,0121	0,0116	0,2998	instrument
rs12233810	4	17859768	T	C	0,0002	36,0116	-0,0218	0,0036	2,0E-09	0,0102	0,0071	0,1492	instrument
rs12357890	10	99762693	A	G	0,0002	45,5215	-0,0222	0,0033	1,5E-11	-0,0019	0,0067	0,7736	instrument
rs12432754	14	94030357	A	G	0,0002	40,8444	-0,0216	0,0034	1,6E-10	0,0020	0,0065	0,7601	instrument
rs12575252	11	8694073	C	G	0,0002	31,8093	-0,0193	0,0034	1,7E-08	0,0006	0,0065	0,9243	instrument
rs12805133	11	66483265	G	A	0,0003	53,8750	0,0238	0,0032	2,1E-13	-0,0052	0,0064	0,4128	instrument
rs12926506	16	81722413	T	C	0,0002	32,0353	-0,0247	0,0044	1,5E-08	0,0198	0,0091	0,0306	instrument
rs13002946	2	100801959	A	T	0,0003	51,9547	-0,0265	0,0037	5,7E-13	0,0094	0,0072	0,1904	instrument
rs13107325	4	103188709	T	C	0,0004	70,8620	0,0520	0,0062	3,9E-17	0,0173	0,0125	0,1657	instrument
rs13174863	5	139080745	G	A	0,0002	42,3276	0,0300	0,0046	7,7E-11	0,0188	0,0098	0,0538	instrument

rs13434116	3	136353074	C	G	0,0003	51,1792	0,0271	0,0038	8,4E-13	0,0006	0,0072	0,9335	instrument
rs150468996	1	1591869	C	T	0,0002	36,2280	-0,0215	0,0036	1,8E-09	0,0024	0,0096	0,8007	instrument
rs16942323	15	89383764	C	T	0,0002	35,6764	0,0603	0,0101	2,3E-09	0,0270	0,0229	0,2378	instrument
rs17066856	18	58049656	C	T	0,0003	47,8997	-0,0391	0,0056	4,5E-12	0,0120	0,0107	0,2617	instrument
rs1708302	7	28198677	C	T	0,0003	63,7915	-0,0257	0,0032	1,4E-15	-0,0223	0,0062	0,0003	outlier
rs1715896	15	56828094	G	A	0,0002	38,3528	0,0203	0,0033	5,9E-10	0,0051	0,0065	0,4276	instrument
rs17167306	7	133529648	C	A	0,0002	29,7349	-0,0256	0,0047	4,9E-08	0,0195	0,0091	0,0330	instrument
rs17391694	1	78623626	T	C	0,0002	34,9869	0,0274	0,0046	3,3E-09	0,0070	0,0103	0,4957	instrument
rs17619973	3	114417675	G	A	0,0002	31,4045	-0,0345	0,0061	2,1E-08	-0,0183	0,0128	0,1524	instrument
rs17763953	14	79969194	C	T	0,0002	33,7209	0,0192	0,0033	6,4E-09	0,0163	0,0065	0,0124	outlier
rs17770336	9	28414625	T	C	0,0003	48,7199	0,0240	0,0034	3,0E-12	-0,0036	0,0067	0,5942	instrument
rs1881459	5	88841488	G	A	0,0003	56,3695	0,0251	0,0033	6,0E-14	-0,0109	0,0068	0,1102	instrument
rs1884897	20	6612832	A	G	0,0005	92,6589	-0,0323	0,0034	6,3E-22	0,0060	0,0066	0,3641	instrument
rs200126474	17	47112109	A	C	0,0002	40,2967	-0,0219	0,0034	2,2E-10	0,0003	0,0069	0,9709	instrument
rs205286	6	34551334	C	A	0,0004	75,4458	0,0299	0,0034	3,8E-18	-0,0037	0,0066	0,5724	instrument
rs2076603	1	17319011	G	A	0,0002	39,3878	0,0212	0,0034	3,5E-10	-0,0084	0,0065	0,1971	instrument
rs2101435	2	233115158	G	C	0,0002	30,7024	0,0200	0,0036	3,0E-08	-0,0140	0,0073	0,0560	instrument
rs2239647	14	33292743	A	C	0,0002	37,8344	0,0200	0,0032	7,7E-10	-0,0068	0,0066	0,3037	instrument
rs2289292	16	30097630	T	C	0,0002	39,5260	-0,0216	0,0034	3,3E-10	0,0009	0,0072	0,9012	instrument
rs2295654	14	101539197	T	C	0,0002	38,1179	-0,0231	0,0037	6,7E-10	0,0026	0,0076	0,7338	instrument
rs2299383	7	103418846	T	C	0,0002	46,4574	0,0224	0,0033	9,4E-12	-0,0137	0,0064	0,0319	instrument
rs2346883	2	47015914	G	T	0,0002	46,9631	-0,0233	0,0034	7,2E-12	-0,0033	0,0067	0,6232	instrument
rs2404324	7	99023461	G	A	0,0002	40,7167	-0,0285	0,0045	1,8E-10	-0,0048	0,0085	0,5698	instrument
rs2436661	15	52145407	A	G	0,0002	36,1438	-0,0195	0,0032	1,8E-09	0,0039	0,0063	0,5333	instrument
rs2450444	10	93010383	A	G	0,0002	42,8628	-0,0221	0,0034	5,9E-11	0,0021	0,0065	0,7507	instrument
rs2644135	1	201856256	C	G	0,0002	40,5637	0,0216	0,0034	1,9E-10	-0,0074	0,0066	0,2657	instrument
rs2708149	2	58951187	A	G	0,0003	50,6939	-0,0235	0,0033	1,1E-12	0,0037	0,0063	0,5565	instrument
rs2727316	17	61961336	G	A	0,0002	29,9534	-0,0197	0,0036	4,4E-08	0,0113	0,0070	0,1063	instrument
rs286818	5	107433446	A	T	0,0004	75,2722	-0,0374	0,0043	4,1E-18	-0,0039	0,0082	0,6366	instrument
rs2953806	8	8914124	T	C	0,0004	68,3095	0,0270	0,0033	1,4E-16	-0,0211	0,0074	0,0045	instrument
rs2994320	1	243641247	G	A	0,0002	32,2848	-0,0232	0,0041	1,3E-08	0,0029	0,0081	0,7175	instrument

rs34409196	17	70703396	C	A	0,0002	34,4448	0,0211	0,0036	4,4E-09	-0,0007	0,0073	0,9269	instrument
rs34811474	4	25408838	A	G	0,0004	75,3937	-0,0331	0,0038	3,9E-18	-0,0045	0,0089	0,6092	instrument
rs35559811	13	58666728	G	A	0,0003	61,7632	-0,0301	0,0038	3,9E-15	-0,0065	0,0073	0,3720	instrument
rs3734554	6	40360781	T	C	0,0002	38,0068	0,0216	0,0035	7,1E-10	-0,0013	0,0074	0,8618	instrument
rs3784710	15	68072458	C	T	0,0003	57,2589	-0,0291	0,0038	3,8E-14	0,0069	0,0074	0,3524	instrument
rs3810291	19	47569003	G	A	0,0003	66,1938	-0,0281	0,0035	4,1E-16	0,0236	0,0067	0,0004	outlier
rs3826408	17	7101292	T	C	0,0002	32,1032	0,0183	0,0032	1,5E-08	0,0134	0,0063	0,0328	instrument
rs3845836	2	219504955	C	T	0,0002	42,5621	0,0212	0,0033	6,9E-11	0,0031	0,0063	0,6260	instrument
rs3895907	1	75006027	A	G	0,0003	51,3510	0,0234	0,0033	7,8E-13	-0,0091	0,0062	0,1444	instrument
rs41271299	6	19839415	T	C	0,0002	30,2937	-0,0402	0,0073	3,7E-08	-0,0379	0,0208	0,0683	instrument
rs4304660	10	22295747	G	A	0,0002	34,9750	0,0217	0,0037	3,3E-09	0,0190	0,0070	0,0065	outlier
rs4482463	2	205375909	C	A	0,0002	36,6869	0,0372	0,0061	1,4E-09	-0,0148	0,0119	0,2161	instrument
rs4680340	3	156861460	C	T	0,0002	30,2277	0,0204	0,0037	3,8E-08	-0,0026	0,0072	0,7140	instrument
rs4790841	17	1835482	T	C	0,0003	57,7290	-0,0340	0,0045	3,0E-14	0,0111	0,0089	0,2127	instrument
rs4794670	17	54927829	T	A	0,0002	30,6802	-0,0214	0,0039	3,0E-08	0,0042	0,0088	0,6315	instrument
rs4796225	17	34843839	G	A	0,0002	38,1179	-0,0200	0,0032	6,7E-10	-0,0105	0,0071	0,1388	instrument
rs4808944	19	19426613	C	A	0,0002	39,0246	-0,0276	0,0044	4,2E-10	0,0336	0,0085	0,0001	outlier
rs4821943	22	40722745	G	A	0,0002	35,5093	-0,0204	0,0034	2,5E-09	0,0369	0,0064	1,0E-08	outlier
rs4842838	15	84582124	G	T	0,0004	74,2864	0,0278	0,0032	6,8E-18	0,0059	0,0062	0,3407	instrument
rs4889606	16	31011183	G	A	0,0004	67,1245	-0,0274	0,0033	2,6E-16	0,0140	0,0065	0,0319	instrument
rs539515	1	177889025	C	A	0,0008	161,7967	0,0505	0,0040	5,0E-37	-0,0303	0,0079	0,0001	outlier
rs55654354	3	82643954	C	T	0,0002	35,7122	-0,0258	0,0043	2,3E-09	0,0044	0,0084	0,6029	instrument
rs56282717	7	150657095	A	G	0,0002	35,3308	-0,0225	0,0038	2,8E-09	0,0138	0,0073	0,0591	instrument
rs61037750	11	43681372	A	G	0,0002	37,4418	0,0214	0,0035	9,5E-10	0,0079	0,0068	0,2393	instrument
rs61813324	1	156049877	T	C	0,0002	42,5230	0,0317	0,0049	7,0E-11	-0,0194	0,0102	0,0582	instrument
rs61910820	11	134581277	G	A	0,0002	32,2280	0,0243	0,0043	1,4E-08	-0,0062	0,0090	0,4953	instrument
rs62004866	15	74223118	G	C	0,0002	44,7289	0,0350	0,0052	2,3E-11	0,0291	0,0101	0,0039	outlier
rs62106258	2	417167	C	T	0,0008	145,2010	-0,0899	0,0075	2,1E-33	0,0142	0,0181	0,4318	instrument
rs62131065	2	25568539	G	A	0,0002	33,3041	-0,0312	0,0054	7,9E-09	0,0084	0,0103	0,4159	instrument
rs6548237	2	621461	A	C	0,0007	137,8262	-0,0500	0,0043	8,4E-32	0,0214	0,0105	0,0416	instrument
rs6585200	10	114768609	G	A	0,0002	45,1848	-0,0218	0,0032	1,8E-11	0,0440	0,0062	1,1E-12	outlier

rs6606652	11	69499786	G	T	0,0002	36,0717	-0,0201	0,0033	1,9E-09	-0,0244	0,0066	0,0002	outlier
rs66922415	18	57848651	G	A	0,0009	163,8383	0,0485	0,0038	1,8E-37	-0,0228	0,0073	0,0018	instrument
rs6852276	4	145261999	G	A	0,0002	33,1427	0,0191	0,0033	8,6E-09	0,0067	0,0068	0,3214	instrument
rs7045925	9	96475306	T	C	0,0002	39,1371	0,0231	0,0037	4,0E-10	-0,0003	0,0072	0,9635	instrument
rs7124681	11	47529947	A	C	0,0003	63,2336	0,0261	0,0033	1,8E-15	-0,0043	0,0063	0,5005	instrument
rs72731237	9	34148743	A	G	0,0002	34,8332	-0,0309	0,0052	3,6E-09	0,0020	0,0095	0,8330	instrument
rs72740699	5	27188818	C	G	0,0002	31,1919	-0,0202	0,0036	2,3E-08	0,0084	0,0073	0,2463	instrument
rs72755233	15	100692953	A	G	0,0004	72,3343	0,0432	0,0051	1,8E-17	0,0039	0,0127	0,7583	instrument
rs72771049	16	19933511	T	G	0,0003	54,8038	-0,0342	0,0046	1,3E-13	0,0134	0,0090	0,1369	instrument
rs72892910	6	50816887	T	G	0,0004	76,1772	0,0376	0,0043	2,6E-18	0,0093	0,0081	0,2519	instrument
rs73052033	3	185828465	C	T	0,0004	70,5929	-0,0351	0,0042	4,4E-17	-0,0075	0,0083	0,3622	instrument
rs7459710	8	77222670	T	C	0,0002	36,1438	-0,0196	0,0033	1,8E-09	0,0134	0,0062	0,0314	instrument
rs7534091	1	118864616	G	A	0,0002	33,4890	0,0212	0,0037	7,2E-09	-0,0151	0,0073	0,0388	instrument
rs7795394	7	113560607	T	A	0,0002	33,1657	0,0192	0,0033	8,5E-09	0,0111	0,0066	0,0915	instrument
rs879620	16	4015729	C	T	0,0002	34,4565	-0,0195	0,0033	4,4E-09	-0,0024	0,0066	0,7122	instrument
rs925421	4	60253877	A	G	0,0002	34,2221	0,0218	0,0037	4,9E-09	0,0143	0,0075	0,0556	instrument
rs9688977	6	154336892	C	T	0,0002	31,2813	0,0257	0,0046	2,2E-08	-0,0079	0,0089	0,3752	instrument
rs9929088	16	29001460	A	C	0,0002	39,2373	0,0218	0,0035	3,8E-10	-0,0052	0,0070	0,4609	instrument

Table S20. Single Nucleotide Polymorphisms (SNPs) used as instruments in the Mendelian randomization analyses for the effect of AFR on endometrial cancer based on the genome-wide significance threshold P = 5E-08. R^2 represents the explained variance in the exposure. SNP-specific instrument strength is given by the respective F-statistic. Some of the genetic variants were identified as outliers in the iterative approach and therefore excluded from the analyses.

SNP	CHR	POS	EA	OA	R^2	F-stat	β_{AFR}	SE_{AFR}	P_{AFR}	$\beta_{Endometrial}$	$SE_{Endometrial}$	$P_{Endometrial}$	$variant_{Endometrial}$
rs10013032	4	120149419	C	T	0,0002	38,6880	0,0208	0,0033	5,0E-10	-0,0028	0,0166	0,8639	instrument
rs10057598	5	64070812	C	T	0,0002	35,5451	0,0196	0,0033	2,5E-09	-0,0049	0,0160	0,7612	instrument
rs10182458	2	25150641	G	A	0,0010	192,9301	0,0449	0,0032	8,1E-44	0,0137	0,0163	0,3988	instrument
rs10484759	6	127037042	T	C	0,0002	31,1472	0,0208	0,0037	2,4E-08	0,0080	0,0185	0,6662	instrument
rs1048932	11	115044850	A	C	0,0002	32,5809	-0,0187	0,0033	1,1E-08	0,0143	0,0161	0,3742	instrument
rs10504303	8	60859424	A	T	0,0002	33,6281	-0,0194	0,0034	6,7E-09	0,0104	0,0168	0,5365	instrument
rs10761785	10	65318766	G	T	0,0002	42,7188	0,0212	0,0032	6,3E-11	0,0115	0,0158	0,4675	instrument
rs10797119	9	92202495	T	C	0,0002	33,4543	-0,0189	0,0033	7,3E-09	0,0230	0,0159	0,1482	instrument

rs10800938	1	190220059	A	G	0,0002	38,7627	0,0202	0,0032	4,8E-10	-0,0146	0,0162	0,3684	instrument
rs10916174	1	227804041	A	G	0,0002	35,8438	0,0266	0,0044	2,1E-09	-0,0342	0,0223	0,1253	instrument
rs10962549	9	16719445	T	C	0,0003	48,5107	0,0303	0,0043	3,3E-12	-0,0109	0,0219	0,6205	instrument
rs11030119	11	27728102	A	G	0,0003	61,7004	0,0274	0,0035	4,0E-15	0,0659	0,0171	0,0001	outlier
rs11049696	12	28681855	G	A	0,0002	32,8211	0,0199	0,0035	1,0E-08	-0,0309	0,0171	0,0707	instrument
rs11159247	14	77509872	A	G	0,0002	31,7642	0,0199	0,0035	1,7E-08	-0,0180	0,0173	0,2976	instrument
rs112566467	1	39562627	T	C	0,0002	31,8431	0,0225	0,0040	1,7E-08	0,0384	0,0194	0,0481	instrument
rs112726905	19	32822348	G	A	0,0002	29,8878	-0,0201	0,0037	4,6E-08	-0,0307	0,0187	0,1005	instrument
rs112920189	7	75094545	A	C	0,0003	55,8900	-0,0243	0,0033	7,7E-14	-0,0064	0,0165	0,6983	instrument
rs113996403	2	95911816	C	G	0,0002	31,2925	0,0724	0,0129	2,2E-08	0,0587	0,0609	0,3346	instrument
rs114938354	5	176732485	C	G	0,0002	40,6530	0,0642	0,0101	1,8E-10	-0,0137	0,0496	0,7825	instrument
rs11642015	16	53802494	T	C	0,0020	380,6361	0,0641	0,0033	1,1E-84	0,0553	0,0159	0,0005	instrument
rs11721374	4	8599520	A	G	0,0002	43,0200	0,0215	0,0033	5,4E-11	0,0241	0,0170	0,1551	instrument
rs1173734	5	32766417	C	T	0,0002	31,3149	-0,0206	0,0037	2,2E-08	-0,0189	0,0186	0,3097	instrument
rs11766944	7	1888051	A	G	0,0002	35,6405	-0,0250	0,0042	2,4E-09	-0,0319	0,0200	0,1098	instrument
rs117700741	19	8637832	T	C	0,0002	31,6403	0,0511	0,0091	1,9E-08	-0,1106	0,0541	0,0408	instrument
rs11851616	14	104065600	A	G	0,0003	57,5620	0,0266	0,0035	3,3E-14	-0,0213	0,0172	0,2163	instrument
rs11881199	19	18837459	T	C	0,0002	36,8202	0,0197	0,0033	1,3E-09	0,0270	0,0163	0,0966	instrument
rs1207949	13	33008393	C	T	0,0002	45,8866	-0,0229	0,0034	1,3E-11	-0,0107	0,0166	0,5201	instrument
rs12140153	1	62579891	T	G	0,0002	30,2497	-0,0316	0,0057	3,8E-08	-0,0220	0,0289	0,4477	instrument
rs12233810	4	17859768	T	C	0,0002	36,0116	-0,0218	0,0036	2,0E-09	-0,0058	0,0179	0,7449	instrument
rs12357890	10	99762693	A	G	0,0002	45,5215	-0,0222	0,0033	1,5E-11	-0,0098	0,0166	0,5529	instrument
rs12432754	14	94030357	A	G	0,0002	40,8444	-0,0216	0,0034	1,6E-10	0,0149	0,0165	0,3672	instrument
rs12805133	11	66483265	G	A	0,0003	53,8750	0,0238	0,0032	2,1E-13	-0,0188	0,0162	0,2433	instrument
rs12926506	16	81722413	T	C	0,0002	32,0353	-0,0247	0,0044	1,5E-08	0,0055	0,0229	0,8116	instrument
rs13002946	2	100801959	A	T	0,0003	51,9547	-0,0265	0,0037	5,7E-13	-0,0108	0,0183	0,5534	instrument
rs13107325	4	103188709	T	C	0,0004	70,8620	0,0520	0,0062	3,9E-17	-0,0106	0,0315	0,7356	instrument
rs13174863	5	139080745	G	A	0,0002	42,3276	0,0300	0,0046	7,7E-11	0,0198	0,0240	0,4074	instrument
rs13434116	3	136353074	C	G	0,0003	51,1792	0,0271	0,0038	8,4E-13	0,0048	0,0185	0,7955	instrument
rs150468996	1	1591869	C	T	0,0002	36,2280	-0,0215	0,0036	1,8E-09	-0,0062	0,0279	0,8234	instrument
rs16942323	15	89383764	C	T	0,0002	35,6764	0,0603	0,0101	2,3E-09	0,0232	0,0555	0,6762	instrument

rs17066856	18	58049656	C	T	0,0003	47,8997	-0,0391	0,0056	4,5E-12	0,0035	0,0276	0,8990	instrument
rs1708302	7	28198677	C	T	0,0003	63,7915	-0,0257	0,0032	1,4E-15	0,0048	0,0158	0,7588	instrument
rs1715896	15	56828094	G	A	0,0002	38,3528	0,0203	0,0033	5,9E-10	0,0030	0,0162	0,8542	instrument
rs17167306	7	133529648	C	A	0,0002	29,7349	-0,0256	0,0047	4,9E-08	0,0131	0,0233	0,5733	instrument
rs17391694	1	78623626	T	C	0,0002	34,9869	0,0274	0,0046	3,3E-09	0,0444	0,0246	0,0713	instrument
rs17619973	3	114417675	G	A	0,0002	31,4045	-0,0345	0,0061	2,1E-08	-0,0096	0,0317	0,7620	instrument
rs17770336	9	28414625	T	C	0,0003	48,7199	0,0240	0,0034	3,0E-12	0,0228	0,0169	0,1766	instrument
rs1881459	5	88841488	G	A	0,0003	56,3695	0,0251	0,0033	6,0E-14	0,0135	0,0169	0,4257	instrument
rs1884897	20	6612832	A	G	0,0005	92,6589	-0,0323	0,0034	6,3E-22	-0,0284	0,0165	0,0866	instrument
rs200126474	17	47112109	A	C	0,0002	40,2967	-0,0219	0,0034	2,2E-10	-0,0049	0,0175	0,7781	instrument
rs205286	6	34551334	C	A	0,0004	75,4458	0,0299	0,0034	3,8E-18	-0,0015	0,0168	0,9294	instrument
rs2076603	1	17319011	G	A	0,0002	39,3878	0,0212	0,0034	3,5E-10	0,0204	0,0164	0,2153	instrument
rs2101435	2	233115158	G	C	0,0002	30,7024	0,0200	0,0036	3,0E-08	0,0138	0,0182	0,4472	instrument
rs2239647	14	33292743	A	C	0,0002	37,8344	0,0200	0,0032	7,7E-10	0,0287	0,0164	0,0809	instrument
rs2289292	16	30097630	T	C	0,0002	39,5260	-0,0216	0,0034	3,3E-10	-0,0088	0,0175	0,6142	instrument
rs2292238	12	56493822	C	A	0,0002	35,7481	-0,0197	0,0033	2,3E-09	-0,0244	0,0165	0,1401	instrument
rs2299383	7	103418846	T	C	0,0002	46,4574	0,0224	0,0033	9,4E-12	-0,0010	0,0161	0,9512	instrument
rs2404324	7	99023461	G	A	0,0002	40,7167	-0,0285	0,0045	1,8E-10	-0,0380	0,0221	0,0852	instrument
rs2436661	15	52145407	A	G	0,0002	36,1438	-0,0195	0,0032	1,8E-09	-0,0194	0,0158	0,2190	instrument
rs2450444	10	93010383	A	G	0,0002	42,8628	-0,0221	0,0034	5,9E-11	0,0040	0,0166	0,8094	instrument
rs2708149	2	58951187	A	G	0,0003	50,6939	-0,0235	0,0033	1,1E-12	0,0011	0,0161	0,9448	instrument
rs2712667	12	99588917	G	C	0,0002	31,4493	0,0190	0,0034	2,1E-08	0,0003	0,0164	0,9866	instrument
rs2727316	17	61961336	G	A	0,0002	29,9534	-0,0197	0,0036	4,4E-08	0,0121	0,0175	0,4882	instrument
rs286818	5	107433446	A	T	0,0004	75,2722	-0,0374	0,0043	4,1E-18	-0,0453	0,0213	0,0332	instrument
rs2953806	8	8914124	T	C	0,0004	68,3095	0,0270	0,0033	1,4E-16	-0,0069	0,0196	0,7255	instrument
rs2994320	1	243641247	G	A	0,0002	32,2848	-0,0232	0,0041	1,3E-08	0,0221	0,0202	0,2727	instrument
rs34409196	17	70703396	C	A	0,0002	34,4448	0,0211	0,0036	4,4E-09	-0,0262	0,0183	0,1511	instrument
rs34811474	4	25408838	A	G	0,0004	75,3937	-0,0331	0,0038	3,9E-18	0,0128	0,0220	0,5591	instrument
rs35559811	13	58666728	G	A	0,0003	61,7632	-0,0301	0,0038	3,9E-15	-0,0348	0,0188	0,0647	instrument
rs3734554	6	40360781	T	C	0,0002	38,0068	0,0216	0,0035	7,1E-10	0,0366	0,0178	0,0399	instrument
rs3784710	15	68072458	C	T	0,0003	57,2589	-0,0291	0,0038	3,8E-14	-0,0452	0,0190	0,0173	instrument

rs3826408	17	7101292	T	C	0,0002	32,1032	0,0183	0,0032	1,5E-08	0,0132	0,0159	0,4084	instrument
rs3845836	2	219504955	C	T	0,0002	42,5621	0,0212	0,0033	6,9E-11	0,0009	0,0160	0,9538	instrument
rs3895907	1	75006027	A	G	0,0003	51,3510	0,0234	0,0033	7,8E-13	-0,0008	0,0158	0,9597	instrument
rs4304660	10	22295747	G	A	0,0002	34,9750	0,0217	0,0037	3,3E-09	0,0421	0,0176	0,0168	instrument
rs4482463	2	205375909	C	A	0,0002	36,6869	0,0372	0,0061	1,4E-09	-0,0064	0,0301	0,8315	instrument
rs4680340	3	156861460	C	T	0,0002	30,2277	0,0204	0,0037	3,8E-08	-0,0278	0,0183	0,1289	instrument
rs4790841	17	1835482	T	C	0,0003	57,7290	-0,0340	0,0045	3,0E-14	0,0101	0,0224	0,6517	instrument
rs4794670	17	54927829	T	A	0,0002	30,6802	-0,0214	0,0039	3,0E-08	-0,0357	0,0206	0,0828	instrument
rs4796225	17	34843839	G	A	0,0002	38,1179	-0,0200	0,0032	6,7E-10	-0,0095	0,0168	0,5707	instrument
rs4808944	19	19426613	C	A	0,0002	39,0246	-0,0276	0,0044	4,2E-10	-0,0150	0,0218	0,4922	instrument
rs4821943	22	40722745	G	A	0,0002	35,5093	-0,0204	0,0034	2,5E-09	-0,0284	0,0165	0,0857	instrument
rs4842838	15	84582124	G	T	0,0004	74,2864	0,0278	0,0032	6,8E-18	-0,0200	0,0158	0,2052	instrument
rs4889606	16	31011183	G	A	0,0004	67,1245	-0,0274	0,0033	2,6E-16	-0,0205	0,0165	0,2139	instrument
rs539515	1	177889025	C	A	0,0008	161,7967	0,0505	0,0040	5,0E-37	0,0006	0,0198	0,9738	instrument
rs55654354	3	82643954	C	T	0,0002	35,7122	-0,0258	0,0043	2,3E-09	0,0139	0,0216	0,5217	instrument
rs56282717	7	150657095	A	G	0,0002	35,3308	-0,0225	0,0038	2,8E-09	-0,0033	0,0187	0,8599	instrument
rs61037750	11	43681372	A	G	0,0002	37,4418	0,0214	0,0035	9,5E-10	-0,0126	0,0172	0,4650	instrument
rs61813324	1	156049877	T	C	0,0002	42,5230	0,0317	0,0049	7,0E-11	0,0120	0,0254	0,6375	instrument
rs61910820	11	134581277	G	A	0,0002	32,2280	0,0243	0,0043	1,4E-08	0,0146	0,0219	0,5070	instrument
rs62004866	15	74223118	G	C	0,0002	44,7289	0,0350	0,0052	2,3E-11	0,0548	0,0253	0,0301	instrument
rs62106258	2	417167	C	T	0,0008	145,2010	-0,0899	0,0075	2,1E-33	-0,1133	0,0461	0,0140	instrument
rs62131065	2	25568539	G	A	0,0002	33,3041	-0,0312	0,0054	7,9E-09	0,0293	0,0258	0,2561	instrument
rs6548237	2	621461	A	C	0,0007	137,8262	-0,0500	0,0043	8,4E-32	-0,0428	0,0263	0,1032	instrument
rs6585200	10	114768609	G	A	0,0002	45,1848	-0,0218	0,0032	1,8E-11	-0,0121	0,0159	0,4485	instrument
rs66922415	18	57848651	G	A	0,0009	163,8383	0,0485	0,0038	1,8E-37	0,0131	0,0186	0,4792	instrument
rs6852276	4	145261999	G	A	0,0002	33,1427	0,0191	0,0033	8,6E-09	0,0257	0,0166	0,1221	instrument
rs704061	12	89771903	C	T	0,0002	39,0746	0,0203	0,0032	4,1E-10	0,0040	0,0167	0,8096	instrument
rs7045925	9	96475306	T	C	0,0002	39,1371	0,0231	0,0037	4,0E-10	-0,0277	0,0183	0,1306	instrument
rs7124681	11	47529947	A	C	0,0003	63,2336	0,0261	0,0033	1,8E-15	0,0225	0,0161	0,1616	instrument
rs7132908	12	50263148	A	G	0,0003	51,0219	0,0237	0,0033	9,2E-13	0,0299	0,0163	0,0670	instrument
rs72731237	9	34148743	A	G	0,0002	34,8332	-0,0309	0,0052	3,6E-09	0,0023	0,0248	0,9271	instrument

rs72740699	5	27188818	C	G	0,0002	31,1919	-0,0202	0,0036	2,3E-08	-0,0281	0,0180	0,1189	instrument
rs72755233	15	100692953	A	G	0,0004	72,3343	0,0432	0,0051	1,8E-17	-0,0123	0,0309	0,6900	instrument
rs72771049	16	19933511	T	G	0,0003	54,8038	-0,0342	0,0046	1,3E-13	-0,0789	0,0229	0,0006	outlier
rs72892910	6	50816887	T	G	0,0004	76,1772	0,0376	0,0043	2,6E-18	0,0290	0,0207	0,1598	instrument
rs73052033	3	185828465	C	T	0,0004	70,5929	-0,0351	0,0042	4,4E-17	-0,0190	0,0210	0,3667	instrument
rs7459710	8	77222670	T	C	0,0002	36,1438	-0,0196	0,0033	1,8E-09	-0,0362	0,0162	0,0253	instrument
rs7534091	1	118864616	G	A	0,0002	33,4890	0,0212	0,0037	7,2E-09	0,0066	0,0182	0,7179	instrument
rs7795394	7	113560607	T	A	0,0002	33,1657	0,0192	0,0033	8,5E-09	0,0045	0,0165	0,7862	instrument
rs879620	16	4015729	C	T	0,0002	34,4565	-0,0195	0,0033	4,4E-09	-0,0126	0,0166	0,4453	instrument
rs925421	4	60253877	A	G	0,0002	34,2221	0,0218	0,0037	4,9E-09	0,0044	0,0186	0,8128	instrument
rs9688977	6	154336892	C	T	0,0002	31,2813	0,0257	0,0046	2,2E-08	0,0254	0,0222	0,2531	instrument
rs9929088	16	29001460	A	C	0,0002	39,2373	0,0218	0,0035	3,8E-10	0,0317	0,0175	0,0693	instrument

Table S21. Single Nucleotide Polymorphisms (SNPs) used as instruments in the Mendelian randomization analyses for the effect of AFR on ovarian cancer based on the genome-wide significance threshold P = 5E-08. R^2 represents the explained variance in the exposure. SNP-specific instrument strength is given by the respective F-statistic. Some of the genetic variants were identified as outliers in the iterative approach and therefore excluded from the analyses.

SNP	CHR	POS	EA	OA	R^2	F-stat	β_{AFR}	SE_{AFR}	P_{AFR}	$\beta_{Ovarian}$	$SE_{Ovarian}$	$P_{Ovarian}$	$variant_{Ovarian}$
rs10013032	4	120149419	C	T	0,0002	38,6880	0,0208	0,0033	5,0E-10	0,0073	0,0139	0,6012	instrument
rs10057598	5	64070812	C	T	0,0002	35,5451	0,0196	0,0033	2,5E-09	0,0209	0,0136	0,1236	instrument
rs10182458	2	25150641	G	A	0,0010	192,9301	0,0449	0,0032	8,1E-44	0,0079	0,0136	0,5582	instrument
rs10484759	6	127037042	T	C	0,0002	31,1472	0,0208	0,0037	2,4E-08	0,0251	0,0156	0,1084	instrument
rs1048932	11	115044850	A	C	0,0002	32,5809	-0,0187	0,0033	1,1E-08	0,0178	0,0135	0,1857	instrument
rs10504303	8	60859424	A	T	0,0002	33,6281	-0,0194	0,0034	6,7E-09	0,0051	0,0140	0,7173	instrument
rs10761785	10	65318766	G	T	0,0002	42,7188	0,0212	0,0032	6,3E-11	-0,0064	0,0132	0,6280	instrument
rs10797119	9	92202495	T	C	0,0002	33,4543	-0,0189	0,0033	7,3E-09	-0,0062	0,0134	0,6436	instrument
rs10800938	1	190220059	A	G	0,0002	38,7627	0,0202	0,0032	4,8E-10	0,0149	0,0135	0,2707	instrument
rs10916174	1	227804041	A	G	0,0002	35,8438	0,0266	0,0044	2,1E-09	-0,0189	0,0189	0,3178	instrument
rs10962549	9	16719445	T	C	0,0003	48,5107	0,0303	0,0043	3,3E-12	0,0385	0,0184	0,0365	instrument
rs11030119	11	27728102	A	G	0,0003	61,7004	0,0274	0,0035	4,0E-15	0,0139	0,0146	0,3421	instrument
rs11159247	14	77509872	A	G	0,0002	31,7642	0,0199	0,0035	1,7E-08	-0,0082	0,0144	0,5663	instrument
rs112566467	1	39562627	T	C	0,0002	31,8431	0,0225	0,0040	1,7E-08	-0,0439	0,0165	0,0079	outlier

rs112726905	19	32822348	G	A	0,0002	29,8878	-0,0201	0,0037	4,6E-08	-0,0082	0,0155	0,5942	instrument
rs112920189	7	75094545	A	C	0,0003	55,8900	-0,0243	0,0033	7,7E-14	0,0123	0,0136	0,3635	instrument
rs113996403	2	95911816	C	G	0,0002	31,2925	0,0724	0,0129	2,2E-08	-0,0344	0,0504	0,4943	instrument
rs114938354	5	176732485	C	G	0,0002	40,6530	0,0642	0,0101	1,8E-10	0,0142	0,0415	0,7318	instrument
rs11642015	16	53802494	T	C	0,0020	380,6361	0,0641	0,0033	1,1E-84	0,0207	0,0133	0,1206	instrument
rs11721374	4	8599520	A	G	0,0002	43,0200	0,0215	0,0033	5,4E-11	0,0066	0,0139	0,6356	instrument
rs1173734	5	32766417	C	T	0,0002	31,3149	-0,0206	0,0037	2,2E-08	0,0011	0,0155	0,9423	instrument
rs11766944	7	1888051	A	G	0,0002	35,6405	-0,0250	0,0042	2,4E-09	-0,0038	0,0165	0,8163	instrument
rs117700741	19	8637832	T	C	0,0002	31,6403	0,0511	0,0091	1,9E-08	0,0149	0,0405	0,7136	instrument
rs11851616	14	104065600	A	G	0,0003	57,5620	0,0266	0,0035	3,3E-14	0,0154	0,0144	0,2853	instrument
rs11881199	19	18837459	T	C	0,0002	36,8202	0,0197	0,0033	1,3E-09	0,0001	0,0135	0,9968	instrument
rs1207949	13	33008393	C	T	0,0002	45,8866	-0,0229	0,0034	1,3E-11	0,0025	0,0139	0,8565	instrument
rs12140153	1	62579891	T	G	0,0002	30,2497	-0,0316	0,0057	3,8E-08	0,0065	0,0244	0,7911	instrument
rs12233810	4	17859768	T	C	0,0002	36,0116	-0,0218	0,0036	2,0E-09	0,0125	0,0151	0,4083	instrument
rs12357890	10	99762693	A	G	0,0002	45,5215	-0,0222	0,0033	1,5E-11	0,0093	0,0137	0,4974	instrument
rs12432754	14	94030357	A	G	0,0002	40,8444	-0,0216	0,0034	1,6E-10	0,0038	0,0138	0,7808	instrument
rs12575252	11	8694073	C	G	0,0002	31,8093	-0,0193	0,0034	1,7E-08	-0,0257	0,0139	0,0647	instrument
rs12805133	11	66483265	G	A	0,0003	53,8750	0,0238	0,0032	2,1E-13	-0,0177	0,0134	0,1853	instrument
rs12926506	16	81722413	T	C	0,0002	32,0353	-0,0247	0,0044	1,5E-08	0,0303	0,0193	0,1168	instrument
rs13002946	2	100801959	A	T	0,0003	51,9547	-0,0265	0,0037	5,7E-13	-0,0306	0,0152	0,0441	instrument
rs13107325	4	103188709	T	C	0,0004	70,8620	0,0520	0,0062	3,9E-17	0,0490	0,0255	0,0548	instrument
rs13174863	5	139080745	G	A	0,0002	42,3276	0,0300	0,0046	7,7E-11	-0,0049	0,0197	0,8023	instrument
rs13434116	3	136353074	C	G	0,0003	51,1792	0,0271	0,0038	8,4E-13	0,0089	0,0155	0,5671	instrument
rs150468996	1	1591869	C	T	0,0002	36,2280	-0,0215	0,0036	1,8E-09	0,0137	0,0182	0,4510	instrument
rs16942323	15	89383764	C	T	0,0002	35,6764	0,0603	0,0101	2,3E-09	-0,0284	0,0445	0,5232	instrument
rs17066856	18	58049656	C	T	0,0003	47,8997	-0,0391	0,0056	4,5E-12	-0,0368	0,0229	0,1088	instrument
rs1708302	7	28198677	C	T	0,0003	63,7915	-0,0257	0,0032	1,4E-15	-0,0051	0,0132	0,6996	instrument
rs1715896	15	56828094	G	A	0,0002	38,3528	0,0203	0,0033	5,9E-10	-0,0361	0,0134	0,0070	outlier
rs17167306	7	133529648	C	A	0,0002	29,7349	-0,0256	0,0047	4,9E-08	-0,0258	0,0194	0,1852	instrument
rs17391694	1	78623626	T	C	0,0002	34,9869	0,0274	0,0046	3,3E-09	0,0040	0,0209	0,8479	instrument
rs17619973	3	114417675	G	A	0,0002	31,4045	-0,0345	0,0061	2,1E-08	0,0392	0,0268	0,1437	instrument

rs17763953	14	79969194	C	T	0,0002	33,7209	0,0192	0,0033	6,4E-09	-0,0145	0,0137	0,2872	instrument
rs17770336	9	28414625	T	C	0,0003	48,7199	0,0240	0,0034	3,0E-12	0,0280	0,0143	0,0502	instrument
rs1881459	5	88841488	G	A	0,0003	56,3695	0,0251	0,0033	6,0E-14	-0,0070	0,0142	0,6214	instrument
rs1884897	20	6612832	A	G	0,0005	92,6589	-0,0323	0,0034	6,3E-22	0,0076	0,0139	0,5827	instrument
rs200126474	17	47112109	A	C	0,0002	40,2967	-0,0219	0,0034	2,2E-10	0,0050	0,0142	0,7237	instrument
rs205286	6	34551334	C	A	0,0004	75,4458	0,0299	0,0034	3,8E-18	0,0142	0,0139	0,3099	instrument
rs2076603	1	17319011	G	A	0,0002	39,3878	0,0212	0,0034	3,5E-10	-0,0437	0,0138	0,0016	outlier
rs2101435	2	233115158	G	C	0,0002	30,7024	0,0200	0,0036	3,0E-08	0,0021	0,0152	0,8890	instrument
rs2239647	14	33292743	A	C	0,0002	37,8344	0,0200	0,0032	7,7E-10	0,0087	0,0139	0,5322	instrument
rs2289292	16	30097630	T	C	0,0002	39,5260	-0,0216	0,0034	3,3E-10	-0,0050	0,0147	0,7337	instrument
rs2295654	14	101539197	T	C	0,0002	38,1179	-0,0231	0,0037	6,7E-10	-0,0017	0,0164	0,9167	instrument
rs2299383	7	103418846	T	C	0,0002	46,4574	0,0224	0,0033	9,4E-12	-0,0164	0,0135	0,2239	instrument
rs2346883	2	47015914	G	T	0,0002	46,9631	-0,0233	0,0034	7,2E-12	0,0049	0,0143	0,7334	instrument
rs2404324	7	99023461	G	A	0,0002	40,7167	-0,0285	0,0045	1,8E-10	0,0284	0,0181	0,1169	instrument
rs2436661	15	52145407	A	G	0,0002	36,1438	-0,0195	0,0032	1,8E-09	0,0071	0,0132	0,5930	instrument
rs2450444	10	93010383	A	G	0,0002	42,8628	-0,0221	0,0034	5,9E-11	-0,0068	0,0140	0,6243	instrument
rs2644135	1	201856256	C	G	0,0002	40,5637	0,0216	0,0034	1,9E-10	-0,0029	0,0141	0,8372	instrument
rs2708149	2	58951187	A	G	0,0003	50,6939	-0,0235	0,0033	1,1E-12	-0,0204	0,0136	0,1327	instrument
rs2727316	17	61961336	G	A	0,0002	29,9534	-0,0197	0,0036	4,4E-08	0,0244	0,0147	0,0969	instrument
rs286818	5	107433446	A	T	0,0004	75,2722	-0,0374	0,0043	4,1E-18	-0,0137	0,0175	0,4347	instrument
rs2953806	8	8914124	T	C	0,0004	68,3095	0,0270	0,0033	1,4E-16	-0,0234	0,0182	0,1983	instrument
rs2994320	1	243641247	G	A	0,0002	32,2848	-0,0232	0,0041	1,3E-08	0,0207	0,0171	0,2268	instrument
rs34409196	17	70703396	C	A	0,0002	34,4448	0,0211	0,0036	4,4E-09	-0,0195	0,0156	0,2092	instrument
rs34811474	4	25408838	A	G	0,0004	75,3937	-0,0331	0,0038	3,9E-18	-0,0124	0,0184	0,4983	instrument
rs35559811	13	58666728	G	A	0,0003	61,7632	-0,0301	0,0038	3,9E-15	0,0243	0,0156	0,1185	instrument
rs3734554	6	40360781	T	C	0,0002	38,0068	0,0216	0,0035	7,1E-10	0,0239	0,0147	0,1030	instrument
rs3784710	15	68072458	C	T	0,0003	57,2589	-0,0291	0,0038	3,8E-14	-0,0053	0,0157	0,7377	instrument
rs3810291	19	47569003	G	A	0,0003	66,1938	-0,0281	0,0035	4,1E-16	0,0090	0,0140	0,5217	instrument
rs3826408	17	7101292	T	C	0,0002	32,1032	0,0183	0,0032	1,5E-08	-0,0034	0,0134	0,7981	instrument
rs3845836	2	219504955	C	T	0,0002	42,5621	0,0212	0,0033	6,9E-11	-0,0253	0,0134	0,0589	instrument
rs3895907	1	75006027	A	G	0,0003	51,3510	0,0234	0,0033	7,8E-13	0,0045	0,0132	0,7321	instrument

rs41271299	6	19839415	T	C	0,0002	30,2937	-0,0402	0,0073	3,7E-08	-0,0089	0,0409	0,8277	instrument
rs4304660	10	22295747	G	A	0,0002	34,9750	0,0217	0,0037	3,3E-09	0,0379	0,0147	0,0100	outlier
rs4482463	2	205375909	C	A	0,0002	36,6869	0,0372	0,0061	1,4E-09	-0,0584	0,0253	0,0210	instrument
rs4680340	3	156861460	C	T	0,0002	30,2277	0,0204	0,0037	3,8E-08	-0,0203	0,0152	0,1822	instrument
rs4790841	17	1835482	T	C	0,0003	57,7290	-0,0340	0,0045	3,0E-14	0,0348	0,0190	0,0675	instrument
rs4794670	17	54927829	T	A	0,0002	30,6802	-0,0214	0,0039	3,0E-08	0,0053	0,0172	0,7575	instrument
rs4796225	17	34843839	G	A	0,0002	38,1179	-0,0200	0,0032	6,7E-10	-0,0046	0,0140	0,7425	instrument
rs4808944	19	19426613	C	A	0,0002	39,0246	-0,0276	0,0044	4,2E-10	0,0446	0,0178	0,0124	outlier
rs4821943	22	40722745	G	A	0,0002	35,5093	-0,0204	0,0034	2,5E-09	-0,0098	0,0138	0,4788	instrument
rs4842838	15	84582124	G	T	0,0004	74,2864	0,0278	0,0032	6,8E-18	-0,0121	0,0132	0,3566	instrument
rs4889606	16	31011183	G	A	0,0004	67,1245	-0,0274	0,0033	2,6E-16	-0,0139	0,0137	0,3132	instrument
rs539515	1	177889025	C	A	0,0008	161,7967	0,0505	0,0040	5,0E-37	0,0023	0,0169	0,8907	instrument
rs55654354	3	82643954	C	T	0,0002	35,7122	-0,0258	0,0043	2,3E-09	-0,0047	0,0178	0,7914	instrument
rs56282717	7	150657095	A	G	0,0002	35,3308	-0,0225	0,0038	2,8E-09	0,0101	0,0156	0,5163	instrument
rs61037750	11	43681372	A	G	0,0002	37,4418	0,0214	0,0035	9,5E-10	0,0245	0,0143	0,0868	instrument
rs61813324	1	156049877	T	C	0,0002	42,5230	0,0317	0,0049	7,0E-11	0,0294	0,0213	0,1665	instrument
rs61910820	11	134581277	G	A	0,0002	32,2280	0,0243	0,0043	1,4E-08	0,0197	0,0183	0,2808	instrument
rs62004866	15	74223118	G	C	0,0002	44,7289	0,0350	0,0052	2,3E-11	0,0321	0,0212	0,1295	instrument
rs62106258	2	417167	C	T	0,0008	145,2010	-0,0899	0,0075	2,1E-33	-0,0406	0,0360	0,2584	instrument
rs62131065	2	25568539	G	A	0,0002	33,3041	-0,0312	0,0054	7,9E-09	0,0035	0,0221	0,8731	instrument
rs6548237	2	621461	A	C	0,0007	137,8262	-0,0500	0,0043	8,4E-32	-0,0056	0,0220	0,8009	instrument
rs6585200	10	114768609	G	A	0,0002	45,1848	-0,0218	0,0032	1,8E-11	-0,0290	0,0132	0,0283	instrument
rs6606652	11	69499786	G	T	0,0002	36,0717	-0,0201	0,0033	1,9E-09	-0,0127	0,0137	0,3525	instrument
rs66922415	18	57848651	G	A	0,0009	163,8383	0,0485	0,0038	1,8E-37	-0,0052	0,0156	0,7406	instrument
rs6852276	4	145261999	G	A	0,0002	33,1427	0,0191	0,0033	8,6E-09	-0,0026	0,0139	0,8524	instrument
rs7045925	9	96475306	T	C	0,0002	39,1371	0,0231	0,0037	4,0E-10	-0,0088	0,0155	0,5676	instrument
rs7124681	11	47529947	A	C	0,0003	63,2336	0,0261	0,0033	1,8E-15	0,0054	0,0135	0,6876	instrument
rs72731237	9	34148743	A	G	0,0002	34,8332	-0,0309	0,0052	3,6E-09	-0,0010	0,0210	0,9633	instrument
rs72740699	5	27188818	C	G	0,0002	31,1919	-0,0202	0,0036	2,3E-08	0,0182	0,0151	0,2255	instrument
rs72755233	15	100692953	A	G	0,0004	72,3343	0,0432	0,0051	1,8E-17	-0,0402	0,0260	0,1224	instrument
rs72771049	16	19933511	T	G	0,0003	54,8038	-0,0342	0,0046	1,3E-13	-0,0393	0,0189	0,0376	instrument

rs72892910	6	50816887	T	G	0,0004	76,1772	0,0376	0,0043	2,6E-18	-0,0005	0,0173	0,9768	instrument
rs73052033	3	185828465	C	T	0,0004	70,5929	-0,0351	0,0042	4,4E-17	-0,0036	0,0174	0,8345	instrument
rs7459710	8	77222670	T	C	0,0002	36,1438	-0,0196	0,0033	1,8E-09	0,0124	0,0133	0,3491	instrument
rs7534091	1	118864616	G	A	0,0002	33,4890	0,0212	0,0037	7,2E-09	-0,0016	0,0151	0,9145	instrument
rs7795394	7	113560607	T	A	0,0002	33,1657	0,0192	0,0033	8,5E-09	-0,0150	0,0138	0,2766	instrument
rs879620	16	4015729	C	T	0,0002	34,4565	-0,0195	0,0033	4,4E-09	0,0027	0,0138	0,8430	instrument
rs925421	4	60253877	A	G	0,0002	34,2221	0,0218	0,0037	4,9E-09	0,0114	0,0154	0,4594	instrument
rs9688977	6	154336892	C	T	0,0002	31,2813	0,0257	0,0046	2,2E-08	-0,0167	0,0186	0,3685	instrument
rs9929088	16	29001460	A	C	0,0002	39,2373	0,0218	0,0035	3,8E-10	0,0093	0,0148	0,5294	instrument

Table S22. Single Nucleotide Polymorphisms (SNPs) used as instruments in the Mendelian randomization analyses for the effect of AFR on ER- breast cancer based on the genome-wide significance threshold P = 5E-08. R^2 represents the explained variance in the exposure. SNP-specific instrument strength is given by the respective F-statistic. Some of the genetic variants were identified as outliers in the iterative approach and therefore excluded from the analyses.

SNP	CHR	POS	EA	OA	R^2	F-stat	β_{AFR}	SE_{AFR}	P_{AFR}	β_{ER-}	SE_{ER-}	P_{ER-}	$variant_{ER-}$
rs10013032	4	120149419	C	T	0,0002	38,6880	0,0208	0,0033	5,0E-10	0,0137	0,0120	0,2563	instrument
rs10057598	5	64070812	C	T	0,0002	35,5451	0,0196	0,0033	2,5E-09	-0,0133	0,0116	0,2507	instrument
rs10182458	2	25150641	G	A	0,0010	192,9301	0,0449	0,0032	8,1E-44	-0,0535	0,0119	7,1E-06	outlier
rs10484759	6	127037042	T	C	0,0002	31,1472	0,0208	0,0037	2,4E-08	-0,0141	0,0137	0,3007	instrument
rs1048932	11	115044850	A	C	0,0002	32,5809	-0,0187	0,0033	1,1E-08	-0,0027	0,0116	0,8156	instrument
rs10504303	8	60859424	A	T	0,0002	33,6281	-0,0194	0,0034	6,7E-09	-0,0113	0,0124	0,3595	instrument
rs10761785	10	65318766	G	T	0,0002	42,7188	0,0212	0,0032	6,3E-11	0,0048	0,0112	0,6695	instrument
rs10797119	9	92202495	T	C	0,0002	33,4543	-0,0189	0,0033	7,3E-09	0,0324	0,0114	0,0046	outlier
rs10800938	1	190220059	A	G	0,0002	38,7627	0,0202	0,0032	4,8E-10	-0,0080	0,0118	0,4991	instrument
rs10916174	1	227804041	A	G	0,0002	35,8438	0,0266	0,0044	2,1E-09	-0,0098	0,0162	0,5448	instrument
rs10962549	9	16719445	T	C	0,0003	48,5107	0,0303	0,0043	3,3E-12	-0,0009	0,0160	0,9544	instrument
rs11030119	11	27728102	A	G	0,0003	61,7004	0,0274	0,0035	4,0E-15	-0,0109	0,0126	0,3841	instrument
rs11159247	14	77509872	A	G	0,0002	31,7642	0,0199	0,0035	1,7E-08	-0,0386	0,0124	0,0018	outlier
rs112566467	1	39562627	T	C	0,0002	31,8431	0,0225	0,0040	1,7E-08	0,0197	0,0141	0,1616	instrument
rs112726905	19	32822348	G	A	0,0002	29,8878	-0,0201	0,0037	4,6E-08	0,0075	0,0137	0,5820	instrument
rs112920189	7	75094545	A	C	0,0003	55,8900	-0,0243	0,0033	7,7E-14	-0,0152	0,0117	0,1931	instrument
rs113996403	2	95911816	C	G	0,0002	31,2925	0,0724	0,0129	2,2E-08	-0,0098	0,0455	0,8295	instrument

rs114938354	5	176732485	C	G	0,0002	40,6530	0,0642	0,0101	1,8E-10	0,0209	0,0367	0,5686 instrument
rs11642015	16	53802494	T	C	0,0020	380,6361	0,0641	0,0033	1,1E-84	-0,0781	0,0115	1,0E-11 outlier
rs11721374	4	8599520	A	G	0,0002	43,0200	0,0215	0,0033	5,4E-11	-0,0040	0,0125	0,7486 instrument
rs1173734	5	32766417	C	T	0,0002	31,3149	-0,0206	0,0037	2,2E-08	0,0139	0,0136	0,3074 instrument
rs11766944	7	1888051	A	G	0,0002	35,6405	-0,0250	0,0042	2,4E-09	-0,0263	0,0140	0,0611 instrument
rs117700741	19	8637832	T	C	0,0002	31,6403	0,0511	0,0091	1,9E-08	0,0225	0,0373	0,5462 instrument
rs11851616	14	104065600	A	G	0,0003	57,5620	0,0266	0,0035	3,3E-14	-0,0013	0,0123	0,9177 instrument
rs11881199	19	18837459	T	C	0,0002	36,8202	0,0197	0,0033	1,3E-09	0,0023	0,0119	0,8488 instrument
rs1207949	13	33008393	C	T	0,0002	45,8866	-0,0229	0,0034	1,3E-11	-0,0049	0,0119	0,6782 instrument
rs12140153	1	62579891	T	G	0,0002	30,2497	-0,0316	0,0057	3,8E-08	-0,0244	0,0216	0,2570 instrument
rs12233810	4	17859768	T	C	0,0002	36,0116	-0,0218	0,0036	2,0E-09	0,0176	0,0129	0,1733 instrument
rs12357890	10	99762693	A	G	0,0002	45,5215	-0,0222	0,0033	1,5E-11	-0,0143	0,0121	0,2372 instrument
rs12432754	14	94030357	A	G	0,0002	40,8444	-0,0216	0,0034	1,6E-10	-0,0044	0,0118	0,7064 instrument
rs12575252	11	8694073	C	G	0,0002	31,8093	-0,0193	0,0034	1,7E-08	-0,0043	0,0119	0,7167 instrument
rs12805133	11	66483265	G	A	0,0003	53,8750	0,0238	0,0032	2,1E-13	-0,0078	0,0116	0,5010 instrument
rs12926506	16	81722413	T	C	0,0002	32,0353	-0,0247	0,0044	1,5E-08	0,0197	0,0167	0,2384 instrument
rs13002946	2	100801959	A	T	0,0003	51,9547	-0,0265	0,0037	5,7E-13	0,0084	0,0131	0,5217 instrument
rs13107325	4	103188709	T	C	0,0004	70,8620	0,0520	0,0062	3,9E-17	-0,0064	0,0226	0,7760 instrument
rs13174863	5	139080745	G	A	0,0002	42,3276	0,0300	0,0046	7,7E-11	-0,0087	0,0178	0,6271 instrument
rs13434116	3	136353074	C	G	0,0003	51,1792	0,0271	0,0038	8,4E-13	-0,0156	0,0132	0,2372 instrument
rs150468996	1	1591869	C	T	0,0002	36,2280	-0,0215	0,0036	1,8E-09	0,0020	0,0178	0,9117 instrument
rs16942323	15	89383764	C	T	0,0002	35,6764	0,0603	0,0101	2,3E-09	-0,0056	0,0422	0,8945 instrument
rs17066856	18	58049656	C	T	0,0003	47,8997	-0,0391	0,0056	4,5E-12	0,0173	0,0195	0,3769 instrument
rs1708302	7	28198677	C	T	0,0003	63,7915	-0,0257	0,0032	1,4E-15	-0,0065	0,0113	0,5617 instrument
rs1715896	15	56828094	G	A	0,0002	38,3528	0,0203	0,0033	5,9E-10	0,0070	0,0119	0,5585 instrument
rs17167306	7	133529648	C	A	0,0002	29,7349	-0,0256	0,0047	4,9E-08	0,0265	0,0165	0,1090 instrument
rs17391694	1	78623626	T	C	0,0002	34,9869	0,0274	0,0046	3,3E-09	0,0157	0,0190	0,4083 instrument
rs17619973	3	114417675	G	A	0,0002	31,4045	-0,0345	0,0061	2,1E-08	-0,0320	0,0234	0,1704 instrument
rs17763953	14	79969194	C	T	0,0002	33,7209	0,0192	0,0033	6,4E-09	0,0165	0,0118	0,1616 instrument
rs17770336	9	28414625	T	C	0,0003	48,7199	0,0240	0,0034	3,0E-12	-0,0112	0,0122	0,3614 instrument
rs1881459	5	88841488	G	A	0,0003	56,3695	0,0251	0,0033	6,0E-14	-0,0167	0,0124	0,1777 instrument

rs1884897	20	6612832	A	G	0,0005	92,6589	-0,0323	0,0034	6,3E-22	0,0179	0,0121	0,1367	instrument
rs200126474	17	47112109	A	C	0,0002	40,2967	-0,0219	0,0034	2,2E-10	0,0013	0,0125	0,9143	instrument
rs205286	6	34551334	C	A	0,0004	75,4458	0,0299	0,0034	3,8E-18	-0,0108	0,0122	0,3764	instrument
rs2076603	1	17319011	G	A	0,0002	39,3878	0,0212	0,0034	3,5E-10	-0,0138	0,0118	0,2413	instrument
rs2101435	2	233115158	G	C	0,0002	30,7024	0,0200	0,0036	3,0E-08	-0,0206	0,0132	0,1207	instrument
rs2239647	14	33292743	A	C	0,0002	37,8344	0,0200	0,0032	7,7E-10	0,0093	0,0120	0,4379	instrument
rs2289292	16	30097630	T	C	0,0002	39,5260	-0,0216	0,0034	3,3E-10	-0,0096	0,0128	0,4534	instrument
rs2295654	14	101539197	T	C	0,0002	38,1179	-0,0231	0,0037	6,7E-10	0,0121	0,0138	0,3795	instrument
rs2299383	7	103418846	T	C	0,0002	46,4574	0,0224	0,0033	9,4E-12	-0,0175	0,0116	0,1315	instrument
rs2346883	2	47015914	G	T	0,0002	46,9631	-0,0233	0,0034	7,2E-12	0,0025	0,0123	0,8382	instrument
rs2404324	7	99023461	G	A	0,0002	40,7167	-0,0285	0,0045	1,8E-10	-0,0095	0,0155	0,5423	instrument
rs2436661	15	52145407	A	G	0,0002	36,1438	-0,0195	0,0032	1,8E-09	0,0010	0,0115	0,9298	instrument
rs2450444	10	93010383	A	G	0,0002	42,8628	-0,0221	0,0034	5,9E-11	-0,0066	0,0119	0,5774	instrument
rs2644135	1	201856256	C	G	0,0002	40,5637	0,0216	0,0034	1,9E-10	0,0005	0,0121	0,9674	instrument
rs2708149	2	58951187	A	G	0,0003	50,6939	-0,0235	0,0033	1,1E-12	0,0000	0,0115	0,9969	instrument
rs2727316	17	61961336	G	A	0,0002	29,9534	-0,0197	0,0036	4,4E-08	0,0069	0,0128	0,5927	instrument
rs286818	5	107433446	A	T	0,0004	75,2722	-0,0374	0,0043	4,1E-18	-0,0029	0,0150	0,8473	instrument
rs2953806	8	8914124	T	C	0,0004	68,3095	0,0270	0,0033	1,4E-16	-0,0329	0,0136	0,0158	instrument
rs2994320	1	243641247	G	A	0,0002	32,2848	-0,0232	0,0041	1,3E-08	-0,0258	0,0148	0,0810	instrument
rs34409196	17	70703396	C	A	0,0002	34,4448	0,0211	0,0036	4,4E-09	0,0071	0,0132	0,5909	instrument
rs34811474	4	25408838	A	G	0,0004	75,3937	-0,0331	0,0038	3,9E-18	-0,0217	0,0165	0,1869	instrument
rs35559811	13	58666728	G	A	0,0003	61,7632	-0,0301	0,0038	3,9E-15	-0,0033	0,0133	0,8017	instrument
rs3734554	6	40360781	T	C	0,0002	38,0068	0,0216	0,0035	7,1E-10	0,0033	0,0133	0,8054	instrument
rs3784710	15	68072458	C	T	0,0003	57,2589	-0,0291	0,0038	3,8E-14	0,0039	0,0135	0,7742	instrument
rs3810291	19	47569003	G	A	0,0003	66,1938	-0,0281	0,0035	4,1E-16	0,0397	0,0123	0,0012	outlier
rs3826408	17	7101292	T	C	0,0002	32,1032	0,0183	0,0032	1,5E-08	0,0226	0,0114	0,0477	instrument
rs3845836	2	219504955	C	T	0,0002	42,5621	0,0212	0,0033	6,9E-11	0,0106	0,0117	0,3639	instrument
rs3895907	1	75006027	A	G	0,0003	51,3510	0,0234	0,0033	7,8E-13	-0,0124	0,0113	0,2734	instrument
rs41271299	6	19839415	T	C	0,0002	30,2937	-0,0402	0,0073	3,7E-08	-0,0745	0,0391	0,0565	instrument
rs4304660	10	22295747	G	A	0,0002	34,9750	0,0217	0,0037	3,3E-09	-0,0339	0,0130	0,0091	instrument
rs4482463	2	205375909	C	A	0,0002	36,6869	0,0372	0,0061	1,4E-09	-0,0435	0,0218	0,0461	instrument

rs4680340	3	156861460	C	T	0,0002	30,2277	0,0204	0,0037	3,8E-08	-0,0039	0,0131	0,7640	instrument
rs4790841	17	1835482	T	C	0,0003	57,7290	-0,0340	0,0045	3,0E-14	0,0107	0,0162	0,5104	instrument
rs4794670	17	54927829	T	A	0,0002	30,6802	-0,0214	0,0039	3,0E-08	0,0083	0,0157	0,5964	instrument
rs4796225	17	34843839	G	A	0,0002	38,1179	-0,0200	0,0032	6,7E-10	-0,0002	0,0127	0,9867	instrument
rs4808944	19	19426613	C	A	0,0002	39,0246	-0,0276	0,0044	4,2E-10	0,0435	0,0153	0,0045	instrument
rs4821943	22	40722745	G	A	0,0002	35,5093	-0,0204	0,0034	2,5E-09	0,0446	0,0117	0,0001	outlier
rs4842838	15	84582124	G	T	0,0004	74,2864	0,0278	0,0032	6,8E-18	-0,0042	0,0112	0,7118	instrument
rs4889606	16	31011183	G	A	0,0004	67,1245	-0,0274	0,0033	2,6E-16	0,0119	0,0118	0,3120	instrument
rs539515	1	177889025	C	A	0,0008	161,7967	0,0505	0,0040	5,0E-37	-0,0164	0,0144	0,2565	instrument
rs55654354	3	82643954	C	T	0,0002	35,7122	-0,0258	0,0043	2,3E-09	0,0131	0,0153	0,3918	instrument
rs56282717	7	150657095	A	G	0,0002	35,3308	-0,0225	0,0038	2,8E-09	0,0090	0,0134	0,5020	instrument
rs61037750	11	43681372	A	G	0,0002	37,4418	0,0214	0,0035	9,5E-10	-0,0052	0,0123	0,6747	instrument
rs61813324	1	156049877	T	C	0,0002	42,5230	0,0317	0,0049	7,0E-11	-0,0033	0,0188	0,8615	instrument
rs61910820	11	134581277	G	A	0,0002	32,2280	0,0243	0,0043	1,4E-08	-0,0011	0,0163	0,9465	instrument
rs62004866	15	74223118	G	C	0,0002	44,7289	0,0350	0,0052	2,3E-11	0,0070	0,0185	0,7046	instrument
rs62106258	2	417167	C	T	0,0008	145,2010	-0,0899	0,0075	2,1E-33	0,0126	0,0326	0,6995	instrument
rs62131065	2	25568539	G	A	0,0002	33,3041	-0,0312	0,0054	7,9E-09	-0,0091	0,0189	0,6318	instrument
rs6548237	2	621461	A	C	0,0007	137,8262	-0,0500	0,0043	8,4E-32	0,0591	0,0189	0,0018	outlier
rs6585200	10	114768609	G	A	0,0002	45,1848	-0,0218	0,0032	1,8E-11	0,0621	0,0113	3,5E-08	outlier
rs6606652	11	69499786	G	T	0,0002	36,0717	-0,0201	0,0033	1,9E-09	-0,0124	0,0121	0,3040	instrument
rs66922415	18	57848651	G	A	0,0009	163,8383	0,0485	0,0038	1,8E-37	-0,0248	0,0133	0,0628	instrument
rs6852276	4	145261999	G	A	0,0002	33,1427	0,0191	0,0033	8,6E-09	-0,0087	0,0123	0,4761	instrument
rs7045925	9	96475306	T	C	0,0002	39,1371	0,0231	0,0037	4,0E-10	-0,0027	0,0131	0,8357	instrument
rs7124681	11	47529947	A	C	0,0003	63,2336	0,0261	0,0033	1,8E-15	-0,0101	0,0115	0,3826	instrument
rs72731237	9	34148743	A	G	0,0002	34,8332	-0,0309	0,0052	3,6E-09	-0,0125	0,0176	0,4767	instrument
rs72740699	5	27188818	C	G	0,0002	31,1919	-0,0202	0,0036	2,3E-08	-0,0050	0,0132	0,7024	instrument
rs72755233	15	100692953	A	G	0,0004	72,3343	0,0432	0,0051	1,8E-17	-0,0090	0,0234	0,7019	instrument
rs72771049	16	19933511	T	G	0,0003	54,8038	-0,0342	0,0046	1,3E-13	0,0367	0,0164	0,0249	instrument
rs72892910	6	50816887	T	G	0,0004	76,1772	0,0376	0,0043	2,6E-18	-0,0363	0,0149	0,0147	instrument
rs73052033	3	185828465	C	T	0,0004	70,5929	-0,0351	0,0042	4,4E-17	-0,0308	0,0152	0,0428	outlier
rs7459710	8	77222670	T	C	0,0002	36,1438	-0,0196	0,0033	1,8E-09	0,0263	0,0113	0,0202	instrument

rs7534091	1	118864616	G	A	0,0002	33,4890	0,0212	0,0037	7,2E-09	-0,0153	0,0131	0,2427	instrument
rs7795394	7	113560607	T	A	0,0002	33,1657	0,0192	0,0033	8,5E-09	0,0134	0,0121	0,2692	instrument
rs879620	16	4015729	C	T	0,0002	34,4565	-0,0195	0,0033	4,4E-09	0,0021	0,0120	0,8603	instrument
rs925421	4	60253877	A	G	0,0002	34,2221	0,0218	0,0037	4,9E-09	0,0054	0,0135	0,6902	instrument
rs9688977	6	154336892	C	T	0,0002	31,2813	0,0257	0,0046	2,2E-08	-0,0024	0,0162	0,8804	instrument
rs9929088	16	29001460	A	C	0,0002	39,2373	0,0218	0,0035	3,8E-10	-0,0073	0,0128	0,5679	instrument

Table S23. Single Nucleotide Polymorphisms (SNPs) used as instruments in the Mendelian randomization analyses for the effect of AFR on ER+ breast cancer based on the genome-wide significance threshold P = 5E-08. R^2 represents the explained variance in the exposure. SNP-specific instrument strength is given by the respective F-statistic. Some of the genetic variants were identified as outliers in the iterative approach and therefore excluded from the analyses.

SNP	CHR	POS	EA	OA	R^2	F-stat	β_{AFR}	SE_{AFR}	P_{AFR}	β_{ER+}	SE_{ER+}	P_{ER+}	$variant_{ER+}$
rs10013032	4	120149419	C	T	0,0002	38,6880	0,0208	0,0033	5,0E-10	-0,0134	0,0079	0,0891	instrument
rs10057598	5	64070812	C	T	0,0002	35,5451	0,0196	0,0033	2,5E-09	-0,0115	0,0076	0,1305	instrument
rs10182458	2	25150641	G	A	0,0010	192,9301	0,0449	0,0032	8,1E-44	-0,0329	0,0079	2,9E-05	outlier
rs10484759	6	127037042	T	C	0,0002	31,1472	0,0208	0,0037	2,4E-08	0,0034	0,0089	0,7005	instrument
rs1048932	11	115044850	A	C	0,0002	32,5809	-0,0187	0,0033	1,1E-08	0,0052	0,0076	0,4929	instrument
rs10504303	8	60859424	A	T	0,0002	33,6281	-0,0194	0,0034	6,7E-09	0,0001	0,0082	0,9866	instrument
rs10761785	10	65318766	G	T	0,0002	42,7188	0,0212	0,0032	6,3E-11	0,0296	0,0074	0,0001	outlier
rs10797119	9	92202495	T	C	0,0002	33,4543	-0,0189	0,0033	7,3E-09	0,0008	0,0075	0,9128	instrument
rs10800938	1	190220059	A	G	0,0002	38,7627	0,0202	0,0032	4,8E-10	-0,0056	0,0078	0,4703	instrument
rs10916174	1	227804041	A	G	0,0002	35,8438	0,0266	0,0044	2,1E-09	-0,0047	0,0105	0,6536	instrument
rs10962549	9	16719445	T	C	0,0003	48,5107	0,0303	0,0043	3,3E-12	0,0180	0,0107	0,0921	instrument
rs11030119	11	27728102	A	G	0,0003	61,7004	0,0274	0,0035	4,0E-15	-0,0036	0,0082	0,6647	instrument
rs11159247	14	77509872	A	G	0,0002	31,7642	0,0199	0,0035	1,7E-08	-0,0277	0,0081	0,0006	outlier
rs112566467	1	39562627	T	C	0,0002	31,8431	0,0225	0,0040	1,7E-08	0,0043	0,0094	0,6483	instrument
rs112726905	19	32822348	G	A	0,0002	29,8878	-0,0201	0,0037	4,6E-08	-0,0090	0,0091	0,3199	instrument
rs112920189	7	75094545	A	C	0,0003	55,8900	-0,0243	0,0033	7,7E-14	-0,0059	0,0076	0,4368	instrument
rs113996403	2	95911816	C	G	0,0002	31,2925	0,0724	0,0129	2,2E-08	-0,0075	0,0286	0,7934	instrument
rs114938354	5	176732485	C	G	0,0002	40,6530	0,0642	0,0101	1,8E-10	0,0307	0,0237	0,1947	instrument
rs11642015	16	53802494	T	C	0,0020	380,6361	0,0641	0,0033	1,1E-84	-0,0592	0,0075	2,5E-15	outlier
rs11721374	4	8599520	A	G	0,0002	43,0200	0,0215	0,0033	5,4E-11	-0,0081	0,0081	0,3166	instrument

rs1173734	5	32766417	C	T	0,0002	31,3149	-0,0206	0,0037	2,2E-08	-0,0008	0,0091	0,9290	instrument
rs11766944	7	1888051	A	G	0,0002	35,6405	-0,0250	0,0042	2,4E-09	0,0110	0,0092	0,2306	instrument
rs117700741	19	8637832	T	C	0,0002	31,6403	0,0511	0,0091	1,9E-08	-0,0276	0,0241	0,2520	instrument
rs11851616	14	104065600	A	G	0,0003	57,5620	0,0266	0,0035	3,3E-14	-0,0036	0,0080	0,6533	instrument
rs11881199	19	18837459	T	C	0,0002	36,8202	0,0197	0,0033	1,3E-09	0,0168	0,0078	0,0308	instrument
rs1207949	13	33008393	C	T	0,0002	45,8866	-0,0229	0,0034	1,3E-11	0,0128	0,0077	0,0976	instrument
rs12140153	1	62579891	T	G	0,0002	30,2497	-0,0316	0,0057	3,8E-08	0,0286	0,0139	0,0391	instrument
rs12233810	4	17859768	T	C	0,0002	36,0116	-0,0218	0,0036	2,0E-09	0,0077	0,0085	0,3658	instrument
rs12357890	10	99762693	A	G	0,0002	45,5215	-0,0222	0,0033	1,5E-11	0,0029	0,0080	0,7146	instrument
rs12432754	14	94030357	A	G	0,0002	40,8444	-0,0216	0,0034	1,6E-10	0,0060	0,0077	0,4376	instrument
rs12575252	11	8694073	C	G	0,0002	31,8093	-0,0193	0,0034	1,7E-08	0,0006	0,0078	0,9382	instrument
rs12805133	11	66483265	G	A	0,0003	53,8750	0,0238	0,0032	2,1E-13	-0,0043	0,0076	0,5694	instrument
rs12926506	16	81722413	T	C	0,0002	32,0353	-0,0247	0,0044	1,5E-08	0,0174	0,0109	0,1109	instrument
rs13002946	2	100801959	A	T	0,0003	51,9547	-0,0265	0,0037	5,7E-13	0,0055	0,0086	0,5241	instrument
rs13107325	4	103188709	T	C	0,0004	70,8620	0,0520	0,0062	3,9E-17	0,0201	0,0149	0,1779	instrument
rs13174863	5	139080745	G	A	0,0002	42,3276	0,0300	0,0046	7,7E-11	0,0306	0,0116	0,0084	outlier
rs13434116	3	136353074	C	G	0,0003	51,1792	0,0271	0,0038	8,4E-13	0,0076	0,0086	0,3779	instrument
rs150468996	1	1591869	C	T	0,0002	36,2280	-0,0215	0,0036	1,8E-09	-0,0033	0,0112	0,7703	instrument
rs16942323	15	89383764	C	T	0,0002	35,6764	0,0603	0,0101	2,3E-09	0,0373	0,0270	0,1680	instrument
rs17066856	18	58049656	C	T	0,0003	47,8997	-0,0391	0,0056	4,5E-12	0,0074	0,0127	0,5626	instrument
rs1708302	7	28198677	C	T	0,0003	63,7915	-0,0257	0,0032	1,4E-15	-0,0333	0,0074	6,2E-06	outlier
rs1715896	15	56828094	G	A	0,0002	38,3528	0,0203	0,0033	5,9E-10	0,0107	0,0078	0,1701	instrument
rs17167306	7	133529648	C	A	0,0002	29,7349	-0,0256	0,0047	4,9E-08	0,0241	0,0109	0,0268	instrument
rs17391694	1	78623626	T	C	0,0002	34,9869	0,0274	0,0046	3,3E-09	0,0072	0,0124	0,5600	instrument
rs17619973	3	114417675	G	A	0,0002	31,4045	-0,0345	0,0061	2,1E-08	-0,0038	0,0153	0,8037	instrument
rs17763953	14	79969194	C	T	0,0002	33,7209	0,0192	0,0033	6,4E-09	0,0112	0,0078	0,1505	instrument
rs17770336	9	28414625	T	C	0,0003	48,7199	0,0240	0,0034	3,0E-12	-0,0010	0,0080	0,9003	instrument
rs1881459	5	88841488	G	A	0,0003	56,3695	0,0251	0,0033	6,0E-14	-0,0124	0,0082	0,1280	instrument
rs1884897	20	6612832	A	G	0,0005	92,6589	-0,0323	0,0034	6,3E-22	0,0046	0,0079	0,5612	instrument
rs200126474	17	47112109	A	C	0,0002	40,2967	-0,0219	0,0034	2,2E-10	-0,0054	0,0082	0,5110	instrument
rs205286	6	34551334	C	A	0,0004	75,4458	0,0299	0,0034	3,8E-18	-0,0051	0,0079	0,5179	instrument

rs2076603	1	17319011	G	A	0,0002	39,3878	0,0212	0,0034	3,5E-10	-0,0093	0,0077	0,2293	instrument
rs2101435	2	233115158	G	C	0,0002	30,7024	0,0200	0,0036	3,0E-08	-0,0192	0,0088	0,0284	instrument
rs2239647	14	33292743	A	C	0,0002	37,8344	0,0200	0,0032	7,7E-10	-0,0039	0,0079	0,6259	instrument
rs2289292	16	30097630	T	C	0,0002	39,5260	-0,0216	0,0034	3,3E-10	-0,0006	0,0084	0,9404	instrument
rs2295654	14	101539197	T	C	0,0002	38,1179	-0,0231	0,0037	6,7E-10	-0,0057	0,0092	0,5360	instrument
rs2299383	7	103418846	T	C	0,0002	46,4574	0,0224	0,0033	9,4E-12	-0,0079	0,0076	0,3020	instrument
rs2346883	2	47015914	G	T	0,0002	46,9631	-0,0233	0,0034	7,2E-12	-0,0067	0,0080	0,4073	instrument
rs2404324	7	99023461	G	A	0,0002	40,7167	-0,0285	0,0045	1,8E-10	0,0043	0,0101	0,6676	instrument
rs2436661	15	52145407	A	G	0,0002	36,1438	-0,0195	0,0032	1,8E-09	0,0143	0,0075	0,0571	instrument
rs2450444	10	93010383	A	G	0,0002	42,8628	-0,0221	0,0034	5,9E-11	0,0031	0,0078	0,6905	instrument
rs2644135	1	201856256	C	G	0,0002	40,5637	0,0216	0,0034	1,9E-10	-0,0112	0,0079	0,1576	instrument
rs2708149	2	58951187	A	G	0,0003	50,6939	-0,0235	0,0033	1,1E-12	-0,0014	0,0076	0,8486	instrument
rs2727316	17	61961336	G	A	0,0002	29,9534	-0,0197	0,0036	4,4E-08	0,0130	0,0083	0,1183	instrument
rs286818	5	107433446	A	T	0,0004	75,2722	-0,0374	0,0043	4,1E-18	-0,0013	0,0098	0,8945	instrument
rs2953806	8	8914124	T	C	0,0004	68,3095	0,0270	0,0033	1,4E-16	-0,0213	0,0091	0,0195	instrument
rs2994320	1	243641247	G	A	0,0002	32,2848	-0,0232	0,0041	1,3E-08	0,0040	0,0096	0,6758	instrument
rs34409196	17	70703396	C	A	0,0002	34,4448	0,0211	0,0036	4,4E-09	0,0040	0,0087	0,6431	instrument
rs34811474	4	25408838	A	G	0,0004	75,3937	-0,0331	0,0038	3,9E-18	0,0080	0,0106	0,4495	instrument
rs35559811	13	58666728	G	A	0,0003	61,7632	-0,0301	0,0038	3,9E-15	-0,0165	0,0087	0,0583	instrument
rs3734554	6	40360781	T	C	0,0002	38,0068	0,0216	0,0035	7,1E-10	0,0003	0,0089	0,9701	instrument
rs3784710	15	68072458	C	T	0,0003	57,2589	-0,0291	0,0038	3,8E-14	0,0082	0,0088	0,3505	instrument
rs3810291	19	47569003	G	A	0,0003	66,1938	-0,0281	0,0035	4,1E-16	0,0188	0,0079	0,0176	instrument
rs3826408	17	7101292	T	C	0,0002	32,1032	0,0183	0,0032	1,5E-08	0,0170	0,0075	0,0237	outlier
rs3845836	2	219504955	C	T	0,0002	42,5621	0,0212	0,0033	6,9E-11	0,0065	0,0076	0,3893	instrument
rs3895907	1	75006027	A	G	0,0003	51,3510	0,0234	0,0033	7,8E-13	-0,0121	0,0074	0,1044	instrument
rs41271299	6	19839415	T	C	0,0002	30,2937	-0,0402	0,0073	3,7E-08	-0,0190	0,0251	0,4482	instrument
rs4304660	10	22295747	G	A	0,0002	34,9750	0,0217	0,0037	3,3E-09	0,0269	0,0083	0,0012	outlier
rs4482463	2	205375909	C	A	0,0002	36,6869	0,0372	0,0061	1,4E-09	-0,0181	0,0142	0,2038	instrument
rs4680340	3	156861460	C	T	0,0002	30,2277	0,0204	0,0037	3,8E-08	-0,0114	0,0086	0,1830	instrument
rs4790841	17	1835482	T	C	0,0003	57,7290	-0,0340	0,0045	3,0E-14	0,0148	0,0106	0,1627	instrument
rs4794670	17	54927829	T	A	0,0002	30,6802	-0,0214	0,0039	3,0E-08	0,0008	0,0105	0,9388	instrument

rs4796225	17	34843839	G	A	0,0002	38,1179	-0,0200	0,0032	6,7E-10	-0,0166	0,0085	0,0495	instrument
rs4808944	19	19426613	C	A	0,0002	39,0246	-0,0276	0,0044	4,2E-10	0,0300	0,0101	0,0030	outlier
rs4821943	22	40722745	G	A	0,0002	35,5093	-0,0204	0,0034	2,5E-09	0,0281	0,0077	0,0003	outlier
rs4842838	15	84582124	G	T	0,0004	74,2864	0,0278	0,0032	6,8E-18	0,0085	0,0074	0,2493	instrument
rs4889606	16	31011183	G	A	0,0004	67,1245	-0,0274	0,0033	2,6E-16	0,0034	0,0077	0,6566	instrument
rs539515	1	177889025	C	A	0,0008	161,7967	0,0505	0,0040	5,0E-37	-0,0254	0,0094	0,0070	instrument
rs55654354	3	82643954	C	T	0,0002	35,7122	-0,0258	0,0043	2,3E-09	0,0018	0,0101	0,8600	instrument
rs56282717	7	150657095	A	G	0,0002	35,3308	-0,0225	0,0038	2,8E-09	0,0199	0,0088	0,0230	instrument
rs61037750	11	43681372	A	G	0,0002	37,4418	0,0214	0,0035	9,5E-10	0,0090	0,0080	0,2641	instrument
rs61813324	1	156049877	T	C	0,0002	42,5230	0,0317	0,0049	7,0E-11	-0,0271	0,0122	0,0265	instrument
rs61910820	11	134581277	G	A	0,0002	32,2280	0,0243	0,0043	1,4E-08	-0,0041	0,0108	0,7033	instrument
rs62004866	15	74223118	G	C	0,0002	44,7289	0,0350	0,0052	2,3E-11	0,0267	0,0120	0,0266	outlier
rs62106258	2	417167	C	T	0,0008	145,2010	-0,0899	0,0075	2,1E-33	0,0324	0,0214	0,1305	instrument
rs62131065	2	25568539	G	A	0,0002	33,3041	-0,0312	0,0054	7,9E-09	0,0153	0,0123	0,2143	instrument
rs6548237	2	621461	A	C	0,0007	137,8262	-0,0500	0,0043	8,4E-32	0,0193	0,0125	0,1223	instrument
rs6585200	10	114768609	G	A	0,0002	45,1848	-0,0218	0,0032	1,8E-11	0,0324	0,0074	1,1E-05	outlier
rs6606652	11	69499786	G	T	0,0002	36,0717	-0,0201	0,0033	1,9E-09	-0,0292	0,0078	0,0002	outlier
rs66922415	18	57848651	G	A	0,0009	163,8383	0,0485	0,0038	1,8E-37	-0,0137	0,0087	0,1168	instrument
rs6852276	4	145261999	G	A	0,0002	33,1427	0,0191	0,0033	8,6E-09	0,0062	0,0081	0,4440	instrument
rs7045925	9	96475306	T	C	0,0002	39,1371	0,0231	0,0037	4,0E-10	-0,0034	0,0086	0,6946	instrument
rs7124681	11	47529947	A	C	0,0003	63,2336	0,0261	0,0033	1,8E-15	-0,0102	0,0076	0,1762	instrument
rs72731237	9	34148743	A	G	0,0002	34,8332	-0,0309	0,0052	3,6E-09	-0,0042	0,0114	0,7086	instrument
rs72740699	5	27188818	C	G	0,0002	31,1919	-0,0202	0,0036	2,3E-08	0,0087	0,0087	0,3181	instrument
rs72755233	15	100692953	A	G	0,0004	72,3343	0,0432	0,0051	1,8E-17	0,0136	0,0152	0,3710	instrument
rs72771049	16	19933511	T	G	0,0003	54,8038	-0,0342	0,0046	1,3E-13	0,0009	0,0108	0,9309	instrument
rs72892910	6	50816887	T	G	0,0004	76,1772	0,0376	0,0043	2,6E-18	0,0192	0,0097	0,0472	outlier
rs73052033	3	185828465	C	T	0,0004	70,5929	-0,0351	0,0042	4,4E-17	-0,0058	0,0099	0,5576	instrument
rs7459710	8	77222670	T	C	0,0002	36,1438	-0,0196	0,0033	1,8E-09	0,0149	0,0074	0,0447	instrument
rs7534091	1	118864616	G	A	0,0002	33,4890	0,0212	0,0037	7,2E-09	-0,0160	0,0086	0,0635	instrument
rs7795394	7	113560607	T	A	0,0002	33,1657	0,0192	0,0033	8,5E-09	0,0098	0,0079	0,2135	instrument
rs879620	16	4015729	C	T	0,0002	34,4565	-0,0195	0,0033	4,4E-09	-0,0059	0,0079	0,4540	instrument

rs925421	4	60253877	A	G	0,0002	34,2221	0,0218	0,0037	4,9E-09	0,0162	0,0089	0,0695	instrument
rs9688977	6	154336892	C	T	0,0002	31,2813	0,0257	0,0046	2,2E-08	-0,0205	0,0106	0,0526	instrument
rs9929088	16	29001460	A	C	0,0002	39,2373	0,0218	0,0035	3,8E-10	-0,0011	0,0084	0,8941	instrument

Table S24. Single Nucleotide Polymorphisms (SNPs) used as instruments in the Mendelian randomization analyses for the effect of AFR on clear cell ovarian cancer based on the genome-wide significance threshold P = 5E-08. R^2 represents the explained variance in the exposure. SNP-specific instrument strength is given by the respective F-statistic. Some of the genetic variants were identified as outliers in the iterative approach and therefore excluded from the analyses.

SNP	CHR	POS	EA	OA	R^2	F-stat	β_{AFR}	SE_{AFR}	P_{AFR}	$\beta_{Clear\ cell}$	$SE_{Clear\ cell}$	$P_{Clear\ cell}$	$variant_{Clear\ cell}$
rs10013032	4	120149419	C	T	0,0002	38,6880	0,0208	0,0033	5,0E-10	0,0385	0,0418	0,3571	instrument
rs10057598	5	64070812	C	T	0,0002	35,5451	0,0196	0,0033	2,5E-09	-0,0377	0,0410	0,3577	instrument
rs10182458	2	25150641	G	A	0,0010	192,9301	0,0449	0,0032	8,1E-44	-0,0220	0,0411	0,5922	instrument
rs10484759	6	127037042	T	C	0,0002	31,1472	0,0208	0,0037	2,4E-08	0,0218	0,0472	0,6443	instrument
rs1048932	11	115044850	A	C	0,0002	32,5809	-0,0187	0,0033	1,1E-08	0,0042	0,0409	0,9181	instrument
rs10504303	8	60859424	A	T	0,0002	33,6281	-0,0194	0,0034	6,7E-09	0,0377	0,0421	0,3714	instrument
rs10761785	10	65318766	G	T	0,0002	42,7188	0,0212	0,0032	6,3E-11	-0,0120	0,0400	0,7654	instrument
rs10797119	9	92202495	T	C	0,0002	33,4543	-0,0189	0,0033	7,3E-09	-0,0517	0,0405	0,2022	instrument
rs10800938	1	190220059	A	G	0,0002	38,7627	0,0202	0,0032	4,8E-10	0,0555	0,0410	0,1754	instrument
rs10916174	1	227804041	A	G	0,0002	35,8438	0,0266	0,0044	2,1E-09	-0,0082	0,0568	0,8849	instrument
rs10962549	9	16719445	T	C	0,0003	48,5107	0,0303	0,0043	3,3E-12	-0,0365	0,0559	0,5126	instrument
rs11030119	11	27728102	A	G	0,0003	61,7004	0,0274	0,0035	4,0E-15	0,0075	0,0440	0,8650	instrument
rs11159247	14	77509872	A	G	0,0002	31,7642	0,0199	0,0035	1,7E-08	0,0457	0,0433	0,2913	instrument
rs112566467	1	39562627	T	C	0,0002	31,8431	0,0225	0,0040	1,7E-08	0,0018	0,0500	0,9713	instrument
rs112726905	19	32822348	G	A	0,0002	29,8878	-0,0201	0,0037	4,6E-08	-0,0984	0,0473	0,0375	instrument
rs112920189	7	75094545	A	C	0,0003	55,8900	-0,0243	0,0033	7,7E-14	-0,0313	0,0412	0,4472	instrument
rs113996403	2	95911816	C	G	0,0002	31,2925	0,0724	0,0129	2,2E-08	0,0751	0,1489	0,6138	instrument
rs114938354	5	176732485	C	G	0,0002	40,6530	0,0642	0,0101	1,8E-10	-0,2025	0,1333	0,1288	instrument
rs11642015	16	53802494	T	C	0,0020	380,6361	0,0641	0,0033	1,1E-84	0,0745	0,0403	0,0642	instrument
rs11721374	4	8599520	A	G	0,0002	43,0200	0,0215	0,0033	5,4E-11	-0,0489	0,0422	0,2464	instrument
rs1173734	5	32766417	C	T	0,0002	31,3149	-0,0206	0,0037	2,2E-08	0,0603	0,0463	0,1926	instrument
rs11766944	7	1888051	A	G	0,0002	35,6405	-0,0250	0,0042	2,4E-09	0,0416	0,0499	0,4053	instrument
rs117700741	19	8637832	T	C	0,0002	31,6403	0,0511	0,0091	1,9E-08	-0,2285	0,1296	0,0777	instrument

rs11851616	14	104065600	A	G	0,0003	57,5620	0,0266	0,0035	3,3E-14	0,0236	0,0436	0,5874	instrument
rs11881199	19	18837459	T	C	0,0002	36,8202	0,0197	0,0033	1,3E-09	0,0273	0,0409	0,5045	instrument
rs1207949	13	33008393	C	T	0,0002	45,8866	-0,0229	0,0034	1,3E-11	0,1052	0,0415	0,0112	instrument
rs12140153	1	62579891	T	G	0,0002	30,2497	-0,0316	0,0057	3,8E-08	0,0035	0,0734	0,9621	instrument
rs12233810	4	17859768	T	C	0,0002	36,0116	-0,0218	0,0036	2,0E-09	0,0261	0,0454	0,5661	instrument
rs12357890	10	99762693	A	G	0,0002	45,5215	-0,0222	0,0033	1,5E-11	-0,0608	0,0413	0,1407	instrument
rs12432754	14	94030357	A	G	0,0002	40,8444	-0,0216	0,0034	1,6E-10	-0,0013	0,0418	0,9758	instrument
rs12575252	11	8694073	C	G	0,0002	31,8093	-0,0193	0,0034	1,7E-08	0,0083	0,0424	0,8439	instrument
rs12805133	11	66483265	G	A	0,0003	53,8750	0,0238	0,0032	2,1E-13	-0,0437	0,0405	0,2806	instrument
rs12926506	16	81722413	T	C	0,0002	32,0353	-0,0247	0,0044	1,5E-08	0,0292	0,0586	0,6182	instrument
rs13002946	2	100801959	A	T	0,0003	51,9547	-0,0265	0,0037	5,7E-13	-0,0836	0,0465	0,0721	instrument
rs13107325	4	103188709	T	C	0,0004	70,8620	0,0520	0,0062	3,9E-17	-0,1122	0,0798	0,1598	instrument
rs13174863	5	139080745	G	A	0,0002	42,3276	0,0300	0,0046	7,7E-11	-0,0300	0,0596	0,6142	instrument
rs13434116	3	136353074	C	G	0,0003	51,1792	0,0271	0,0038	8,4E-13	0,0527	0,0466	0,2574	instrument
rs150468996	1	1591869	C	T	0,0002	36,2280	-0,0215	0,0036	1,8E-09	0,0277	0,0544	0,6112	instrument
rs16942323	15	89383764	C	T	0,0002	35,6764	0,0603	0,0101	2,3E-09	0,0478	0,1308	0,7149	instrument
rs17066856	18	58049656	C	T	0,0003	47,8997	-0,0391	0,0056	4,5E-12	0,0107	0,0692	0,8769	instrument
rs1708302	7	28198677	C	T	0,0003	63,7915	-0,0257	0,0032	1,4E-15	0,0047	0,0401	0,9056	instrument
rs1715896	15	56828094	G	A	0,0002	38,3528	0,0203	0,0033	5,9E-10	0,0096	0,0404	0,8113	instrument
rs17167306	7	133529648	C	A	0,0002	29,7349	-0,0256	0,0047	4,9E-08	-0,0600	0,0595	0,3134	instrument
rs17391694	1	78623626	T	C	0,0002	34,9869	0,0274	0,0046	3,3E-09	-0,0866	0,0633	0,1711	instrument
rs17619973	3	114417675	G	A	0,0002	31,4045	-0,0345	0,0061	2,1E-08	0,1015	0,0805	0,2073	instrument
rs17763953	14	79969194	C	T	0,0002	33,7209	0,0192	0,0033	6,4E-09	-0,0170	0,0415	0,6825	instrument
rs17770336	9	28414625	T	C	0,0003	48,7199	0,0240	0,0034	3,0E-12	0,0370	0,0432	0,3921	instrument
rs1881459	5	88841488	G	A	0,0003	56,3695	0,0251	0,0033	6,0E-14	0,0313	0,0426	0,4625	instrument
rs1884897	20	6612832	A	G	0,0005	92,6589	-0,0323	0,0034	6,3E-22	-0,0232	0,0420	0,5808	instrument
rs200126474	17	47112109	A	C	0,0002	40,2967	-0,0219	0,0034	2,2E-10	0,0307	0,0424	0,4689	instrument
rs205286	6	34551334	C	A	0,0004	75,4458	0,0299	0,0034	3,8E-18	-0,0336	0,0426	0,4308	instrument
rs2076603	1	17319011	G	A	0,0002	39,3878	0,0212	0,0034	3,5E-10	-0,0980	0,0423	0,0204	instrument
rs2101435	2	233115158	G	C	0,0002	30,7024	0,0200	0,0036	3,0E-08	-0,0072	0,0458	0,8752	instrument
rs2239647	14	33292743	A	C	0,0002	37,8344	0,0200	0,0032	7,7E-10	-0,0306	0,0422	0,4688	instrument

rs2289292	16	30097630	T	C	0,0002	39,5260	-0,0216	0,0034	3,3E-10	-0,0124	0,0446	0,7813	instrument
rs2295654	14	101539197	T	C	0,0002	38,1179	-0,0231	0,0037	6,7E-10	0,0148	0,0495	0,7650	instrument
rs2299383	7	103418846	T	C	0,0002	46,4574	0,0224	0,0033	9,4E-12	-0,0668	0,0409	0,1022	instrument
rs2346883	2	47015914	G	T	0,0002	46,9631	-0,0233	0,0034	7,2E-12	-0,0083	0,0434	0,8489	instrument
rs2404324	7	99023461	G	A	0,0002	40,7167	-0,0285	0,0045	1,8E-10	0,0051	0,0554	0,9261	instrument
rs2436661	15	52145407	A	G	0,0002	36,1438	-0,0195	0,0032	1,8E-09	0,0106	0,0399	0,7904	instrument
rs2450444	10	93010383	A	G	0,0002	42,8628	-0,0221	0,0034	5,9E-11	0,0156	0,0423	0,7118	instrument
rs2644135	1	201856256	C	G	0,0002	40,5637	0,0216	0,0034	1,9E-10	0,0572	0,0422	0,1761	instrument
rs2708149	2	58951187	A	G	0,0003	50,6939	-0,0235	0,0033	1,1E-12	0,0615	0,0410	0,1334	instrument
rs2727316	17	61961336	G	A	0,0002	29,9534	-0,0197	0,0036	4,4E-08	0,0326	0,0446	0,4650	instrument
rs286818	5	107433446	A	T	0,0004	75,2722	-0,0374	0,0043	4,1E-18	0,0463	0,0527	0,3792	instrument
rs2953806	8	8914124	T	C	0,0004	68,3095	0,0270	0,0033	1,4E-16	-0,0290	0,0561	0,6046	instrument
rs2994320	1	243641247	G	A	0,0002	32,2848	-0,0232	0,0041	1,3E-08	0,0376	0,0512	0,4631	instrument
rs34409196	17	70703396	C	A	0,0002	34,4448	0,0211	0,0036	4,4E-09	0,1090	0,0461	0,0179	instrument
rs34811474	4	25408838	A	G	0,0004	75,3937	-0,0331	0,0038	3,9E-18	0,0837	0,0546	0,1253	instrument
rs35559811	13	58666728	G	A	0,0003	61,7632	-0,0301	0,0038	3,9E-15	0,0560	0,0470	0,2340	instrument
rs3734554	6	40360781	T	C	0,0002	38,0068	0,0216	0,0035	7,1E-10	0,0095	0,0443	0,8295	instrument
rs3784710	15	68072458	C	T	0,0003	57,2589	-0,0291	0,0038	3,8E-14	-0,0526	0,0483	0,2762	instrument
rs3810291	19	47569003	G	A	0,0003	66,1938	-0,0281	0,0035	4,1E-16	-0,1163	0,0431	0,0069	outlier
rs3826408	17	7101292	T	C	0,0002	32,1032	0,0183	0,0032	1,5E-08	0,0596	0,0407	0,1435	instrument
rs3845836	2	219504955	C	T	0,0002	42,5621	0,0212	0,0033	6,9E-11	0,0102	0,0405	0,8017	instrument
rs3895907	1	75006027	A	G	0,0003	51,3510	0,0234	0,0033	7,8E-13	0,0191	0,0403	0,6361	instrument
rs41271299	6	19839415	T	C	0,0002	30,2937	-0,0402	0,0073	3,7E-08	0,0413	0,1210	0,7326	instrument
rs4304660	10	22295747	G	A	0,0002	34,9750	0,0217	0,0037	3,3E-09	0,0450	0,0442	0,3100	instrument
rs4482463	2	205375909	C	A	0,0002	36,6869	0,0372	0,0061	1,4E-09	-0,0674	0,0765	0,3782	instrument
rs4680340	3	156861460	C	T	0,0002	30,2277	0,0204	0,0037	3,8E-08	-0,0478	0,0466	0,3030	instrument
rs4790841	17	1835482	T	C	0,0003	57,7290	-0,0340	0,0045	3,0E-14	0,0976	0,0569	0,0864	instrument
rs4794670	17	54927829	T	A	0,0002	30,6802	-0,0214	0,0039	3,0E-08	-0,0141	0,0511	0,7834	instrument
rs4796225	17	34843839	G	A	0,0002	38,1179	-0,0200	0,0032	6,7E-10	0,0011	0,0418	0,9785	instrument
rs4808944	19	19426613	C	A	0,0002	39,0246	-0,0276	0,0044	4,2E-10	0,0381	0,0541	0,4816	instrument
rs4821943	22	40722745	G	A	0,0002	35,5093	-0,0204	0,0034	2,5E-09	-0,0309	0,0418	0,4588	instrument

rs4842838	15	84582124	G	T	0,0004	74,2864	0,0278	0,0032	6,8E-18	0,0206	0,0400	0,6070	instrument
rs4889606	16	31011183	G	A	0,0004	67,1245	-0,0274	0,0033	2,6E-16	0,0267	0,0417	0,5226	instrument
rs539515	1	177889025	C	A	0,0008	161,7967	0,0505	0,0040	5,0E-37	-0,0543	0,0514	0,2906	instrument
rs55654354	3	82643954	C	T	0,0002	35,7122	-0,0258	0,0043	2,3E-09	-0,0421	0,0541	0,4367	instrument
rs56282717	7	150657095	A	G	0,0002	35,3308	-0,0225	0,0038	2,8E-09	0,0170	0,0470	0,7181	instrument
rs61037750	11	43681372	A	G	0,0002	37,4418	0,0214	0,0035	9,5E-10	0,0397	0,0433	0,3588	instrument
rs61813324	1	156049877	T	C	0,0002	42,5230	0,0317	0,0049	7,0E-11	0,0826	0,0638	0,1953	instrument
rs61910820	11	134581277	G	A	0,0002	32,2280	0,0243	0,0043	1,4E-08	0,0141	0,0553	0,7989	instrument
rs62004866	15	74223118	G	C	0,0002	44,7289	0,0350	0,0052	2,3E-11	0,0169	0,0638	0,7915	instrument
rs62106258	2	417167	C	T	0,0008	145,2010	-0,0899	0,0075	2,1E-33	-0,1763	0,1121	0,1157	instrument
rs62131065	2	25568539	G	A	0,0002	33,3041	-0,0312	0,0054	7,9E-09	-0,0338	0,0677	0,6182	instrument
rs6548237	2	621461	A	C	0,0007	137,8262	-0,0500	0,0043	8,4E-32	-0,0722	0,0674	0,2843	instrument
rs6585200	10	114768609	G	A	0,0002	45,1848	-0,0218	0,0032	1,8E-11	0,0299	0,0399	0,4527	instrument
rs6606652	11	69499786	G	T	0,0002	36,0717	-0,0201	0,0033	1,9E-09	0,0606	0,0413	0,1425	instrument
rs66922415	18	57848651	G	A	0,0009	163,8383	0,0485	0,0038	1,8E-37	0,0224	0,0468	0,6320	instrument
rs6852276	4	145261999	G	A	0,0002	33,1427	0,0191	0,0033	8,6E-09	-0,0588	0,0419	0,1608	instrument
rs7045925	9	96475306	T	C	0,0002	39,1371	0,0231	0,0037	4,0E-10	-0,1008	0,0476	0,0342	instrument
rs7124681	11	47529947	A	C	0,0003	63,2336	0,0261	0,0033	1,8E-15	0,0089	0,0408	0,8269	instrument
rs72731237	9	34148743	A	G	0,0002	34,8332	-0,0309	0,0052	3,6E-09	-0,0376	0,0641	0,5572	instrument
rs72740699	5	27188818	C	G	0,0002	31,1919	-0,0202	0,0036	2,3E-08	0,0626	0,0453	0,1670	instrument
rs72755233	15	100692953	A	G	0,0004	72,3343	0,0432	0,0051	1,8E-17	-0,0926	0,0791	0,2423	instrument
rs72771049	16	19933511	T	G	0,0003	54,8038	-0,0342	0,0046	1,3E-13	-0,0130	0,0568	0,8194	instrument
rs72892910	6	50816887	T	G	0,0004	76,1772	0,0376	0,0043	2,6E-18	-0,0569	0,0533	0,2854	instrument
rs73052033	3	185828465	C	T	0,0004	70,5929	-0,0351	0,0042	4,4E-17	0,0109	0,0528	0,8372	instrument
rs7459710	8	77222670	T	C	0,0002	36,1438	-0,0196	0,0033	1,8E-09	0,0201	0,0401	0,6162	instrument
rs7534091	1	118864616	G	A	0,0002	33,4890	0,0212	0,0037	7,2E-09	-0,0214	0,0462	0,6436	instrument
rs7795394	7	113560607	T	A	0,0002	33,1657	0,0192	0,0033	8,5E-09	-0,0234	0,0419	0,5769	instrument
rs879620	16	4015729	C	T	0,0002	34,4565	-0,0195	0,0033	4,4E-09	-0,0227	0,0417	0,5867	instrument
rs925421	4	60253877	A	G	0,0002	34,2221	0,0218	0,0037	4,9E-09	0,0062	0,0463	0,8935	instrument
rs9688977	6	154336892	C	T	0,0002	31,2813	0,0257	0,0046	2,2E-08	-0,0206	0,0559	0,7127	instrument
rs9929088	16	29001460	A	C	0,0002	39,2373	0,0218	0,0035	3,8E-10	0,0754	0,0442	0,0878	instrument

Table S25. Single Nucleotide Polymorphisms (SNPs) used as instruments in the Mendelian randomization analyses for the effect of AFR on endometrioid ovarian cancer based on the genome-wide significance threshold P = 5E-08. R² represents the explained variance in the exposure. SNP-specific instrument strength is given by the respective F-statistic. Some of the genetic variants were identified as outliers in the iterative approach and therefore excluded from the analyses.

SNP	CHR	POS	EA	OA	R^2	F-stat	β_{AFR}	SE_{AFR}	P_{AFR}	$\beta_{Endometrioid}$	$SE_{Endometrioid}$	$P_{Endometrioid}$	$variant_{Endometrioid}$
rs10013032	4	120149419	C	T	0,0002	38,6880	0,0208	0,0033	5,0E-10	0,0102	0,0301	0,7357	instrument
rs10057598	5	64070812	C	T	0,0002	35,5451	0,0196	0,0033	2,5E-09	0,0512	0,0293	0,0812	instrument
rs10182458	2	25150641	G	A	0,0010	192,9301	0,0449	0,0032	8,1E-44	-0,0244	0,0295	0,4089	instrument
rs10484759	6	127037042	T	C	0,0002	31,1472	0,0208	0,0037	2,4E-08	-0,0041	0,0339	0,9026	instrument
rs1048932	11	115044850	A	C	0,0002	32,5809	-0,0187	0,0033	1,1E-08	0,0147	0,0292	0,6152	instrument
rs10504303	8	60859424	A	T	0,0002	33,6281	-0,0194	0,0034	6,7E-09	0,0291	0,0304	0,3377	instrument
rs10761785	10	65318766	G	T	0,0002	42,7188	0,0212	0,0032	6,3E-11	-0,0777	0,0287	0,0067	outlier
rs10797119	9	92202495	T	C	0,0002	33,4543	-0,0189	0,0033	7,3E-09	-0,0232	0,0290	0,4235	instrument
rs10800938	1	190220059	A	G	0,0002	38,7627	0,0202	0,0032	4,8E-10	0,0381	0,0294	0,1938	instrument
rs10916174	1	227804041	A	G	0,0002	35,8438	0,0266	0,0044	2,1E-09	-0,0532	0,0411	0,1956	instrument
rs10962549	9	16719445	T	C	0,0003	48,5107	0,0303	0,0043	3,3E-12	0,0575	0,0392	0,1446	instrument
rs11030119	11	27728102	A	G	0,0003	61,7004	0,0274	0,0035	4,0E-15	0,0161	0,0317	0,6124	instrument
rs11159247	14	77509872	A	G	0,0002	31,7642	0,0199	0,0035	1,7E-08	-0,0361	0,0312	0,2474	instrument
rs112566467	1	39562627	T	C	0,0002	31,8431	0,0225	0,0040	1,7E-08	-0,0179	0,0358	0,6170	instrument
rs112726905	19	32822348	G	A	0,0002	29,8878	-0,0201	0,0037	4,6E-08	0,0127	0,0334	0,7041	instrument
rs112920189	7	75094545	A	C	0,0003	55,8900	-0,0243	0,0033	7,7E-14	0,0430	0,0294	0,1440	instrument
rs113996403	2	95911816	C	G	0,0002	31,2925	0,0724	0,0129	2,2E-08	0,0790	0,1065	0,4581	instrument
rs114938354	5	176732485	C	G	0,0002	40,6530	0,0642	0,0101	1,8E-10	0,0695	0,0886	0,4329	instrument
rs11642015	16	53802494	T	C	0,0020	380,6361	0,0641	0,0033	1,1E-84	0,0182	0,0290	0,5307	instrument
rs11721374	4	8599520	A	G	0,0002	43,0200	0,0215	0,0033	5,4E-11	0,0258	0,0302	0,3922	instrument
rs1173734	5	32766417	C	T	0,0002	31,3149	-0,0206	0,0037	2,2E-08	-0,0127	0,0336	0,7047	instrument
rs11766944	7	1888051	A	G	0,0002	35,6405	-0,0250	0,0042	2,4E-09	0,0149	0,0358	0,6782	instrument
rs117700741	19	8637832	T	C	0,0002	31,6403	0,0511	0,0091	1,9E-08	0,0122	0,0871	0,8888	instrument
rs11851616	14	104065600	A	G	0,0003	57,5620	0,0266	0,0035	3,3E-14	0,0520	0,0310	0,0937	instrument
rs11881199	19	18837459	T	C	0,0002	36,8202	0,0197	0,0033	1,3E-09	-0,0128	0,0294	0,6625	instrument
rs1207949	13	33008393	C	T	0,0002	45,8866	-0,0229	0,0034	1,3E-11	0,0045	0,0300	0,8816	instrument
rs12140153	1	62579891	T	G	0,0002	30,2497	-0,0316	0,0057	3,8E-08	0,0568	0,0521	0,2761	instrument

rs12233810	4	17859768	T	C	0,0002	36,0116	-0,0218	0,0036	2,0E-09	-0,0380	0,0329	0,2474	instrument
rs12357890	10	99762693	A	G	0,0002	45,5215	-0,0222	0,0033	1,5E-11	0,0590	0,0296	0,0463	instrument
rs12432754	14	94030357	A	G	0,0002	40,8444	-0,0216	0,0034	1,6E-10	0,0560	0,0298	0,0596	instrument
rs12575252	11	8694073	C	G	0,0002	31,8093	-0,0193	0,0034	1,7E-08	-0,0126	0,0303	0,6771	instrument
rs12805133	11	66483265	G	A	0,0003	53,8750	0,0238	0,0032	2,1E-13	-0,0351	0,0290	0,2261	instrument
rs12926506	16	81722413	T	C	0,0002	32,0353	-0,0247	0,0044	1,5E-08	0,0462	0,0418	0,2681	instrument
rs13002946	2	100801959	A	T	0,0003	51,9547	-0,0265	0,0037	5,7E-13	-0,0236	0,0329	0,4729	instrument
rs13107325	4	103188709	T	C	0,0004	70,8620	0,0520	0,0062	3,9E-17	0,0022	0,0554	0,9682	instrument
rs13174863	5	139080745	G	A	0,0002	42,3276	0,0300	0,0046	7,7E-11	0,0336	0,0424	0,4284	instrument
rs13434116	3	136353074	C	G	0,0003	51,1792	0,0271	0,0038	8,4E-13	0,0093	0,0335	0,7818	instrument
rs150468996	1	1591869	C	T	0,0002	36,2280	-0,0215	0,0036	1,8E-09	0,0141	0,0394	0,7208	instrument
rs16942323	15	89383764	C	T	0,0002	35,6764	0,0603	0,0101	2,3E-09	-0,0527	0,0972	0,5880	instrument
rs17066856	18	58049656	C	T	0,0003	47,8997	-0,0391	0,0056	4,5E-12	-0,1131	0,0509	0,0263	instrument
rs1708302	7	28198677	C	T	0,0003	63,7915	-0,0257	0,0032	1,4E-15	0,0699	0,0286	0,0146	outlier
rs1715896	15	56828094	G	A	0,0002	38,3528	0,0203	0,0033	5,9E-10	-0,0375	0,0290	0,1957	instrument
rs17167306	7	133529648	C	A	0,0002	29,7349	-0,0256	0,0047	4,9E-08	-0,0759	0,0426	0,0750	instrument
rs17391694	1	78623626	T	C	0,0002	34,9869	0,0274	0,0046	3,3E-09	0,0045	0,0451	0,9209	instrument
rs17619973	3	114417675	G	A	0,0002	31,4045	-0,0345	0,0061	2,1E-08	-0,0425	0,0596	0,4760	instrument
rs17763953	14	79969194	C	T	0,0002	33,7209	0,0192	0,0033	6,4E-09	0,0306	0,0295	0,3003	instrument
rs17770336	9	28414625	T	C	0,0003	48,7199	0,0240	0,0034	3,0E-12	0,0326	0,0309	0,2912	instrument
rs1881459	5	88841488	G	A	0,0003	56,3695	0,0251	0,0033	6,0E-14	0,0188	0,0306	0,5384	instrument
rs1884897	20	6612832	A	G	0,0005	92,6589	-0,0323	0,0034	6,3E-22	-0,0164	0,0300	0,5836	instrument
rs200126474	17	47112109	A	C	0,0002	40,2967	-0,0219	0,0034	2,2E-10	-0,0437	0,0308	0,1554	instrument
rs205286	6	34551334	C	A	0,0004	75,4458	0,0299	0,0034	3,8E-18	-0,0236	0,0305	0,4380	instrument
rs2076603	1	17319011	G	A	0,0002	39,3878	0,0212	0,0034	3,5E-10	-0,0140	0,0300	0,6412	instrument
rs2101435	2	233115158	G	C	0,0002	30,7024	0,0200	0,0036	3,0E-08	0,0310	0,0328	0,3444	instrument
rs2239647	14	33292743	A	C	0,0002	37,8344	0,0200	0,0032	7,7E-10	-0,0443	0,0302	0,1432	instrument
rs2289292	16	30097630	T	C	0,0002	39,5260	-0,0216	0,0034	3,3E-10	-0,0532	0,0321	0,0968	instrument
rs2295654	14	101539197	T	C	0,0002	38,1179	-0,0231	0,0037	6,7E-10	-0,0023	0,0355	0,9478	instrument
rs2299383	7	103418846	T	C	0,0002	46,4574	0,0224	0,0033	9,4E-12	-0,0125	0,0292	0,6693	instrument
rs2346883	2	47015914	G	T	0,0002	46,9631	-0,0233	0,0034	7,2E-12	0,0185	0,0310	0,5514	instrument

rs2404324	7	99023461	G	A	0,0002	40,7167	-0,0285	0,0045	1,8E-10	0,0151	0,0394	0,7008	instrument
rs2436661	15	52145407	A	G	0,0002	36,1438	-0,0195	0,0032	1,8E-09	-0,0021	0,0286	0,9416	instrument
rs2450444	10	93010383	A	G	0,0002	42,8628	-0,0221	0,0034	5,9E-11	-0,0069	0,0302	0,8194	instrument
rs2644135	1	201856256	C	G	0,0002	40,5637	0,0216	0,0034	1,9E-10	0,0630	0,0302	0,0375	instrument
rs2708149	2	58951187	A	G	0,0003	50,6939	-0,0235	0,0033	1,1E-12	0,0011	0,0294	0,9712	instrument
rs2727316	17	61961336	G	A	0,0002	29,9534	-0,0197	0,0036	4,4E-08	0,0348	0,0319	0,2748	instrument
rs286818	5	107433446	A	T	0,0004	75,2722	-0,0374	0,0043	4,1E-18	0,0247	0,0378	0,5134	instrument
rs2953806	8	8914124	T	C	0,0004	68,3095	0,0270	0,0033	1,4E-16	-0,0008	0,0392	0,9842	instrument
rs2994320	1	243641247	G	A	0,0002	32,2848	-0,0232	0,0041	1,3E-08	-0,0283	0,0373	0,4476	instrument
rs34409196	17	70703396	C	A	0,0002	34,4448	0,0211	0,0036	4,4E-09	-0,0180	0,0336	0,5917	instrument
rs34811474	4	25408838	A	G	0,0004	75,3937	-0,0331	0,0038	3,9E-18	-0,0097	0,0399	0,8088	instrument
rs35559811	13	58666728	G	A	0,0003	61,7632	-0,0301	0,0038	3,9E-15	0,0170	0,0338	0,6148	instrument
rs3734554	6	40360781	T	C	0,0002	38,0068	0,0216	0,0035	7,1E-10	0,0175	0,0319	0,5837	instrument
rs3784710	15	68072458	C	T	0,0003	57,2589	-0,0291	0,0038	3,8E-14	0,0457	0,0339	0,1766	instrument
rs3810291	19	47569003	G	A	0,0003	66,1938	-0,0281	0,0035	4,1E-16	0,0525	0,0302	0,0822	instrument
rs3826408	17	7101292	T	C	0,0002	32,1032	0,0183	0,0032	1,5E-08	-0,0023	0,0292	0,9367	instrument
rs3845836	2	219504955	C	T	0,0002	42,5621	0,0212	0,0033	6,9E-11	0,0025	0,0290	0,9321	instrument
rs3895907	1	75006027	A	G	0,0003	51,3510	0,0234	0,0033	7,8E-13	0,0283	0,0288	0,3257	instrument
rs41271299	6	19839415	T	C	0,0002	30,2937	-0,0402	0,0073	3,7E-08	-0,0673	0,0890	0,4494	instrument
rs4304660	10	22295747	G	A	0,0002	34,9750	0,0217	0,0037	3,3E-09	0,0371	0,0318	0,2450	instrument
rs4482463	2	205375909	C	A	0,0002	36,6869	0,0372	0,0061	1,4E-09	-0,0682	0,0551	0,2158	instrument
rs4680340	3	156861460	C	T	0,0002	30,2277	0,0204	0,0037	3,8E-08	-0,0034	0,0329	0,9176	instrument
rs4790841	17	1835482	T	C	0,0003	57,7290	-0,0340	0,0045	3,0E-14	0,0230	0,0414	0,5783	instrument
rs4794670	17	54927829	T	A	0,0002	30,6802	-0,0214	0,0039	3,0E-08	0,0136	0,0371	0,7138	instrument
rs4796225	17	34843839	G	A	0,0002	38,1179	-0,0200	0,0032	6,7E-10	-0,0253	0,0302	0,4016	instrument
rs4808944	19	19426613	C	A	0,0002	39,0246	-0,0276	0,0044	4,2E-10	-0,0131	0,0393	0,7385	instrument
rs4821943	22	40722745	G	A	0,0002	35,5093	-0,0204	0,0034	2,5E-09	-0,0403	0,0299	0,1778	instrument
rs4842838	15	84582124	G	T	0,0004	74,2864	0,0278	0,0032	6,8E-18	0,0005	0,0286	0,9856	instrument
rs4889606	16	31011183	G	A	0,0004	67,1245	-0,0274	0,0033	2,6E-16	-0,0430	0,0300	0,1518	instrument
rs539515	1	177889025	C	A	0,0008	161,7967	0,0505	0,0040	5,0E-37	0,0458	0,0362	0,2052	instrument
rs55654354	3	82643954	C	T	0,0002	35,7122	-0,0258	0,0043	2,3E-09	-0,0130	0,0386	0,7364	instrument

rs56282717	7	150657095	A	G	0,0002	35,3308	-0,0225	0,0038	2,8E-09	0,0216	0,0334	0,5183	instrument
rs61037750	11	43681372	A	G	0,0002	37,4418	0,0214	0,0035	9,5E-10	0,0438	0,0310	0,1582	instrument
rs61813324	1	156049877	T	C	0,0002	42,5230	0,0317	0,0049	7,0E-11	0,0977	0,0455	0,0319	instrument
rs61910820	11	134581277	G	A	0,0002	32,2280	0,0243	0,0043	1,4E-08	-0,0126	0,0398	0,7517	instrument
rs62004866	15	74223118	G	C	0,0002	44,7289	0,0350	0,0052	2,3E-11	0,0472	0,0456	0,3008	instrument
rs62106258	2	417167	C	T	0,0008	145,2010	-0,0899	0,0075	2,1E-33	-0,1031	0,0793	0,1938	instrument
rs62131065	2	25568539	G	A	0,0002	33,3041	-0,0312	0,0054	7,9E-09	-0,0761	0,0490	0,1201	instrument
rs6548237	2	621461	A	C	0,0007	137,8262	-0,0500	0,0043	8,4E-32	-0,0704	0,0483	0,1449	instrument
rs6585200	10	114768609	G	A	0,0002	45,1848	-0,0218	0,0032	1,8E-11	-0,0325	0,0286	0,2559	instrument
rs6606652	11	69499786	G	T	0,0002	36,0717	-0,0201	0,0033	1,9E-09	-0,0537	0,0298	0,0719	instrument
rs66922415	18	57848651	G	A	0,0009	163,8383	0,0485	0,0038	1,8E-37	0,0520	0,0334	0,1195	instrument
rs6852276	4	145261999	G	A	0,0002	33,1427	0,0191	0,0033	8,6E-09	-0,0131	0,0301	0,6645	instrument
rs7045925	9	96475306	T	C	0,0002	39,1371	0,0231	0,0037	4,0E-10	-0,0082	0,0335	0,8068	instrument
rs7124681	11	47529947	A	C	0,0003	63,2336	0,0261	0,0033	1,8E-15	-0,0273	0,0292	0,3503	instrument
rs72731237	9	34148743	A	G	0,0002	34,8332	-0,0309	0,0052	3,6E-09	-0,0250	0,0458	0,5850	instrument
rs72740699	5	27188818	C	G	0,0002	31,1919	-0,0202	0,0036	2,3E-08	-0,0068	0,0329	0,8370	instrument
rs72755233	15	100692953	A	G	0,0004	72,3343	0,0432	0,0051	1,8E-17	-0,1417	0,0577	0,0140	outlier
rs72771049	16	19933511	T	G	0,0003	54,8038	-0,0342	0,0046	1,3E-13	-0,0001	0,0405	0,9981	instrument
rs72892910	6	50816887	T	G	0,0004	76,1772	0,0376	0,0043	2,6E-18	0,0784	0,0369	0,0338	instrument
rs73052033	3	185828465	C	T	0,0004	70,5929	-0,0351	0,0042	4,4E-17	-0,0313	0,0380	0,4094	instrument
rs7459710	8	77222670	T	C	0,0002	36,1438	-0,0196	0,0033	1,8E-09	-0,0084	0,0288	0,7713	instrument
rs7534091	1	118864616	G	A	0,0002	33,4890	0,0212	0,0037	7,2E-09	-0,0478	0,0331	0,1483	instrument
rs7795394	7	113560607	T	A	0,0002	33,1657	0,0192	0,0033	8,5E-09	-0,0502	0,0300	0,0945	instrument
rs879620	16	4015729	C	T	0,0002	34,4565	-0,0195	0,0033	4,4E-09	0,0122	0,0298	0,6816	instrument
rs925421	4	60253877	A	G	0,0002	34,2221	0,0218	0,0037	4,9E-09	0,0064	0,0333	0,8475	instrument
rs9688977	6	154336892	C	T	0,0002	31,2813	0,0257	0,0046	2,2E-08	0,0091	0,0399	0,8188	instrument
rs9929088	16	29001460	A	C	0,0002	39,2373	0,0218	0,0035	3,8E-10	0,0590	0,0318	0,0638	instrument

Table S26. Single Nucleotide Polymorphisms (SNPs) used as instruments in the Mendelian randomization analyses for the effect of AFR on high or low serous ovarian cancer based on the genome-wide significance threshold P = 5E-08. R² represents the explained variance in the exposure. SNP-specific instrument strength is given by the respective F-statistic. Some of the genetic variants were identified as outliers in the iterative approach and therefore excluded from the analyses.

SNP	CHR	POS	EA	OA	R^2	F-stat	β_{AFR}	SE_{AFR}	P_{AFR}	$\beta_{High\ low\ serous}$	$S_{High\ low\ serous}$	$P_{High\ low\ serous}$	$variant_{High\ low\ serous}$
rs10013032	4	120149419	C	T	0,0002	38,6880	0,0208	0,0033	5,0E-10	0,0063	0,0160	0,6940	instrument
rs10057598	5	64070812	C	T	0,0002	35,5451	0,0196	0,0033	2,5E-09	0,0345	0,0157	0,0279	instrument
rs10182458	2	25150641	G	A	0,0010	192,9301	0,0449	0,0032	8,1E-44	0,0032	0,0157	0,8375	instrument
rs10484759	6	127037042	T	C	0,0002	31,1472	0,0208	0,0037	2,4E-08	0,0327	0,0180	0,0698	instrument
rs1048932	11	115044850	A	C	0,0002	32,5809	-0,0187	0,0033	1,1E-08	0,0155	0,0155	0,3180	instrument
rs10504303	8	60859424	A	T	0,0002	33,6281	-0,0194	0,0034	6,7E-09	-0,0072	0,0162	0,6576	instrument
rs10761785	10	65318766	G	T	0,0002	42,7188	0,0212	0,0032	6,3E-11	-0,0039	0,0152	0,7991	instrument
rs10797119	9	92202495	T	C	0,0002	33,4543	-0,0189	0,0033	7,3E-09	0,0123	0,0154	0,4279	instrument
rs10800938	1	190220059	A	G	0,0002	38,7627	0,0202	0,0032	4,8E-10	0,0022	0,0157	0,8901	instrument
rs10916174	1	227804041	A	G	0,0002	35,8438	0,0266	0,0044	2,1E-09	-0,0103	0,0218	0,6378	instrument
rs10962549	9	16719445	T	C	0,0003	48,5107	0,0303	0,0043	3,3E-12	0,0513	0,0212	0,0157	instrument
rs11030119	11	27728102	A	G	0,0003	61,7004	0,0274	0,0035	4,0E-15	0,0147	0,0169	0,3851	instrument
rs11159247	14	77509872	A	G	0,0002	31,7642	0,0199	0,0035	1,7E-08	-0,0018	0,0166	0,9117	instrument
rs112566467	1	39562627	T	C	0,0002	31,8431	0,0225	0,0040	1,7E-08	-0,0510	0,0191	0,0076	outlier
rs112726905	19	32822348	G	A	0,0002	29,8878	-0,0201	0,0037	4,6E-08	0,0020	0,0179	0,9109	instrument
rs112920189	7	75094545	A	C	0,0003	55,8900	-0,0243	0,0033	7,7E-14	0,0111	0,0157	0,4781	instrument
rs113996403	2	95911816	C	G	0,0002	31,2925	0,0724	0,0129	2,2E-08	-0,0586	0,0586	0,3174	instrument
rs114938354	5	176732485	C	G	0,0002	40,6530	0,0642	0,0101	1,8E-10	-0,0084	0,0481	0,8619	instrument
rs11642015	16	53802494	T	C	0,0020	380,6361	0,0641	0,0033	1,1E-84	0,0108	0,0154	0,4819	instrument
rs11721374	4	8599520	A	G	0,0002	43,0200	0,0215	0,0033	5,4E-11	0,0045	0,0161	0,7805	instrument
rs1173734	5	32766417	C	T	0,0002	31,3149	-0,0206	0,0037	2,2E-08	-0,0072	0,0178	0,6882	instrument
rs11766944	7	1888051	A	G	0,0002	35,6405	-0,0250	0,0042	2,4E-09	0,0072	0,0190	0,7044	instrument
rs117700741	19	8637832	T	C	0,0002	31,6403	0,0511	0,0091	1,9E-08	0,0355	0,0465	0,4453	instrument
rs11851616	14	104065600	A	G	0,0003	57,5620	0,0266	0,0035	3,3E-14	-0,0008	0,0166	0,9597	instrument
rs11881199	19	18837459	T	C	0,0002	36,8202	0,0197	0,0033	1,3E-09	-0,0032	0,0156	0,8379	instrument
rs1207949	13	33008393	C	T	0,0002	45,8866	-0,0229	0,0034	1,3E-11	-0,0153	0,0161	0,3406	instrument
rs12140153	1	62579891	T	G	0,0002	30,2497	-0,0316	0,0057	3,8E-08	0,0111	0,0282	0,6948	instrument

rs12233810	4	17859768	T	C	0,0002	36,0116	-0,0218	0,0036	2,0E-09	0,0365	0,0174	0,0357	instrument
rs12357890	10	99762693	A	G	0,0002	45,5215	-0,0222	0,0033	1,5E-11	0,0050	0,0158	0,7542	instrument
rs12432754	14	94030357	A	G	0,0002	40,8444	-0,0216	0,0034	1,6E-10	-0,0147	0,0160	0,3566	instrument
rs12575252	11	8694073	C	G	0,0002	31,8093	-0,0193	0,0034	1,7E-08	-0,0422	0,0161	0,0087	outlier
rs12805133	11	66483265	G	A	0,0003	53,8750	0,0238	0,0032	2,1E-13	-0,0079	0,0155	0,6091	instrument
rs12926506	16	81722413	T	C	0,0002	32,0353	-0,0247	0,0044	1,5E-08	0,0186	0,0224	0,4074	instrument
rs13002946	2	100801959	A	T	0,0003	51,9547	-0,0265	0,0037	5,7E-13	-0,0339	0,0176	0,0539	instrument
rs13107325	4	103188709	T	C	0,0004	70,8620	0,0520	0,0062	3,9E-17	0,0455	0,0294	0,1210	instrument
rs13174863	5	139080745	G	A	0,0002	42,3276	0,0300	0,0046	7,7E-11	0,0006	0,0227	0,9791	instrument
rs13434116	3	136353074	C	G	0,0003	51,1792	0,0271	0,0038	8,4E-13	0,0074	0,0179	0,6792	instrument
rs150468996	1	1591869	C	T	0,0002	36,2280	-0,0215	0,0036	1,8E-09	0,0097	0,0210	0,6446	instrument
rs16942323	15	89383764	C	T	0,0002	35,6764	0,0603	0,0101	2,3E-09	-0,0247	0,0512	0,6292	instrument
rs17066856	18	58049656	C	T	0,0003	47,8997	-0,0391	0,0056	4,5E-12	-0,0306	0,0264	0,2475	instrument
rs1708302	7	28198677	C	T	0,0003	63,7915	-0,0257	0,0032	1,4E-15	-0,0225	0,0152	0,1378	instrument
rs1715896	15	56828094	G	A	0,0002	38,3528	0,0203	0,0033	5,9E-10	-0,0332	0,0155	0,0318	instrument
rs17167306	7	133529648	C	A	0,0002	29,7349	-0,0256	0,0047	4,9E-08	-0,0118	0,0224	0,5989	instrument
rs17391694	1	78623626	T	C	0,0002	34,9869	0,0274	0,0046	3,3E-09	0,0114	0,0241	0,6371	instrument
rs17619973	3	114417675	G	A	0,0002	31,4045	-0,0345	0,0061	2,1E-08	0,0589	0,0308	0,0557	instrument
rs17763953	14	79969194	C	T	0,0002	33,7209	0,0192	0,0033	6,4E-09	-0,0176	0,0158	0,2653	instrument
rs17770336	9	28414625	T	C	0,0003	48,7199	0,0240	0,0034	3,0E-12	0,0293	0,0165	0,0766	instrument
rs1881459	5	88841488	G	A	0,0003	56,3695	0,0251	0,0033	6,0E-14	-0,0215	0,0164	0,1884	instrument
rs1884897	20	6612832	A	G	0,0005	92,6589	-0,0323	0,0034	6,3E-22	0,0146	0,0160	0,3623	instrument
rs200126474	17	47112109	A	C	0,0002	40,2967	-0,0219	0,0034	2,2E-10	0,0064	0,0163	0,6948	instrument
rs205286	6	34551334	C	A	0,0004	75,4458	0,0299	0,0034	3,8E-18	0,0248	0,0161	0,1228	instrument
rs2076603	1	17319011	G	A	0,0002	39,3878	0,0212	0,0034	3,5E-10	-0,0497	0,0160	0,0019	outlier
rs2101435	2	233115158	G	C	0,0002	30,7024	0,0200	0,0036	3,0E-08	-0,0027	0,0176	0,8773	instrument
rs2239647	14	33292743	A	C	0,0002	37,8344	0,0200	0,0032	7,7E-10	0,0063	0,0161	0,6962	instrument
rs2289292	16	30097630	T	C	0,0002	39,5260	-0,0216	0,0034	3,3E-10	0,0060	0,0170	0,7235	instrument
rs2295654	14	101539197	T	C	0,0002	38,1179	-0,0231	0,0037	6,7E-10	-0,0045	0,0190	0,8141	instrument
rs2299383	7	103418846	T	C	0,0002	46,4574	0,0224	0,0033	9,4E-12	-0,0133	0,0156	0,3944	instrument
rs2346883	2	47015914	G	T	0,0002	46,9631	-0,0233	0,0034	7,2E-12	-0,0088	0,0166	0,5948	instrument

rs2404324	7	99023461	G	A	0,0002	40,7167	-0,0285	0,0045	1,8E-10	0,0285	0,0210	0,1743	instrument
rs2436661	15	52145407	A	G	0,0002	36,1438	-0,0195	0,0032	1,8E-09	0,0018	0,0153	0,9073	instrument
rs2450444	10	93010383	A	G	0,0002	42,8628	-0,0221	0,0034	5,9E-11	-0,0114	0,0162	0,4788	instrument
rs2644135	1	201856256	C	G	0,0002	40,5637	0,0216	0,0034	1,9E-10	-0,0272	0,0164	0,0969	instrument
rs2708149	2	58951187	A	G	0,0003	50,6939	-0,0235	0,0033	1,1E-12	-0,0449	0,0157	0,0043	outlier
rs2727316	17	61961336	G	A	0,0002	29,9534	-0,0197	0,0036	4,4E-08	0,0286	0,0170	0,0927	instrument
rs286818	5	107433446	A	T	0,0004	75,2722	-0,0374	0,0043	4,1E-18	-0,0295	0,0203	0,1465	instrument
rs2953806	8	8914124	T	C	0,0004	68,3095	0,0270	0,0033	1,4E-16	-0,0198	0,0211	0,3475	instrument
rs2994320	1	243641247	G	A	0,0002	32,2848	-0,0232	0,0041	1,3E-08	0,0324	0,0197	0,1012	instrument
rs34409196	17	70703396	C	A	0,0002	34,4448	0,0211	0,0036	4,4E-09	-0,0192	0,0180	0,2874	instrument
rs34811474	4	25408838	A	G	0,0004	75,3937	-0,0331	0,0038	3,9E-18	-0,0216	0,0212	0,3093	instrument
rs35559811	13	58666728	G	A	0,0003	61,7632	-0,0301	0,0038	3,9E-15	0,0350	0,0179	0,0508	instrument
rs3734554	6	40360781	T	C	0,0002	38,0068	0,0216	0,0035	7,1E-10	0,0263	0,0169	0,1198	instrument
rs3784710	15	68072458	C	T	0,0003	57,2589	-0,0291	0,0038	3,8E-14	-0,0208	0,0182	0,2533	instrument
rs3810291	19	47569003	G	A	0,0003	66,1938	-0,0281	0,0035	4,1E-16	0,0143	0,0162	0,3767	instrument
rs3826408	17	7101292	T	C	0,0002	32,1032	0,0183	0,0032	1,5E-08	-0,0117	0,0156	0,4510	instrument
rs3845836	2	219504955	C	T	0,0002	42,5621	0,0212	0,0033	6,9E-11	-0,0352	0,0155	0,0229	instrument
rs3895907	1	75006027	A	G	0,0003	51,3510	0,0234	0,0033	7,8E-13	0,0030	0,0153	0,8464	instrument
rs41271299	6	19839415	T	C	0,0002	30,2937	-0,0402	0,0073	3,7E-08	-0,0200	0,0473	0,6726	instrument
rs4304660	10	22295747	G	A	0,0002	34,9750	0,0217	0,0037	3,3E-09	0,0318	0,0170	0,0612	instrument
rs4482463	2	205375909	C	A	0,0002	36,6869	0,0372	0,0061	1,4E-09	-0,0804	0,0293	0,0061	outlier
rs4680340	3	156861460	C	T	0,0002	30,2277	0,0204	0,0037	3,8E-08	-0,0291	0,0177	0,0994	instrument
rs4790841	17	1835482	T	C	0,0003	57,7290	-0,0340	0,0045	3,0E-14	0,0270	0,0220	0,2196	instrument
rs4794670	17	54927829	T	A	0,0002	30,6802	-0,0214	0,0039	3,0E-08	0,0183	0,0198	0,3546	instrument
rs4796225	17	34843839	G	A	0,0002	38,1179	-0,0200	0,0032	6,7E-10	0,0141	0,0161	0,3805	instrument
rs4808944	19	19426613	C	A	0,0002	39,0246	-0,0276	0,0044	4,2E-10	0,0497	0,0206	0,0156	instrument
rs4821943	22	40722745	G	A	0,0002	35,5093	-0,0204	0,0034	2,5E-09	-0,0124	0,0160	0,4375	instrument
rs4842838	15	84582124	G	T	0,0004	74,2864	0,0278	0,0032	6,8E-18	-0,0141	0,0152	0,3529	instrument
rs4889606	16	31011183	G	A	0,0004	67,1245	-0,0274	0,0033	2,6E-16	-0,0116	0,0159	0,4668	instrument
rs539515	1	177889025	C	A	0,0008	161,7967	0,0505	0,0040	5,0E-37	-0,0107	0,0195	0,5820	instrument
rs55654354	3	82643954	C	T	0,0002	35,7122	-0,0258	0,0043	2,3E-09	0,0068	0,0205	0,7421	instrument

rs56282717	7	150657095	A	G	0,0002	35,3308	-0,0225	0,0038	2,8E-09	0,0019	0,0180	0,9151	instrument
rs61037750	11	43681372	A	G	0,0002	37,4418	0,0214	0,0035	9,5E-10	0,0172	0,0165	0,2980	instrument
rs61813324	1	156049877	T	C	0,0002	42,5230	0,0317	0,0049	7,0E-11	0,0215	0,0247	0,3839	instrument
rs61910820	11	134581277	G	A	0,0002	32,2280	0,0243	0,0043	1,4E-08	0,0185	0,0212	0,3811	instrument
rs62004866	15	74223118	G	C	0,0002	44,7289	0,0350	0,0052	2,3E-11	0,0413	0,0246	0,0923	instrument
rs62106258	2	417167	C	T	0,0008	145,2010	-0,0899	0,0075	2,1E-33	-0,0130	0,0414	0,7538	instrument
rs62131065	2	25568539	G	A	0,0002	33,3041	-0,0312	0,0054	7,9E-09	0,0279	0,0255	0,2740	instrument
rs6548237	2	621461	A	C	0,0007	137,8262	-0,0500	0,0043	8,4E-32	-0,0096	0,0255	0,7076	instrument
rs6585200	10	114768609	G	A	0,0002	45,1848	-0,0218	0,0032	1,8E-11	-0,0336	0,0153	0,0278	instrument
rs6606652	11	69499786	G	T	0,0002	36,0717	-0,0201	0,0033	1,9E-09	-0,0048	0,0158	0,7622	instrument
rs66922415	18	57848651	G	A	0,0009	163,8383	0,0485	0,0038	1,8E-37	-0,0152	0,0180	0,3975	instrument
rs6852276	4	145261999	G	A	0,0002	33,1427	0,0191	0,0033	8,6E-09	0,0111	0,0160	0,4866	instrument
rs7045925	9	96475306	T	C	0,0002	39,1371	0,0231	0,0037	4,0E-10	0,0110	0,0178	0,5370	instrument
rs7124681	11	47529947	A	C	0,0003	63,2336	0,0261	0,0033	1,8E-15	0,0170	0,0156	0,2750	instrument
rs72731237	9	34148743	A	G	0,0002	34,8332	-0,0309	0,0052	3,6E-09	-0,0095	0,0244	0,6970	instrument
rs72740699	5	27188818	C	G	0,0002	31,1919	-0,0202	0,0036	2,3E-08	0,0218	0,0174	0,2105	instrument
rs72755233	15	100692953	A	G	0,0004	72,3343	0,0432	0,0051	1,8E-17	-0,0173	0,0300	0,5644	instrument
rs72771049	16	19933511	T	G	0,0003	54,8038	-0,0342	0,0046	1,3E-13	-0,0422	0,0219	0,0535	instrument
rs72892910	6	50816887	T	G	0,0004	76,1772	0,0376	0,0043	2,6E-18	-0,0134	0,0201	0,5058	instrument
rs73052033	3	185828465	C	T	0,0004	70,5929	-0,0351	0,0042	4,4E-17	-0,0089	0,0202	0,6607	instrument
rs7459710	8	77222670	T	C	0,0002	36,1438	-0,0196	0,0033	1,8E-09	0,0200	0,0153	0,1926	instrument
rs7534091	1	118864616	G	A	0,0002	33,4890	0,0212	0,0037	7,2E-09	0,0122	0,0175	0,4854	instrument
rs7795394	7	113560607	T	A	0,0002	33,1657	0,0192	0,0033	8,5E-09	-0,0082	0,0159	0,6068	instrument
rs879620	16	4015729	C	T	0,0002	34,4565	-0,0195	0,0033	4,4E-09	0,0009	0,0159	0,9524	instrument
rs925421	4	60253877	A	G	0,0002	34,2221	0,0218	0,0037	4,9E-09	0,0080	0,0177	0,6509	instrument
rs9688977	6	154336892	C	T	0,0002	31,2813	0,0257	0,0046	2,2E-08	-0,0178	0,0214	0,4068	instrument
rs9929088	16	29001460	A	C	0,0002	39,2373	0,0218	0,0035	3,8E-10	-0,0048	0,0171	0,7771	instrument

Table S27. Single Nucleotide Polymorphisms (SNPs) used as instruments in the Mendelian randomization analyses for the effect of LFR on breast cancer based on the genome-wide significance threshold P = 5E-08. R^2 represents the explained variance in the exposure. SNP-specific instrument strength is given by the respective F-statistic. Some of the genetic variants were identified as outliers in the iterative approach and therefore excluded from the analyses.

SNP	CHR	POS	EA	OA	R2	Fstat	β_{LFR}	SE_{LFR}	P_{LFR}	β_{Breast}	SE_{Breast}	P_{breast}	$variant_{Breast}$
rs1002436	1	89146852	A	G	0,0002	33,9070	0,0189	0,0032	5,8E-09	-0,0172	0,0063	0,0063	outlier
rs1007358	7	46201355	G	A	0,0002	31,5391	-0,0221	0,0039	2,0E-08	0,0002	0,0079	0,9765	instrument
rs10172196	2	36780549	A	G	0,0003	53,8017	-0,0259	0,0035	2,2E-13	-0,0052	0,0068	0,4429	instrument
rs1020048	9	86666993	A	C	0,0002	40,1825	-0,0265	0,0042	2,3E-10	0,0077	0,0079	0,3258	instrument
rs10445034	16	88840564	T	C	0,0002	38,3776	0,0239	0,0039	5,8E-10	-0,0046	0,0075	0,5374	instrument
rs10894192	11	130266117	T	A	0,0002	44,5418	0,0221	0,0033	2,5E-11	-0,0031	0,0070	0,6524	instrument
rs10901171	9	133427194	C	T	0,0002	43,2039	0,0220	0,0033	4,9E-11	-0,0023	0,0068	0,7416	instrument
rs11205303	1	149906413	C	T	0,0018	340,7680	-0,0605	0,0033	4,8E-76	0,0497	0,0064	1,1E-14	outlier
rs112540634	6	34623905	T	C	0,0006	106,9145	-0,0487	0,0047	4,7E-25	-0,0105	0,0089	0,2368	instrument
rs11259936	15	84580582	A	C	0,0012	235,0064	0,0495	0,0032	5,0E-53	0,0059	0,0062	0,3413	instrument
rs112635299	14	94838142	T	G	0,0004	70,8115	-0,0942	0,0112	3,9E-17	-0,0562	0,0242	0,0203	instrument
rs113059026	10	32158478	A	C	0,0002	42,1586	0,0233	0,0036	8,4E-11	-0,0213	0,0068	0,0019	outlier
rs11466399	1	218591623	A	G	0,0003	63,9993	-0,0287	0,0036	1,3E-15	0,0050	0,0068	0,4698	instrument
rs11666808	19	18383506	T	C	0,0002	46,9631	-0,0230	0,0033	7,2E-12	0,0091	0,0066	0,1671	instrument
rs11695471	2	25457708	A	T	0,0005	88,6790	0,0324	0,0034	4,7E-21	-0,0176	0,0070	0,0121	instrument
rs12103006	16	24726237	A	G	0,0002	31,2925	0,0183	0,0033	2,2E-08	-0,0008	0,0063	0,9021	instrument
rs12238852	9	98382084	T	C	0,0002	40,8700	-0,0296	0,0046	1,6E-10	0,0116	0,0090	0,1988	instrument
rs12756815	1	113207243	A	C	0,0003	62,4410	0,0316	0,0040	2,7E-15	-0,0059	0,0077	0,4400	instrument
rs13128180	4	56286188	T	G	0,0003	57,4558	-0,0267	0,0035	3,5E-14	0,0055	0,0067	0,4080	instrument
rs1313237	14	60848224	C	A	0,0003	50,8506	-0,0236	0,0033	1,0E-12	-0,0008	0,0064	0,9020	instrument
rs132941	22	38545942	C	T	0,0003	64,2716	-0,0261	0,0033	1,1E-15	-0,0364	0,0062	4,8E-09	outlier
rs13306455	19	7184721	T	C	0,0002	32,1259	-0,0244	0,0043	1,4E-08	-0,0021	0,0083	0,8001	instrument
rs138809270	2	219929394	T	C	0,0002	39,7778	0,0828	0,0131	2,8E-10	-0,0136	0,0247	0,5800	instrument
rs143384	20	34025756	G	A	0,0009	179,8262	-0,0441	0,0033	5,4E-41	-0,0020	0,0064	0,7561	instrument
rs143840904	11	2813322	T	C	0,0002	44,7289	0,0842	0,0126	2,3E-11	-0,0224	0,0321	0,4851	instrument
rs1452815	8	78123634	G	A	0,0002	30,3818	-0,0197	0,0036	3,6E-08	0,0195	0,0068	0,0045	outlier
rs1513304	3	99269750	C	T	0,0002	40,5765	-0,0226	0,0035	1,9E-10	0,0273	0,0073	0,0002	outlier

rs1550436	15	74221157	T	C	0,0004	77,3160	0,0286	0,0032	1,5E-18	0,0127	0,0062	0,0400	instrument
rs16942324	15	89383854	A	C	0,0004	70,6937	0,0844	0,0100	4,2E-17	0,0232	0,0230	0,3129	instrument
rs16948048	17	47440466	G	A	0,0002	42,5621	0,0218	0,0033	6,9E-11	0,0106	0,0064	0,0974	instrument
rs16953461	17	46261736	G	T	0,0003	54,8186	0,0292	0,0039	1,3E-13	0,0064	0,0076	0,3970	instrument
rs16972537	16	73802389	A	G	0,0002	33,0736	-0,0194	0,0034	8,9E-09	-0,0085	0,0067	0,2040	instrument
rs17698176	17	44819595	G	T	0,0003	62,1883	-0,0307	0,0039	3,1E-15	0,0163	0,0080	0,0413	instrument
rs17744040	9	16842137	C	T	0,0002	34,5740	0,0309	0,0053	4,1E-09	0,0163	0,0094	0,0821	instrument
rs1801282	3	12393125	G	C	0,0003	51,5519	-0,0356	0,0050	7,0E-13	-0,0165	0,0094	0,0794	instrument
rs1980030	10	120970027	G	A	0,0002	32,0806	-0,0186	0,0033	1,5E-08	0,0026	0,0066	0,6889	instrument
rs2007482	6	2169724	T	C	0,0002	36,6626	0,0199	0,0033	1,4E-09	-0,0140	0,0067	0,0360	instrument
rs2058908	16	53806145	T	C	0,0002	44,3951	0,0243	0,0037	2,7E-11	0,0345	0,0073	2,0E-06	outlier
rs2126643	1	103404384	T	C	0,0002	40,1318	-0,0218	0,0034	2,4E-10	0,0048	0,0065	0,4671	instrument
rs2158694	7	2863443	G	T	0,0008	161,7967	0,0450	0,0035	4,5E-37	0,0144	0,0067	0,0331	outlier
rs2173295	1	54943264	C	A	0,0002	33,0737	0,0276	0,0048	8,9E-09	0,0074	0,0100	0,4606	instrument
rs2274432	1	184020945	A	G	0,0005	86,6007	-0,0315	0,0034	1,3E-20	0,0097	0,0065	0,1343	instrument
rs2293576	11	47434986	A	G	0,0003	48,9295	0,0240	0,0034	2,7E-12	-0,0178	0,0066	0,0069	outlier
rs2370983	14	79903376	G	A	0,0002	33,4890	0,0196	0,0034	7,2E-09	-0,0185	0,0069	0,0076	outlier
rs2516633	11	62371773	A	C	0,0002	31,4718	0,0197	0,0035	2,0E-08	0,0066	0,0072	0,3601	instrument
rs256904	5	55810305	A	T	0,0003	66,6012	-0,0302	0,0037	3,3E-16	-0,0041	0,0075	0,5811	instrument
rs2641699	16	84886306	C	G	0,0002	35,6047	0,0200	0,0034	2,4E-09	0,0101	0,0070	0,1458	instrument
rs264762	5	64384386	A	C	0,0003	52,4171	0,0248	0,0034	4,5E-13	0,0205	0,0066	0,0018	outlier
rs2712169	2	217671349	G	A	0,0003	49,6879	-0,0233	0,0033	1,8E-12	0,0140	0,0063	0,0274	instrument
rs2715094	7	50730452	G	A	0,0003	57,7290	-0,0282	0,0037	3,0E-14	-0,0008	0,0076	0,9153	instrument
rs2768641	9	96948863	G	A	0,0002	34,4213	-0,0455	0,0078	4,4E-09	-0,0366	0,0162	0,0241	instrument
rs2811462	3	129303436	G	T	0,0003	52,3447	-0,0281	0,0039	4,7E-13	0,0225	0,0077	0,0035	outlier
rs2820446	1	219748818	G	C	0,0007	132,7090	-0,0405	0,0035	1,1E-30	-0,0181	0,0069	0,0090	outlier
rs2844795	6	30073847	C	T	0,0002	41,4345	-0,0208	0,0032	1,2E-10	-0,0005	0,0063	0,9426	instrument
rs28539246	17	29226630	A	T	0,0003	48,5804	0,0231	0,0033	3,2E-12	-0,0348	0,0064	4,6E-08	outlier
rs28716235	8	135602976	C	A	0,0002	33,4427	0,0194	0,0034	7,4E-09	-0,0117	0,0064	0,0701	instrument
rs2886564	1	42496924	G	A	0,0002	31,4381	-0,0200	0,0036	2,1E-08	0,0004	0,0067	0,9497	instrument
rs3020421	6	152348122	A	G	0,0002	35,0224	-0,0206	0,0035	3,3E-09	0,0081	0,0066	0,2198	instrument

rs3218036	19	30305684	A	G	0,0002	29,8550	-0,0188	0,0034	4,7E-08	0,0234	0,0066	0,0004	outlier
rs34517439	1	78450517	A	C	0,0003	65,2049	-0,0403	0,0050	6,8E-16	0,0104	0,0105	0,3211	instrument
rs34580448	5	82810884	C	T	0,0004	79,5477	0,0727	0,0081	4,8E-19	-0,0102	0,0162	0,5289	instrument
rs35307904	9	78511889	A	G	0,0006	116,4229	0,0537	0,0050	3,8E-27	0,0115	0,0105	0,2767	instrument
rs35874463	15	67457698	G	A	0,0003	66,0962	-0,0563	0,0069	4,3E-16	0,0672	0,0142	2,3E-06	outlier
rs3738814	1	17331676	G	A	0,0003	60,4500	0,0253	0,0033	7,6E-15	-0,0102	0,0062	0,1005	instrument
rs3791679	2	56096892	G	A	0,0009	179,2902	0,0516	0,0039	7,3E-41	-0,0014	0,0073	0,8457	instrument
rs3794782	17	18127914	C	G	0,0002	33,0277	-0,0191	0,0033	9,1E-09	-0,0100	0,0064	0,1171	instrument
rs3823974	7	20442796	C	T	0,0003	60,9486	0,0258	0,0033	5,9E-15	-0,0038	0,0063	0,5452	instrument
rs3850639	1	172253634	C	T	0,0003	56,8058	-0,0297	0,0039	4,8E-14	-0,0086	0,0075	0,2550	instrument
rs4073717	5	170864021	T	G	0,0002	33,2233	0,0233	0,0040	8,2E-09	0,0030	0,0080	0,7117	instrument
rs41284816	13	50655989	T	G	0,0002	46,3484	-0,0835	0,0123	9,9E-12	0,0364	0,0204	0,0748	instrument
rs41313250	1	62483623	G	A	0,0002	36,9903	0,0319	0,0052	1,2E-09	-0,0059	0,0097	0,5472	instrument
rs4236324	7	27302727	C	T	0,0002	37,5030	-0,0274	0,0045	9,1E-10	-0,0071	0,0089	0,4221	instrument
rs4246308	15	100765202	A	G	0,0003	59,4589	0,0250	0,0032	1,3E-14	0,0101	0,0062	0,1018	instrument
rs4321401	2	69348216	T	C	0,0003	48,9715	0,0227	0,0032	2,6E-12	-0,0068	0,0068	0,3145	instrument
rs4421120	5	32763118	A	G	0,0003	50,1542	-0,0311	0,0044	1,4E-12	-0,0017	0,0090	0,8471	instrument
rs4432932	5	115039414	G	A	0,0002	31,1249	-0,0204	0,0037	2,4E-08	-0,0046	0,0069	0,5020	instrument
rs4518624	8	130715510	A	G	0,0002	38,0685	0,0203	0,0033	6,8E-10	-0,0018	0,0063	0,7767	instrument
rs454345	5	88729851	G	A	0,0002	33,8255	-0,0194	0,0033	6,0E-09	0,0103	0,0065	0,1127	instrument
rs4803461	19	41908871	G	A	0,0002	33,2003	-0,0190	0,0033	8,3E-09	0,0060	0,0066	0,3685	instrument
rs4842133	9	139127566	A	C	0,0004	69,4715	-0,0300	0,0036	7,8E-17	0,0162	0,0072	0,0254	instrument
rs4901541	14	55235282	C	T	0,0002	40,4873	0,0211	0,0033	2,0E-10	-0,0048	0,0070	0,4947	instrument
rs4936122	11	130734264	C	A	0,0002	30,3818	-0,0181	0,0033	3,6E-08	-0,0035	0,0065	0,5878	instrument
rs496298	10	131355130	A	G	0,0003	65,4151	0,0276	0,0034	6,1E-16	-0,0026	0,0066	0,6917	instrument
rs4973032	2	232990462	T	G	0,0002	40,7423	0,0769	0,0121	1,7E-10	0,0247	0,0221	0,2636	instrument
rs4974072	3	41222586	A	G	0,0002	43,3091	-0,0214	0,0033	4,7E-11	0,0055	0,0062	0,3780	instrument
rs538656	18	57850422	T	G	0,0002	31,4269	-0,0213	0,0038	2,1E-08	-0,0226	0,0073	0,0020	outlier
rs544148	6	117466494	A	G	0,0002	30,7134	0,0182	0,0033	3,0E-08	0,0048	0,0063	0,4482	instrument
rs55633655	5	131598756	T	G	0,0003	53,1144	0,0237	0,0033	3,2E-13	-0,0233	0,0062	0,0002	outlier
rs56021161	14	64817100	A	T	0,0002	30,5916	0,0362	0,0065	3,2E-08	0,0130	0,0133	0,3301	instrument

rs56186137	16	28825953	G	A	0,0006	108,1589	-0,0341	0,0033	2,6E-25	0,0042	0,0064	0,5078	instrument
rs56277550	21	18092263	A	G	0,0002	33,5006	0,0187	0,0032	7,1E-09	-0,0042	0,0065	0,5154	instrument
rs57674902	20	4096741	T	C	0,0002	38,1302	-0,0210	0,0034	6,6E-10	0,0076	0,0071	0,2846	instrument
rs584961	11	75277628	A	G	0,0002	35,1408	-0,0302	0,0051	3,1E-09	0,0345	0,0108	0,0014	outlier
rs61734651	20	61451332	T	C	0,0002	37,8836	-0,0421	0,0068	7,5E-10	-0,0144	0,0147	0,3280	instrument
rs61834590	1	227421117	G	T	0,0002	43,2696	0,0298	0,0045	4,8E-11	-0,0107	0,0087	0,2218	instrument
rs62106258	2	417167	C	T	0,0002	32,2053	0,0424	0,0075	1,4E-08	0,0142	0,0181	0,4318	instrument
rs62271373	3	150066540	A	T	0,0002	41,1775	0,0455	0,0071	1,4E-10	-0,0119	0,0164	0,4672	instrument
rs62515452	8	57187356	T	C	0,0002	34,2338	-0,0267	0,0046	4,9E-09	0,0011	0,0098	0,9094	instrument
rs62621197	19	8670147	T	C	0,0008	150,7968	0,1140	0,0093	1,1E-34	-0,0203	0,0218	0,3535	instrument
rs640245	11	30393360	T	C	0,0002	37,0512	-0,0198	0,0033	1,1E-09	-0,0032	0,0063	0,6166	instrument
rs6486059	11	12824581	A	G	0,0003	52,4315	-0,0238	0,0033	4,5E-13	-0,0004	0,0065	0,9483	instrument
rs6503410	17	43094140	T	C	0,0002	35,0934	0,0215	0,0036	3,2E-09	0,0030	0,0069	0,6684	instrument
rs6585827	10	124165615	A	G	0,0002	45,0505	-0,0217	0,0032	1,9E-11	0,0031	0,0064	0,6294	instrument
rs6717858	2	165539661	C	T	0,0003	64,7696	-0,0265	0,0033	8,5E-16	-0,0091	0,0063	0,1461	instrument
rs6739701	2	105132332	G	A	0,0002	44,8493	-0,0220	0,0033	2,1E-11	-0,0057	0,0071	0,4201	instrument
rs6785012	3	141109348	T	C	0,0012	231,9505	-0,0496	0,0033	2,3E-52	0,0473	0,0062	3,3E-14	outlier
rs68049170	10	72432047	A	G	0,0003	56,1594	-0,0273	0,0036	6,7E-14	0,0005	0,0070	0,9440	instrument
rs6843340	4	48619937	T	C	0,0002	30,9912	-0,0180	0,0032	2,6E-08	0,0237	0,0062	0,0001	outlier
rs6887247	5	157881647	C	T	0,0003	53,8897	-0,0257	0,0035	2,1E-13	0,0165	0,0069	0,0169	instrument
rs702605	5	53167028	T	C	0,0002	34,1286	-0,0189	0,0032	5,2E-09	-0,0171	0,0062	0,0057	outlier
rs71531541	6	7066481	C	T	0,0002	30,1727	0,0200	0,0036	4,0E-08	-0,0141	0,0071	0,0490	instrument
rs7155279	14	92485881	T	G	0,0003	47,9967	0,0233	0,0034	4,3E-12	0,0088	0,0064	0,1687	instrument
rs7170686	15	48821350	A	G	0,0002	36,2761	0,0334	0,0055	1,7E-09	0,0249	0,0117	0,0338	instrument
rs7191697	16	89678165	A	G	0,0002	46,8946	0,0369	0,0054	7,5E-12	-0,0180	0,0115	0,1177	instrument
rs7234167	18	46744610	C	G	0,0002	30,4480	0,0206	0,0037	3,4E-08	-0,0114	0,0071	0,1082	instrument
rs7244464	18	20729714	A	C	0,0005	90,7247	0,0372	0,0039	1,7E-21	0,0158	0,0076	0,0380	instrument
rs72708236	1	155980320	G	T	0,0003	54,7890	0,0703	0,0095	1,3E-13	-0,0297	0,0202	0,1424	instrument
rs72755233	15	100692953	A	G	0,0008	160,7807	0,0645	0,0051	7,6E-37	0,0039	0,0127	0,7583	instrument
rs72776438	5	77438797	T	G	0,0002	36,1798	0,0228	0,0038	1,8E-09	-0,0050	0,0083	0,5460	instrument
rs73004967	19	19717056	G	A	0,0002	40,6530	0,0405	0,0064	1,8E-10	0,0080	0,0124	0,5197	instrument

rs73175572	3	185490184	G	A	0,0002	36,0236	-0,0314	0,0052	2,0E-09	0,0105	0,0108	0,3316	instrument
rs732563	8	23345526	T	C	0,0002	41,4088	0,0208	0,0032	1,2E-10	-0,0087	0,0062	0,1561	instrument
rs73407643	6	35169123	C	T	0,0002	36,6869	0,0751	0,0124	1,4E-09	0,0292	0,0242	0,2281	instrument
rs741207	4	18321092	T	C	0,0002	35,8677	-0,0200	0,0033	2,1E-09	0,0062	0,0063	0,3274	instrument
rs7502307	17	65989961	G	C	0,0003	54,1543	-0,0300	0,0041	1,9E-13	0,0054	0,0079	0,4904	instrument
rs75223147	6	19831020	G	T	0,0003	56,7154	-0,0848	0,0113	5,1E-14	-0,0347	0,0349	0,3202	instrument
rs75621580	4	12916514	A	G	0,0002	30,7689	0,0425	0,0077	2,9E-08	0,0192	0,0164	0,2415	instrument
rs7626449	3	70921329	A	G	0,0002	39,4254	-0,0225	0,0036	3,4E-10	0,0142	0,0069	0,0390	instrument
rs7641375	3	57568444	A	C	0,0002	43,0725	-0,0219	0,0033	5,3E-11	-0,0035	0,0064	0,5840	instrument
rs7689420	4	145568352	T	C	0,0008	162,5608	0,0548	0,0043	3,4E-37	0,0012	0,0084	0,8830	instrument
rs7697556	4	73515313	T	C	0,0005	96,6869	-0,0318	0,0032	8,2E-23	0,0071	0,0065	0,2714	instrument
rs7718768	5	178536089	A	G	0,0003	62,8525	-0,0271	0,0034	2,2E-15	0,0080	0,0068	0,2359	instrument
rs7733331	5	32828846	T	C	0,0002	34,2221	-0,0193	0,0033	4,9E-09	-0,0051	0,0067	0,4419	instrument
rs7741741	6	142655801	A	T	0,0005	102,6158	0,0362	0,0036	4,2E-24	0,0004	0,0068	0,9529	instrument
rs7752292	6	131317876	A	T	0,0002	42,4187	-0,0218	0,0033	7,4E-11	-0,0115	0,0064	0,0732	instrument
rs7804293	7	92287849	G	T	0,0004	73,9248	-0,0315	0,0037	8,1E-18	0,0255	0,0069	0,0002	outlier
rs78198962	2	233094868	T	C	0,0002	29,7349	-0,0499	0,0092	5,0E-08	0,0210	0,0239	0,3795	instrument
rs78821460	5	127874149	C	G	0,0002	32,3189	0,0330	0,0058	1,3E-08	-0,0003	0,0113	0,9795	instrument
rs79312203	20	32082962	A	G	0,0002	36,7596	-0,0326	0,0054	1,3E-09	-0,0193	0,0105	0,0657	instrument
rs7952436	11	67024534	T	C	0,0006	119,9012	0,0637	0,0058	6,7E-28	-0,0119	0,0143	0,4048	instrument
rs7994151	13	115038879	G	A	0,0002	41,0364	0,0289	0,0045	1,5E-10	0,0088	0,0087	0,3080	instrument
rs8003703	14	33411639	A	T	0,0002	31,7078	0,0642	0,0114	1,8E-08	0,0063	0,0253	0,8024	instrument
rs80085026	7	148636421	G	C	0,0003	55,0558	-0,0308	0,0042	1,2E-13	0,0306	0,0084	0,0003	outlier
rs8103728	19	33900257	C	G	0,0004	82,6818	0,0313	0,0034	9,7E-20	0,0168	0,0067	0,0125	outlier
rs832540	5	56199202	G	A	0,0002	32,1826	0,0193	0,0034	1,4E-08	0,1027	0,0064	0,0000	outlier
rs849141	7	28185091	A	G	0,0003	66,0149	-0,0289	0,0036	4,5E-16	-0,0088	0,0069	0,1996	instrument
rs862041	14	74986403	T	C	0,0003	50,5374	0,0240	0,0034	1,2E-12	-0,0010	0,0066	0,8769	instrument
rs9346545	6	168806788	C	A	0,0002	29,9315	0,0197	0,0036	4,5E-08	-0,0054	0,0068	0,4289	instrument
rs9358912	6	26211146	T	G	0,0012	232,2552	0,0554	0,0036	2,0E-52	0,0175	0,0068	0,0097	outlier
rs9391253	6	105367616	T	A	0,0005	93,9339	-0,0335	0,0035	3,3E-22	-0,0018	0,0066	0,7843	instrument
rs9398433	6	116753939	G	T	0,0002	43,0200	-0,0219	0,0033	5,4E-11	-0,0059	0,0064	0,3536	instrument

rs9442571	1	9349611	A	T	0,0002	41,2288	-0,0313	0,0049	1,4E-10	-0,0052	0,0102	0,6119	instrument
rs9496567	6	100602753	A	G	0,0002	30,3267	-0,0208	0,0038	3,7E-08	0,0092	0,0083	0,2687	instrument
rs9523325	13	92051677	T	G	0,0002	39,1496	-0,0258	0,0041	3,9E-10	0,0151	0,0078	0,0519	instrument
rs9614671	22	45839056	A	G	0,0002	29,7676	-0,0221	0,0040	4,9E-08	0,0107	0,0079	0,1753	instrument
rs979131	20	6566965	C	T	0,0002	33,8953	-0,0199	0,0034	5,8E-09	-0,0035	0,0066	0,5928	instrument
rs9809116	3	72397279	G	A	0,0002	35,6763	0,0199	0,0033	2,3E-09	-0,0153	0,0066	0,0206	instrument
rs994014	4	82165790	C	T	0,0005	90,3251	-0,0333	0,0035	2,0E-21	-0,0125	0,0068	0,0663	instrument
rs9943184	1	170784430	T	G	0,0002	36,8809	-0,0294	0,0048	1,3E-09	0,0019	0,0093	0,8342	instrument
rs9965184	18	40776606	T	A	0,0002	33,1657	0,0196	0,0034	8,5E-09	-0,0073	0,0065	0,2672	instrument

Table S28. Single Nucleotide Polymorphisms (SNPs) used as instruments in the Mendelian randomization analyses for the effect of LFR on endometrial cancer based on the genome-wide significance threshold P = 5E-08. R^2 represents the explained variance in the exposure. SNP-specific instrument strength is given by the respective F-statistic. Some of the genetic variants were identified as outliers in the iterative approach and therefore excluded from the analyses.

SNP	CHR	POS	EA	OA	R2	Fstat	β_{LFR}	SE_{LFR}	P_{LFR}	$\beta_{Endometrial}$	$SE_{Endometrial}$	$P_{Endometrial}$	$variant_{Endometrial}$
rs1002436	1	89146852	A	G	0,0002	33,9070	0,0189	0,0032	5,8E-09	0,0291	0,0160	0,0689	instrument
rs1007358	7	46201355	G	A	0,0002	31,5391	-0,0221	0,0039	2,0E-08	-0,0057	0,0198	0,7717	instrument
rs10172196	2	36780549	A	G	0,0003	53,8017	-0,0259	0,0035	2,2E-13	0,0037	0,0173	0,8326	instrument
rs1020048	9	86666993	A	C	0,0002	40,1825	-0,0265	0,0042	2,3E-10	-0,0083	0,0202	0,6811	instrument
rs10445034	16	88840564	T	C	0,0002	38,3776	0,0239	0,0039	5,8E-10	-0,0293	0,0190	0,1230	instrument
rs10773051	12	124510391	T	C	0,0002	39,2999	-0,0246	0,0039	3,6E-10	0,0128	0,0189	0,5003	instrument
rs10894192	11	130266117	T	A	0,0002	44,5418	0,0221	0,0033	2,5E-11	-0,0109	0,0169	0,5210	instrument
rs10901171	9	133427194	C	T	0,0002	43,2039	0,0220	0,0033	4,9E-11	-0,0015	0,0167	0,9280	instrument
rs11049361	12	28284841	T	A	0,0003	64,1915	0,0287	0,0036	1,1E-15	-0,0528	0,0177	0,0028	outlier
rs11104474	12	77647657	A	G	0,0002	30,5032	0,0204	0,0037	3,3E-08	0,0116	0,0180	0,5194	instrument
rs11107120	12	93985482	C	T	0,0003	57,5923	-0,0295	0,0039	3,2E-14	-0,0014	0,0192	0,9423	instrument
rs11205303	1	149906413	C	T	0,0018	340,7680	-0,0605	0,0033	4,8E-76	-0,0151	0,0163	0,3533	instrument
rs112424236	12	51170077	C	T	0,0003	56,0845	-0,0262	0,0035	7,0E-14	-0,0176	0,0174	0,3105	instrument
rs112540634	6	34623905	T	C	0,0006	106,9145	-0,0487	0,0047	4,7E-25	-0,0259	0,0230	0,2602	instrument
rs11259936	15	84580582	A	C	0,0012	235,0064	0,0495	0,0032	5,0E-53	-0,0197	0,0158	0,2121	instrument
rs112635299	14	94838142	T	G	0,0004	70,8115	-0,0942	0,0112	3,9E-17	-0,1230	0,0585	0,0355	instrument
rs113059026	10	32158478	A	C	0,0002	42,1586	0,0233	0,0036	8,4E-11	0,0044	0,0173	0,8008	instrument

rs11466399	1	218591623	A	G	0,0003	63,9993	-0,0287	0,0036	1,3E-15	0,0237	0,0175	0,1756	instrument
rs11666808	19	18383506	T	C	0,0002	46,9631	-0,0230	0,0033	7,2E-12	-0,0076	0,0167	0,6482	instrument
rs116896792	12	122948907	C	T	0,0004	66,9444	0,0457	0,0056	2,8E-16	-0,0624	0,0286	0,0290	instrument
rs11695471	2	25457708	A	T	0,0005	88,6790	0,0324	0,0034	4,7E-21	0,0162	0,0173	0,3500	instrument
rs12103006	16	24726237	A	G	0,0002	31,2925	0,0183	0,0033	2,2E-08	0,0365	0,0160	0,0227	instrument
rs12238852	9	98382084	T	C	0,0002	40,8700	-0,0296	0,0046	1,6E-10	0,0217	0,0229	0,3428	instrument
rs12756815	1	113207243	A	C	0,0003	62,4410	0,0316	0,0040	2,7E-15	0,0020	0,0194	0,9165	instrument
rs12811171	12	580772	T	C	0,0002	44,1290	-0,0225	0,0034	3,1E-11	-0,0105	0,0167	0,5305	instrument
rs13128180	4	56286188	T	G	0,0003	57,4558	-0,0267	0,0035	3,5E-14	0,0042	0,0171	0,8063	instrument
rs1313237	14	60848224	C	A	0,0003	50,8506	-0,0236	0,0033	1,0E-12	-0,0060	0,0164	0,7134	instrument
rs132941	22	38545942	C	T	0,0003	64,2716	-0,0261	0,0033	1,1E-15	0,0071	0,0158	0,6540	instrument
rs13306455	19	7184721	T	C	0,0002	32,1259	-0,0244	0,0043	1,4E-08	-0,0159	0,0210	0,4488	instrument
rs138809270	2	219929394	T	C	0,0002	39,7778	0,0828	0,0131	2,8E-10	-0,1178	0,0653	0,0713	instrument
rs143384	20	34025756	G	A	0,0009	179,8262	-0,0441	0,0033	5,4E-41	0,0593	0,0162	0,0003	outlier
rs143840904	11	2813322	T	C	0,0002	44,7289	0,0842	0,0126	2,3E-11	0,0349	0,0765	0,6476	instrument
rs1452815	8	78123634	G	A	0,0002	30,3818	-0,0197	0,0036	3,6E-08	-0,0005	0,0174	0,9786	instrument
rs1513304	3	99269750	C	T	0,0002	40,5765	-0,0226	0,0035	1,9E-10	0,0180	0,0182	0,3232	instrument
rs1550436	15	74221157	T	C	0,0004	77,3160	0,0286	0,0032	1,5E-18	0,0289	0,0158	0,0686	instrument
rs16942324	15	89383854	A	C	0,0004	70,6937	0,0844	0,0100	4,2E-17	0,0455	0,0551	0,4088	instrument
rs16948048	17	47440466	G	A	0,0002	42,5621	0,0218	0,0033	6,9E-11	0,0077	0,0163	0,6358	instrument
rs16953461	17	46261736	G	T	0,0003	54,8186	0,0292	0,0039	1,3E-13	0,0625	0,0190	0,0010	outlier
rs16972537	16	73802389	A	G	0,0002	33,0736	-0,0194	0,0034	8,9E-09	0,0125	0,0167	0,4543	instrument
rs17698176	17	44819595	G	T	0,0003	62,1883	-0,0307	0,0039	3,1E-15	-0,0050	0,0202	0,8029	instrument
rs17744040	9	16842137	C	T	0,0002	34,5740	0,0309	0,0053	4,1E-09	-0,0112	0,0243	0,6437	instrument
rs1801282	3	12393125	G	C	0,0003	51,5519	-0,0356	0,0050	7,0E-13	-0,0737	0,0242	0,0024	outlier
rs1980030	10	120970027	G	A	0,0002	32,0806	-0,0186	0,0033	1,5E-08	0,0041	0,0164	0,8039	instrument
rs2007482	6	2169724	T	C	0,0002	36,6626	0,0199	0,0033	1,4E-09	-0,0143	0,0166	0,3889	instrument
rs2058908	16	53806145	T	C	0,0002	44,3951	0,0243	0,0037	2,7E-11	-0,0173	0,0184	0,3463	instrument
rs2126643	1	103404384	T	C	0,0002	40,1318	-0,0218	0,0034	2,4E-10	0,0087	0,0167	0,6027	instrument
rs2158694	7	2863443	G	T	0,0008	161,7967	0,0450	0,0035	4,5E-37	-0,0030	0,0173	0,8632	instrument
rs2173295	1	54943264	C	A	0,0002	33,0737	0,0276	0,0048	8,9E-09	0,0379	0,0245	0,1226	instrument

rs2274432	1	184020945	A	G	0,0005	86,6007	-0,0315	0,0034	1,3E-20	-0,0188	0,0166	0,2565	instrument
rs2293576	11	47434986	A	G	0,0003	48,9295	0,0240	0,0034	2,7E-12	-0,0058	0,0168	0,7307	instrument
rs2370983	14	79903376	G	A	0,0002	33,4890	0,0196	0,0034	7,2E-09	-0,0480	0,0171	0,0049	outlier
rs2516633	11	62371773	A	C	0,0002	31,4718	0,0197	0,0035	2,0E-08	-0,0226	0,0177	0,2020	instrument
rs256904	5	55810305	A	T	0,0003	66,6012	-0,0302	0,0037	3,3E-16	-0,0090	0,0187	0,6298	instrument
rs2641699	16	84886306	C	G	0,0002	35,6047	0,0200	0,0034	2,4E-09	0,0237	0,0170	0,1639	instrument
rs264762	5	64384386	A	C	0,0003	52,4171	0,0248	0,0034	4,5E-13	0,0157	0,0169	0,3527	instrument
rs2712169	2	217671349	G	A	0,0003	49,6879	-0,0233	0,0033	1,8E-12	0,0061	0,0162	0,7068	instrument
rs2715094	7	50730452	G	A	0,0003	57,7290	-0,0282	0,0037	3,0E-14	-0,0014	0,0187	0,9384	instrument
rs2768641	9	96948863	G	A	0,0002	34,4213	-0,0455	0,0078	4,4E-09	-0,0210	0,0404	0,6026	instrument
rs2811462	3	129303436	G	T	0,0003	52,3447	-0,0281	0,0039	4,7E-13	0,0104	0,0194	0,5909	instrument
rs2820446	1	219748818	G	C	0,0007	132,7090	-0,0405	0,0035	1,1E-30	-0,0531	0,0176	0,0026	outlier
rs2844795	6	30073847	C	T	0,0002	41,4345	-0,0208	0,0032	1,2E-10	-0,0219	0,0163	0,1796	instrument
rs28539246	17	29226630	A	T	0,0003	48,5804	0,0231	0,0033	3,2E-12	-0,0173	0,0162	0,2860	instrument
rs3020421	6	152348122	A	G	0,0002	35,0224	-0,0206	0,0035	3,3E-09	0,0699	0,0167	2,7E-05	outlier
rs3218036	19	30305684	A	G	0,0002	29,8550	-0,0188	0,0034	4,7E-08	0,0018	0,0169	0,9138	instrument
rs34517439	1	78450517	A	C	0,0003	65,2049	-0,0403	0,0050	6,8E-16	0,0424	0,0254	0,0947	instrument
rs34580448	5	82810884	C	T	0,0004	79,5477	0,0727	0,0081	4,8E-19	-0,0185	0,0421	0,6603	instrument
rs35307904	9	78511889	A	G	0,0006	116,4229	0,0537	0,0050	3,8E-27	0,0094	0,0256	0,7133	instrument
rs35874463	15	67457698	G	A	0,0003	66,0962	-0,0563	0,0069	4,3E-16	0,0717	0,0367	0,0509	instrument
rs3738814	1	17331676	G	A	0,0003	60,4500	0,0253	0,0033	7,6E-15	0,0016	0,0158	0,9207	instrument
rs3791679	2	56096892	G	A	0,0009	179,2902	0,0516	0,0039	7,3E-41	-0,0347	0,0187	0,0631	instrument
rs3794782	17	18127914	C	G	0,0002	33,0277	-0,0191	0,0033	9,1E-09	-0,0123	0,0163	0,4495	instrument
rs3823974	7	20442796	C	T	0,0003	60,9486	0,0258	0,0033	5,9E-15	-0,0153	0,0160	0,3387	instrument
rs3850639	1	172253634	C	T	0,0003	56,8058	-0,0297	0,0039	4,8E-14	-0,0356	0,0192	0,0643	instrument
rs4073717	5	170864021	T	G	0,0002	33,2233	0,0233	0,0040	8,2E-09	-0,0330	0,0201	0,1008	instrument
rs41284816	13	50655989	T	G	0,0002	46,3484	-0,0835	0,0123	9,9E-12	0,0784	0,0530	0,1389	instrument
rs41313250	1	62483623	G	A	0,0002	36,9903	0,0319	0,0052	1,2E-09	-0,0030	0,0252	0,9066	instrument
rs4236324	7	27302727	C	T	0,0002	37,5030	-0,0274	0,0045	9,1E-10	-0,0061	0,0221	0,7805	instrument
rs4321401	2	69348216	T	C	0,0003	48,9715	0,0227	0,0032	2,6E-12	-0,0226	0,0168	0,1785	instrument
rs4421120	5	32763118	A	G	0,0003	50,1542	-0,0311	0,0044	1,4E-12	-0,0467	0,0221	0,0350	instrument

rs4432932	5	115039414	G	A	0,0002	31,1249	-0,0204	0,0037	2,4E-08	0,0269	0,0176	0,1257	instrument
rs4518624	8	130715510	A	G	0,0002	38,0685	0,0203	0,0033	6,8E-10	0,0079	0,0160	0,6223	instrument
rs454345	5	88729851	G	A	0,0002	33,8255	-0,0194	0,0033	6,0E-09	0,0255	0,0165	0,1208	instrument
rs4803461	19	41908871	G	A	0,0002	33,2003	-0,0190	0,0033	8,3E-09	0,0050	0,0165	0,7627	instrument
rs4842133	9	139127566	A	C	0,0004	69,4715	-0,0300	0,0036	7,8E-17	-0,0040	0,0183	0,8269	instrument
rs4901541	14	55235282	C	T	0,0002	40,4873	0,0211	0,0033	2,0E-10	0,0056	0,0169	0,7412	instrument
rs4936122	11	130734264	C	A	0,0002	30,3818	-0,0181	0,0033	3,6E-08	-0,0005	0,0163	0,9765	instrument
rs496298	10	131355130	A	G	0,0003	65,4151	0,0276	0,0034	6,1E-16	0,0033	0,0167	0,8417	instrument
rs4973032	2	232990462	T	G	0,0002	40,7423	0,0769	0,0121	1,7E-10	-0,0093	0,0567	0,8693	instrument
rs4974072	3	41222586	A	G	0,0002	43,3091	-0,0214	0,0033	4,7E-11	0,0507	0,0159	0,0014	outlier
rs538656	18	57850422	T	G	0,0002	31,4269	-0,0213	0,0038	2,1E-08	0,0134	0,0185	0,4710	instrument
rs544148	6	117466494	A	G	0,0002	30,7134	0,0182	0,0033	3,0E-08	-0,0115	0,0161	0,4738	instrument
rs55633655	5	131598756	T	G	0,0003	53,1144	0,0237	0,0033	3,2E-13	0,0009	0,0161	0,9545	instrument
rs56021161	14	64817100	A	T	0,0002	30,5916	0,0362	0,0065	3,2E-08	0,0288	0,0332	0,3849	instrument
rs56186137	16	28825953	G	A	0,0006	108,1589	-0,0341	0,0033	2,6E-25	0,0542	0,0161	0,0008	outlier
rs56277550	21	18092263	A	G	0,0002	33,5006	0,0187	0,0032	7,1E-09	-0,0060	0,0162	0,7117	instrument
rs57674902	20	4096741	T	C	0,0002	38,1302	-0,0210	0,0034	6,6E-10	0,0242	0,0172	0,1598	instrument
rs584961	11	75277628	A	G	0,0002	35,1408	-0,0302	0,0051	3,1E-09	0,0012	0,0262	0,9628	instrument
rs61734651	20	61451332	T	C	0,0002	37,8836	-0,0421	0,0068	7,5E-10	0,0270	0,0370	0,4652	instrument
rs61834590	1	227421117	G	T	0,0002	43,2696	0,0298	0,0045	4,8E-11	-0,0400	0,0220	0,0695	instrument
rs62106258	2	417167	C	T	0,0002	32,2053	0,0424	0,0075	1,4E-08	-0,1133	0,0461	0,0140	instrument
rs62271373	3	150066540	A	T	0,0002	41,1775	0,0455	0,0071	1,4E-10	-0,0181	0,0386	0,6400	instrument
rs62515452	8	57187356	T	C	0,0002	34,2338	-0,0267	0,0046	4,9E-09	0,0159	0,0240	0,5055	instrument
rs62621197	19	8670147	T	C	0,0008	150,7968	0,1140	0,0093	1,1E-34	-0,0510	0,0583	0,3809	instrument
rs640245	11	30393360	T	C	0,0002	37,0512	-0,0198	0,0033	1,1E-09	-0,0131	0,0159	0,4105	instrument
rs6486059	11	12824581	A	G	0,0003	52,4315	-0,0238	0,0033	4,5E-13	0,0098	0,0163	0,5473	instrument
rs6503410	17	43094140	T	C	0,0002	35,0934	0,0215	0,0036	3,2E-09	-0,0155	0,0177	0,3807	instrument
rs6585827	10	124165615	A	G	0,0002	45,0505	-0,0217	0,0032	1,9E-11	0,0013	0,0161	0,9358	instrument
rs6717858	2	165539661	C	T	0,0003	64,7696	-0,0265	0,0033	8,5E-16	-0,0072	0,0161	0,6559	instrument
rs6739701	2	105132332	G	A	0,0002	44,8493	-0,0220	0,0033	2,1E-11	0,0166	0,0170	0,3298	instrument
rs6785012	3	141109348	T	C	0,0012	231,9505	-0,0496	0,0033	2,3E-52	0,0343	0,0159	0,0307	instrument

rs68049170	10	72432047	A	G	0,0003	56,1594	-0,0273	0,0036	6,7E-14	0,0047	0,0178	0,7933	instrument
rs6843340	4	48619937	T	C	0,0002	30,9912	-0,0180	0,0032	2,6E-08	0,0133	0,0158	0,3999	instrument
rs6887247	5	157881647	C	T	0,0003	53,8897	-0,0257	0,0035	2,1E-13	-0,0251	0,0173	0,1478	instrument
rs701006	12	58106836	A	G	0,0002	32,5353	0,0188	0,0033	1,2E-08	0,0012	0,0160	0,9422	instrument
rs702605	5	53167028	T	C	0,0002	34,1286	-0,0189	0,0032	5,2E-09	-0,0088	0,0157	0,5754	instrument
rs704067	12	89726027	A	G	0,0003	50,7508	-0,0232	0,0033	1,0E-12	0,0070	0,0170	0,6815	instrument
rs71531541	6	7066481	C	T	0,0002	30,1727	0,0200	0,0036	4,0E-08	0,0045	0,0181	0,8031	instrument
rs7155279	14	92485881	T	G	0,0003	47,9967	0,0233	0,0034	4,3E-12	0,0016	0,0165	0,9219	instrument
rs7170686	15	48821350	A	G	0,0002	36,2761	0,0334	0,0055	1,7E-09	-0,0107	0,0283	0,7050	instrument
rs7191697	16	89678165	A	G	0,0002	46,8946	0,0369	0,0054	7,5E-12	-0,0252	0,0282	0,3731	instrument
rs7234167	18	46744610	C	G	0,0002	30,4480	0,0206	0,0037	3,4E-08	0,0058	0,0184	0,7531	instrument
rs7244464	18	20729714	A	C	0,0005	90,7247	0,0372	0,0039	1,7E-21	0,0235	0,0193	0,2231	instrument
rs72708236	1	155980320	G	T	0,0003	54,7890	0,0703	0,0095	1,3E-13	-0,0252	0,0505	0,6176	instrument
rs72755233	15	100692953	A	G	0,0008	160,7807	0,0645	0,0051	7,6E-37	-0,0123	0,0309	0,6900	instrument
rs72776438	5	77438797	T	G	0,0002	36,1798	0,0228	0,0038	1,8E-09	0,0151	0,0199	0,4471	instrument
rs73004967	19	19717056	G	A	0,0002	40,6530	0,0405	0,0064	1,8E-10	0,0037	0,0306	0,9040	instrument
rs73175572	3	185490184	G	A	0,0002	36,0236	-0,0314	0,0052	2,0E-09	-0,0548	0,0264	0,0377	instrument
rs732563	8	23345526	T	C	0,0002	41,4088	0,0208	0,0032	1,2E-10	0,0338	0,0157	0,0317	instrument
rs741207	4	18321092	T	C	0,0002	35,8677	-0,0200	0,0033	2,1E-09	-0,0111	0,0162	0,4926	instrument
rs7502307	17	65989961	G	C	0,0003	54,1543	-0,0300	0,0041	1,9E-13	0,0864	0,0200	1,6E-05	outlier
rs75223147	6	19831020	G	T	0,0003	56,7154	-0,0848	0,0113	5,1E-14	0,0378	0,0821	0,6450	instrument
rs75621580	4	12916514	A	G	0,0002	30,7689	0,0425	0,0077	2,9E-08	0,0077	0,0394	0,8458	instrument
rs7626449	3	70921329	A	G	0,0002	39,4254	-0,0225	0,0036	3,4E-10	-0,0164	0,0175	0,3463	instrument
rs7641375	3	57568444	A	C	0,0002	43,0725	-0,0219	0,0033	5,3E-11	-0,0243	0,0166	0,1449	instrument
rs7689420	4	145568352	T	C	0,0008	162,5608	0,0548	0,0043	3,4E-37	0,0183	0,0214	0,3913	instrument
rs7697556	4	73515313	T	C	0,0005	96,6869	-0,0318	0,0032	8,2E-23	-0,0166	0,0161	0,3046	instrument
rs7718768	5	178536089	A	G	0,0003	62,8525	-0,0271	0,0034	2,2E-15	-0,0255	0,0170	0,1347	instrument
rs7733331	5	32828846	T	C	0,0002	34,2221	-0,0193	0,0033	4,9E-09	0,0162	0,0166	0,3293	instrument
rs7741741	6	142655801	A	T	0,0005	102,6158	0,0362	0,0036	4,2E-24	0,0102	0,0175	0,5600	instrument
rs7752292	6	131317876	A	T	0,0002	42,4187	-0,0218	0,0033	7,4E-11	-0,0230	0,0164	0,1603	instrument
rs7804293	7	92287849	G	T	0,0004	73,9248	-0,0315	0,0037	8,1E-18	-0,0079	0,0178	0,6595	instrument

rs78198962	2	233094868	T	C	0,0002	29,7349	-0,0499	0,0092	5,0E-08	0,0972	0,0563	0,0841	instrument
rs78821460	5	127874149	C	G	0,0002	32,3189	0,0330	0,0058	1,3E-08	-0,0113	0,0287	0,6926	instrument
rs79312203	20	32082962	A	G	0,0002	36,7596	-0,0326	0,0054	1,3E-09	0,0097	0,0271	0,7204	instrument
rs7952436	11	67024534	T	C	0,0006	119,9012	0,0637	0,0058	6,7E-28	-0,0637	0,0349	0,0677	instrument
rs7959830	12	66347368	T	G	0,0004	83,9597	0,0301	0,0033	5,1E-20	-0,0053	0,0162	0,7440	instrument
rs7994151	13	115038879	G	A	0,0002	41,0364	0,0289	0,0045	1,5E-10	-0,0140	0,0223	0,5302	instrument
rs8003703	14	33411639	A	T	0,0002	31,7078	0,0642	0,0114	1,8E-08	0,0672	0,0609	0,2693	instrument
rs80085026	7	148636421	G	C	0,0003	55,0558	-0,0308	0,0042	1,2E-13	0,0162	0,0208	0,4345	instrument
rs8103728	19	33900257	C	G	0,0004	82,6818	0,0313	0,0034	9,7E-20	0,0222	0,0170	0,1911	instrument
rs832540	5	56199202	G	A	0,0002	32,1826	0,0193	0,0034	1,4E-08	-0,0488	0,0165	0,0031	outlier
rs849141	7	28185091	A	G	0,0003	66,0149	-0,0289	0,0036	4,5E-16	-0,0096	0,0174	0,5826	instrument
rs862041	14	74986403	T	C	0,0003	50,5374	0,0240	0,0034	1,2E-12	0,0084	0,0166	0,6119	instrument
rs9346545	6	168806788	C	A	0,0002	29,9315	0,0197	0,0036	4,5E-08	-0,0100	0,0174	0,5653	instrument
rs9358912	6	26211146	T	G	0,0012	232,2552	0,0554	0,0036	2,0E-52	0,0180	0,0174	0,2991	instrument
rs9391253	6	105367616	T	A	0,0005	93,9339	-0,0335	0,0035	3,3E-22	0,0204	0,0168	0,2262	instrument
rs9398433	6	116753939	G	T	0,0002	43,0200	-0,0219	0,0033	5,4E-11	-0,0026	0,0162	0,8729	instrument
rs9442571	1	9349611	A	T	0,0002	41,2288	-0,0313	0,0049	1,4E-10	-0,0128	0,0251	0,6097	instrument
rs9496567	6	100602753	A	G	0,0002	30,3267	-0,0208	0,0038	3,7E-08	-0,0140	0,0202	0,4866	instrument
rs9523325	13	92051677	T	G	0,0002	39,1496	-0,0258	0,0041	3,9E-10	-0,0019	0,0196	0,9227	instrument
rs9614671	22	45839056	A	G	0,0002	29,7676	-0,0221	0,0040	4,9E-08	-0,0081	0,0199	0,6825	instrument
rs979131	20	6566965	C	T	0,0002	33,8953	-0,0199	0,0034	5,8E-09	-0,0283	0,0166	0,0885	instrument
rs9809116	3	72397279	G	A	0,0002	35,6763	0,0199	0,0033	2,3E-09	0,0057	0,0166	0,7328	instrument
rs994014	4	82165790	C	T	0,0005	90,3251	-0,0333	0,0035	2,0E-21	-0,0178	0,0172	0,2989	instrument
rs9943184	1	170784430	T	G	0,0002	36,8809	-0,0294	0,0048	1,3E-09	0,0162	0,0235	0,4900	instrument
rs9965184	18	40776606	T	A	0,0002	33,1657	0,0196	0,0034	8,5E-09	-0,0256	0,0168	0,1267	instrument

Table S29. Single Nucleotide Polymorphisms (SNPs) used as instruments in the Mendelian randomization analyses for the effect of LFR on ovarian cancer based on the genome-wide significance threshold P = 5E-08. R^2 represents the explained variance in the exposure. SNP-specific instrument strength is given by the respective F-statistic. Some of the genetic variants were identified as outliers in the iterative approach and therefore excluded from the analyses.

SNP	CHR	POS	EA	OA	R2	Fstat	β_{LFR}	SE_{LFR}	P_{LFR}	$\beta_{Ovarian}$	$SE_{Ovarian}$	$P_{Ovarian}$	$variant_{Ovarian}$
rs1002436	1	89146852	A	G	0,0002	33,9070	0,0189	0,0032	5,8E-09	-0,0093	0,0134	0,4867	instrument
rs1007358	7	46201355	G	A	0,0002	31,5391	-0,0221	0,0039	2,0E-08	0,0157	0,0163	0,3351	instrument
rs10172196	2	36780549	A	G	0,0003	53,8017	-0,0259	0,0035	2,2E-13	-0,0032	0,0146	0,8240	instrument
rs1020048	9	86666993	A	C	0,0002	40,1825	-0,0265	0,0042	2,3E-10	-0,0139	0,0168	0,4069	instrument
rs10445034	16	88840564	T	C	0,0002	38,3776	0,0239	0,0039	5,8E-10	-0,0154	0,0158	0,3301	instrument
rs10901171	9	133427194	C	T	0,0002	43,2039	0,0220	0,0033	4,9E-11	0,0161	0,0140	0,2503	instrument
rs11205303	1	149906413	C	T	0,0018	340,7680	-0,0605	0,0033	4,8E-76	-0,0053	0,0135	0,6933	instrument
rs112540634	6	34623905	T	C	0,0006	106,9145	-0,0487	0,0047	4,7E-25	0,0194	0,0188	0,3035	instrument
rs11259936	15	84580582	A	C	0,0012	235,0064	0,0495	0,0032	5,0E-53	-0,0121	0,0132	0,3576	instrument
rs112635299	14	94838142	T	G	0,0004	70,8115	-0,0942	0,0112	3,9E-17	0,0039	0,0498	0,9382	instrument
rs113059026	10	32158478	A	C	0,0002	42,1586	0,0233	0,0036	8,4E-11	-0,0076	0,0146	0,5999	instrument
rs11466399	1	218591623	A	G	0,0003	63,9993	-0,0287	0,0036	1,3E-15	-0,0011	0,0146	0,9386	instrument
rs11666808	19	18383506	T	C	0,0002	46,9631	-0,0230	0,0033	7,2E-12	0,0190	0,0138	0,1690	instrument
rs11695471	2	25457708	A	T	0,0005	88,6790	0,0324	0,0034	4,7E-21	-0,0270	0,0146	0,0643	instrument
rs12103006	16	24726237	A	G	0,0002	31,2925	0,0183	0,0033	2,2E-08	0,0049	0,0134	0,7165	instrument
rs12238852	9	98382084	T	C	0,0002	40,8700	-0,0296	0,0046	1,6E-10	-0,0181	0,0191	0,3439	instrument
rs12756815	1	113207243	A	C	0,0003	62,4410	0,0316	0,0040	2,7E-15	-0,0248	0,0163	0,1286	instrument
rs13128180	4	56286188	T	G	0,0003	57,4558	-0,0267	0,0035	3,5E-14	0,0302	0,0142	0,0337	instrument
rs1313237	14	60848224	C	A	0,0003	50,8506	-0,0236	0,0033	1,0E-12	-0,0146	0,0136	0,2834	instrument
rs132941	22	38545942	C	T	0,0003	64,2716	-0,0261	0,0033	1,1E-15	-0,0119	0,0133	0,3696	instrument
rs13306455	19	7184721	T	C	0,0002	32,1259	-0,0244	0,0043	1,4E-08	-0,0233	0,0176	0,1867	instrument
rs138809270	2	219929394	T	C	0,0002	39,7778	0,0828	0,0131	2,8E-10	-0,0596	0,0522	0,2534	instrument
rs143384	20	34025756	G	A	0,0009	179,8262	-0,0441	0,0033	5,4E-41	0,0061	0,0134	0,6497	instrument
rs143840904	11	2813322	T	C	0,0002	44,7289	0,0842	0,0126	2,3E-11	-0,0747	0,0653	0,2527	instrument
rs1452815	8	78123634	G	A	0,0002	30,3818	-0,0197	0,0036	3,6E-08	-0,0168	0,0147	0,2523	instrument
rs1513304	3	99269750	C	T	0,0002	40,5765	-0,0226	0,0035	1,9E-10	-0,0004	0,0154	0,9786	instrument
rs1550436	15	74221157	T	C	0,0004	77,3160	0,0286	0,0032	1,5E-18	0,0111	0,0132	0,4019	instrument

rs16942324	15	89383854	A	C	0,0004	70,6937	0,0844	0,0100	4,2E-17	-0,0291	0,0446	0,5138	instrument
rs16948048	17	47440466	G	A	0,0002	42,5621	0,0218	0,0033	6,9E-11	-0,0208	0,0136	0,1259	instrument
rs16953461	17	46261736	G	T	0,0003	54,8186	0,0292	0,0039	1,3E-13	-0,0319	0,0167	0,0552	instrument
rs16972537	16	73802389	A	G	0,0002	33,0736	-0,0194	0,0034	8,9E-09	0,0141	0,0141	0,3156	instrument
rs17698176	17	44819595	G	T	0,0003	62,1883	-0,0307	0,0039	3,1E-15	-0,0232	0,0179	0,1935	instrument
rs17744040	9	16842137	C	T	0,0002	34,5740	0,0309	0,0053	4,1E-09	-0,1207	0,0205	3,6E-09	outlier
rs1801282	3	12393125	G	C	0,0003	51,5519	-0,0356	0,0050	7,0E-13	-0,0072	0,0200	0,7188	instrument
rs1980030	10	120970027	G	A	0,0002	32,0806	-0,0186	0,0033	1,5E-08	0,0010	0,0138	0,9431	instrument
rs2007482	6	2169724	T	C	0,0002	36,6626	0,0199	0,0033	1,4E-09	0,0025	0,0137	0,8580	instrument
rs2058908	16	53806145	T	C	0,0002	44,3951	0,0243	0,0037	2,7E-11	-0,0210	0,0153	0,1683	instrument
rs2126643	1	103404384	T	C	0,0002	40,1318	-0,0218	0,0034	2,4E-10	0,0006	0,0139	0,9671	instrument
rs2158694	7	2863443	G	T	0,0008	161,7967	0,0450	0,0035	4,5E-37	-0,0182	0,0145	0,2109	instrument
rs2173295	1	54943264	C	A	0,0002	33,0737	0,0276	0,0048	8,9E-09	-0,0164	0,0207	0,4286	instrument
rs2274432	1	184020945	A	G	0,0005	86,6007	-0,0315	0,0034	1,3E-20	0,0027	0,0139	0,8468	instrument
rs2293576	11	47434986	A	G	0,0003	48,9295	0,0240	0,0034	2,7E-12	0,0111	0,0140	0,4281	instrument
rs2370983	14	79903376	G	A	0,0002	33,4890	0,0196	0,0034	7,2E-09	0,0097	0,0142	0,4957	instrument
rs2516633	11	62371773	A	C	0,0002	31,4718	0,0197	0,0035	2,0E-08	-0,0197	0,0151	0,1926	instrument
rs256904	5	55810305	A	T	0,0003	66,6012	-0,0302	0,0037	3,3E-16	0,0104	0,0154	0,4984	instrument
rs2641699	16	84886306	C	G	0,0002	35,6047	0,0200	0,0034	2,4E-09	0,0080	0,0142	0,5717	instrument
rs264762	5	64384386	A	C	0,0003	52,4171	0,0248	0,0034	4,5E-13	-0,0157	0,0140	0,2623	instrument
rs2712169	2	217671349	G	A	0,0003	49,6879	-0,0233	0,0033	1,8E-12	0,0183	0,0134	0,1745	instrument
rs2715094	7	50730452	G	A	0,0003	57,7290	-0,0282	0,0037	3,0E-14	0,0228	0,0157	0,1459	instrument
rs2768641	9	96948863	G	A	0,0002	34,4213	-0,0455	0,0078	4,4E-09	-0,0014	0,0329	0,9659	instrument
rs2811462	3	129303436	G	T	0,0003	52,3447	-0,0281	0,0039	4,7E-13	0,0241	0,0161	0,1343	instrument
rs2820446	1	219748818	G	C	0,0007	132,7090	-0,0405	0,0035	1,1E-30	0,0006	0,0147	0,9669	instrument
rs2844795	6	30073847	C	T	0,0002	41,4345	-0,0208	0,0032	1,2E-10	0,0082	0,0134	0,5404	instrument
rs28539246	17	29226630	A	T	0,0003	48,5804	0,0231	0,0033	3,2E-12	-0,0413	0,0135	0,0022	outlier
rs28716235	8	135602976	C	A	0,0002	33,4427	0,0194	0,0034	7,4E-09	-0,0328	0,0137	0,0169	instrument
rs2886564	1	42496924	G	A	0,0002	31,4381	-0,0200	0,0036	2,1E-08	-0,0076	0,0145	0,5995	instrument
rs3020421	6	152348122	A	G	0,0002	35,0224	-0,0206	0,0035	3,3E-09	0,0142	0,0141	0,3127	instrument
rs3218036	19	30305684	A	G	0,0002	29,8550	-0,0188	0,0034	4,7E-08	0,0480	0,0140	0,0006	outlier

rs34517439	1	78450517	A	C	0,0003	65,2049	-0,0403	0,0050	6,8E-16	-0,0016	0,0216	0,9426	instrument
rs34580448	5	82810884	C	T	0,0004	79,5477	0,0727	0,0081	4,8E-19	0,0017	0,0342	0,9613	instrument
rs35307904	9	78511889	A	G	0,0006	116,4229	0,0537	0,0050	3,8E-27	-0,0092	0,0218	0,6741	instrument
rs35874463	15	67457698	G	A	0,0003	66,0962	-0,0563	0,0069	4,3E-16	-0,0411	0,0301	0,1720	instrument
rs3738814	1	17331676	G	A	0,0003	60,4500	0,0253	0,0033	7,6E-15	-0,0361	0,0133	0,0066	outlier
rs3791679	2	56096892	G	A	0,0009	179,2902	0,0516	0,0039	7,3E-41	0,0115	0,0154	0,4535	instrument
rs3794782	17	18127914	C	G	0,0002	33,0277	-0,0191	0,0033	9,1E-09	-0,0156	0,0136	0,2500	instrument
rs3823974	7	20442796	C	T	0,0003	60,9486	0,0258	0,0033	5,9E-15	-0,0108	0,0134	0,4176	instrument
rs3850639	1	172253634	C	T	0,0003	56,8058	-0,0297	0,0039	4,8E-14	0,0175	0,0160	0,2742	instrument
rs4073717	5	170864021	T	G	0,0002	33,2233	0,0233	0,0040	8,2E-09	0,0080	0,0167	0,6313	instrument
rs41284816	13	50655989	T	G	0,0002	46,3484	-0,0835	0,0123	9,9E-12	-0,0167	0,0453	0,7121	instrument
rs41313250	1	62483623	G	A	0,0002	36,9903	0,0319	0,0052	1,2E-09	-0,0193	0,0202	0,3414	instrument
rs4236324	7	27302727	C	T	0,0002	37,5030	-0,0274	0,0045	9,1E-10	-0,0179	0,0185	0,3315	instrument
rs4246308	15	100765202	A	G	0,0003	59,4589	0,0250	0,0032	1,3E-14	0,0066	0,0132	0,6171	instrument
rs4321401	2	69348216	T	C	0,0003	48,9715	0,0227	0,0032	2,6E-12	0,0015	0,0143	0,9167	instrument
rs4421120	5	32763118	A	G	0,0003	50,1542	-0,0311	0,0044	1,4E-12	-0,0054	0,0185	0,7718	instrument
rs4432932	5	115039414	G	A	0,0002	31,1249	-0,0204	0,0037	2,4E-08	-0,0043	0,0146	0,7674	instrument
rs4518624	8	130715510	A	G	0,0002	38,0685	0,0203	0,0033	6,8E-10	0,0144	0,0135	0,2870	instrument
rs454345	5	88729851	G	A	0,0002	33,8255	-0,0194	0,0033	6,0E-09	-0,0138	0,0138	0,3150	instrument
rs4803461	19	41908871	G	A	0,0002	33,2003	-0,0190	0,0033	8,3E-09	0,0007	0,0137	0,9595	instrument
rs4842133	9	139127566	A	C	0,0004	69,4715	-0,0300	0,0036	7,8E-17	-0,0110	0,0152	0,4679	instrument
rs4901541	14	55235282	C	T	0,0002	40,4873	0,0211	0,0033	2,0E-10	0,0037	0,0142	0,7938	instrument
rs4936122	11	130734264	C	A	0,0002	30,3818	-0,0181	0,0033	3,6E-08	-0,0081	0,0137	0,5549	instrument
rs496298	10	131355130	A	G	0,0003	65,4151	0,0276	0,0034	6,1E-16	-0,0312	0,0140	0,0256	instrument
rs4973032	2	232990462	T	G	0,0002	40,7423	0,0769	0,0121	1,7E-10	-0,0543	0,0477	0,2548	instrument
rs4974072	3	41222586	A	G	0,0002	43,3091	-0,0214	0,0033	4,7E-11	-0,0046	0,0133	0,7292	instrument
rs538656	18	57850422	T	G	0,0002	31,4269	-0,0213	0,0038	2,1E-08	-0,0032	0,0155	0,8348	instrument
rs544148	6	117466494	A	G	0,0002	30,7134	0,0182	0,0033	3,0E-08	0,0193	0,0135	0,1509	instrument
rs55633655	5	131598756	T	G	0,0003	53,1144	0,0237	0,0033	3,2E-13	-0,0104	0,0132	0,4321	instrument
rs56021161	14	64817100	A	T	0,0002	30,5916	0,0362	0,0065	3,2E-08	0,0169	0,0274	0,5383	instrument
rs56186137	16	28825953	G	A	0,0006	108,1589	-0,0341	0,0033	2,6E-25	0,0059	0,0136	0,6641	instrument

rs56277550	21	18092263	A	G	0,0002	33,5006	0,0187	0,0032	7,1E-09	-0,0122	0,0135	0,3660	instrument
rs57674902	20	4096741	T	C	0,0002	38,1302	-0,0210	0,0034	6,6E-10	-0,0042	0,0144	0,7725	instrument
rs584961	11	75277628	A	G	0,0002	35,1408	-0,0302	0,0051	3,1E-09	0,0111	0,0220	0,6126	instrument
rs61734651	20	61451332	T	C	0,0002	37,8836	-0,0421	0,0068	7,5E-10	-0,0351	0,0303	0,2474	instrument
rs61834590	1	227421117	G	T	0,0002	43,2696	0,0298	0,0045	4,8E-11	-0,0114	0,0186	0,5393	instrument
rs62106258	2	417167	C	T	0,0002	32,2053	0,0424	0,0075	1,4E-08	-0,0406	0,0360	0,2584	instrument
rs62271373	3	150066540	A	T	0,0002	41,1775	0,0455	0,0071	1,4E-10	-0,0333	0,0311	0,2850	instrument
rs62515452	8	57187356	T	C	0,0002	34,2338	-0,0267	0,0046	4,9E-09	-0,0076	0,0202	0,7068	instrument
rs62621197	19	8670147	T	C	0,0008	150,7968	0,1140	0,0093	1,1E-34	0,0250	0,0449	0,5772	instrument
rs640245	11	30393360	T	C	0,0002	37,0512	-0,0198	0,0033	1,1E-09	-0,0193	0,0133	0,1481	instrument
rs6486059	11	12824581	A	G	0,0003	52,4315	-0,0238	0,0033	4,5E-13	-0,0210	0,0137	0,1237	instrument
rs6503410	17	43094140	T	C	0,0002	35,0934	0,0215	0,0036	3,2E-09	0,0259	0,0150	0,0857	instrument
rs6585827	10	124165615	A	G	0,0002	45,0505	-0,0217	0,0032	1,9E-11	0,0151	0,0134	0,2582	instrument
rs6717858	2	165539661	C	T	0,0003	64,7696	-0,0265	0,0033	8,5E-16	-0,0187	0,0135	0,1642	instrument
rs6739701	2	105132332	G	A	0,0002	44,8493	-0,0220	0,0033	2,1E-11	-0,0160	0,0141	0,2569	instrument
rs6785012	3	141109348	T	C	0,0012	231,9505	-0,0496	0,0033	2,3E-52	-0,0060	0,0133	0,6519	instrument
rs68049170	10	72432047	A	G	0,0003	56,1594	-0,0273	0,0036	6,7E-14	0,0134	0,0149	0,3694	instrument
rs6843340	4	48619937	T	C	0,0002	30,9912	-0,0180	0,0032	2,6E-08	0,0057	0,0132	0,6656	instrument
rs6887247	5	157881647	C	T	0,0003	53,8897	-0,0257	0,0035	2,1E-13	0,0262	0,0145	0,0710	instrument
rs702605	5	53167028	T	C	0,0002	34,1286	-0,0189	0,0032	5,2E-09	-0,0099	0,0132	0,4531	instrument
rs71531541	6	7066481	C	T	0,0002	30,1727	0,0200	0,0036	4,0E-08	0,0076	0,0151	0,6154	instrument
rs7155279	14	92485881	T	G	0,0003	47,9967	0,0233	0,0034	4,3E-12	0,0024	0,0137	0,8609	instrument
rs7170686	15	48821350	A	G	0,0002	36,2761	0,0334	0,0055	1,7E-09	-0,0028	0,0231	0,9050	instrument
rs7191697	16	89678165	A	G	0,0002	46,8946	0,0369	0,0054	7,5E-12	-0,0094	0,0241	0,6961	instrument
rs7234167	18	46744610	C	G	0,0002	30,4480	0,0206	0,0037	3,4E-08	0,0036	0,0152	0,8097	instrument
rs7244464	18	20729714	A	C	0,0005	90,7247	0,0372	0,0039	1,7E-21	-0,0282	0,0163	0,0825	instrument
rs72708236	1	155980320	G	T	0,0003	54,7890	0,0703	0,0095	1,3E-13	-0,0183	0,0411	0,6558	instrument
rs72755233	15	100692953	A	G	0,0008	160,7807	0,0645	0,0051	7,6E-37	-0,0402	0,0260	0,1224	instrument
rs72776438	5	77438797	T	G	0,0002	36,1798	0,0228	0,0038	1,8E-09	0,0180	0,0164	0,2735	instrument
rs73004967	19	19717056	G	A	0,0002	40,6530	0,0405	0,0064	1,8E-10	0,0380	0,0268	0,1564	instrument
rs73175572	3	185490184	G	A	0,0002	36,0236	-0,0314	0,0052	2,0E-09	-0,0132	0,0219	0,5459	instrument

rs732563	8	23345526	T	C	0,0002	41,4088	0,0208	0,0032	1,2E-10	-0,0083	0,0132	0,5302	instrument
rs73407643	6	35169123	C	T	0,0002	36,6869	0,0751	0,0124	1,4E-09	0,0091	0,0513	0,8585	instrument
rs741207	4	18321092	T	C	0,0002	35,8677	-0,0200	0,0033	2,1E-09	-0,0158	0,0135	0,2397	instrument
rs7502307	17	65989961	G	C	0,0003	54,1543	-0,0300	0,0041	1,9E-13	-0,0207	0,0165	0,2118	instrument
rs75223147	6	19831020	G	T	0,0003	56,7154	-0,0848	0,0113	5,1E-14	0,0492	0,0659	0,4558	instrument
rs75621580	4	12916514	A	G	0,0002	30,7689	0,0425	0,0077	2,9E-08	0,0067	0,0337	0,8418	instrument
rs7626449	3	70921329	A	G	0,0002	39,4254	-0,0225	0,0036	3,4E-10	-0,0105	0,0145	0,4713	instrument
rs7641375	3	57568444	A	C	0,0002	43,0725	-0,0219	0,0033	5,3E-11	-0,0112	0,0138	0,4153	instrument
rs7689420	4	145568352	T	C	0,0008	162,5608	0,0548	0,0043	3,4E-37	-0,0052	0,0179	0,7733	instrument
rs7697556	4	73515313	T	C	0,0005	96,6869	-0,0318	0,0032	8,2E-23	-0,0010	0,0136	0,9430	instrument
rs7718768	5	178536089	A	G	0,0003	62,8525	-0,0271	0,0034	2,2E-15	-0,0227	0,0142	0,1103	instrument
rs7733331	5	32828846	T	C	0,0002	34,2221	-0,0193	0,0033	4,9E-09	-0,0046	0,0138	0,7401	instrument
rs7741741	6	142655801	A	T	0,0005	102,6158	0,0362	0,0036	4,2E-24	0,0175	0,0146	0,2317	instrument
rs7752292	6	131317876	A	T	0,0002	42,4187	-0,0218	0,0033	7,4E-11	0,0054	0,0137	0,6920	instrument
rs7804293	7	92287849	G	T	0,0004	73,9248	-0,0315	0,0037	8,1E-18	0,0208	0,0148	0,1598	instrument
rs78198962	2	233094868	T	C	0,0002	29,7349	-0,0499	0,0092	5,0E-08	-0,0638	0,0465	0,1695	instrument
rs78821460	5	127874149	C	G	0,0002	32,3189	0,0330	0,0058	1,3E-08	-0,0015	0,0238	0,9505	instrument
rs79312203	20	32082962	A	G	0,0002	36,7596	-0,0326	0,0054	1,3E-09	0,0084	0,0222	0,7061	instrument
rs7952436	11	67024534	T	C	0,0006	119,9012	0,0637	0,0058	6,7E-28	-0,0313	0,0292	0,2849	instrument
rs7994151	13	115038879	G	A	0,0002	41,0364	0,0289	0,0045	1,5E-10	-0,0226	0,0186	0,2244	instrument
rs8003703	14	33411639	A	T	0,0002	31,7078	0,0642	0,0114	1,8E-08	0,0605	0,0511	0,2365	instrument
rs80085026	7	148636421	G	C	0,0003	55,0558	-0,0308	0,0042	1,2E-13	0,0019	0,0173	0,9106	instrument
rs8103728	19	33900257	C	G	0,0004	82,6818	0,0313	0,0034	9,7E-20	0,0054	0,0141	0,7025	instrument
rs832540	5	56199202	G	A	0,0002	32,1826	0,0193	0,0034	1,4E-08	-0,0269	0,0138	0,0521	instrument
rs849141	7	28185091	A	G	0,0003	66,0149	-0,0289	0,0036	4,5E-16	-0,0207	0,0147	0,1591	instrument
rs862041	14	74986403	T	C	0,0003	50,5374	0,0240	0,0034	1,2E-12	0,0107	0,0139	0,4408	instrument
rs9346545	6	168806788	C	A	0,0002	29,9315	0,0197	0,0036	4,5E-08	-0,0139	0,0147	0,3437	instrument
rs9358912	6	26211146	T	G	0,0012	232,2552	0,0554	0,0036	2,0E-52	-0,0029	0,0144	0,8407	instrument
rs9391253	6	105367616	T	A	0,0005	93,9339	-0,0335	0,0035	3,3E-22	0,0150	0,0142	0,2884	instrument
rs9398433	6	116753939	G	T	0,0002	43,0200	-0,0219	0,0033	5,4E-11	0,0181	0,0135	0,1805	instrument
rs9442571	1	9349611	A	T	0,0002	41,2288	-0,0313	0,0049	1,4E-10	-0,0174	0,0210	0,4068	instrument

rs9496567	6	100602753	A	G	0,0002	30,3267	-0,0208	0,0038	3,7E-08	0,0102	0,0166	0,5392	instrument
rs9523325	13	92051677	T	G	0,0002	39,1496	-0,0258	0,0041	3,9E-10	0,0091	0,0164	0,5779	instrument
rs9614671	22	45839056	A	G	0,0002	29,7676	-0,0221	0,0040	4,9E-08	0,0127	0,0167	0,4472	instrument
rs979131	20	6566965	C	T	0,0002	33,8953	-0,0199	0,0034	5,8E-09	-0,0165	0,0139	0,2339	instrument
rs9809116	3	72397279	G	A	0,0002	35,6763	0,0199	0,0033	2,3E-09	0,0081	0,0138	0,5572	instrument
rs994014	4	82165790	C	T	0,0005	90,3251	-0,0333	0,0035	2,0E-21	-0,0182	0,0143	0,2042	instrument
rs9943184	1	170784430	T	G	0,0002	36,8809	-0,0294	0,0048	1,3E-09	-0,0017	0,0198	0,9310	instrument
rs9965184	18	40776606	T	A	0,0002	33,1657	0,0196	0,0034	8,5E-09	0,0016	0,0140	0,9077	instrument

Table S30. Single Nucleotide Polymorphisms (SNPs) used as instruments in the Mendelian randomization analyses for the effect of LFR on ER- breast cancer based on the genome-wide significance threshold P = 5E-08. R² represents the explained variance in the exposure. SNP-specific instrument strength is given by the respective F-statistic. Some of the genetic variants were identified as outliers in the iterative approach and therefore excluded from the analyses.

SNP	CHR	POS	EA	OA	R2	Fstat	β_{LFR}	SE_{LFR}	P_{LFR}	β_{ER-}	SE_{ER-}	P_{ER-}	$variant_{ER-}$
rs1002436	1	89146852	A	G	0,0002	33,9070	0,0189	0,0032	5,8E-09	-0,0110	0,0115	0,3361	instrument
rs1007358	7	46201355	G	A	0,0002	31,5391	-0,0221	0,0039	2,0E-08	0,0100	0,0143	0,4826	instrument
rs10172196	2	36780549	A	G	0,0003	53,8017	-0,0259	0,0035	2,2E-13	0,0200	0,0123	0,1046	instrument
rs1020048	9	86666993	A	C	0,0002	40,1825	-0,0265	0,0042	2,3E-10	0,0072	0,0144	0,6152	instrument
rs10445034	16	88840564	T	C	0,0002	38,3776	0,0239	0,0039	5,8E-10	0,0031	0,0136	0,8201	instrument
rs10894192	11	130266117	T	A	0,0002	44,5418	0,0221	0,0033	2,5E-11	0,0108	0,0126	0,3918	instrument
rs10901171	9	133427194	C	T	0,0002	43,2039	0,0220	0,0033	4,9E-11	0,0032	0,0123	0,7979	instrument
rs11205303	1	149906413	C	T	0,0018	340,7680	-0,0605	0,0033	4,8E-76	0,0404	0,0119	0,0007	outlier
rs112540634	6	34623905	T	C	0,0006	106,9145	-0,0487	0,0047	4,7E-25	-0,0157	0,0163	0,3335	instrument
rs11259936	15	84580582	A	C	0,0012	235,0064	0,0495	0,0032	5,0E-53	-0,0041	0,0113	0,7128	instrument
rs112635299	14	94838142	T	G	0,0004	70,8115	-0,0942	0,0112	3,9E-17	-0,0183	0,0454	0,6876	instrument
rs113059026	10	32158478	A	C	0,0002	42,1586	0,0233	0,0036	8,4E-11	-0,0224	0,0125	0,0739	instrument
rs11466399	1	218591623	A	G	0,0003	63,9993	-0,0287	0,0036	1,3E-15	0,0033	0,0124	0,7914	instrument
rs11666808	19	18383506	T	C	0,0002	46,9631	-0,0230	0,0033	7,2E-12	0,0028	0,0119	0,8165	instrument
rs11695471	2	25457708	A	T	0,0005	88,6790	0,0324	0,0034	4,7E-21	-0,0325	0,0128	0,0111	instrument
rs12103006	16	24726237	A	G	0,0002	31,2925	0,0183	0,0033	2,2E-08	-0,0065	0,0115	0,5731	instrument
rs12238852	9	98382084	T	C	0,0002	40,8700	-0,0296	0,0046	1,6E-10	0,0152	0,0165	0,3566	instrument
rs12756815	1	113207243	A	C	0,0003	62,4410	0,0316	0,0040	2,7E-15	0,0064	0,0141	0,6509	instrument

rs13128180	4	56286188	T	G	0,0003	57,4558	-0,0267	0,0035	3,5E-14	0,0188	0,0121	0,1189	instrument
rs1313237	14	60848224	C	A	0,0003	50,8506	-0,0236	0,0033	1,0E-12	0,0105	0,0117	0,3654	instrument
rs132941	22	38545942	C	T	0,0003	64,2716	-0,0261	0,0033	1,1E-15	-0,0084	0,0113	0,4570	instrument
rs13306455	19	7184721	T	C	0,0002	32,1259	-0,0244	0,0043	1,4E-08	-0,0139	0,0152	0,3609	instrument
rs138809270	2	219929394	T	C	0,0002	39,7778	0,0828	0,0131	2,8E-10	-0,0528	0,0467	0,2584	instrument
rs143384	20	34025756	G	A	0,0009	179,8262	-0,0441	0,0033	5,4E-41	-0,0221	0,0116	0,0558	instrument
rs143840904	11	2813322	T	C	0,0002	44,7289	0,0842	0,0126	2,3E-11	-0,0093	0,0587	0,8738	instrument
rs1452815	8	78123634	G	A	0,0002	30,3818	-0,0197	0,0036	3,6E-08	0,0074	0,0125	0,5561	instrument
rs1513304	3	99269750	C	T	0,0002	40,5765	-0,0226	0,0035	1,9E-10	0,0281	0,0133	0,0345	instrument
rs1550436	15	74221157	T	C	0,0004	77,3160	0,0286	0,0032	1,5E-18	0,0085	0,0113	0,4513	instrument
rs16942324	15	89383854	A	C	0,0004	70,6937	0,0844	0,0100	4,2E-17	-0,0129	0,0425	0,7621	instrument
rs16948048	17	47440466	G	A	0,0002	42,5621	0,0218	0,0033	6,9E-11	0,0038	0,0117	0,7427	instrument
rs16953461	17	46261736	G	T	0,0003	54,8186	0,0292	0,0039	1,3E-13	-0,0071	0,0139	0,6075	instrument
rs16972537	16	73802389	A	G	0,0002	33,0736	-0,0194	0,0034	8,9E-09	0,0018	0,0121	0,8835	instrument
rs17698176	17	44819595	G	T	0,0003	62,1883	-0,0307	0,0039	3,1E-15	0,0001	0,0148	0,9922	instrument
rs17744040	9	16842137	C	T	0,0002	34,5740	0,0309	0,0053	4,1E-09	-0,0079	0,0172	0,6475	instrument
rs1801282	3	12393125	G	C	0,0003	51,5519	-0,0356	0,0050	7,0E-13	0,0187	0,0170	0,2723	instrument
rs1980030	10	120970027	G	A	0,0002	32,0806	-0,0186	0,0033	1,5E-08	0,0011	0,0120	0,9257	instrument
rs2007482	6	2169724	T	C	0,0002	36,6626	0,0199	0,0033	1,4E-09	-0,0125	0,0121	0,3024	instrument
rs2058908	16	53806145	T	C	0,0002	44,3951	0,0243	0,0037	2,7E-11	0,0544	0,0131	3,4E-05	outlier
rs2126643	1	103404384	T	C	0,0002	40,1318	-0,0218	0,0034	2,4E-10	0,0046	0,0119	0,6968	instrument
rs2158694	7	2863443	G	T	0,0008	161,7967	0,0450	0,0035	4,5E-37	-0,0028	0,0123	0,8209	instrument
rs2173295	1	54943264	C	A	0,0002	33,0737	0,0276	0,0048	8,9E-09	-0,0184	0,0182	0,3142	instrument
rs2274432	1	184020945	A	G	0,0005	86,6007	-0,0315	0,0034	1,3E-20	0,0276	0,0119	0,0199	instrument
rs2293576	11	47434986	A	G	0,0003	48,9295	0,0240	0,0034	2,7E-12	-0,0109	0,0119	0,3619	instrument
rs2370983	14	79903376	G	A	0,0002	33,4890	0,0196	0,0034	7,2E-09	-0,0080	0,0126	0,5264	instrument
rs2516633	11	62371773	A	C	0,0002	31,4718	0,0197	0,0035	2,0E-08	0,0275	0,0130	0,0346	instrument
rs256904	5	55810305	A	T	0,0003	66,6012	-0,0302	0,0037	3,3E-16	-0,0132	0,0135	0,3284	instrument
rs2641699	16	84886306	C	G	0,0002	35,6047	0,0200	0,0034	2,4E-09	0,0056	0,0125	0,6526	instrument
rs264762	5	64384386	A	C	0,0003	52,4171	0,0248	0,0034	4,5E-13	0,0165	0,0120	0,1682	instrument
rs2712169	2	217671349	G	A	0,0003	49,6879	-0,0233	0,0033	1,8E-12	-0,0070	0,0116	0,5446	instrument

rs2715094	7	50730452	G	A	0,0003	57,7290	-0,0282	0,0037	3,0E-14	-0,0043	0,0138	0,7544	instrument
rs2768641	9	96948863	G	A	0,0002	34,4213	-0,0455	0,0078	4,4E-09	-0,0613	0,0298	0,0396	instrument
rs2811462	3	129303436	G	T	0,0003	52,3447	-0,0281	0,0039	4,7E-13	0,0206	0,0139	0,1381	instrument
rs2820446	1	219748818	G	C	0,0007	132,7090	-0,0405	0,0035	1,1E-30	0,0071	0,0127	0,5729	instrument
rs2844795	6	30073847	C	T	0,0002	41,4345	-0,0208	0,0032	1,2E-10	0,0010	0,0116	0,9313	instrument
rs28539246	17	29226630	A	T	0,0003	48,5804	0,0231	0,0033	3,2E-12	-0,0330	0,0116	0,0046	outlier
rs28716235	8	135602976	C	A	0,0002	33,4427	0,0194	0,0034	7,4E-09	-0,0011	0,0118	0,9241	instrument
rs2886564	1	42496924	G	A	0,0002	31,4381	-0,0200	0,0036	2,1E-08	-0,0054	0,0123	0,6609	instrument
rs3020421	6	152348122	A	G	0,0002	35,0224	-0,0206	0,0035	3,3E-09	-0,0165	0,0120	0,1691	instrument
rs3218036	19	30305684	A	G	0,0002	29,8550	-0,0188	0,0034	4,7E-08	0,0599	0,0119	5,2E-07	outlier
rs34517439	1	78450517	A	C	0,0003	65,2049	-0,0403	0,0050	6,8E-16	0,0054	0,0196	0,7819	instrument
rs34580448	5	82810884	C	T	0,0004	79,5477	0,0727	0,0081	4,8E-19	-0,0233	0,0295	0,4296	instrument
rs35307904	9	78511889	A	G	0,0006	116,4229	0,0537	0,0050	3,8E-27	0,0021	0,0190	0,9117	instrument
rs35874463	15	67457698	G	A	0,0003	66,0962	-0,0563	0,0069	4,3E-16	0,0120	0,0265	0,6505	instrument
rs3738814	1	17331676	G	A	0,0003	60,4500	0,0253	0,0033	7,6E-15	-0,0082	0,0113	0,4676	instrument
rs3791679	2	56096892	G	A	0,0009	179,2902	0,0516	0,0039	7,3E-41	0,0094	0,0131	0,4732	instrument
rs3794782	17	18127914	C	G	0,0002	33,0277	-0,0191	0,0033	9,1E-09	-0,0161	0,0116	0,1657	instrument
rs3823974	7	20442796	C	T	0,0003	60,9486	0,0258	0,0033	5,9E-15	0,0101	0,0114	0,3762	instrument
rs3850639	1	172253634	C	T	0,0003	56,8058	-0,0297	0,0039	4,8E-14	0,0042	0,0137	0,7569	instrument
rs4073717	5	170864021	T	G	0,0002	33,2233	0,0233	0,0040	8,2E-09	0,0139	0,0145	0,3376	instrument
rs41284816	13	50655989	T	G	0,0002	46,3484	-0,0835	0,0123	9,9E-12	0,0410	0,0376	0,2756	instrument
rs41313250	1	62483623	G	A	0,0002	36,9903	0,0319	0,0052	1,2E-09	-0,0038	0,0177	0,8318	instrument
rs4236324	7	27302727	C	T	0,0002	37,5030	-0,0274	0,0045	9,1E-10	-0,0006	0,0161	0,9723	instrument
rs4246308	15	100765202	A	G	0,0003	59,4589	0,0250	0,0032	1,3E-14	-0,0021	0,0113	0,8548	instrument
rs4321401	2	69348216	T	C	0,0003	48,9715	0,0227	0,0032	2,6E-12	-0,0114	0,0123	0,3548	instrument
rs4421120	5	32763118	A	G	0,0003	50,1542	-0,0311	0,0044	1,4E-12	0,0109	0,0163	0,5022	instrument
rs4432932	5	115039414	G	A	0,0002	31,1249	-0,0204	0,0037	2,4E-08	0,0147	0,0125	0,2409	instrument
rs4518624	8	130715510	A	G	0,0002	38,0685	0,0203	0,0033	6,8E-10	-0,0062	0,0115	0,5910	instrument
rs454345	5	88729851	G	A	0,0002	33,8255	-0,0194	0,0033	6,0E-09	0,0208	0,0118	0,0780	instrument
rs4803461	19	41908871	G	A	0,0002	33,2003	-0,0190	0,0033	8,3E-09	-0,0054	0,0122	0,6546	instrument
rs4842133	9	139127566	A	C	0,0004	69,4715	-0,0300	0,0036	7,8E-17	0,0264	0,0133	0,0479	instrument

rs4901541	14	55235282	C	T	0,0002	40,4873	0,0211	0,0033	2,0E-10	0,0018	0,0126	0,8882	instrument
rs4936122	11	130734264	C	A	0,0002	30,3818	-0,0181	0,0033	3,6E-08	-0,0079	0,0118	0,5012	instrument
rs496298	10	131355130	A	G	0,0003	65,4151	0,0276	0,0034	6,1E-16	-0,0137	0,0121	0,2569	instrument
rs4973032	2	232990462	T	G	0,0002	40,7423	0,0769	0,0121	1,7E-10	0,0461	0,0403	0,2530	instrument
rs4974072	3	41222586	A	G	0,0002	43,3091	-0,0214	0,0033	4,7E-11	0,0081	0,0114	0,4779	instrument
rs538656	18	57850422	T	G	0,0002	31,4269	-0,0213	0,0038	2,1E-08	-0,0244	0,0133	0,0667	instrument
rs544148	6	117466494	A	G	0,0002	30,7134	0,0182	0,0033	3,0E-08	0,0228	0,0115	0,0476	instrument
rs55633655	5	131598756	T	G	0,0003	53,1144	0,0237	0,0033	3,2E-13	-0,0032	0,0113	0,7797	instrument
rs56021161	14	64817100	A	T	0,0002	30,5916	0,0362	0,0065	3,2E-08	-0,0048	0,0244	0,8448	instrument
rs56186137	16	28825953	G	A	0,0006	108,1589	-0,0341	0,0033	2,6E-25	-0,0054	0,0116	0,6412	instrument
rs56277550	21	18092263	A	G	0,0002	33,5006	0,0187	0,0032	7,1E-09	-0,0169	0,0117	0,1494	instrument
rs57674902	20	4096741	T	C	0,0002	38,1302	-0,0210	0,0034	6,6E-10	0,0065	0,0128	0,6119	instrument
rs584961	11	75277628	A	G	0,0002	35,1408	-0,0302	0,0051	3,1E-09	0,0122	0,0198	0,5372	instrument
rs61734651	20	61451332	T	C	0,0002	37,8836	-0,0421	0,0068	7,5E-10	-0,0114	0,0273	0,6767	instrument
rs61834590	1	227421117	G	T	0,0002	43,2696	0,0298	0,0045	4,8E-11	0,0006	0,0159	0,9675	instrument
rs62106258	2	417167	C	T	0,0002	32,2053	0,0424	0,0075	1,4E-08	0,0126	0,0326	0,6995	instrument
rs62271373	3	150066540	A	T	0,0002	41,1775	0,0455	0,0071	1,4E-10	-0,0003	0,0293	0,9923	instrument
rs62515452	8	57187356	T	C	0,0002	34,2338	-0,0267	0,0046	4,9E-09	-0,0048	0,0181	0,7913	instrument
rs62621197	19	8670147	T	C	0,0008	150,7968	0,1140	0,0093	1,1E-34	0,0331	0,0403	0,4108	instrument
rs640245	11	30393360	T	C	0,0002	37,0512	-0,0198	0,0033	1,1E-09	-0,0047	0,0115	0,6798	instrument
rs6486059	11	12824581	A	G	0,0003	52,4315	-0,0238	0,0033	4,5E-13	-0,0062	0,0118	0,5977	instrument
rs6503410	17	43094140	T	C	0,0002	35,0934	0,0215	0,0036	3,2E-09	-0,0090	0,0127	0,4747	instrument
rs6585827	10	124165615	A	G	0,0002	45,0505	-0,0217	0,0032	1,9E-11	0,0091	0,0116	0,4340	instrument
rs6717858	2	165539661	C	T	0,0003	64,7696	-0,0265	0,0033	8,5E-16	-0,0217	0,0115	0,0582	instrument
rs6739701	2	105132332	G	A	0,0002	44,8493	-0,0220	0,0033	2,1E-11	-0,0085	0,0127	0,5048	instrument
rs6785012	3	141109348	T	C	0,0012	231,9505	-0,0496	0,0033	2,3E-52	0,0150	0,0114	0,1861	instrument
rs68049170	10	72432047	A	G	0,0003	56,1594	-0,0273	0,0036	6,7E-14	0,0112	0,0128	0,3792	instrument
rs6843340	4	48619937	T	C	0,0002	30,9912	-0,0180	0,0032	2,6E-08	0,0059	0,0113	0,6012	instrument
rs6887247	5	157881647	C	T	0,0003	53,8897	-0,0257	0,0035	2,1E-13	0,0120	0,0126	0,3419	instrument
rs702605	5	53167028	T	C	0,0002	34,1286	-0,0189	0,0032	5,2E-09	-0,0177	0,0112	0,1150	instrument
rs71531541	6	7066481	C	T	0,0002	30,1727	0,0200	0,0036	4,0E-08	-0,0095	0,0130	0,4649	instrument

rs7155279	14	92485881	T	G	0,0003	47,9967	0,0233	0,0034	4,3E-12	-0,0083	0,0117	0,4812	instrument
rs7170686	15	48821350	A	G	0,0002	36,2761	0,0334	0,0055	1,7E-09	0,0420	0,0207	0,0423	instrument
rs7191697	16	89678165	A	G	0,0002	46,8946	0,0369	0,0054	7,5E-12	-0,0290	0,0217	0,1828	instrument
rs7234167	18	46744610	C	G	0,0002	30,4480	0,0206	0,0037	3,4E-08	0,0076	0,0129	0,5541	instrument
rs7244464	18	20729714	A	C	0,0005	90,7247	0,0372	0,0039	1,7E-21	0,0130	0,0139	0,3488	instrument
rs72708236	1	155980320	G	T	0,0003	54,7890	0,0703	0,0095	1,3E-13	0,0054	0,0369	0,8842	instrument
rs72755233	15	100692953	A	G	0,0008	160,7807	0,0645	0,0051	7,6E-37	-0,0090	0,0234	0,7019	instrument
rs72776438	5	77438797	T	G	0,0002	36,1798	0,0228	0,0038	1,8E-09	0,0060	0,0150	0,6876	instrument
rs73004967	19	19717056	G	A	0,0002	40,6530	0,0405	0,0064	1,8E-10	0,0162	0,0228	0,4785	instrument
rs73175572	3	185490184	G	A	0,0002	36,0236	-0,0314	0,0052	2,0E-09	-0,0067	0,0197	0,7330	instrument
rs732563	8	23345526	T	C	0,0002	41,4088	0,0208	0,0032	1,2E-10	-0,0025	0,0112	0,8213	instrument
rs73407643	6	35169123	C	T	0,0002	36,6869	0,0751	0,0124	1,4E-09	0,0573	0,0446	0,1983	instrument
rs741207	4	18321092	T	C	0,0002	35,8677	-0,0200	0,0033	2,1E-09	-0,0037	0,0115	0,7487	instrument
rs7502307	17	65989961	G	C	0,0003	54,1543	-0,0300	0,0041	1,9E-13	0,0225	0,0145	0,1203	instrument
rs75223147	6	19831020	G	T	0,0003	56,7154	-0,0848	0,0113	5,1E-14	-0,1286	0,0674	0,0563	instrument
rs75621580	4	12916514	A	G	0,0002	30,7689	0,0425	0,0077	2,9E-08	0,0003	0,0301	0,9923	instrument
rs7626449	3	70921329	A	G	0,0002	39,4254	-0,0225	0,0036	3,4E-10	0,0147	0,0125	0,2387	instrument
rs7641375	3	57568444	A	C	0,0002	43,0725	-0,0219	0,0033	5,3E-11	-0,0198	0,0118	0,0933	instrument
rs7689420	4	145568352	T	C	0,0008	162,5608	0,0548	0,0043	3,4E-37	0,0147	0,0152	0,3338	instrument
rs7697556	4	73515313	T	C	0,0005	96,6869	-0,0318	0,0032	8,2E-23	0,0175	0,0118	0,1374	instrument
rs7718768	5	178536089	A	G	0,0003	62,8525	-0,0271	0,0034	2,2E-15	0,0146	0,0123	0,2344	instrument
rs7733331	5	32828846	T	C	0,0002	34,2221	-0,0193	0,0033	4,9E-09	0,0044	0,0121	0,7157	instrument
rs7741741	6	142655801	A	T	0,0005	102,6158	0,0362	0,0036	4,2E-24	0,0084	0,0125	0,5011	instrument
rs7752292	6	131317876	A	T	0,0002	42,4187	-0,0218	0,0033	7,4E-11	-0,0036	0,0117	0,7601	instrument
rs7804293	7	92287849	G	T	0,0004	73,9248	-0,0315	0,0037	8,1E-18	0,0229	0,0125	0,0665	instrument
rs78198962	2	233094868	T	C	0,0002	29,7349	-0,0499	0,0092	5,0E-08	-0,0185	0,0437	0,6720	instrument
rs78821460	5	127874149	C	G	0,0002	32,3189	0,0330	0,0058	1,3E-08	0,0428	0,0204	0,0362	instrument
rs79312203	20	32082962	A	G	0,0002	36,7596	-0,0326	0,0054	1,3E-09	-0,0212	0,0192	0,2692	instrument
rs7952436	11	67024534	T	C	0,0006	119,9012	0,0637	0,0058	6,7E-28	0,0077	0,0265	0,7712	instrument
rs7994151	13	115038879	G	A	0,0002	41,0364	0,0289	0,0045	1,5E-10	-0,0041	0,0158	0,7980	instrument
rs8003703	14	33411639	A	T	0,0002	31,7078	0,0642	0,0114	1,8E-08	-0,0140	0,0471	0,7658	instrument

rs80085026	7	148636421	G	C	0,0003	55,0558	-0,0308	0,0042	1,2E-13	0,0030	0,0152	0,8416	instrument
rs8103728	19	33900257	C	G	0,0004	82,6818	0,0313	0,0034	9,7E-20	0,0070	0,0122	0,5672	instrument
rs832540	5	56199202	G	A	0,0002	32,1826	0,0193	0,0034	1,4E-08	0,0488	0,0117	3,1E-05	outlier
rs849141	7	28185091	A	G	0,0003	66,0149	-0,0289	0,0036	4,5E-16	0,0056	0,0125	0,6544	instrument
rs862041	14	74986403	T	C	0,0003	50,5374	0,0240	0,0034	1,2E-12	0,0004	0,0120	0,9724	instrument
rs9346545	6	168806788	C	A	0,0002	29,9315	0,0197	0,0036	4,5E-08	-0,0081	0,0124	0,5148	instrument
rs9358912	6	26211146	T	G	0,0012	232,2552	0,0554	0,0036	2,0E-52	0,0153	0,0123	0,2127	instrument
rs9391253	6	105367616	T	A	0,0005	93,9339	-0,0335	0,0035	3,3E-22	-0,0094	0,0121	0,4397	instrument
rs9398433	6	116753939	G	T	0,0002	43,0200	-0,0219	0,0033	5,4E-11	-0,0111	0,0116	0,3369	instrument
rs9442571	1	9349611	A	T	0,0002	41,2288	-0,0313	0,0049	1,4E-10	0,0329	0,0184	0,0743	instrument
rs9496567	6	100602753	A	G	0,0002	30,3267	-0,0208	0,0038	3,7E-08	-0,0207	0,0150	0,1687	instrument
rs9523325	13	92051677	T	G	0,0002	39,1496	-0,0258	0,0041	3,9E-10	0,0179	0,0143	0,2122	instrument
rs9614671	22	45839056	A	G	0,0002	29,7676	-0,0221	0,0040	4,9E-08	0,0225	0,0143	0,1156	instrument
rs979131	20	6566965	C	T	0,0002	33,8953	-0,0199	0,0034	5,8E-09	0,0082	0,0119	0,4901	instrument
rs9809116	3	72397279	G	A	0,0002	35,6763	0,0199	0,0033	2,3E-09	-0,0162	0,0118	0,1703	instrument
rs994014	4	82165790	C	T	0,0005	90,3251	-0,0333	0,0035	2,0E-21	-0,0095	0,0124	0,4442	instrument
rs9943184	1	170784430	T	G	0,0002	36,8809	-0,0294	0,0048	1,3E-09	-0,0261	0,0170	0,1235	instrument
rs9965184	18	40776606	T	A	0,0002	33,1657	0,0196	0,0034	8,5E-09	-0,0027	0,0120	0,8185	instrument

Table S31. Single Nucleotide Polymorphisms (SNPs) used as instruments in the Mendelian randomization analyses for the effect of LFR on ER+ breast cancer based on the genome-wide significance threshold P = 5E-08. R^2 represents the explained variance in the exposure. SNP-specific instrument strength is given by the respective F-statistic. Some of the genetic variants were identified as outliers in the iterative approach and therefore excluded from the analyses.

SNP	CHR	POS	EA	OA	R2	Fstat	β_{LFR}	SE_{LFR}	P_{LFR}	β_{ER+}	SE_{ER+}	P_{ER+}	$variant_{ER+}$
rs1002436	1	89146852	A	G	0,0002	33,9070	0,0189	0,0032	5,8E-09	-0,0182	0,0075	0,0157	instrument
rs1007358	7	46201355	G	A	0,0002	31,5391	-0,0221	0,0039	2,0E-08	-0,0012	0,0095	0,8995	instrument
rs10172196	2	36780549	A	G	0,0003	53,8017	-0,0259	0,0035	2,2E-13	-0,0158	0,0081	0,0523	instrument
rs1020048	9	86666993	A	C	0,0002	40,1825	-0,0265	0,0042	2,3E-10	0,0058	0,0094	0,5354	instrument
rs10445034	16	88840564	T	C	0,0002	38,3776	0,0239	0,0039	5,8E-10	-0,0134	0,0089	0,1321	instrument
rs10894192	11	130266117	T	A	0,0002	44,5418	0,0221	0,0033	2,5E-11	-0,0093	0,0083	0,2655	instrument
rs10901171	9	133427194	C	T	0,0002	43,2039	0,0220	0,0033	4,9E-11	-0,0007	0,0082	0,9284	instrument
rs11205303	1	149906413	C	T	0,0018	340,7680	-0,0605	0,0033	4,8E-76	0,0572	0,0077	8,4E-14	outlier

rs112540634	6	34623905	T	C	0,0006	106,9145	-0,0487	0,0047	4,7E-25	-0,0078	0,0106	0,4617	instrument
rs11259936	15	84580582	A	C	0,0012	235,0064	0,0495	0,0032	5,0E-53	0,0084	0,0074	0,2536	instrument
rs112635299	14	94838142	T	G	0,0004	70,8115	-0,0942	0,0112	3,9E-17	-0,0745	0,0288	0,0097	outlier
rs113059026	10	32158478	A	C	0,0002	42,1586	0,0233	0,0036	8,4E-11	-0,0262	0,0082	0,0014	outlier
rs11466399	1	218591623	A	G	0,0003	63,9993	-0,0287	0,0036	1,3E-15	0,0081	0,0082	0,3224	instrument
rs11666808	19	18383506	T	C	0,0002	46,9631	-0,0230	0,0033	7,2E-12	0,0032	0,0078	0,6868	instrument
rs11695471	2	25457708	A	T	0,0005	88,6790	0,0324	0,0034	4,7E-21	-0,0135	0,0084	0,1071	instrument
rs12103006	16	24726237	A	G	0,0002	31,2925	0,0183	0,0033	2,2E-08	0,0030	0,0075	0,6893	instrument
rs12238852	9	98382084	T	C	0,0002	40,8700	-0,0296	0,0046	1,6E-10	0,0101	0,0108	0,3496	instrument
rs12756815	1	113207243	A	C	0,0003	62,4410	0,0316	0,0040	2,7E-15	-0,0021	0,0091	0,8170	instrument
rs13128180	4	56286188	T	G	0,0003	57,4558	-0,0267	0,0035	3,5E-14	-0,0048	0,0079	0,5484	instrument
rs1313237	14	60848224	C	A	0,0003	50,8506	-0,0236	0,0033	1,0E-12	-0,0007	0,0076	0,9245	instrument
rs132941	22	38545942	C	T	0,0003	64,2716	-0,0261	0,0033	1,1E-15	-0,0409	0,0074	3,5E-08	outlier
rs13306455	19	7184721	T	C	0,0002	32,1259	-0,0244	0,0043	1,4E-08	-0,0045	0,0099	0,6469	instrument
rs138809270	2	219929394	T	C	0,0002	39,7778	0,0828	0,0131	2,8E-10	-0,0138	0,0294	0,6383	instrument
rs143384	20	34025756	G	A	0,0009	179,8262	-0,0441	0,0033	5,4E-41	0,0019	0,0076	0,8018	instrument
rs143840904	11	2813322	T	C	0,0002	44,7289	0,0842	0,0126	2,3E-11	-0,0338	0,0385	0,3797	instrument
rs1452815	8	78123634	G	A	0,0002	30,3818	-0,0197	0,0036	3,6E-08	0,0230	0,0082	0,0049	outlier
rs1513304	3	99269750	C	T	0,0002	40,5765	-0,0226	0,0035	1,9E-10	0,0317	0,0087	0,0003	outlier
rs1550436	15	74221157	T	C	0,0004	77,3160	0,0286	0,0032	1,5E-18	0,0139	0,0074	0,0595	instrument
rs16942324	15	89383854	A	C	0,0004	70,6937	0,0844	0,0100	4,2E-17	0,0341	0,0272	0,2091	instrument
rs16948048	17	47440466	G	A	0,0002	42,5621	0,0218	0,0033	6,9E-11	0,0104	0,0076	0,1727	instrument
rs16953461	17	46261736	G	T	0,0003	54,8186	0,0292	0,0039	1,3E-13	0,0126	0,0091	0,1631	instrument
rs16972537	16	73802389	A	G	0,0002	33,0736	-0,0194	0,0034	8,9E-09	-0,0134	0,0080	0,0946	instrument
rs17698176	17	44819595	G	T	0,0003	62,1883	-0,0307	0,0039	3,1E-15	0,0229	0,0095	0,0163	instrument
rs17744040	9	16842137	C	T	0,0002	34,5740	0,0309	0,0053	4,1E-09	0,0156	0,0111	0,1606	instrument
rs1801282	3	12393125	G	C	0,0003	51,5519	-0,0356	0,0050	7,0E-13	-0,0246	0,0112	0,0282	instrument
rs1980030	10	120970027	G	A	0,0002	32,0806	-0,0186	0,0033	1,5E-08	0,0079	0,0079	0,3159	instrument
rs2007482	6	2169724	T	C	0,0002	36,6626	0,0199	0,0033	1,4E-09	-0,0128	0,0080	0,1085	instrument
rs2058908	16	53806145	T	C	0,0002	44,3951	0,0243	0,0037	2,7E-11	0,0249	0,0086	0,0039	outlier
rs2126643	1	103404384	T	C	0,0002	40,1318	-0,0218	0,0034	2,4E-10	0,0085	0,0078	0,2734	instrument

rs2158694	7	2863443	G	T	0,0008	161,7967	0,0450	0,0035	4,5E-37	0,0146	0,0080	0,0685	instrument
rs2173295	1	54943264	C	A	0,0002	33,0737	0,0276	0,0048	8,9E-09	0,0141	0,0119	0,2378	instrument
rs2274432	1	184020945	A	G	0,0005	86,6007	-0,0315	0,0034	1,3E-20	0,0077	0,0078	0,3222	instrument
rs2293576	11	47434986	A	G	0,0003	48,9295	0,0240	0,0034	2,7E-12	-0,0116	0,0078	0,1393	instrument
rs2370983	14	79903376	G	A	0,0002	33,4890	0,0196	0,0034	7,2E-09	-0,0163	0,0083	0,0495	instrument
rs2516633	11	62371773	A	C	0,0002	31,4718	0,0197	0,0035	2,0E-08	0,0012	0,0086	0,8894	instrument
rs256904	5	55810305	A	T	0,0003	66,6012	-0,0302	0,0037	3,3E-16	-0,0035	0,0089	0,6969	instrument
rs2641699	16	84886306	C	G	0,0002	35,6047	0,0200	0,0034	2,4E-09	0,0062	0,0083	0,4538	instrument
rs264762	5	64384386	A	C	0,0003	52,4171	0,0248	0,0034	4,5E-13	0,0250	0,0078	0,0014	outlier
rs2712169	2	217671349	G	A	0,0003	49,6879	-0,0233	0,0033	1,8E-12	0,0141	0,0076	0,0626	instrument
rs2715094	7	50730452	G	A	0,0003	57,7290	-0,0282	0,0037	3,0E-14	0,0014	0,0090	0,8785	instrument
rs2768641	9	96948863	G	A	0,0002	34,4213	-0,0455	0,0078	4,4E-09	-0,0533	0,0194	0,0059	outlier
rs2811462	3	129303436	G	T	0,0003	52,3447	-0,0281	0,0039	4,7E-13	0,0305	0,0092	0,0009	outlier
rs2820446	1	219748818	G	C	0,0007	132,7090	-0,0405	0,0035	1,1E-30	-0,0251	0,0083	0,0025	outlier
rs2844795	6	30073847	C	T	0,0002	41,4345	-0,0208	0,0032	1,2E-10	-0,0041	0,0076	0,5867	instrument
rs28539246	17	29226630	A	T	0,0003	48,5804	0,0231	0,0033	3,2E-12	-0,0325	0,0076	1,9E-05	outlier
rs28716235	8	135602976	C	A	0,0002	33,4427	0,0194	0,0034	7,4E-09	-0,0101	0,0077	0,1890	instrument
rs2886564	1	42496924	G	A	0,0002	31,4381	-0,0200	0,0036	2,1E-08	0,0005	0,0080	0,9535	instrument
rs3020421	6	152348122	A	G	0,0002	35,0224	-0,0206	0,0035	3,3E-09	0,0128	0,0078	0,1036	instrument
rs3218036	19	30305684	A	G	0,0002	29,8550	-0,0188	0,0034	4,7E-08	0,0191	0,0078	0,0145	instrument
rs34517439	1	78450517	A	C	0,0003	65,2049	-0,0403	0,0050	6,8E-16	0,0157	0,0126	0,2146	instrument
rs34580448	5	82810884	C	T	0,0004	79,5477	0,0727	0,0081	4,8E-19	-0,0137	0,0193	0,4788	instrument
rs35307904	9	78511889	A	G	0,0006	116,4229	0,0537	0,0050	3,8E-27	0,0108	0,0125	0,3857	instrument
rs35874463	15	67457698	G	A	0,0003	66,0962	-0,0563	0,0069	4,3E-16	0,0819	0,0166	8,6E-07	outlier
rs3738814	1	17331676	G	A	0,0003	60,4500	0,0253	0,0033	7,6E-15	-0,0130	0,0074	0,0803	instrument
rs3791679	2	56096892	G	A	0,0009	179,2902	0,0516	0,0039	7,3E-41	-0,0023	0,0087	0,7921	instrument
rs3794782	17	18127914	C	G	0,0002	33,0277	-0,0191	0,0033	9,1E-09	-0,0097	0,0076	0,2017	instrument
rs3823974	7	20442796	C	T	0,0003	60,9486	0,0258	0,0033	5,9E-15	-0,0080	0,0075	0,2872	instrument
rs3850639	1	172253634	C	T	0,0003	56,8058	-0,0297	0,0039	4,8E-14	-0,0170	0,0090	0,0590	instrument
rs4073717	5	170864021	T	G	0,0002	33,2233	0,0233	0,0040	8,2E-09	-0,0009	0,0095	0,9217	instrument
rs41284816	13	50655989	T	G	0,0002	46,3484	-0,0835	0,0123	9,9E-12	0,0397	0,0243	0,1029	instrument

rs41313250	1	62483623	G	A	0,0002	36,9903	0,0319	0,0052	1,2E-09	-0,0068	0,0115	0,5576	instrument
rs4236324	7	27302727	C	T	0,0002	37,5030	-0,0274	0,0045	9,1E-10	-0,0027	0,0106	0,8013	instrument
rs4246308	15	100765202	A	G	0,0003	59,4589	0,0250	0,0032	1,3E-14	0,0201	0,0074	0,0067	outlier
rs4321401	2	69348216	T	C	0,0003	48,9715	0,0227	0,0032	2,6E-12	-0,0012	0,0081	0,8871	instrument
rs4421120	5	32763118	A	G	0,0003	50,1542	-0,0311	0,0044	1,4E-12	-0,0062	0,0108	0,5671	instrument
rs4432932	5	115039414	G	A	0,0002	31,1249	-0,0204	0,0037	2,4E-08	-0,0062	0,0082	0,4531	instrument
rs4518624	8	130715510	A	G	0,0002	38,0685	0,0203	0,0033	6,8E-10	-0,0014	0,0076	0,8541	instrument
rs454345	5	88729851	G	A	0,0002	33,8255	-0,0194	0,0033	6,0E-09	0,0095	0,0077	0,2210	instrument
rs4803461	19	41908871	G	A	0,0002	33,2003	-0,0190	0,0033	8,3E-09	0,0112	0,0080	0,1615	instrument
rs4842133	9	139127566	A	C	0,0004	69,4715	-0,0300	0,0036	7,8E-17	0,0187	0,0086	0,0304	instrument
rs4901541	14	55235282	C	T	0,0002	40,4873	0,0211	0,0033	2,0E-10	-0,0007	0,0084	0,9357	instrument
rs4936122	11	130734264	C	A	0,0002	30,3818	-0,0181	0,0033	3,6E-08	-0,0025	0,0078	0,7517	instrument
rs496298	10	131355130	A	G	0,0003	65,4151	0,0276	0,0034	6,1E-16	0,0003	0,0079	0,9689	instrument
rs4973032	2	232990462	T	G	0,0002	40,7423	0,0769	0,0121	1,7E-10	0,0107	0,0263	0,6847	instrument
rs4974072	3	41222586	A	G	0,0002	43,3091	-0,0214	0,0033	4,7E-11	0,0092	0,0075	0,2188	instrument
rs538656	18	57850422	T	G	0,0002	31,4269	-0,0213	0,0038	2,1E-08	-0,0131	0,0087	0,1331	instrument
rs544148	6	117466494	A	G	0,0002	30,7134	0,0182	0,0033	3,0E-08	0,0046	0,0076	0,5433	instrument
rs55633655	5	131598756	T	G	0,0003	53,1144	0,0237	0,0033	3,2E-13	-0,0262	0,0074	0,0004	outlier
rs56021161	14	64817100	A	T	0,0002	30,5916	0,0362	0,0065	3,2E-08	0,0106	0,0159	0,5058	instrument
rs56186137	16	28825953	G	A	0,0006	108,1589	-0,0341	0,0033	2,6E-25	0,0091	0,0076	0,2308	instrument
rs56277550	21	18092263	A	G	0,0002	33,5006	0,0187	0,0032	7,1E-09	0,0003	0,0077	0,9670	instrument
rs57674902	20	4096741	T	C	0,0002	38,1302	-0,0210	0,0034	6,6E-10	0,0091	0,0085	0,2863	instrument
rs584961	11	75277628	A	G	0,0002	35,1408	-0,0302	0,0051	3,1E-09	0,0377	0,0129	0,0034	outlier
rs61734651	20	61451332	T	C	0,0002	37,8836	-0,0421	0,0068	7,5E-10	-0,0256	0,0175	0,1435	instrument
rs61834590	1	227421117	G	T	0,0002	43,2696	0,0298	0,0045	4,8E-11	-0,0116	0,0104	0,2654	instrument
rs62106258	2	417167	C	T	0,0002	32,2053	0,0424	0,0075	1,4E-08	0,0324	0,0214	0,1305	instrument
rs62271373	3	150066540	A	T	0,0002	41,1775	0,0455	0,0071	1,4E-10	-0,0130	0,0196	0,5088	instrument
rs62515452	8	57187356	T	C	0,0002	34,2338	-0,0267	0,0046	4,9E-09	0,0117	0,0117	0,3193	instrument
rs62621197	19	8670147	T	C	0,0008	150,7968	0,1140	0,0093	1,1E-34	-0,0424	0,0260	0,1033	instrument
rs640245	11	30393360	T	C	0,0002	37,0512	-0,0198	0,0033	1,1E-09	0,0031	0,0075	0,6771	instrument
rs6486059	11	12824581	A	G	0,0003	52,4315	-0,0238	0,0033	4,5E-13	0,0037	0,0077	0,6354	instrument

rs6503410	17	43094140	T	C	0,0002	35,0934	0,0215	0,0036	3,2E-09	0,0013	0,0082	0,8757	instrument
rs6585827	10	124165615	A	G	0,0002	45,0505	-0,0217	0,0032	1,9E-11	-0,0058	0,0076	0,4503	instrument
rs6717858	2	165539661	C	T	0,0003	64,7696	-0,0265	0,0033	8,5E-16	0,0008	0,0075	0,9137	instrument
rs6739701	2	105132332	G	A	0,0002	44,8493	-0,0220	0,0033	2,1E-11	0,0023	0,0085	0,7874	instrument
rs6785012	3	141109348	T	C	0,0012	231,9505	-0,0496	0,0033	2,3E-52	0,0546	0,0074	2,1E-13	outlier
rs68049170	10	72432047	A	G	0,0003	56,1594	-0,0273	0,0036	6,7E-14	0,0079	0,0083	0,3413	instrument
rs6843340	4	48619937	T	C	0,0002	30,9912	-0,0180	0,0032	2,6E-08	0,0221	0,0074	0,0027	outlier
rs6887247	5	157881647	C	T	0,0003	53,8897	-0,0257	0,0035	2,1E-13	0,0174	0,0082	0,0346	instrument
rs702605	5	53167028	T	C	0,0002	34,1286	-0,0189	0,0032	5,2E-09	-0,0187	0,0074	0,0112	outlier
rs71531541	6	7066481	C	T	0,0002	30,1727	0,0200	0,0036	4,0E-08	-0,0172	0,0085	0,0435	instrument
rs7155279	14	92485881	T	G	0,0003	47,9967	0,0233	0,0034	4,3E-12	0,0063	0,0077	0,4109	instrument
rs7170686	15	48821350	A	G	0,0002	36,2761	0,0334	0,0055	1,7E-09	0,0241	0,0140	0,0849	instrument
rs7191697	16	89678165	A	G	0,0002	46,8946	0,0369	0,0054	7,5E-12	-0,0019	0,0138	0,8901	instrument
rs7234167	18	46744610	C	G	0,0002	30,4480	0,0206	0,0037	3,4E-08	-0,0105	0,0085	0,2169	instrument
rs7244464	18	20729714	A	C	0,0005	90,7247	0,0372	0,0039	1,7E-21	0,0150	0,0091	0,0991	instrument
rs72708236	1	155980320	G	T	0,0003	54,7890	0,0703	0,0095	1,3E-13	-0,0336	0,0242	0,1660	instrument
rs72755233	15	100692953	A	G	0,0008	160,7807	0,0645	0,0051	7,6E-37	0,0136	0,0152	0,3710	instrument
rs72776438	5	77438797	T	G	0,0002	36,1798	0,0228	0,0038	1,8E-09	0,0031	0,0100	0,7525	instrument
rs73004967	19	19717056	G	A	0,0002	40,6530	0,0405	0,0064	1,8E-10	0,0138	0,0148	0,3523	instrument
rs73175572	3	185490184	G	A	0,0002	36,0236	-0,0314	0,0052	2,0E-09	0,0130	0,0129	0,3142	instrument
rs732563	8	23345526	T	C	0,0002	41,4088	0,0208	0,0032	1,2E-10	-0,0053	0,0074	0,4690	instrument
rs73407643	6	35169123	C	T	0,0002	36,6869	0,0751	0,0124	1,4E-09	-0,0324	0,0293	0,2682	instrument
rs741207	4	18321092	T	C	0,0002	35,8677	-0,0200	0,0033	2,1E-09	0,0096	0,0076	0,2023	instrument
rs7502307	17	65989961	G	C	0,0003	54,1543	-0,0300	0,0041	1,9E-13	0,0022	0,0094	0,8151	instrument
rs75223147	6	19831020	G	T	0,0003	56,7154	-0,0848	0,0113	5,1E-14	-0,0247	0,0417	0,5539	instrument
rs75621580	4	12916514	A	G	0,0002	30,7689	0,0425	0,0077	2,9E-08	0,0136	0,0196	0,4879	instrument
rs7626449	3	70921329	A	G	0,0002	39,4254	-0,0225	0,0036	3,4E-10	0,0169	0,0082	0,0396	instrument
rs7641375	3	57568444	A	C	0,0002	43,0725	-0,0219	0,0033	5,3E-11	-0,0010	0,0077	0,8946	instrument
rs7689420	4	145568352	T	C	0,0008	162,5608	0,0548	0,0043	3,4E-37	0,0010	0,0099	0,9228	instrument
rs7697556	4	73515313	T	C	0,0005	96,6869	-0,0318	0,0032	8,2E-23	0,0098	0,0078	0,2055	instrument
rs7718768	5	178536089	A	G	0,0003	62,8525	-0,0271	0,0034	2,2E-15	0,0046	0,0081	0,5657	instrument

rs7733331	5	32828846	T	C	0,0002	34,2221	-0,0193	0,0033	4,9E-09	-0,0040	0,0080	0,6199	instrument
rs7741741	6	142655801	A	T	0,0005	102,6158	0,0362	0,0036	4,2E-24	-0,0022	0,0082	0,7905	instrument
rs7752292	6	131317876	A	T	0,0002	42,4187	-0,0218	0,0033	7,4E-11	-0,0068	0,0077	0,3722	instrument
rs7804293	7	92287849	G	T	0,0004	73,9248	-0,0315	0,0037	8,1E-18	0,0199	0,0082	0,0153	instrument
rs78198962	2	233094868	T	C	0,0002	29,7349	-0,0499	0,0092	5,0E-08	0,0332	0,0285	0,2454	instrument
rs78821460	5	127874149	C	G	0,0002	32,3189	0,0330	0,0058	1,3E-08	-0,0037	0,0135	0,7853	instrument
rs79312203	20	32082962	A	G	0,0002	36,7596	-0,0326	0,0054	1,3E-09	-0,0228	0,0125	0,0684	instrument
rs7952436	11	67024534	T	C	0,0006	119,9012	0,0637	0,0058	6,7E-28	-0,0073	0,0171	0,6692	instrument
rs7994151	13	115038879	G	A	0,0002	41,0364	0,0289	0,0045	1,5E-10	0,0098	0,0104	0,3443	instrument
rs8003703	14	33411639	A	T	0,0002	31,7078	0,0642	0,0114	1,8E-08	0,0022	0,0303	0,9424	instrument
rs80085026	7	148636421	G	C	0,0003	55,0558	-0,0308	0,0042	1,2E-13	0,0311	0,0100	0,0018	outlier
rs8103728	19	33900257	C	G	0,0004	82,6818	0,0313	0,0034	9,7E-20	0,0264	0,0080	0,0010	outlier
rs832540	5	56199202	G	A	0,0002	32,1826	0,0193	0,0034	1,4E-08	0,1163	0,0076	0,0000	outlier
rs849141	7	28185091	A	G	0,0003	66,0149	-0,0289	0,0036	4,5E-16	-0,0208	0,0082	0,0112	outlier
rs862041	14	74986403	T	C	0,0003	50,5374	0,0240	0,0034	1,2E-12	-0,0017	0,0079	0,8275	instrument
rs9346545	6	168806788	C	A	0,0002	29,9315	0,0197	0,0036	4,5E-08	-0,0092	0,0081	0,2569	instrument
rs9358912	6	26211146	T	G	0,0012	232,2552	0,0554	0,0036	2,0E-52	0,0189	0,0081	0,0191	outlier
rs9391253	6	105367616	T	A	0,0005	93,9339	-0,0335	0,0035	3,3E-22	0,0026	0,0079	0,7456	instrument
rs9398433	6	116753939	G	T	0,0002	43,0200	-0,0219	0,0033	5,4E-11	-0,0010	0,0076	0,9006	instrument
rs9442571	1	9349611	A	T	0,0002	41,2288	-0,0313	0,0049	1,4E-10	-0,0161	0,0123	0,1920	instrument
rs9496567	6	100602753	A	G	0,0002	30,3267	-0,0208	0,0038	3,7E-08	0,0167	0,0099	0,0928	instrument
rs9523325	13	92051677	T	G	0,0002	39,1496	-0,0258	0,0041	3,9E-10	0,0248	0,0093	0,0075	instrument
rs9614671	22	45839056	A	G	0,0002	29,7676	-0,0221	0,0040	4,9E-08	0,0118	0,0094	0,2074	instrument
rs979131	20	6566965	C	T	0,0002	33,8953	-0,0199	0,0034	5,8E-09	-0,0044	0,0078	0,5757	instrument
rs9809116	3	72397279	G	A	0,0002	35,6763	0,0199	0,0033	2,3E-09	-0,0141	0,0079	0,0736	instrument
rs994014	4	82165790	C	T	0,0005	90,3251	-0,0333	0,0035	2,0E-21	-0,0099	0,0081	0,2241	instrument
rs9943184	1	170784430	T	G	0,0002	36,8809	-0,0294	0,0048	1,3E-09	0,0033	0,0111	0,7633	instrument
rs9965184	18	40776606	T	A	0,0002	33,1657	0,0196	0,0034	8,5E-09	-0,0113	0,0078	0,1494	instrument

Table S32. Single Nucleotide Polymorphisms (SNPs) used as instruments in the Mendelian randomization analyses for the effect of LFR on clear cell ovarian cancer based on the genome-wide significance threshold P = 5E-08. R² represents the explained variance in the exposure. SNP-specific instrument strength is given by the respective F-statistic. Some of the genetic variants were identified as outliers in the iterative approach and therefore excluded from the analyses.

SNP	CHR	POS	EA	OA	R2	Fstat	β_{LFR}	SE_{LFR}	P_{LFR}	$\beta_{Clear\ cell}$	$SE_{Clear\ cell}$	$P_{Clear\ cell}$	$variant_{Clear\ cell}$
rs1002436	1	89146852	A	G	0,0002	33,9070	0,0189	0,0032	5,8E-09	-0,0513	0,0406	0,2066	instrument
rs1007358	7	46201355	G	A	0,0002	31,5391	-0,0221	0,0039	2,0E-08	0,0734	0,0488	0,1331	instrument
rs10172196	2	36780549	A	G	0,0003	53,8017	-0,0259	0,0035	2,2E-13	0,0139	0,0441	0,7526	instrument
rs1020048	9	86666993	A	C	0,0002	40,1825	-0,0265	0,0042	2,3E-10	0,0220	0,0505	0,6634	instrument
rs10445034	16	88840564	T	C	0,0002	38,3776	0,0239	0,0039	5,8E-10	-0,1212	0,0490	0,0134	instrument
rs10901171	9	133427194	C	T	0,0002	43,2039	0,0220	0,0033	4,9E-11	0,0735	0,0421	0,0804	instrument
rs11205303	1	149906413	C	T	0,0018	340,7680	-0,0605	0,0033	4,8E-76	0,0691	0,0408	0,0902	instrument
rs112540634	6	34623905	T	C	0,0006	106,9145	-0,0487	0,0047	4,7E-25	0,0507	0,0567	0,3714	instrument
rs11259936	15	84580582	A	C	0,0012	235,0064	0,0495	0,0032	5,0E-53	0,0173	0,0400	0,6648	instrument
rs112635299	14	94838142	T	G	0,0004	70,8115	-0,0942	0,0112	3,9E-17	0,0373	0,1471	0,7998	instrument
rs113059026	10	32158478	A	C	0,0002	42,1586	0,0233	0,0036	8,4E-11	-0,0967	0,0445	0,0297	instrument
rs11466399	1	218591623	A	G	0,0003	63,9993	-0,0287	0,0036	1,3E-15	0,0125	0,0443	0,7773	instrument
rs11666808	19	18383506	T	C	0,0002	46,9631	-0,0230	0,0033	7,2E-12	-0,0085	0,0420	0,8392	instrument
rs11695471	2	25457708	A	T	0,0005	88,6790	0,0324	0,0034	4,7E-21	-0,1060	0,0442	0,0164	instrument
rs12103006	16	24726237	A	G	0,0002	31,2925	0,0183	0,0033	2,2E-08	-0,0555	0,0407	0,1727	instrument
rs12238852	9	98382084	T	C	0,0002	40,8700	-0,0296	0,0046	1,6E-10	0,0709	0,0567	0,2111	instrument
rs12756815	1	113207243	A	C	0,0003	62,4410	0,0316	0,0040	2,7E-15	-0,0029	0,0495	0,9529	instrument
rs13128180	4	56286188	T	G	0,0003	57,4558	-0,0267	0,0035	3,5E-14	-0,0321	0,0435	0,4600	instrument
rs1313237	14	60848224	C	A	0,0003	50,8506	-0,0236	0,0033	1,0E-12	-0,0459	0,0414	0,2679	instrument
rs132941	22	38545942	C	T	0,0003	64,2716	-0,0261	0,0033	1,1E-15	-0,0295	0,0401	0,4617	instrument
rs13306455	19	7184721	T	C	0,0002	32,1259	-0,0244	0,0043	1,4E-08	0,0601	0,0524	0,2518	instrument
rs138809270	2	219929394	T	C	0,0002	39,7778	0,0828	0,0131	2,8E-10	-0,0737	0,1582	0,6411	instrument
rs143384	20	34025756	G	A	0,0009	179,8262	-0,0441	0,0033	5,4E-41	-0,0305	0,0409	0,4556	instrument
rs143840904	11	2813322	T	C	0,0002	44,7289	0,0842	0,0126	2,3E-11	0,0848	0,1937	0,6616	instrument
rs1452815	8	78123634	G	A	0,0002	30,3818	-0,0197	0,0036	3,6E-08	0,0722	0,0438	0,0989	instrument
rs1513304	3	99269750	C	T	0,0002	40,5765	-0,0226	0,0035	1,9E-10	0,0025	0,0464	0,9570	instrument
rs1550436	15	74221157	T	C	0,0004	77,3160	0,0286	0,0032	1,5E-18	0,0171	0,0400	0,6682	instrument

rs16942324	15	89383854	A	C	0,0004	70,6937	0,0844	0,0100	4,2E-17	0,0555	0,1307	0,6712	instrument
rs16948048	17	47440466	G	A	0,0002	42,5621	0,0218	0,0033	6,9E-11	-0,0375	0,0412	0,3628	instrument
rs16953461	17	46261736	G	T	0,0003	54,8186	0,0292	0,0039	1,3E-13	0,0019	0,0501	0,9705	instrument
rs16972537	16	73802389	A	G	0,0002	33,0736	-0,0194	0,0034	8,9E-09	-0,0118	0,0423	0,7807	instrument
rs17698176	17	44819595	G	T	0,0003	62,1883	-0,0307	0,0039	3,1E-15	-0,0135	0,0534	0,8001	instrument
rs17744040	9	16842137	C	T	0,0002	34,5740	0,0309	0,0053	4,1E-09	-0,0050	0,0608	0,9350	instrument
rs1801282	3	12393125	G	C	0,0003	51,5519	-0,0356	0,0050	7,0E-13	-0,0344	0,0614	0,5751	instrument
rs1980030	10	120970027	G	A	0,0002	32,0806	-0,0186	0,0033	1,5E-08	0,0610	0,0413	0,1397	instrument
rs2007482	6	2169724	T	C	0,0002	36,6626	0,0199	0,0033	1,4E-09	0,0020	0,0414	0,9621	instrument
rs2058908	16	53806145	T	C	0,0002	44,3951	0,0243	0,0037	2,7E-11	-0,0522	0,0461	0,2578	instrument
rs2126643	1	103404384	T	C	0,0002	40,1318	-0,0218	0,0034	2,4E-10	0,0517	0,0420	0,2181	instrument
rs2158694	7	2863443	G	T	0,0008	161,7967	0,0450	0,0035	4,5E-37	-0,0657	0,0444	0,1391	instrument
rs2173295	1	54943264	C	A	0,0002	33,0737	0,0276	0,0048	8,9E-09	-0,0993	0,0632	0,1163	instrument
rs2274432	1	184020945	A	G	0,0005	86,6007	-0,0315	0,0034	1,3E-20	0,0135	0,0421	0,7476	instrument
rs2293576	11	47434986	A	G	0,0003	48,9295	0,0240	0,0034	2,7E-12	0,0346	0,0421	0,4104	instrument
rs2370983	14	79903376	G	A	0,0002	33,4890	0,0196	0,0034	7,2E-09	0,0575	0,0428	0,1785	instrument
rs2516633	11	62371773	A	C	0,0002	31,4718	0,0197	0,0035	2,0E-08	-0,0196	0,0450	0,6621	instrument
rs256904	5	55810305	A	T	0,0003	66,6012	-0,0302	0,0037	3,3E-16	-0,0619	0,0471	0,1891	instrument
rs2641699	16	84886306	C	G	0,0002	35,6047	0,0200	0,0034	2,4E-09	0,0305	0,0424	0,4729	instrument
rs264762	5	64384386	A	C	0,0003	52,4171	0,0248	0,0034	4,5E-13	-0,1148	0,0431	0,0077	instrument
rs2712169	2	217671349	G	A	0,0003	49,6879	-0,0233	0,0033	1,8E-12	0,0395	0,0405	0,3301	instrument
rs2715094	7	50730452	G	A	0,0003	57,7290	-0,0282	0,0037	3,0E-14	0,0788	0,0465	0,0897	instrument
rs2768641	9	96948863	G	A	0,0002	34,4213	-0,0455	0,0078	4,4E-09	-0,1504	0,1034	0,1459	instrument
rs2811462	3	129303436	G	T	0,0003	52,3447	-0,0281	0,0039	4,7E-13	-0,0307	0,0492	0,5334	instrument
rs2820446	1	219748818	G	C	0,0007	132,7090	-0,0405	0,0035	1,1E-30	0,0778	0,0440	0,0771	instrument
rs2844795	6	30073847	C	T	0,0002	41,4345	-0,0208	0,0032	1,2E-10	0,0828	0,0407	0,0419	instrument
rs28539246	17	29226630	A	T	0,0003	48,5804	0,0231	0,0033	3,2E-12	-0,0391	0,0413	0,3437	instrument
rs28716235	8	135602976	C	A	0,0002	33,4427	0,0194	0,0034	7,4E-09	-0,0534	0,0418	0,2015	instrument
rs2886564	1	42496924	G	A	0,0002	31,4381	-0,0200	0,0036	2,1E-08	-0,0027	0,0440	0,9506	instrument
rs3020421	6	152348122	A	G	0,0002	35,0224	-0,0206	0,0035	3,3E-09	-0,0742	0,0434	0,0870	instrument
rs3218036	19	30305684	A	G	0,0002	29,8550	-0,0188	0,0034	4,7E-08	-0,0624	0,0428	0,1451	instrument

rs34517439	1	78450517	A	C	0,0003	65,2049	-0,0403	0,0050	6,8E-16	-0,0774	0,0654	0,2362	instrument
rs34580448	5	82810884	C	T	0,0004	79,5477	0,0727	0,0081	4,8E-19	0,0587	0,1032	0,5695	instrument
rs35307904	9	78511889	A	G	0,0006	116,4229	0,0537	0,0050	3,8E-27	0,0279	0,0644	0,6661	instrument
rs35874463	15	67457698	G	A	0,0003	66,0962	-0,0563	0,0069	4,3E-16	-0,1731	0,0936	0,0642	instrument
rs3738814	1	17331676	G	A	0,0003	60,4500	0,0253	0,0033	7,6E-15	-0,0997	0,0405	0,0139	instrument
rs3791679	2	56096892	G	A	0,0009	179,2902	0,0516	0,0039	7,3E-41	-0,0321	0,0476	0,4992	instrument
rs3794782	17	18127914	C	G	0,0002	33,0277	-0,0191	0,0033	9,1E-09	-0,0552	0,0413	0,1811	instrument
rs3823974	7	20442796	C	T	0,0003	60,9486	0,0258	0,0033	5,9E-15	-0,0262	0,0407	0,5203	instrument
rs3850639	1	172253634	C	T	0,0003	56,8058	-0,0297	0,0039	4,8E-14	0,0508	0,0484	0,2944	instrument
rs4073717	5	170864021	T	G	0,0002	33,2233	0,0233	0,0040	8,2E-09	-0,0432	0,0509	0,3964	instrument
rs41284816	13	50655989	T	G	0,0002	46,3484	-0,0835	0,0123	9,9E-12	0,1337	0,1317	0,3100	instrument
rs41313250	1	62483623	G	A	0,0002	36,9903	0,0319	0,0052	1,2E-09	0,0236	0,0607	0,6981	instrument
rs4236324	7	27302727	C	T	0,0002	37,5030	-0,0274	0,0045	9,1E-10	-0,0460	0,0560	0,4115	instrument
rs4246308	15	100765202	A	G	0,0003	59,4589	0,0250	0,0032	1,3E-14	0,0159	0,0400	0,6903	instrument
rs4321401	2	69348216	T	C	0,0003	48,9715	0,0227	0,0032	2,6E-12	-0,0159	0,0434	0,7148	instrument
rs4421120	5	32763118	A	G	0,0003	50,1542	-0,0311	0,0044	1,4E-12	0,0514	0,0550	0,3501	instrument
rs4432932	5	115039414	G	A	0,0002	31,1249	-0,0204	0,0037	2,4E-08	0,0127	0,0441	0,7735	instrument
rs4518624	8	130715510	A	G	0,0002	38,0685	0,0203	0,0033	6,8E-10	0,0514	0,0408	0,2073	instrument
rs454345	5	88729851	G	A	0,0002	33,8255	-0,0194	0,0033	6,0E-09	-0,0350	0,0417	0,4008	instrument
rs4803461	19	41908871	G	A	0,0002	33,2003	-0,0190	0,0033	8,3E-09	-0,0546	0,0416	0,1889	instrument
rs4842133	9	139127566	A	C	0,0004	69,4715	-0,0300	0,0036	7,8E-17	-0,0093	0,0461	0,8395	instrument
rs4901541	14	55235282	C	T	0,0002	40,4873	0,0211	0,0033	2,0E-10	-0,0561	0,0427	0,1888	instrument
rs4936122	11	130734264	C	A	0,0002	30,3818	-0,0181	0,0033	3,6E-08	0,0342	0,0409	0,4032	instrument
rs496298	10	131355130	A	G	0,0003	65,4151	0,0276	0,0034	6,1E-16	-0,0897	0,0425	0,0350	instrument
rs4973032	2	232990462	T	G	0,0002	40,7423	0,0769	0,0121	1,7E-10	-0,2139	0,1527	0,1612	instrument
rs4974072	3	41222586	A	G	0,0002	43,3091	-0,0214	0,0033	4,7E-11	0,0179	0,0402	0,6558	instrument
rs538656	18	57850422	T	G	0,0002	31,4269	-0,0213	0,0038	2,1E-08	0,0202	0,0468	0,6665	instrument
rs544148	6	117466494	A	G	0,0002	30,7134	0,0182	0,0033	3,0E-08	0,0261	0,0406	0,5206	instrument
rs55633655	5	131598756	T	G	0,0003	53,1144	0,0237	0,0033	3,2E-13	0,0008	0,0401	0,9837	instrument
rs56021161	14	64817100	A	T	0,0002	30,5916	0,0362	0,0065	3,2E-08	0,0513	0,0819	0,5316	instrument
rs56186137	16	28825953	G	A	0,0006	108,1589	-0,0341	0,0033	2,6E-25	0,0709	0,0411	0,0845	instrument

rs56277550	21	18092263	A	G	0,0002	33,5006	0,0187	0,0032	7,1E-09	-0,0145	0,0404	0,7203	instrument
rs57674902	20	4096741	T	C	0,0002	38,1302	-0,0210	0,0034	6,6E-10	-0,0058	0,0435	0,8936	instrument
rs584961	11	75277628	A	G	0,0002	35,1408	-0,0302	0,0051	3,1E-09	0,0530	0,0645	0,4113	instrument
rs61734651	20	61451332	T	C	0,0002	37,8836	-0,0421	0,0068	7,5E-10	-0,0706	0,0917	0,4413	instrument
rs61834590	1	227421117	G	T	0,0002	43,2696	0,0298	0,0045	4,8E-11	-0,0035	0,0564	0,9506	instrument
rs62106258	2	417167	C	T	0,0002	32,2053	0,0424	0,0075	1,4E-08	-0,1763	0,1121	0,1157	instrument
rs62271373	3	150066540	A	T	0,0002	41,1775	0,0455	0,0071	1,4E-10	-0,0361	0,0932	0,6982	instrument
rs62515452	8	57187356	T	C	0,0002	34,2338	-0,0267	0,0046	4,9E-09	0,0471	0,0601	0,4326	instrument
rs62621197	19	8670147	T	C	0,0008	150,7968	0,1140	0,0093	1,1E-34	-0,1894	0,1422	0,1827	instrument
rs640245	11	30393360	T	C	0,0002	37,0512	-0,0198	0,0033	1,1E-09	-0,0320	0,0403	0,4265	instrument
rs6486059	11	12824581	A	G	0,0003	52,4315	-0,0238	0,0033	4,5E-13	-0,0491	0,0414	0,2358	instrument
rs6503410	17	43094140	T	C	0,0002	35,0934	0,0215	0,0036	3,2E-09	-0,0069	0,0459	0,8804	instrument
rs6585827	10	124165615	A	G	0,0002	45,0505	-0,0217	0,0032	1,9E-11	0,0200	0,0405	0,6220	instrument
rs6717858	2	165539661	C	T	0,0003	64,7696	-0,0265	0,0033	8,5E-16	-0,0064	0,0406	0,8744	instrument
rs6739701	2	105132332	G	A	0,0002	44,8493	-0,0220	0,0033	2,1E-11	-0,0410	0,0422	0,3309	instrument
rs6785012	3	141109348	T	C	0,0012	231,9505	-0,0496	0,0033	2,3E-52	-0,0052	0,0403	0,8980	instrument
rs68049170	10	72432047	A	G	0,0003	56,1594	-0,0273	0,0036	6,7E-14	0,0668	0,0448	0,1362	instrument
rs6843340	4	48619937	T	C	0,0002	30,9912	-0,0180	0,0032	2,6E-08	-0,0789	0,0400	0,0486	instrument
rs6887247	5	157881647	C	T	0,0003	53,8897	-0,0257	0,0035	2,1E-13	0,0678	0,0437	0,1206	instrument
rs702605	5	53167028	T	C	0,0002	34,1286	-0,0189	0,0032	5,2E-09	-0,0743	0,0403	0,0650	instrument
rs71531541	6	7066481	C	T	0,0002	30,1727	0,0200	0,0036	4,0E-08	0,0262	0,0461	0,5690	instrument
rs7155279	14	92485881	T	G	0,0003	47,9967	0,0233	0,0034	4,3E-12	0,0192	0,0419	0,6462	instrument
rs7170686	15	48821350	A	G	0,0002	36,2761	0,0334	0,0055	1,7E-09	-0,1126	0,0711	0,1133	instrument
rs7191697	16	89678165	A	G	0,0002	46,8946	0,0369	0,0054	7,5E-12	0,0151	0,0720	0,8336	instrument
rs7234167	18	46744610	C	G	0,0002	30,4480	0,0206	0,0037	3,4E-08	0,0125	0,0459	0,7857	instrument
rs7244464	18	20729714	A	C	0,0005	90,7247	0,0372	0,0039	1,7E-21	-0,0081	0,0491	0,8688	instrument
rs72708236	1	155980320	G	T	0,0003	54,7890	0,0703	0,0095	1,3E-13	0,0492	0,1217	0,6861	instrument
rs72755233	15	100692953	A	G	0,0008	160,7807	0,0645	0,0051	7,6E-37	-0,0926	0,0791	0,2423	instrument
rs72776438	5	77438797	T	G	0,0002	36,1798	0,0228	0,0038	1,8E-09	-0,0081	0,0491	0,8692	instrument
rs73004967	19	19717056	G	A	0,0002	40,6530	0,0405	0,0064	1,8E-10	0,0777	0,0806	0,3351	instrument
rs73175572	3	185490184	G	A	0,0002	36,0236	-0,0314	0,0052	2,0E-09	0,0292	0,0655	0,6565	instrument

rs732563	8	23345526	T	C	0,0002	41,4088	0,0208	0,0032	1,2E-10	-0,0290	0,0399	0,4683	instrument
rs73407643	6	35169123	C	T	0,0002	36,6869	0,0751	0,0124	1,4E-09	0,1468	0,1512	0,3318	instrument
rs741207	4	18321092	T	C	0,0002	35,8677	-0,0200	0,0033	2,1E-09	0,0087	0,0408	0,8306	instrument
rs7502307	17	65989961	G	C	0,0003	54,1543	-0,0300	0,0041	1,9E-13	0,0317	0,0499	0,5252	instrument
rs75223147	6	19831020	G	T	0,0003	56,7154	-0,0848	0,0113	5,1E-14	0,1598	0,1922	0,4057	instrument
rs75621580	4	12916514	A	G	0,0002	30,7689	0,0425	0,0077	2,9E-08	0,0361	0,1004	0,7195	instrument
rs7626449	3	70921329	A	G	0,0002	39,4254	-0,0225	0,0036	3,4E-10	0,0240	0,0439	0,5855	instrument
rs7641375	3	57568444	A	C	0,0002	43,0725	-0,0219	0,0033	5,3E-11	-0,0444	0,0419	0,2888	instrument
rs7689420	4	145568352	T	C	0,0008	162,5608	0,0548	0,0043	3,4E-37	-0,1967	0,0565	0,0005	outlier
rs7697556	4	73515313	T	C	0,0005	96,6869	-0,0318	0,0032	8,2E-23	0,0642	0,0409	0,1167	instrument
rs7718768	5	178536089	A	G	0,0003	62,8525	-0,0271	0,0034	2,2E-15	-0,1289	0,0435	0,0031	outlier
rs7733331	5	32828846	T	C	0,0002	34,2221	-0,0193	0,0033	4,9E-09	0,0311	0,0418	0,4570	instrument
rs7741741	6	142655801	A	T	0,0005	102,6158	0,0362	0,0036	4,2E-24	0,0056	0,0445	0,9005	instrument
rs7752292	6	131317876	A	T	0,0002	42,4187	-0,0218	0,0033	7,4E-11	-0,0044	0,0416	0,9164	instrument
rs7804293	7	92287849	G	T	0,0004	73,9248	-0,0315	0,0037	8,1E-18	0,0286	0,0449	0,5241	instrument
rs78198962	2	233094868	T	C	0,0002	29,7349	-0,0499	0,0092	5,0E-08	-0,1269	0,1397	0,3636	instrument
rs78821460	5	127874149	C	G	0,0002	32,3189	0,0330	0,0058	1,3E-08	-0,0041	0,0723	0,9546	instrument
rs79312203	20	32082962	A	G	0,0002	36,7596	-0,0326	0,0054	1,3E-09	0,0141	0,0668	0,8324	instrument
rs7952436	11	67024534	T	C	0,0006	119,9012	0,0637	0,0058	6,7E-28	-0,0751	0,0885	0,3962	instrument
rs7994151	13	115038879	G	A	0,0002	41,0364	0,0289	0,0045	1,5E-10	0,1141	0,0550	0,0380	instrument
rs8003703	14	33411639	A	T	0,0002	31,7078	0,0642	0,0114	1,8E-08	0,1440	0,1517	0,3426	instrument
rs80085026	7	148636421	G	C	0,0003	55,0558	-0,0308	0,0042	1,2E-13	-0,0009	0,0523	0,9869	instrument
rs8103728	19	33900257	C	G	0,0004	82,6818	0,0313	0,0034	9,7E-20	0,0232	0,0425	0,5857	instrument
rs832540	5	56199202	G	A	0,0002	32,1826	0,0193	0,0034	1,4E-08	-0,0234	0,0420	0,5769	instrument
rs849141	7	28185091	A	G	0,0003	66,0149	-0,0289	0,0036	4,5E-16	0,0183	0,0443	0,6792	instrument
rs862041	14	74986403	T	C	0,0003	50,5374	0,0240	0,0034	1,2E-12	0,0322	0,0422	0,4453	instrument
rs9346545	6	168806788	C	A	0,0002	29,9315	0,0197	0,0036	4,5E-08	-0,0249	0,0448	0,5778	instrument
rs9358912	6	26211146	T	G	0,0012	232,2552	0,0554	0,0036	2,0E-52	0,0556	0,0438	0,2041	instrument
rs9391253	6	105367616	T	A	0,0005	93,9339	-0,0335	0,0035	3,3E-22	0,0253	0,0426	0,5528	instrument
rs9398433	6	116753939	G	T	0,0002	43,0200	-0,0219	0,0033	5,4E-11	0,0687	0,0407	0,0920	instrument
rs9442571	1	9349611	A	T	0,0002	41,2288	-0,0313	0,0049	1,4E-10	-0,0294	0,0641	0,6465	instrument

rs9496567	6	100602753	A	G	0,0002	30,3267	-0,0208	0,0038	3,7E-08	-0,0494	0,0505	0,3276	instrument
rs9523325	13	92051677	T	G	0,0002	39,1496	-0,0258	0,0041	3,9E-10	-0,1155	0,0511	0,0238	instrument
rs9614671	22	45839056	A	G	0,0002	29,7676	-0,0221	0,0040	4,9E-08	0,0298	0,0504	0,5545	instrument
rs979131	20	6566965	C	T	0,0002	33,8953	-0,0199	0,0034	5,8E-09	-0,0515	0,0420	0,2195	instrument
rs9809116	3	72397279	G	A	0,0002	35,6763	0,0199	0,0033	2,3E-09	-0,0407	0,0417	0,3290	instrument
rs994014	4	82165790	C	T	0,0005	90,3251	-0,0333	0,0035	2,0E-21	0,0006	0,0434	0,9896	instrument
rs9943184	1	170784430	T	G	0,0002	36,8809	-0,0294	0,0048	1,3E-09	-0,0567	0,0610	0,3524	instrument
rs9965184	18	40776606	T	A	0,0002	33,1657	0,0196	0,0034	8,5E-09	0,0452	0,0422	0,2842	instrument

Table S33. Single Nucleotide Polymorphisms (SNPs) used as instruments in the Mendelian randomization analyses for the effect of LFR on endometrioid ovarian cancer based on the genome-wide significance threshold P = 5E-08. R^2 represents the explained variance in the exposure. SNP-specific instrument strength is given by the respective F-statistic. Some of the genetic variants were identified as outliers in the iterative approach and therefore excluded from the analyses.

SNP	CHR	POS	EA	OA	R2	Fstat	β_{LFR}	SE_{LFR}	P_{LFR}	$\beta_{Endometrioid}$	$SE_{Endometrioid}$	$P_{Endometrioid}$	$variant_{Endometrioid}$
rs1002436	1	89146852	A	G	0,0002	33,9070	0,0189	0,0032	5,8E-09	-0,0196	0,0291	0,4992	instrument
rs1007358	7	46201355	G	A	0,0002	31,5391	-0,0221	0,0039	2,0E-08	-0,0086	0,0354	0,8073	instrument
rs10172196	2	36780549	A	G	0,0003	53,8017	-0,0259	0,0035	2,2E-13	-0,0101	0,0315	0,7487	instrument
rs1020048	9	86666993	A	C	0,0002	40,1825	-0,0265	0,0042	2,3E-10	0,0010	0,0362	0,9782	instrument
rs10445034	16	88840564	T	C	0,0002	38,3776	0,0239	0,0039	5,8E-10	-0,0421	0,0345	0,2228	instrument
rs10901171	9	133427194	C	T	0,0002	43,2039	0,0220	0,0033	4,9E-11	-0,0198	0,0305	0,5161	instrument
rs11205303	1	149906413	C	T	0,0018	340,7680	-0,0605	0,0033	4,8E-76	0,0015	0,0293	0,9587	instrument
rs112540634	6	34623905	T	C	0,0006	106,9145	-0,0487	0,0047	4,7E-25	-0,0402	0,0414	0,3319	instrument
rs11259936	15	84580582	A	C	0,0012	235,0064	0,0495	0,0032	5,0E-53	0,0009	0,0286	0,9754	instrument
rs112635299	14	94838142	T	G	0,0004	70,8115	-0,0942	0,0112	3,9E-17	0,0353	0,1070	0,7417	instrument
rs113059026	10	32158478	A	C	0,0002	42,1586	0,0233	0,0036	8,4E-11	-0,0011	0,0315	0,9718	instrument
rs11466399	1	218591623	A	G	0,0003	63,9993	-0,0287	0,0036	1,3E-15	-0,0553	0,0319	0,0831	instrument
rs11666808	19	18383506	T	C	0,0002	46,9631	-0,0230	0,0033	7,2E-12	0,0335	0,0299	0,2616	instrument
rs11695471	2	25457708	A	T	0,0005	88,6790	0,0324	0,0034	4,7E-21	-0,0755	0,0317	0,0173	instrument
rs12103006	16	24726237	A	G	0,0002	31,2925	0,0183	0,0033	2,2E-08	0,0537	0,0290	0,0638	instrument
rs12238852	9	98382084	T	C	0,0002	40,8700	-0,0296	0,0046	1,6E-10	0,0344	0,0411	0,4018	instrument
rs12756815	1	113207243	A	C	0,0003	62,4410	0,0316	0,0040	2,7E-15	-0,0311	0,0355	0,3811	instrument
rs13128180	4	56286188	T	G	0,0003	57,4558	-0,0267	0,0035	3,5E-14	0,0492	0,0308	0,1100	instrument

rs1313237	14	60848224	C	A	0,0003	50,8506	-0,0236	0,0033	1,0E-12	0,0395	0,0294	0,1796	instrument
rs132941	22	38545942	C	T	0,0003	64,2716	-0,0261	0,0033	1,1E-15	-0,0180	0,0287	0,5307	instrument
rs13306455	19	7184721	T	C	0,0002	32,1259	-0,0244	0,0043	1,4E-08	-0,0589	0,0385	0,1257	instrument
rs138809270	2	219929394	T	C	0,0002	39,7778	0,0828	0,0131	2,8E-10	-0,0243	0,1104	0,8258	instrument
rs143384	20	34025756	G	A	0,0009	179,8262	-0,0441	0,0033	5,4E-41	0,0006	0,0292	0,9841	instrument
rs143840904	11	2813322	T	C	0,0002	44,7289	0,0842	0,0126	2,3E-11	-0,0519	0,1402	0,7112	instrument
rs1452815	8	78123634	G	A	0,0002	30,3818	-0,0197	0,0036	3,6E-08	-0,0415	0,0318	0,1920	instrument
rs1513304	3	99269750	C	T	0,0002	40,5765	-0,0226	0,0035	1,9E-10	0,0238	0,0333	0,4744	instrument
rs1550436	15	74221157	T	C	0,0004	77,3160	0,0286	0,0032	1,5E-18	0,0102	0,0286	0,7219	instrument
rs16942324	15	89383854	A	C	0,0004	70,6937	0,0844	0,0100	4,2E-17	-0,0464	0,0972	0,6332	instrument
rs16948048	17	47440466	G	A	0,0002	42,5621	0,0218	0,0033	6,9E-11	-0,0225	0,0295	0,4457	instrument
rs16953461	17	46261736	G	T	0,0003	54,8186	0,0292	0,0039	1,3E-13	0,0186	0,0358	0,6039	instrument
rs16972537	16	73802389	A	G	0,0002	33,0736	-0,0194	0,0034	8,9E-09	0,0142	0,0303	0,6397	instrument
rs17698176	17	44819595	G	T	0,0003	62,1883	-0,0307	0,0039	3,1E-15	-0,0245	0,0386	0,5254	instrument
rs17744040	9	16842137	C	T	0,0002	34,5740	0,0309	0,0053	4,1E-09	-0,0524	0,0441	0,2325	instrument
rs1801282	3	12393125	G	C	0,0003	51,5519	-0,0356	0,0050	7,0E-13	-0,0851	0,0443	0,0545	instrument
rs1980030	10	120970027	G	A	0,0002	32,0806	-0,0186	0,0033	1,5E-08	0,0211	0,0296	0,4766	instrument
rs2007482	6	2169724	T	C	0,0002	36,6626	0,0199	0,0033	1,4E-09	-0,0321	0,0298	0,2825	instrument
rs2058908	16	53806145	T	C	0,0002	44,3951	0,0243	0,0037	2,7E-11	0,0164	0,0329	0,6175	instrument
rs2126643	1	103404384	T	C	0,0002	40,1318	-0,0218	0,0034	2,4E-10	-0,0388	0,0302	0,1991	instrument
rs2158694	7	2863443	G	T	0,0008	161,7967	0,0450	0,0035	4,5E-37	0,0061	0,0313	0,8450	instrument
rs2173295	1	54943264	C	A	0,0002	33,0737	0,0276	0,0048	8,9E-09	0,0006	0,0445	0,9901	instrument
rs2274432	1	184020945	A	G	0,0005	86,6007	-0,0315	0,0034	1,3E-20	-0,0664	0,0303	0,0287	instrument
rs2293576	11	47434986	A	G	0,0003	48,9295	0,0240	0,0034	2,7E-12	0,0094	0,0302	0,7554	instrument
rs2370983	14	79903376	G	A	0,0002	33,4890	0,0196	0,0034	7,2E-09	-0,0179	0,0309	0,5612	instrument
rs2516633	11	62371773	A	C	0,0002	31,4718	0,0197	0,0035	2,0E-08	0,0335	0,0323	0,3003	instrument
rs256904	5	55810305	A	T	0,0003	66,6012	-0,0302	0,0037	3,3E-16	0,0232	0,0334	0,4875	instrument
rs2641699	16	84886306	C	G	0,0002	35,6047	0,0200	0,0034	2,4E-09	0,0461	0,0306	0,1312	instrument
rs264762	5	64384386	A	C	0,0003	52,4171	0,0248	0,0034	4,5E-13	-0,0162	0,0305	0,5953	instrument
rs2712169	2	217671349	G	A	0,0003	49,6879	-0,0233	0,0033	1,8E-12	0,0452	0,0290	0,1194	instrument
rs2715094	7	50730452	G	A	0,0003	57,7290	-0,0282	0,0037	3,0E-14	-0,0046	0,0340	0,8922	instrument

rs2768641	9	96948863	G	A	0,0002	34,4213	-0,0455	0,0078	4,4E-09	-0,0219	0,0717	0,7603	instrument
rs2811462	3	129303436	G	T	0,0003	52,3447	-0,0281	0,0039	4,7E-13	0,0902	0,0346	0,0091	outlier
rs2820446	1	219748818	G	C	0,0007	132,7090	-0,0405	0,0035	1,1E-30	-0,0313	0,0321	0,3296	instrument
rs2844795	6	30073847	C	T	0,0002	41,4345	-0,0208	0,0032	1,2E-10	-0,0148	0,0293	0,6137	instrument
rs28539246	17	29226630	A	T	0,0003	48,5804	0,0231	0,0033	3,2E-12	-0,0233	0,0294	0,4278	instrument
rs28716235	8	135602976	C	A	0,0002	33,4427	0,0194	0,0034	7,4E-09	-0,0521	0,0298	0,0809	instrument
rs2886564	1	42496924	G	A	0,0002	31,4381	-0,0200	0,0036	2,1E-08	0,0151	0,0314	0,6304	instrument
rs3020421	6	152348122	A	G	0,0002	35,0224	-0,0206	0,0035	3,3E-09	0,0504	0,0305	0,0982	instrument
rs3218036	19	30305684	A	G	0,0002	29,8550	-0,0188	0,0034	4,7E-08	0,0218	0,0303	0,4717	instrument
rs34517439	1	78450517	A	C	0,0003	65,2049	-0,0403	0,0050	6,8E-16	-0,0488	0,0471	0,3001	instrument
rs34580448	5	82810884	C	T	0,0004	79,5477	0,0727	0,0081	4,8E-19	-0,0908	0,0769	0,2372	instrument
rs35307904	9	78511889	A	G	0,0006	116,4229	0,0537	0,0050	3,8E-27	0,0239	0,0469	0,6111	instrument
rs35874463	15	67457698	G	A	0,0003	66,0962	-0,0563	0,0069	4,3E-16	-0,0966	0,0659	0,1425	instrument
rs3738814	1	17331676	G	A	0,0003	60,4500	0,0253	0,0033	7,6E-15	-0,0481	0,0288	0,0952	instrument
rs3791679	2	56096892	G	A	0,0009	179,2902	0,0516	0,0039	7,3E-41	-0,0184	0,0336	0,5840	instrument
rs3794782	17	18127914	C	G	0,0002	33,0277	-0,0191	0,0033	9,1E-09	0,0270	0,0294	0,3586	instrument
rs3823974	7	20442796	C	T	0,0003	60,9486	0,0258	0,0033	5,9E-15	-0,0073	0,0290	0,8002	instrument
rs3850639	1	172253634	C	T	0,0003	56,8058	-0,0297	0,0039	4,8E-14	0,0604	0,0346	0,0806	instrument
rs4073717	5	170864021	T	G	0,0002	33,2233	0,0233	0,0040	8,2E-09	-0,0036	0,0361	0,9213	instrument
rs41284816	13	50655989	T	G	0,0002	46,3484	-0,0835	0,0123	9,9E-12	0,0135	0,0973	0,8893	instrument
rs41313250	1	62483623	G	A	0,0002	36,9903	0,0319	0,0052	1,2E-09	0,0797	0,0428	0,0627	instrument
rs4236324	7	27302727	C	T	0,0002	37,5030	-0,0274	0,0045	9,1E-10	-0,0288	0,0400	0,4722	instrument
rs4246308	15	100765202	A	G	0,0003	59,4589	0,0250	0,0032	1,3E-14	-0,0206	0,0286	0,4712	instrument
rs4321401	2	69348216	T	C	0,0003	48,9715	0,0227	0,0032	2,6E-12	-0,0294	0,0311	0,3443	instrument
rs4421120	5	32763118	A	G	0,0003	50,1542	-0,0311	0,0044	1,4E-12	0,0031	0,0401	0,9375	instrument
rs4432932	5	115039414	G	A	0,0002	31,1249	-0,0204	0,0037	2,4E-08	-0,0445	0,0318	0,1616	instrument
rs4518624	8	130715510	A	G	0,0002	38,0685	0,0203	0,0033	6,8E-10	0,0439	0,0292	0,1336	instrument
rs454345	5	88729851	G	A	0,0002	33,8255	-0,0194	0,0033	6,0E-09	-0,0616	0,0299	0,0395	instrument
rs4803461	19	41908871	G	A	0,0002	33,2003	-0,0190	0,0033	8,3E-09	0,0176	0,0296	0,5523	instrument
rs4842133	9	139127566	A	C	0,0004	69,4715	-0,0300	0,0036	7,8E-17	0,0072	0,0329	0,8272	instrument
rs4901541	14	55235282	C	T	0,0002	40,4873	0,0211	0,0033	2,0E-10	0,0150	0,0306	0,6250	instrument

rs4936122	11	130734264	C	A	0,0002	30,3818	-0,0181	0,0033	3,6E-08	-0,0342	0,0295	0,2466	instrument
rs496298	10	131355130	A	G	0,0003	65,4151	0,0276	0,0034	6,1E-16	-0,0089	0,0302	0,7682	instrument
rs4973032	2	232990462	T	G	0,0002	40,7423	0,0769	0,0121	1,7E-10	-0,0577	0,1053	0,5840	instrument
rs4974072	3	41222586	A	G	0,0002	43,3091	-0,0214	0,0033	4,7E-11	-0,0014	0,0288	0,9621	instrument
rs538656	18	57850422	T	G	0,0002	31,4269	-0,0213	0,0038	2,1E-08	0,0516	0,0333	0,1215	instrument
rs544148	6	117466494	A	G	0,0002	30,7134	0,0182	0,0033	3,0E-08	0,0462	0,0291	0,1124	instrument
rs55633655	5	131598756	T	G	0,0003	53,1144	0,0237	0,0033	3,2E-13	-0,0153	0,0287	0,5939	instrument
rs56021161	14	64817100	A	T	0,0002	30,5916	0,0362	0,0065	3,2E-08	0,0793	0,0585	0,1749	instrument
rs56186137	16	28825953	G	A	0,0006	108,1589	-0,0341	0,0033	2,6E-25	0,0697	0,0294	0,0179	instrument
rs56277550	21	18092263	A	G	0,0002	33,5006	0,0187	0,0032	7,1E-09	-0,0187	0,0290	0,5180	instrument
rs57674902	20	4096741	T	C	0,0002	38,1302	-0,0210	0,0034	6,6E-10	0,0274	0,0312	0,3805	instrument
rs584961	11	75277628	A	G	0,0002	35,1408	-0,0302	0,0051	3,1E-09	0,0577	0,0467	0,2158	instrument
rs61734651	20	61451332	T	C	0,0002	37,8836	-0,0421	0,0068	7,5E-10	-0,0076	0,0652	0,9073	instrument
rs61834590	1	227421117	G	T	0,0002	43,2696	0,0298	0,0045	4,8E-11	-0,0236	0,0405	0,5601	instrument
rs62106258	2	417167	C	T	0,0002	32,2053	0,0424	0,0075	1,4E-08	-0,1031	0,0793	0,1938	instrument
rs62271373	3	150066540	A	T	0,0002	41,1775	0,0455	0,0071	1,4E-10	0,1071	0,0649	0,0986	instrument
rs62515452	8	57187356	T	C	0,0002	34,2338	-0,0267	0,0046	4,9E-09	-0,0321	0,0437	0,4633	instrument
rs62621197	19	8670147	T	C	0,0008	150,7968	0,1140	0,0093	1,1E-34	0,0270	0,0964	0,7798	instrument
rs640245	11	30393360	T	C	0,0002	37,0512	-0,0198	0,0033	1,1E-09	-0,0362	0,0289	0,2102	instrument
rs6486059	11	12824581	A	G	0,0003	52,4315	-0,0238	0,0033	4,5E-13	-0,0657	0,0296	0,0263	instrument
rs6503410	17	43094140	T	C	0,0002	35,0934	0,0215	0,0036	3,2E-09	-0,0049	0,0328	0,8802	instrument
rs6585827	10	124165615	A	G	0,0002	45,0505	-0,0217	0,0032	1,9E-11	0,0251	0,0290	0,3857	instrument
rs6717858	2	165539661	C	T	0,0003	64,7696	-0,0265	0,0033	8,5E-16	-0,0216	0,0292	0,4590	instrument
rs6739701	2	105132332	G	A	0,0002	44,8493	-0,0220	0,0033	2,1E-11	-0,0124	0,0305	0,6854	instrument
rs6785012	3	141109348	T	C	0,0012	231,9505	-0,0496	0,0033	2,3E-52	0,0107	0,0288	0,7112	instrument
rs68049170	10	72432047	A	G	0,0003	56,1594	-0,0273	0,0036	6,7E-14	0,0553	0,0321	0,0851	instrument
rs6843340	4	48619937	T	C	0,0002	30,9912	-0,0180	0,0032	2,6E-08	0,0131	0,0286	0,6469	instrument
rs6887247	5	157881647	C	T	0,0003	53,8897	-0,0257	0,0035	2,1E-13	0,0218	0,0315	0,4898	instrument
rs702605	5	53167028	T	C	0,0002	34,1286	-0,0189	0,0032	5,2E-09	0,0010	0,0287	0,9723	instrument
rs71531541	6	7066481	C	T	0,0002	30,1727	0,0200	0,0036	4,0E-08	-0,0264	0,0331	0,4259	instrument
rs7155279	14	92485881	T	G	0,0003	47,9967	0,0233	0,0034	4,3E-12	0,0648	0,0297	0,0292	instrument

rs7170686	15	48821350	A	G	0,0002	36,2761	0,0334	0,0055	1,7E-09	0,0567	0,0494	0,2506	instrument
rs7191697	16	89678165	A	G	0,0002	46,8946	0,0369	0,0054	7,5E-12	-0,0222	0,0522	0,6711	instrument
rs7234167	18	46744610	C	G	0,0002	30,4480	0,0206	0,0037	3,4E-08	0,0358	0,0327	0,2741	instrument
rs7244464	18	20729714	A	C	0,0005	90,7247	0,0372	0,0039	1,7E-21	0,0498	0,0349	0,1539	instrument
rs72708236	1	155980320	G	T	0,0003	54,7890	0,0703	0,0095	1,3E-13	-0,0768	0,0905	0,3964	instrument
rs72755233	15	100692953	A	G	0,0008	160,7807	0,0645	0,0051	7,6E-37	-0,1417	0,0577	0,0140	instrument
rs72776438	5	77438797	T	G	0,0002	36,1798	0,0228	0,0038	1,8E-09	-0,0066	0,0356	0,8523	instrument
rs73004967	19	19717056	G	A	0,0002	40,6530	0,0405	0,0064	1,8E-10	0,0057	0,0587	0,9224	instrument
rs73175572	3	185490184	G	A	0,0002	36,0236	-0,0314	0,0052	2,0E-09	0,0194	0,0473	0,6818	instrument
rs732563	8	23345526	T	C	0,0002	41,4088	0,0208	0,0032	1,2E-10	0,0201	0,0286	0,4827	instrument
rs73407643	6	35169123	C	T	0,0002	36,6869	0,0751	0,0124	1,4E-09	-0,0024	0,1124	0,9828	instrument
rs741207	4	18321092	T	C	0,0002	35,8677	-0,0200	0,0033	2,1E-09	-0,0515	0,0293	0,0782	instrument
rs7502307	17	65989961	G	C	0,0003	54,1543	-0,0300	0,0041	1,9E-13	0,0186	0,0357	0,6034	instrument
rs75223147	6	19831020	G	T	0,0003	56,7154	-0,0848	0,0113	5,1E-14	-0,2370	0,1510	0,1165	instrument
rs75621580	4	12916514	A	G	0,0002	30,7689	0,0425	0,0077	2,9E-08	-0,0067	0,0730	0,9264	instrument
rs7626449	3	70921329	A	G	0,0002	39,4254	-0,0225	0,0036	3,4E-10	0,0096	0,0315	0,7610	instrument
rs7641375	3	57568444	A	C	0,0002	43,0725	-0,0219	0,0033	5,3E-11	-0,0188	0,0299	0,5301	instrument
rs7689420	4	145568352	T	C	0,0008	162,5608	0,0548	0,0043	3,4E-37	0,0562	0,0381	0,1409	instrument
rs7697556	4	73515313	T	C	0,0005	96,6869	-0,0318	0,0032	8,2E-23	0,0349	0,0294	0,2361	instrument
rs7718768	5	178536089	A	G	0,0003	62,8525	-0,0271	0,0034	2,2E-15	-0,0034	0,0307	0,9128	instrument
rs7733331	5	32828846	T	C	0,0002	34,2221	-0,0193	0,0033	4,9E-09	0,0160	0,0301	0,5945	instrument
rs7741741	6	142655801	A	T	0,0005	102,6158	0,0362	0,0036	4,2E-24	0,0268	0,0317	0,3981	instrument
rs7752292	6	131317876	A	T	0,0002	42,4187	-0,0218	0,0033	7,4E-11	0,0034	0,0297	0,9095	instrument
rs7804293	7	92287849	G	T	0,0004	73,9248	-0,0315	0,0037	8,1E-18	-0,0130	0,0323	0,6879	instrument
rs78198962	2	233094868	T	C	0,0002	29,7349	-0,0499	0,0092	5,0E-08	-0,1154	0,1018	0,2567	instrument
rs78821460	5	127874149	C	G	0,0002	32,3189	0,0330	0,0058	1,3E-08	0,0648	0,0510	0,2038	instrument
rs79312203	20	32082962	A	G	0,0002	36,7596	-0,0326	0,0054	1,3E-09	-0,0172	0,0483	0,7220	instrument
rs7952436	11	67024534	T	C	0,0006	119,9012	0,0637	0,0058	6,7E-28	-0,0092	0,0629	0,8840	instrument
rs7994151	13	115038879	G	A	0,0002	41,0364	0,0289	0,0045	1,5E-10	-0,0249	0,0404	0,5385	instrument
rs8003703	14	33411639	A	T	0,0002	31,7078	0,0642	0,0114	1,8E-08	-0,2034	0,1184	0,0859	instrument
rs80085026	7	148636421	G	C	0,0003	55,0558	-0,0308	0,0042	1,2E-13	0,0531	0,0370	0,1511	instrument

rs8103728	19	33900257	C	G	0,0004	82,6818	0,0313	0,0034	9,7E-20	-0,0506	0,0306	0,0982	instrument
rs832540	5	56199202	G	A	0,0002	32,1826	0,0193	0,0034	1,4E-08	0,0405	0,0299	0,1752	instrument
rs849141	7	28185091	A	G	0,0003	66,0149	-0,0289	0,0036	4,5E-16	0,0494	0,0317	0,1189	instrument
rs862041	14	74986403	T	C	0,0003	50,5374	0,0240	0,0034	1,2E-12	-0,0022	0,0302	0,9411	instrument
rs9346545	6	168806788	C	A	0,0002	29,9315	0,0197	0,0036	4,5E-08	-0,0461	0,0321	0,1503	instrument
rs9358912	6	26211146	T	G	0,0012	232,2552	0,0554	0,0036	2,0E-52	0,0317	0,0313	0,3097	instrument
rs9391253	6	105367616	T	A	0,0005	93,9339	-0,0335	0,0035	3,3E-22	0,0185	0,0306	0,5452	instrument
rs9398433	6	116753939	G	T	0,0002	43,0200	-0,0219	0,0033	5,4E-11	-0,0073	0,0293	0,8043	instrument
rs9442571	1	9349611	A	T	0,0002	41,2288	-0,0313	0,0049	1,4E-10	-0,0117	0,0457	0,7981	instrument
rs9496567	6	100602753	A	G	0,0002	30,3267	-0,0208	0,0038	3,7E-08	-0,0099	0,0362	0,7845	instrument
rs9523325	13	92051677	T	G	0,0002	39,1496	-0,0258	0,0041	3,9E-10	0,0119	0,0357	0,7382	instrument
rs9614671	22	45839056	A	G	0,0002	29,7676	-0,0221	0,0040	4,9E-08	0,0546	0,0359	0,1282	instrument
rs979131	20	6566965	C	T	0,0002	33,8953	-0,0199	0,0034	5,8E-09	-0,0730	0,0301	0,0153	instrument
rs9809116	3	72397279	G	A	0,0002	35,6763	0,0199	0,0033	2,3E-09	0,0292	0,0298	0,3284	instrument
rs994014	4	82165790	C	T	0,0005	90,3251	-0,0333	0,0035	2,0E-21	-0,0729	0,0313	0,0200	instrument
rs9943184	1	170784430	T	G	0,0002	36,8809	-0,0294	0,0048	1,3E-09	0,0122	0,0428	0,7761	instrument
rs9965184	18	40776606	T	A	0,0002	33,1657	0,0196	0,0034	8,5E-09	-0,0565	0,0305	0,0635	instrument

Table S34. Single Nucleotide Polymorphisms (SNPs) used as instruments in the Mendelian randomization analyses for the effect of LFR on high or low serous ovarian cancer based on the genome-wide significance threshold P = 5E-08. R^2 represents the explained variance in the exposure. SNP-specific instrument strength is given by the respective F-statistic. Some of the genetic variants were identified as outliers in the iterative approach and therefore excluded from the analyses.

SNP	CHR	POS	EA	OA	R2	Fstat	β_{LFR}	SE_{LFR}	P_{LFR}	$\beta_{High\ low\ serous}$	$S_{High\ low\ serous}$	$P_{High\ low\ serous}$	$variant_{High\ low\ serous}$
rs1002436	1	89146852	A	G	0,0002	33,9070	0,0189	0,0032	5,8E-09	0,0044	0,0155	0,7764	instrument
rs1007358	7	46201355	G	A	0,0002	31,5391	-0,0221	0,0039	2,0E-08	-0,0109	0,0189	0,5645	instrument
rs10172196	2	36780549	A	G	0,0003	53,8017	-0,0259	0,0035	2,2E-13	-0,0116	0,0168	0,4909	instrument
rs1020048	9	86666993	A	C	0,0002	40,1825	-0,0265	0,0042	2,3E-10	-0,0150	0,0194	0,4400	instrument
rs10445034	16	88840564	T	C	0,0002	38,3776	0,0239	0,0039	5,8E-10	-0,0015	0,0182	0,9343	instrument
rs11205303	1	149906413	C	T	0,0018	340,7680	-0,0605	0,0033	4,8E-76	-0,0170	0,0156	0,2760	instrument
rs112540634	6	34623905	T	C	0,0006	106,9145	-0,0487	0,0047	4,7E-25	0,0226	0,0217	0,2983	instrument
rs11259936	15	84580582	A	C	0,0012	235,0064	0,0495	0,0032	5,0E-53	-0,0138	0,0152	0,3622	instrument
rs112635299	14	94838142	T	G	0,0004	70,8115	-0,0942	0,0112	3,9E-17	0,0182	0,0574	0,7513	instrument

rs113059026	10	32158478	A	C	0,0002	42,1586	0,0233	0,0036	8,4E-11	0,0008	0,0168	0,9602	instrument
rs11466399	1	218591623	A	G	0,0003	63,9993	-0,0287	0,0036	1,3E-15	0,0112	0,0169	0,5089	instrument
rs11666808	19	18383506	T	C	0,0002	46,9631	-0,0230	0,0033	7,2E-12	0,0151	0,0160	0,3464	instrument
rs11695471	2	25457708	A	T	0,0005	88,6790	0,0324	0,0034	4,7E-21	-0,0152	0,0168	0,3660	instrument
rs12103006	16	24726237	A	G	0,0002	31,2925	0,0183	0,0033	2,2E-08	0,0046	0,0154	0,7637	instrument
rs12238852	9	98382084	T	C	0,0002	40,8700	-0,0296	0,0046	1,6E-10	-0,0396	0,0222	0,0738	instrument
rs12756815	1	113207243	A	C	0,0003	62,4410	0,0316	0,0040	2,7E-15	-0,0091	0,0189	0,6303	instrument
rs13128180	4	56286188	T	G	0,0003	57,4558	-0,0267	0,0035	3,5E-14	0,0367	0,0164	0,0255	instrument
rs1313237	14	60848224	C	A	0,0003	50,8506	-0,0236	0,0033	1,0E-12	-0,0194	0,0157	0,2176	instrument
rs132941	22	38545942	C	T	0,0003	64,2716	-0,0261	0,0033	1,1E-15	-0,0032	0,0153	0,8337	instrument
rs13306455	19	7184721	T	C	0,0002	32,1259	-0,0244	0,0043	1,4E-08	-0,0156	0,0204	0,4440	instrument
rs138809270	2	219929394	T	C	0,0002	39,7778	0,0828	0,0131	2,8E-10	-0,0857	0,0606	0,1572	instrument
rs143384	20	34025756	G	A	0,0009	179,8262	-0,0441	0,0033	5,4E-41	0,0034	0,0155	0,8268	instrument
rs143840904	11	2813322	T	C	0,0002	44,7289	0,0842	0,0126	2,3E-11	-0,1142	0,0762	0,1340	instrument
rs1452815	8	78123634	G	A	0,0002	30,3818	-0,0197	0,0036	3,6E-08	-0,0146	0,0170	0,3899	instrument
rs1513304	3	99269750	C	T	0,0002	40,5765	-0,0226	0,0035	1,9E-10	0,0079	0,0178	0,6549	instrument
rs1550436	15	74221157	T	C	0,0004	77,3160	0,0286	0,0032	1,5E-18	0,0037	0,0152	0,8064	instrument
rs16942324	15	89383854	A	C	0,0004	70,6937	0,0844	0,0100	4,2E-17	-0,0287	0,0513	0,5765	instrument
rs16948048	17	47440466	G	A	0,0002	42,5621	0,0218	0,0033	6,9E-11	-0,0287	0,0157	0,0675	instrument
rs16953461	17	46261736	G	T	0,0003	54,8186	0,0292	0,0039	1,3E-13	-0,0468	0,0193	0,0153	instrument
rs16972537	16	73802389	A	G	0,0002	33,0736	-0,0194	0,0034	8,9E-09	0,0152	0,0162	0,3473	instrument
rs17698176	17	44819595	G	T	0,0003	62,1883	-0,0307	0,0039	3,1E-15	-0,0302	0,0207	0,1447	instrument
rs17744040	9	16842137	C	T	0,0002	34,5740	0,0309	0,0053	4,1E-09	-0,1630	0,0241	8,9E-12	outlier
rs1801282	3	12393125	G	C	0,0003	51,5519	-0,0356	0,0050	7,0E-13	0,0152	0,0230	0,5079	instrument
rs1980030	10	120970027	G	A	0,0002	32,0806	-0,0186	0,0033	1,5E-08	-0,0145	0,0159	0,3641	instrument
rs2007482	6	2169724	T	C	0,0002	36,6626	0,0199	0,0033	1,4E-09	-0,0045	0,0159	0,7784	instrument
rs2058908	16	53806145	T	C	0,0002	44,3951	0,0243	0,0037	2,7E-11	-0,0117	0,0176	0,5050	instrument
rs2126643	1	103404384	T	C	0,0002	40,1318	-0,0218	0,0034	2,4E-10	0,0043	0,0160	0,7880	instrument
rs2158694	7	2863443	G	T	0,0008	161,7967	0,0450	0,0035	4,5E-37	-0,0169	0,0168	0,3139	instrument
rs2173295	1	54943264	C	A	0,0002	33,0737	0,0276	0,0048	8,9E-09	-0,0268	0,0240	0,2644	instrument
rs2274432	1	184020945	A	G	0,0005	86,6007	-0,0315	0,0034	1,3E-20	0,0101	0,0161	0,5293	instrument

rs2293576	11	47434986	A	G	0,0003	48,9295	0,0240	0,0034	2,7E-12	0,0071	0,0161	0,6612	instrument
rs2370983	14	79903376	G	A	0,0002	33,4890	0,0196	0,0034	7,2E-09	0,0114	0,0165	0,4870	instrument
rs2516633	11	62371773	A	C	0,0002	31,4718	0,0197	0,0035	2,0E-08	-0,0198	0,0174	0,2558	instrument
rs256904	5	55810305	A	T	0,0003	66,6012	-0,0302	0,0037	3,3E-16	0,0183	0,0177	0,3012	instrument
rs2641699	16	84886306	C	G	0,0002	35,6047	0,0200	0,0034	2,4E-09	0,0010	0,0164	0,9511	instrument
rs264762	5	64384386	A	C	0,0003	52,4171	0,0248	0,0034	4,5E-13	-0,0079	0,0162	0,6256	instrument
rs2712169	2	217671349	G	A	0,0003	49,6879	-0,0233	0,0033	1,8E-12	0,0174	0,0155	0,2616	instrument
rs2715094	7	50730452	G	A	0,0003	57,7290	-0,0282	0,0037	3,0E-14	0,0246	0,0181	0,1741	instrument
rs2768641	9	96948863	G	A	0,0002	34,4213	-0,0455	0,0078	4,4E-09	0,0165	0,0378	0,6631	instrument
rs2811462	3	129303436	G	T	0,0003	52,3447	-0,0281	0,0039	4,7E-13	0,0237	0,0186	0,2035	instrument
rs2820446	1	219748818	G	C	0,0007	132,7090	-0,0405	0,0035	1,1E-30	0,0015	0,0170	0,9290	instrument
rs2844795	6	30073847	C	T	0,0002	41,4345	-0,0208	0,0032	1,2E-10	0,0023	0,0155	0,8818	instrument
rs28539246	17	29226630	A	T	0,0003	48,5804	0,0231	0,0033	3,2E-12	-0,0437	0,0156	0,0051	outlier
rs28716235	8	135602976	C	A	0,0002	33,4427	0,0194	0,0034	7,4E-09	-0,0247	0,0159	0,1189	instrument
rs2886564	1	42496924	G	A	0,0002	31,4381	-0,0200	0,0036	2,1E-08	-0,0107	0,0168	0,5217	instrument
rs3020421	6	152348122	A	G	0,0002	35,0224	-0,0206	0,0035	3,3E-09	0,0222	0,0163	0,1732	instrument
rs3218036	19	30305684	A	G	0,0002	29,8550	-0,0188	0,0034	4,7E-08	0,0582	0,0161	0,0003	outlier
rs34517439	1	78450517	A	C	0,0003	65,2049	-0,0403	0,0050	6,8E-16	0,0075	0,0249	0,7636	instrument
rs34580448	5	82810884	C	T	0,0004	79,5477	0,0727	0,0081	4,8E-19	0,0265	0,0393	0,5002	instrument
rs35307904	9	78511889	A	G	0,0006	116,4229	0,0537	0,0050	3,8E-27	-0,0195	0,0252	0,4386	instrument
rs35874463	15	67457698	G	A	0,0003	66,0962	-0,0563	0,0069	4,3E-16	-0,0322	0,0347	0,3532	instrument
rs3738814	1	17331676	G	A	0,0003	60,4500	0,0253	0,0033	7,6E-15	-0,0235	0,0154	0,1254	instrument
rs3791679	2	56096892	G	A	0,0009	179,2902	0,0516	0,0039	7,3E-41	0,0207	0,0178	0,2437	instrument
rs3794782	17	18127914	C	G	0,0002	33,0277	-0,0191	0,0033	9,1E-09	-0,0312	0,0157	0,0475	instrument
rs3823974	7	20442796	C	T	0,0003	60,9486	0,0258	0,0033	5,9E-15	0,0058	0,0154	0,7066	instrument
rs3850639	1	172253634	C	T	0,0003	56,8058	-0,0297	0,0039	4,8E-14	-0,0010	0,0186	0,9552	instrument
rs4073717	5	170864021	T	G	0,0002	33,2233	0,0233	0,0040	8,2E-09	0,0261	0,0192	0,1738	instrument
rs41284816	13	50655989	T	G	0,0002	46,3484	-0,0835	0,0123	9,9E-12	-0,0344	0,0528	0,5149	instrument
rs41313250	1	62483623	G	A	0,0002	36,9903	0,0319	0,0052	1,2E-09	-0,0269	0,0234	0,2516	instrument
rs4236324	7	27302727	C	T	0,0002	37,5030	-0,0274	0,0045	9,1E-10	-0,0238	0,0214	0,2656	instrument
rs4246308	15	100765202	A	G	0,0003	59,4589	0,0250	0,0032	1,3E-14	0,0194	0,0152	0,2016	instrument

rs4321401	2	69348216	T	C	0,0003	48,9715	0,0227	0,0032	2,6E-12	0,0217	0,0165	0,1886	instrument
rs4421120	5	32763118	A	G	0,0003	50,1542	-0,0311	0,0044	1,4E-12	-0,0086	0,0214	0,6866	instrument
rs4432932	5	115039414	G	A	0,0002	31,1249	-0,0204	0,0037	2,4E-08	0,0049	0,0169	0,7708	instrument
rs4518624	8	130715510	A	G	0,0002	38,0685	0,0203	0,0033	6,8E-10	0,0074	0,0156	0,6331	instrument
rs454345	5	88729851	G	A	0,0002	33,8255	-0,0194	0,0033	6,0E-09	-0,0017	0,0159	0,9125	instrument
rs4803461	19	41908871	G	A	0,0002	33,2003	-0,0190	0,0033	8,3E-09	-0,0015	0,0158	0,9230	instrument
rs4842133	9	139127566	A	C	0,0004	69,4715	-0,0300	0,0036	7,8E-17	-0,0172	0,0176	0,3269	instrument
rs4901541	14	55235282	C	T	0,0002	40,4873	0,0211	0,0033	2,0E-10	0,0076	0,0163	0,6417	instrument
rs4936122	11	130734264	C	A	0,0002	30,3818	-0,0181	0,0033	3,6E-08	-0,0139	0,0158	0,3772	instrument
rs496298	10	131355130	A	G	0,0003	65,4151	0,0276	0,0034	6,1E-16	-0,0370	0,0161	0,0220	instrument
rs4973032	2	232990462	T	G	0,0002	40,7423	0,0769	0,0121	1,7E-10	-0,0736	0,0552	0,1820	instrument
rs4974072	3	41222586	A	G	0,0002	43,3091	-0,0214	0,0033	4,7E-11	-0,0137	0,0153	0,3705	instrument
rs538656	18	57850422	T	G	0,0002	31,4269	-0,0213	0,0038	2,1E-08	-0,0125	0,0180	0,4849	instrument
rs544148	6	117466494	A	G	0,0002	30,7134	0,0182	0,0033	3,0E-08	0,0144	0,0155	0,3545	instrument
rs55633655	5	131598756	T	G	0,0003	53,1144	0,0237	0,0033	3,2E-13	-0,0227	0,0153	0,1381	instrument
rs56021161	14	64817100	A	T	0,0002	30,5916	0,0362	0,0065	3,2E-08	0,0067	0,0317	0,8335	instrument
rs56186137	16	28825953	G	A	0,0006	108,1589	-0,0341	0,0033	2,6E-25	-0,0054	0,0157	0,7332	instrument
rs56277550	21	18092263	A	G	0,0002	33,5006	0,0187	0,0032	7,1E-09	-0,0126	0,0155	0,4176	instrument
rs57674902	20	4096741	T	C	0,0002	38,1302	-0,0210	0,0034	6,6E-10	-0,0108	0,0166	0,5170	instrument
rs584961	11	75277628	A	G	0,0002	35,1408	-0,0302	0,0051	3,1E-09	0,0014	0,0255	0,9553	instrument
rs61734651	20	61451332	T	C	0,0002	37,8836	-0,0421	0,0068	7,5E-10	-0,0087	0,0350	0,8025	instrument
rs61834590	1	227421117	G	T	0,0002	43,2696	0,0298	0,0045	4,8E-11	-0,0171	0,0216	0,4266	instrument
rs62106258	2	417167	C	T	0,0002	32,2053	0,0424	0,0075	1,4E-08	-0,0130	0,0414	0,7538	instrument
rs62271373	3	150066540	A	T	0,0002	41,1775	0,0455	0,0071	1,4E-10	-0,0393	0,0359	0,2731	instrument
rs62515452	8	57187356	T	C	0,0002	34,2338	-0,0267	0,0046	4,9E-09	0,0030	0,0233	0,8978	instrument
rs62621197	19	8670147	T	C	0,0008	150,7968	0,1140	0,0093	1,1E-34	0,0262	0,0517	0,6119	instrument
rs640245	11	30393360	T	C	0,0002	37,0512	-0,0198	0,0033	1,1E-09	-0,0145	0,0154	0,3463	instrument
rs6486059	11	12824581	A	G	0,0003	52,4315	-0,0238	0,0033	4,5E-13	-0,0093	0,0157	0,5565	instrument
rs6503410	17	43094140	T	C	0,0002	35,0934	0,0215	0,0036	3,2E-09	0,0420	0,0173	0,0154	instrument
rs6585827	10	124165615	A	G	0,0002	45,0505	-0,0217	0,0032	1,9E-11	0,0207	0,0154	0,1803	instrument
rs6717858	2	165539661	C	T	0,0003	64,7696	-0,0265	0,0033	8,5E-16	-0,0271	0,0156	0,0818	instrument

rs6739701	2	105132332	G	A	0,0002	44,8493	-0,0220	0,0033	2,1E-11	-0,0206	0,0162	0,2041	instrument
rs6785012	3	141109348	T	C	0,0012	231,9505	-0,0496	0,0033	2,3E-52	-0,0149	0,0154	0,3336	instrument
rs68049170	10	72432047	A	G	0,0003	56,1594	-0,0273	0,0036	6,7E-14	0,0003	0,0173	0,9841	instrument
rs6843340	4	48619937	T	C	0,0002	30,9912	-0,0180	0,0032	2,6E-08	0,0134	0,0153	0,3804	instrument
rs6887247	5	157881647	C	T	0,0003	53,8897	-0,0257	0,0035	2,1E-13	0,0133	0,0168	0,4296	instrument
rs702605	5	53167028	T	C	0,0002	34,1286	-0,0189	0,0032	5,2E-09	-0,0090	0,0153	0,5570	instrument
rs71531541	6	7066481	C	T	0,0002	30,1727	0,0200	0,0036	4,0E-08	0,0097	0,0175	0,5797	instrument
rs7155279	14	92485881	T	G	0,0003	47,9967	0,0233	0,0034	4,3E-12	-0,0055	0,0159	0,7309	instrument
rs7170686	15	48821350	A	G	0,0002	36,2761	0,0334	0,0055	1,7E-09	-0,0095	0,0266	0,7215	instrument
rs7191697	16	89678165	A	G	0,0002	46,8946	0,0369	0,0054	7,5E-12	0,0026	0,0278	0,9258	instrument
rs7234167	18	46744610	C	G	0,0002	30,4480	0,0206	0,0037	3,4E-08	4,9E-05	0,0175	0,9978	instrument
rs7244464	18	20729714	A	C	0,0005	90,7247	0,0372	0,0039	1,7E-21	-0,0550	0,0189	0,0036	outlier
rs72708236	1	155980320	G	T	0,0003	54,7890	0,0703	0,0095	1,3E-13	-0,0161	0,0476	0,7358	instrument
rs72755233	15	100692953	A	G	0,0008	160,7807	0,0645	0,0051	7,6E-37	-0,0173	0,0300	0,5644	instrument
rs72776438	5	77438797	T	G	0,0002	36,1798	0,0228	0,0038	1,8E-09	0,0109	0,0190	0,5659	instrument
rs73004967	19	19717056	G	A	0,0002	40,6530	0,0405	0,0064	1,8E-10	0,0441	0,0310	0,1552	instrument
rs73175572	3	185490184	G	A	0,0002	36,0236	-0,0314	0,0052	2,0E-09	-0,0141	0,0253	0,5769	instrument
rs732563	8	23345526	T	C	0,0002	41,4088	0,0208	0,0032	1,2E-10	-0,0021	0,0152	0,8904	instrument
rs73407643	6	35169123	C	T	0,0002	36,6869	0,0751	0,0124	1,4E-09	0,0218	0,0591	0,7126	instrument
rs741207	4	18321092	T	C	0,0002	35,8677	-0,0200	0,0033	2,1E-09	-0,0118	0,0156	0,4495	instrument
rs7502307	17	65989961	G	C	0,0003	54,1543	-0,0300	0,0041	1,9E-13	-0,0310	0,0192	0,1058	instrument
rs75223147	6	19831020	G	T	0,0003	56,7154	-0,0848	0,0113	5,1E-14	0,0785	0,0756	0,2993	instrument
rs75621580	4	12916514	A	G	0,0002	30,7689	0,0425	0,0077	2,9E-08	0,0358	0,0388	0,3561	instrument
rs7626449	3	70921329	A	G	0,0002	39,4254	-0,0225	0,0036	3,4E-10	-0,0083	0,0168	0,6201	instrument
rs7641375	3	57568444	A	C	0,0002	43,0725	-0,0219	0,0033	5,3E-11	-0,0120	0,0159	0,4509	instrument
rs7689420	4	145568352	T	C	0,0008	162,5608	0,0548	0,0043	3,4E-37	0,0074	0,0206	0,7208	instrument
rs7697556	4	73515313	T	C	0,0005	96,6869	-0,0318	0,0032	8,2E-23	-0,0079	0,0156	0,6117	instrument
rs7718768	5	178536089	A	G	0,0003	62,8525	-0,0271	0,0034	2,2E-15	-0,0173	0,0164	0,2929	instrument
rs7733331	5	32828846	T	C	0,0002	34,2221	-0,0193	0,0033	4,9E-09	-0,0209	0,0160	0,1920	instrument
rs7741741	6	142655801	A	T	0,0005	102,6158	0,0362	0,0036	4,2E-24	0,0217	0,0169	0,1992	instrument
rs7752292	6	131317876	A	T	0,0002	42,4187	-0,0218	0,0033	7,4E-11	0,0145	0,0158	0,3601	instrument

rs7804293	7	92287849	G	T	0,0004	73,9248	-0,0315	0,0037	8,1E-18	0,0254	0,0171	0,1364	instrument
rs78198962	2	233094868	T	C	0,0002	29,7349	-0,0499	0,0092	5,0E-08	-0,0593	0,0535	0,2681	instrument
rs78821460	5	127874149	C	G	0,0002	32,3189	0,0330	0,0058	1,3E-08	0,0041	0,0275	0,8821	instrument
rs79312203	20	32082962	A	G	0,0002	36,7596	-0,0326	0,0054	1,3E-09	-0,0083	0,0256	0,7448	instrument
rs7952436	11	67024534	T	C	0,0006	119,9012	0,0637	0,0058	6,7E-28	-0,0172	0,0337	0,6090	instrument
rs7994151	13	115038879	G	A	0,0002	41,0364	0,0289	0,0045	1,5E-10	-0,0328	0,0215	0,1271	instrument
rs8003703	14	33411639	A	T	0,0002	31,7078	0,0642	0,0114	1,8E-08	0,1097	0,0585	0,0606	instrument
rs80085026	7	148636421	G	C	0,0003	55,0558	-0,0308	0,0042	1,2E-13	-0,0041	0,0200	0,8358	instrument
rs8103728	19	33900257	C	G	0,0004	82,6818	0,0313	0,0034	9,7E-20	0,0078	0,0162	0,6303	instrument
rs832540	5	56199202	G	A	0,0002	32,1826	0,0193	0,0034	1,4E-08	-0,0419	0,0160	0,0090	outlier
rs849141	7	28185091	A	G	0,0003	66,0149	-0,0289	0,0036	4,5E-16	-0,0346	0,0170	0,0423	instrument
rs862041	14	74986403	T	C	0,0003	50,5374	0,0240	0,0034	1,2E-12	0,0029	0,0161	0,8556	instrument
rs9346545	6	168806788	C	A	0,0002	29,9315	0,0197	0,0036	4,5E-08	0,0019	0,0170	0,9091	instrument
rs9358912	6	26211146	T	G	0,0012	232,2552	0,0554	0,0036	2,0E-52	-0,0152	0,0167	0,3640	instrument
rs9391253	6	105367616	T	A	0,0005	93,9339	-0,0335	0,0035	3,3E-22	0,0136	0,0164	0,4046	instrument
rs9398433	6	116753939	G	T	0,0002	43,0200	-0,0219	0,0033	5,4E-11	0,0086	0,0156	0,5828	instrument
rs9442571	1	9349611	A	T	0,0002	41,2288	-0,0313	0,0049	1,4E-10	-0,0134	0,0244	0,5821	instrument
rs9496567	6	100602753	A	G	0,0002	30,3267	-0,0208	0,0038	3,7E-08	0,0239	0,0192	0,2118	instrument
rs9523325	13	92051677	T	G	0,0002	39,1496	-0,0258	0,0041	3,9E-10	0,0209	0,0190	0,2708	instrument
rs9614671	22	45839056	A	G	0,0002	29,7676	-0,0221	0,0040	4,9E-08	-0,0017	0,0193	0,9314	instrument
rs979131	20	6566965	C	T	0,0002	33,8953	-0,0199	0,0034	5,8E-09	-0,0051	0,0160	0,7472	instrument
rs9809116	3	72397279	G	A	0,0002	35,6763	0,0199	0,0033	2,3E-09	-0,0001	0,0159	0,9960	instrument
rs994014	4	82165790	C	T	0,0005	90,3251	-0,0333	0,0035	2,0E-21	-0,0034	0,0165	0,8388	instrument
rs9943184	1	170784430	T	G	0,0002	36,8809	-0,0294	0,0048	1,3E-09	0,0056	0,0228	0,8049	instrument
rs9965184	18	40776606	T	A	0,0002	33,1657	0,0196	0,0034	8,5E-09	0,0096	0,0161	0,5511	instrument
